# Supplementary material for: Discovery of Compounds That Selectively Repress the Amyloidogenic Processing of the Amyloid Precursor Protein: Design, Synthesis and Pharmacological Evaluation of Diphenylpyrazoles
Source: Int J Mol Sci. 2022 Oct 28;23(21):13111. doi: 10.3390/ijms232113111 (PMC9658513; doi:10.3390/ijms232113111)
Supplement: Supplementary file 1 [file ijms-23-13111-s001.zip › ijms-1952913-supplementary.pdf]

Discovery of Compounds that Selectively Repress the Amyloidogenic Processing of the Amyloid Precursor Protein : Design, Synthesis and Pharmacological Evaluation of Diphenylpyrazoles

Christophe Mesangeau [1], Pascal Carato [1,#], Nicolas Renault [2], Mathilde Coevoet [1], Paul-Emmanuel Larchanché [1], Amélie Barczyk [2], Luc Buée [1], Nicolas Sergeant [1], Patricia Melnyk [1,*]

[1] Univ. Lille, Inserm, CHU Lille, U1172 - LilNCog - Lille Neurosciences & Cognition, F-59000 Lille, France
[2] Univ. Lille, Inserm, CHU Lille, U1286 - INFINITE - Institute for Translational Research in Inflammation, F-59000 Lille, France
# Present address : Univ Poitiers, CIC Inserm 1402, F-86073 Poitiers, France
* Correspondence: patricia.melnyk@univ-lille.fr

Supporting information

^{1}H and ^{13}C NMR, MS spectra of compounds **5-72**

HPLC chromatograms of compounds **24-35** and **61-72**

1-[4-[3-(dimethylamino)propyl]phenyl]ethanone (**5**)

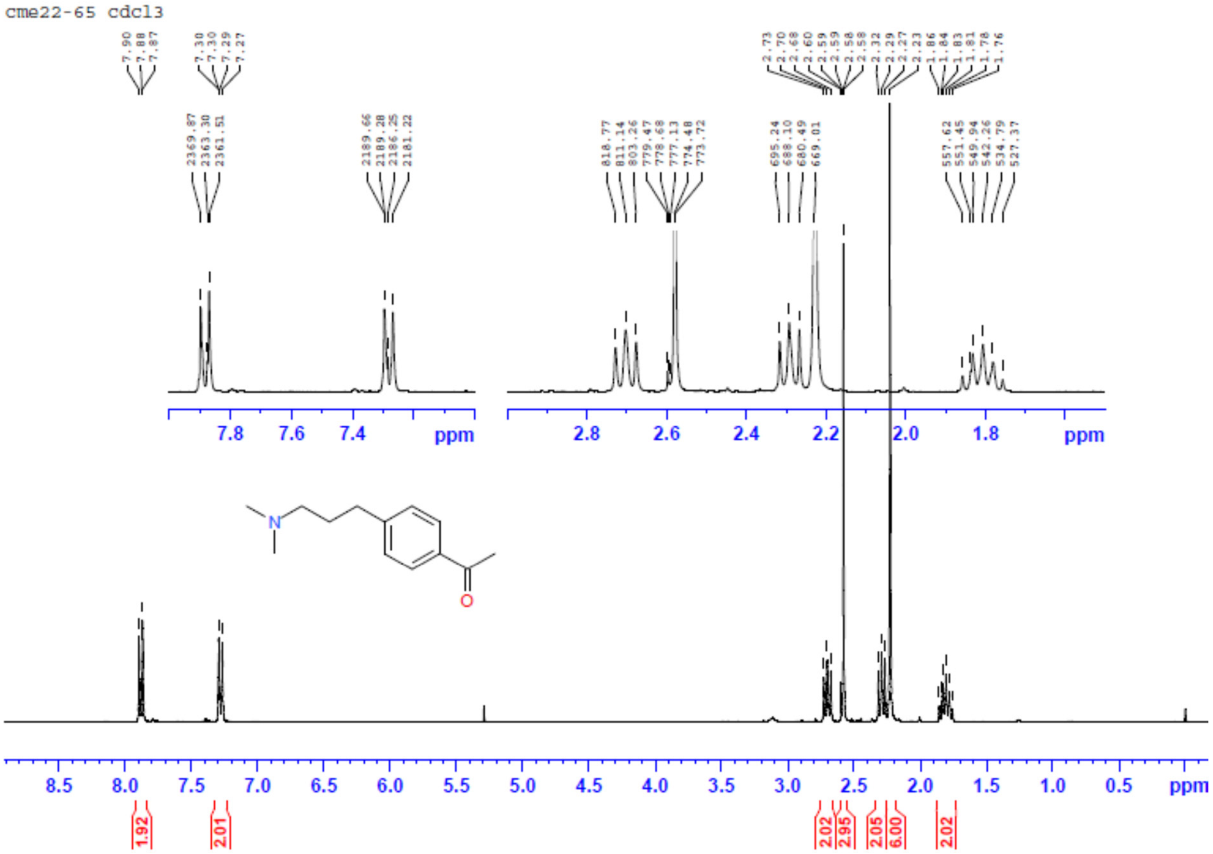

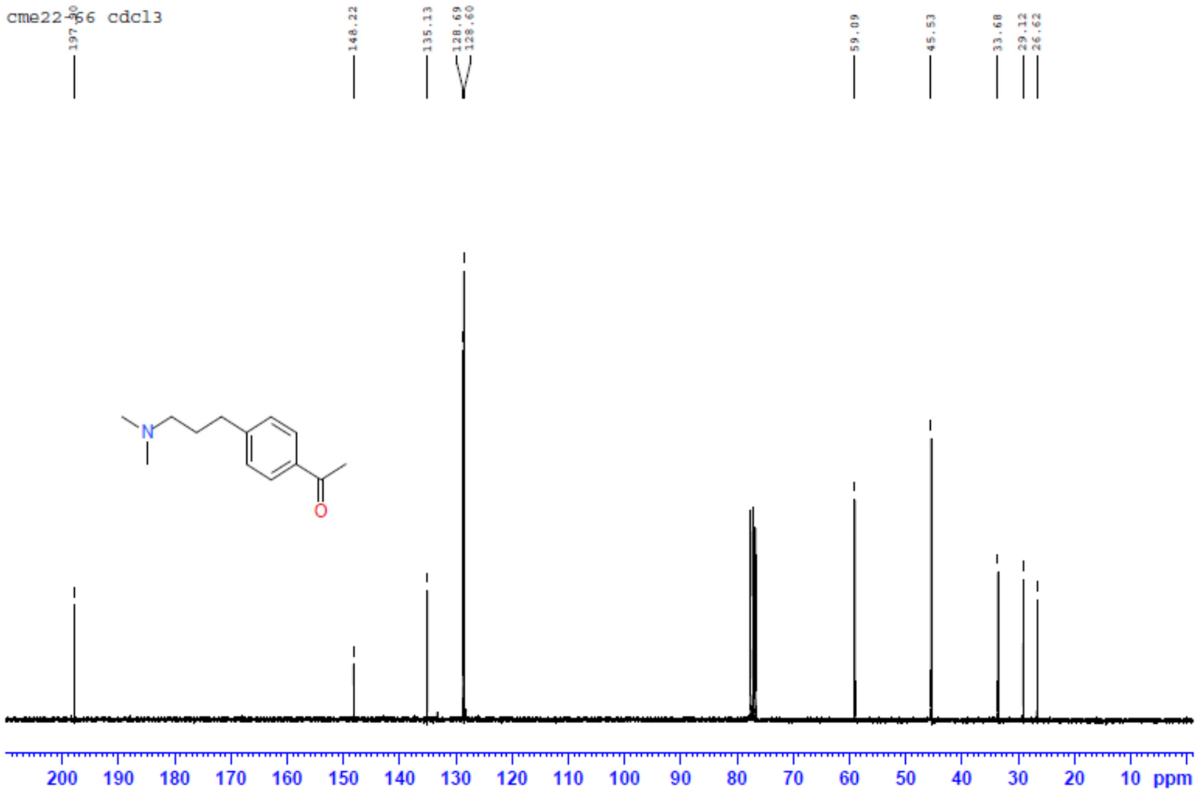

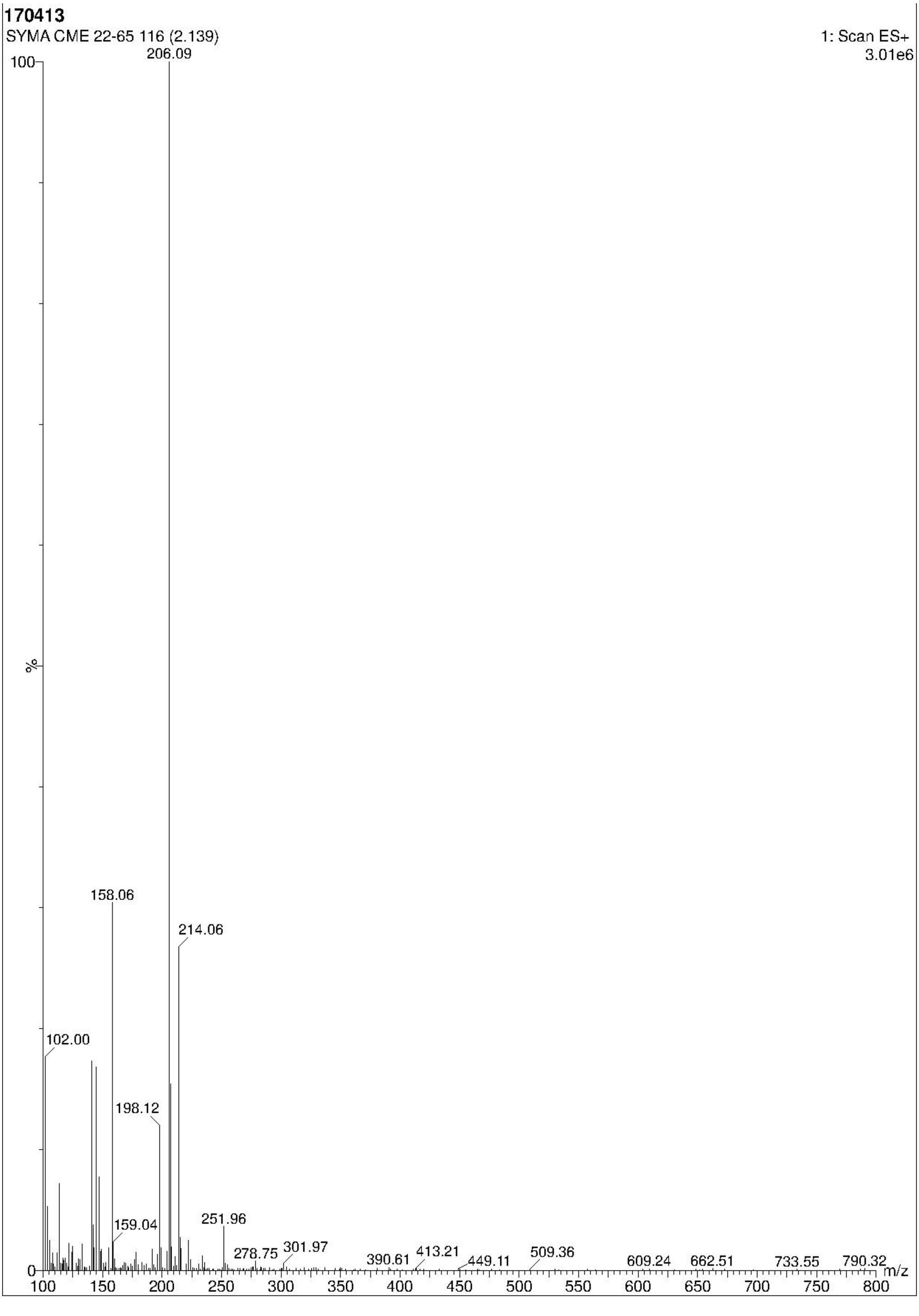

170413
SYMA CME 22-65 116 (2.139)
1: Scan ES+
3.01e6
206.09
158.06
214.06
102.00
198.12
159.04
251.96
278.75
301.97
390.61
413.21
449.11
509.36
609.24
662.51
733.55
790.32
%
m/z

methyl 4-[2-[1-[4-[3-(dimethylamino)propyl]phenyl] ethylidene]hydrazino]benzoate (**6**)

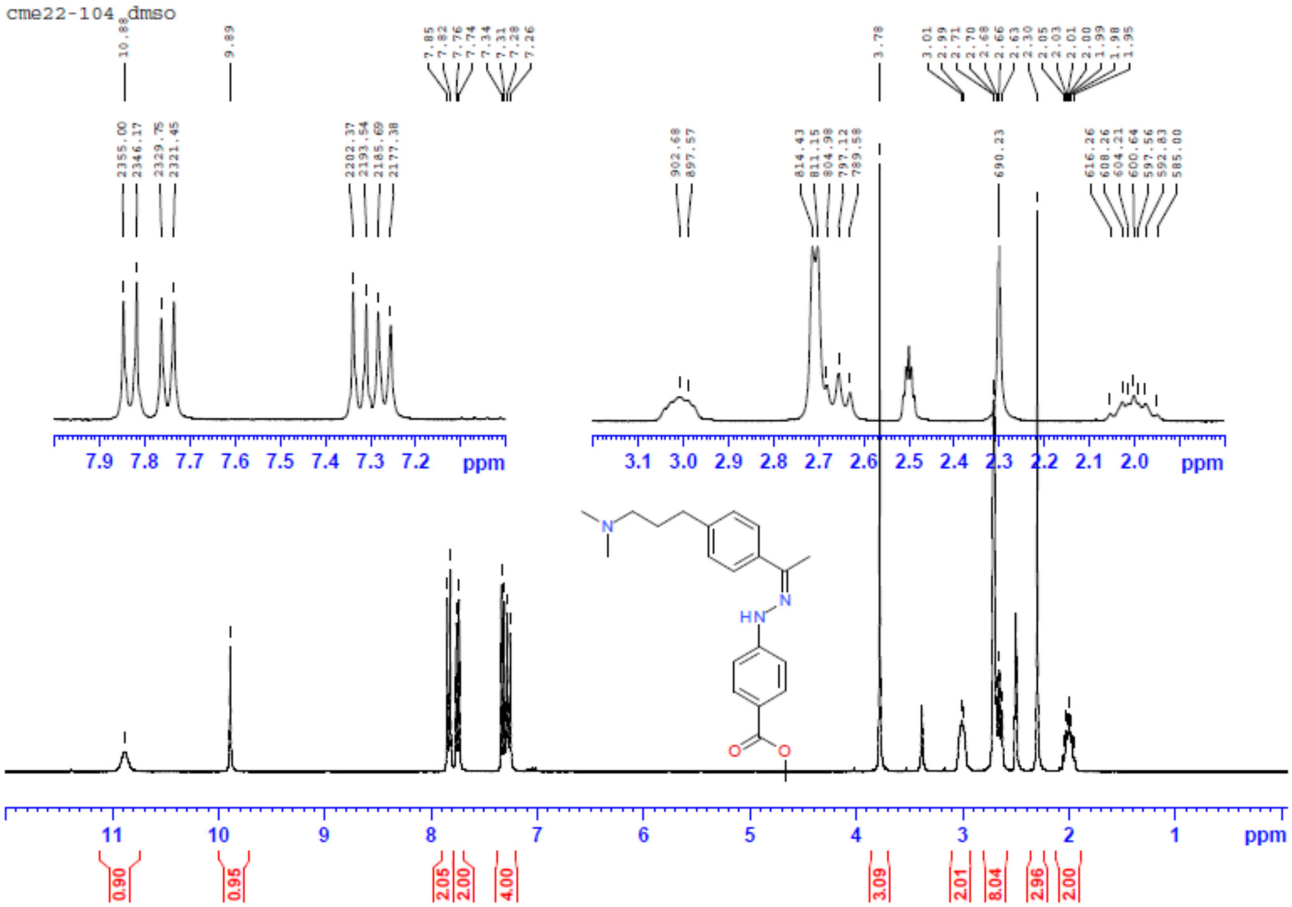

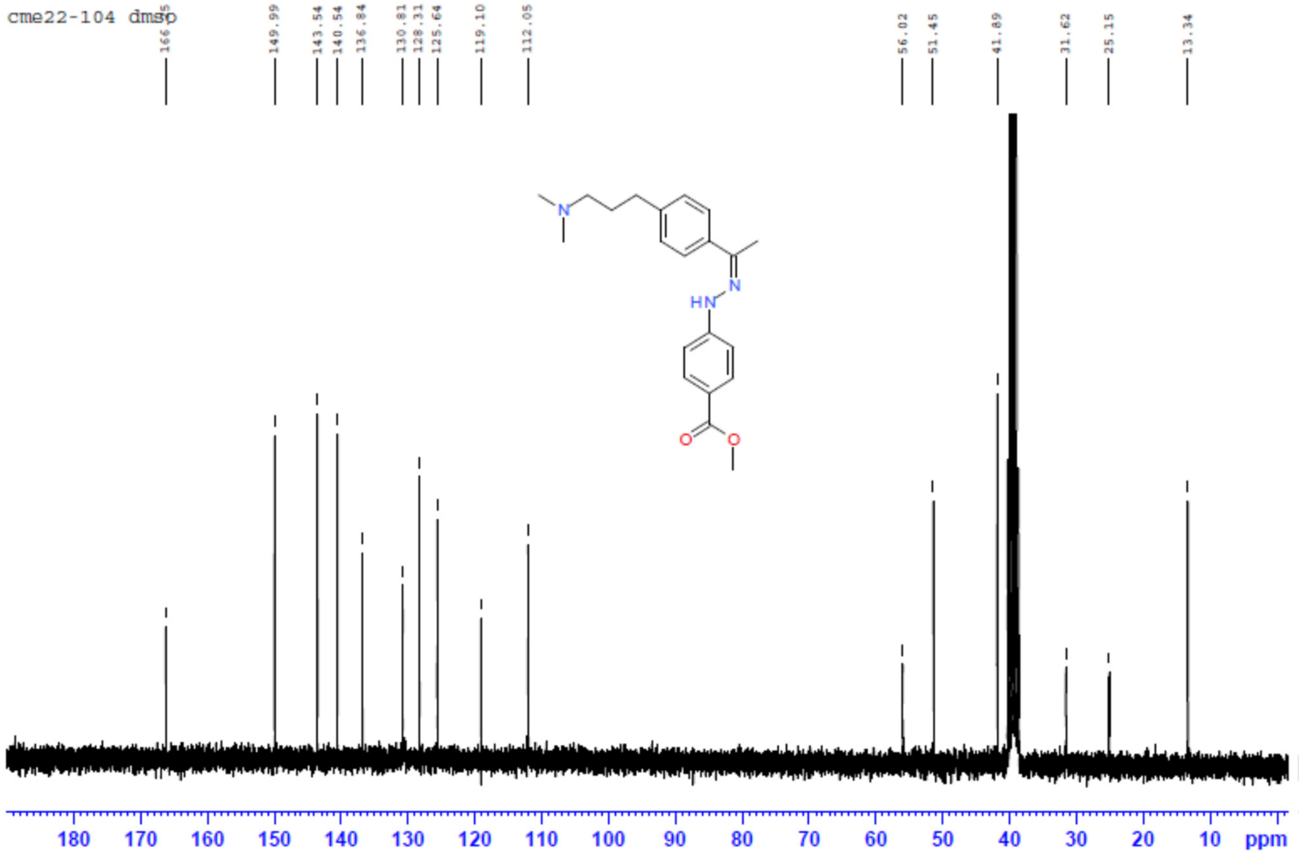

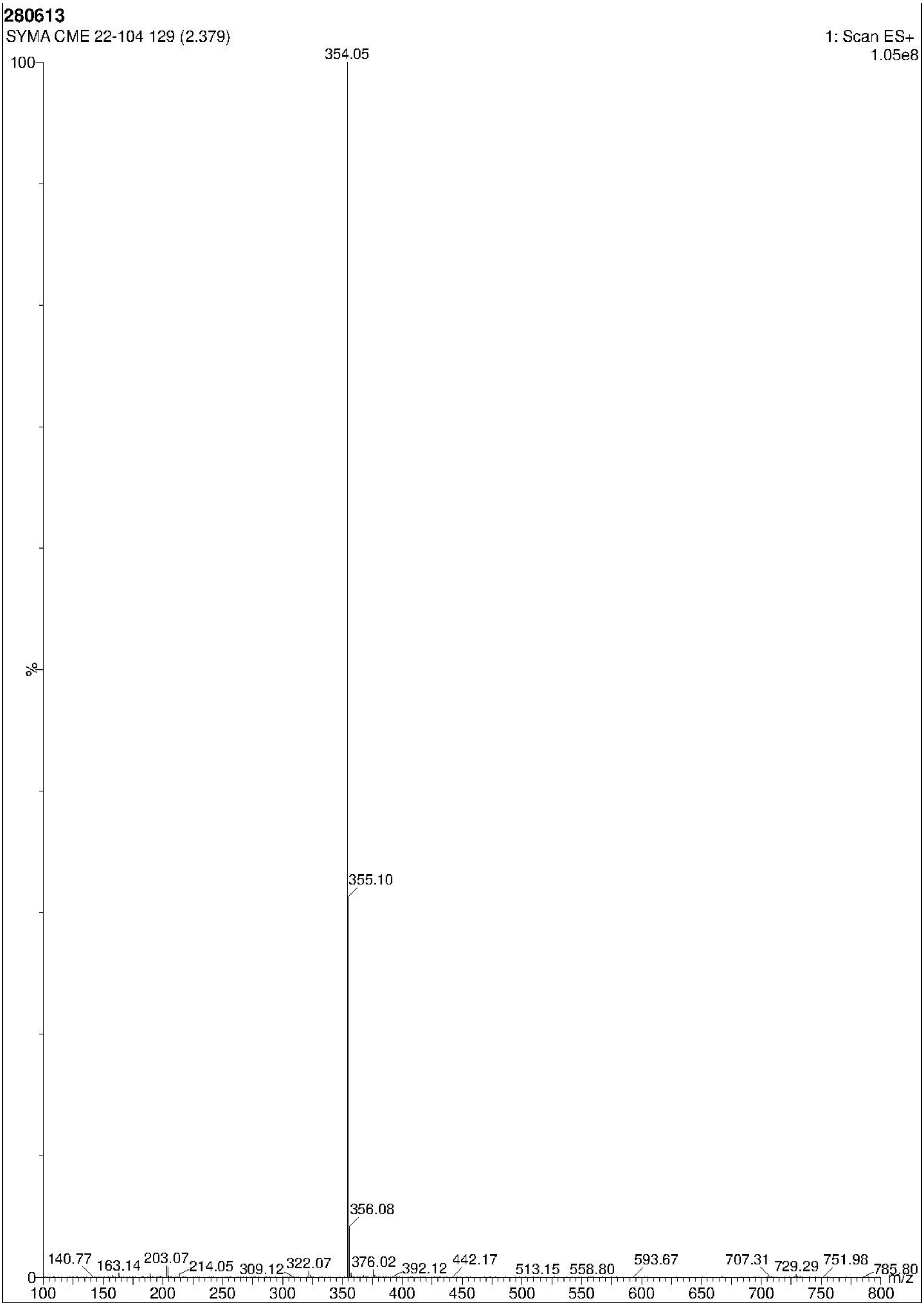
280613
SYMA CME 22-104 129 (2.379)
1: Scan ES+
1.05e8
100
%
0
354.05
355.10
356.08
140.77
163.14
203.07
214.05
309.12
322.07
376.02
392.12
442.17
513.15
558.80
593.67
707.31
729.29
751.98
785.80
m/z
100
150
200
250
300
350
400
450
500
550
600
650
700
750
800

methyl 3-[2-[1-[4-[3-(dimethylamino)propyl]phenyl]ethylidene] hydrazino]benzoate hydrochloride (**7**)

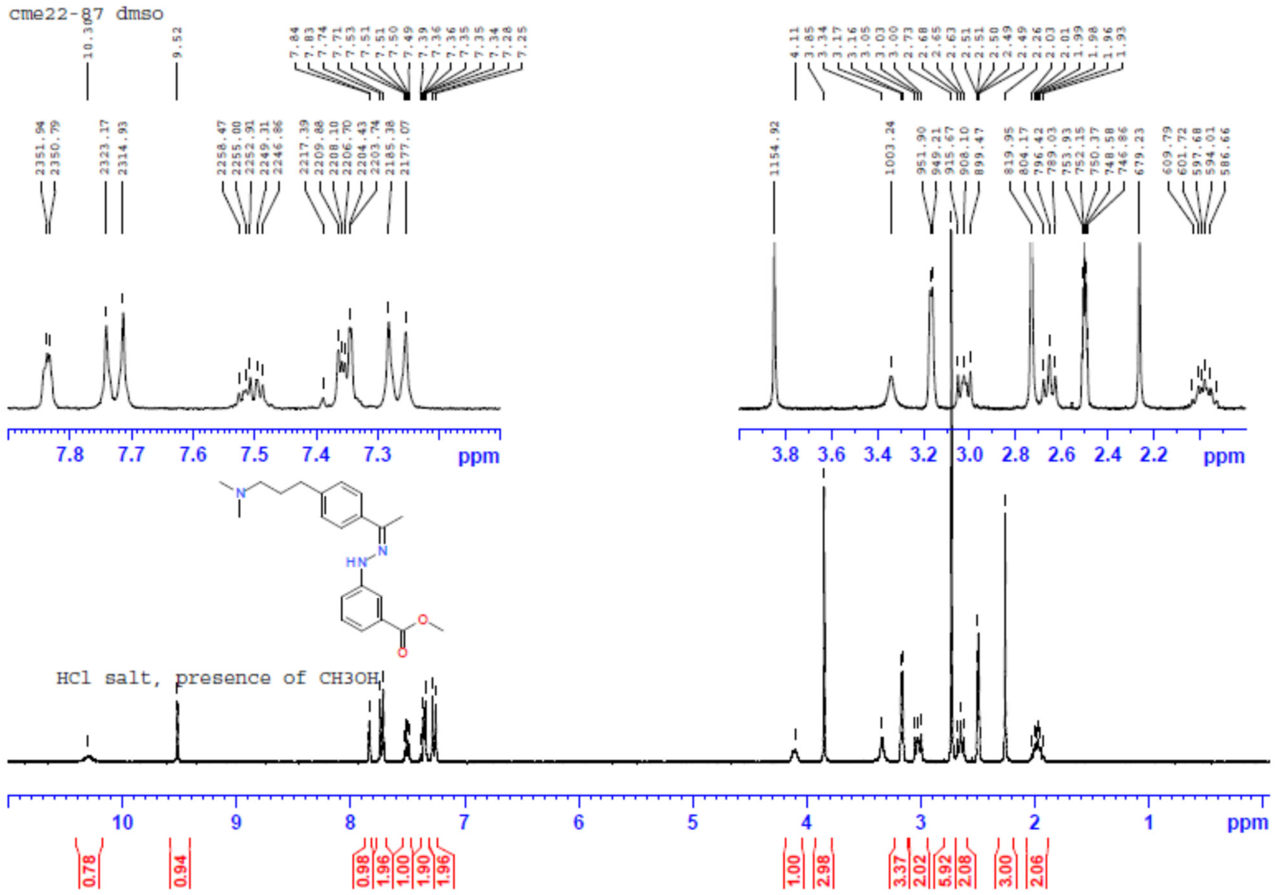

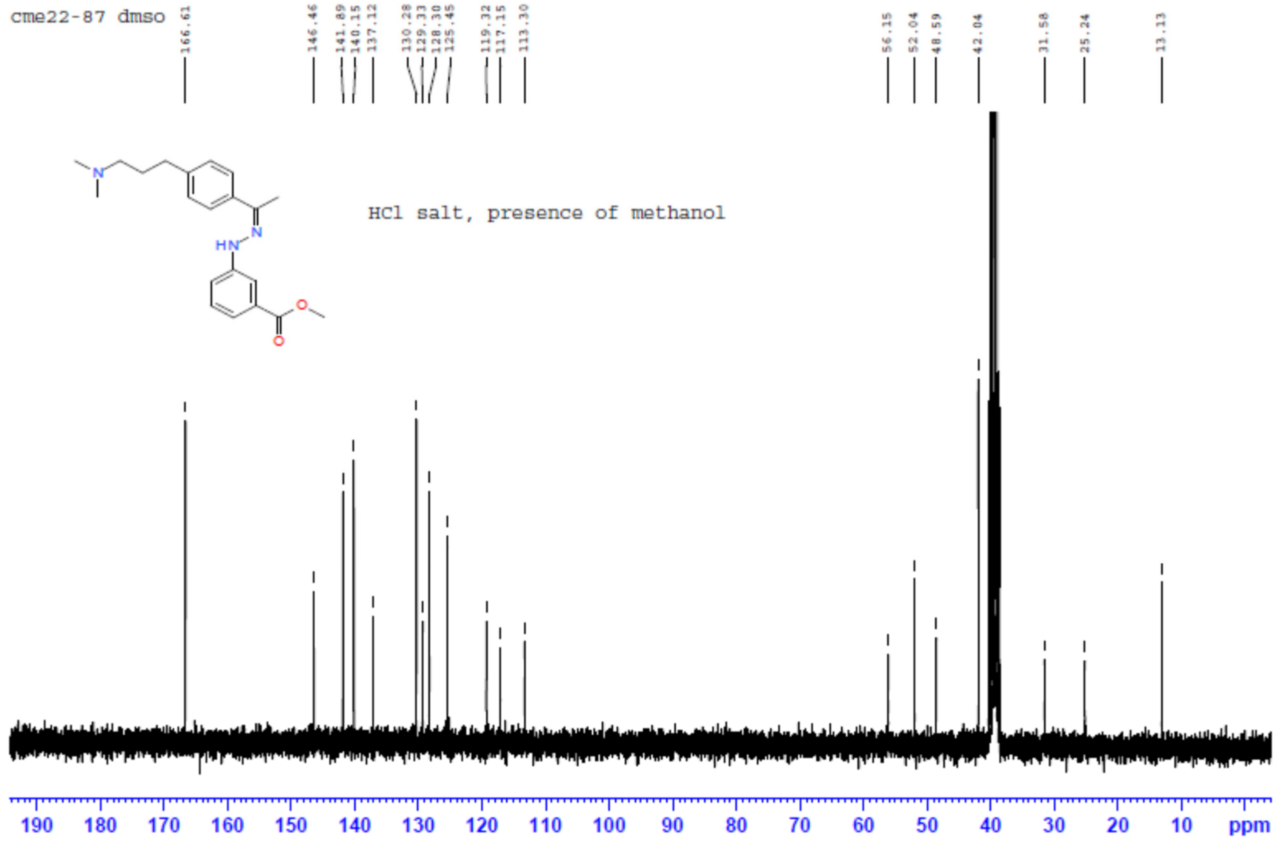

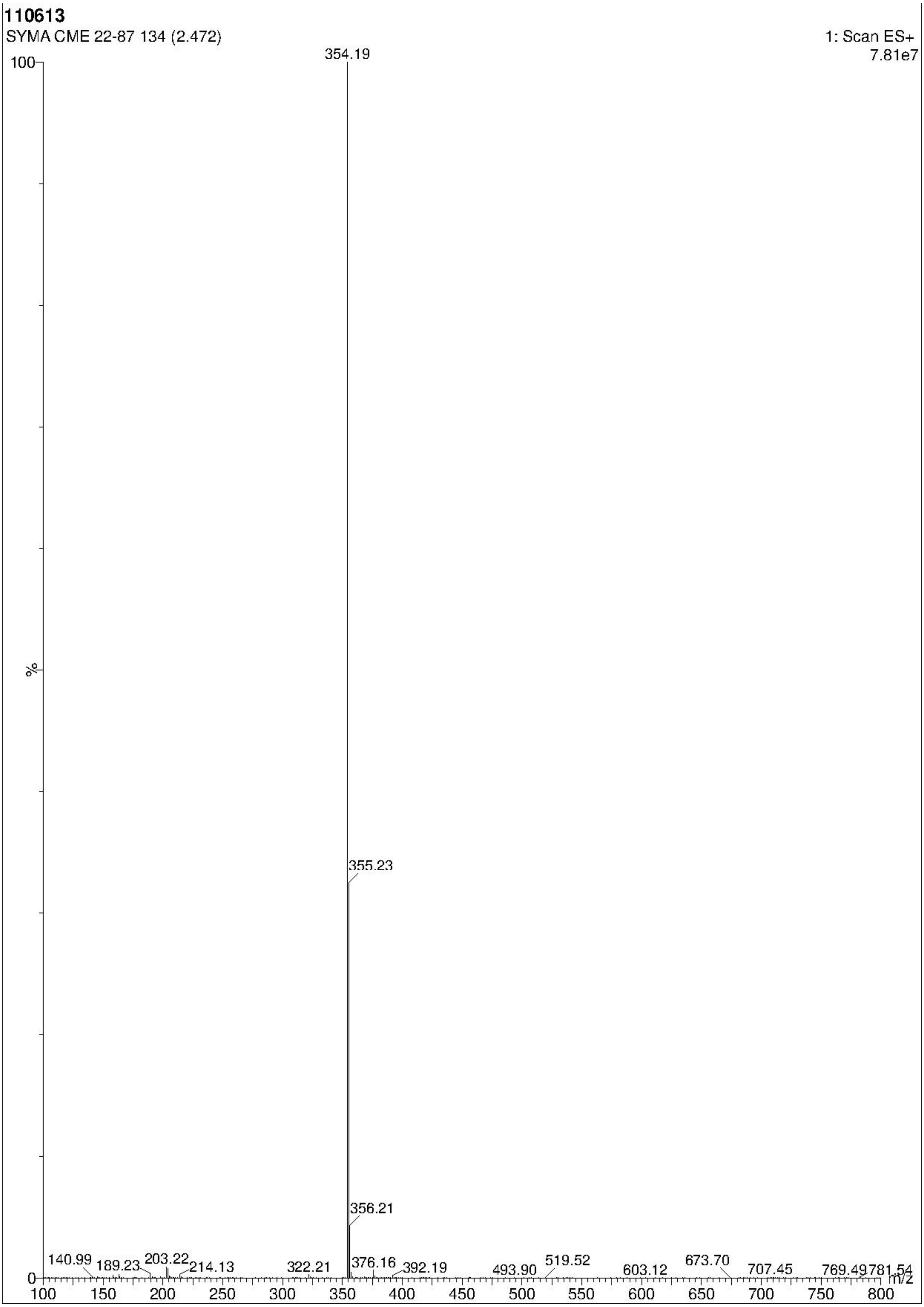

110613
SYMA CME 22-87 134 (2.472)
1: Scan ES+
7.81e7
354.19
355.23
356.21
140.99
189.23
203.22
214.13
322.21
376.16
392.19
493.90
519.52
603.12
673.70
707.45
769.49
781.54
100
%
0
100 150 200 250 300 350 400 450 500 550 600 650 700 750 800
m/z

methyl 4-[3-[4-[3-(dimethylamino)propyl]phenyl]-4-formyl-pyrazol-1-yl]benzoate (**8**)

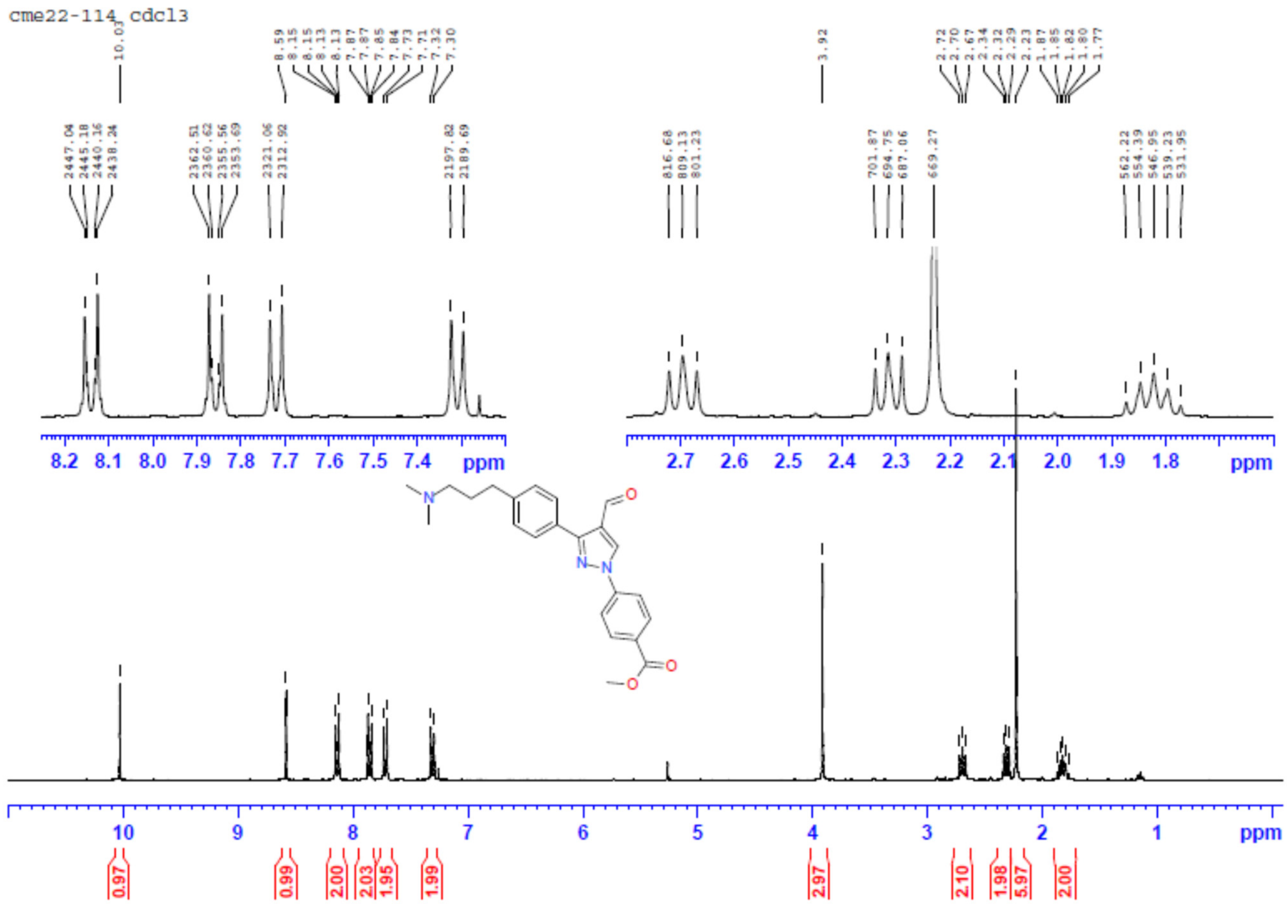

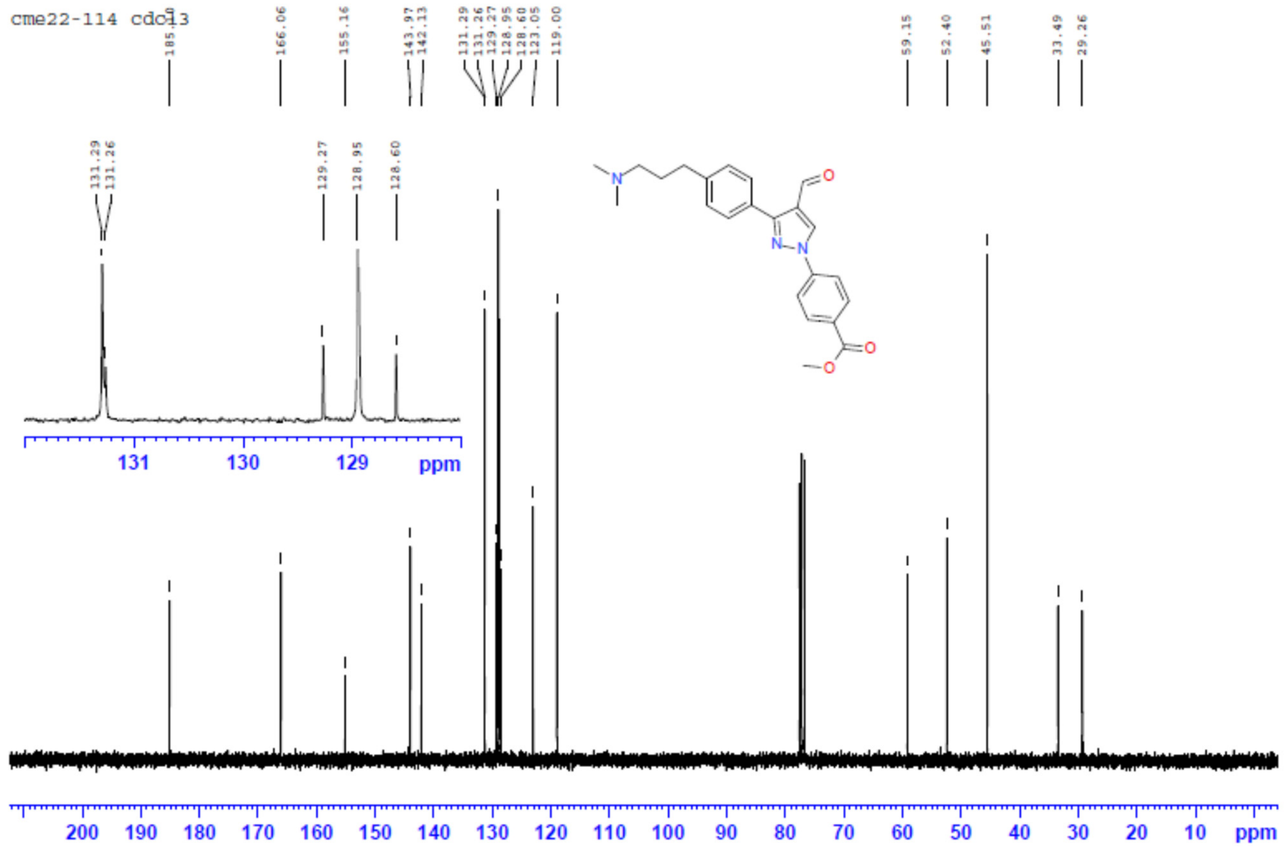

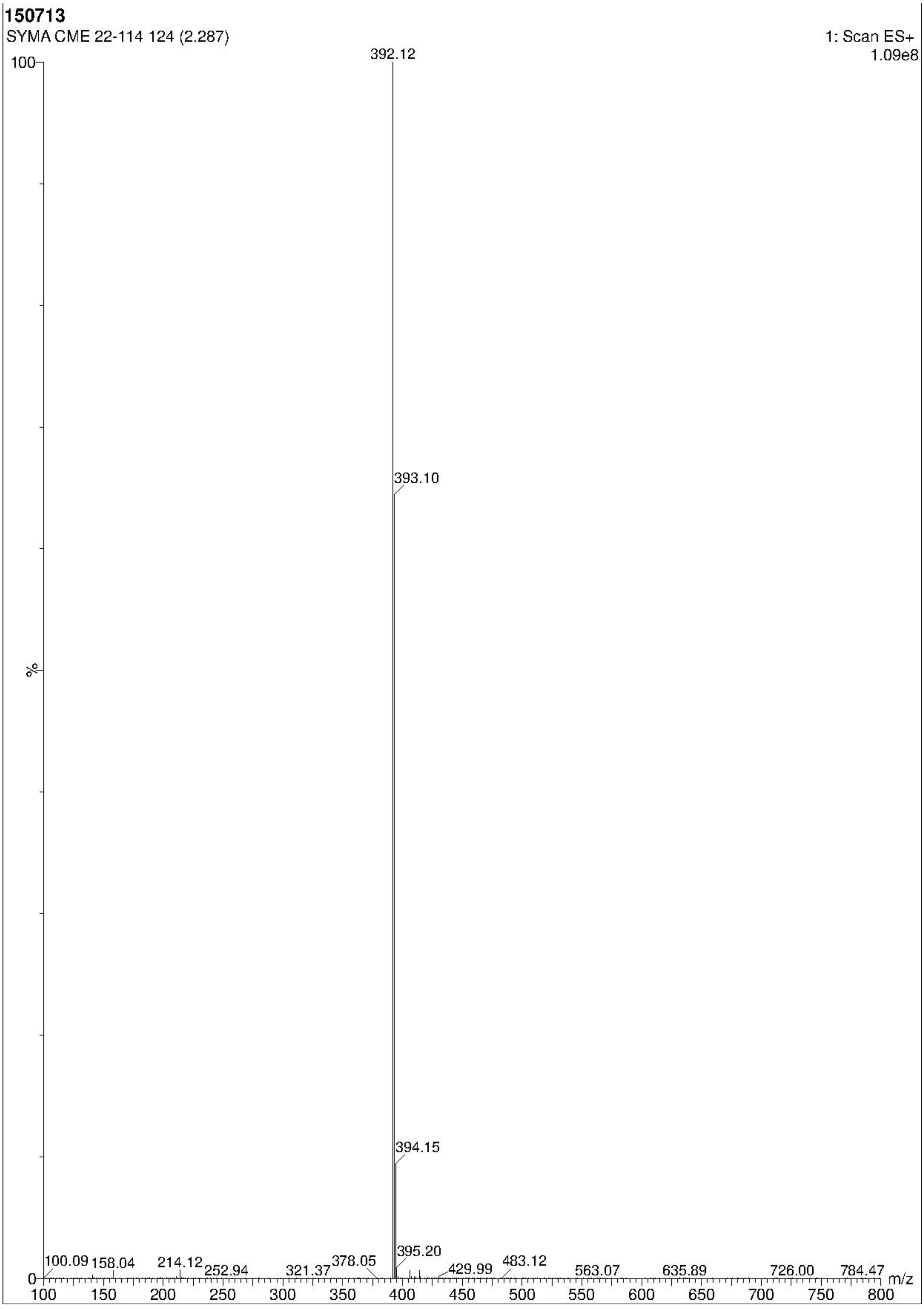
150713
SYMA CME 22-114 124 (2.287)
1: Scan ES+
1.09e8
392.12
393.10
394.15
395.20
100.09
158.04
214.12
252.94
321.37
378.05
429.99
483.12
563.07
635.89
726.00
784.47
%
m/z
100
0
100
150
200
250
300
350
400
450
500
550
600
650
700
750
800

methyl 3-[3-[4-[3-(dimethylamino)propyl]phenyl]-4-formyl-pyrazol-1-yl]benzoate (**9**)

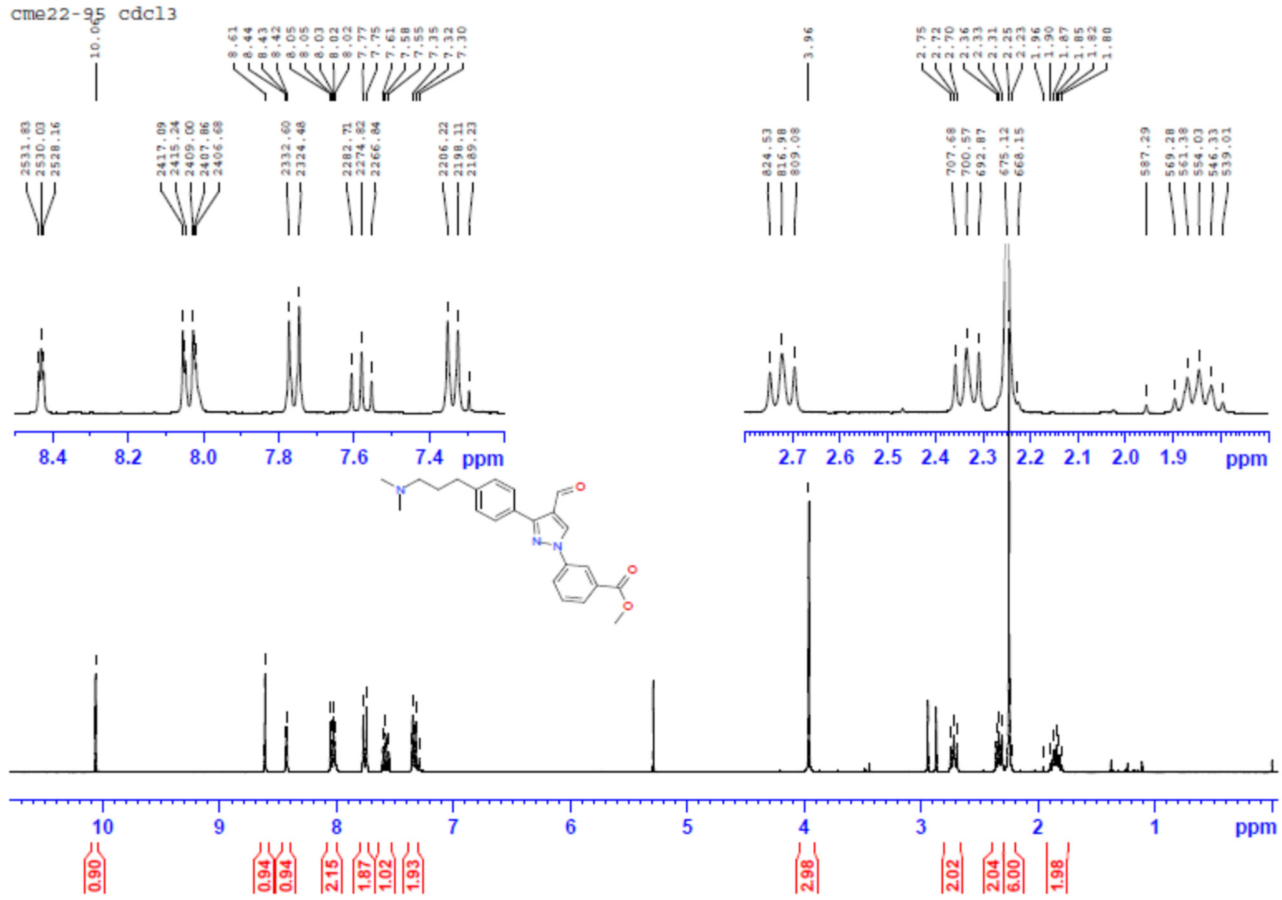

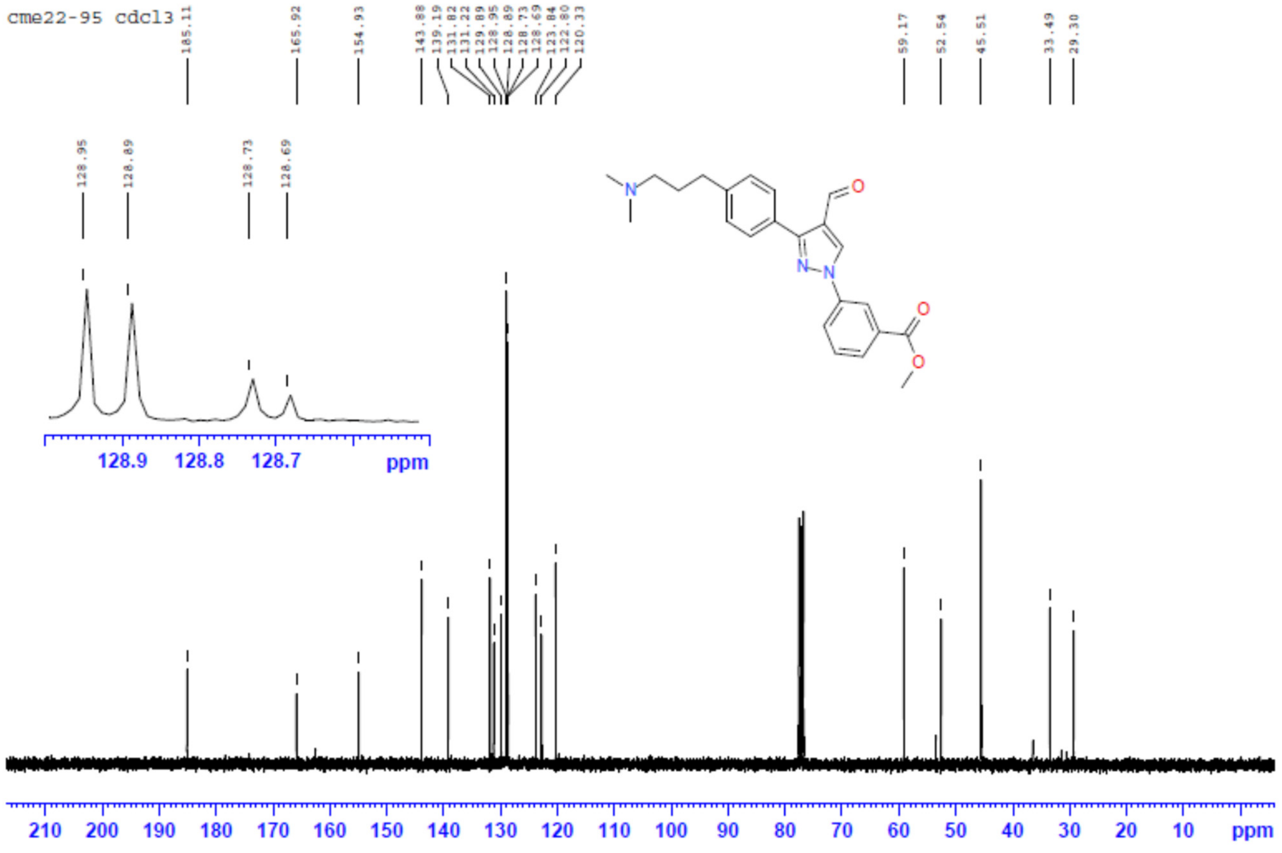

100613

SYMA CME 22-95 127 (2.342)

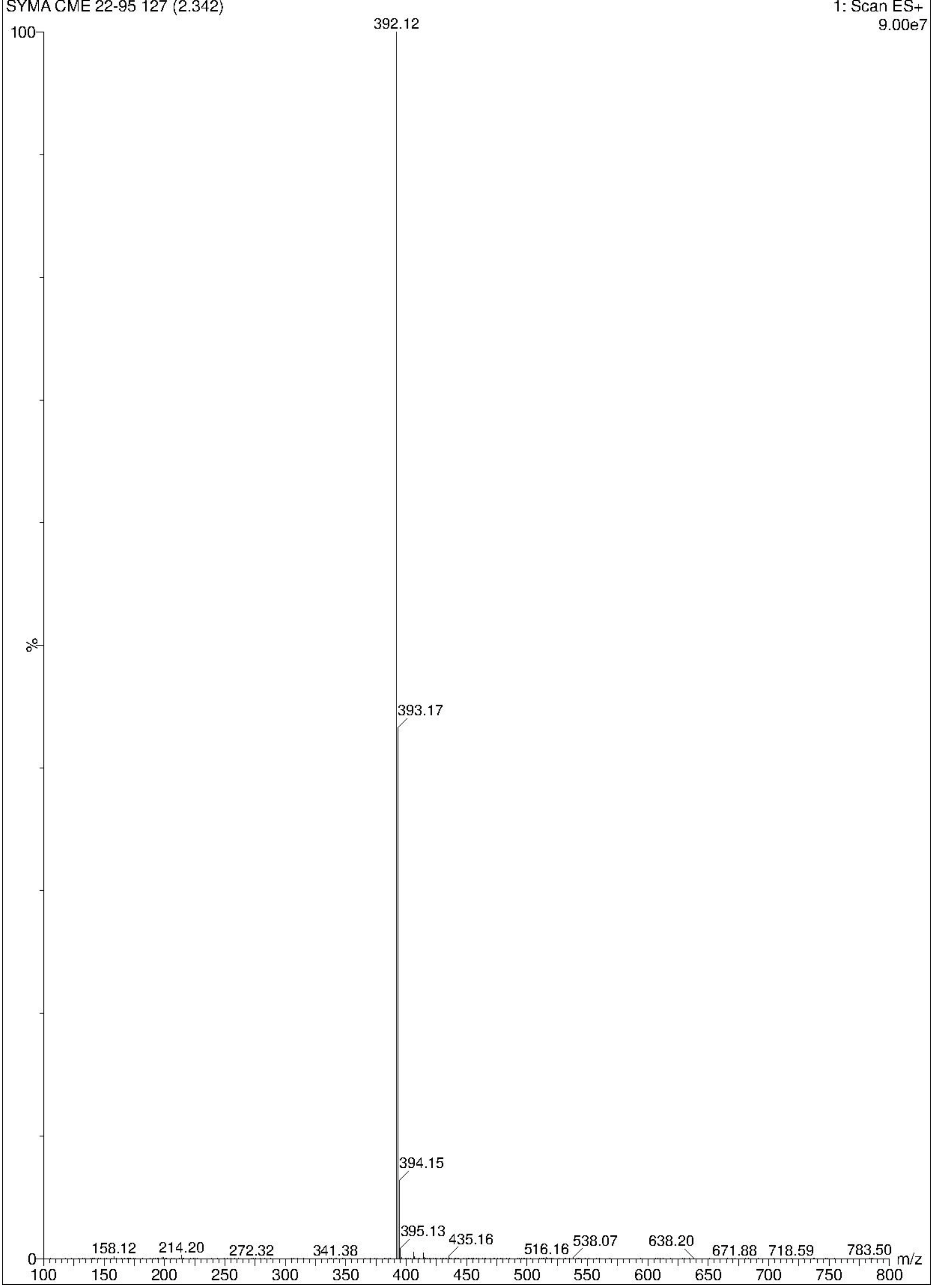

methyl 4-[4-(dimethylaminomethyl)-3-[4-[3-(dimethylamino)propyl]phenyl]pyrazol-1-yl]benzoate (**10**)

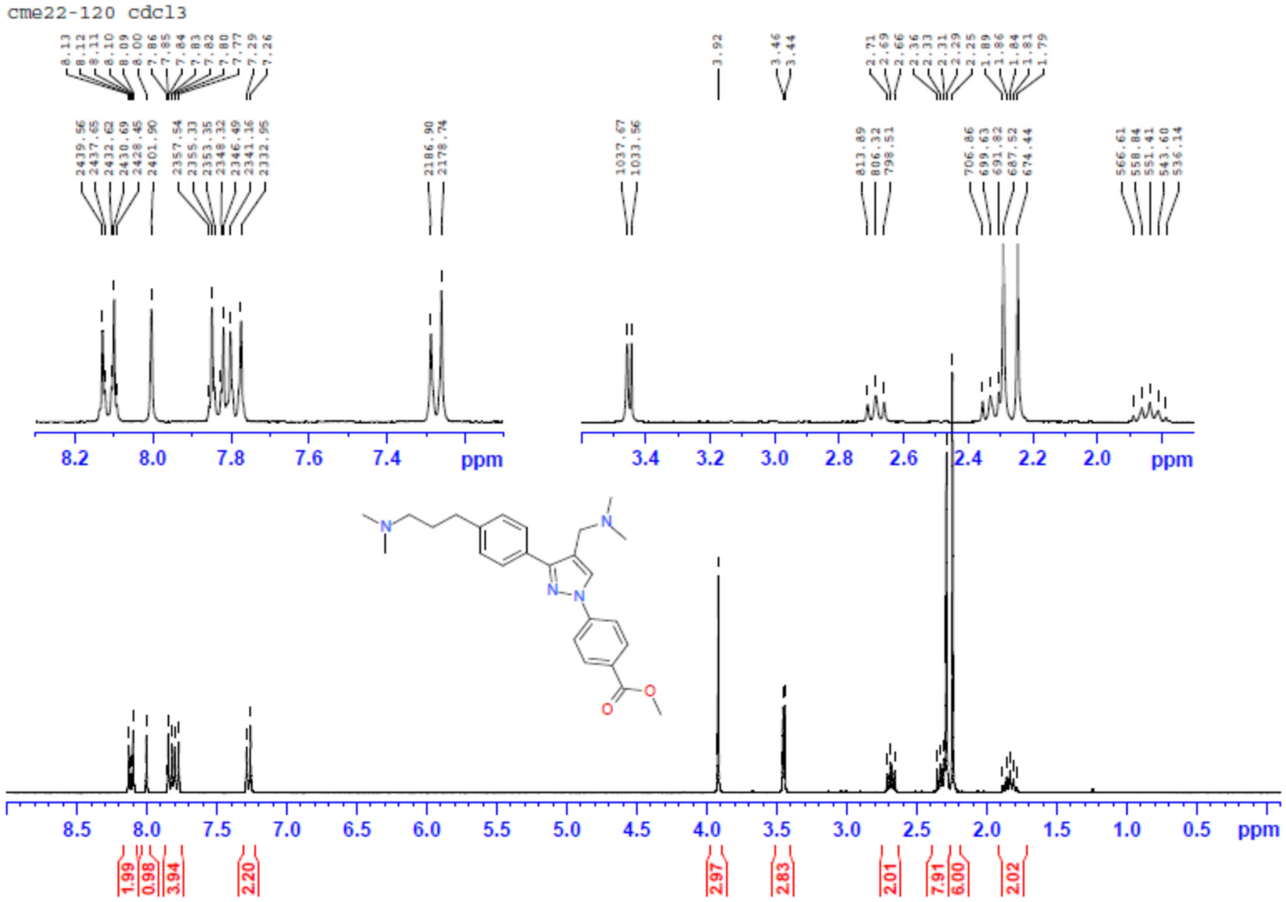

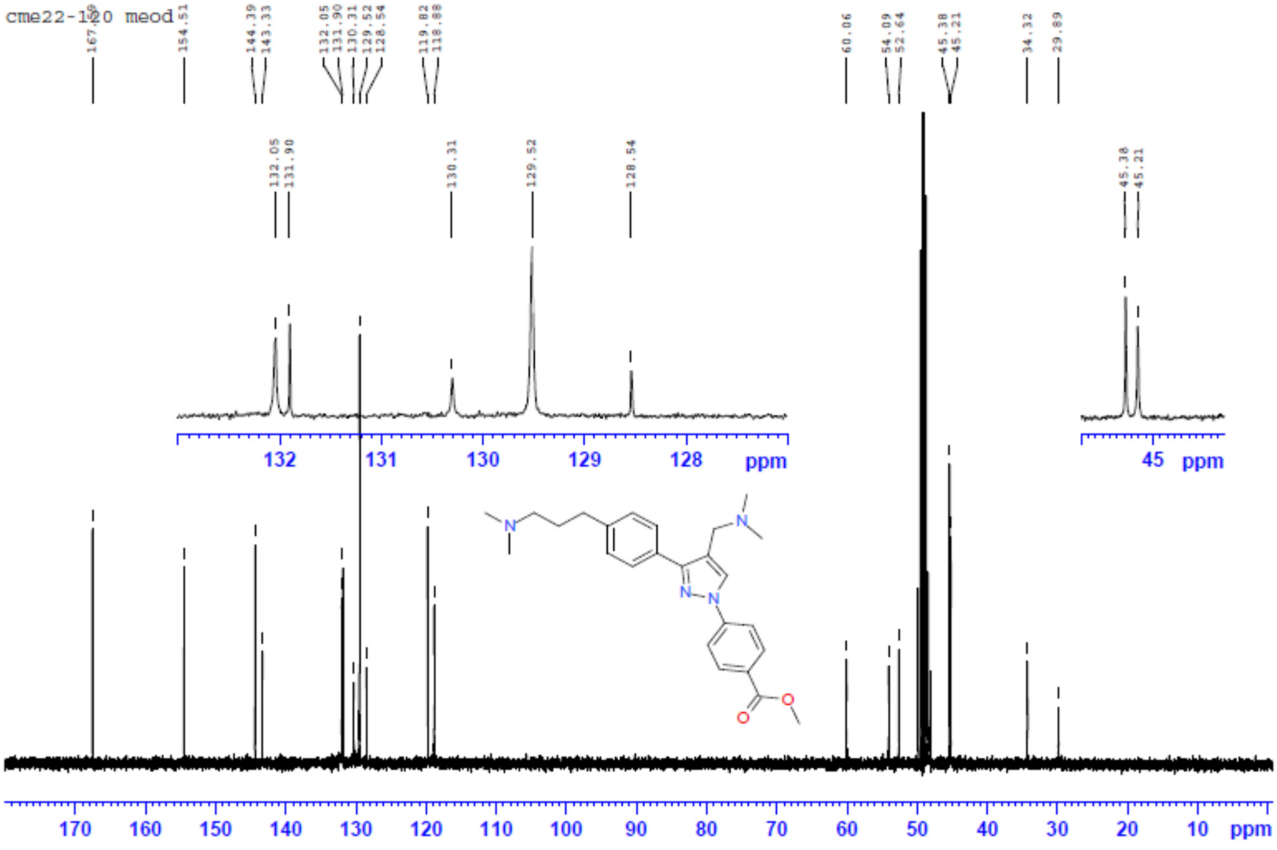

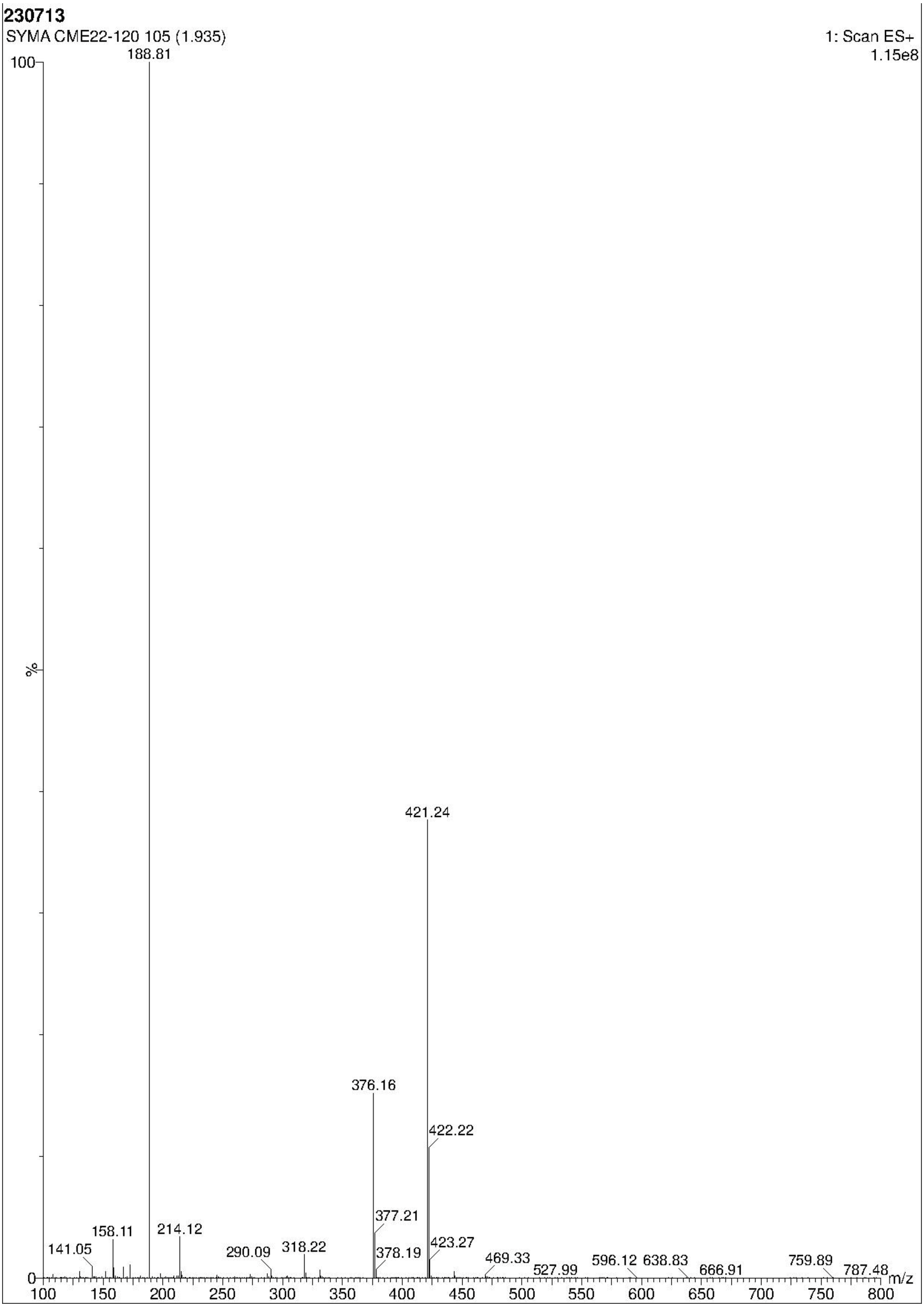
230713
SYMA CME22-120 105 (1.935)
1: Scan ES+
1.15e8
100
%
0
188.81
421.24
376.16
422.22
377.21
423.27
378.19
158.11
214.12
141.05
290.09
318.22
469.33
527.99
596.12
638.83
666.91
759.89
787.48
m/z
100
150
200
250
300
350
400
450
500
550
600
650
700
750
800

methyl 3-[4-(dimethylaminomethyl)-3-[4-[3-(dimethylamino)propyl]phenyl]pyrazol-1-yl]benzoate (**11**)

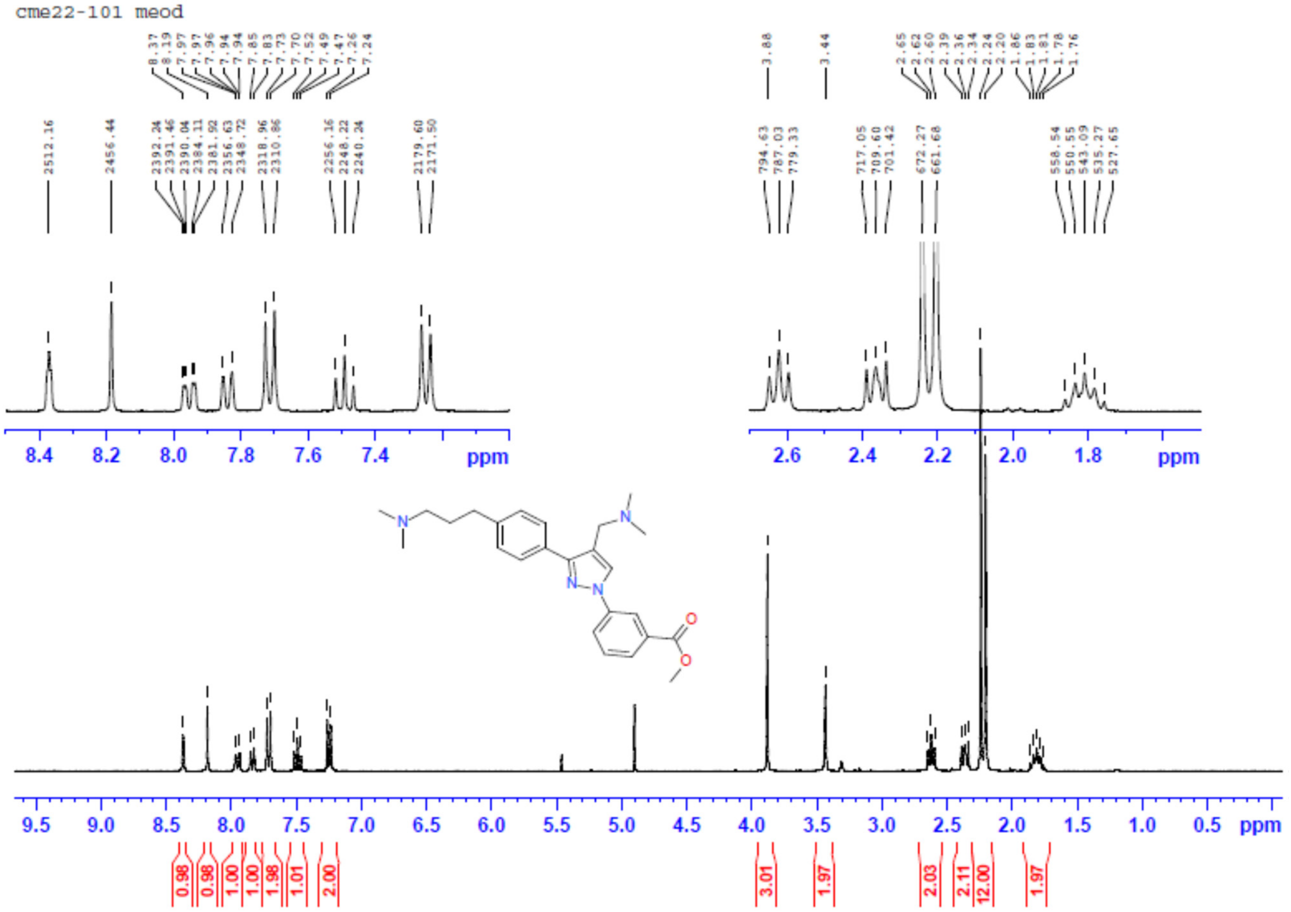

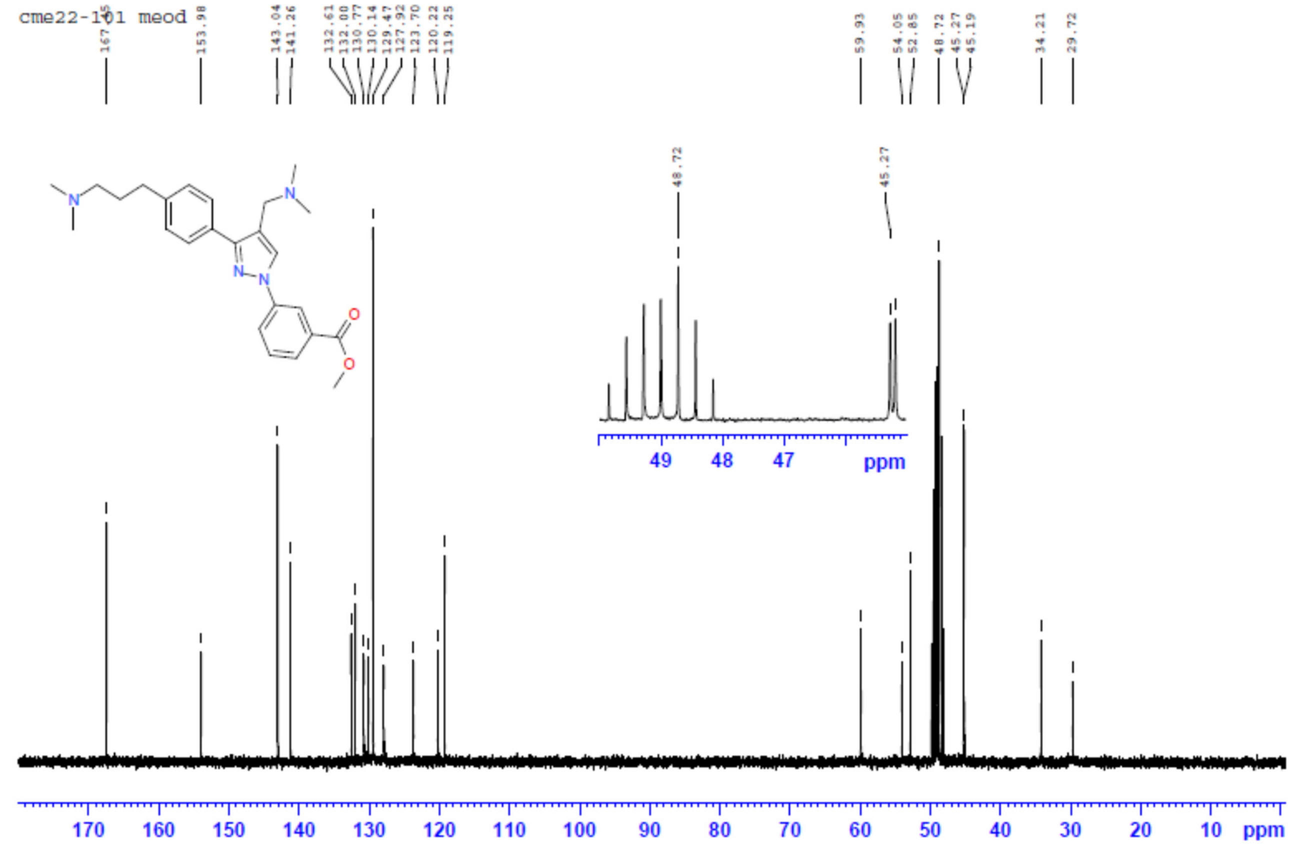

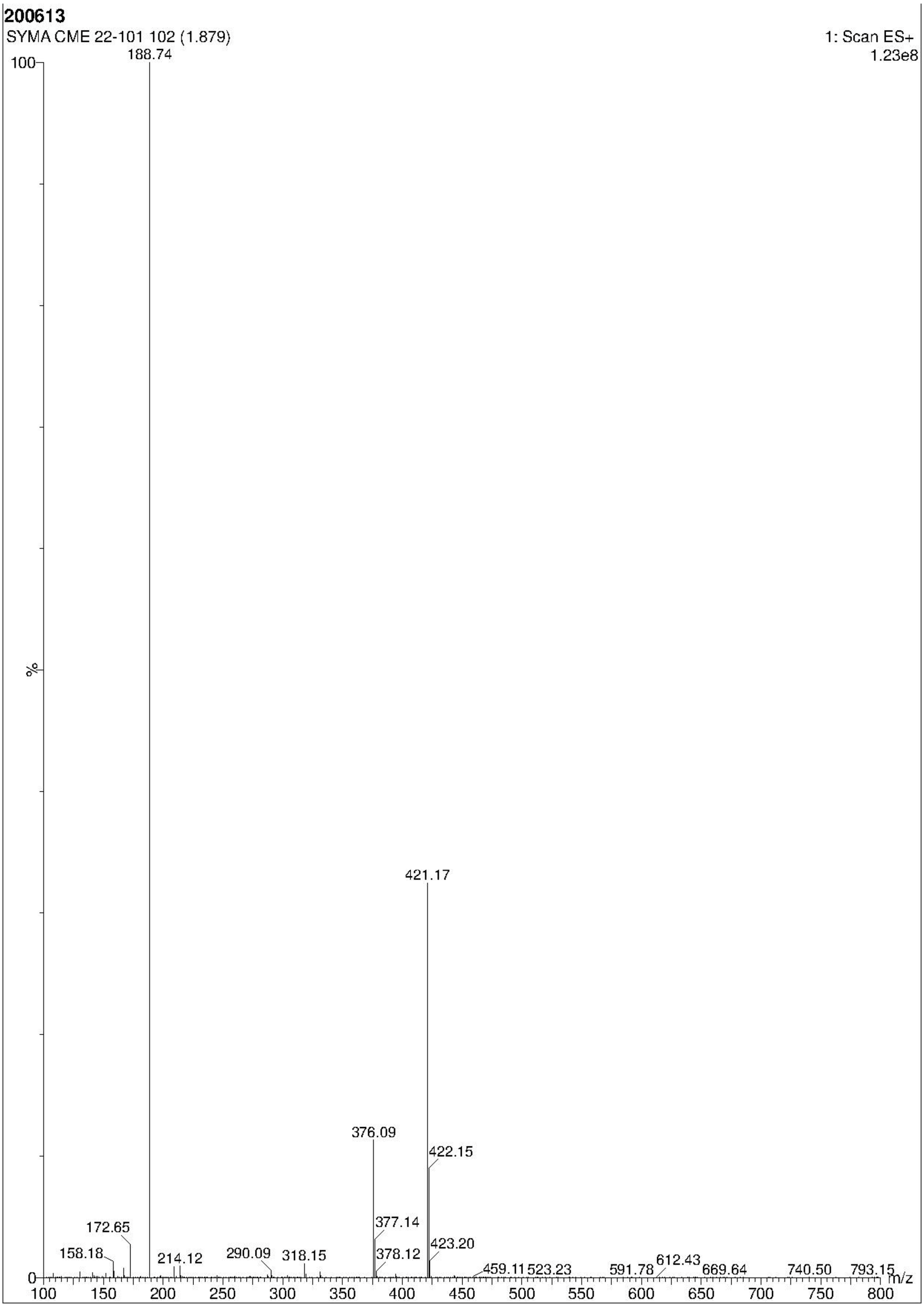
200613
SYMA CME 22-101 102 (1.879)
1: Scan ES+
1.23e8
188.74
421.17
376.09
422.15
377.14
172.65
423.20
158.18
214.12
290.09
318.15
378.12
459.11
523.23
591.78
612.43
669.64
740.50
793.15
100
%
0
m/z
100
150
200
250
300
350
400
450
500
550
600
650
700
750
800

methyl 4-[4-(3-aminopropyl)-3-[4-[3-(dimethylamino)propyl]phenyl]pyrazol-1-yl]benzoate (**12**)

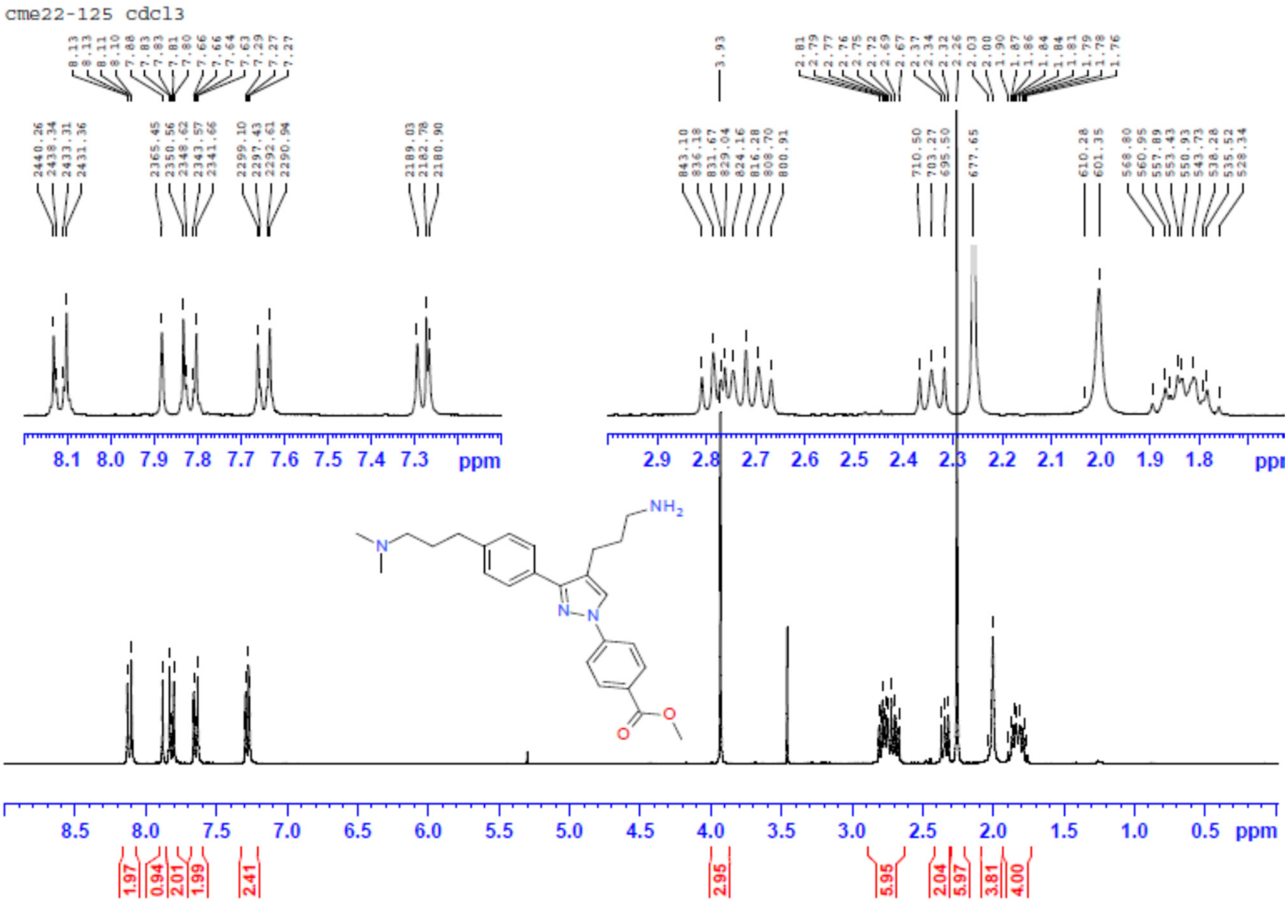

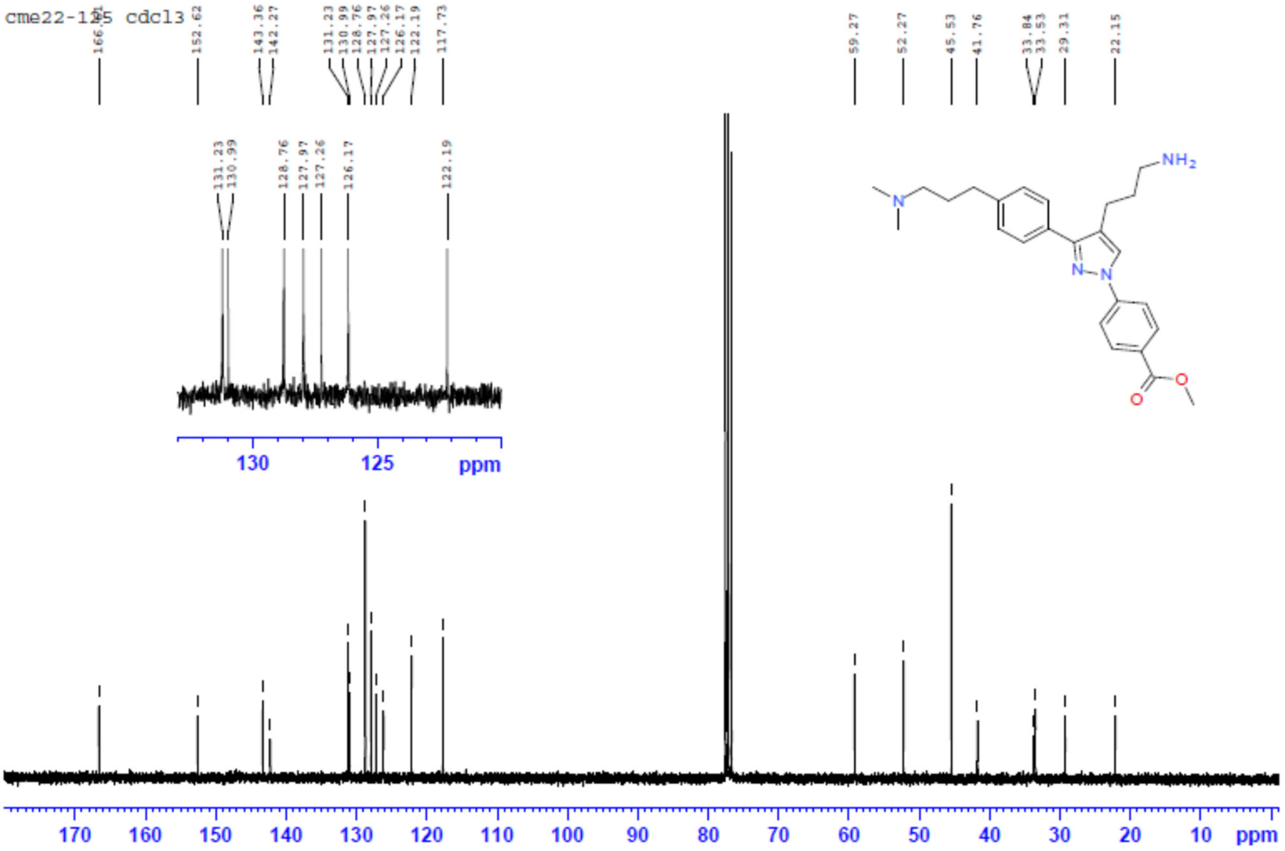

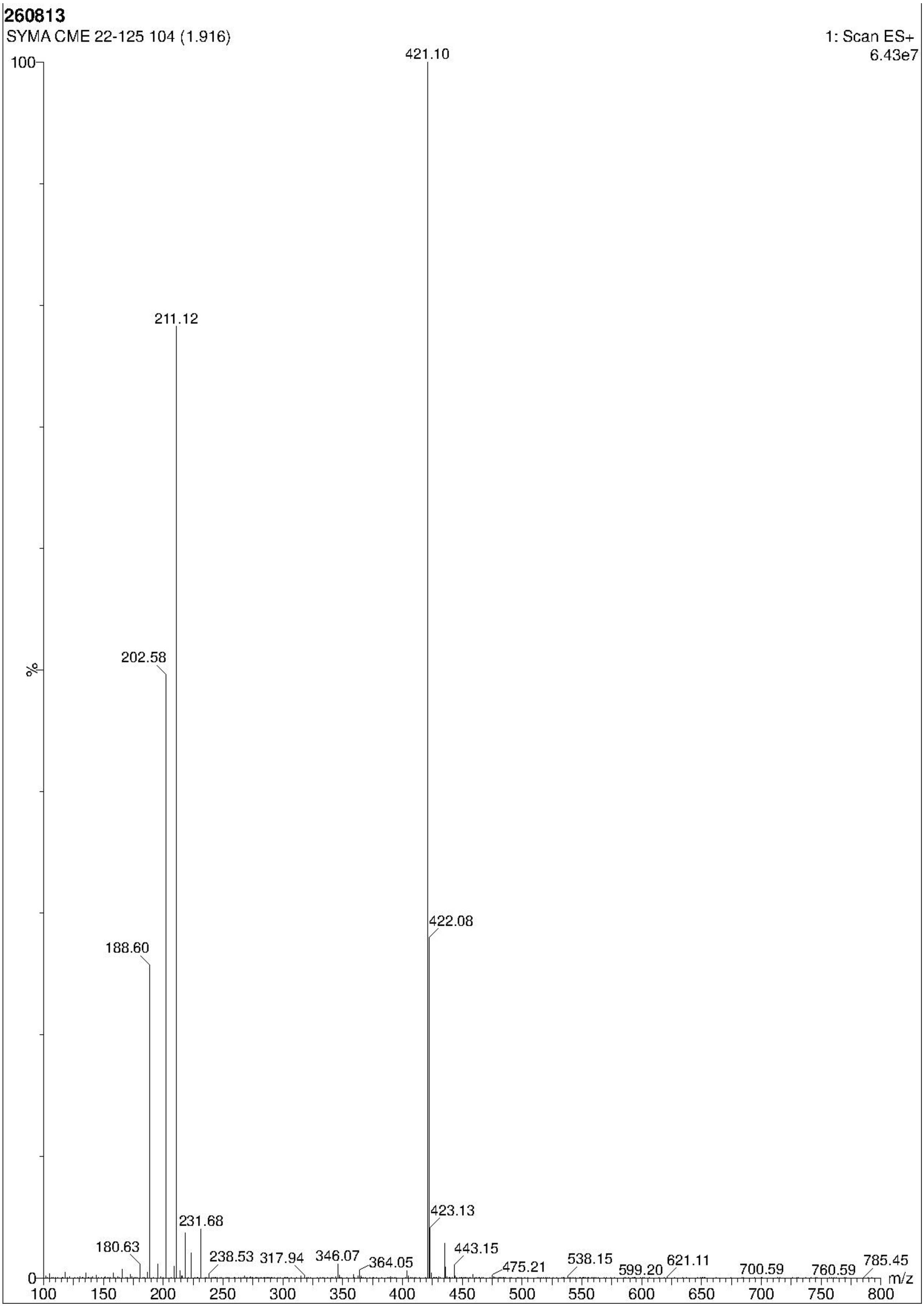

260813
SYMA CME 22-125 104 (1.916)
1: Scan ES+
6.43e7
100
%
0
421.10
211.12
202.58
188.60
422.08
423.13
231.68
180.63
238.53
317.94
346.07
364.05
443.15
475.21
538.15
599.20
621.11
700.59
760.59
785.45
m/z
100
150
200
250
300
350
400
450
500
550
600
650
700
750
800

methyl 3-[4-(3-aminopropyl)-3-[4-[3-(dimethylamino)propyl]phenyl]pyrazol-1-yl]benzoate (**13**)

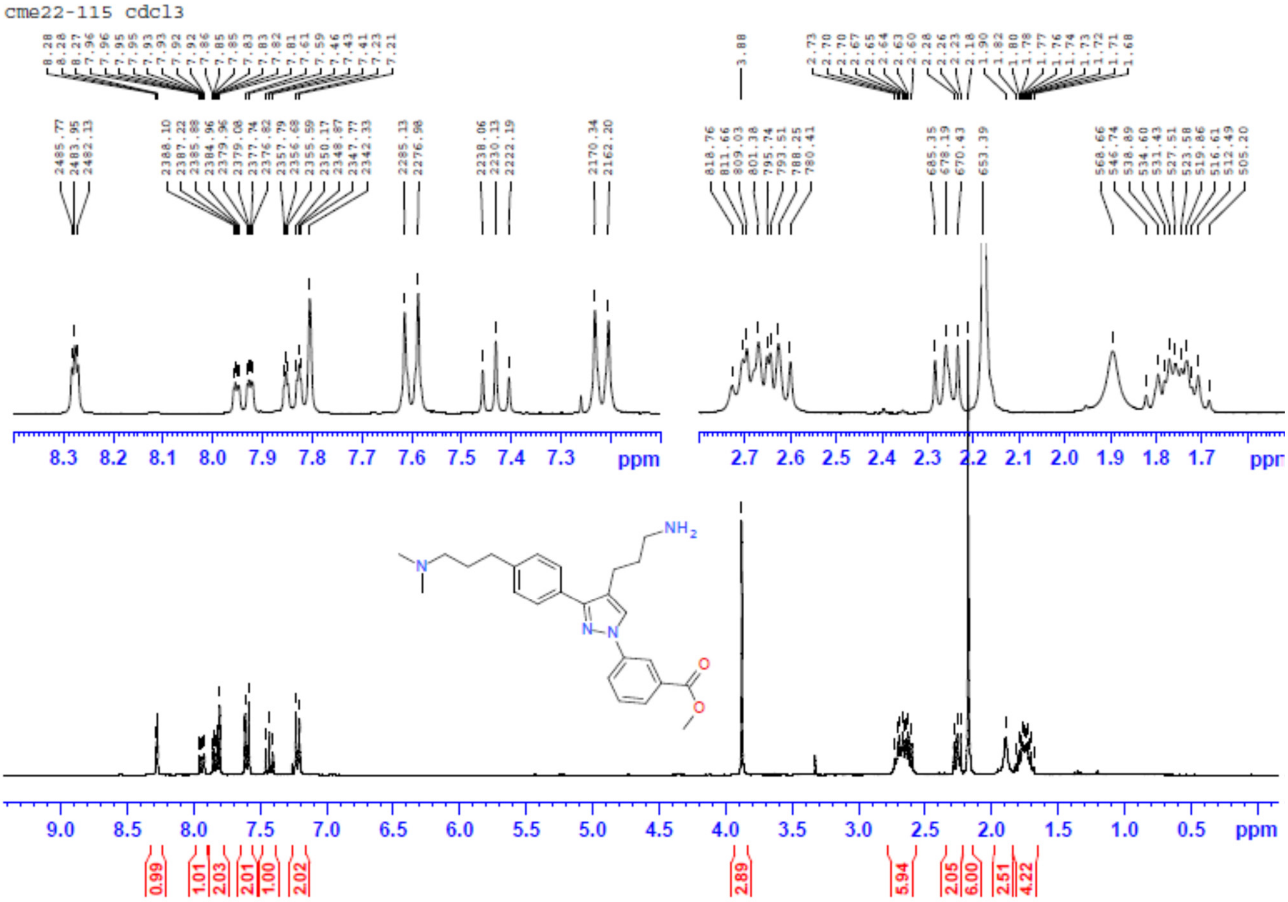

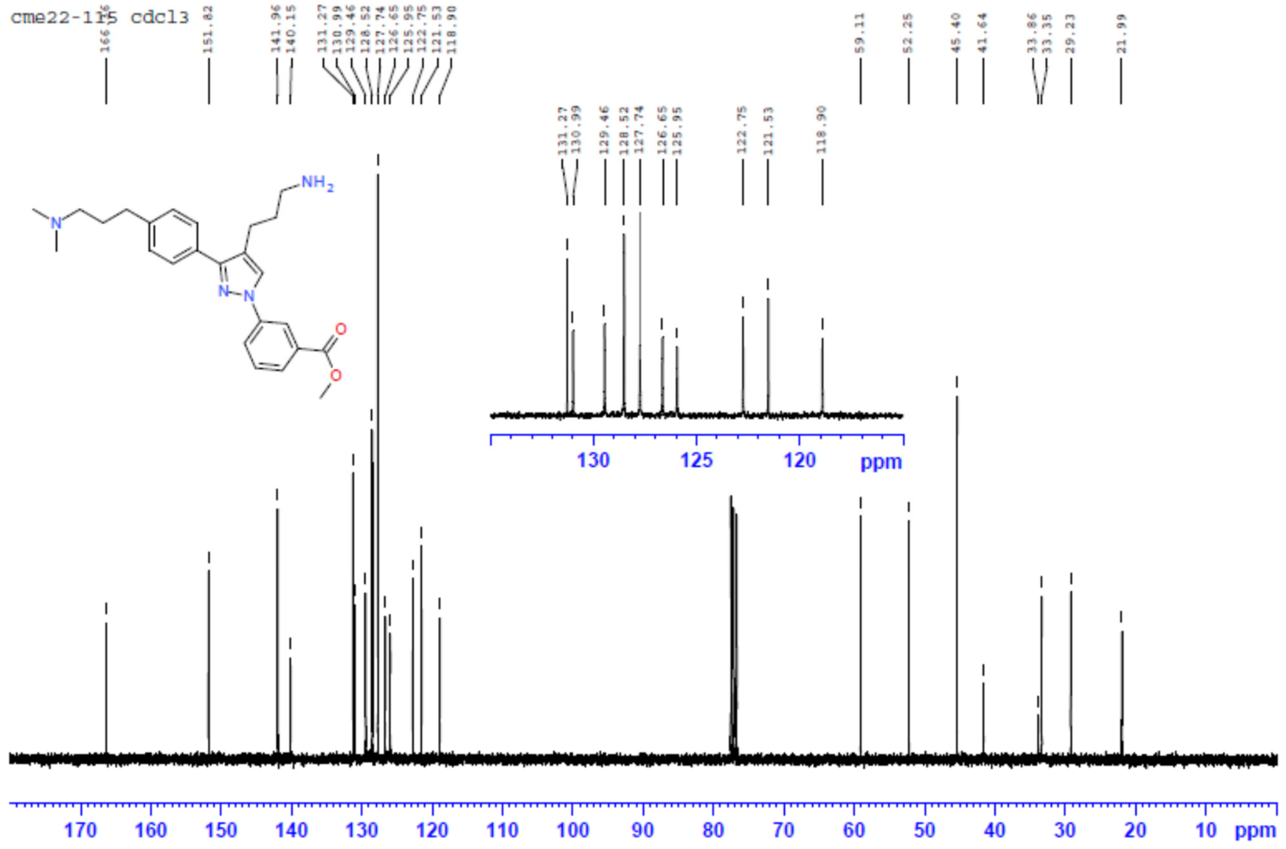

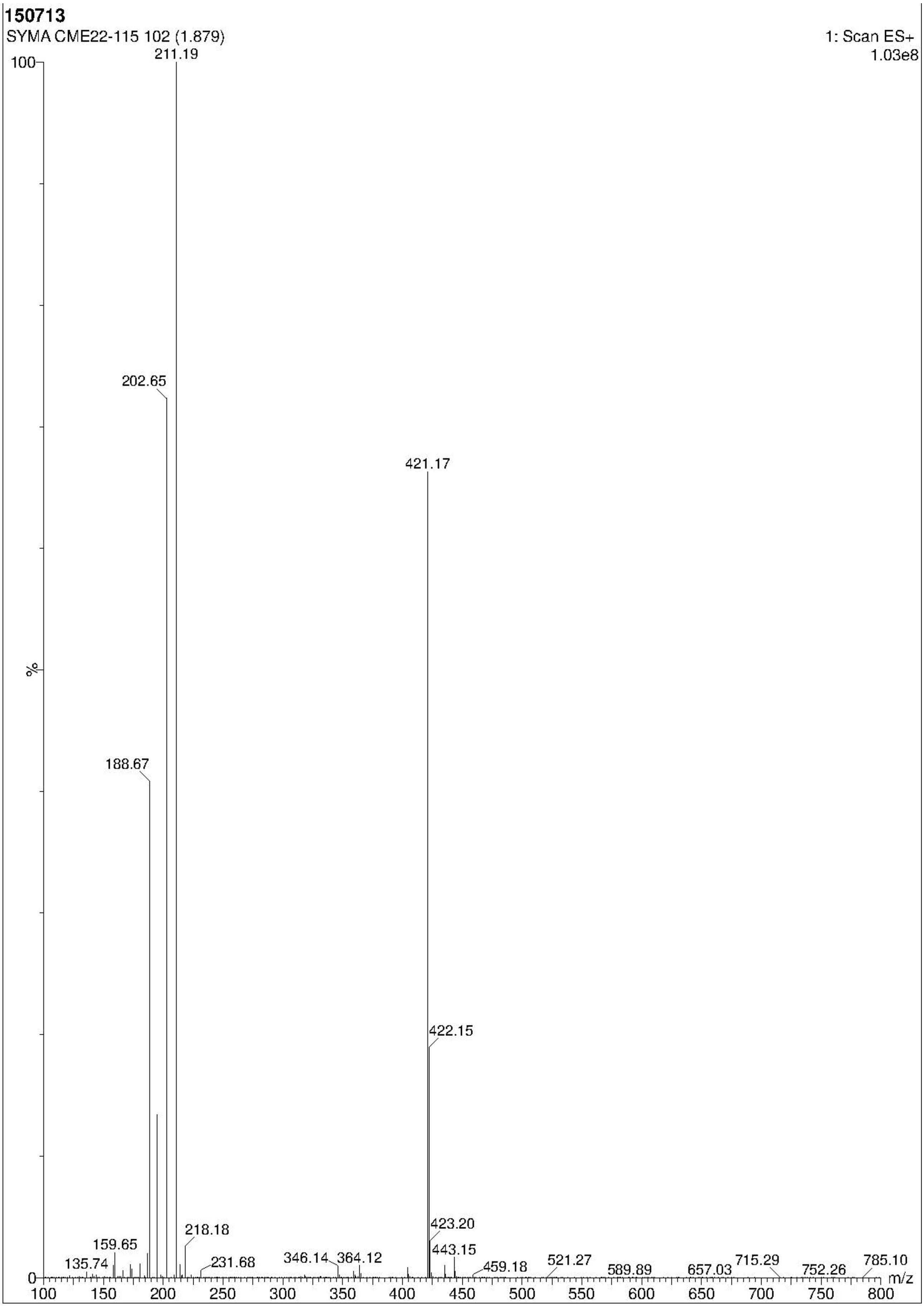
150713
SYMA CME22-115 102 (1.879)
1: Scan ES+
1.03e8
100
%
0
211.19
202.65
421.17
188.67
422.15
423.20
218.18
159.65
443.15
135.74
231.68
346.14
364.12
459.18
521.27
589.89
657.03
715.29
752.26
785.10
m/z
100
150
200
250
300
350
400
450
500
550
600
650
700
750
800

methyl 4-[4-[3-(dimethylamino)propyl]-3-[4-[3-(dimethylamino)propyl]phenyl]pyrazol-1-yl] benzoate (**14**)

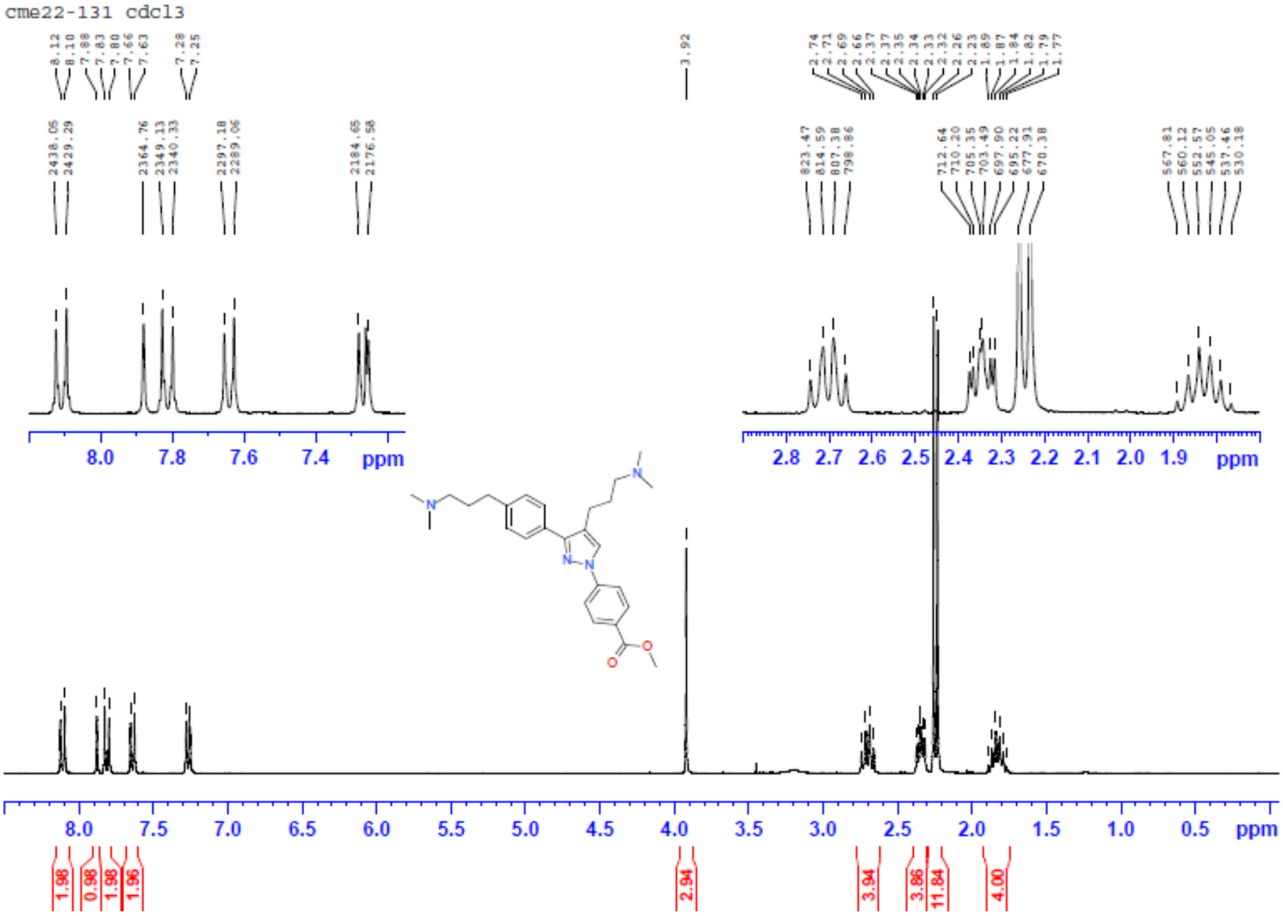

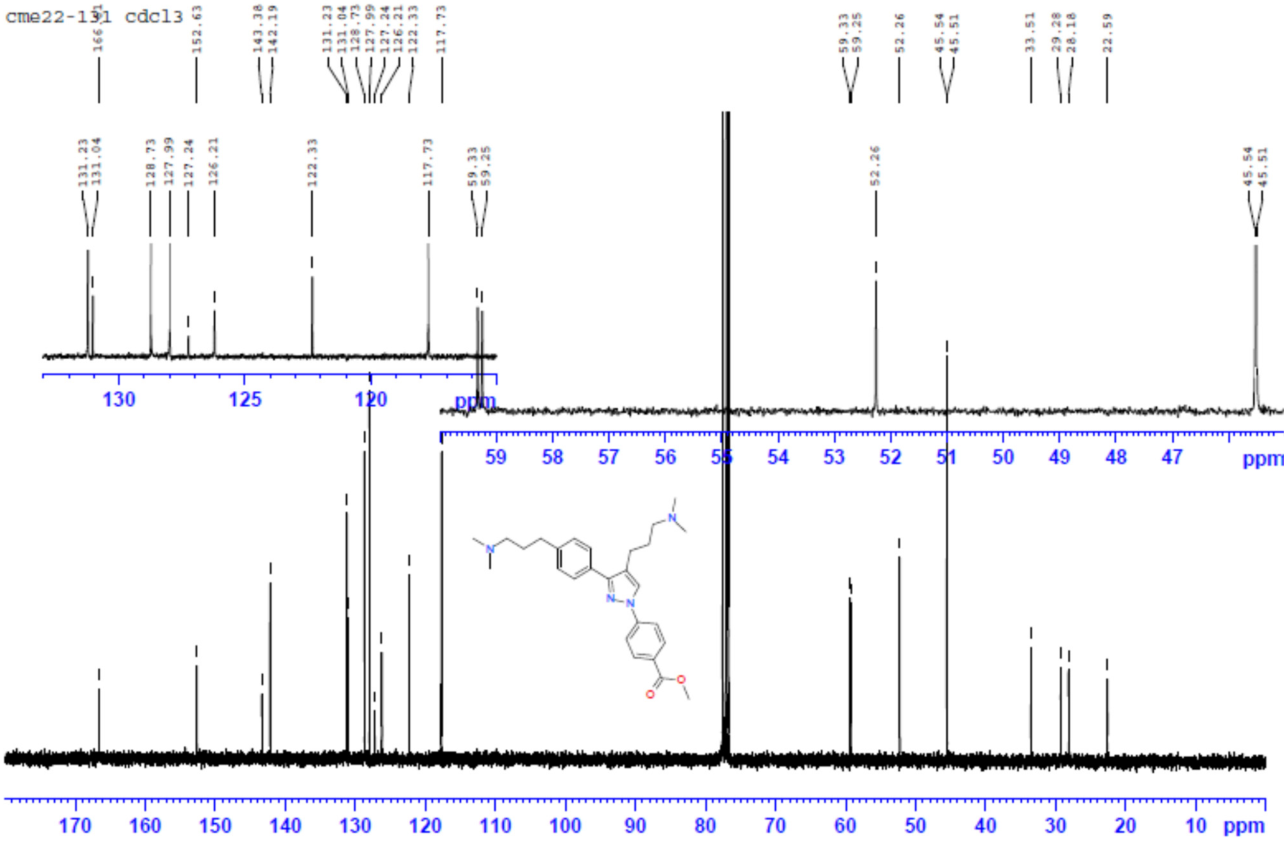

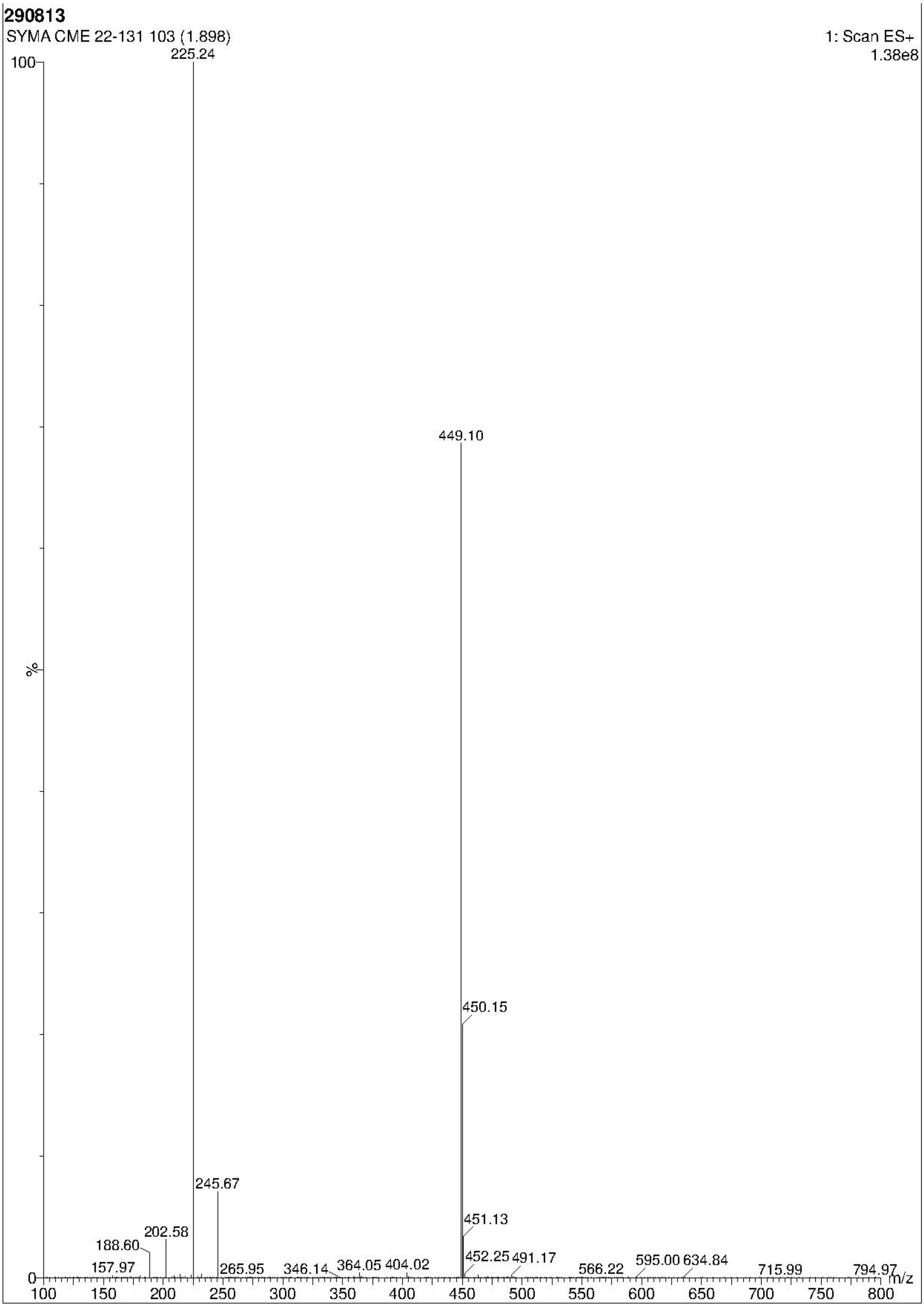
290813
SYMA CME 22-131 103 (1.898)
1: Scan ES+
1.38e8
225.24
449.10
450.15
245.67
451.13
202.58
188.60
157.97
265.95
346.14
364.05
404.02
452.25
491.17
566.22
595.00
634.84
715.99
794.97
m/z
%
100
0
100
150
200
250
300
350
400
450
500
550
600
650
700
750
800

methyl 3-[4-[3-(dimethylamino)propyl]-3-[4-[3-(dimethylamino)propyl]phenyl]pyrazol-1-yl] benzoate (**15**)

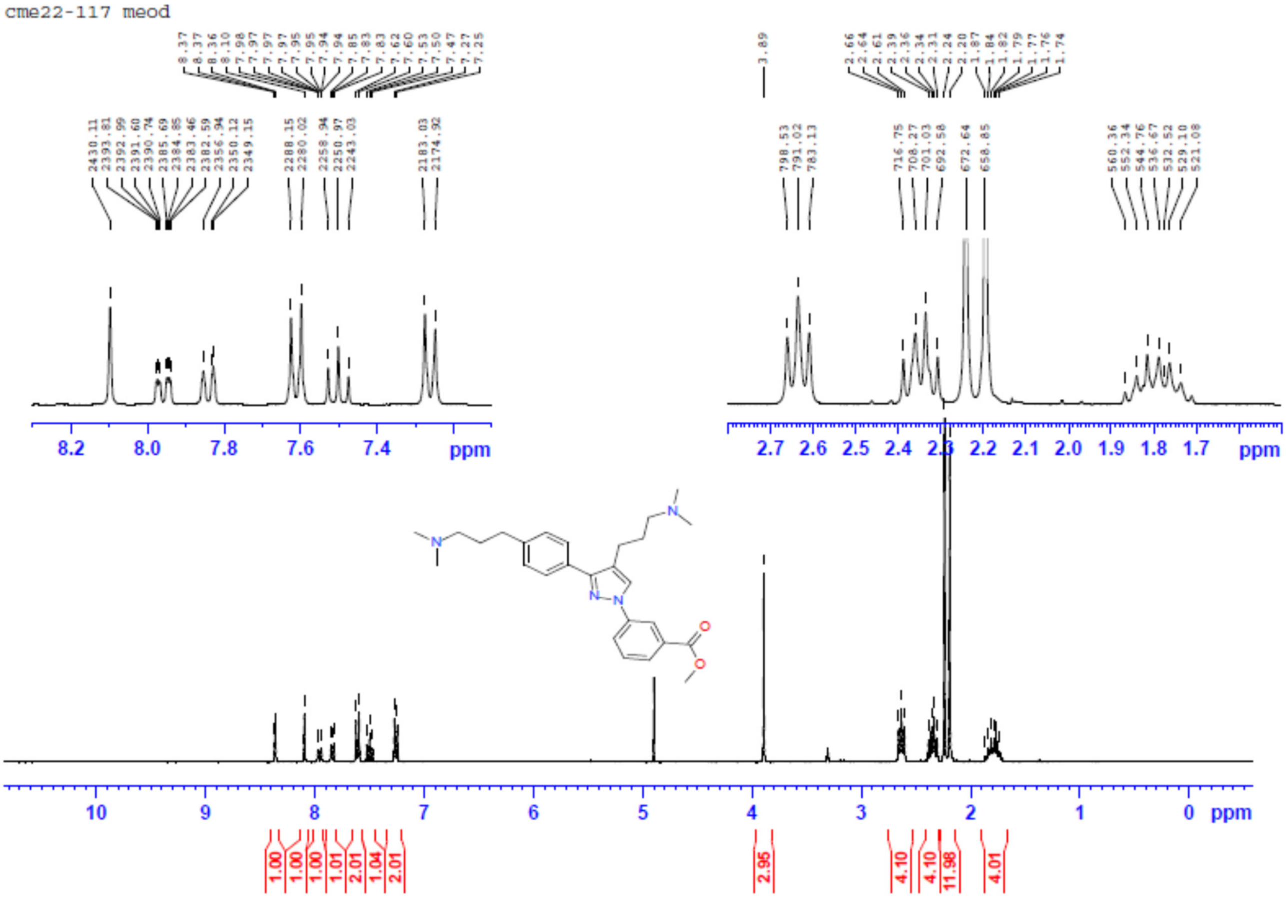

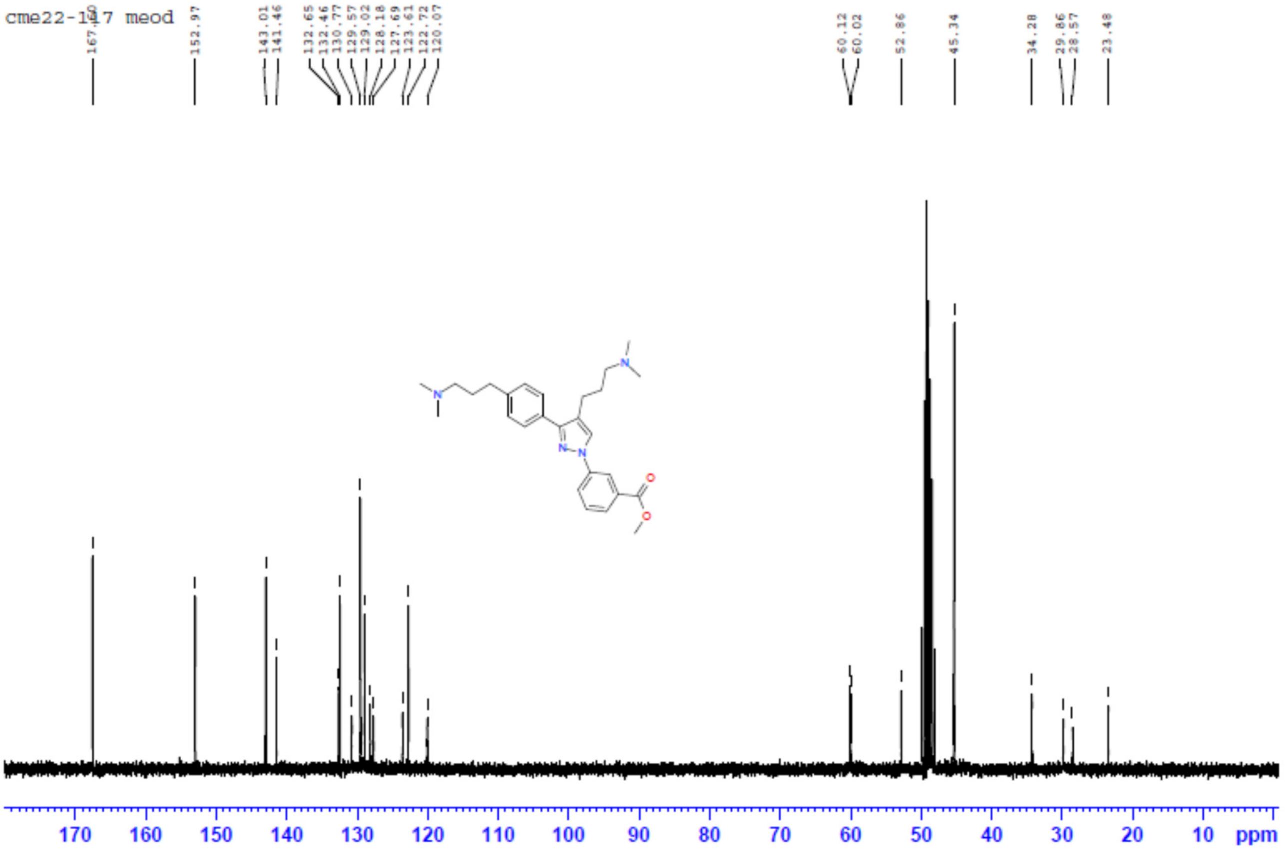

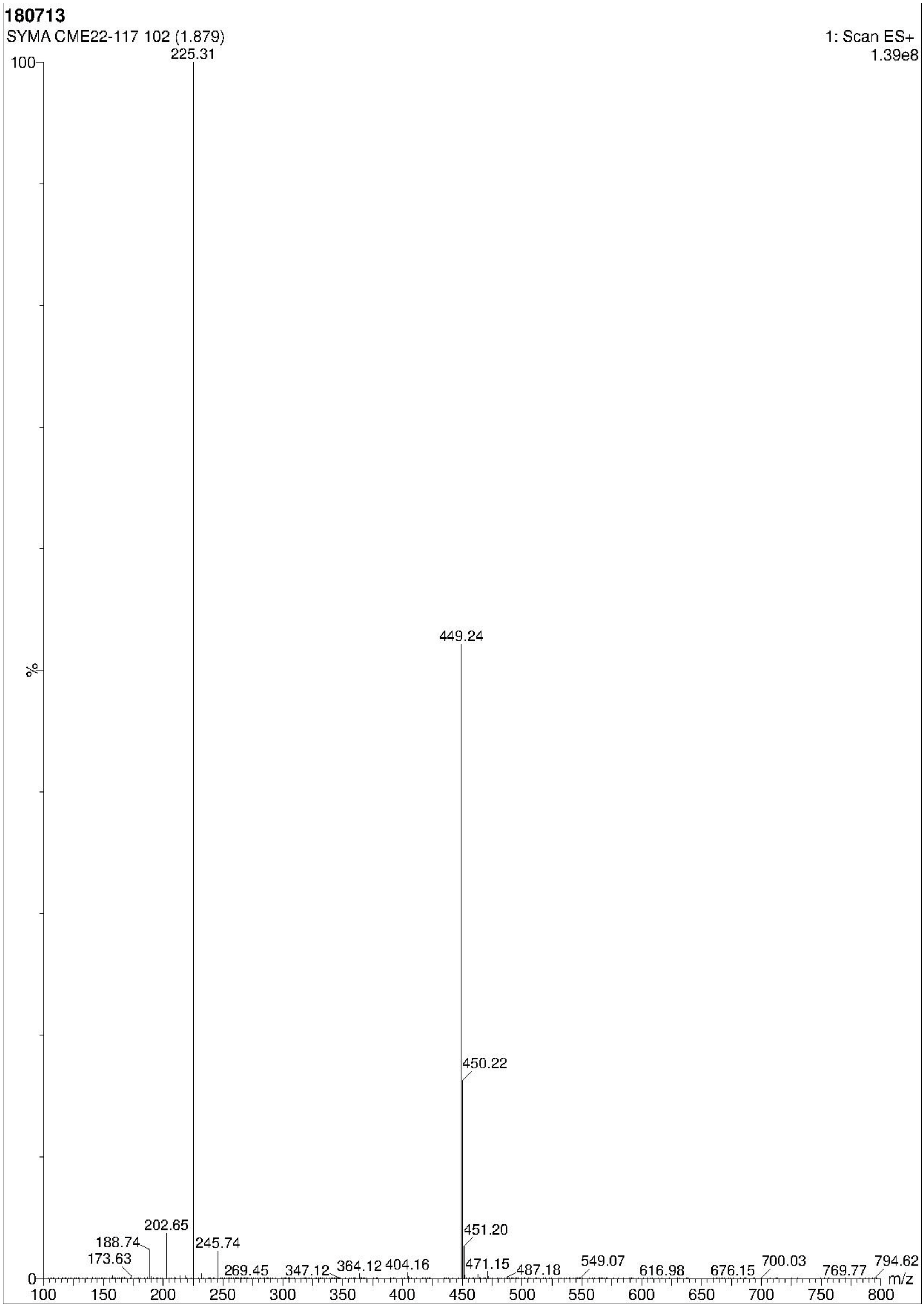

180713
SYMA CME22-117 102 (1.879)
1: Scan ES+
1.39e8
100
%
0
225.31
449.24
450.22
451.20
202.65
188.74
173.63
245.74
269.45
347.12
364.12
404.16
471.15
487.18
549.07
616.98
676.15
700.03
769.77
794.62
m/z
100
150
200
250
300
350
400
450
500
550
600
650
700
750
800

[4-[4-(dimethylaminomethyl)-3-[4-[3-(dimethylamino)propyl]phenyl]pyrazol-1-yl]phenyl]methanol (**16**)

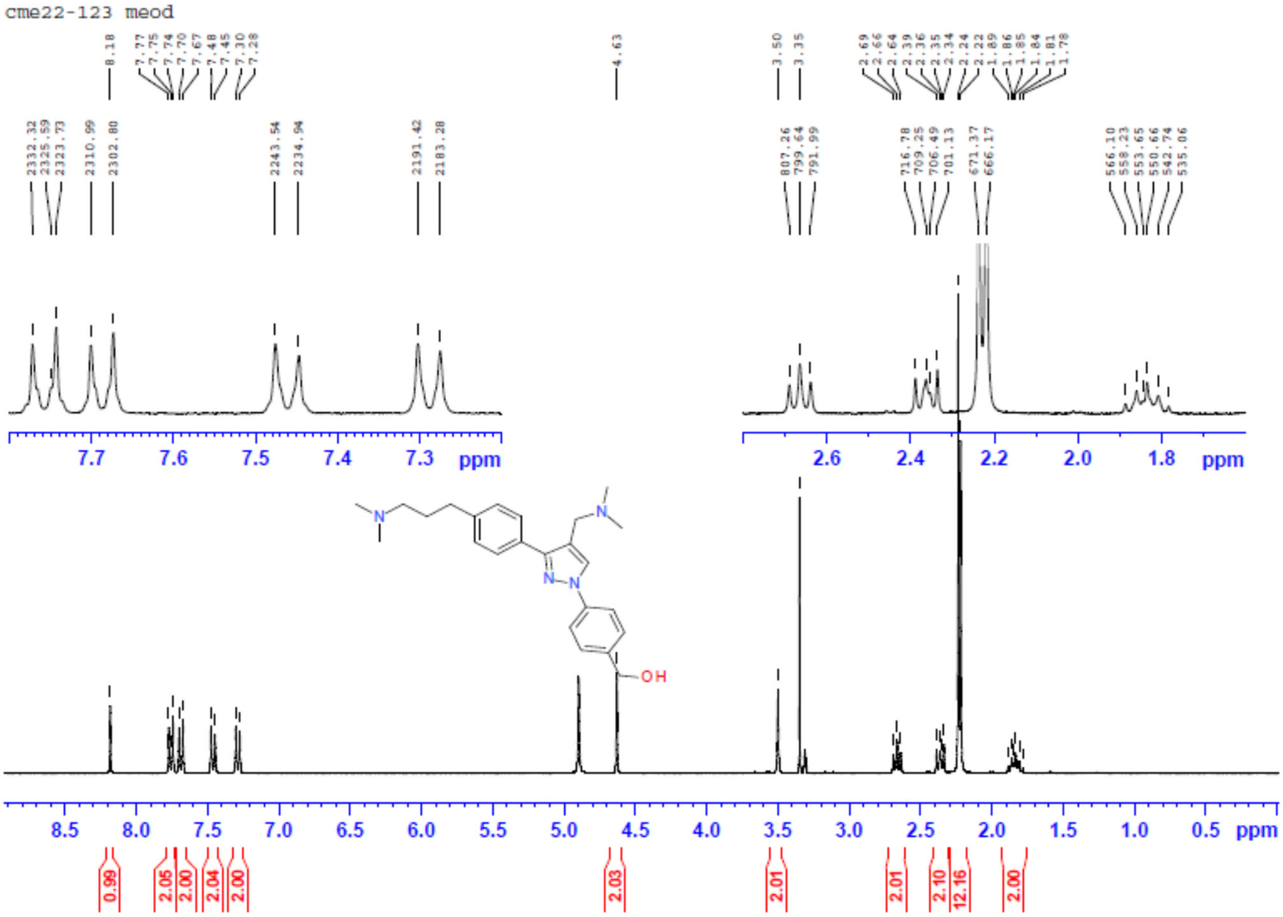

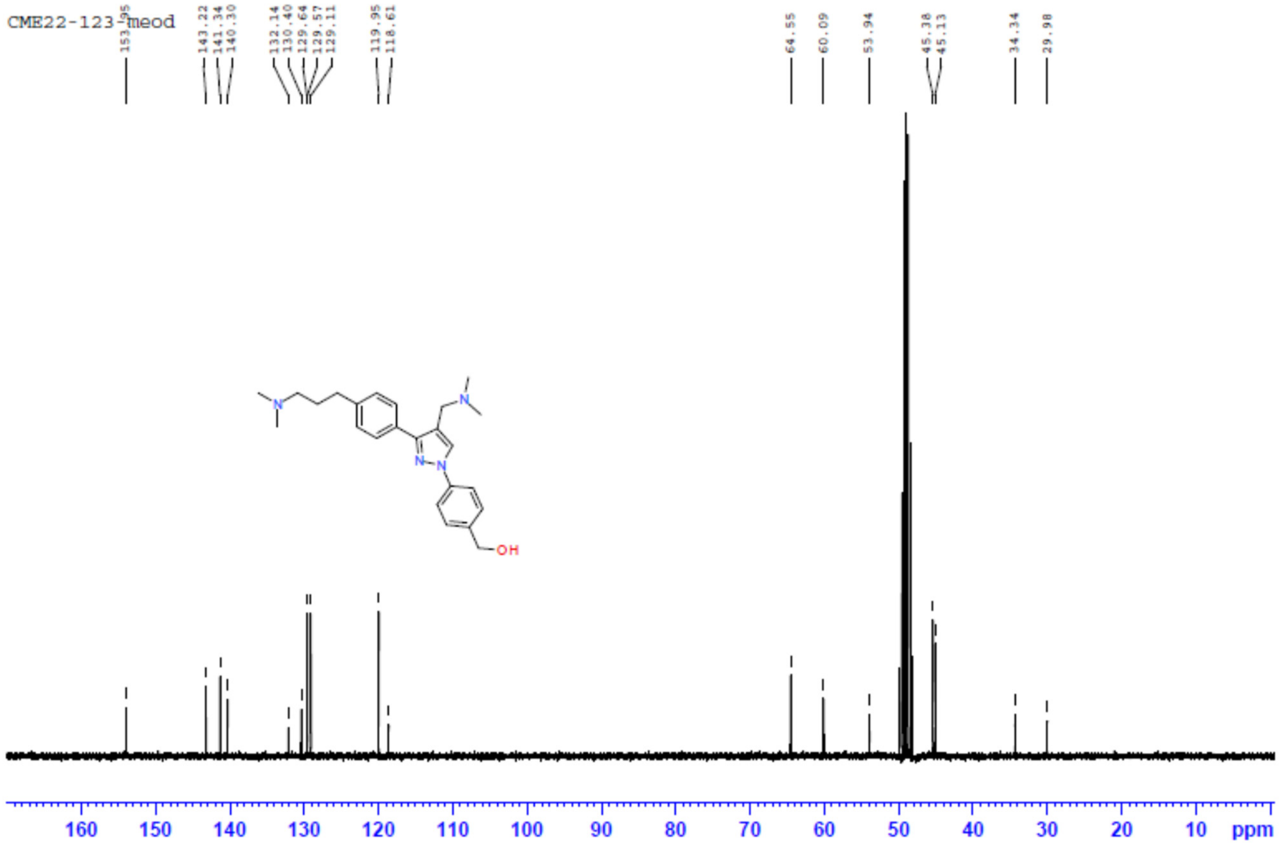

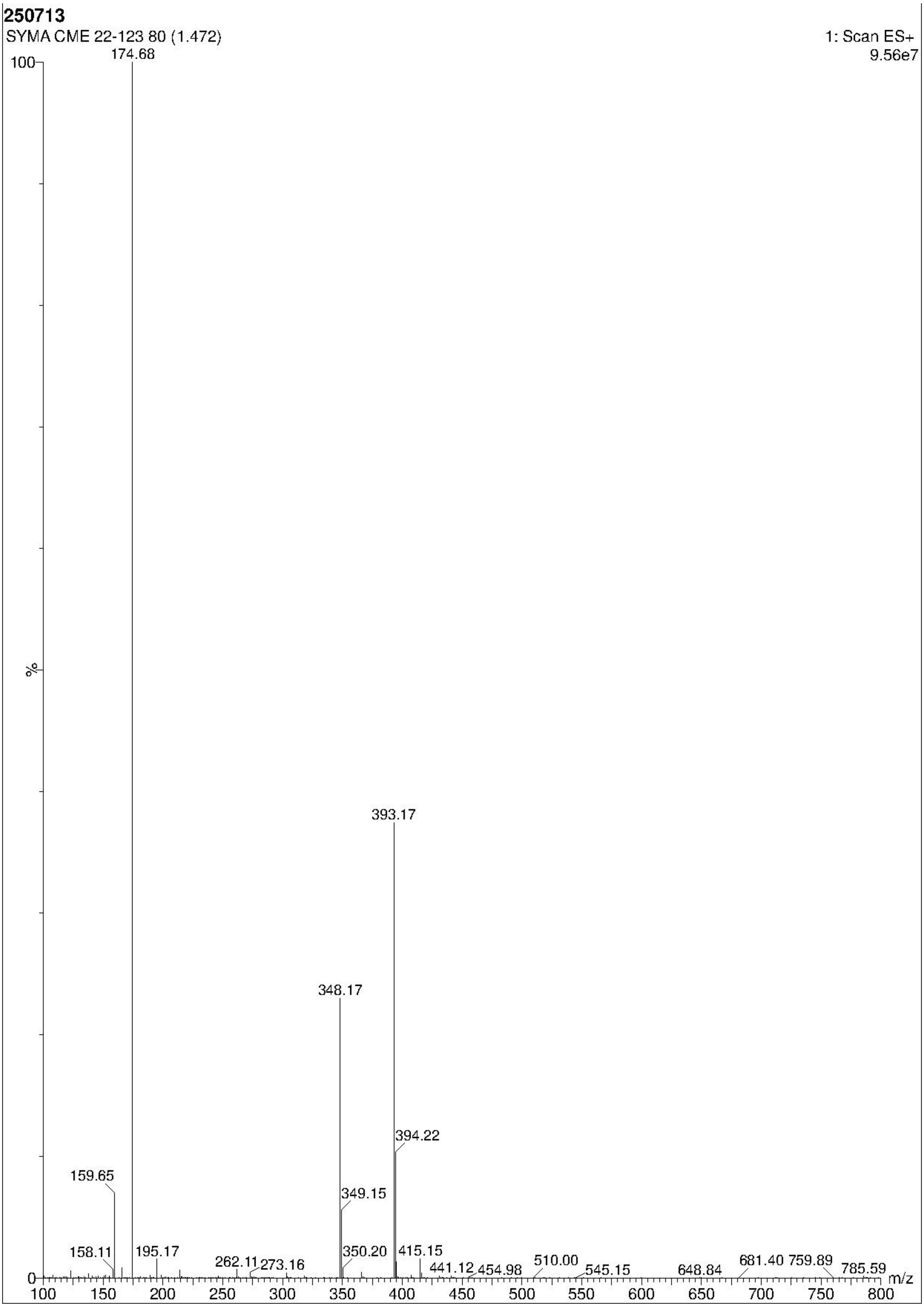
250713
SYMA CME 22-123 80 (1.472)
1: Scan ES+
9.56e7
100
%
0
174.68
393.17
348.17
394.22
159.65
349.15
158.11
195.17
262.11
273.16
350.20
415.15
441.12
454.98
510.00
545.15
648.84
681.40
759.89
785.59
m/z
100
150
200
250
300
350
400
450
500
550
600
650
700
750
800

[4-[4-[3-(dimethylamino)propyl]-3-[4-[3-(dimethylamino)propyl]phenyl]pyrazol-1-yl]phenyl] methanol (**17**)

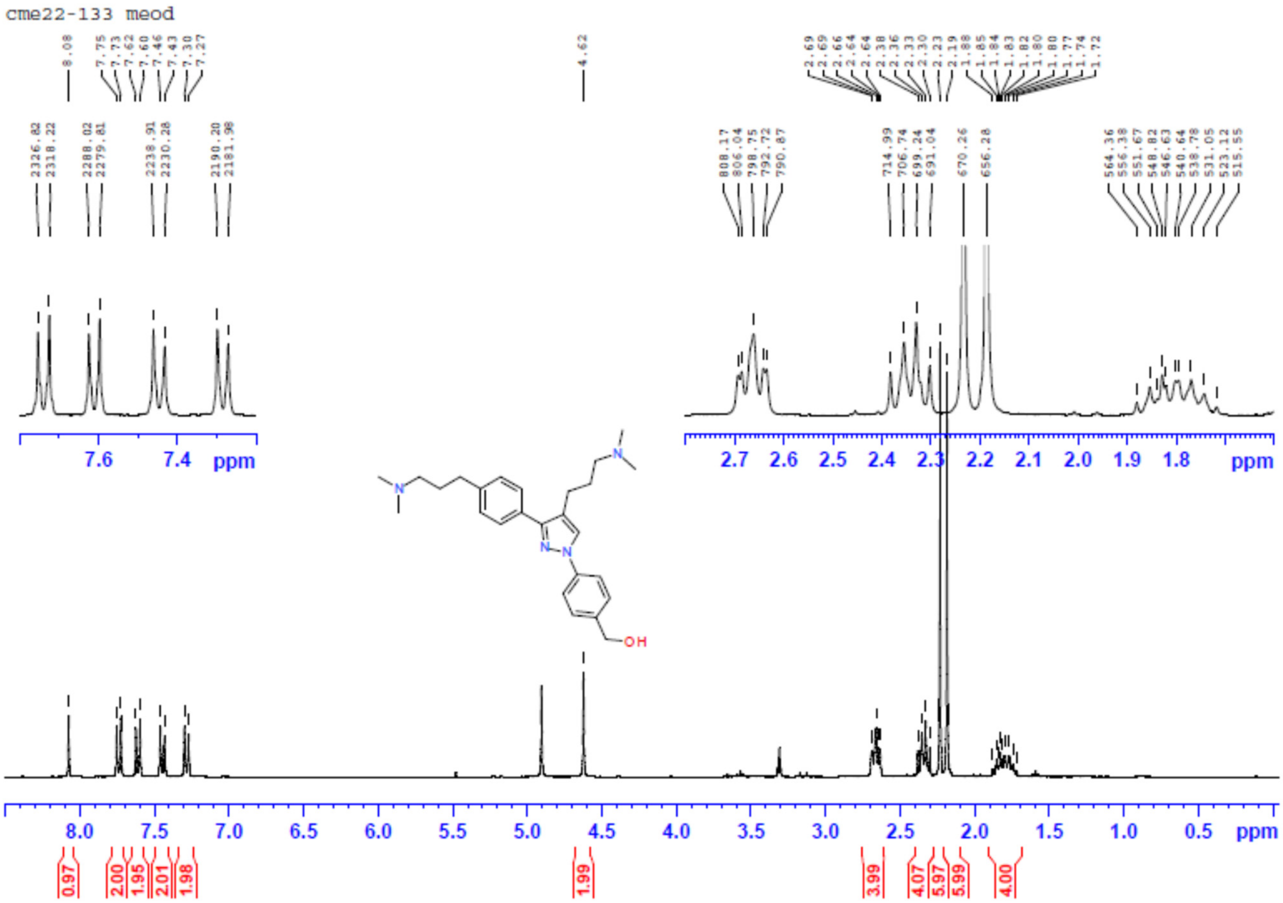

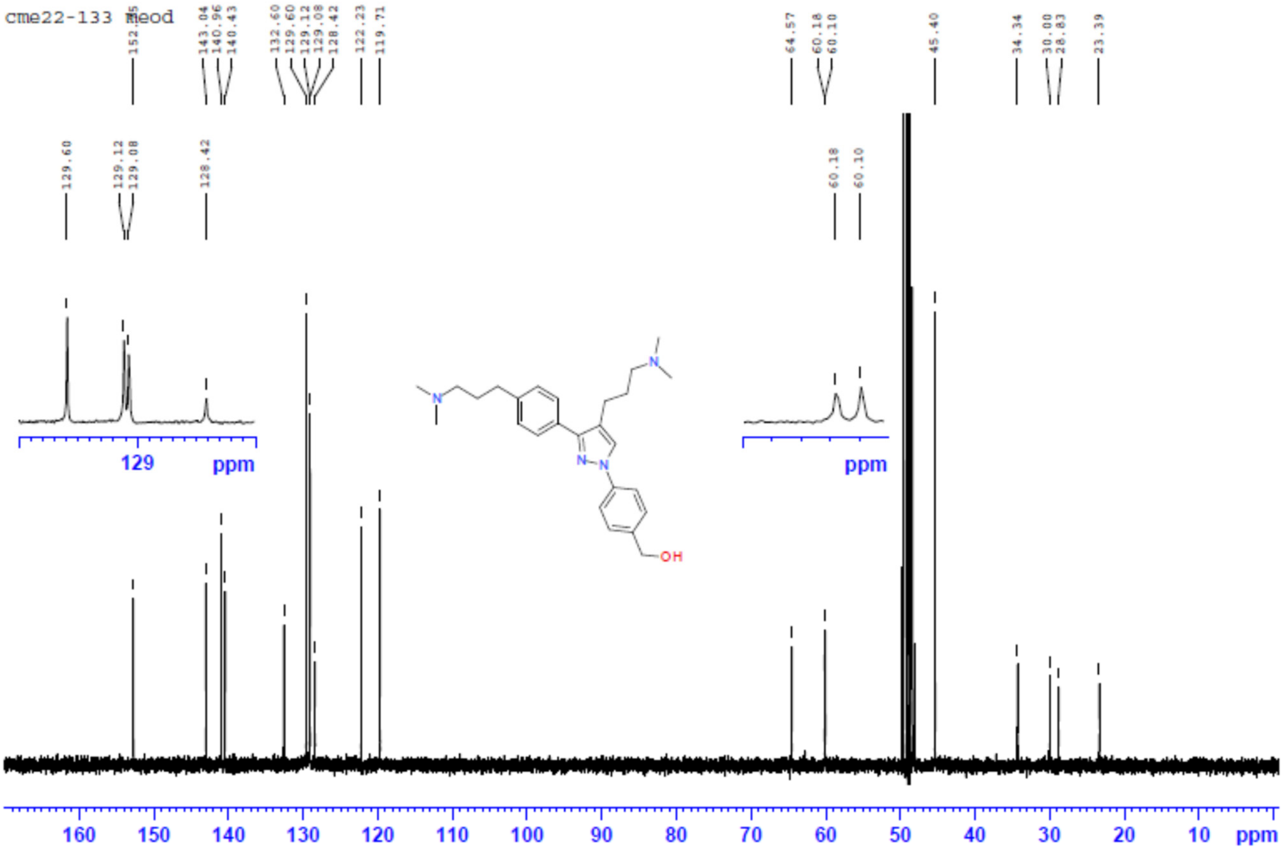

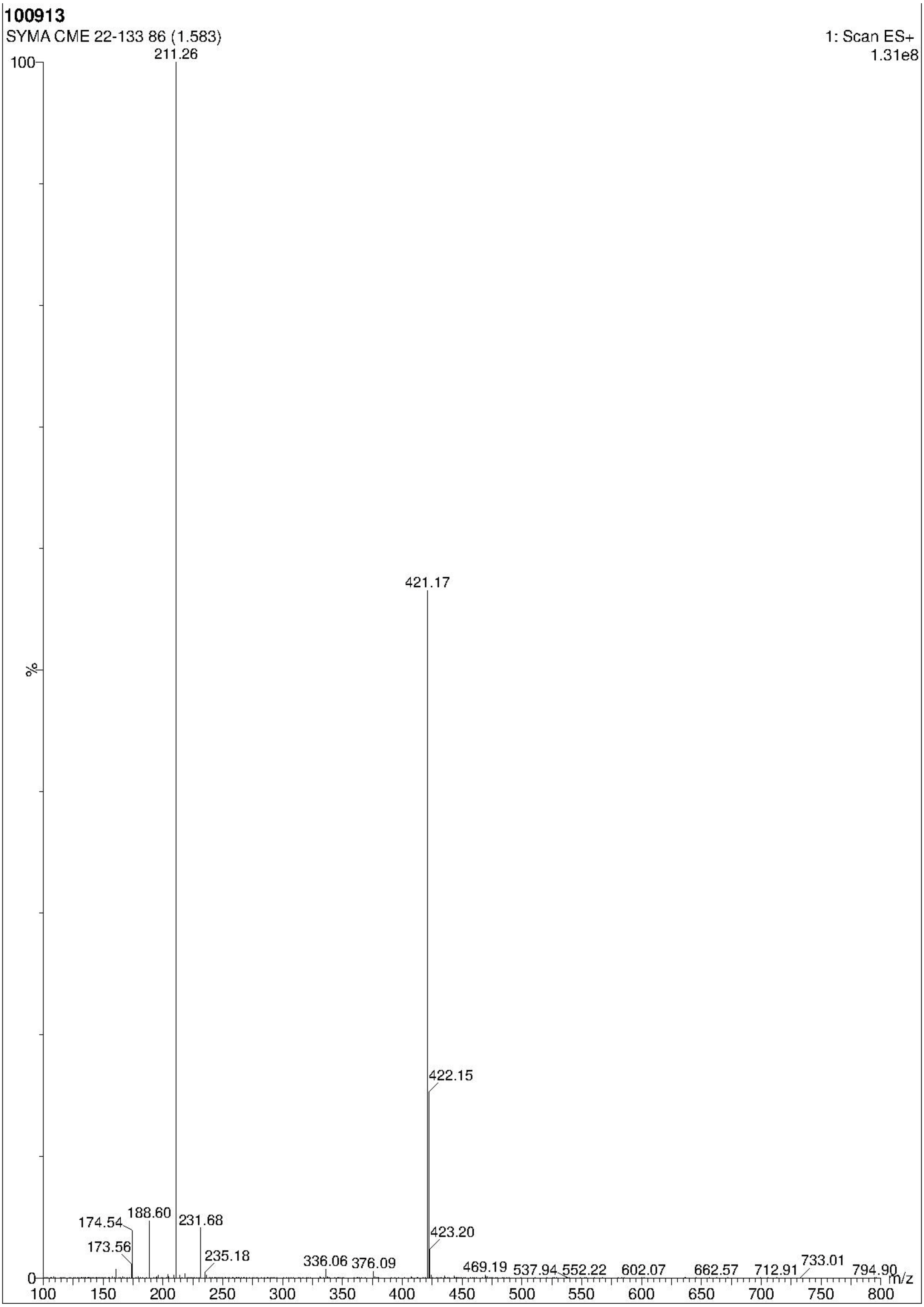

100913
SYMA CME 22-133 86 (1.583)
1: Scan ES+
1.31e8
211.26
421.17
422.15
423.20
174.54
188.60
231.68
173.56
235.18
336.06
376.09
469.19
537.94
552.22
602.07
662.57
712.91
733.01
794.90
%
m/z
100
0
100 150 200 250 300 350 400 450 500 550 600 650 700 750 800

[3-[4-(dimethylaminomethyl)-3-[4-[3-(dimethylamino)propyl]phenyl]pyrazol-1-yl]phenyl]methanol (**18**)

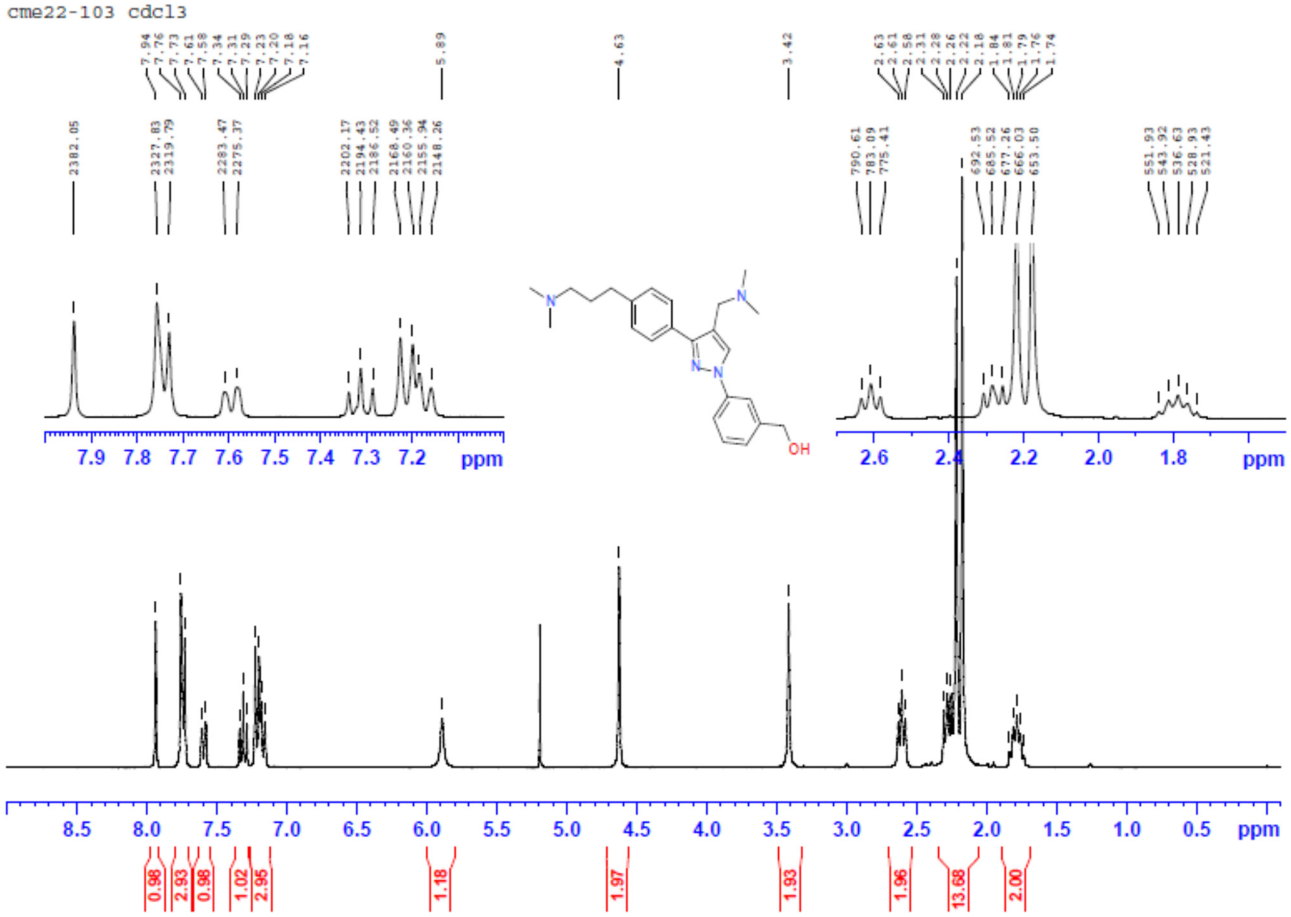

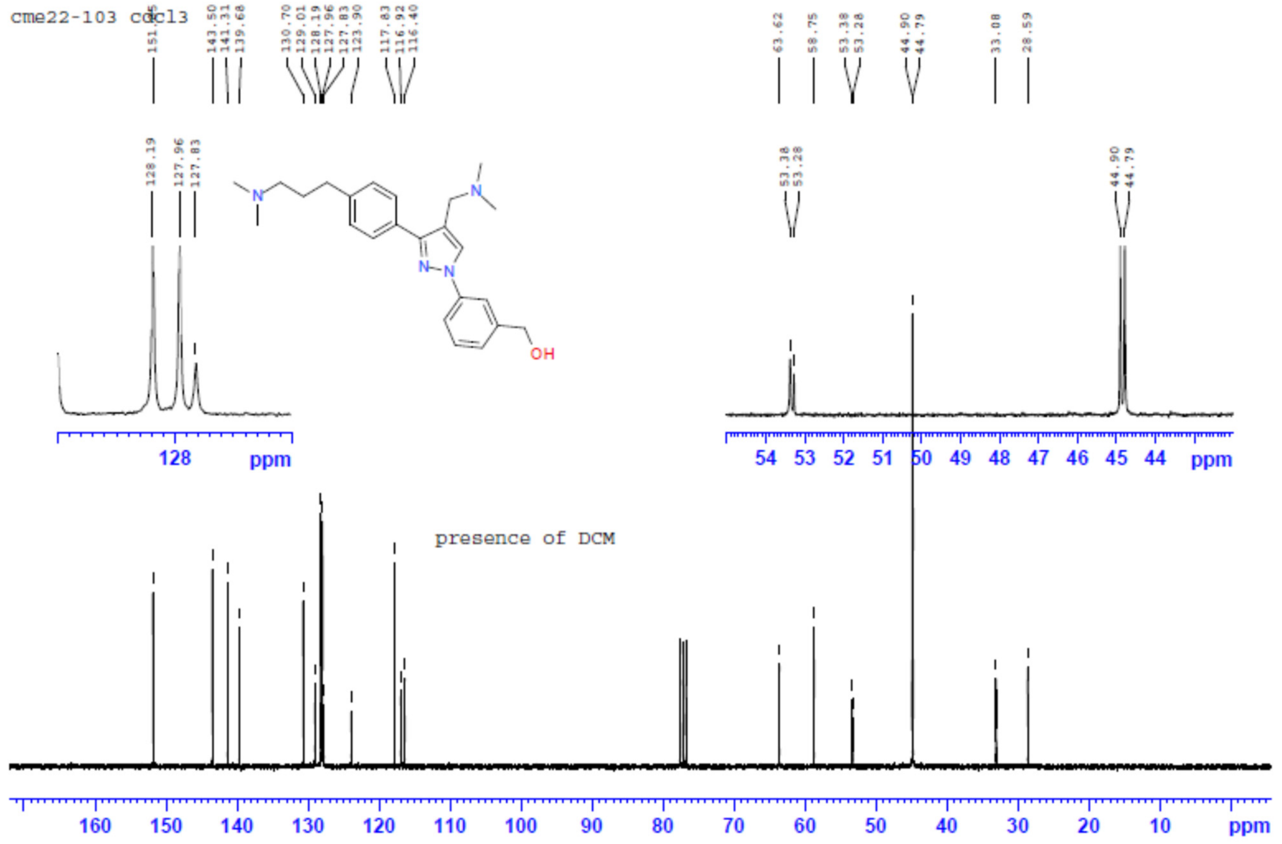

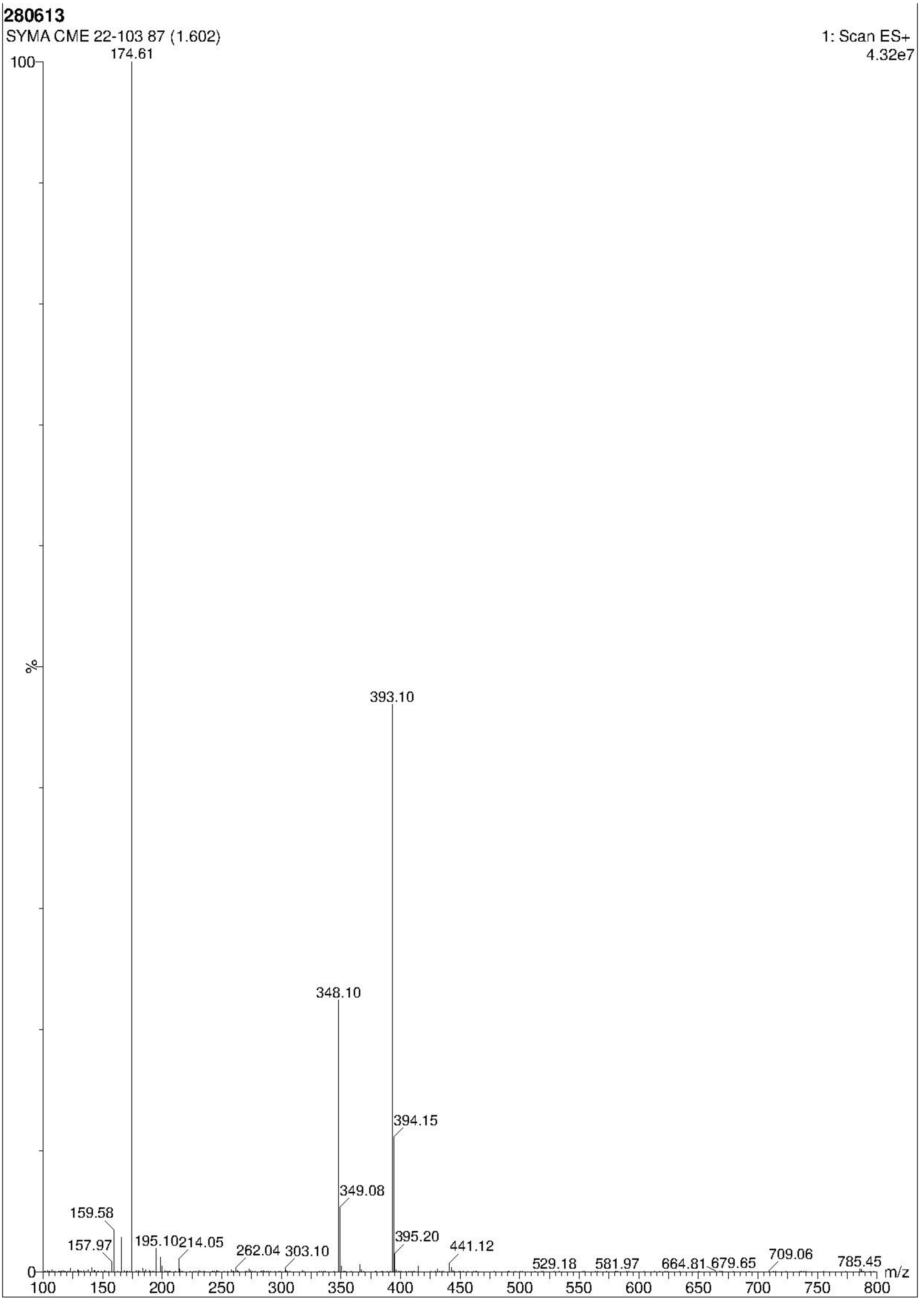

280613
SYMA CME 22-103 87 (1.602)
1: Scan ES+
4.32e7
100
%
0
174.61
393.10
348.10
394.15
349.08
159.58
157.97
195.10
214.05
262.04
303.10
395.20
441.12
529.18
581.97
664.81
679.65
709.06
785.45
m/z
100 150 200 250 300 350 400 450 500 550 600 650 700 750 800

[3-[4-[3-(dimethylamino)propyl]-3-[4-[3-(dimethylamino)propyl]phenyl]pyrazol-1-yl]phenyl]methanol (**19**)

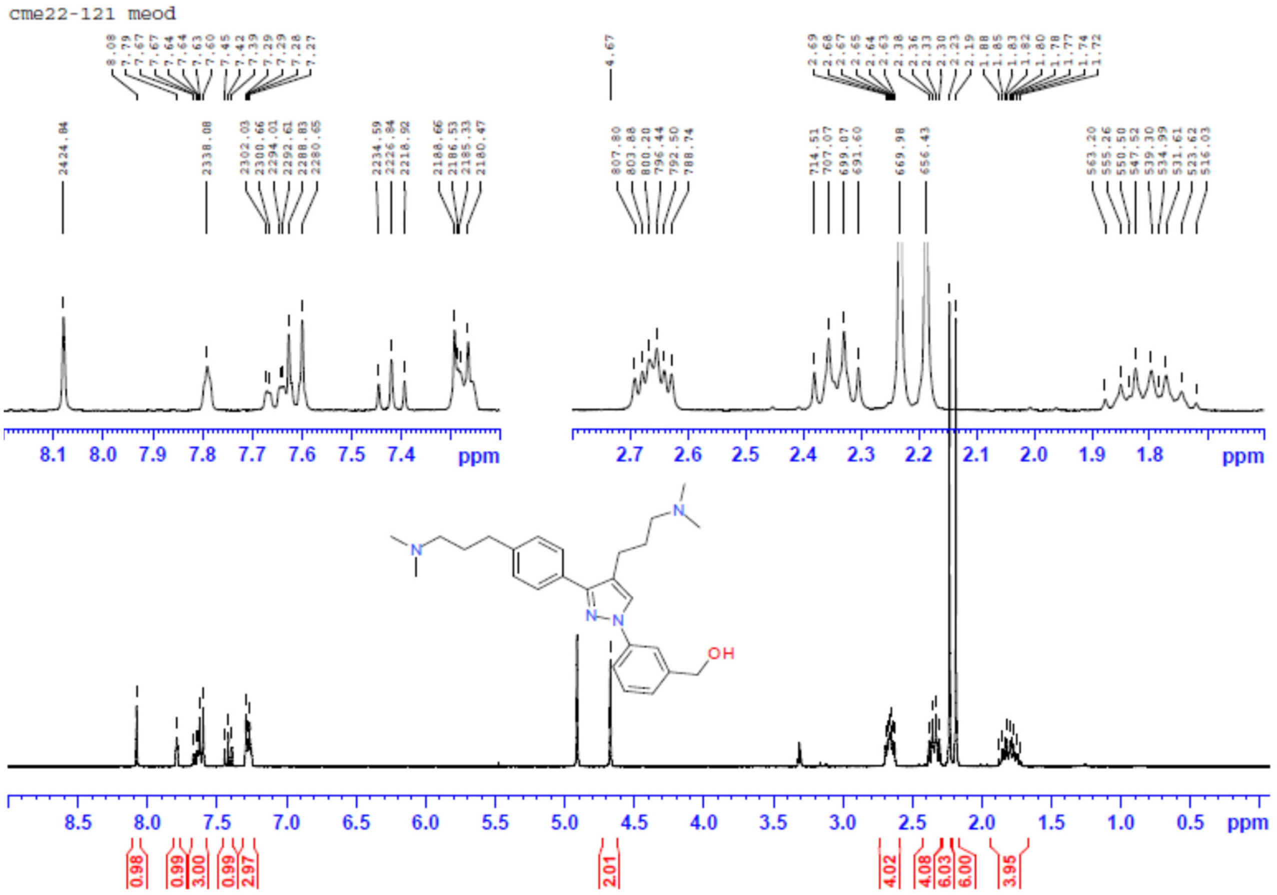

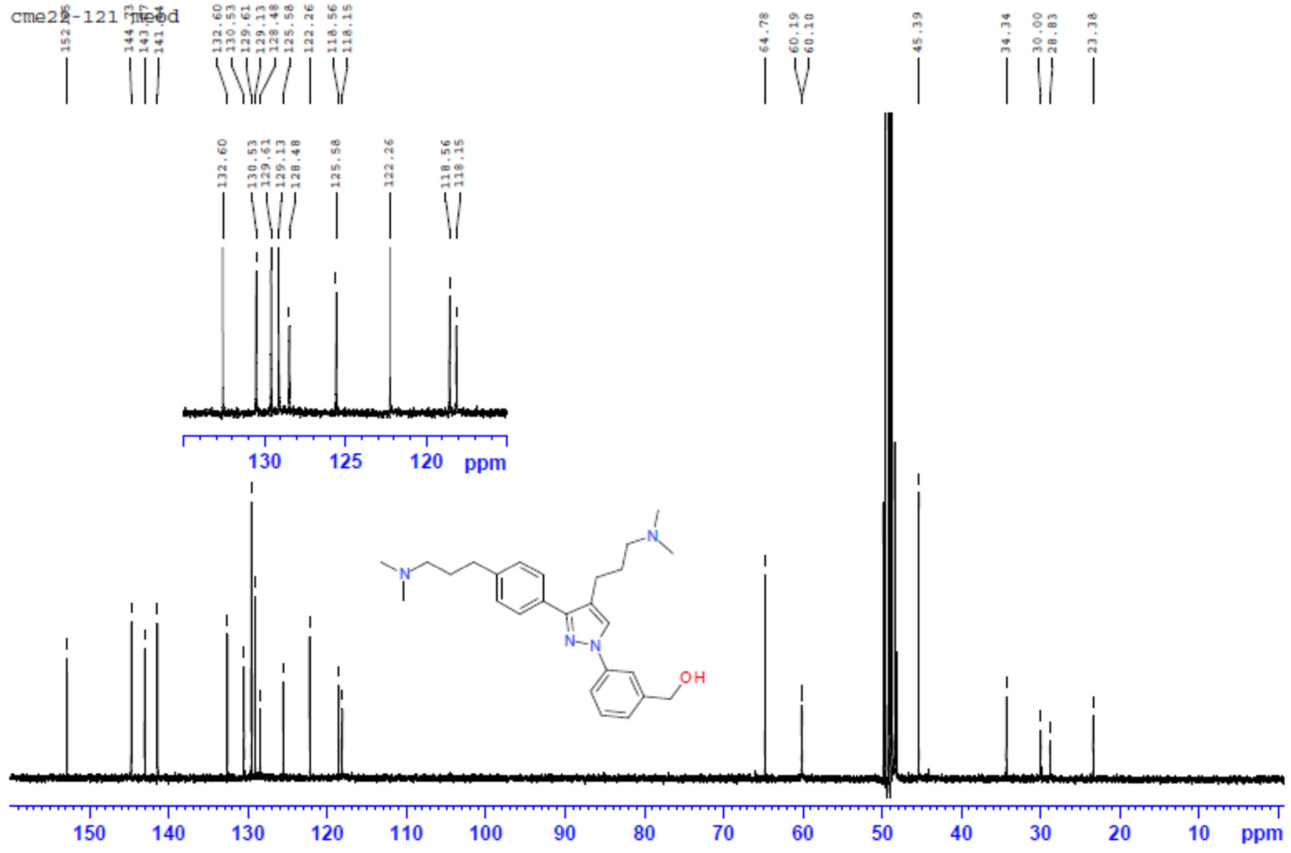

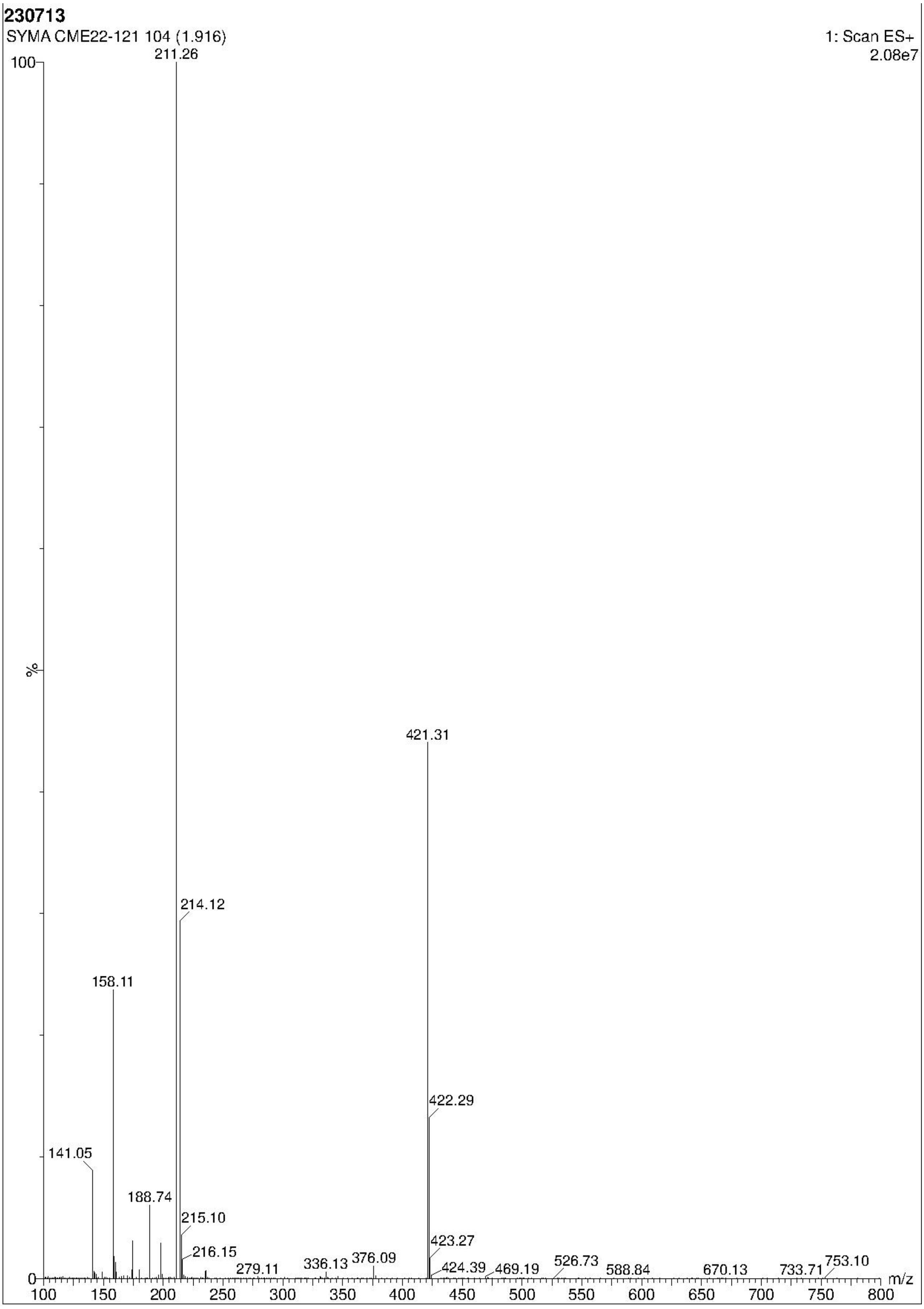
230713
SYMA CME22-121 104 (1.916)
1: Scan ES+
2.08e7
100
%
0
211.26
421.31
214.12
158.11
422.29
141.05
188.74
215.10
423.27
216.15
279.11
336.13
376.09
424.39
469.19
526.73
588.84
670.13
733.71
753.10
m/z
100
150
200
250
300
350
400
450
500
550
600
650
700
750
800

4-[4-(dimethylaminomethyl)-3-[4-[3-(dimethylamino)propyl]phenyl]pyrazol-1-yl]benzaldehyde (**20**)

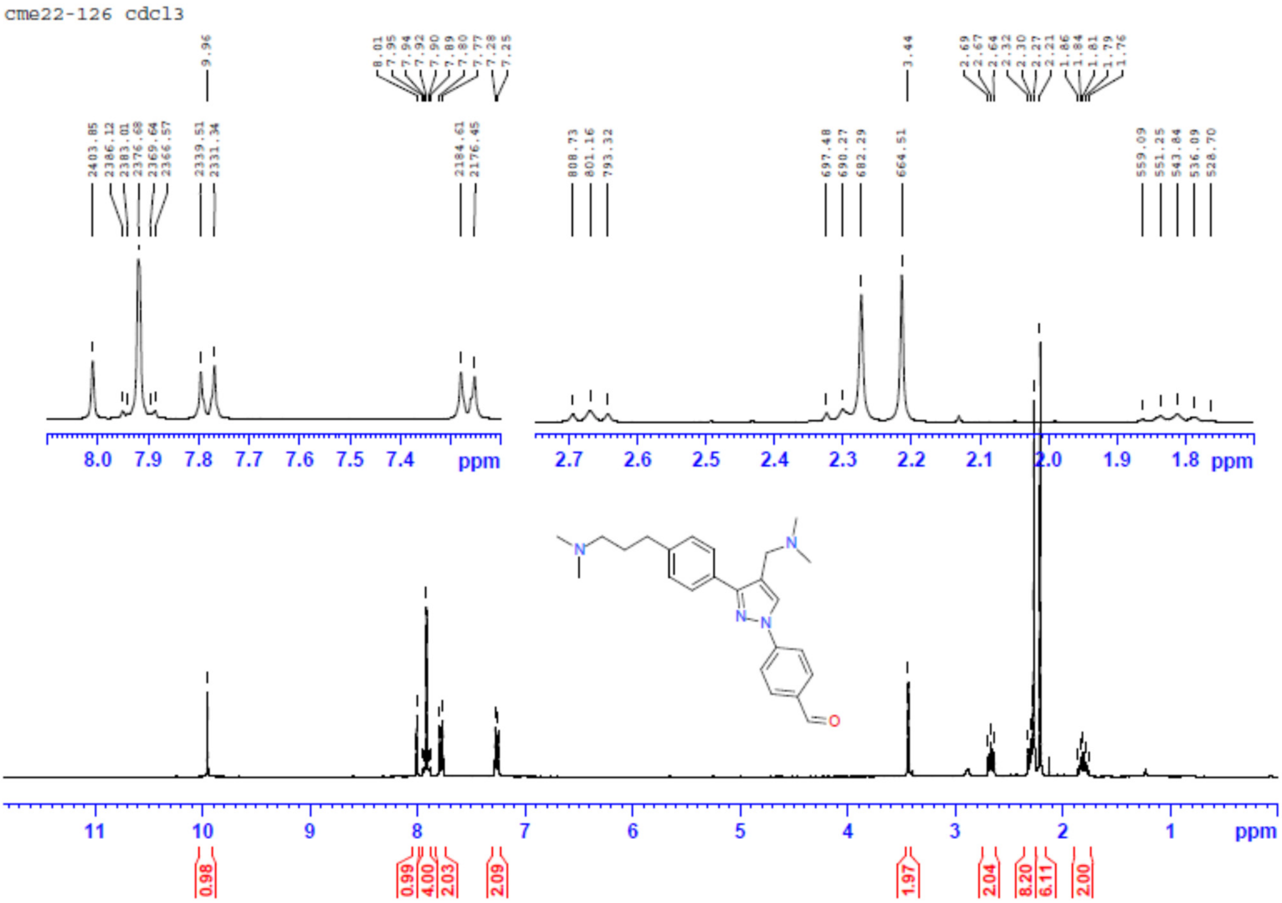

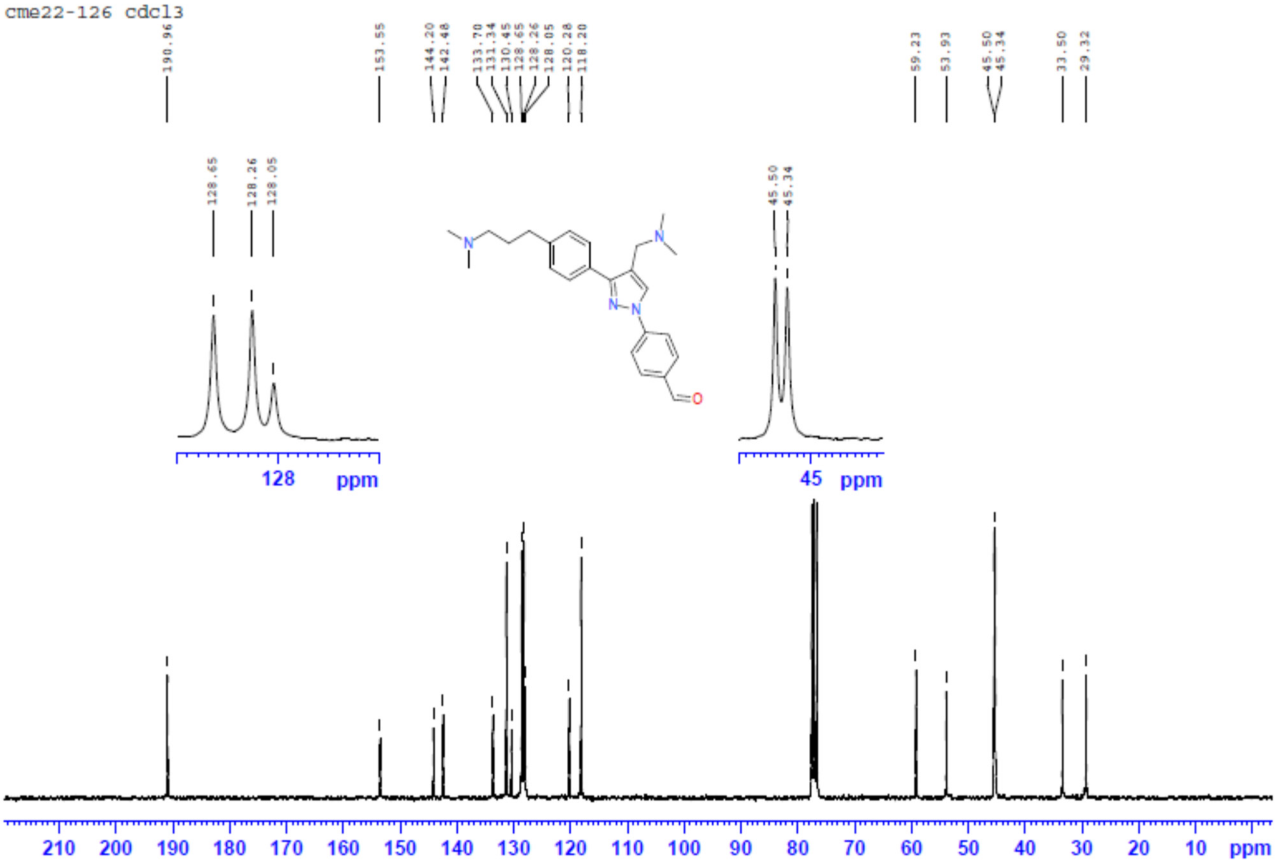

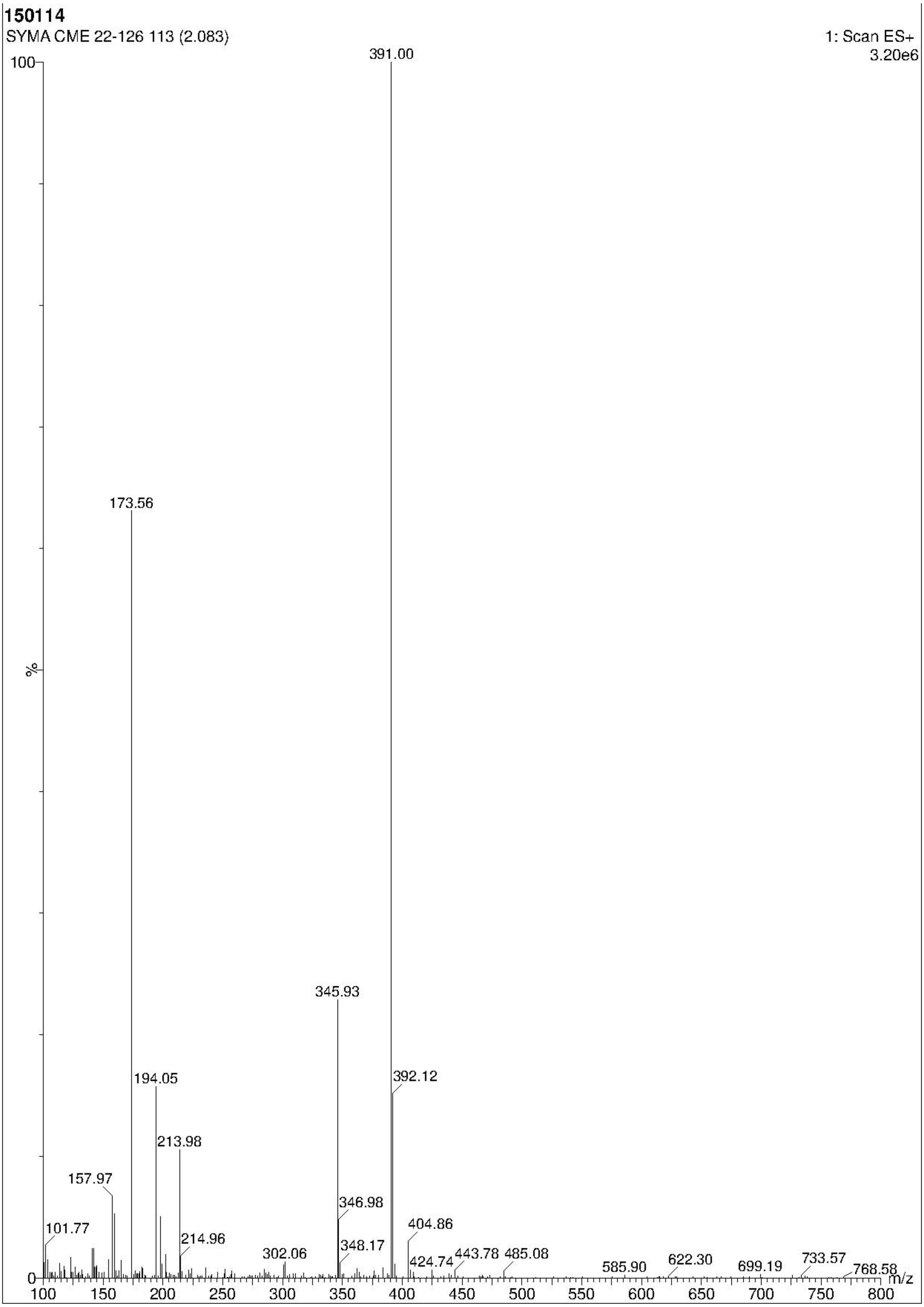
150114
SYMA CME 22-126 113 (2.083)
1: Scan ES+
3.20e6
100
%
0
391.00
173.56
345.93
194.05
392.12
213.98
157.97
346.98
404.86
101.77
214.96
348.17
302.06
424.74
443.78
485.08
585.90
622.30
699.19
733.57
768.58
m/z
100
150
200
250
300
350
400
450
500
550
600
650
700
750
800

4-[4-[3-(dimethylamino)propyl]-3-[4-[3-(dimethylamino)propyl]phenyl]pyrazol-1-yl]benzaldehyde (**21**)

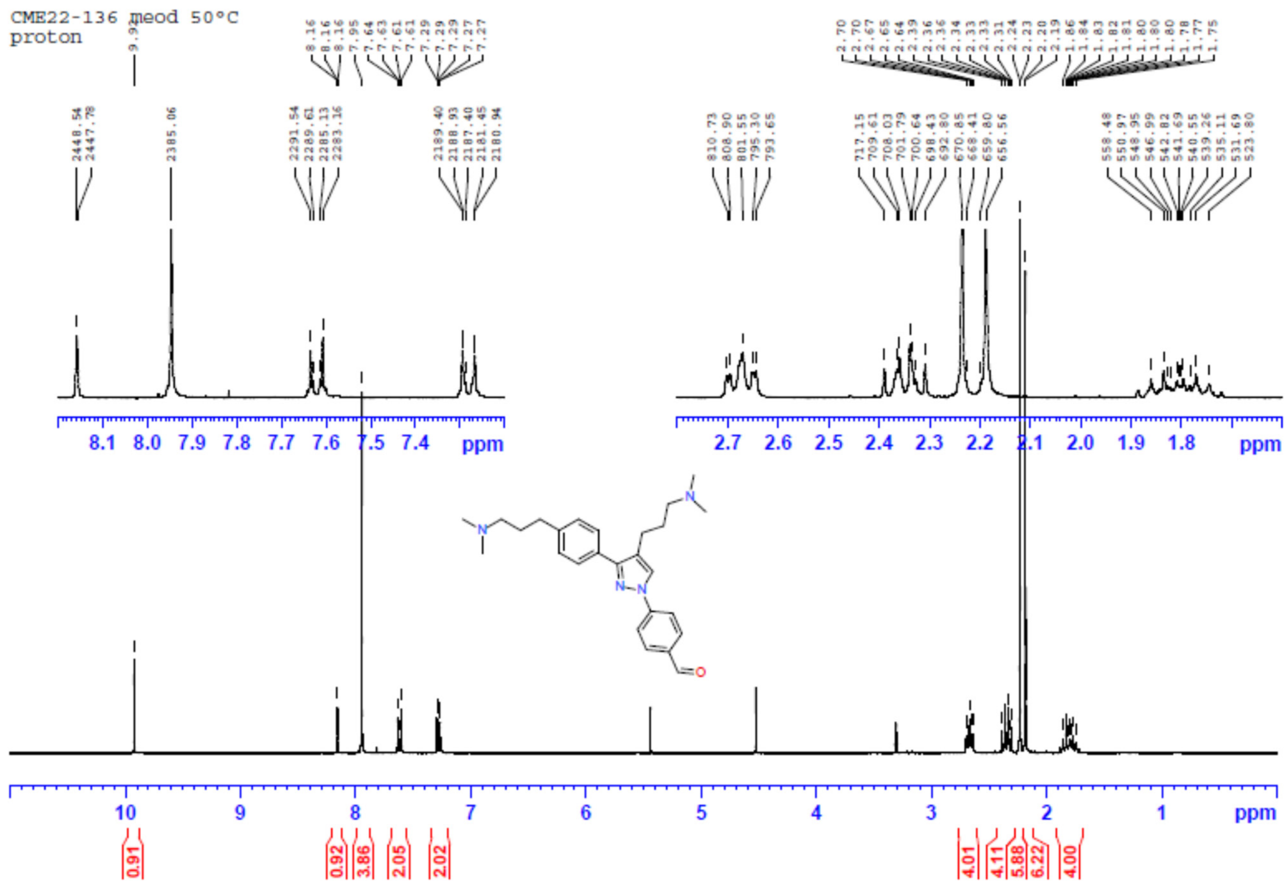

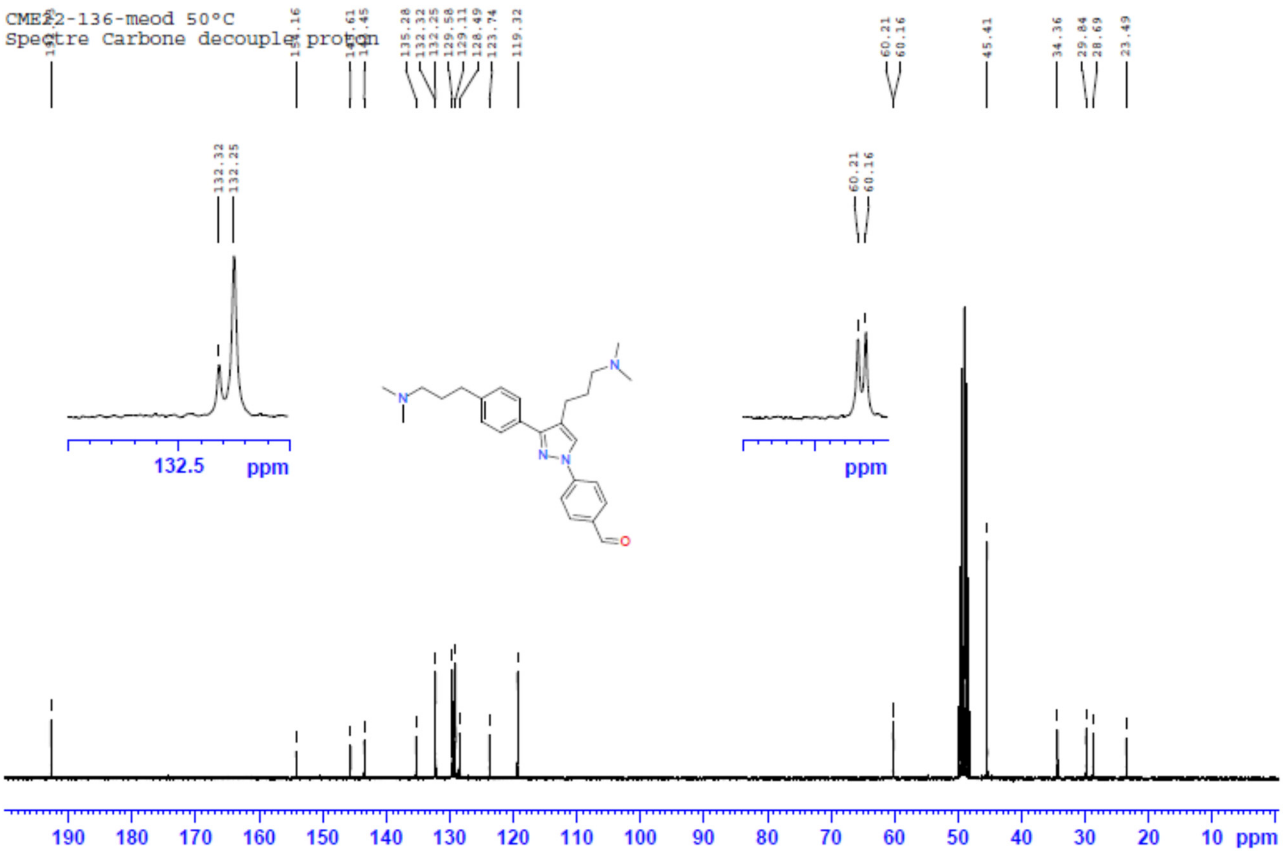

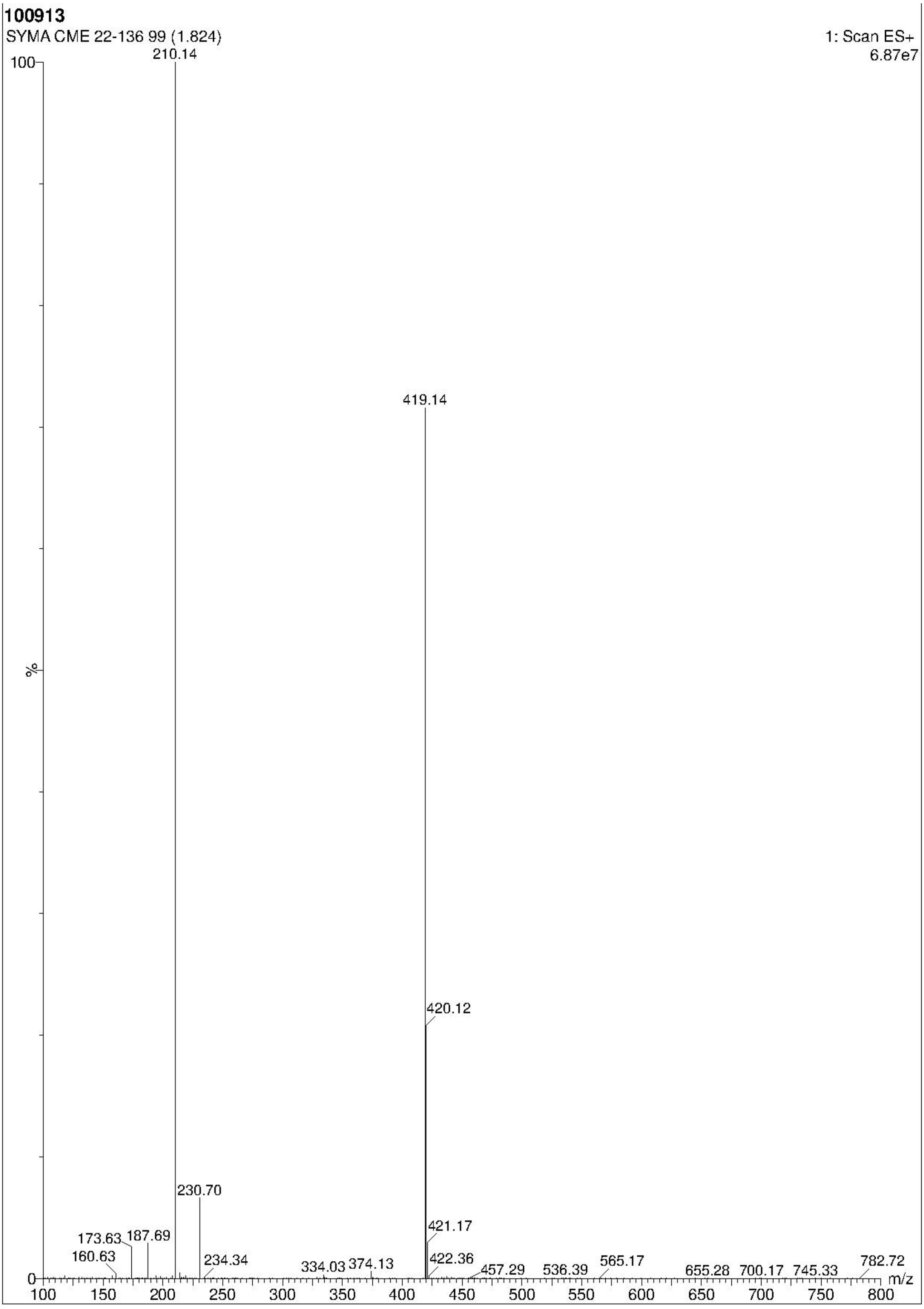
100913
SYMA CME 22-136 99 (1.824)
1: Scan ES+
6.87e7
100
%
0
210.14
419.14
420.12
230.70
421.17
173.63
187.69
160.63
234.34
422.36
334.03
374.13
457.29
536.39
565.17
655.28
700.17
745.33
782.72
m/z
100
150
200
250
300
350
400
450
500
550
600
650
700
750
800

3-[4-(dimethylaminomethyl)-3-[4-[3-(dimethylamino)propyl]phenyl]pyrazol-1-yl]benzaldehyde (**22**)

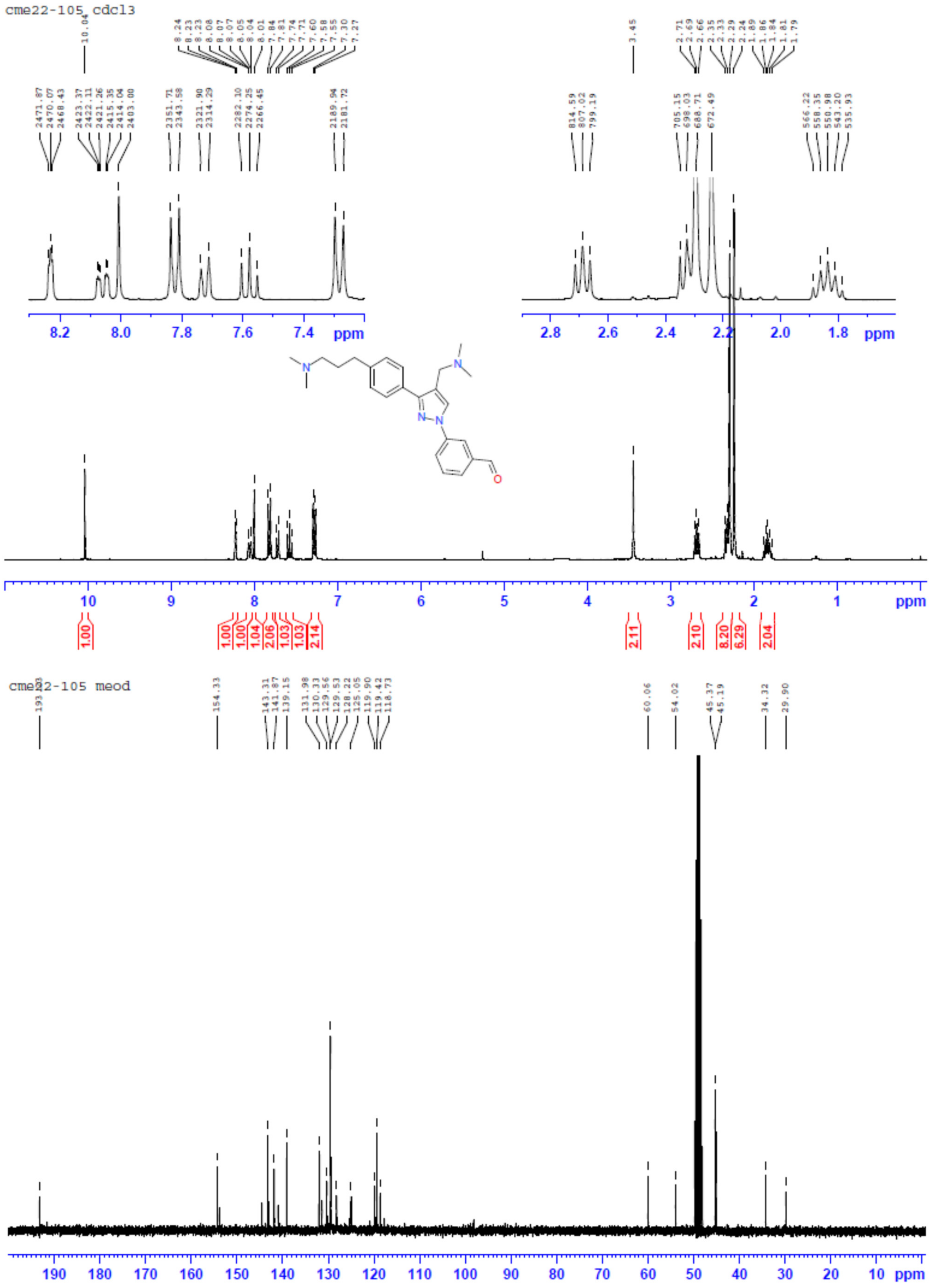

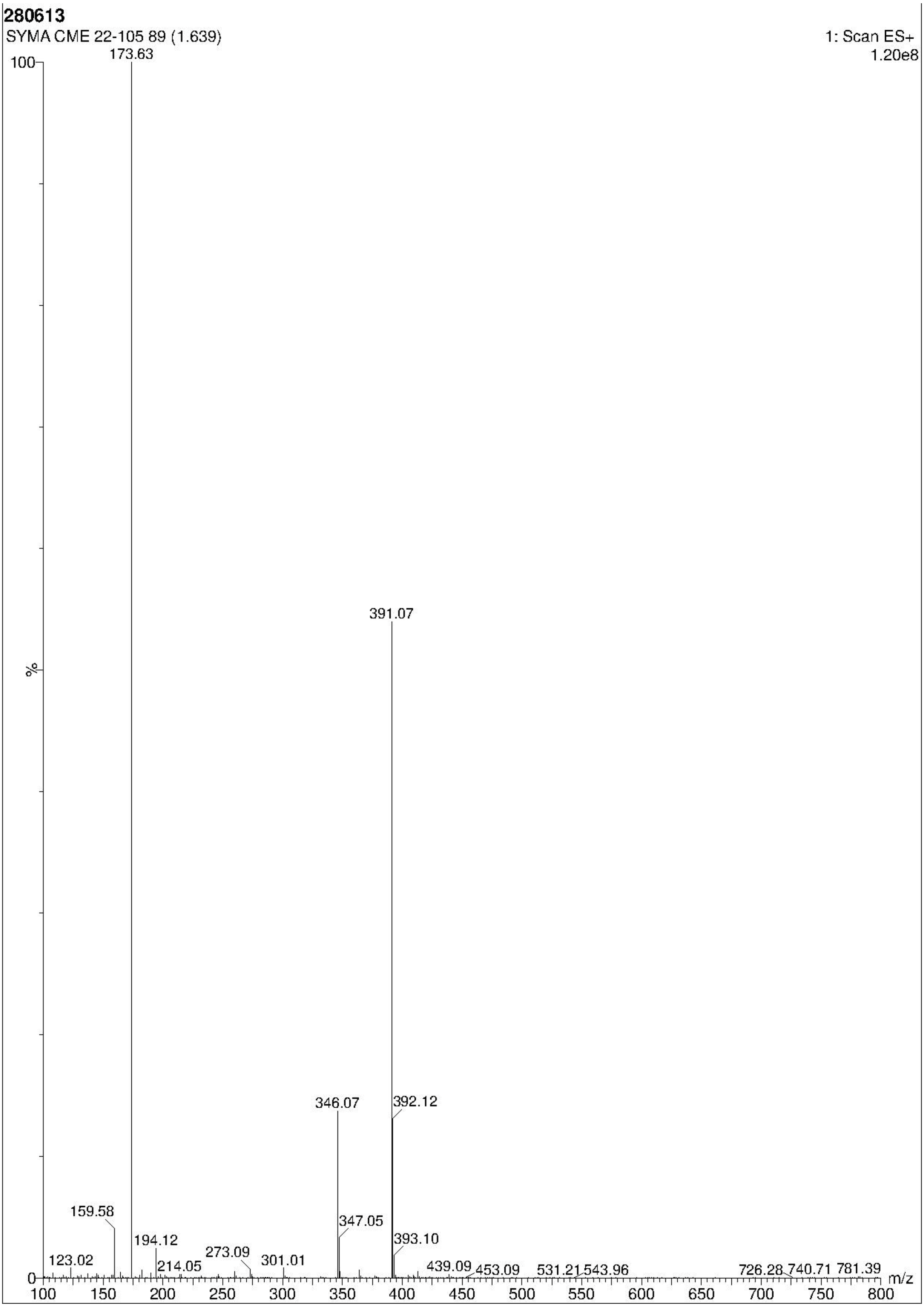
280613
SYMA CME 22-105 89 (1.639)
1: Scan ES+
1.20e8
100
%
0
173.63
391.07
346.07
392.12
159.58
347.05
194.12
393.10
123.02
214.05
273.09
301.01
439.09
453.09
531.21
543.96
726.28
740.71
781.39
m/z
100
150
200
250
300
350
400
450
500
550
600
650
700
750
800

3-[4-[3-(dimethylamino)propyl]-3-[4-[3-(dimethylamino)propyl]phenyl]pyrazol-1-yl]benzaldehyde (**23**)

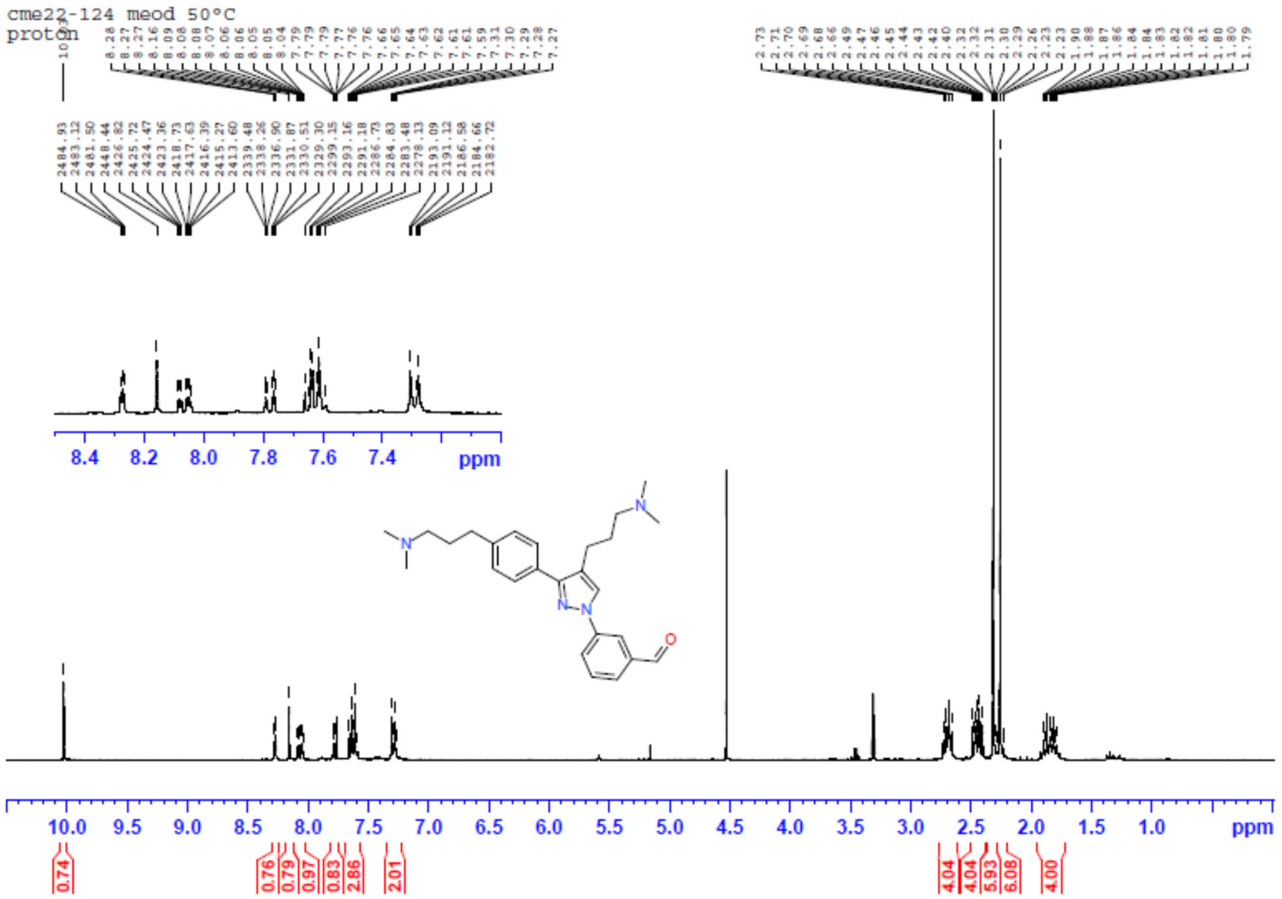

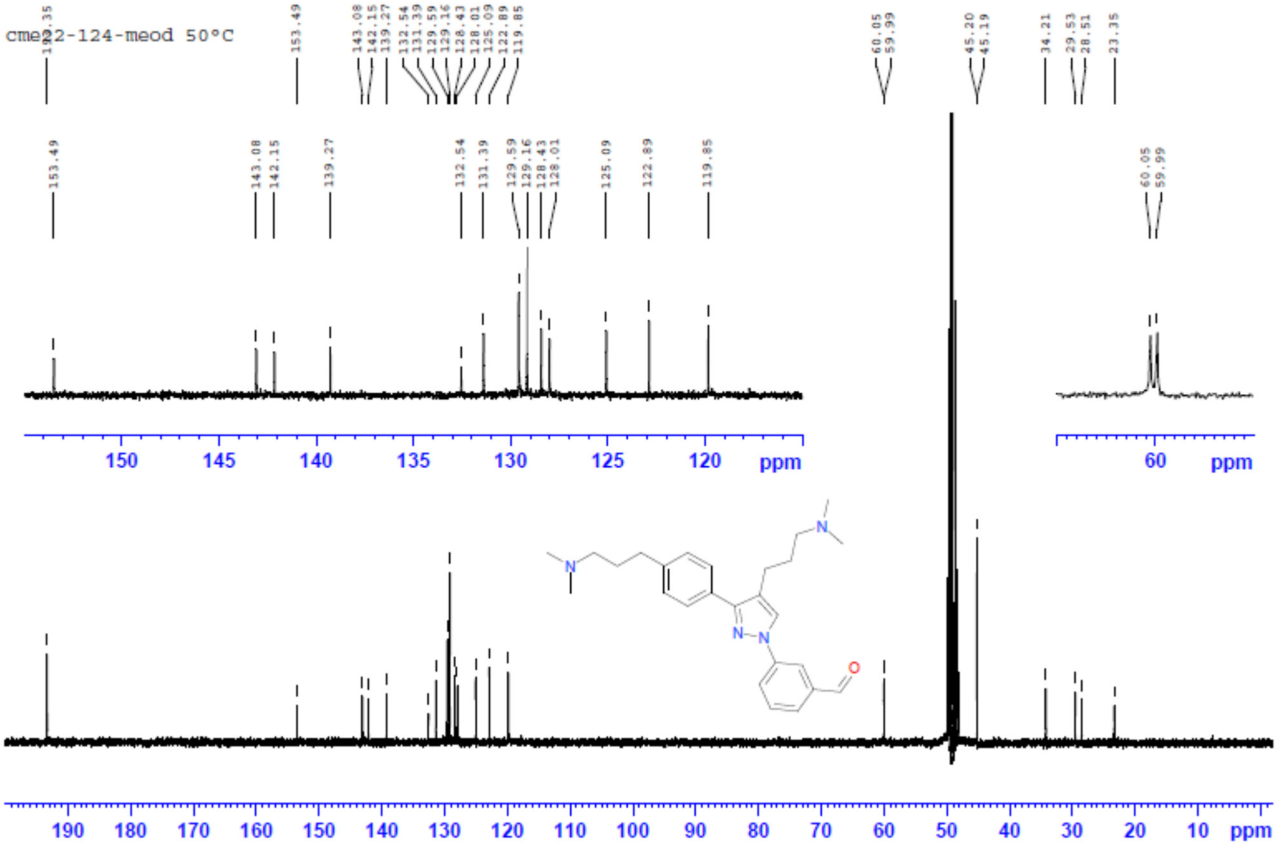

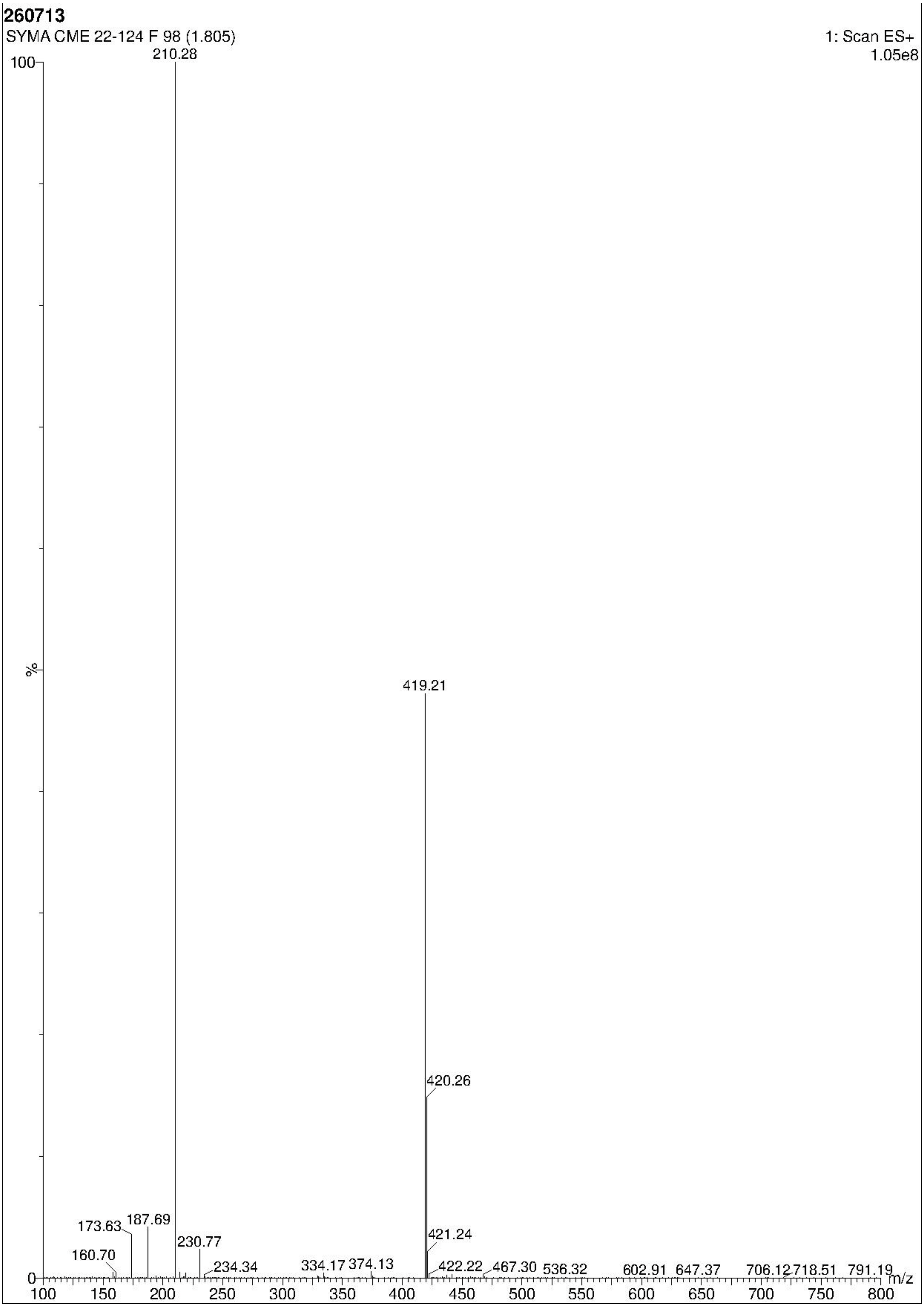
260713
SYMA CME 22-124 F 98 (1.805)
1: Scan ES+
1.05e8
100
%
0
210.28
419.21
420.26
421.24
422.22
173.63
187.69
160.70
230.77
234.34
334.17
374.13
467.30
536.32
602.91
647.37
706.12
718.51
791.19
m/z
100
150
200
250
300
350
400
450
500
550
600
650
700
750
800

3-[4-[4-(dimethylaminomethyl)-1-[4-(dimethylaminomethyl)phenyl]pyrazol-3-yl]phenyl]-*N*,*N*-dimethyl-propan-1-amine (**24**)

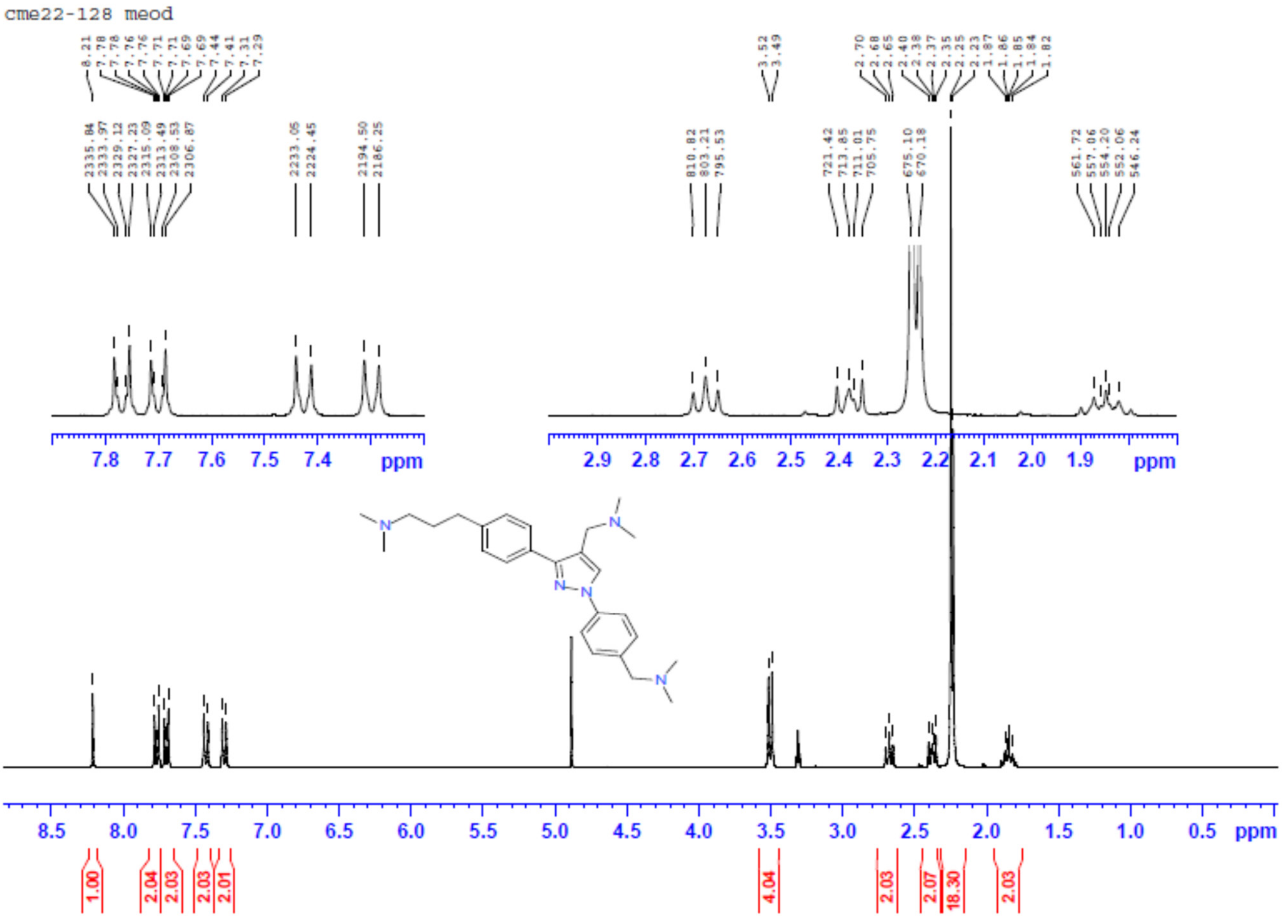

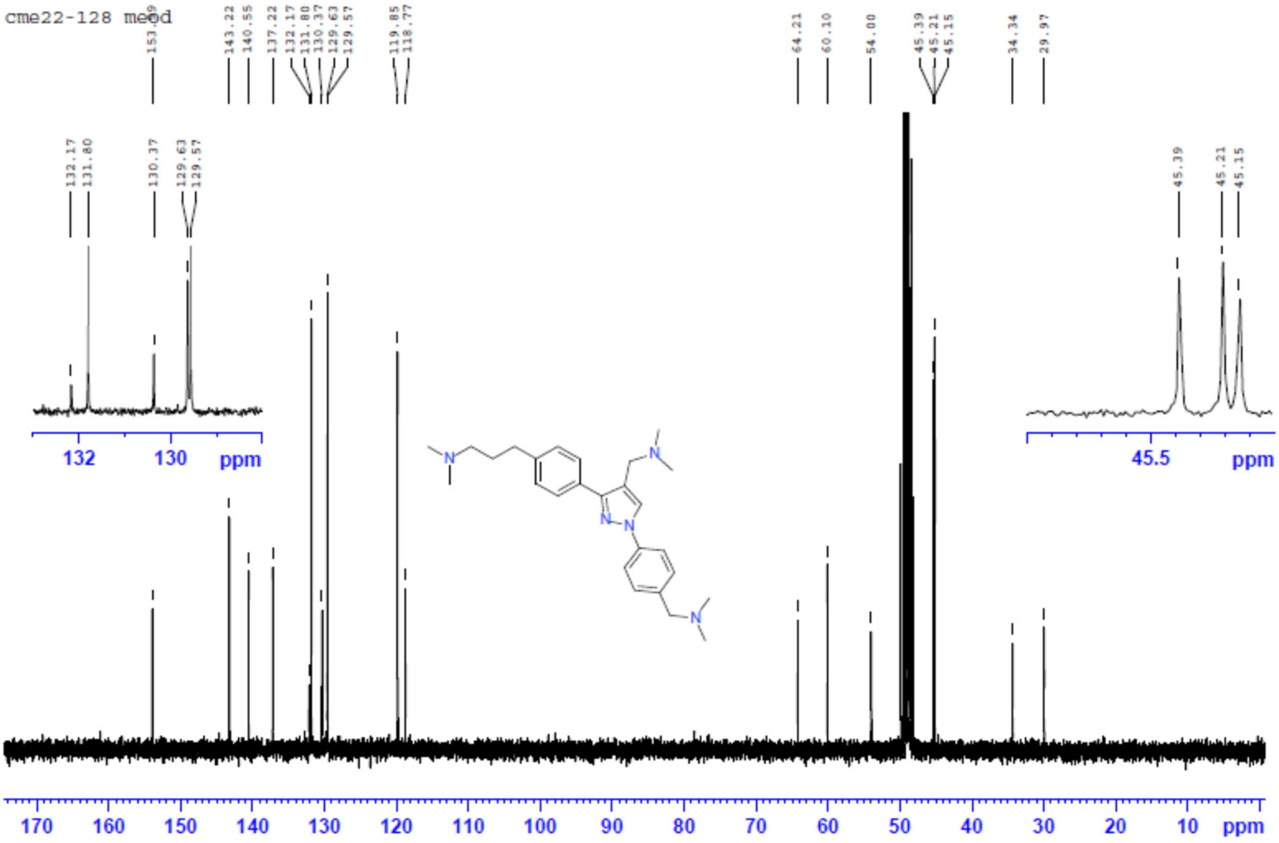

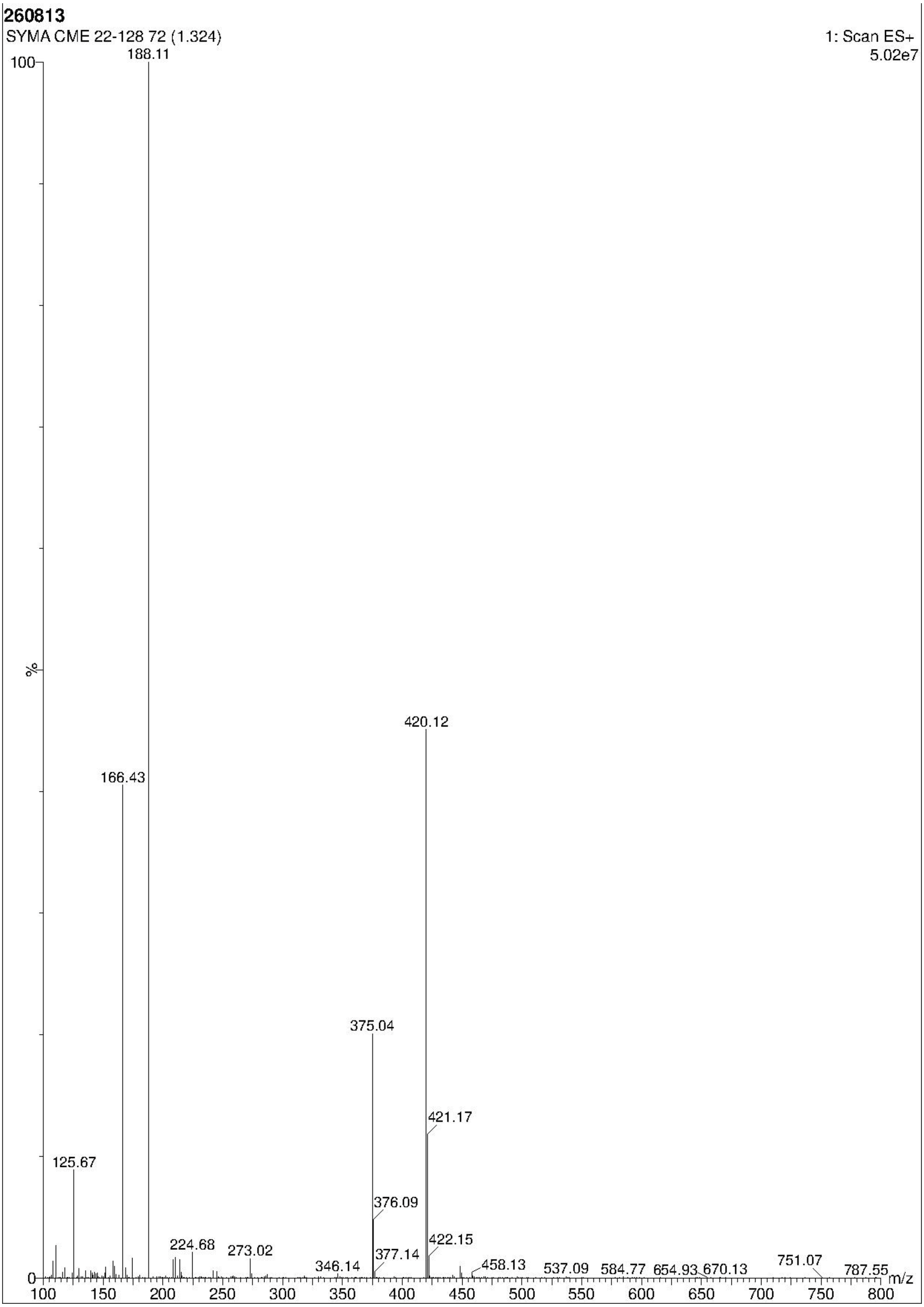
260813
SYMA CME 22-128 72 (1.324)
1: Scan ES+
5.02e7
100
%
0
188.11
166.43
420.12
375.04
421.17
125.67
376.09
422.15
224.68
273.02
377.14
346.14
458.13
537.09
584.77
654.93
670.13
751.07
787.55
m/z
100
150
200
250
300
350
400
450
500
550
600
650
700
750
800

HPLC C4-column

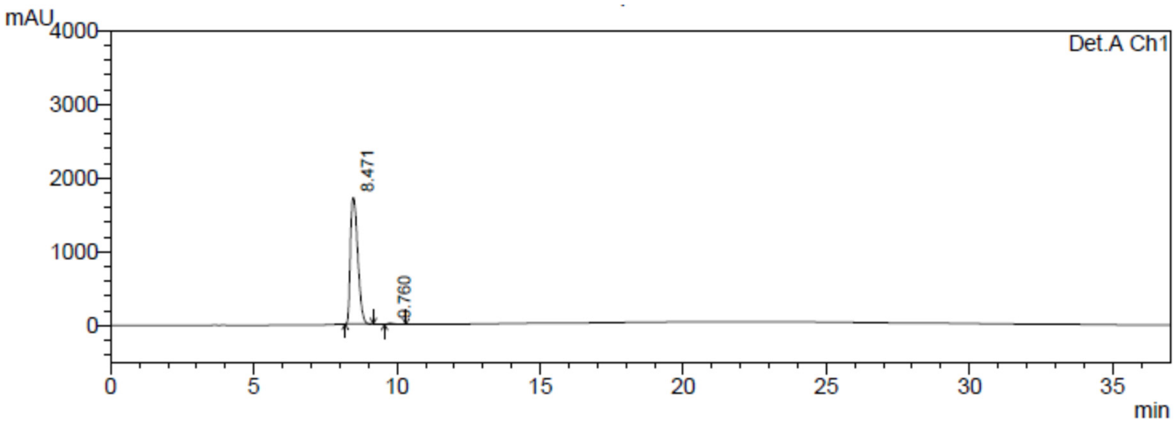

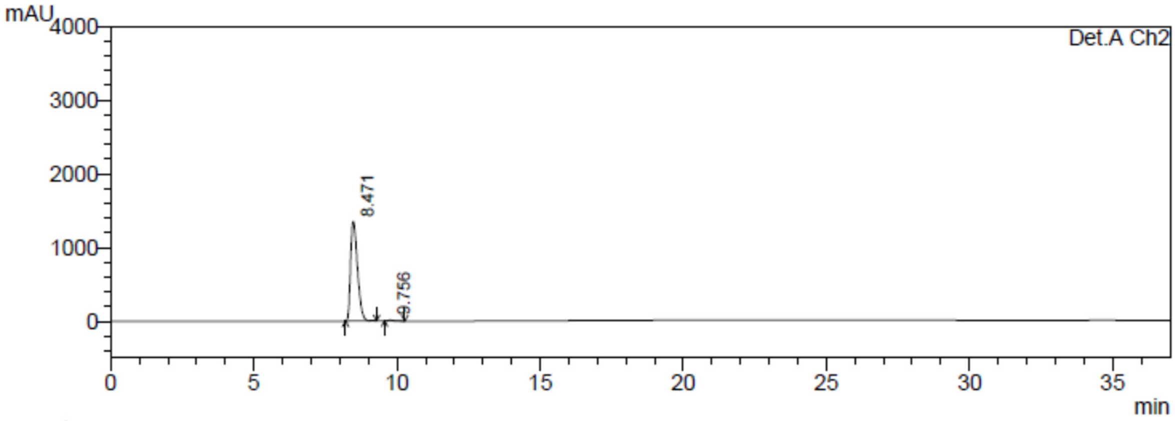

1 Det.A Ch1/215nm
2 Det.A Ch2/254nm

PeakTable

Detector A Ch1 215nm

Peak#	Ret. Time	Area	Area %
1	8.471	30516920	99.158
2	9.760	259155	0.842
Total		30776075	100.000

PeakTable

Detector A Ch2 254nm

Peak#	Ret. Time	Area	Area %
1	8.471	22579462	99.327
2	9.756	153095	0.673
Total		22732557	100.000

HPLC C18-column

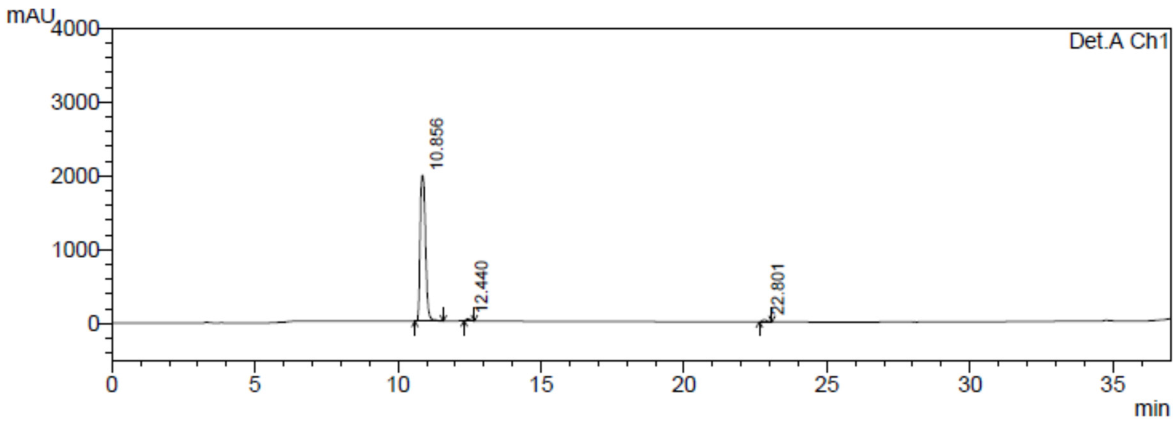

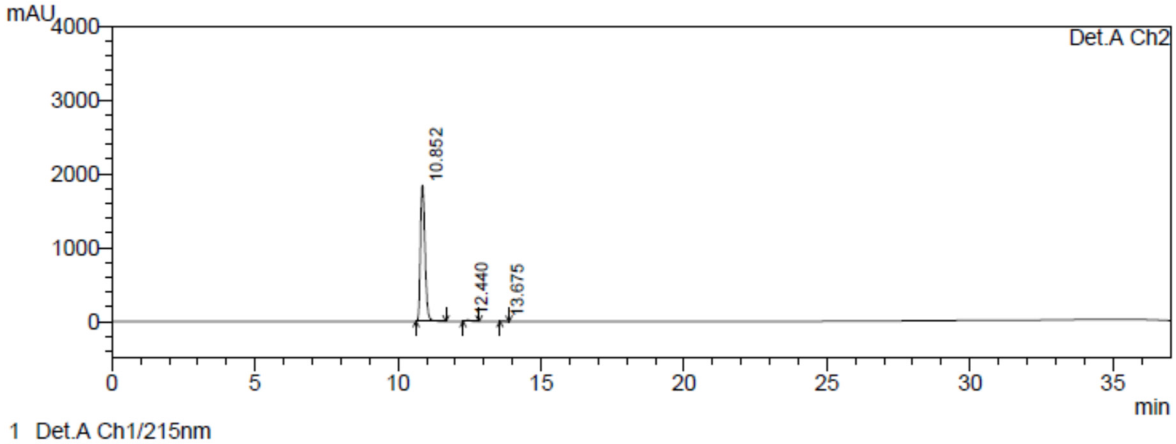

1 Det.A Ch1/215nm
2 Det.A Ch2/254nm

PeakTable

Detector A Ch1 215nm

Peak#	Ret. Time	Area	Area %
1	10.856	25810360	97.973
2	12.440	244348	0.928
3	22.801	289763	1.100
Total		26344471	100.000

PeakTable

Detector A Ch2 254nm

Peak#	Ret. Time	Area	Area %
1	10.852	20633859	98.820
2	12.440	180925	0.866
3	13.675	65413	0.313
Total		20880198	100.000

3-[4-[1-[4-(dimethylaminomethyl)phenyl]-4-[3-(dimethylamino)propyl]pyrazol-3-yl]phenyl]-*N*,*N*-dimethyl-propan-1-amine (**25**)

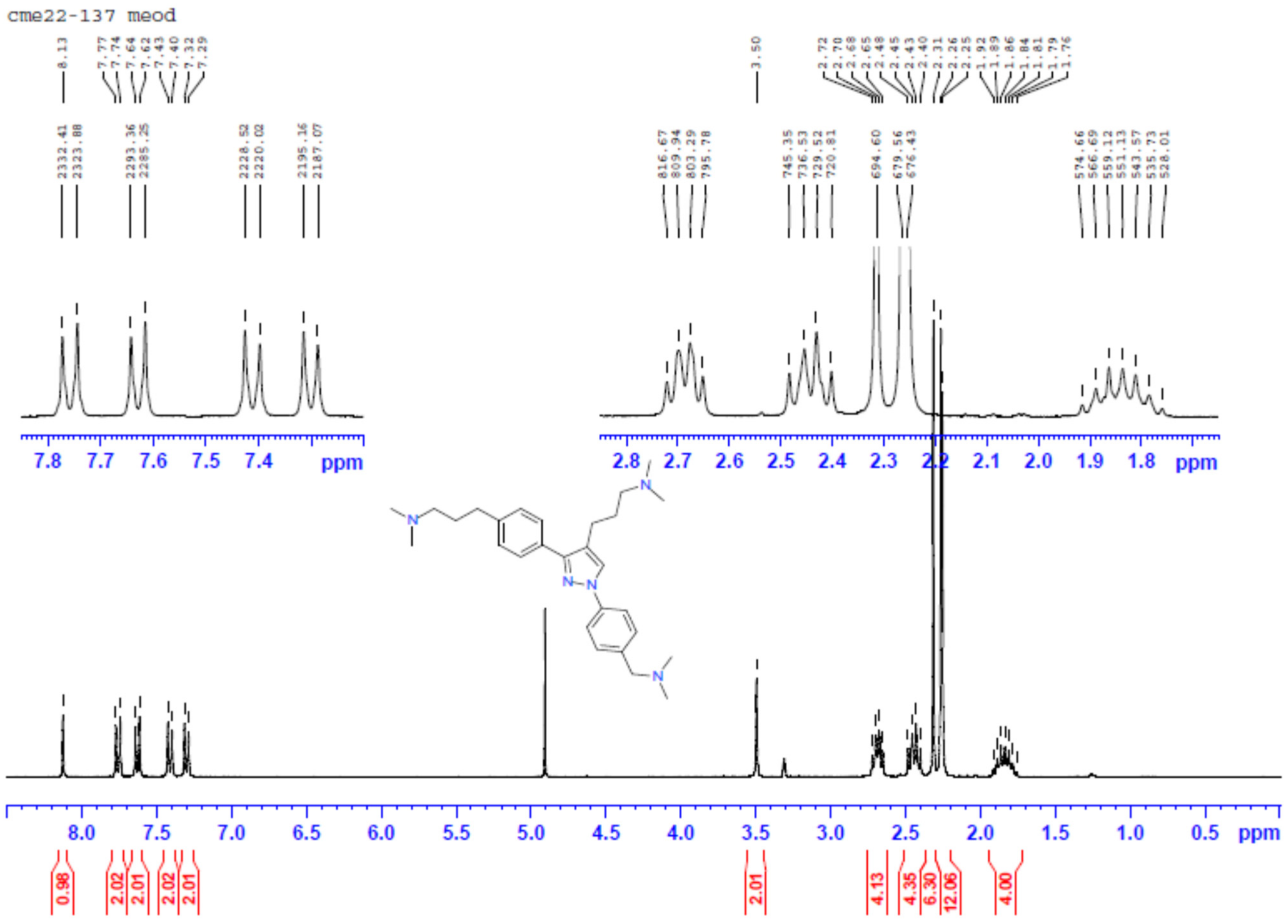

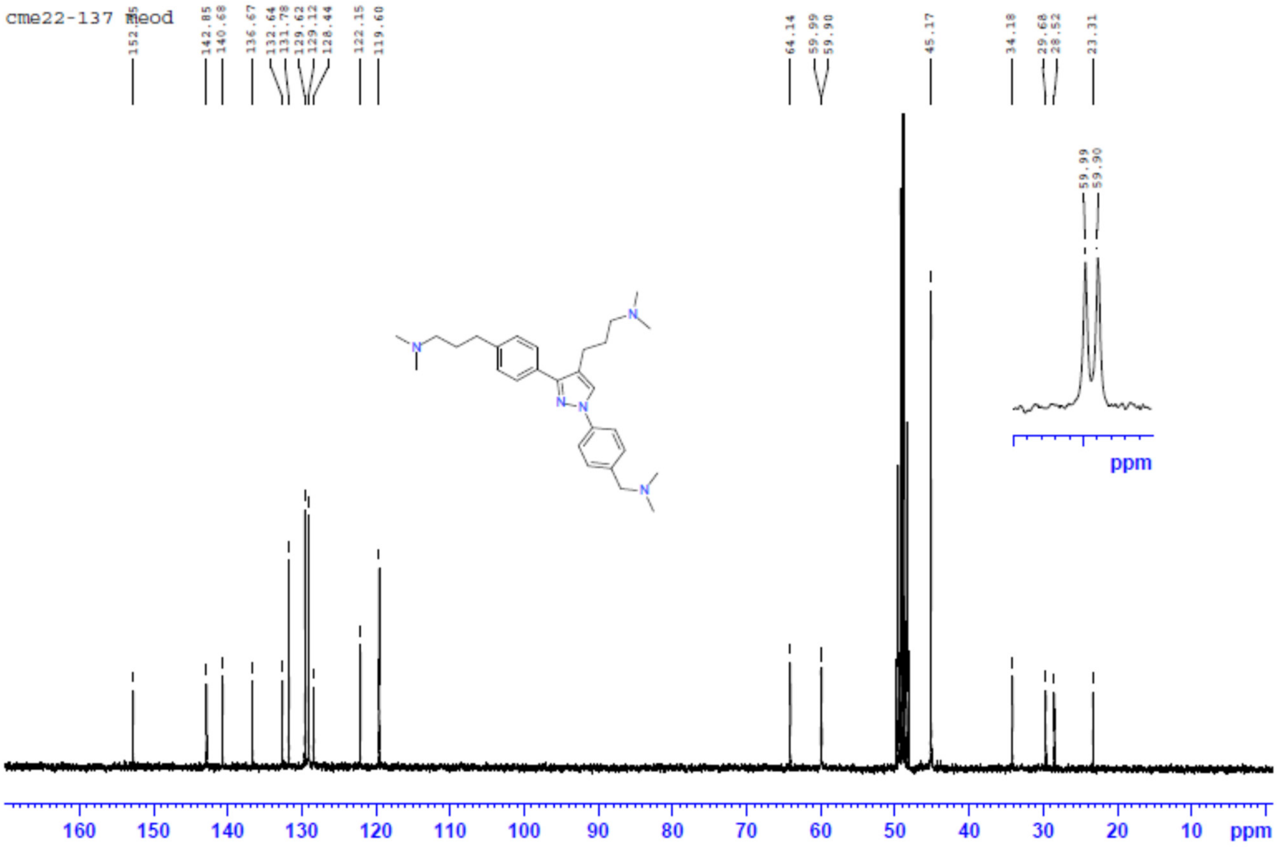

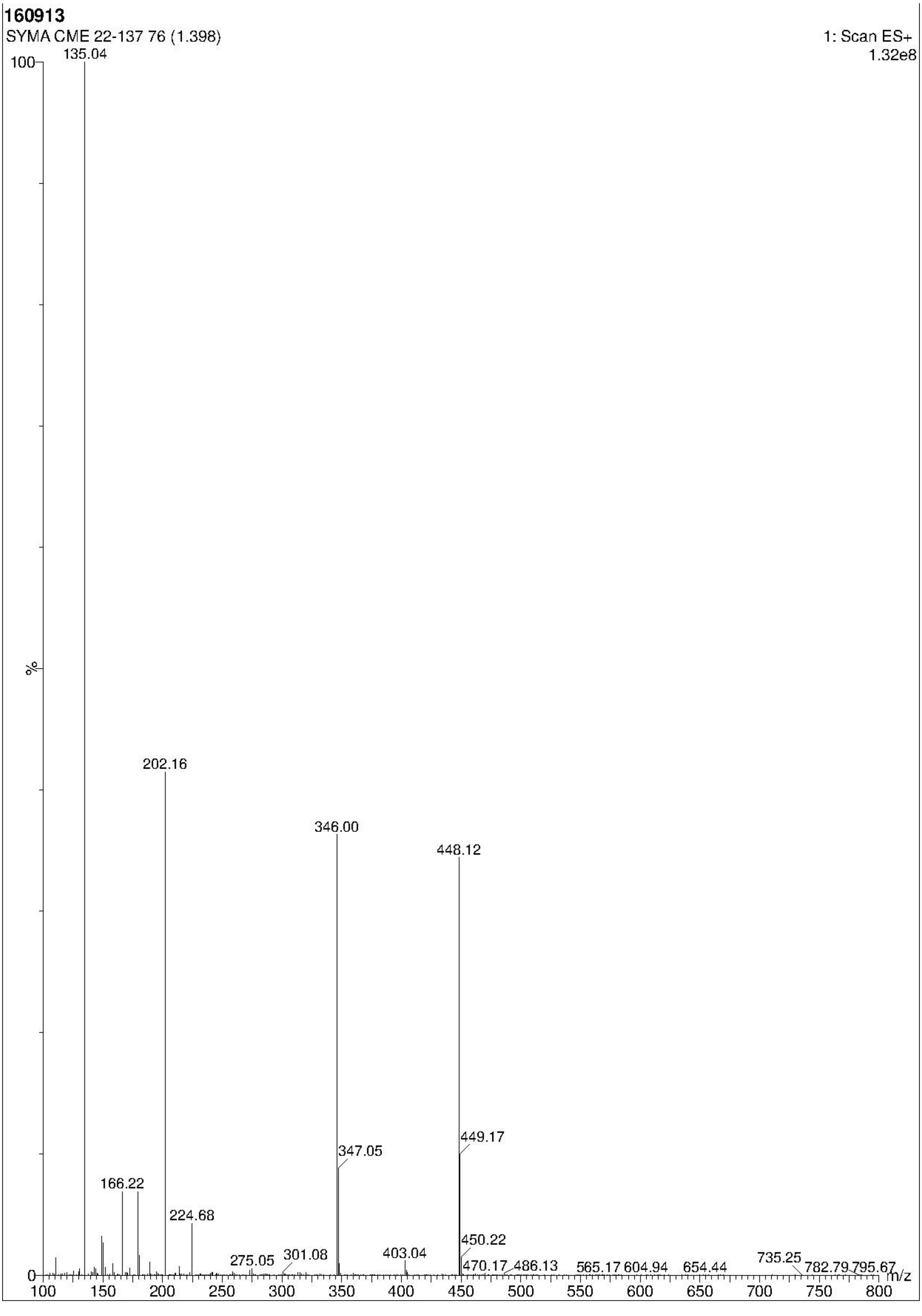
160913
SYMA CME 22-137 76 (1.398)
1: Scan ES+
1.32e8
100
%
0
135.04
202.16
346.00
448.12
449.17
347.05
166.22
224.68
450.22
275.05
301.08
403.04
470.17
486.13
565.17
604.94
654.44
735.25
782.79
795.67
m/z
100
150
200
250
300
350
400
450
500
550
600
650
700
750
800

HPLC C4-column

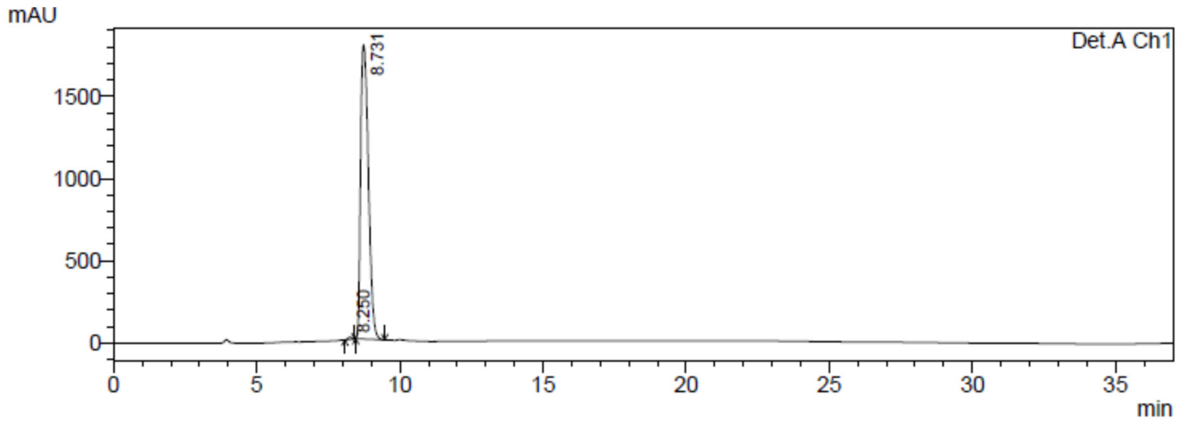

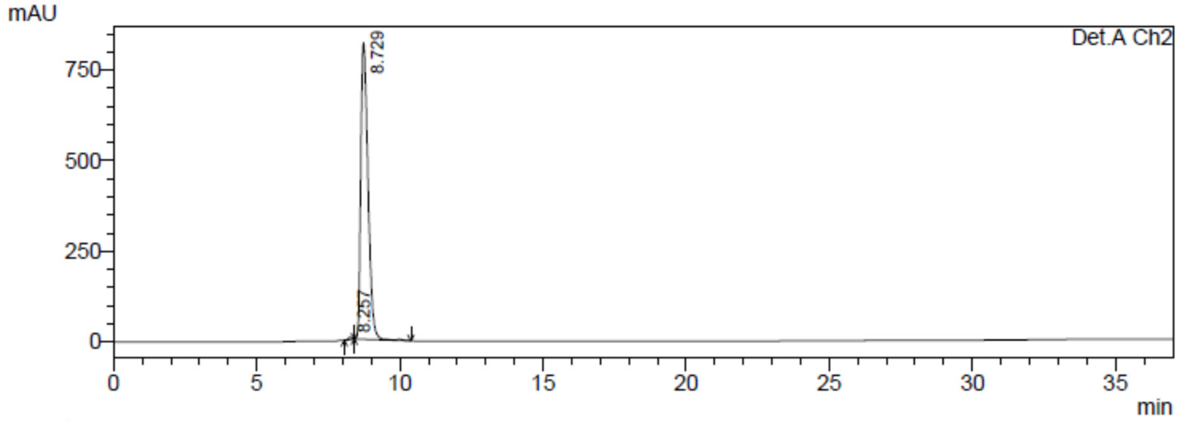

1 Det.A Ch1/215nm
2 Det.A Ch2/254nm

PeakTable

Detector A Ch1 215nm

Peak#	Ret. Time	Area	Area %
1	8.250	127070	0.375
2	8.731	33789184	99.625
Total		33916255	100.000

PeakTable

Detector A Ch2 254nm

Peak#	Ret. Time	Area	Area %
1	8.257	56652	0.394
2	8.729	14308304	99.606
Total		14364957	100.000

HPLC C18-column

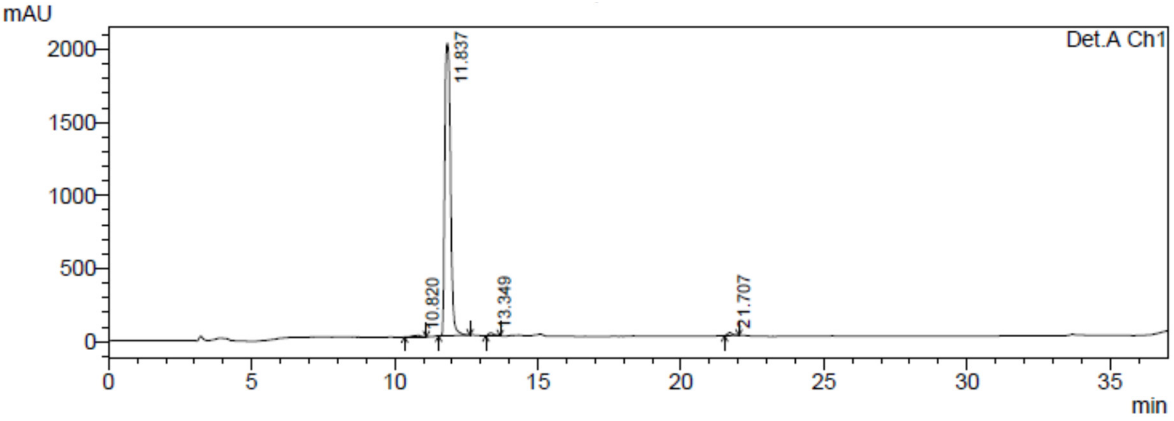

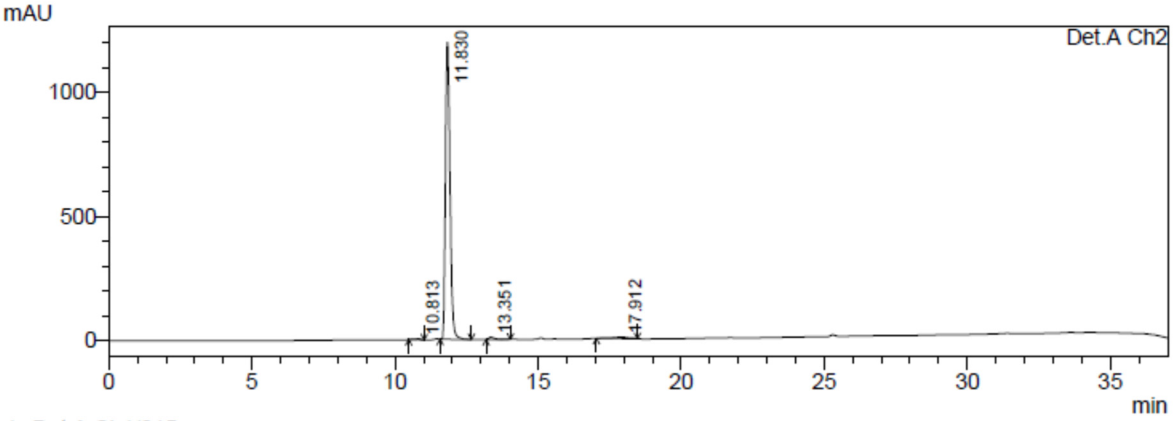

1 Det.A Ch1/215nm
2 Det.A Ch2/254nm

PeakTable

Detector A Ch1 215nm

Peak#	Ret. Time	Area	Area %
1	10.820	188175	0.665
2	11.837	27611585	97.609
3	13.349	260915	0.922
4	21.707	227416	0.804
Total		28288091	100.000

PeakTable

Detector A Ch2 254nm

Peak#	Ret. Time	Area	Area %
1	10.813	75343	0.552
2	11.830	13232821	96.870
3	13.351	107092	0.784
4	17.912	245107	1.794
Total		13660363	100.000

3-[4-[4-(dimethylaminomethyl)-1-[3-(dimethylaminomethyl)phenyl]pyrazol-3-yl]phenyl]-*N*,*N*-dimethyl-propan-1-amine (**26**)

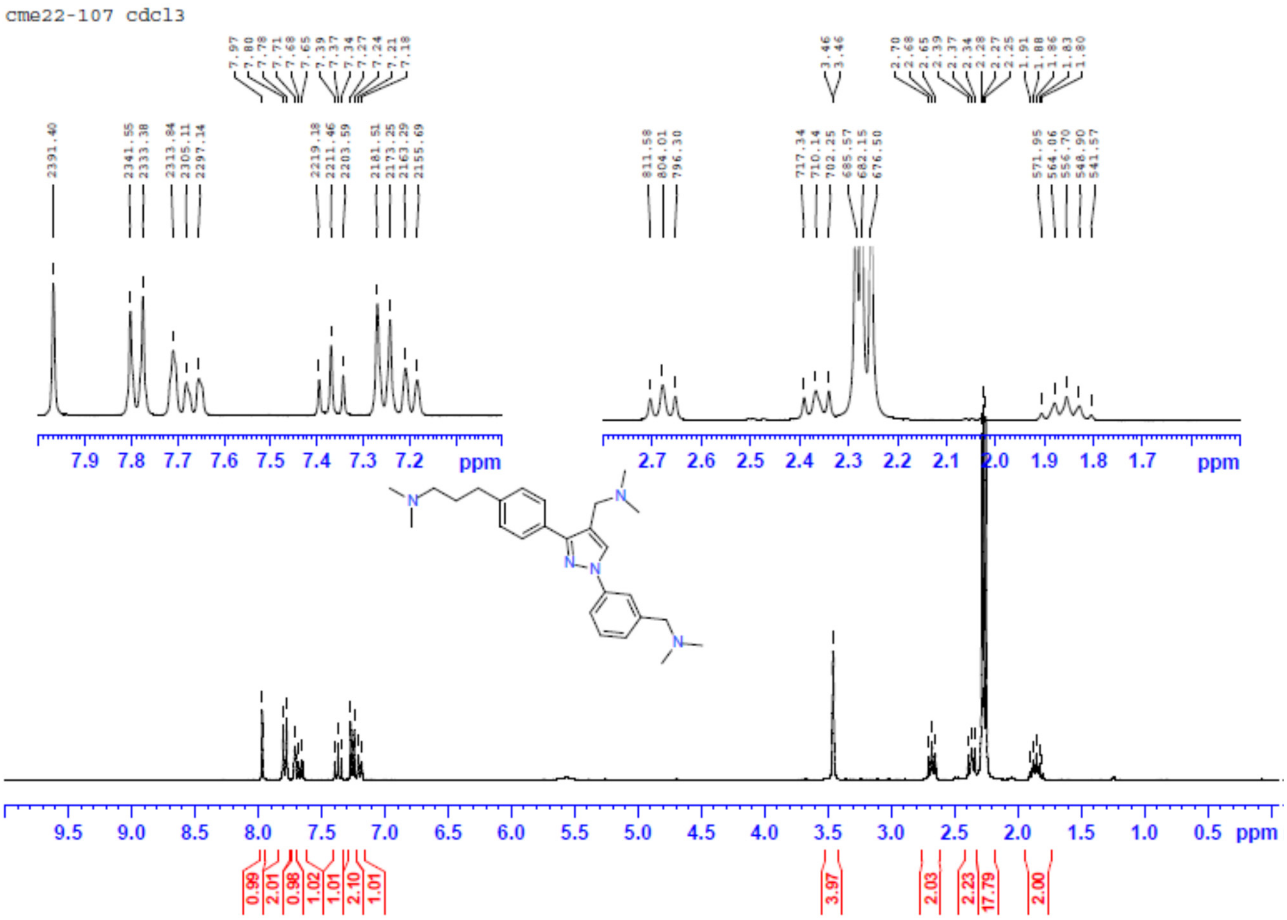

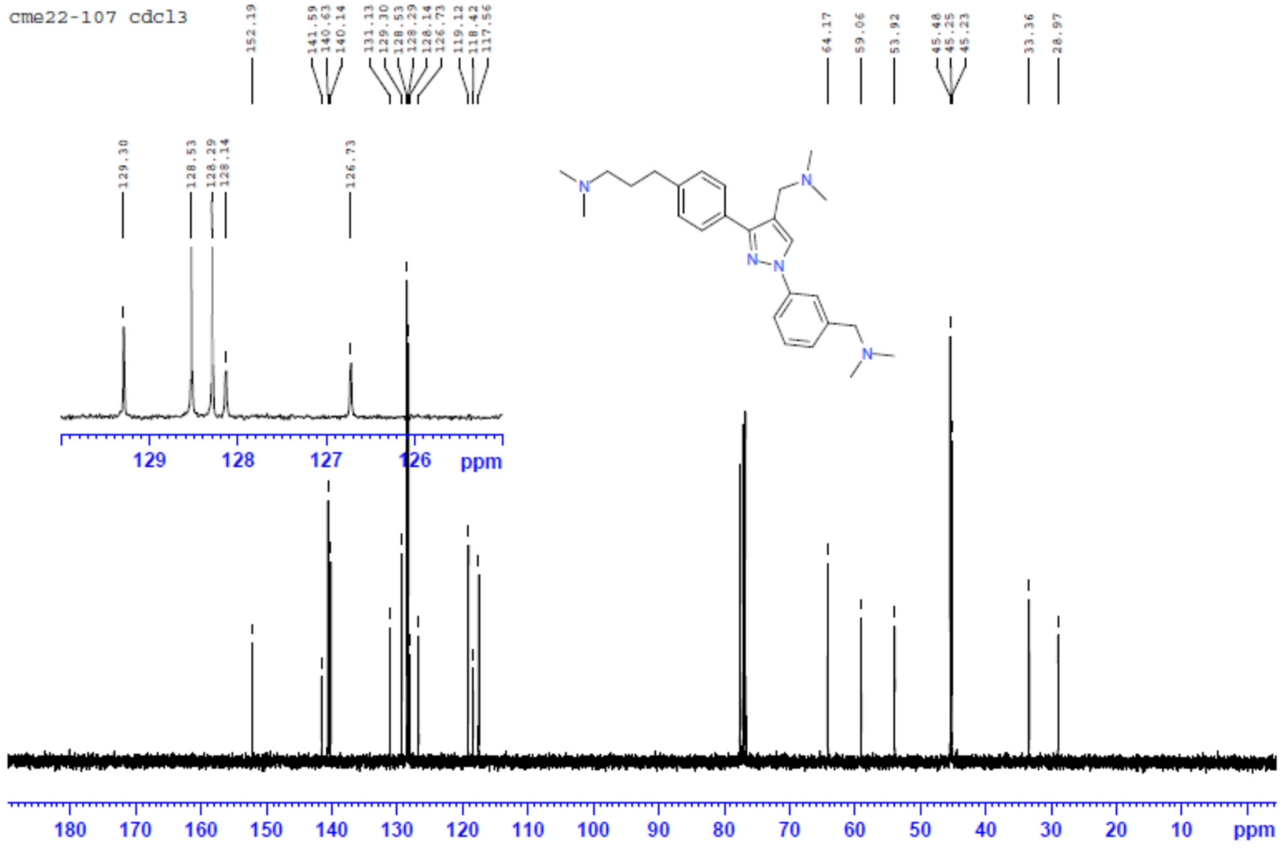

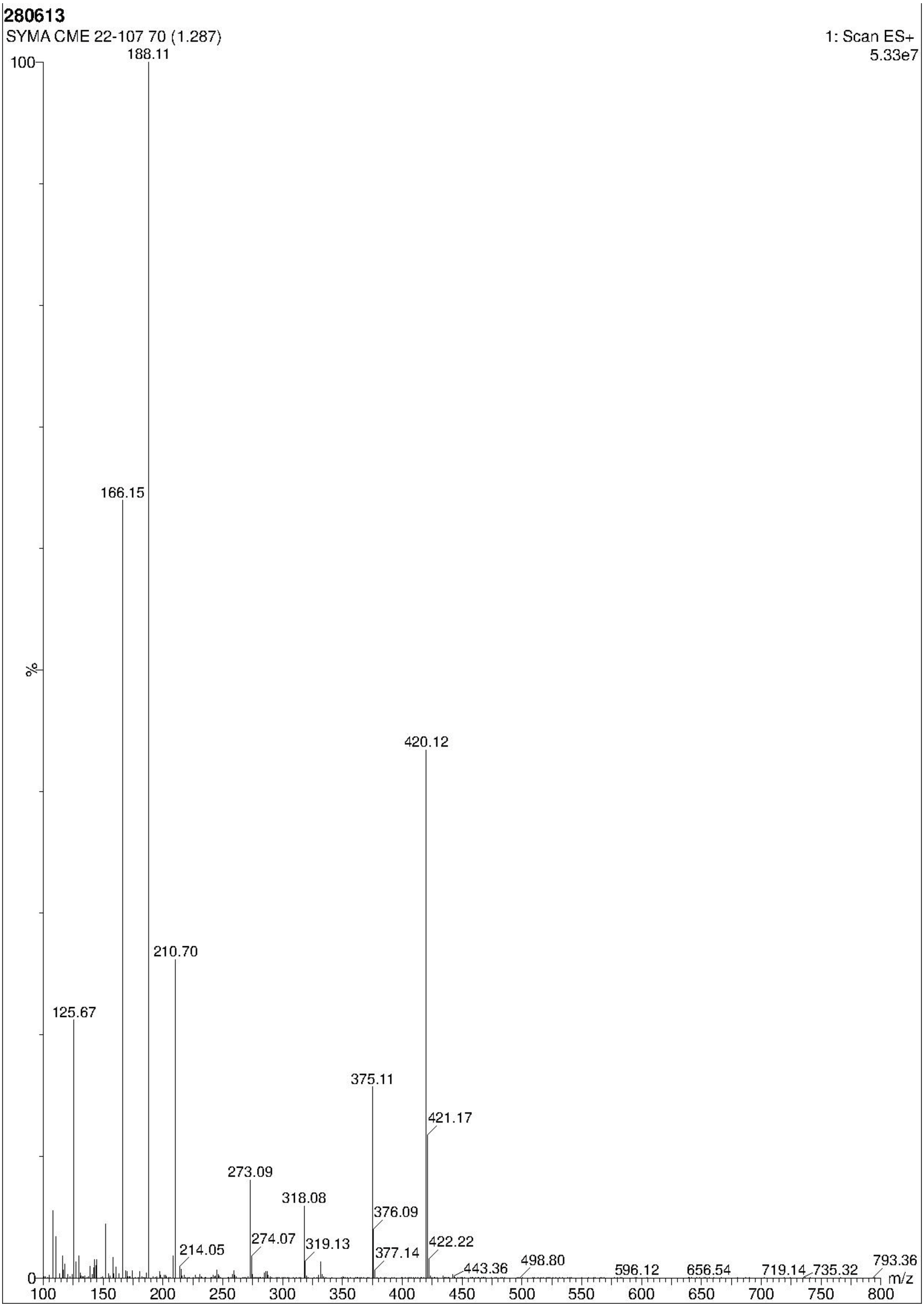
280613
SYMA CME 22-107 70 (1.287)
1: Scan ES+
5.33e7
100
%
0
188.11
166.15
420.12
210.70
125.67
375.11
421.17
273.09
318.08
376.09
274.07
319.13
422.22
214.05
377.14
443.36
498.80
596.12
656.54
719.14
735.32
793.36
m/z
100
150
200
250
300
350
400
450
500
550
600
650
700
750
800

HPLC C4-column

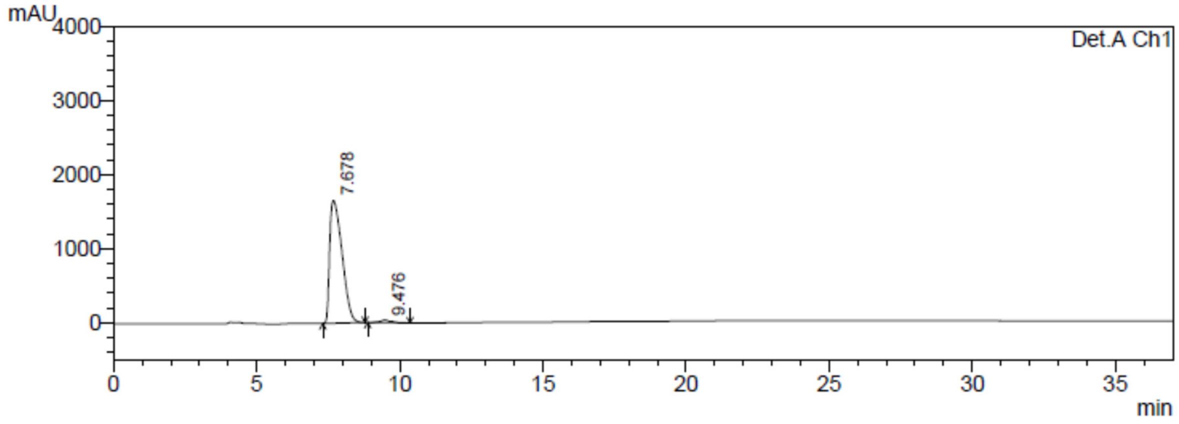

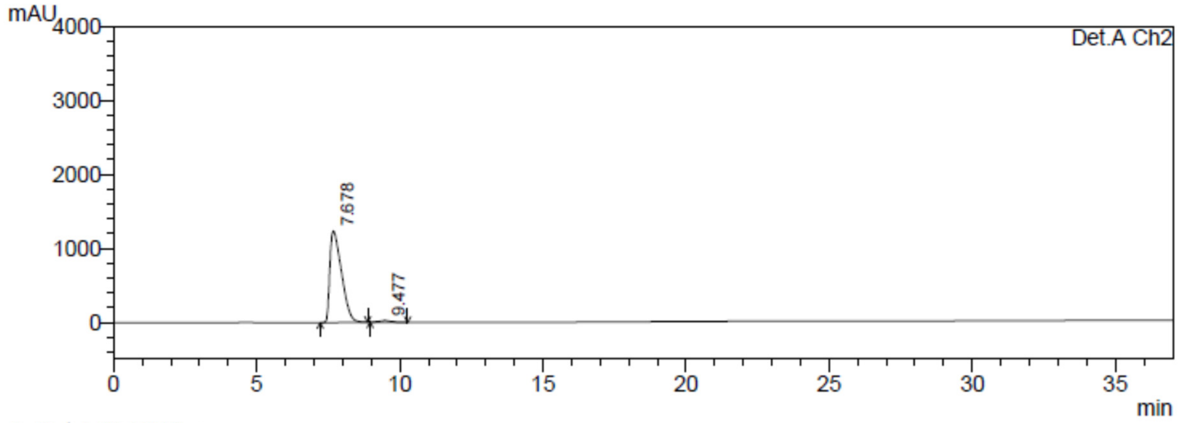

1 Det.A Ch1/215nm
2 Det.A Ch2/254nm

PeakTable

Detector A Ch1 215nm

Peak#	Ret. Time	Area	Area %
1	7.678	49798982	98.181
2	9.476	922444	1.819
Total		50721426	100.000

PeakTable

Detector A Ch2 254nm

Peak#	Ret. Time	Area	Area %
1	7.678	34831175	98.393
2	9.477	568993	1.607
Total		35400168	100.000

HPLC C18-column

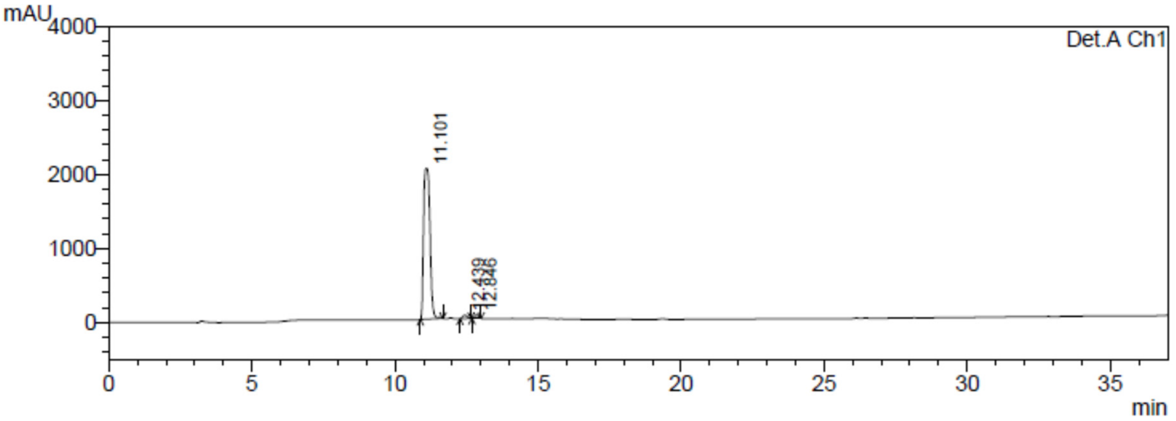

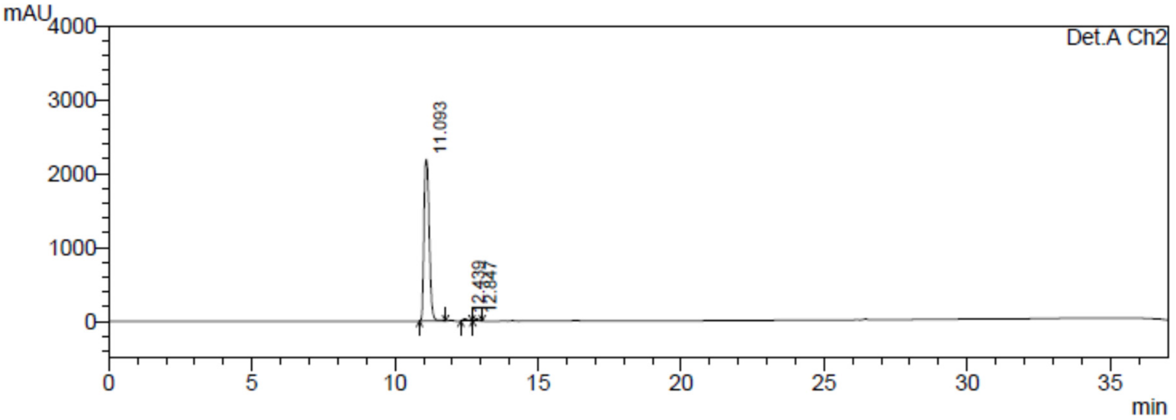

1 Det.A Ch1/215nm
2 Det.A Ch2/254nm

PeakTable

Detector A Ch1 215nm

Peak#	Ret. Time	Area	Area %
1	11.101	31376803	97.148
2	12.439	506876	1.569
3	12.846	414337	1.283
Total		32298016	100.000

PeakTable

Detector A Ch2 254nm

Peak#	Ret. Time	Area	Area %
1	11.093	27872976	98.074
2	12.439	280735	0.988
3	12.847	266706	0.938
Total		28420417	100.000

3-[4-[1-[3-(dimethylaminomethyl)phenyl]-4-[3-(dimethylamino)propyl]pyrazol-3-yl]phenyl]-*N*,*N*-dimethyl-propan-1-amine (**27**)

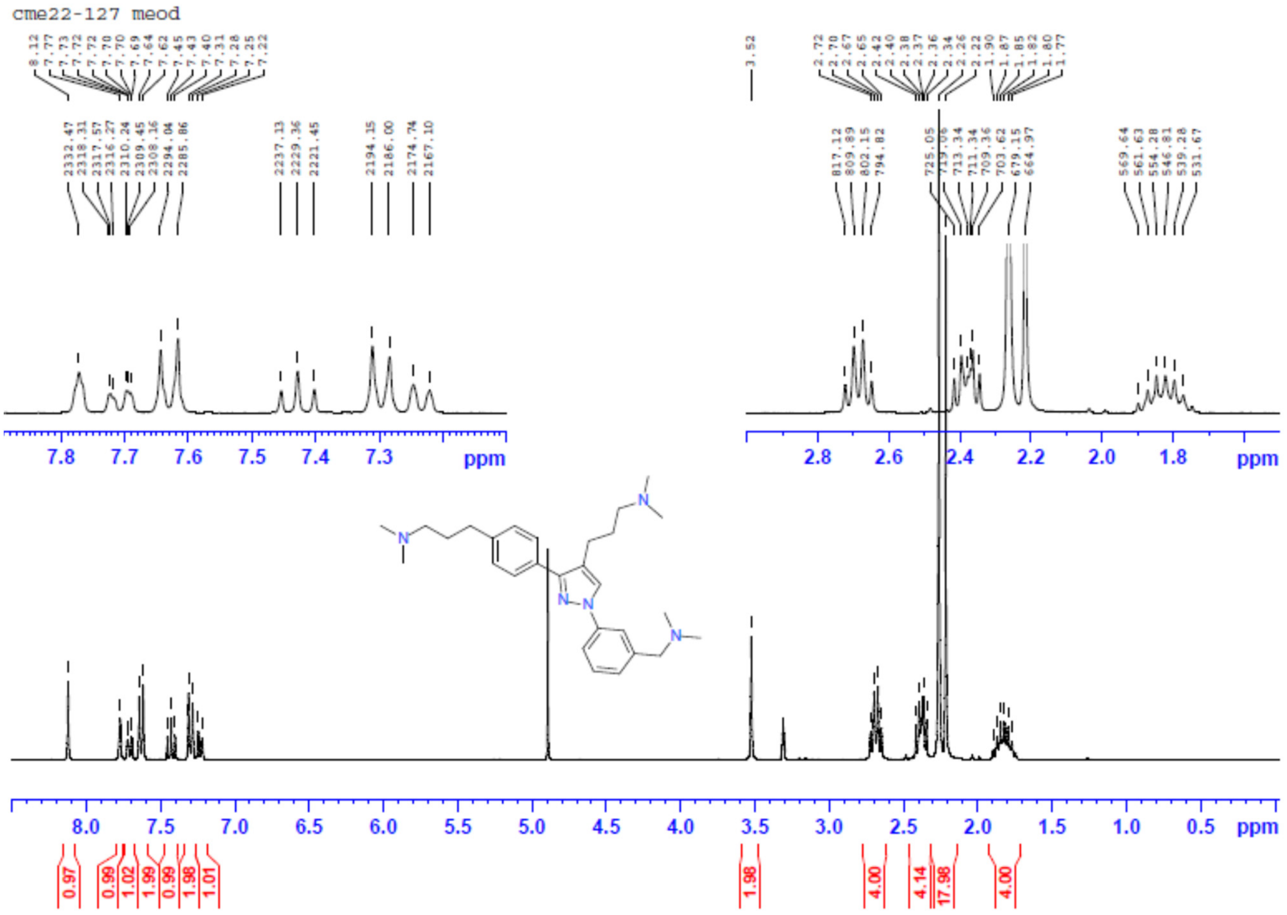

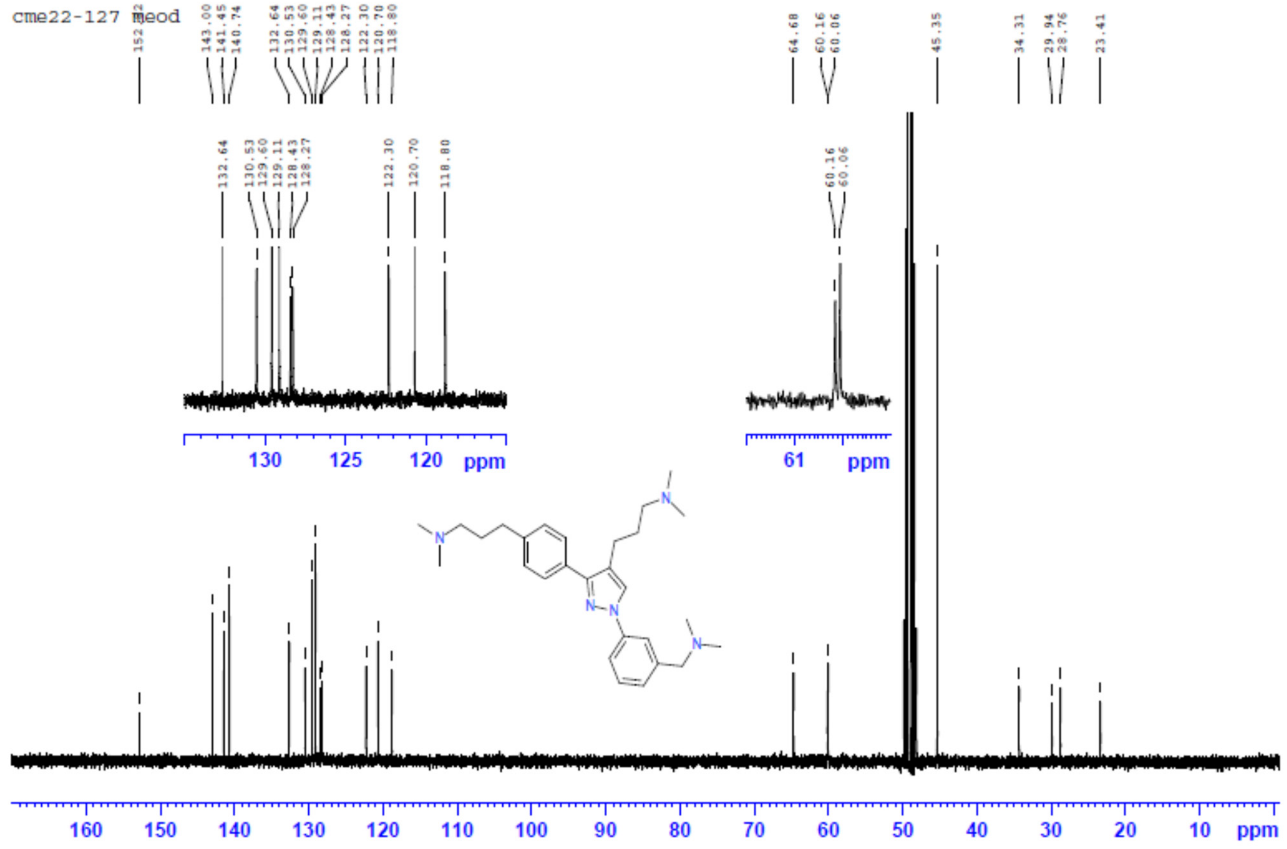

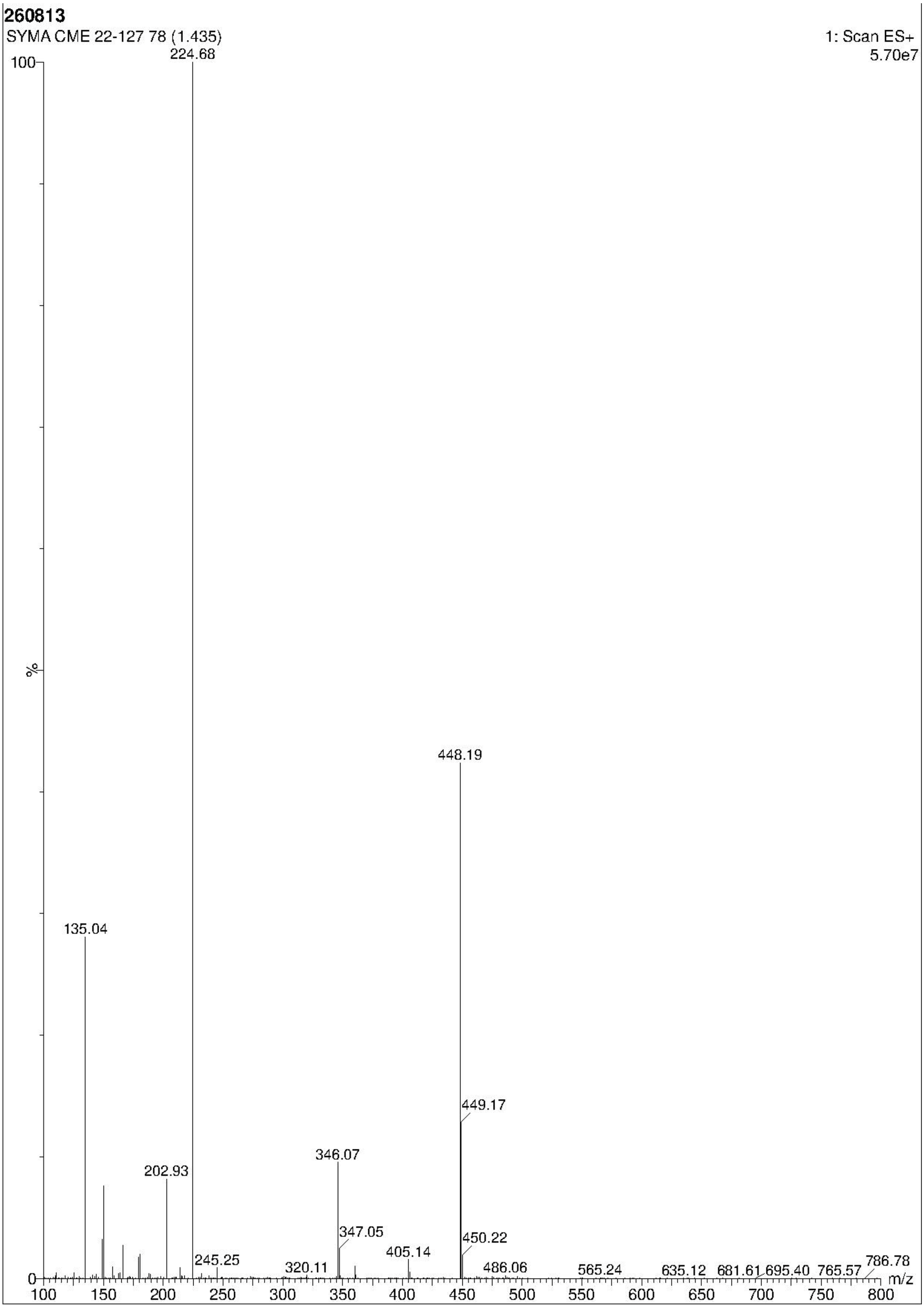
260813
SYMA CME 22-127 78 (1.435)
1: Scan ES+
5.70e7
100
%
0
224.68
448.19
135.04
449.17
346.07
202.93
347.05
450.22
405.14
245.25
320.11
486.06
565.24
635.12
681.61
695.40
765.57
786.78
100
150
200
250
300
350
400
450
500
550
600
650
700
750
800
m/z

HPLC C4-column

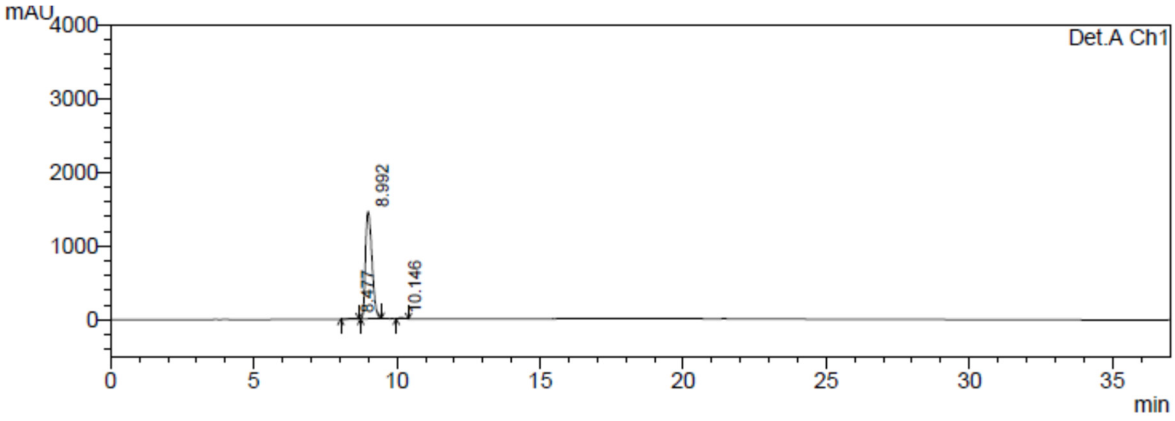

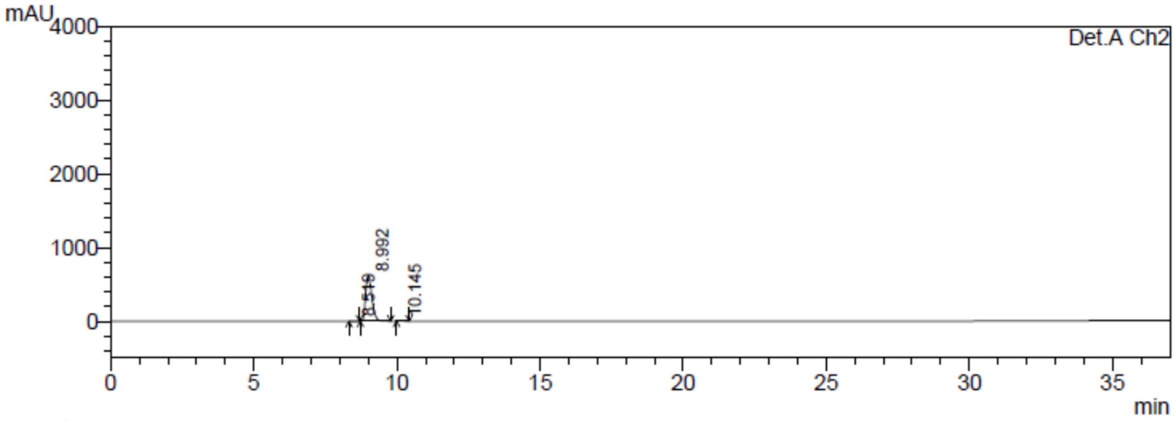

1 Det.A Ch1/215nm
2 Det.A Ch2/254nm

PeakTable
Detector A Ch1 215nm

Peak#	Ret. Time	Area	Area %
1	8.477	187370	0.815
2	8.992	22625159	98.399
3	10.146	180764	0.786
Total		22993293	100.000

PeakTable
Detector A Ch2 254nm

Peak#	Ret. Time	Area	Area %
1	8.519	32009	0.341
2	8.992	9293694	98.946
3	10.145	66982	0.713
Total		9392685	100.000

HPLC C18-column

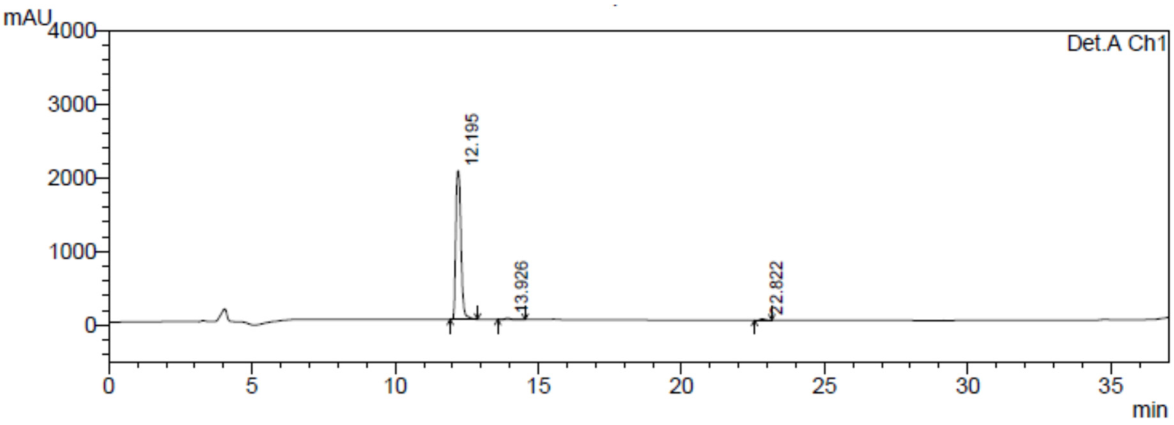

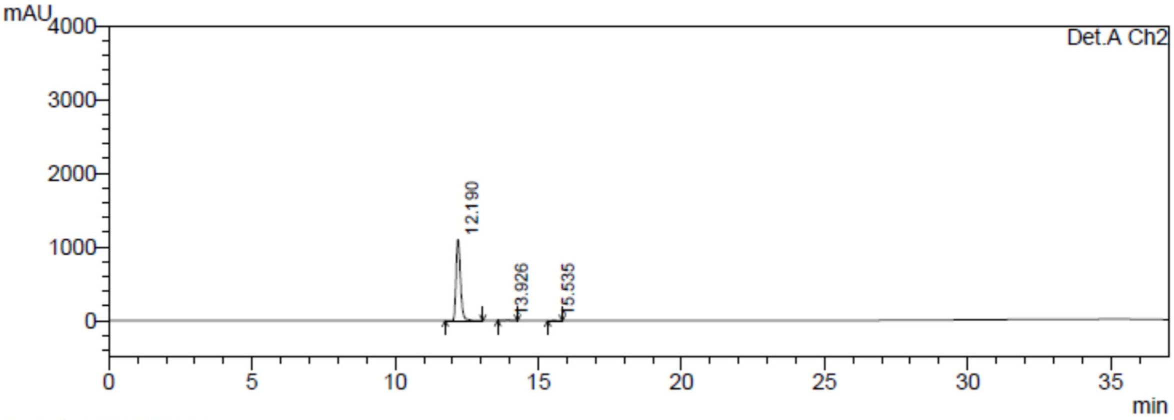

1 Det.A Ch1/215nm
2 Det.A Ch2/254nm

PeakTable

Detector A Ch1 215nm

Peak#	Ret. Time	Area	Area %
1	12.195	25522697	98.474
2	13.926	176038	0.679
3	22.822	219426	0.847
Total		25918161	100.000

PeakTable

Detector A Ch2 254nm

Peak#	Ret. Time	Area	Area %
1	12.190	11788328	98.754
2	13.926	72206	0.605
3	15.535	76471	0.641
Total		11937004	100.000

3-[4-[4-(dimethylaminomethyl)-1-[4-[(4-methylpiperazin-1-yl)methyl]phenyl]pyrazol-3-yl]phenyl]-*N*,*N*-dimethyl-propan-1-amine (**28**)

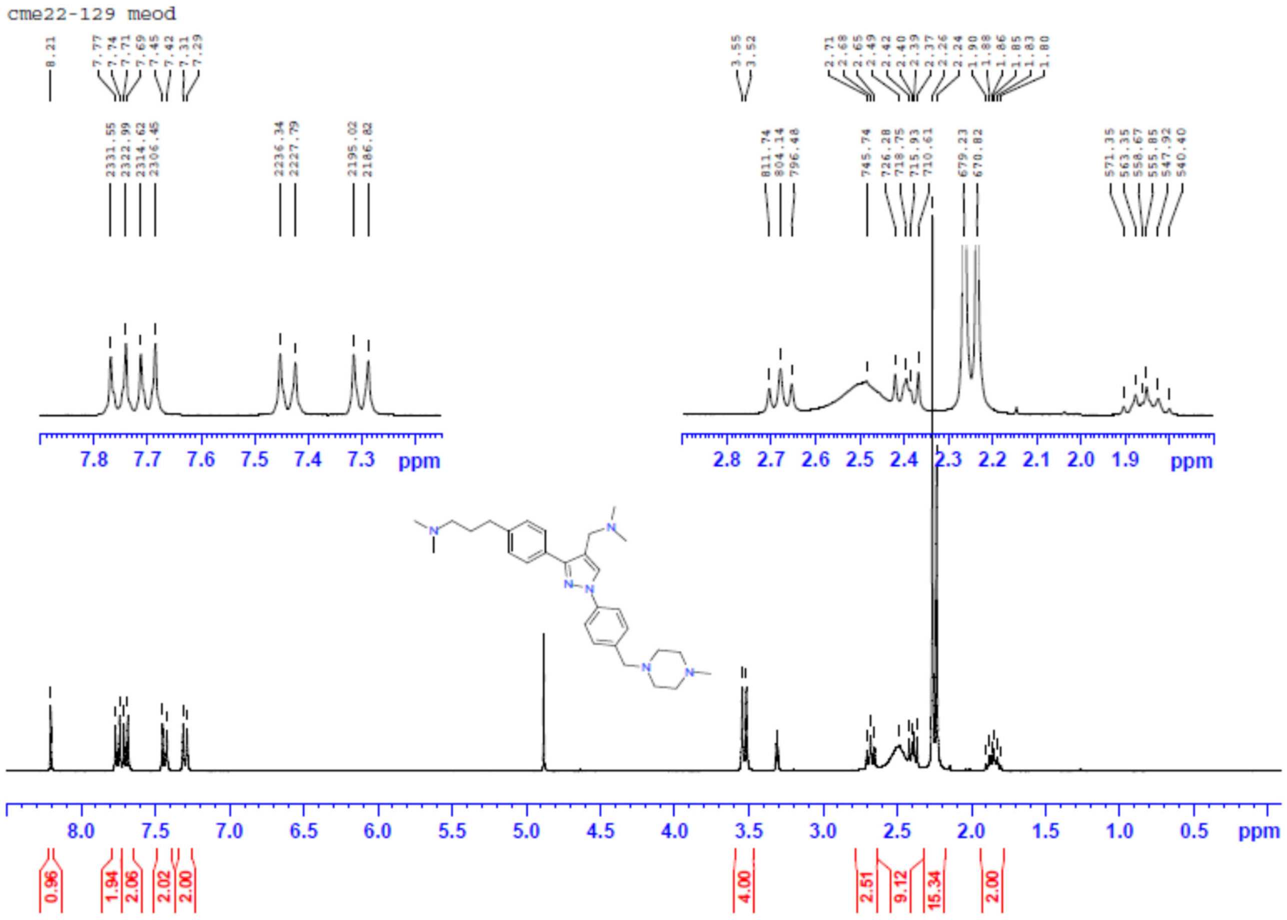

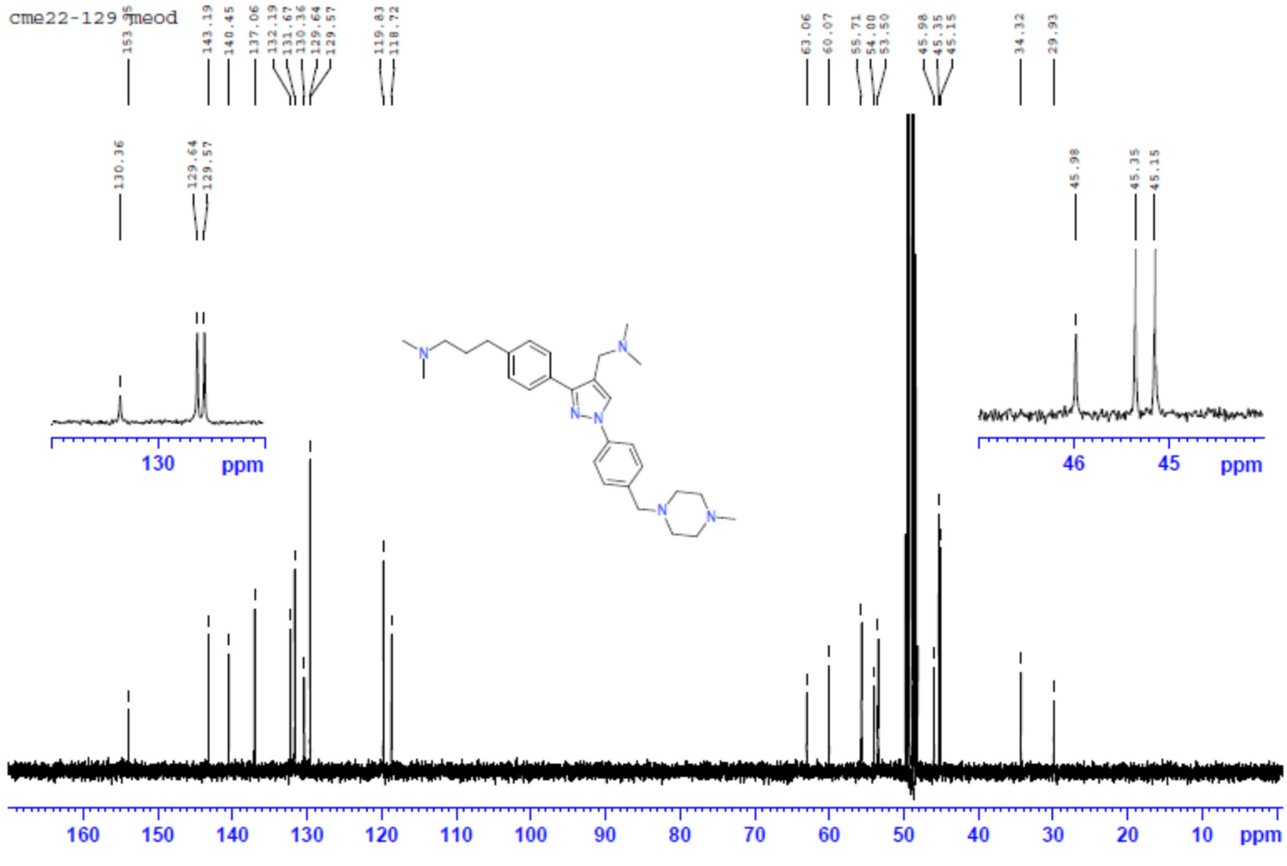

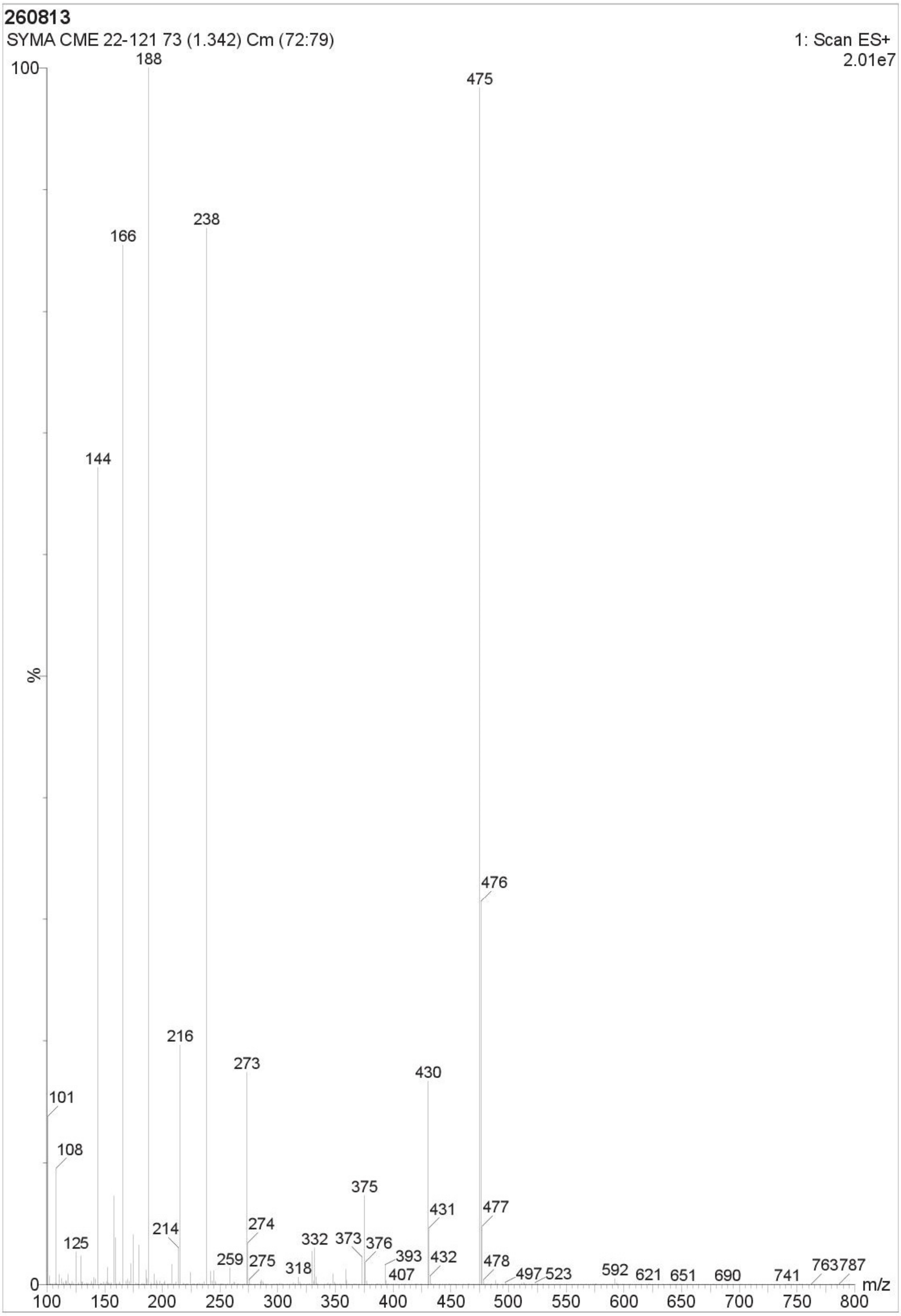
260813
SYMA CME 22-121 73 (1.342) Cm (72:79)
1: Scan ES+
2.01e7
%
m/z
188
475
238
166
144
476
216
273
430
101
108
375
431
477
274
214
125
332
373
376
393
432
478
259
275
318
407
497
523
592
621
651
690
741
763
787

HPLC C4-column

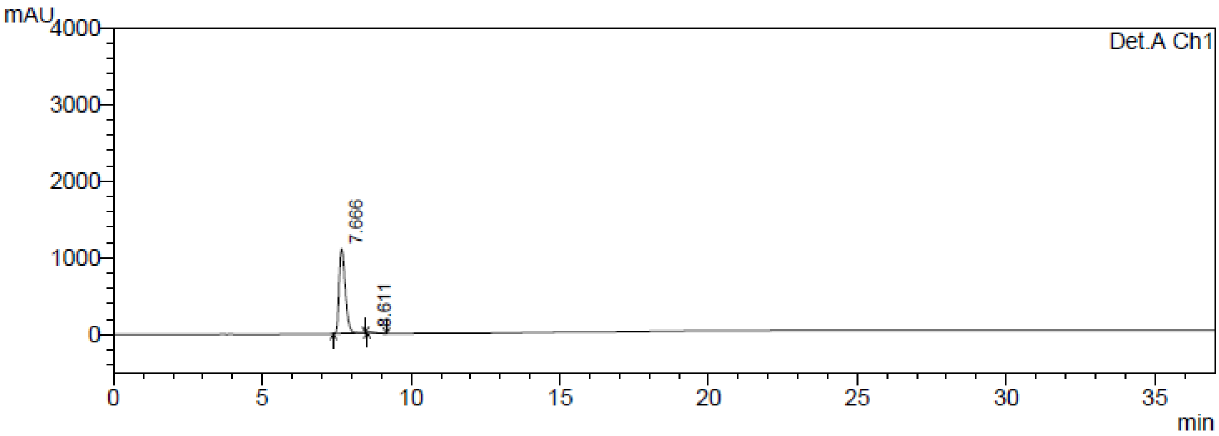

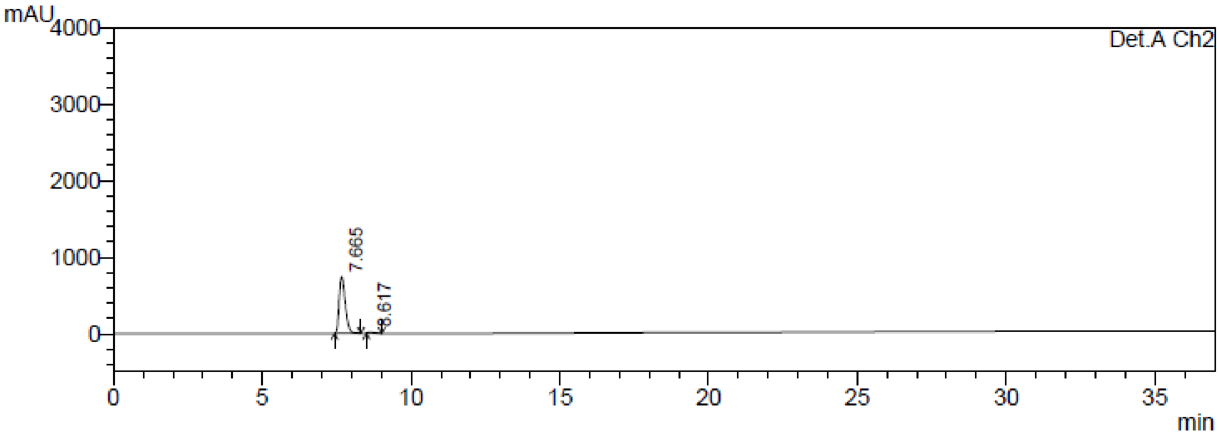

1 Det.A Ch1/215nm
2 Det.A Ch2/254nm

PeakTable

Detector A Ch1 215nm

Peak#	Ret. Time	Area	Area %
1	7.666	15854170	99.251
2	8.611	119667	0.749
Total		15973837	100.000

PeakTable

Detector A Ch2 254nm

Peak#	Ret. Time	Area	Area %
1	7.665	10552345	98.317
2	8.617	180643	1.683
Total		10732988	100.000

HPLC C18-column

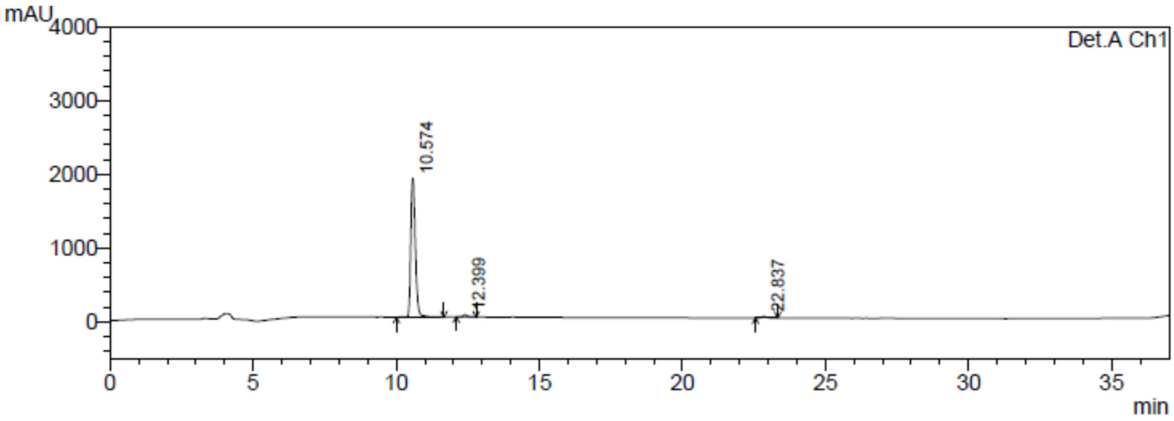

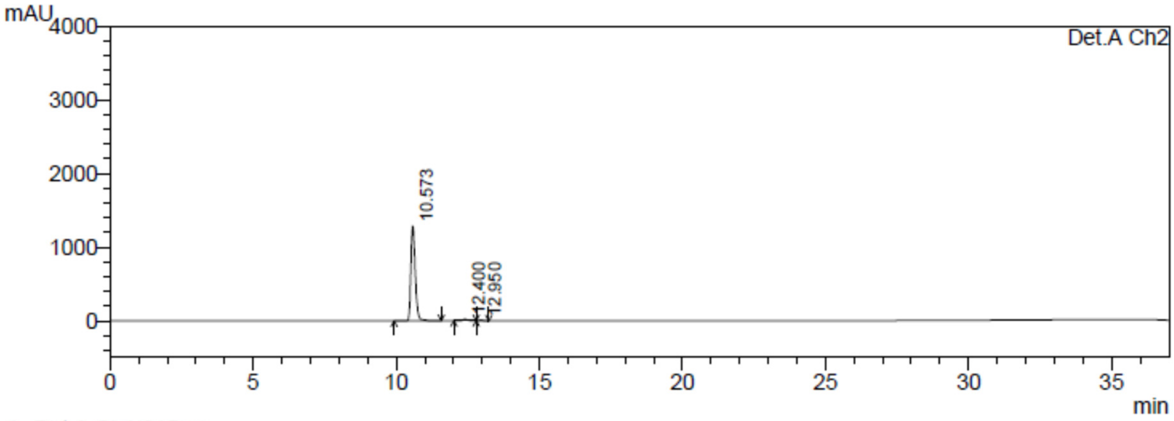

1 Det.A Ch1/215nm
2 Det.A Ch2/254nm

PeakTable

Detector A Ch1 215nm

Peak#	Ret. Time	Area	Area %
1	10.574	20951033	97.485
2	12.399	290256	1.351
3	22.837	250181	1.164
Total		21491470	100.000

PeakTable

Detector A Ch2 254nm

Peak#	Ret. Time	Area	Area %
1	10.573	13759120	97.768
2	12.400	224577	1.596
3	12.950	89584	0.637
Total		14073281	100.000

3-[4-[4-[3-(dimethylamino)propyl]-1-[4-[(4-methylpiperazin-1-yl)methyl]phenyl]pyrazol-3-yl]phenyl]-*N*,*N*-dimethyl-propan-1-amine (**29**)

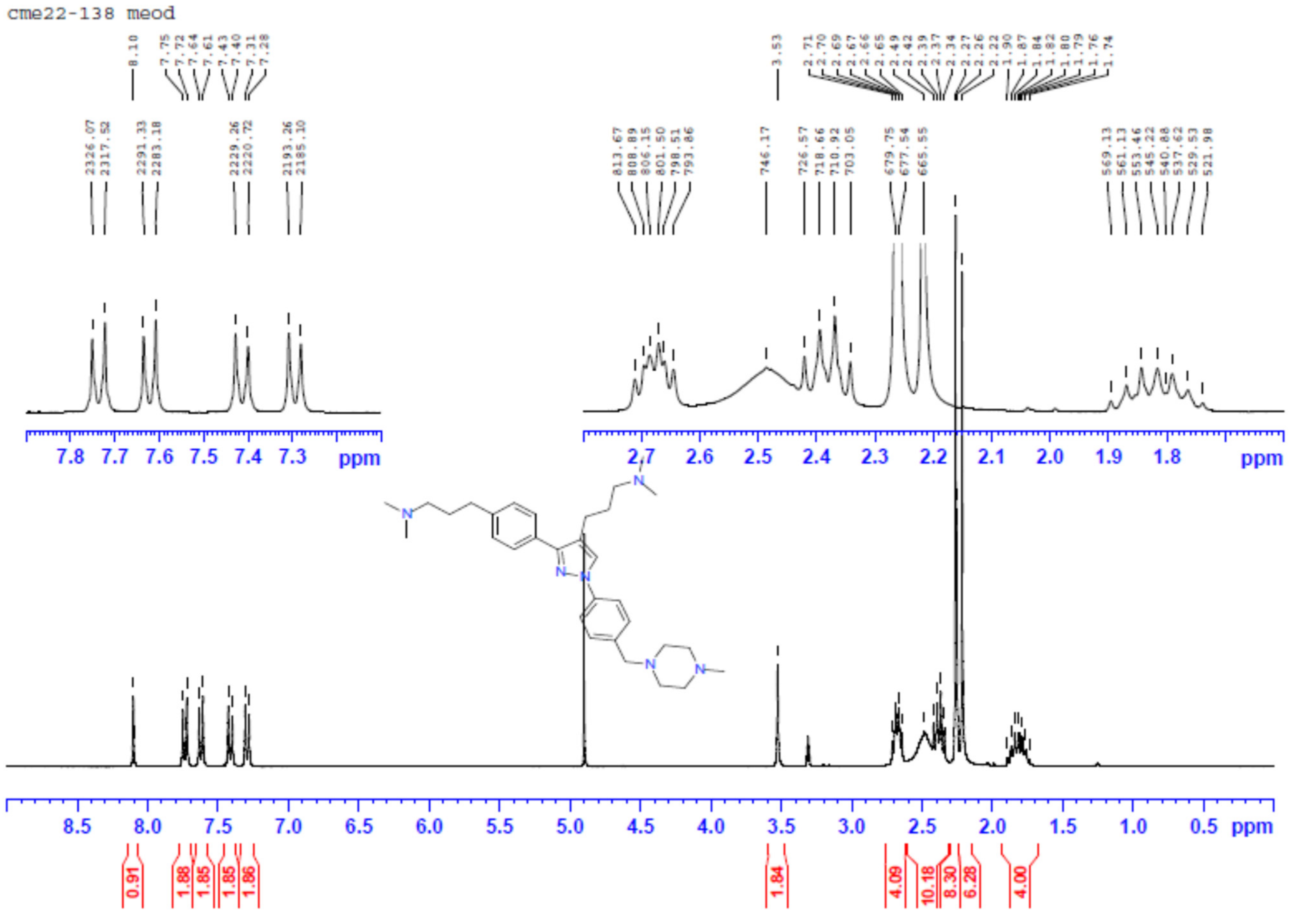

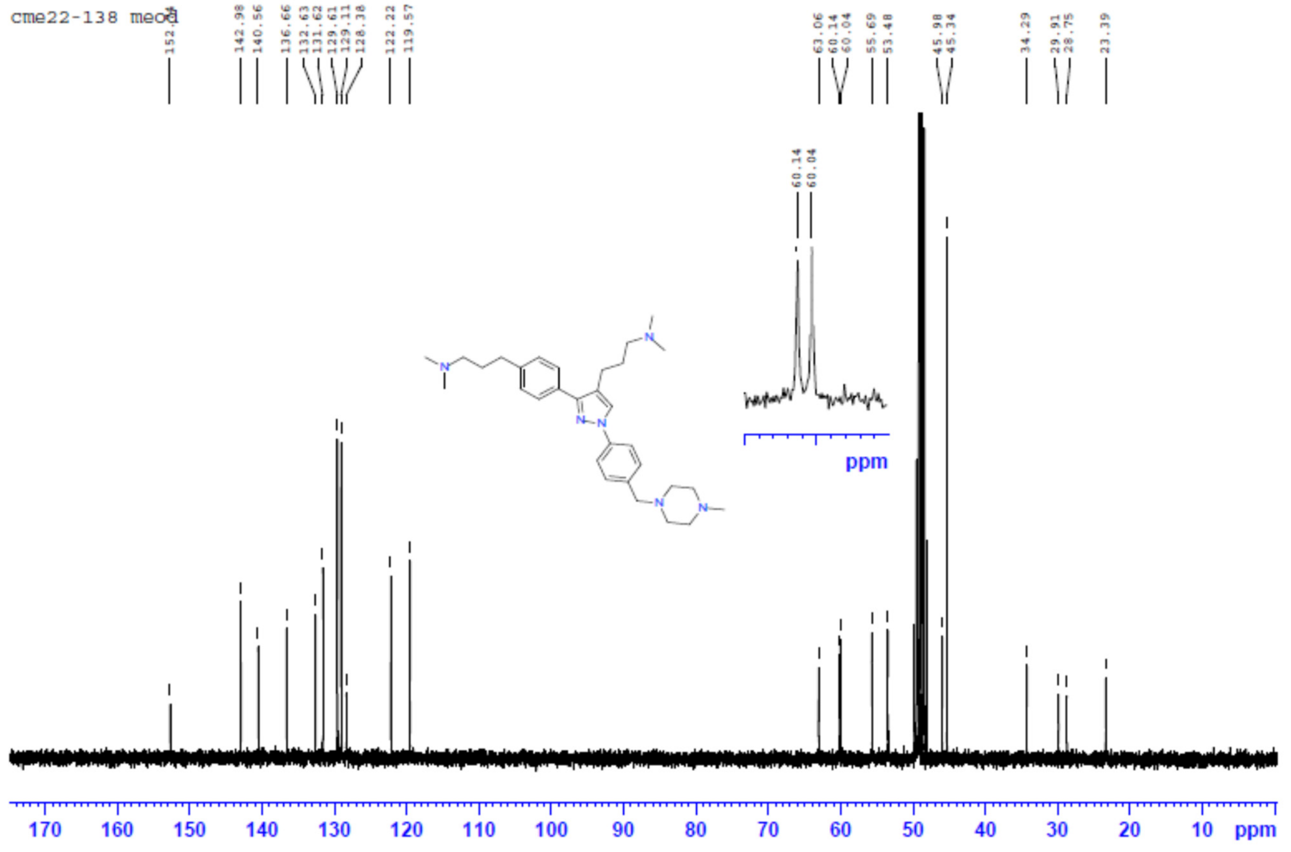

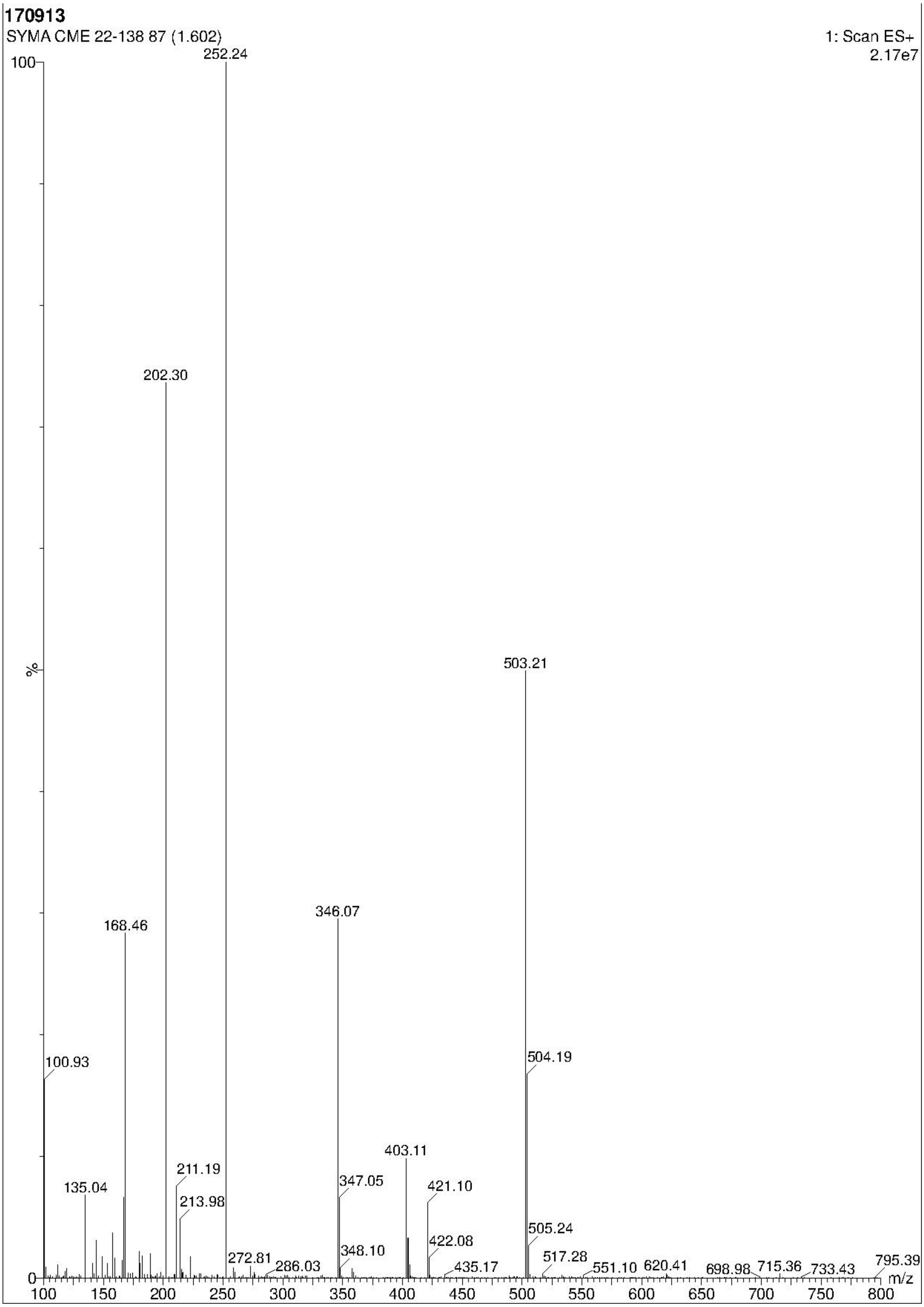
170913
SYMA CME 22-138 87 (1.602)
1: Scan ES+
2.17e7
100
%
0
252.24
202.30
503.21
346.07
168.46
100.93
504.19
403.11
211.19
135.04
347.05
421.10
213.98
505.24
422.08
348.10
272.81
286.03
517.28
435.17
551.10
620.41
698.98
715.36
733.43
795.39
m/z
100
150
200
250
300
350
400
450
500
550
600
650
700
750
800

HPLC C4-column

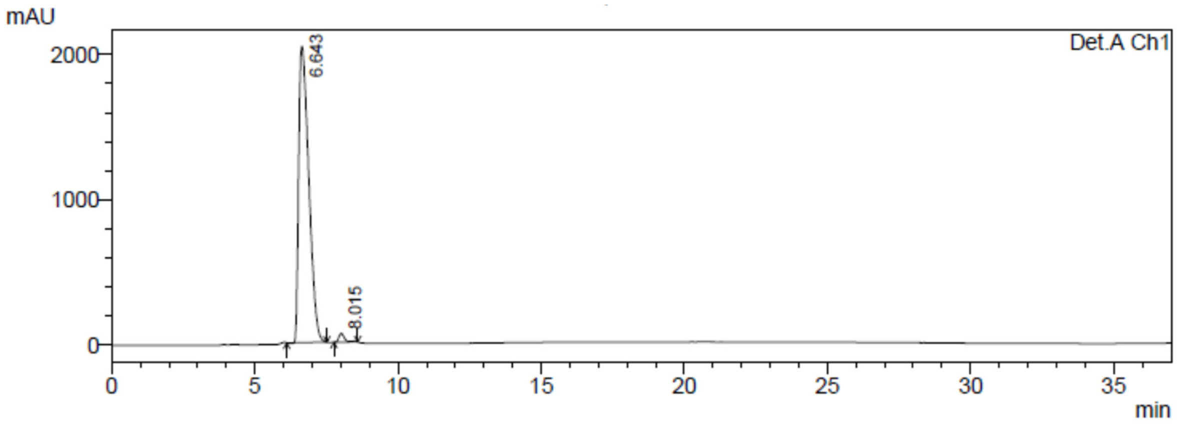

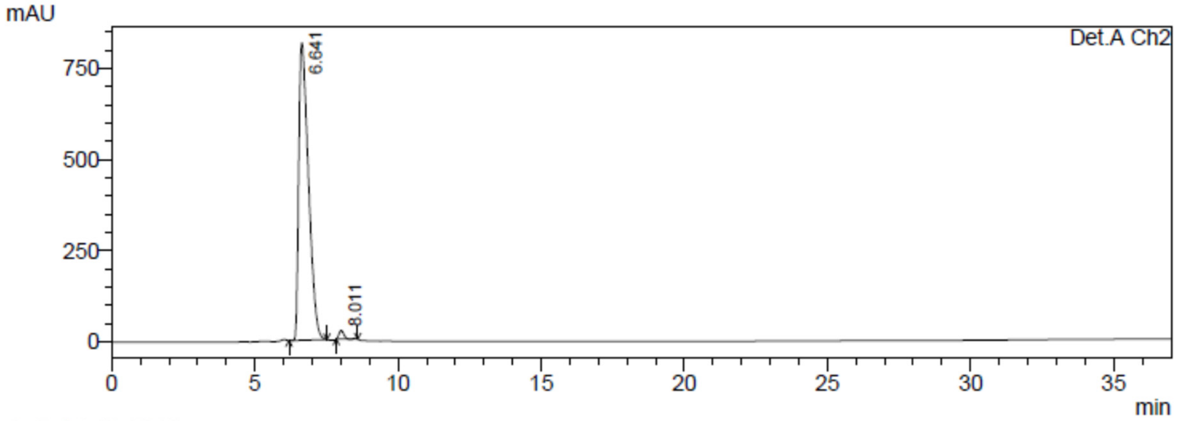

1 Det.A Ch1/215nm
2 Det.A Ch2/254nm

PeakTable

Detector A Ch1 215nm

Peak#	Ret. Time	Area	Area %
1	6.643	50092411	98.330
2	8.015	850599	1.670
Total		50943010	100.000

PeakTable

Detector A Ch2 254nm

Peak#	Ret. Time	Area	Area %
1	6.641	19288415	98.469
2	8.011	299981	1.531
Total		19588395	100.000

HPLC C18-column

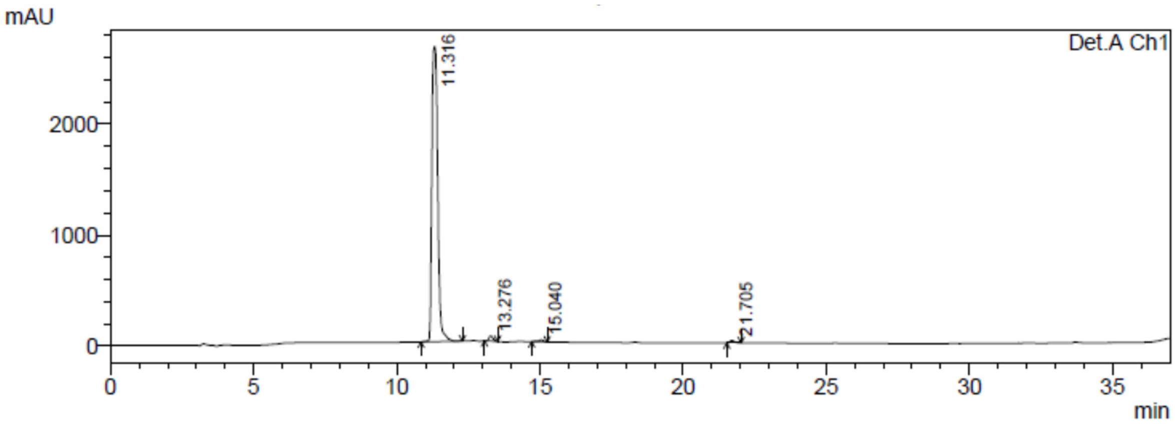

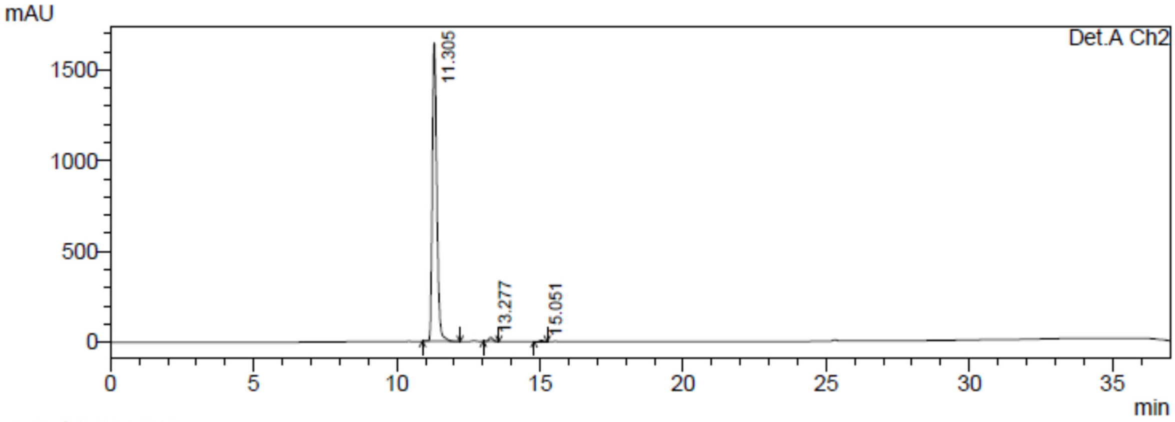

1 Det.A Ch1/215nm
2 Det.A Ch2/254nm

PeakTable

Detector A Ch1 215nm

Peak#	Ret. Time	Area	Area %
1	11.316	37894069	97.553
2	13.276	465743	1.199
3	15.040	234896	0.605
4	21.705	250015	0.644
Total		38844723	100.000

PeakTable

Detector A Ch2 254nm

Peak#	Ret. Time	Area	Area %
1	11.305	18098230	98.529
2	13.277	205397	1.118
3	15.051	64826	0.353
Total		18368453	100.000

3-[4-[4-(dimethylaminomethyl)-1-[3-[(4-methylpiperazin-1-yl)methyl]phenyl]pyrazol-3-yl]phenyl]-*N*,*N*-dimethyl-propan-1-amine (**30**)

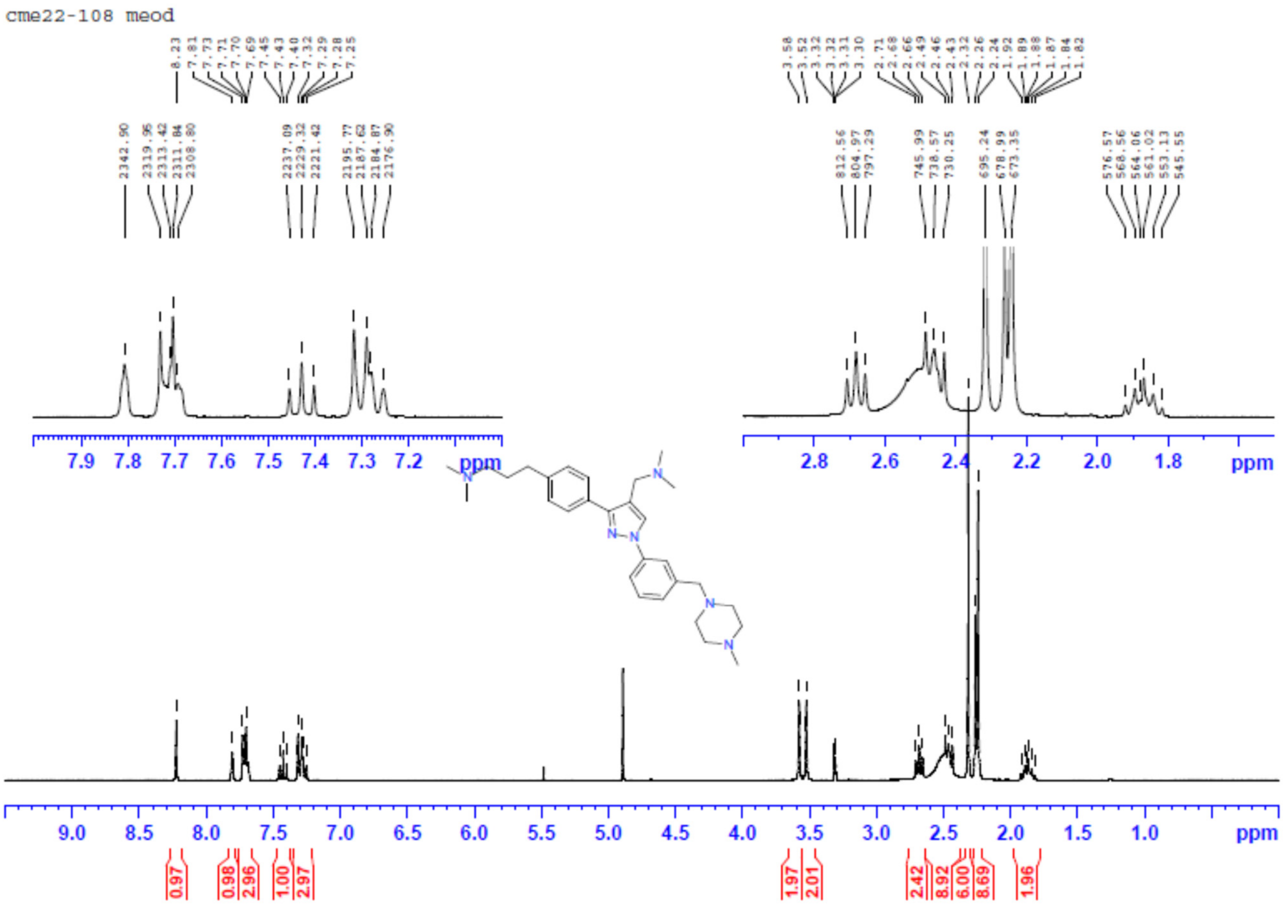

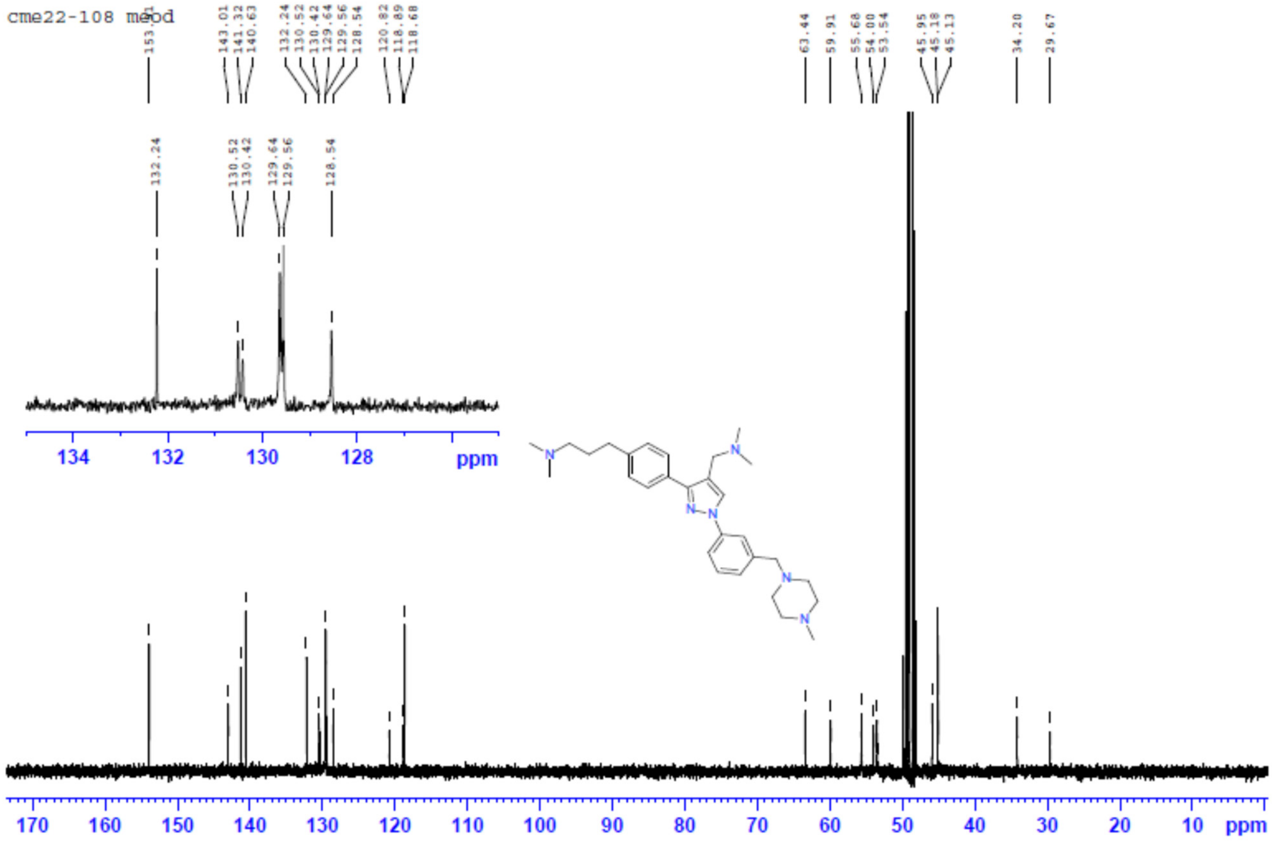

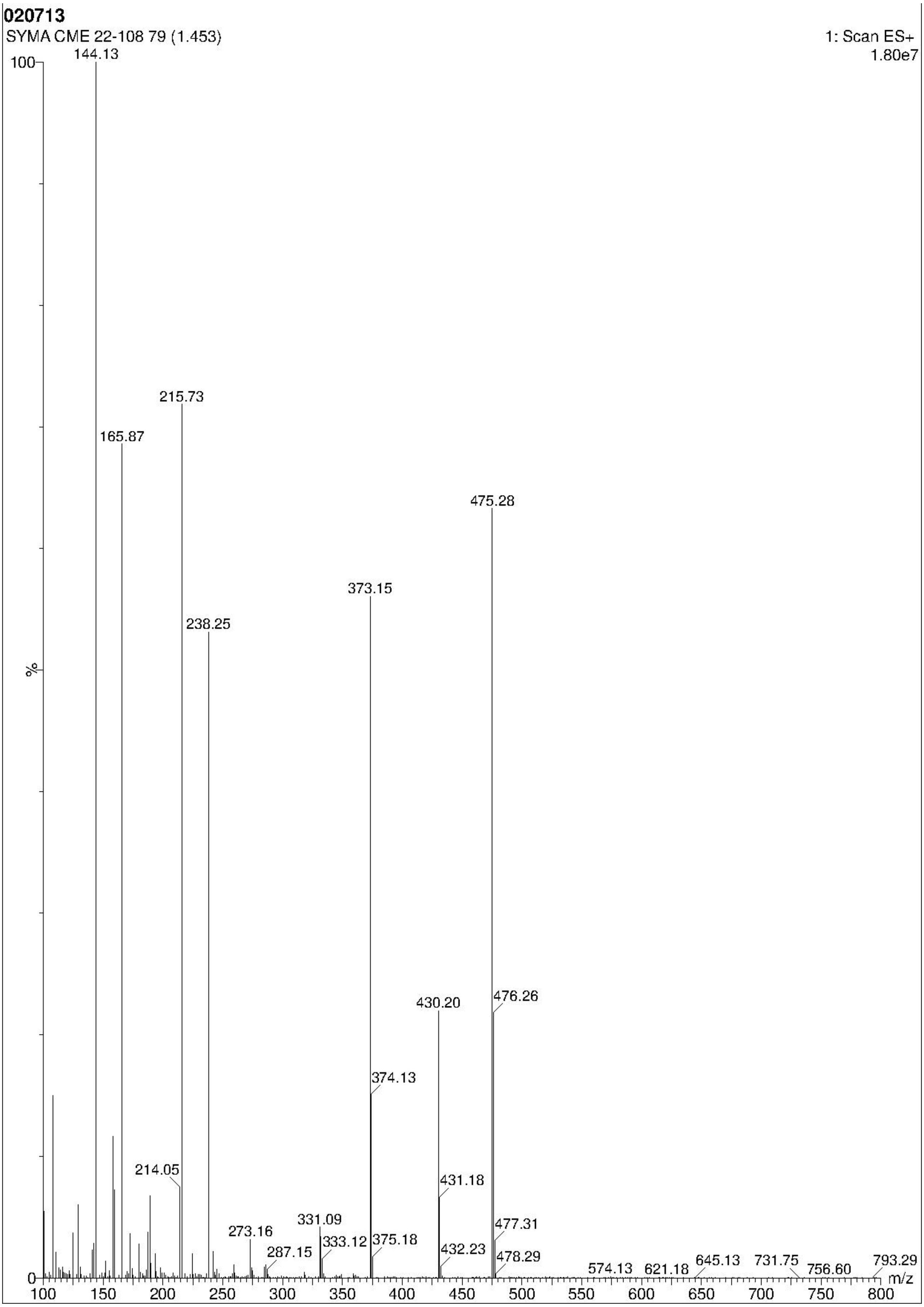
020713
SYMA CME 22-108 79 (1.453)
1: Scan ES+
1.80e7
%
m/z
144.13
215.73
165.87
475.28
373.15
238.25
430.20
476.26
374.13
214.05
431.18
331.09
477.31
273.16
333.12
375.18
287.15
432.23
478.29
574.13
621.18
645.13
731.75
756.60
793.29
100
150
200
250
300
350
400
450
500
550
600
650
700
750
800

HPLC C4-column

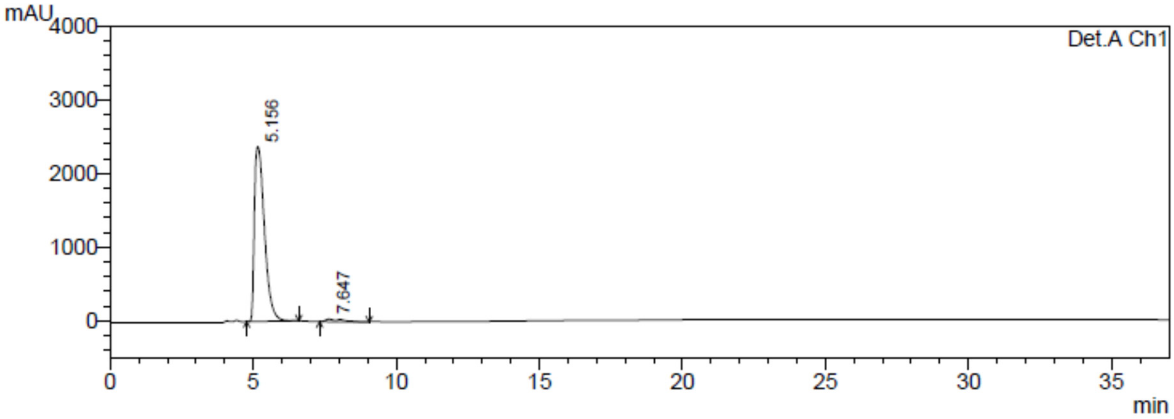

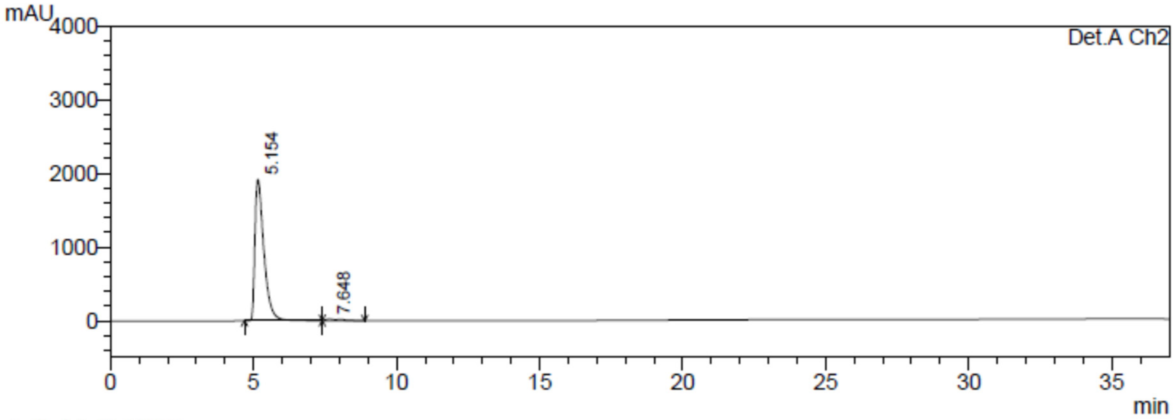

1 Det.A Ch1/215nm
2 Det.A Ch2/254nm

PeakTable

Detector A Ch1 215nm

Peak#	Ret. Time	Area	Area %
1	5.156	58093888	97.914
2	7.647	1237637	2.086
Total		59331525	100.000

PeakTable

Detector A Ch2 254nm

Peak#	Ret. Time	Area	Area %
1	5.154	41794906	98.095
2	7.648	811730	1.905
Total		42606636	100.000

HPLC C18-column

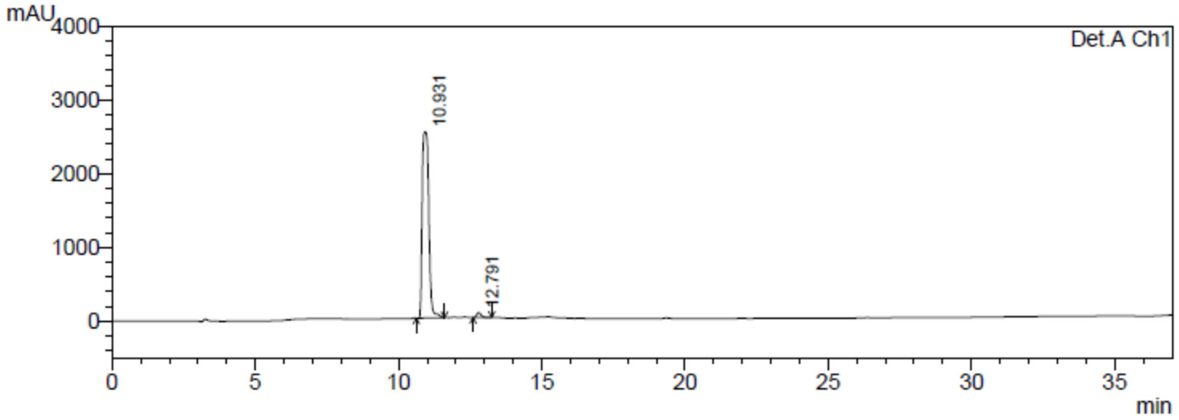

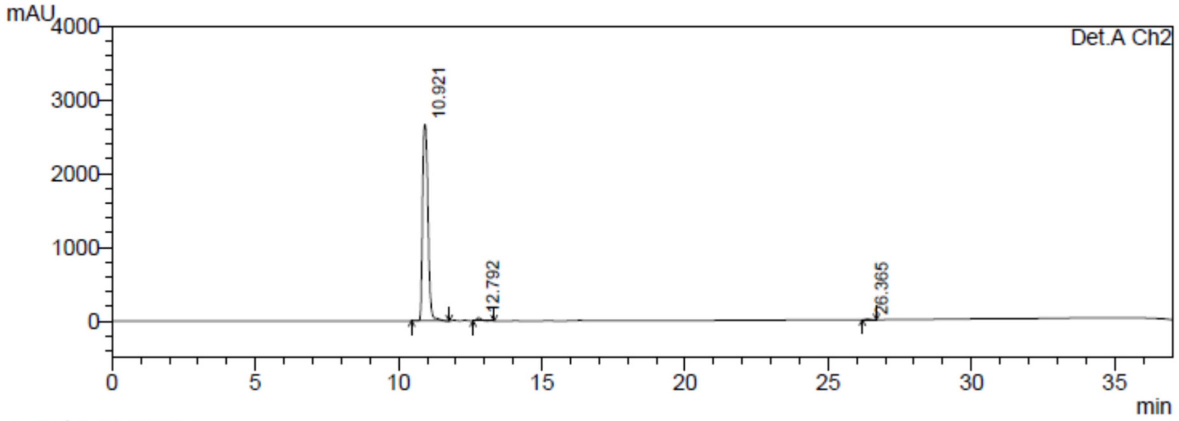

1 Det.A Ch1/215nm
2 Det.A Ch2/254nm

PeakTable

Detector A Ch1 215nm

Peak#	Ret. Time	Area	Area %
1	10.931	40427002	98.382
2	12.791	665002	1.618
Total		41092005	100.000

PeakTable

Detector A Ch2 254nm

Peak#	Ret. Time	Area	Area %
1	10.921	34857093	98.497
2	12.792	433322	1.224
3	26.365	98481	0.278
Total		35388895	100.000

3-[4-[4-[3-(dimethylamino)propyl]-1-[3-[(4-methylpiperazin-1-yl)methyl]phenyl]pyrazol-3-yl]phenyl]-*N*,*N*-dimethyl-propan-1-amine (**31**)

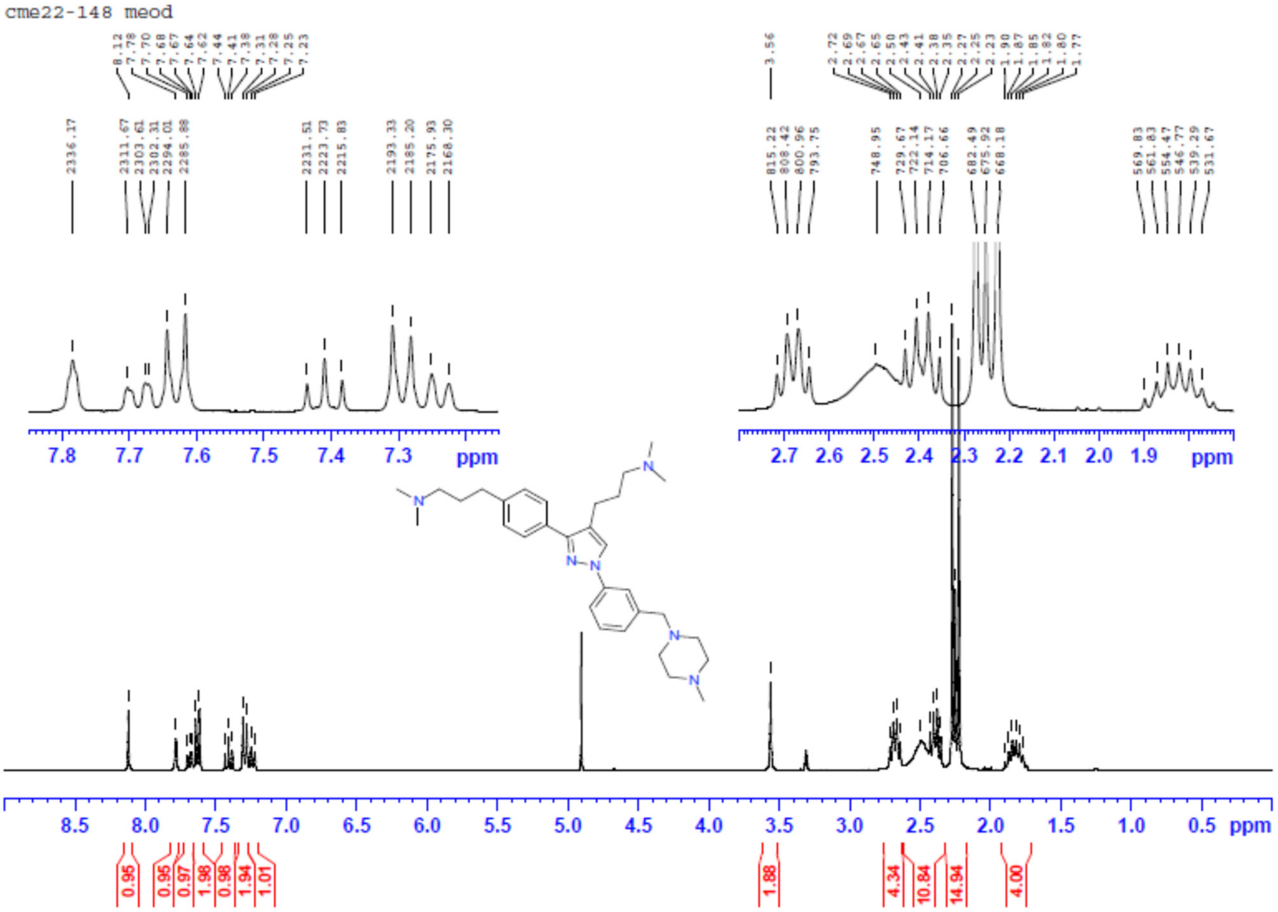

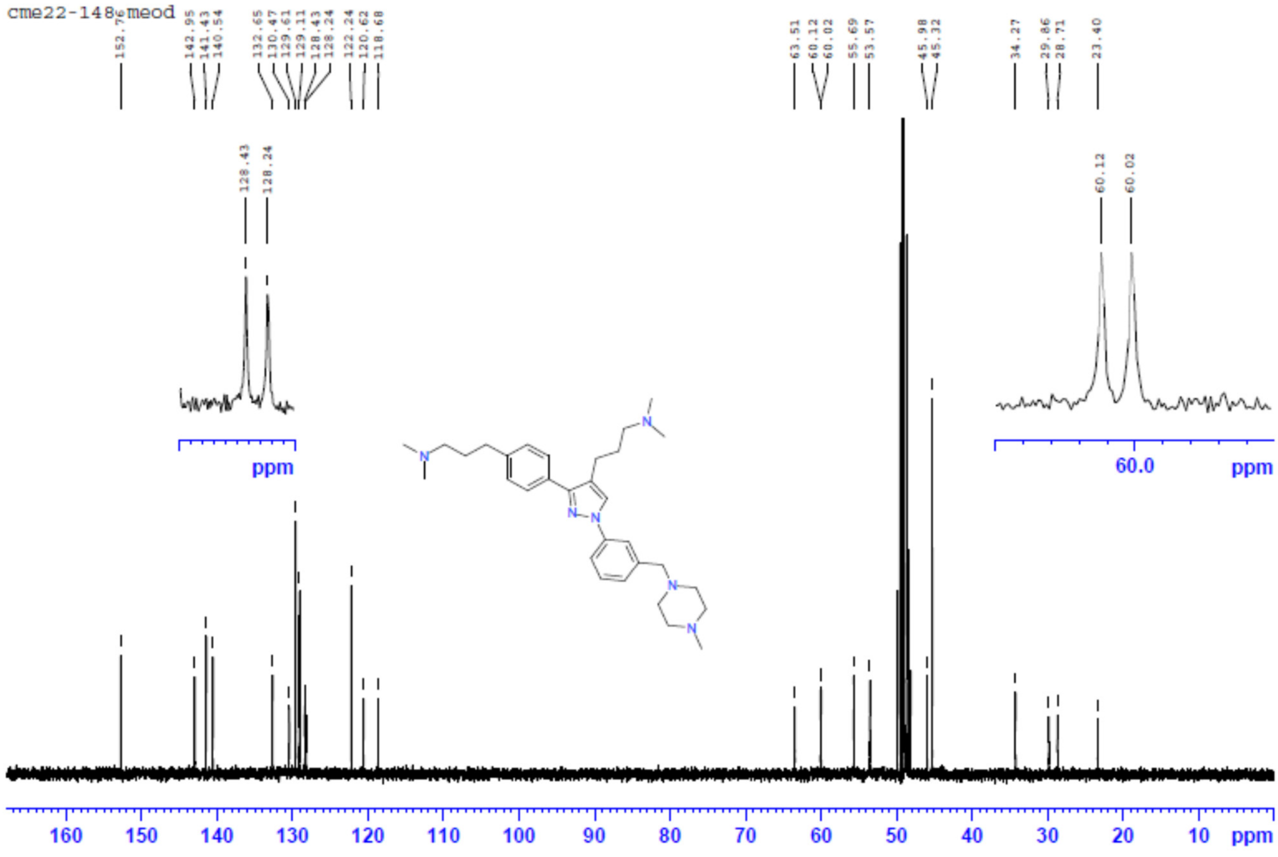

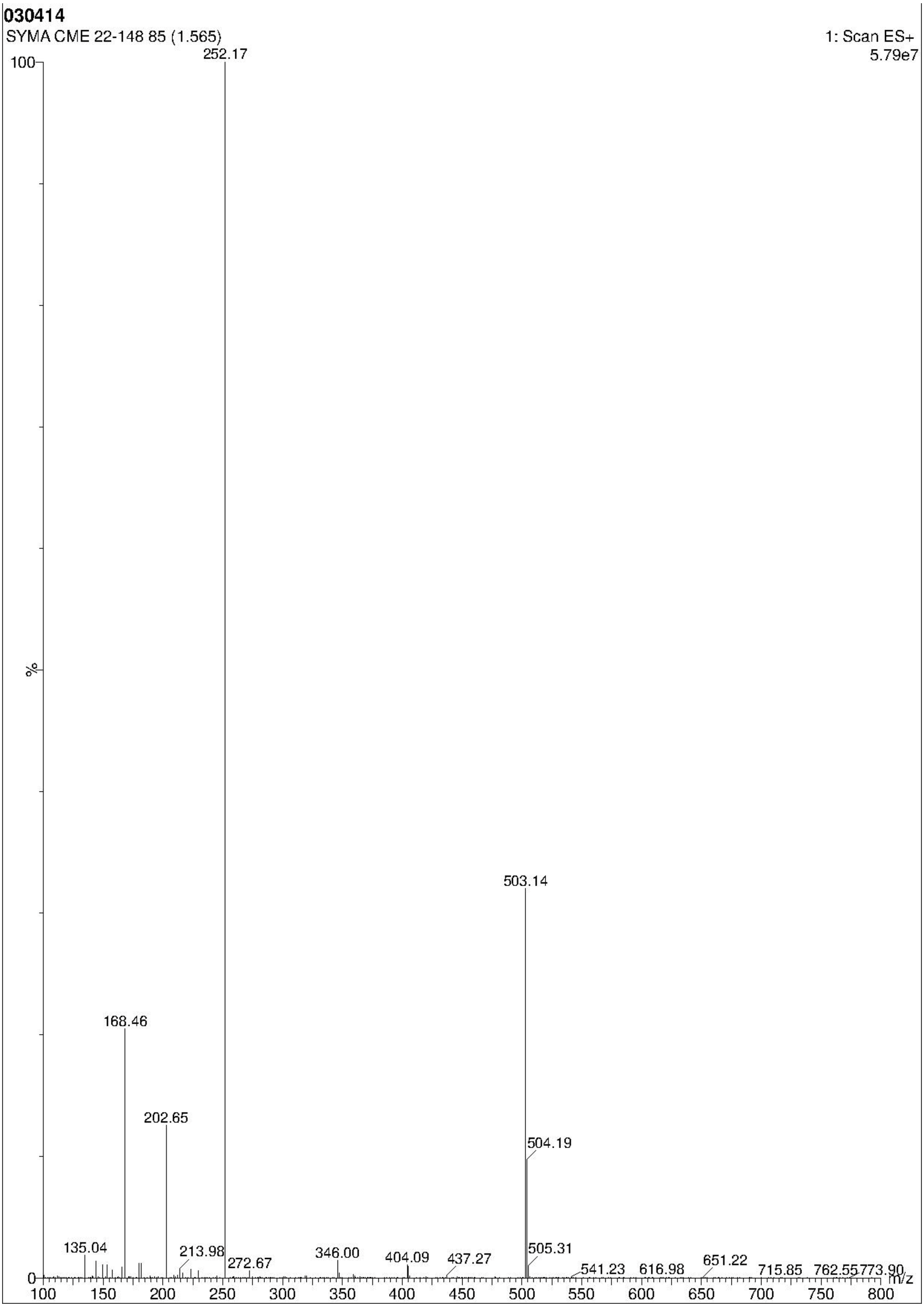

030414
SYMA CME 22-148 85 (1.565)
1: Scan ES+
5.79e7
252.17
503.14
168.46
202.65
504.19
135.04
213.98
272.67
346.00
404.09
437.27
505.31
541.23
616.98
651.22
715.85
762.55
773.90
m/z
%
100
0
100
150
200
250
300
350
400
450
500
550
600
650
700
750
800

HPLC C4-column

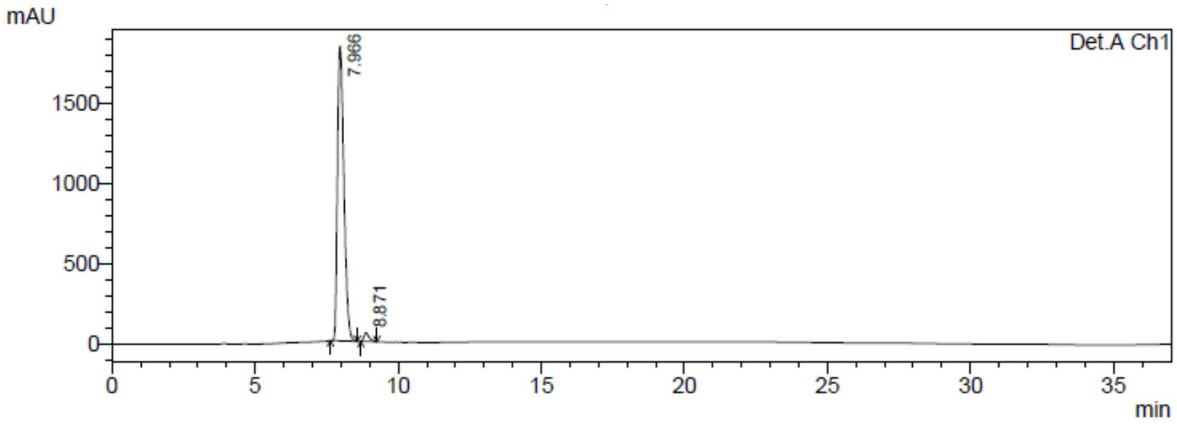

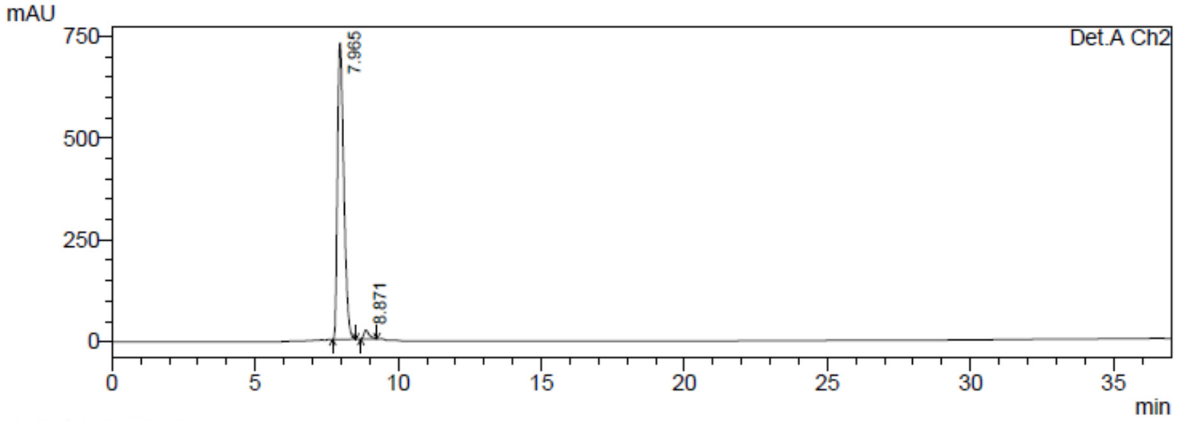

1 Det.A Ch1/215nm
2 Det.A Ch2/254nm

PeakTable

Detector A Ch1 215nm

Peak#	Ret. Time	Area	Area %
1	7.966	28775837	97.633
2	8.871	697551	2.367
Total		29473388	100.000

PeakTable

Detector A Ch2 254nm

Peak#	Ret. Time	Area	Area %
1	7.965	11177187	97.508
2	8.871	285702	2.492
Total		11462889	100.000

HPLC C18-column

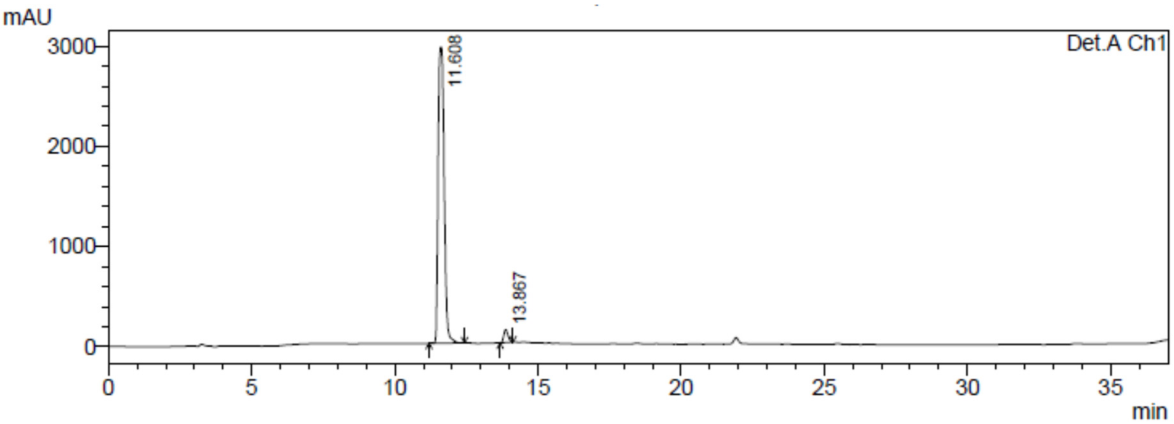

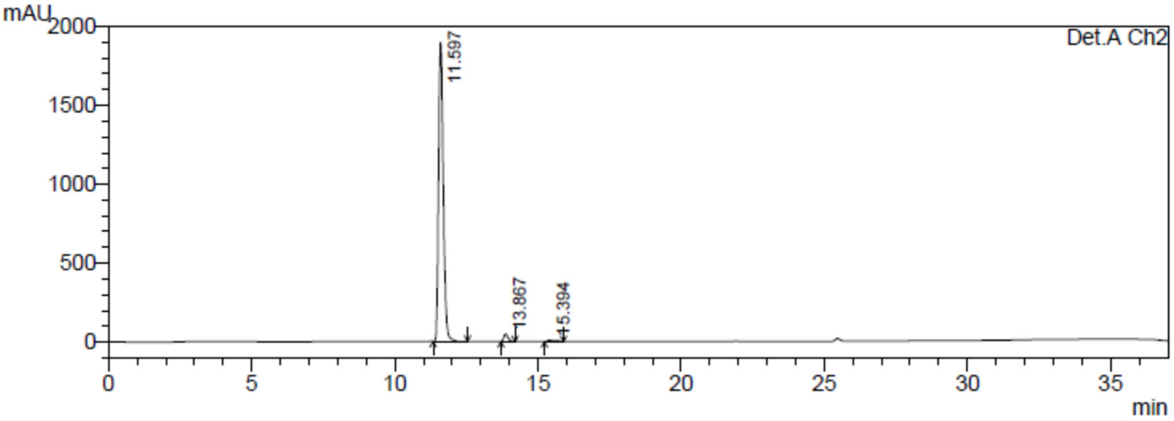

1 Det.A Ch1/215nm
2 Det.A Ch2/254nm

PeakTable

Detector A Ch1 215nm

Peak#	Ret. Time	Area	Area %
1	11.608	43519268	97.064
2	13.867	1316501	2.936
Total		44835769	100.000

PeakTable

Detector A Ch2 254nm

Peak#	Ret. Time	Area	Area %
1	11.597	21764263	97.007
2	13.867	497582	2.218
3	15.394	174017	0.776
Total		22435861	100.000

N'-[[4-[4-(dimethylaminomethyl)-3-[4-[3-(dimethylamino)propyl]phenyl]pyrazol-1-yl]phenyl]methyl]-*N*,*N*,*N'*-trimethyl-propane-1,3-diamine (**32**)

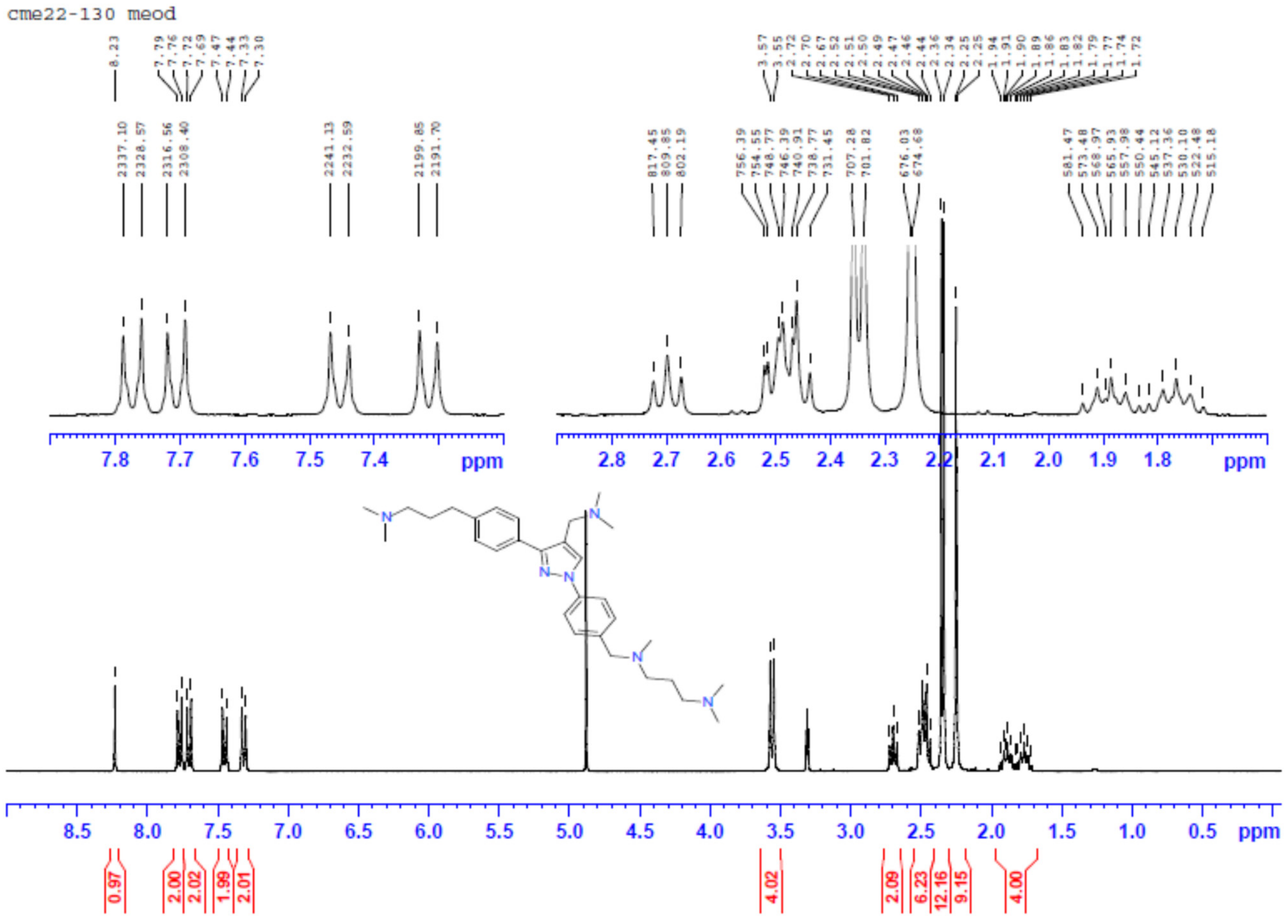

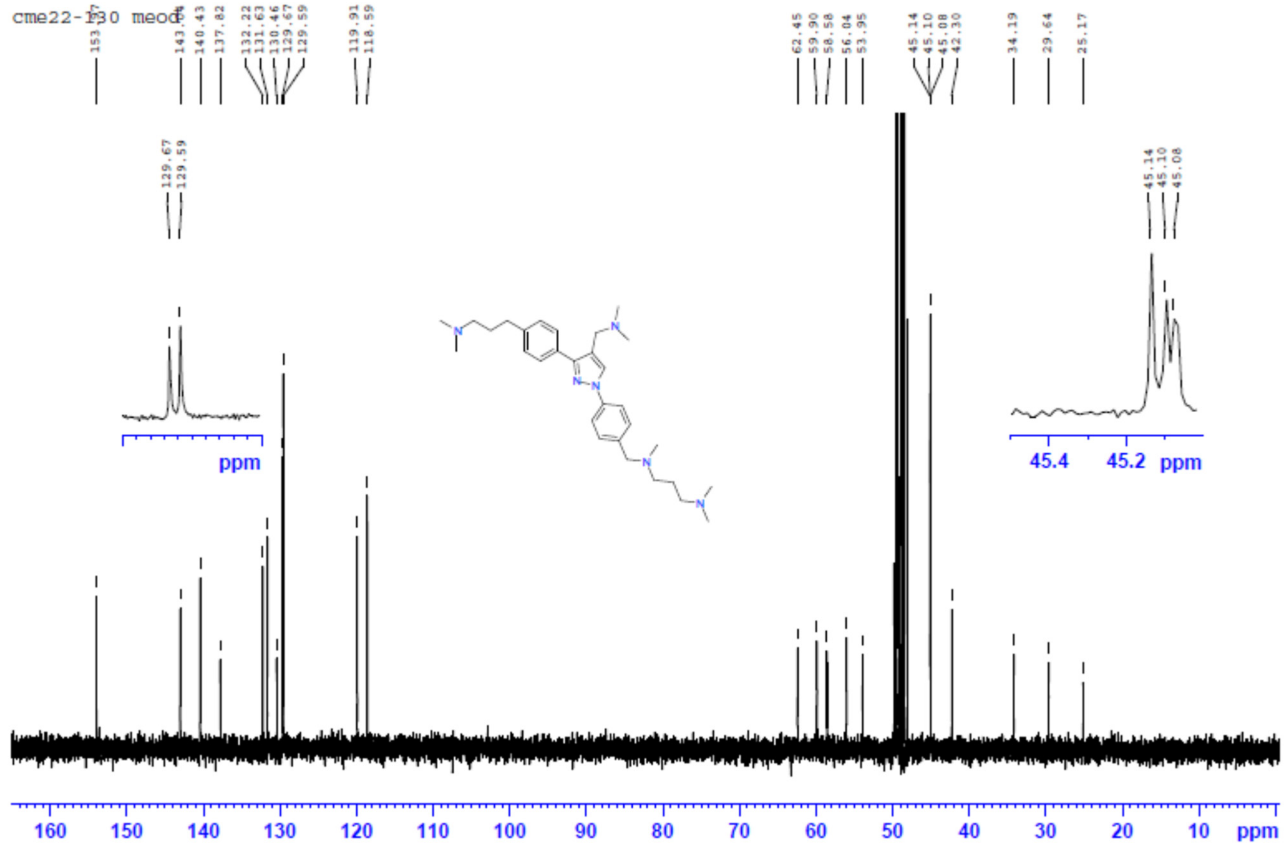

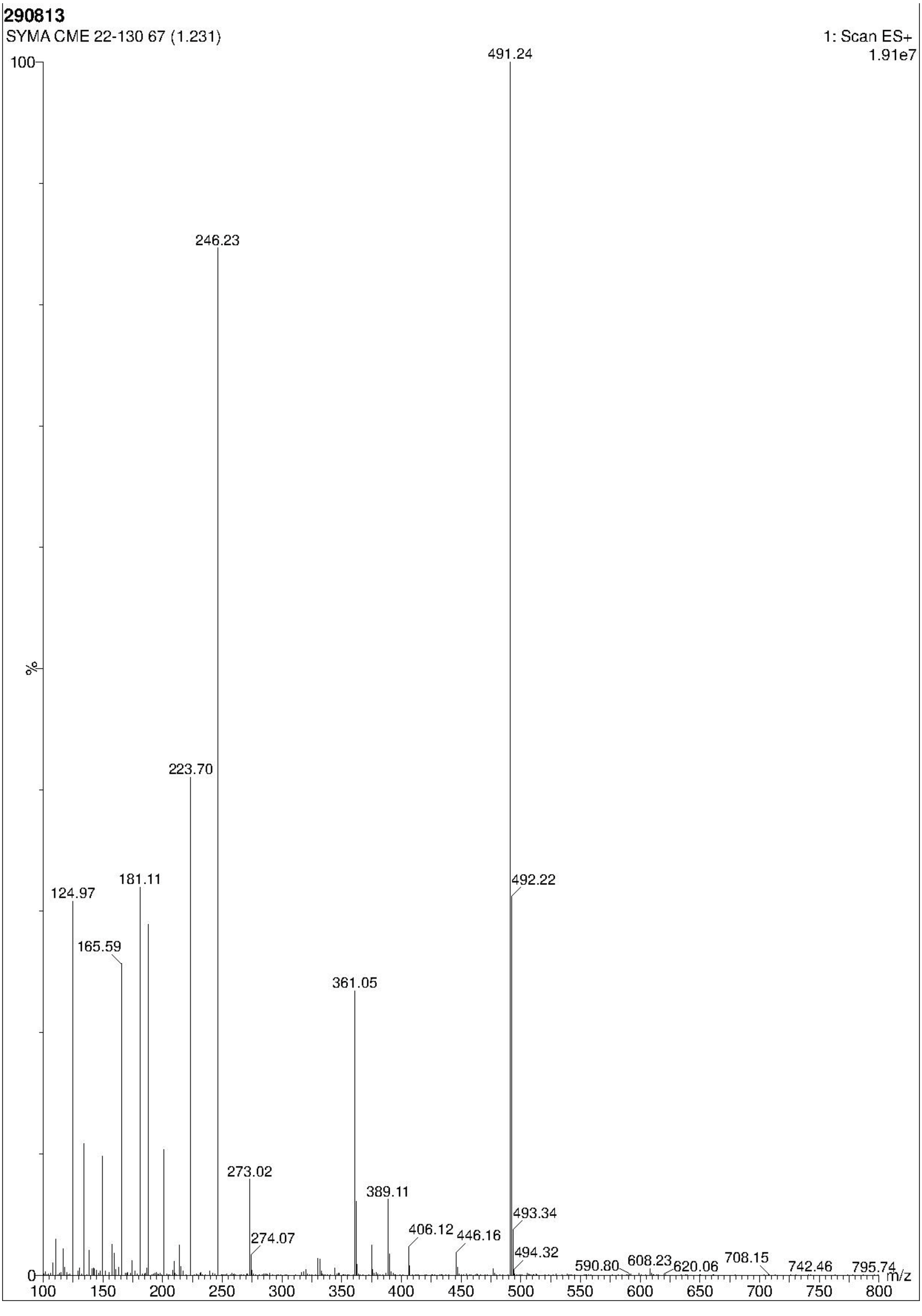
290813
SYMA CME 22-130 67 (1.231)
1: Scan ES+
1.91e7
491.24
246.23
223.70
181.11
124.97
165.59
492.22
361.05
273.02
389.11
493.34
274.07
406.12
446.16
494.32
590.80
608.23
620.06
708.15
742.46
795.74
%
m/z
100
150
200
250
300
350
400
450
500
550
600
650
700
750
800
0

HPLC C4-column

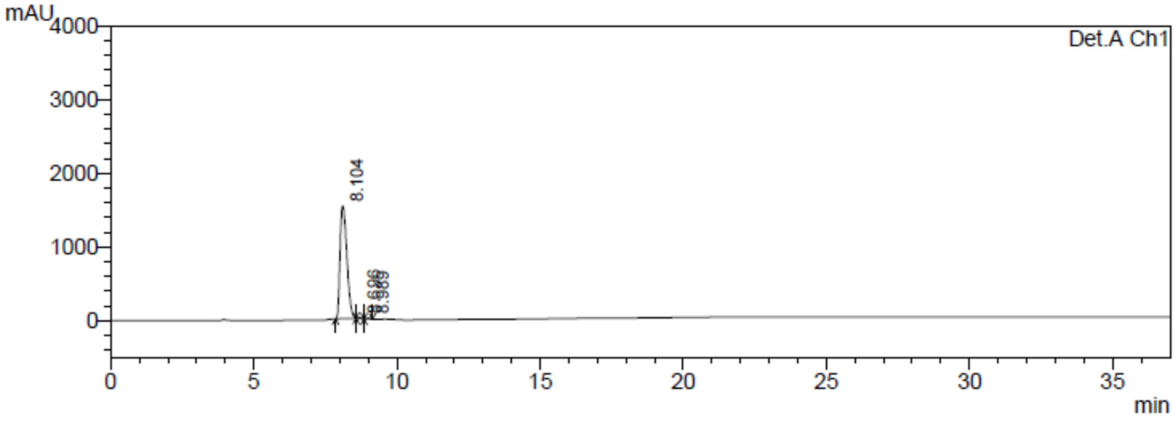

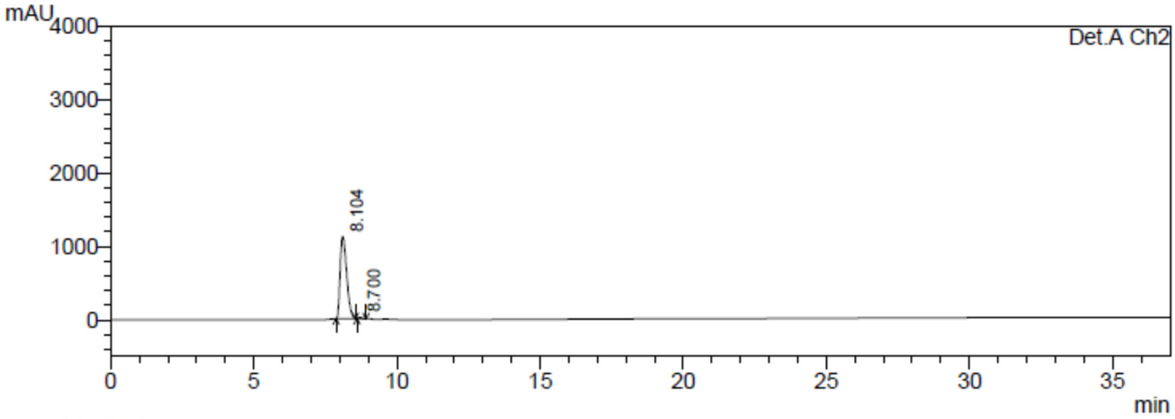

1 Det.A Ch1/215nm
2 Det.A Ch2/254nm

PeakTable

Detector A Ch1 215nm

Peak#	Ret. Time	Area	Area %
1	8.104	25272467	99.174
2	8.696	84951	0.333
3	8.989	125414	0.492
Total		25482832	100.000

PeakTable

Detector A Ch2 254nm

Peak#	Ret. Time	Area	Area %
1	8.104	17638220	99.274
2	8.700	129061	0.726
Total		17767281	100.000

HPLC C18-column

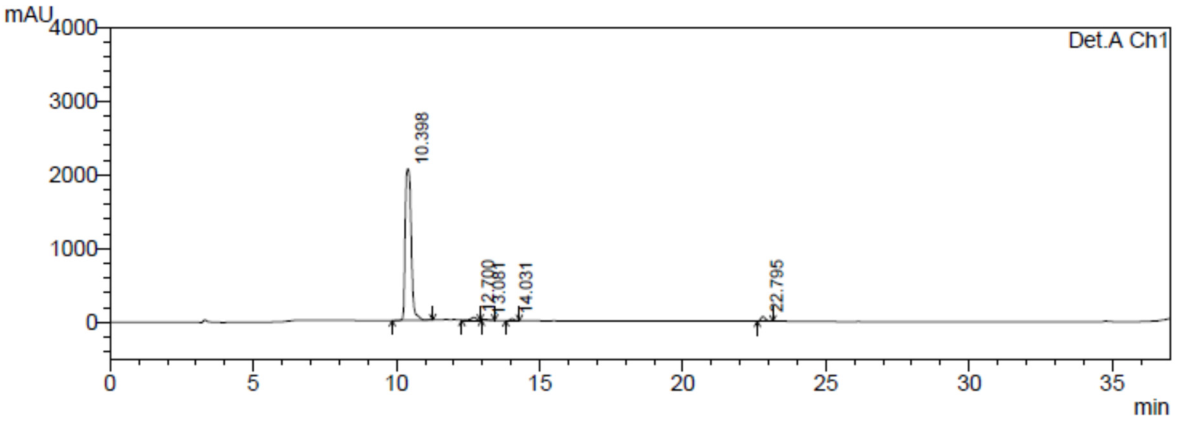

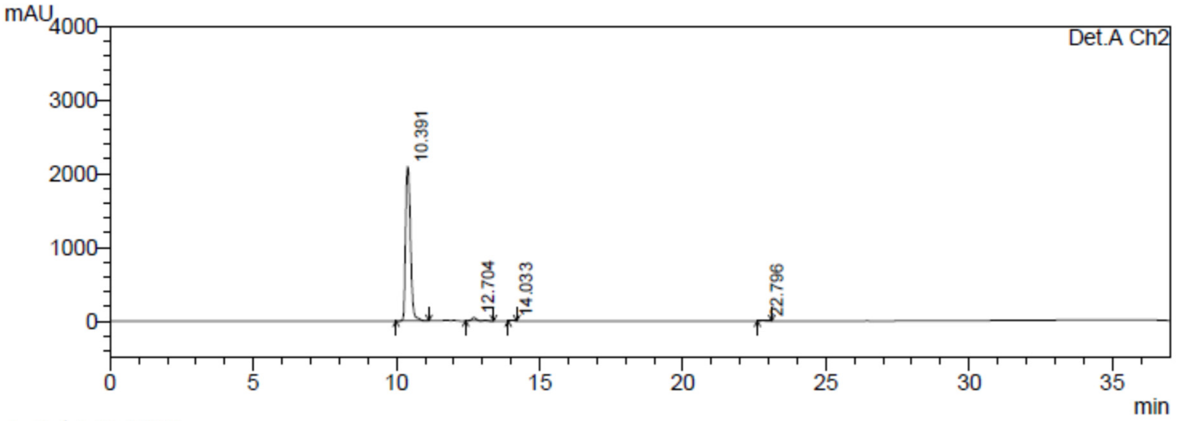

1 Det.A Ch1/215nm
2 Det.A Ch2/254nm

PeakTable

Detector A Ch1 215nm

Peak#	Ret. Time	Area	Area %
1	10.398	29673687	95.105
2	12.700	478873	1.535
3	13.081	121454	0.389
4	14.031	229791	0.736
5	22.795	697287	2.235
Total		31201093	100.000

PeakTable

Detector A Ch2 254nm

Peak#	Ret. Time	Area	Area %
1	10.391	24848240	97.349
2	12.704	560318	2.195
3	14.033	75340	0.295
4	22.796	41102	0.161
Total		25525000	100.000

N'-[[4-[4-[3-(dimethylamino)propyl]-3-[4-[3-(dimethylamino)propyl]phenyl]pyrazol-1-yl]phenyl]methyl]-*N*,*N*,*N'*-trimethyl-propane-1,3-diamine (**33**)

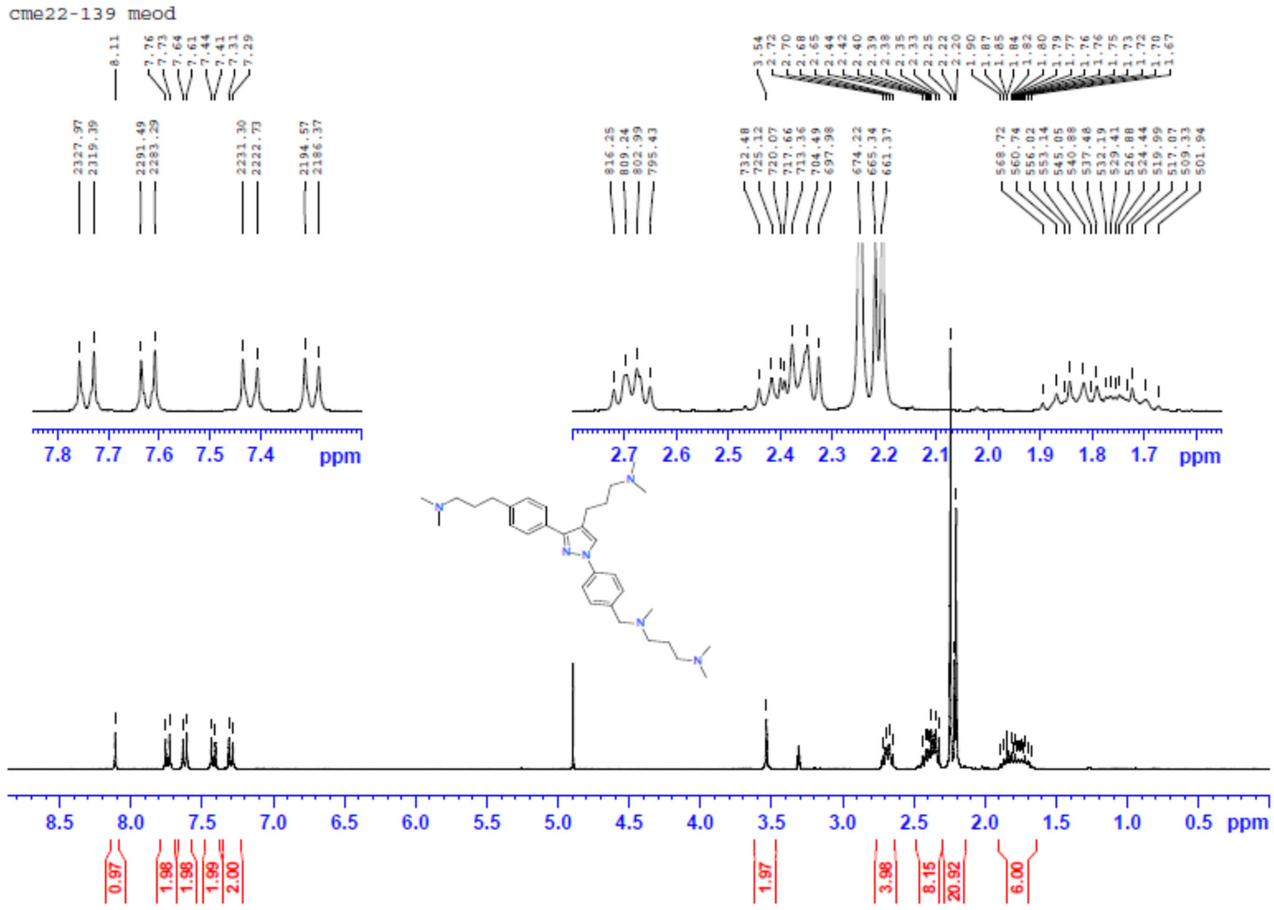

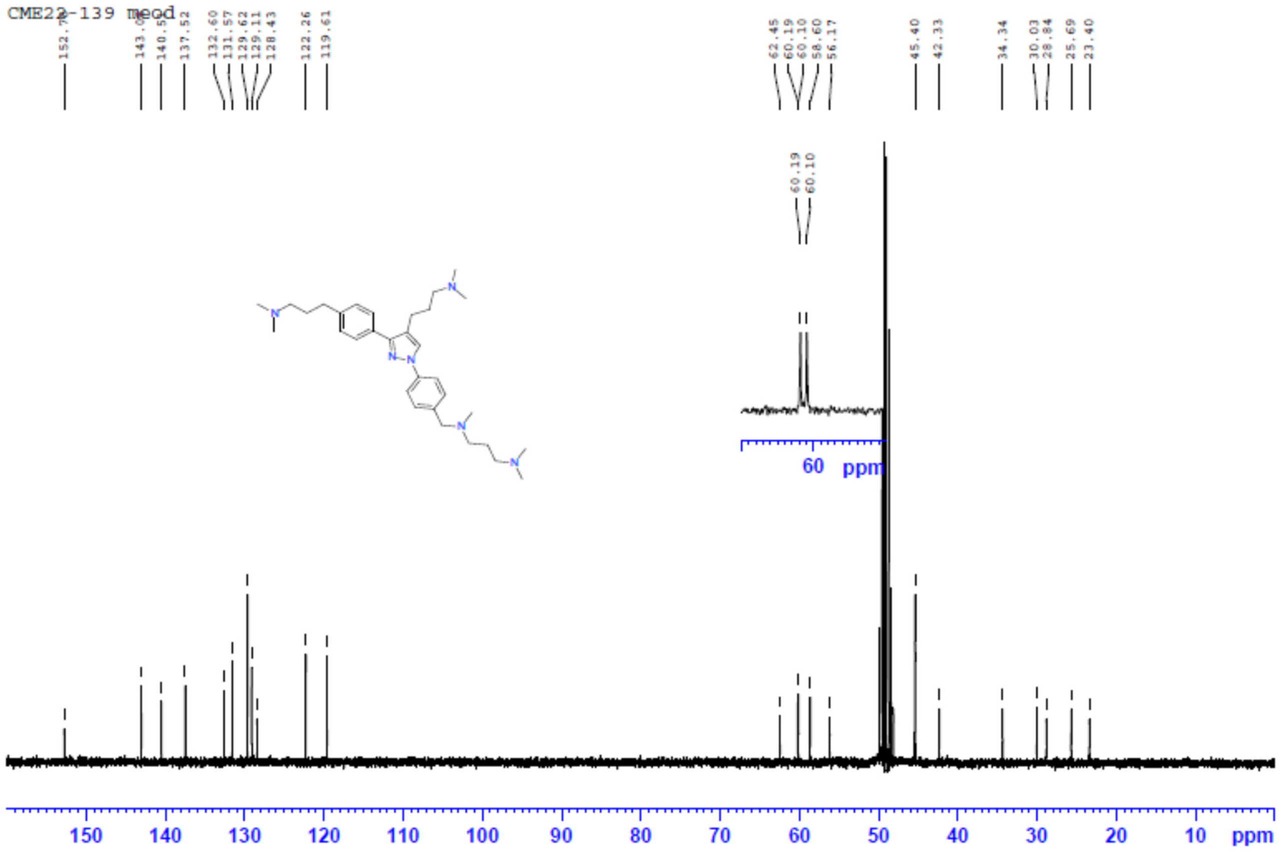

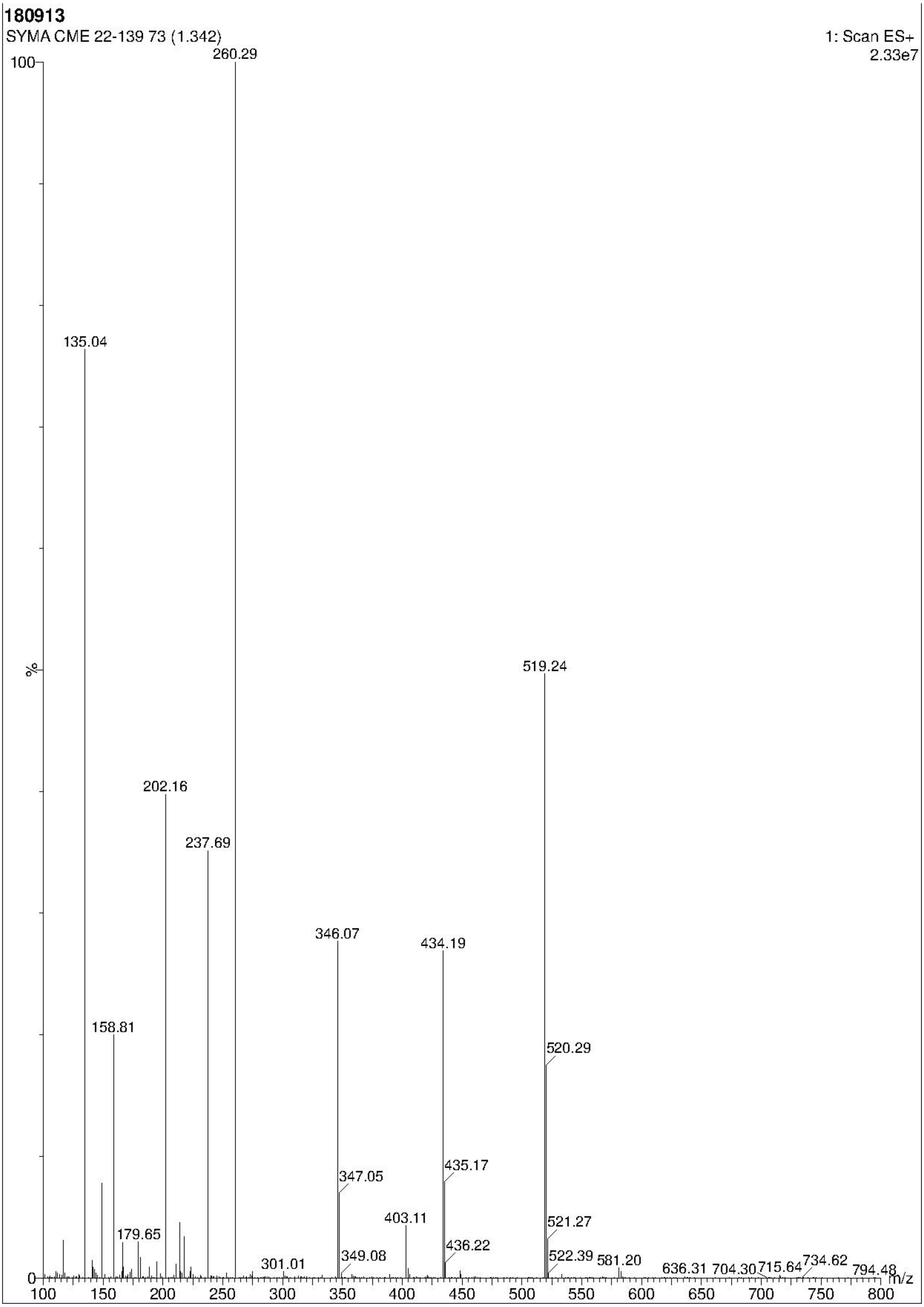
180913
SYMA CME 22-139 73 (1.342)
1: Scan ES+
2.33e7
100
%
0
260.29
135.04
519.24
202.16
237.69
346.07
434.19
158.81
520.29
435.17
347.05
403.11
521.27
179.65
436.22
349.08
522.39
581.20
301.01
636.31
704.30
715.64
734.62
794.48
m/z
100
150
200
250
300
350
400
450
500
550
600
650
700
750
800

HPLC C4-column

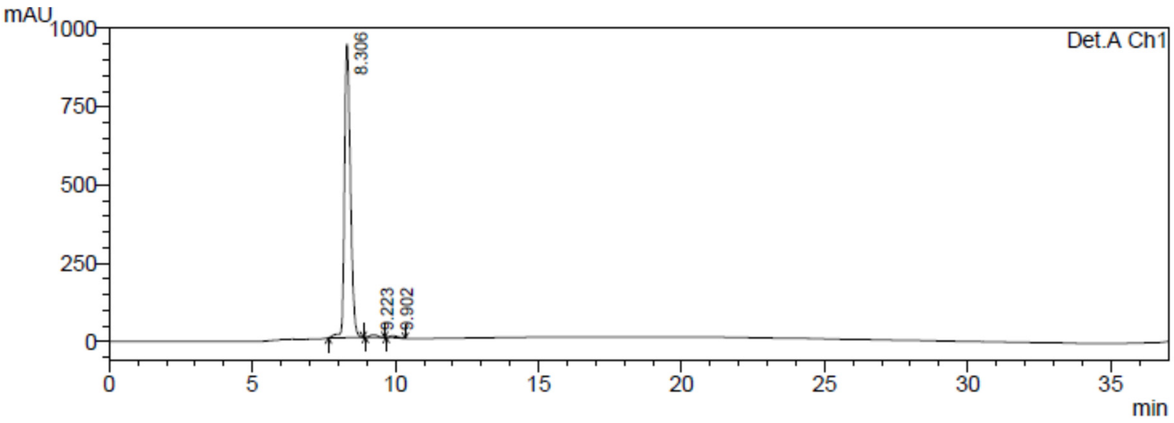

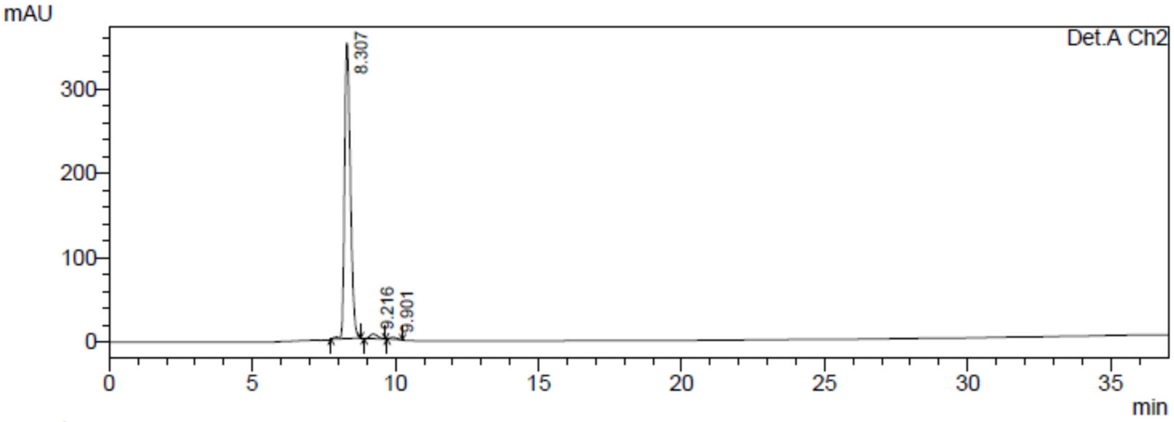

1 Det.A Ch1/215nm
2 Det.A Ch2/254nm

PeakTable

Detector A Ch1 215nm

Peak#	Ret. Time	Area	Area %
1	8.306	13554593	98.066
2	9.223	149699	1.083
3	9.902	117647	0.851
Total		13821939	100.000

PeakTable

Detector A Ch2 254nm

Peak#	Ret. Time	Area	Area %
1	8.307	4956549	97.256
2	9.216	102715	2.015
3	9.901	37121	0.728
Total		5096385	100.000

HPLC C18-column

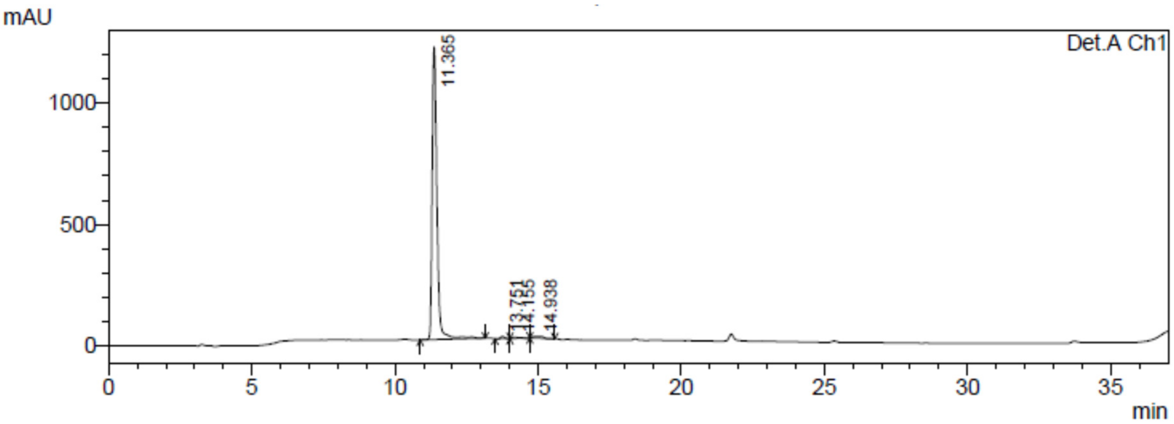

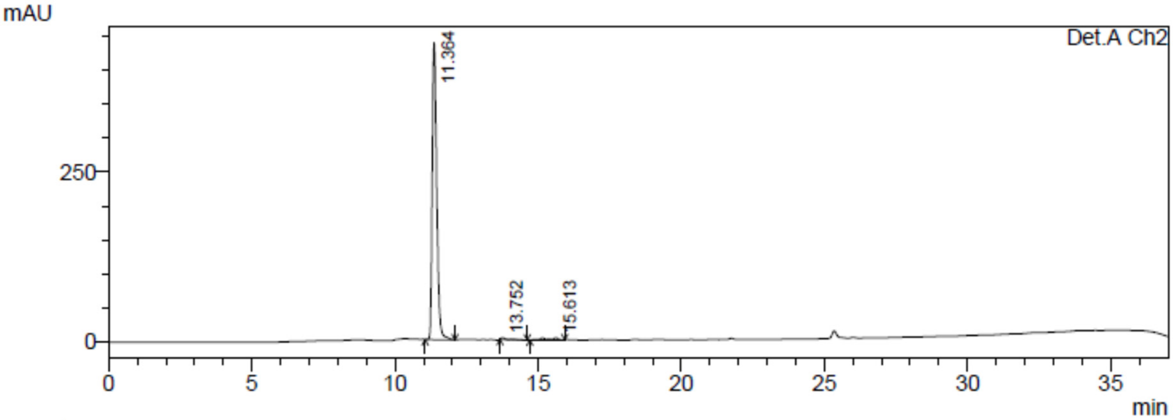

1 Det.A Ch1/215nm
2 Det.A Ch2/254nm

PeakTable

Detector A Ch1 215nm

Peak#	Ret. Time	Area	Area %
1	11.365	13947963	97.847
2	13.751	72658	0.510
3	14.155	82597	0.579
4	14.938	151615	1.064
Total		14254834	100.000

PeakTable

Detector A Ch2 254nm

Peak#	Ret. Time	Area	Area %
1	11.364	4777739	97.699
2	13.752	27440	0.561
3	15.613	85107	1.740
Total		4890287	100.000

N'-[[3-[4-(dimethylaminomethyl)-3-[4-[3-(dimethylamino)propyl]phenyl]pyrazol-1-yl]phenyl]methyl]-*N*,*N*,*N'*-trimethyl-propane-1,3-diamine (**34**)

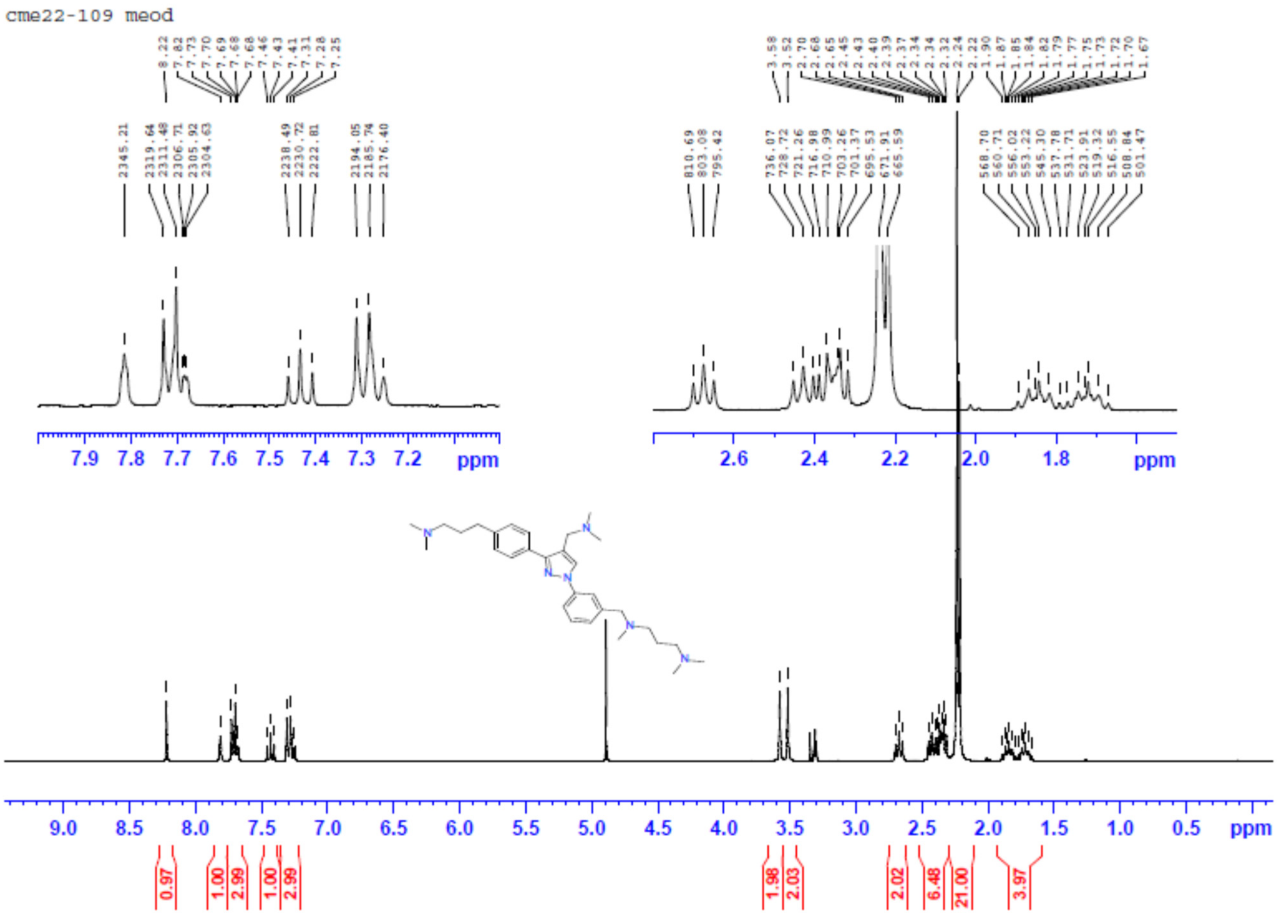

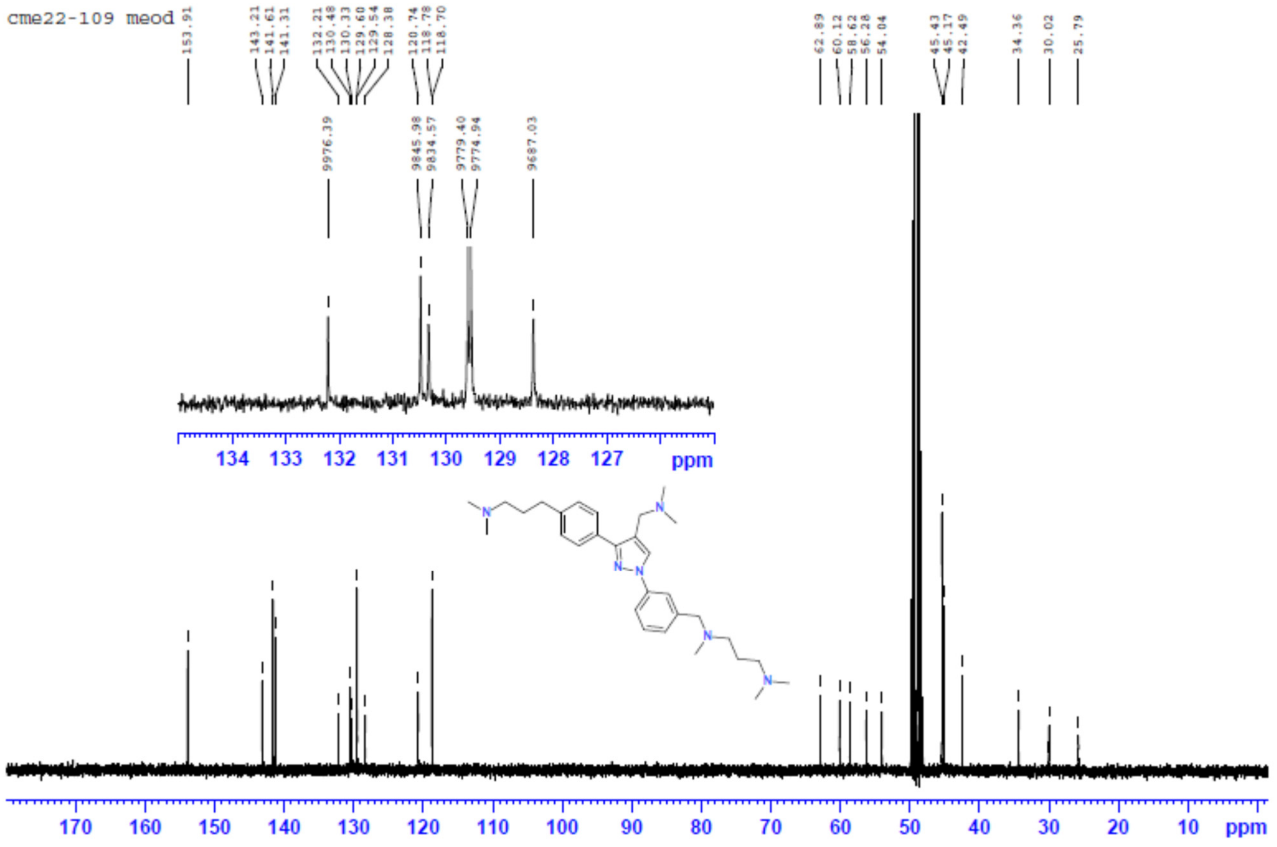

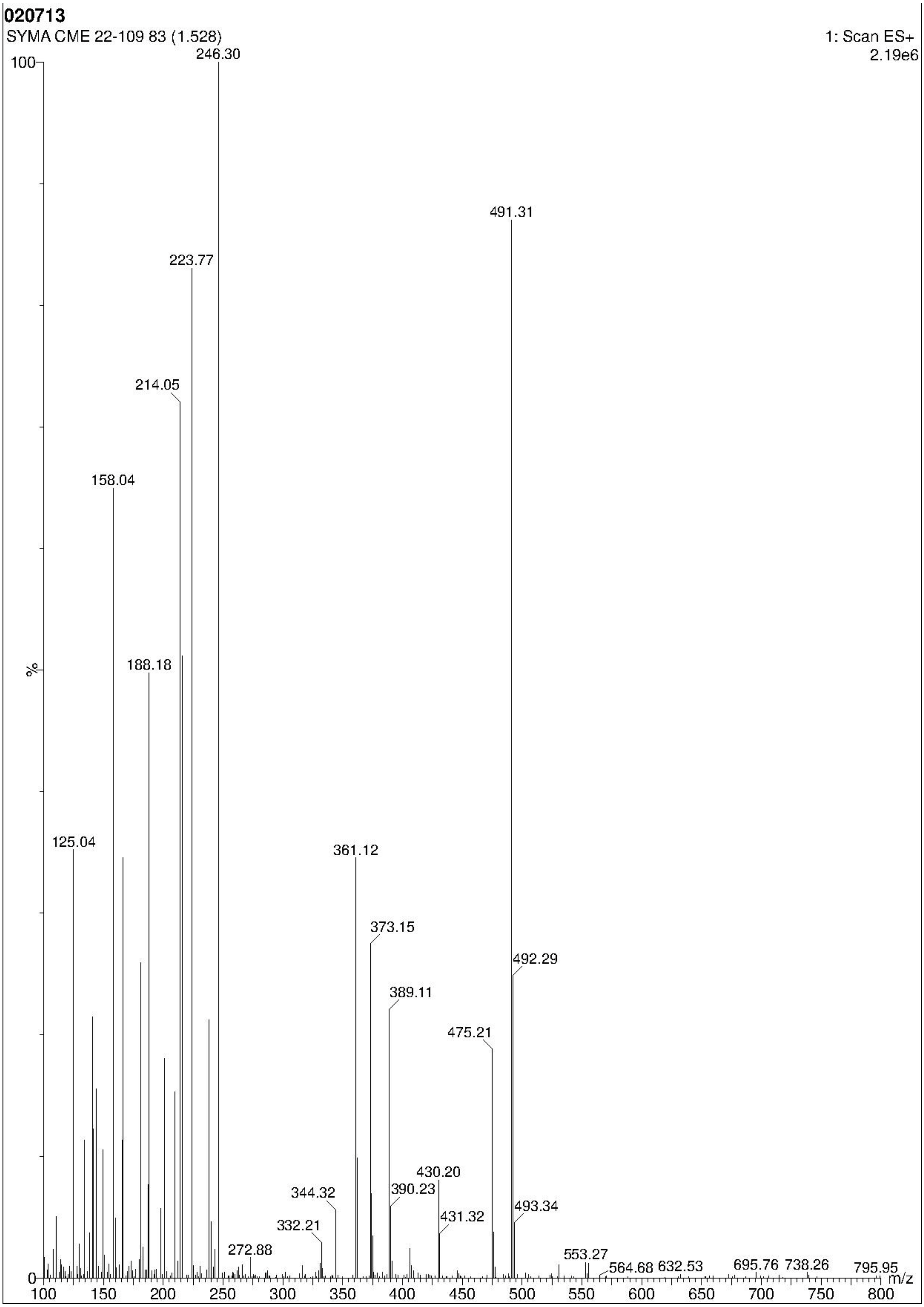
020713
SYMA CME 22-109 83 (1.528)
1: Scan ES+
2.19e6
246.30
491.31
223.77
214.05
158.04
188.18
125.04
361.12
373.15
492.29
389.11
475.21
430.20
344.32
390.23
493.34
431.32
332.21
272.88
553.27
564.68
632.53
695.76
738.26
795.95
%
m/z

HPLC C4-column

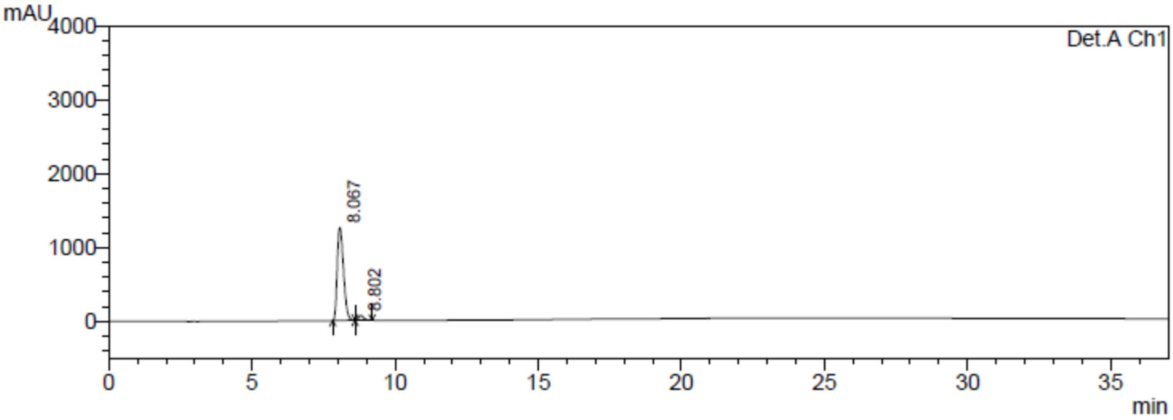

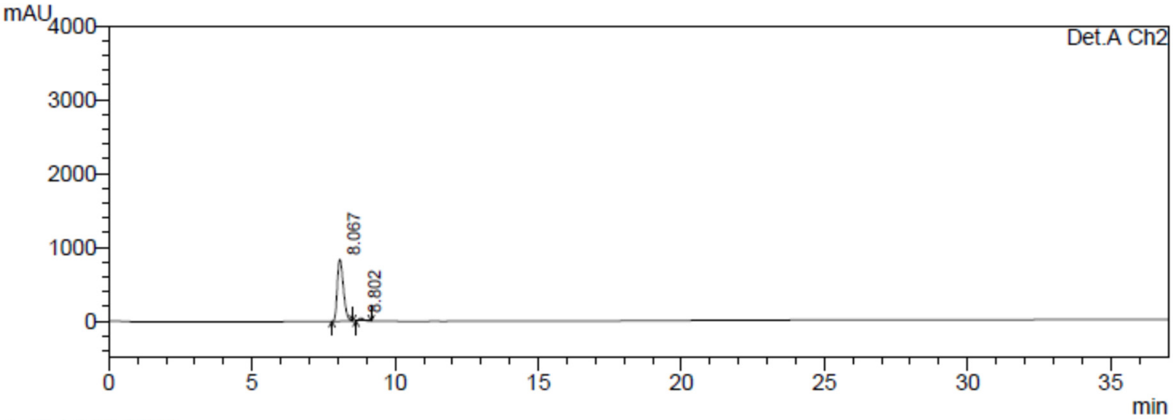

1 Det.A Ch1/215nm
2 Det.A Ch2/254nm

PeakTable

Detector A Ch1 215nm

Peak#	Ret. Time	Area	Area %
1	8.067	19197644	96.116
2	8.802	775778	3.884
Total		19973421	100.000

PeakTable

Detector A Ch2 254nm

Peak#	Ret. Time	Area	Area %
1	8.067	12413093	96.391
2	8.802	464802	3.609
Total		12877895	100.000

HPLC C18-column

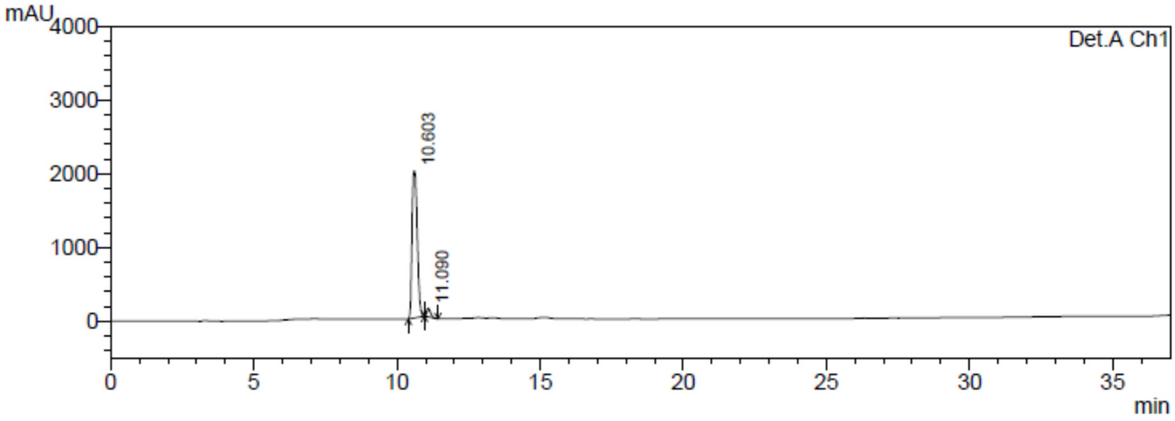

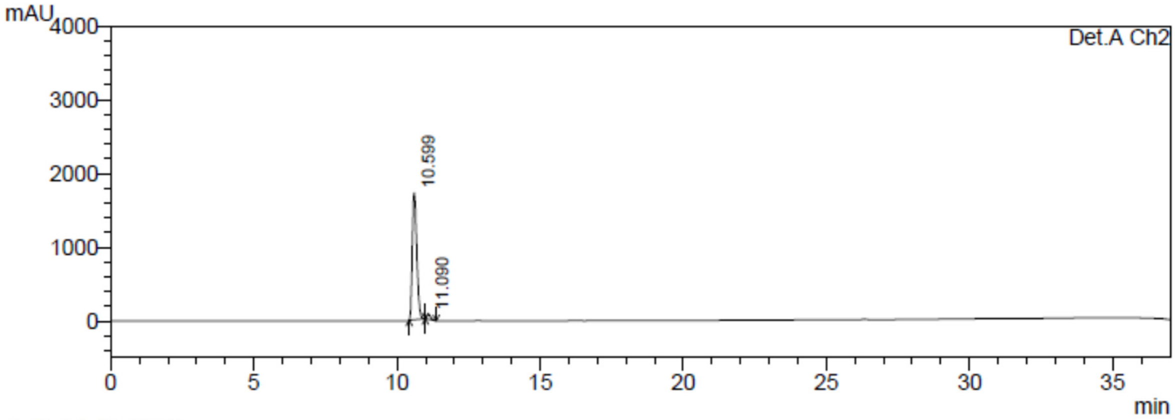

1 Det.A Ch1/215nm
2 Det.A Ch2/254nm

PeakTable

Detector A Ch1 215nm

Peak#	Ret. Time	Area	Area %
1	10.603	25534542	96.010
2	11.090	1061290	3.990
Total		26595832	100.000

PeakTable

Detector A Ch2 254nm

Peak#	Ret. Time	Area	Area %
1	10.599	18818000	96.997
2	11.090	582506	3.003
Total		19400506	100.000

N'-[[3-[4-[3-(dimethylamino)propyl]-3-[4-[3-(dimethylamino)propyl]phenyl]pyrazol-1-yl]phenyl]methyl]-*N*,*N*,*N'*-trimethyl-propane-1,3-diamine (**35**)

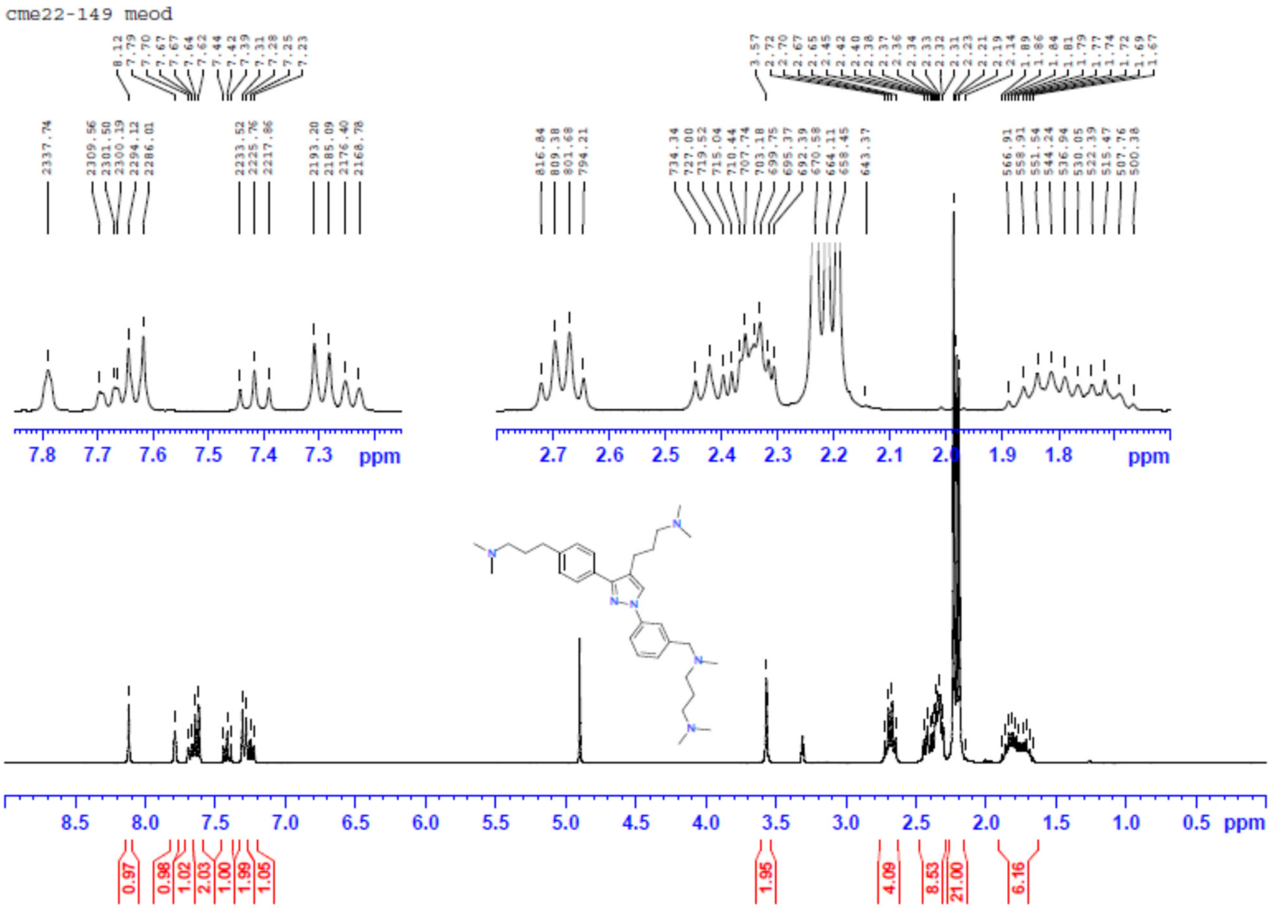

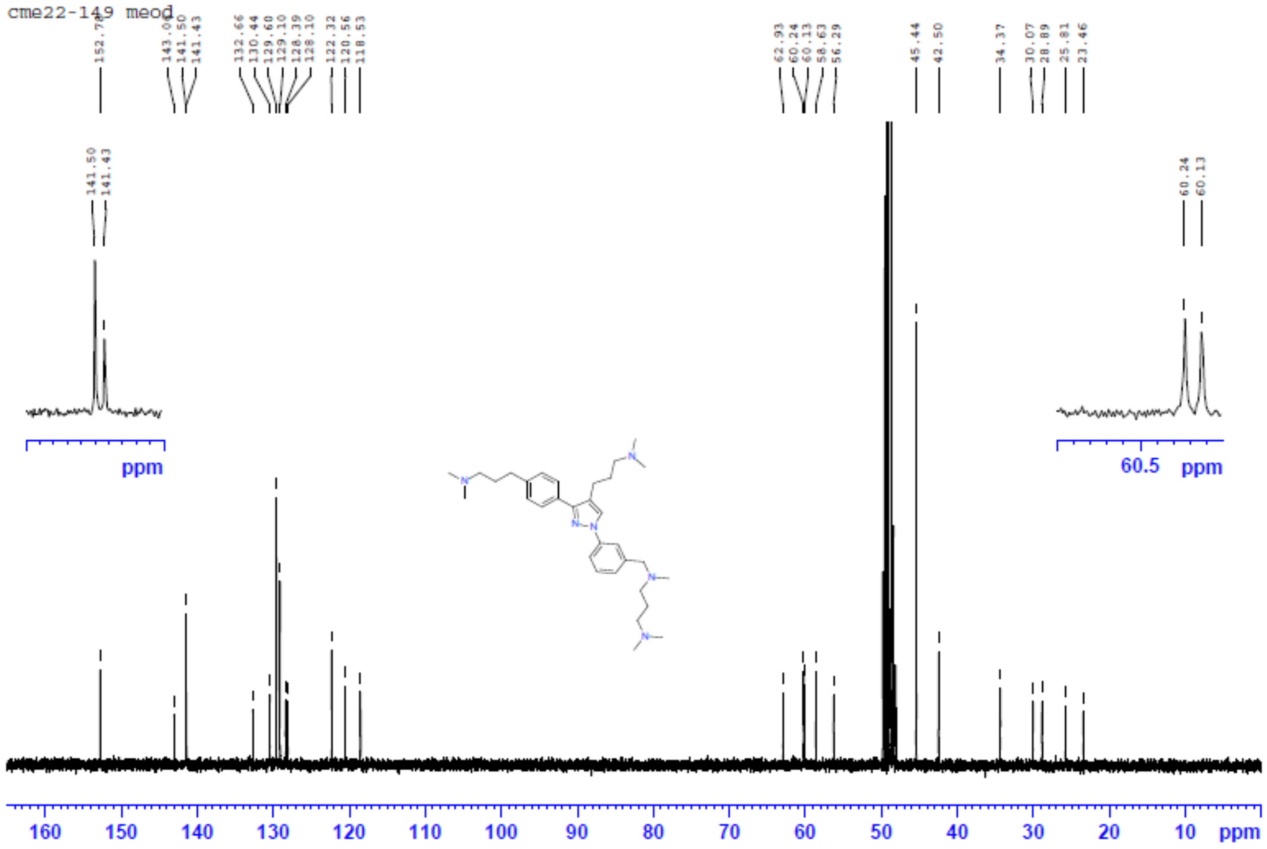

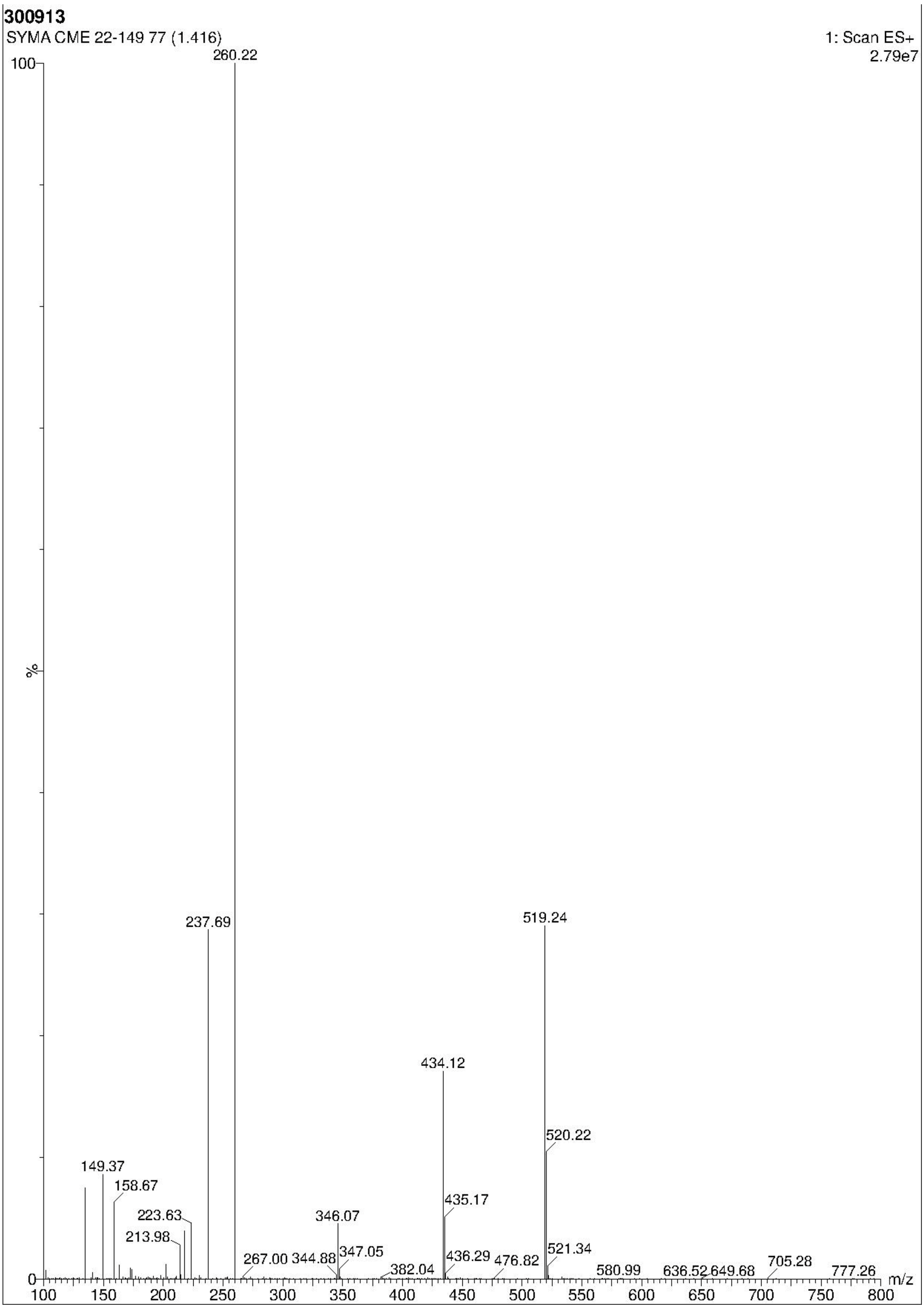

300913
SYMA CME 22-149 77 (1.416)
1: Scan ES+
2.79e7
260.22
237.69
519.24
434.12
520.22
149.37
158.67
435.17
223.63
346.07
213.98
347.05
521.34
267.00
344.88
436.29
476.82
382.04
580.99
636.52
649.68
705.28
777.26
100
%
0
m/z
100 150 200 250 300 350 400 450 500 550 600 650 700 750 800

HPLC C4-column

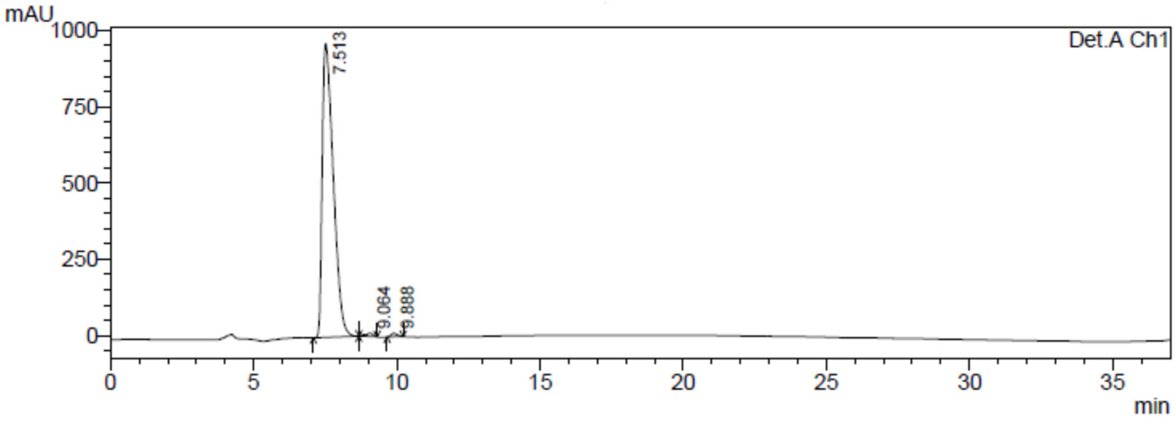

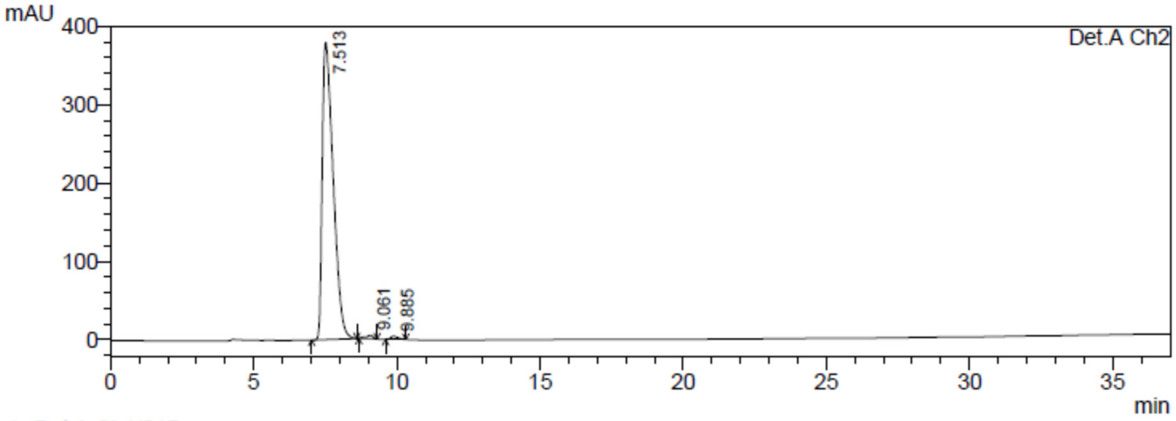

1 Det.A Ch1/215nm
2 Det.A Ch2/254nm

PeakTable

Detector A Ch1 215nm

Peak#	Ret. Time	Area	Area %
1	7.513	25836667	98.552
2	9.064	204699	0.781
3	9.888	174789	0.667
Total		26216156	100.000

PeakTable

Detector A Ch2 254nm

Peak#	Ret. Time	Area	Area %
1	7.513	10070904	98.569
2	9.061	77932	0.763
3	9.885	68270	0.668
Total		10217106	100.000

HPLC C18-column

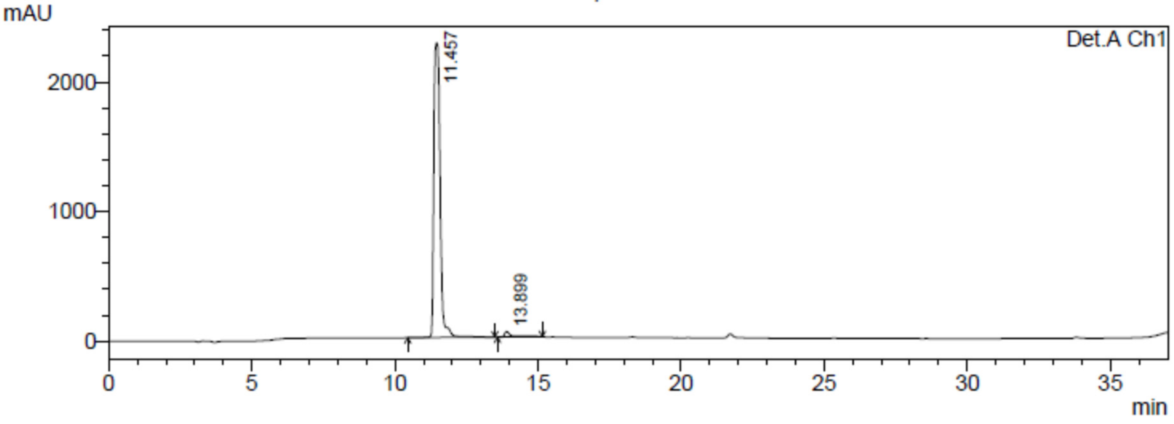

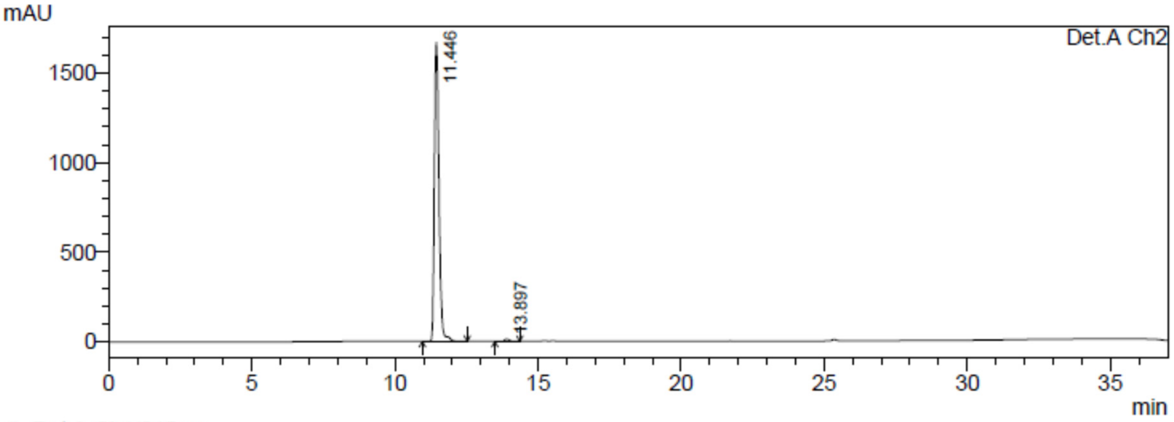

1 Det.A Ch1/215nm
2 Det.A Ch2/254nm

PeakTable

Detector A Ch1 215nm

Peak#	Ret. Time	Area	Area %
1	11.457	34721356	97.848
2	13.899	763816	2.152
Total		35485172	100.000

PeakTable

Detector A Ch2 254nm

Peak#	Ret. Time	Area	Area %
1	11.446	18639888	99.234
2	13.897	143897	0.766
Total		18783785	100.000

methyl 3-[2-[1-(4-cyanophenyl)ethylidene]hydrazino]benzoate (**38**)

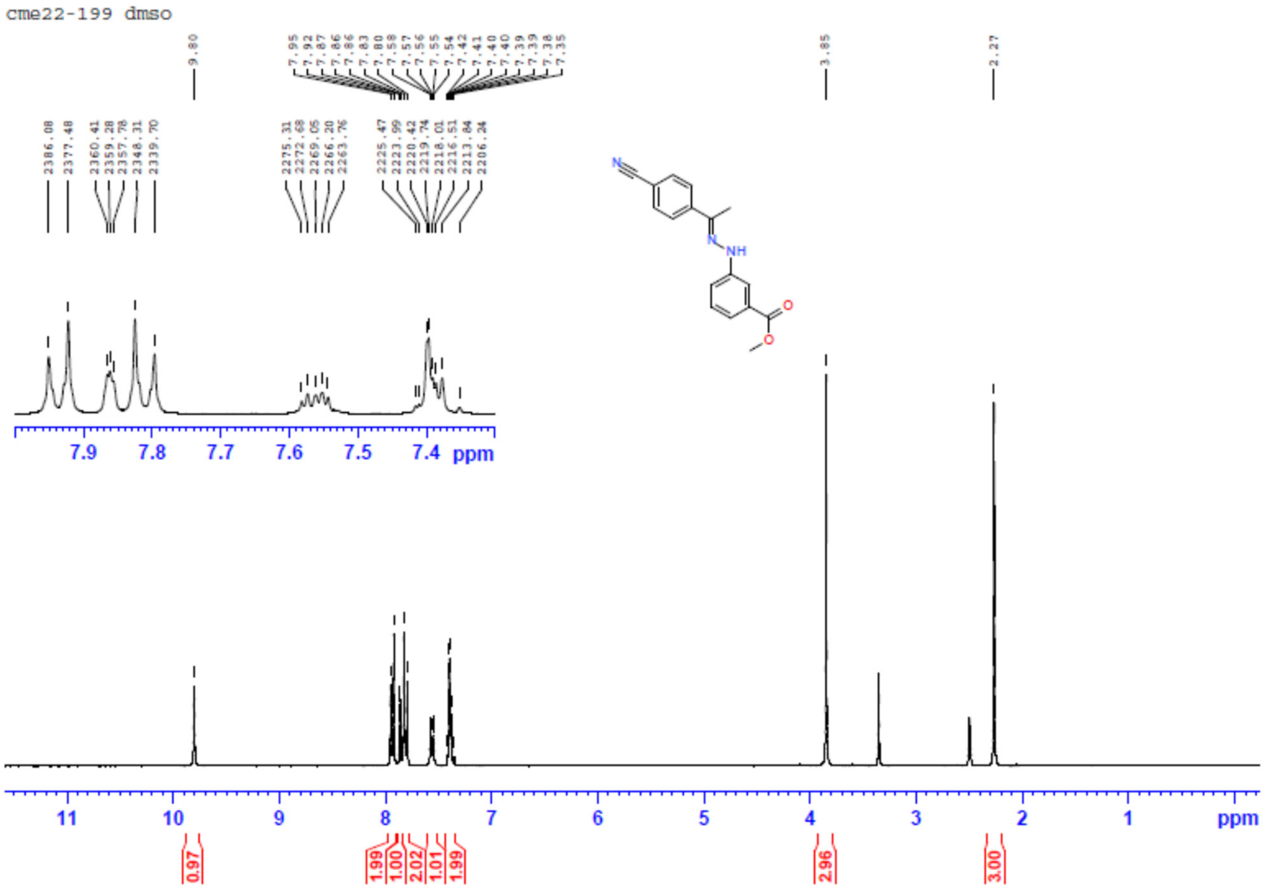

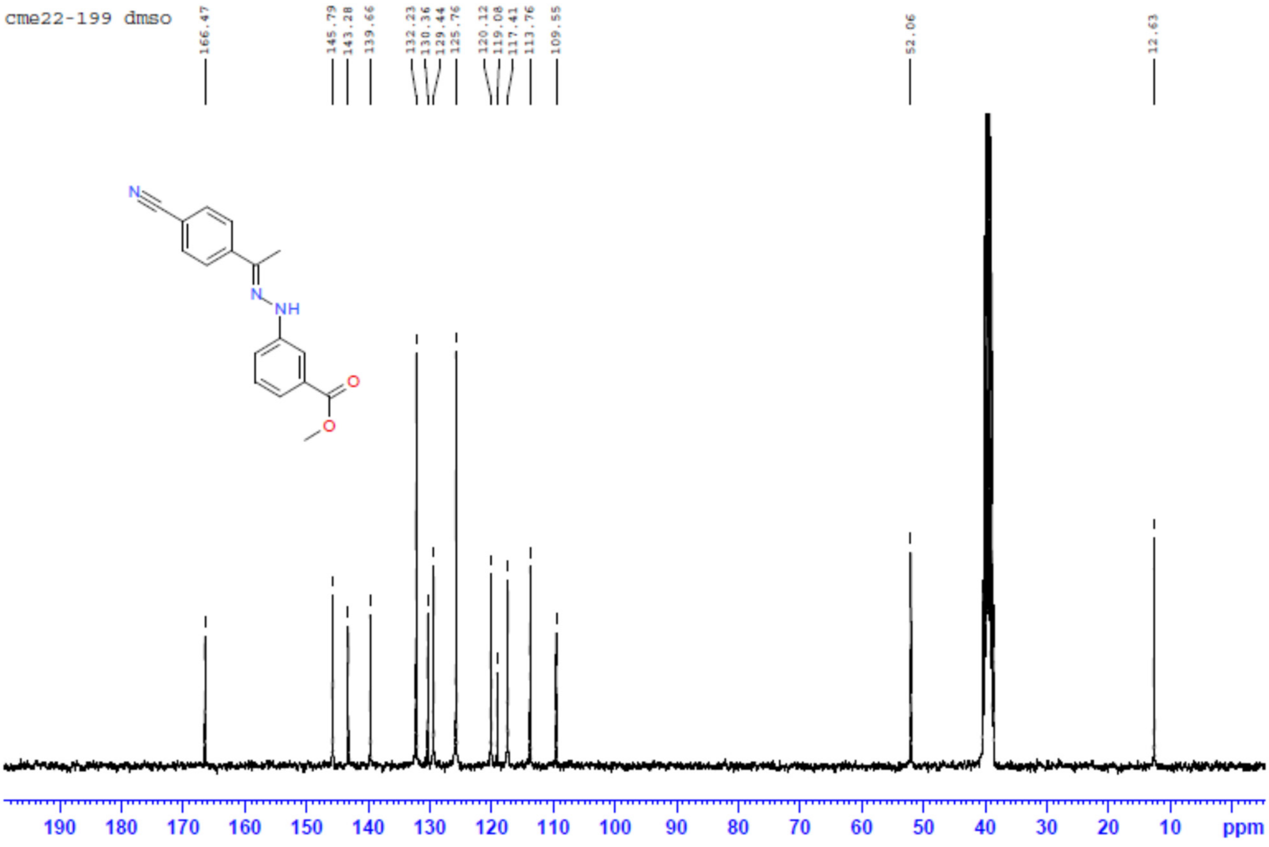

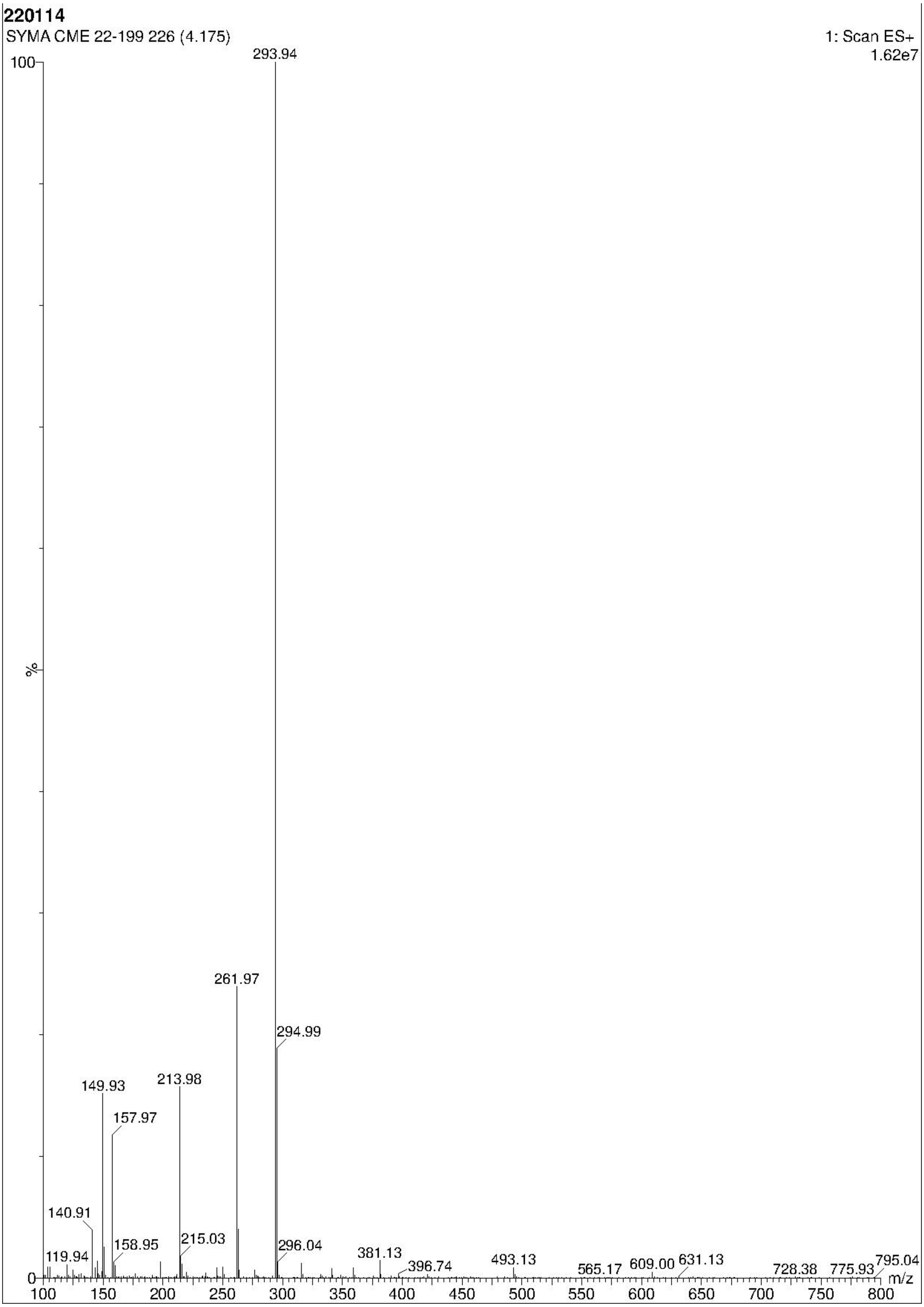

220114
SYMA CME 22-199 226 (4.175)
1: Scan ES+
1.62e7
100
%
0
293.94
261.97
294.99
149.93
213.98
157.97
140.91
119.94
158.95
215.03
296.04
381.13
396.74
493.13
565.17
609.00
631.13
728.38
775.93
795.04
m/z
100
150
200
250
300
350
400
450
500
550
600
650
700
750
800

methyl 3-[2-[1-(3-cyanophenyl)ethylidene]hydrazino]benzoate (**39**)

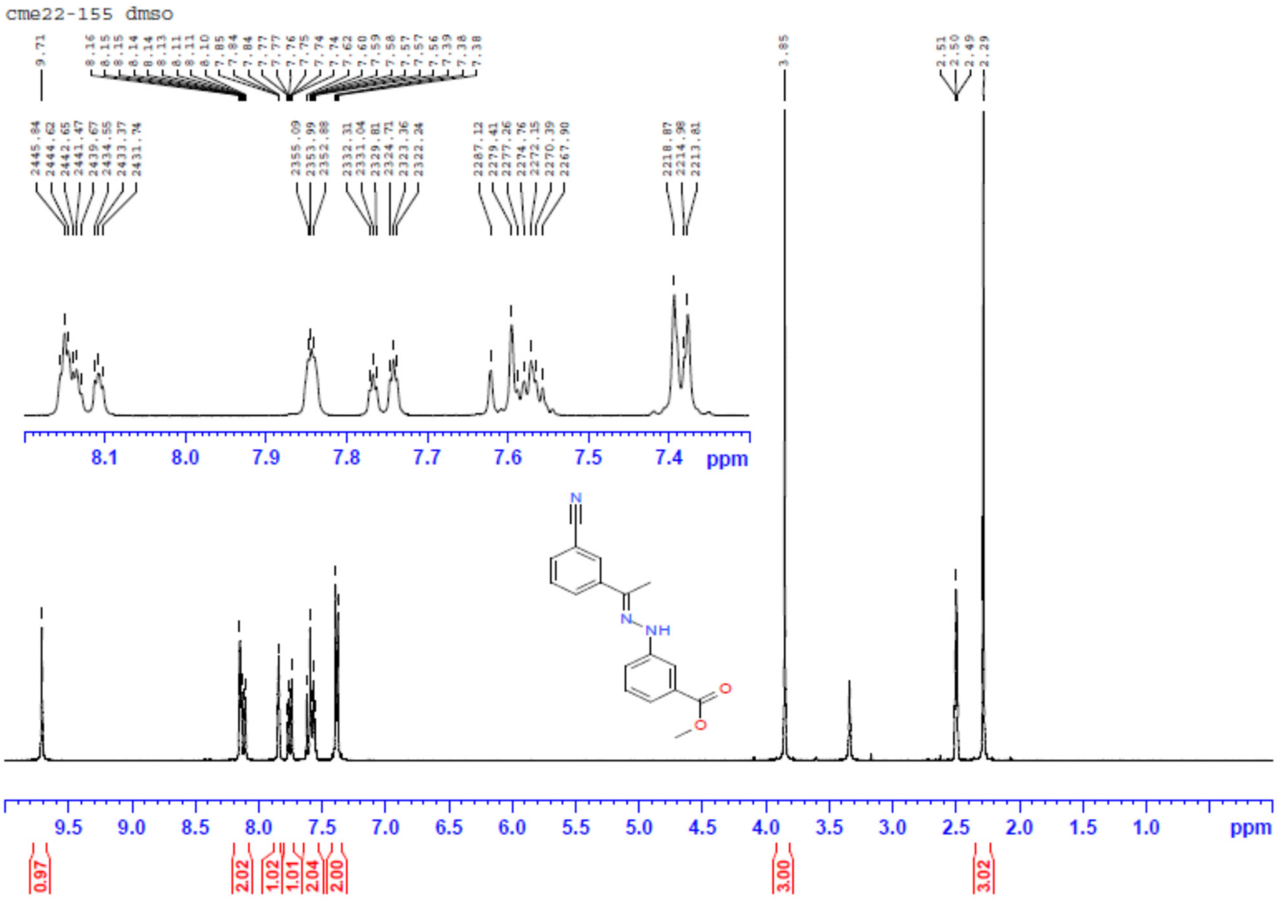

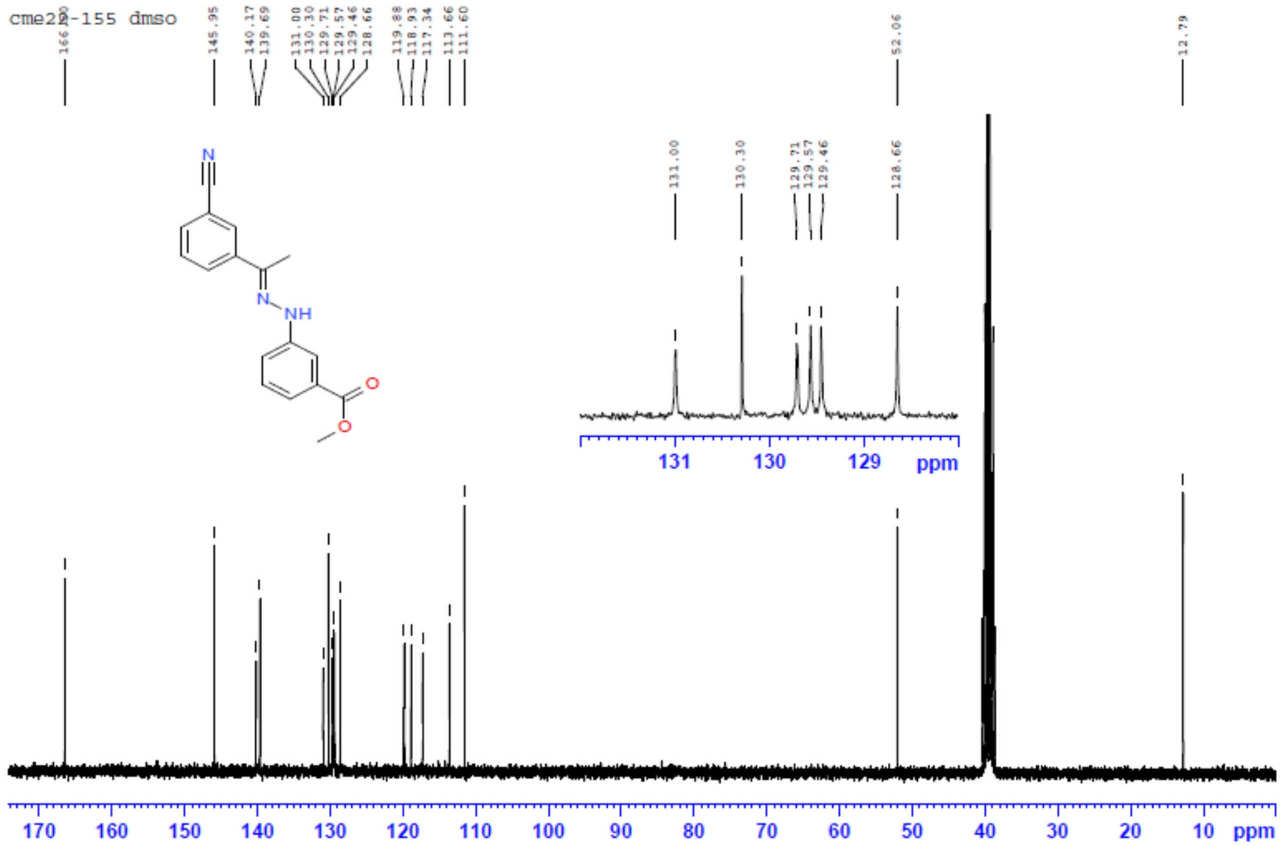

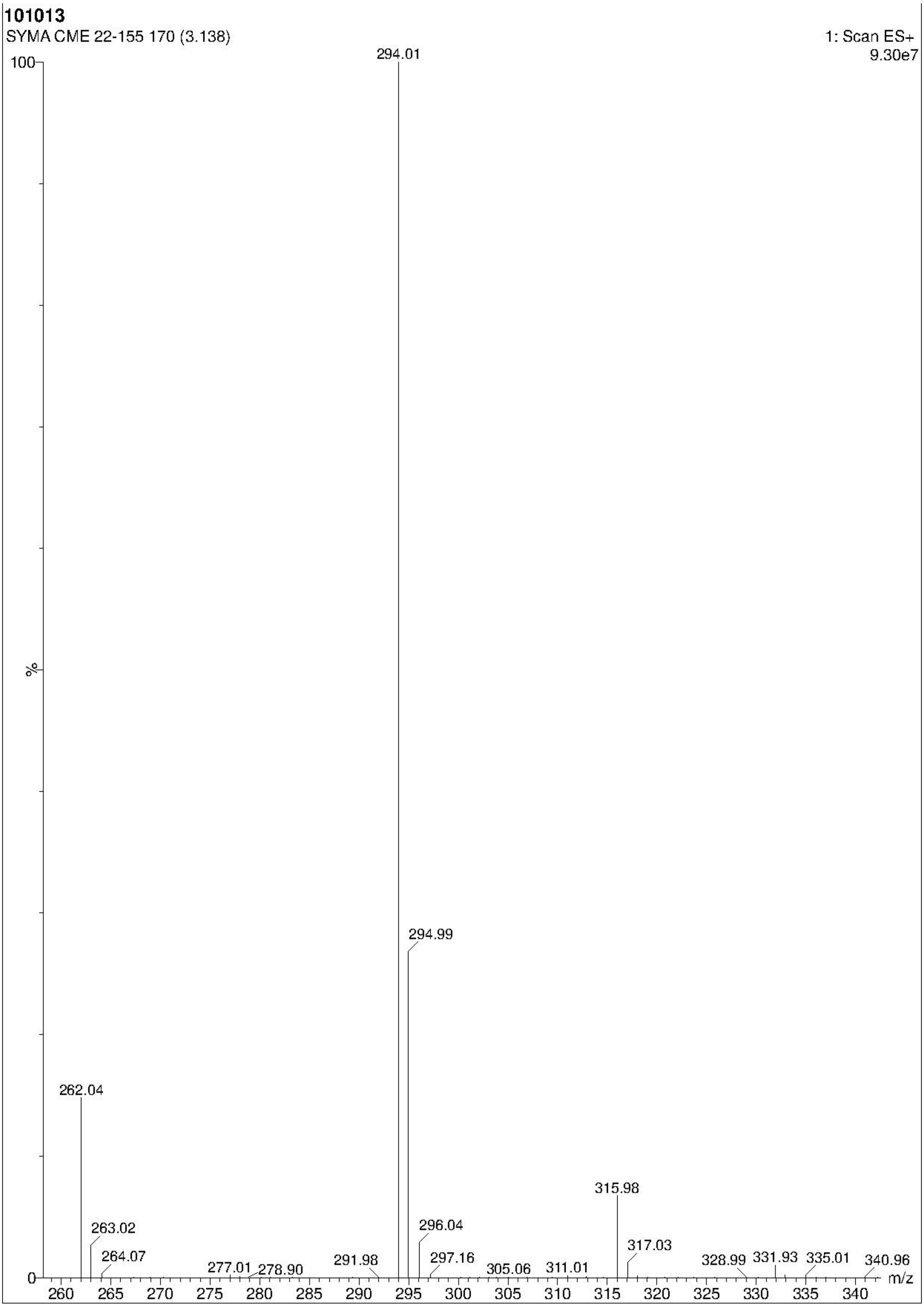
101013
SYMA CME 22-155 170 (3.138)
1: Scan ES+
9.30e7
100
%
0
294.01
294.99
262.04
263.02
264.07
277.01
278.90
291.98
296.04
297.16
305.06
311.01
315.98
317.03
328.99
331.93
335.01
340.96
260
265
270
275
280
285
290
295
300
305
310
315
320
325
330
335
340
m/z

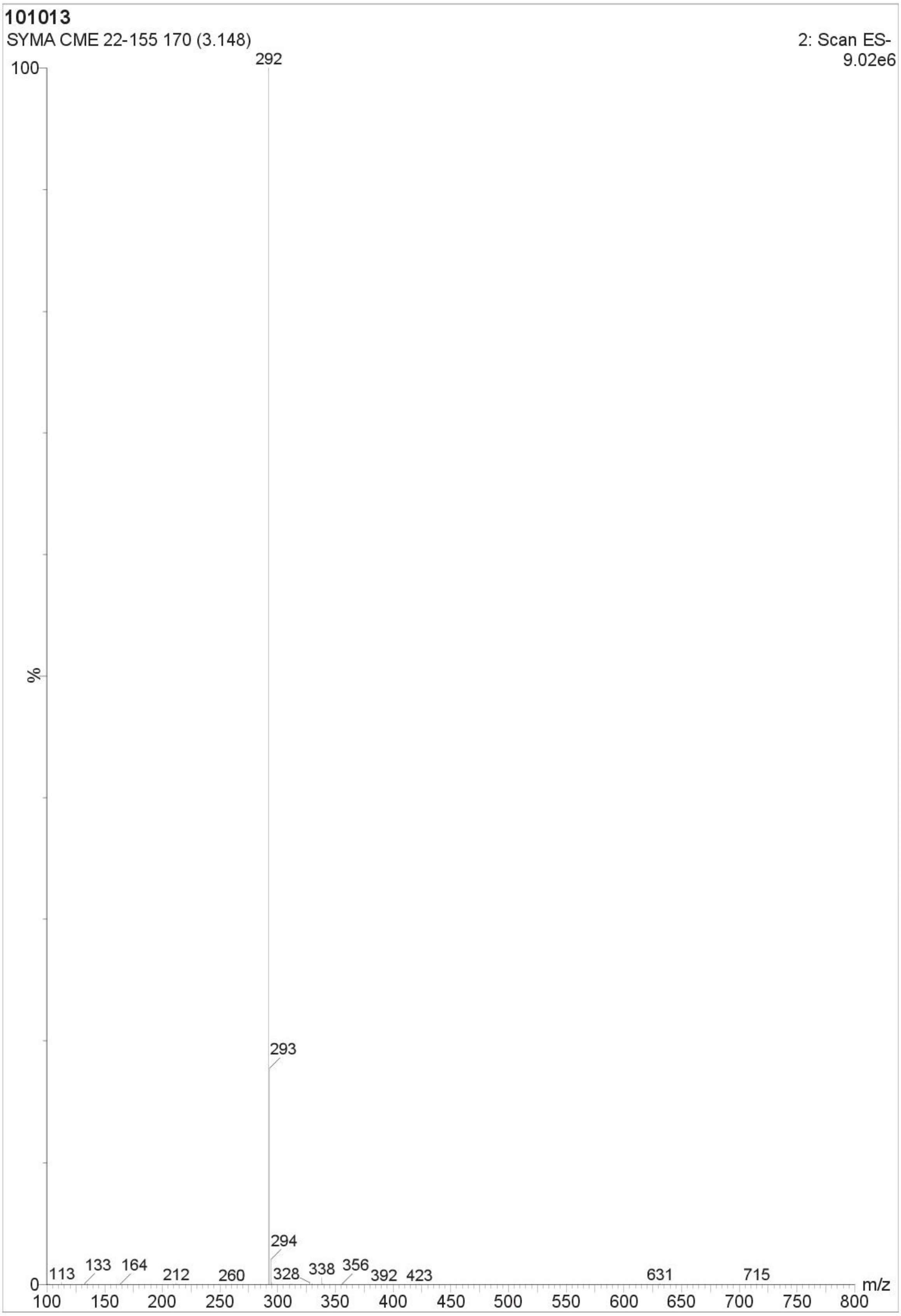

101013
SYMA CME 22-155 170 (3.148)
2: Scan ES-
9.02e6
100
%
0
292
293
294
113
133
164
212
260
328
338
356
392
423
631
715
m/z
100 150 200 250 300 350 400 450 500 550 600 650 700 750 800

methyl 3-[3-(4-cyanophenyl)-4-formyl-pyrazol-1-yl]benzoate (**40**)

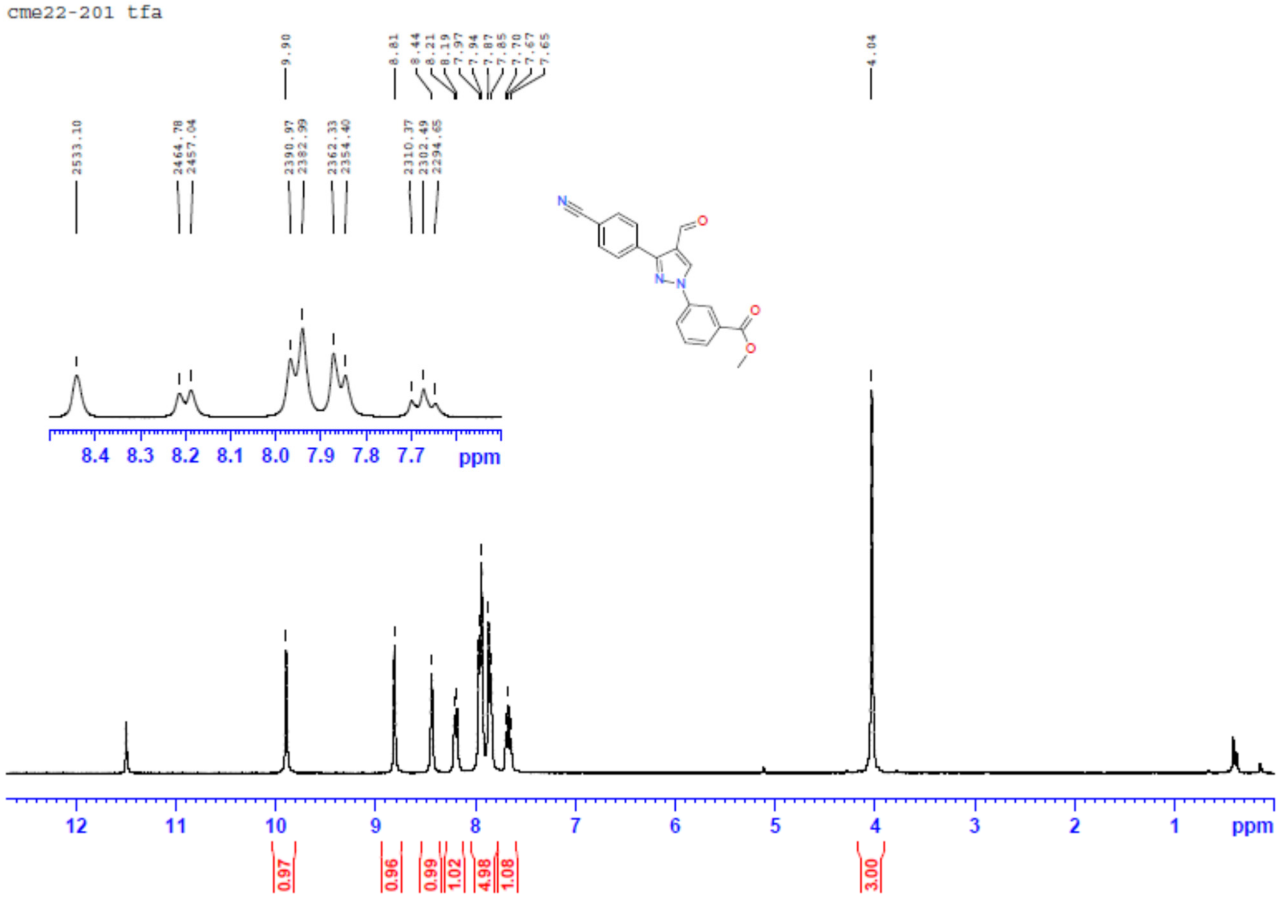

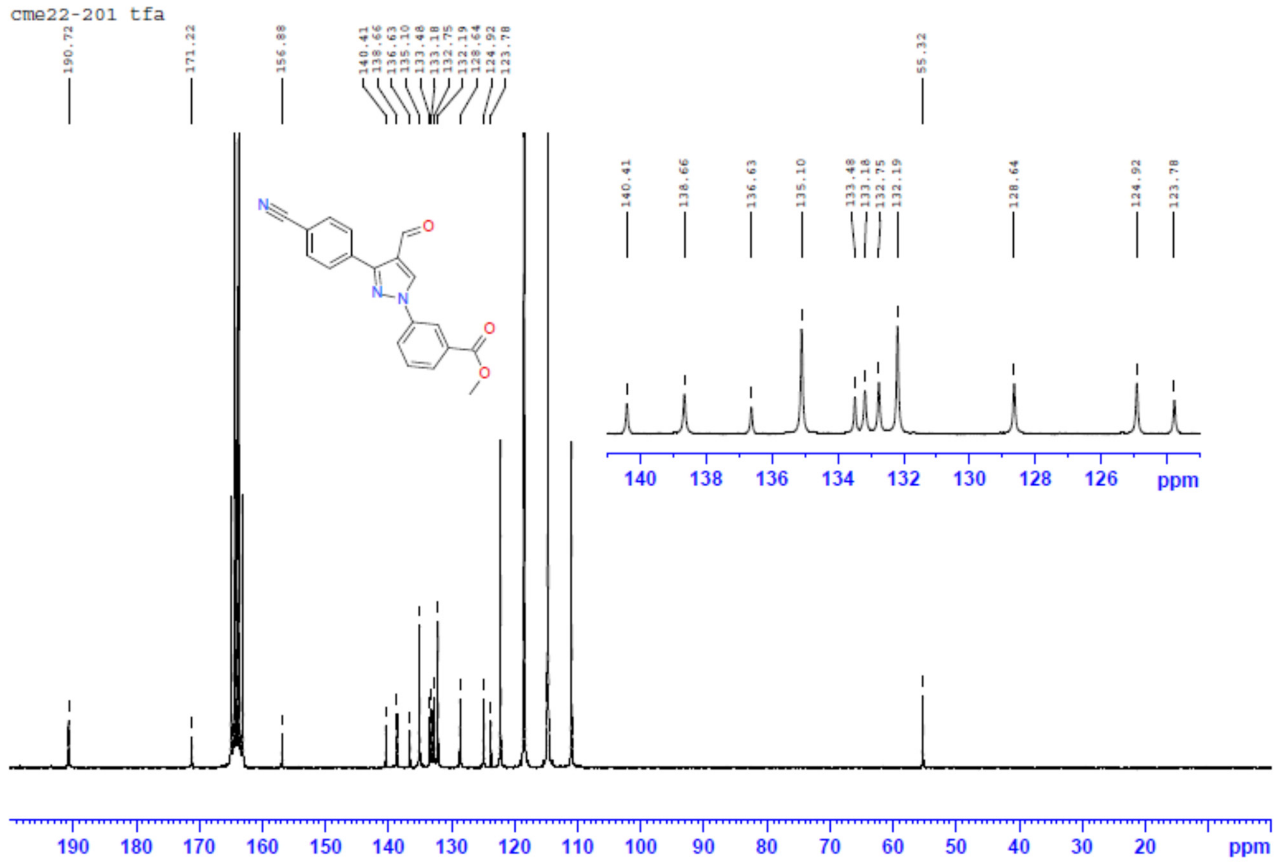

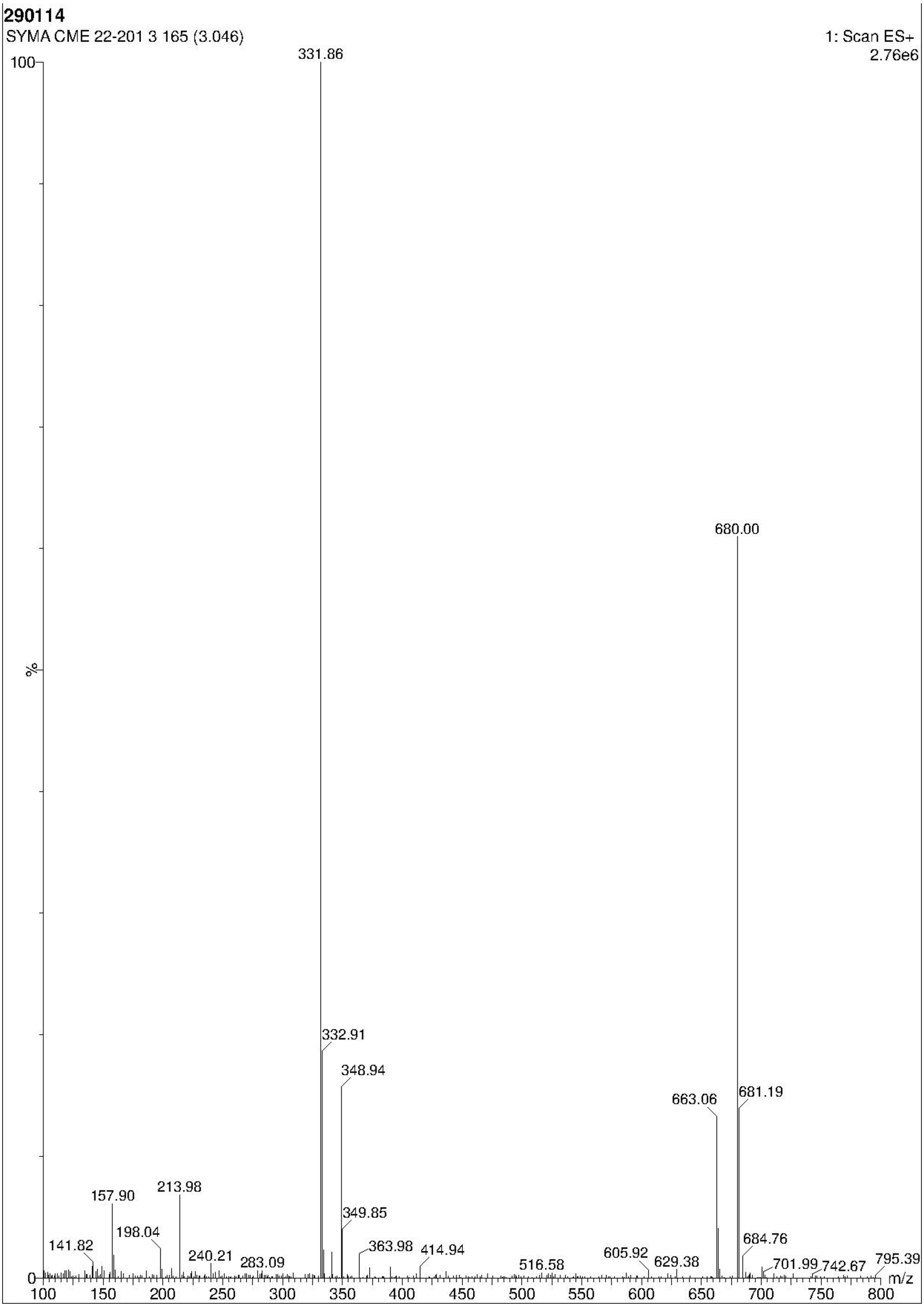
290114
SYMA CME 22-201 3 165 (3.046)
1: Scan ES+
2.76e6
331.86
680.00
332.91
348.94
663.06
681.19
157.90
213.98
349.85
198.04
141.82
240.21
283.09
363.98
414.94
516.58
605.92
629.38
684.76
701.99
742.67
795.39
%
m/z
100
0
100 150 200 250 300 350 400 450 500 550 600 650 700 750 800

methyl 3-[3-(3-cyanophenyl)-4-formyl-pyrazol-1-yl]benzoate (**41**)

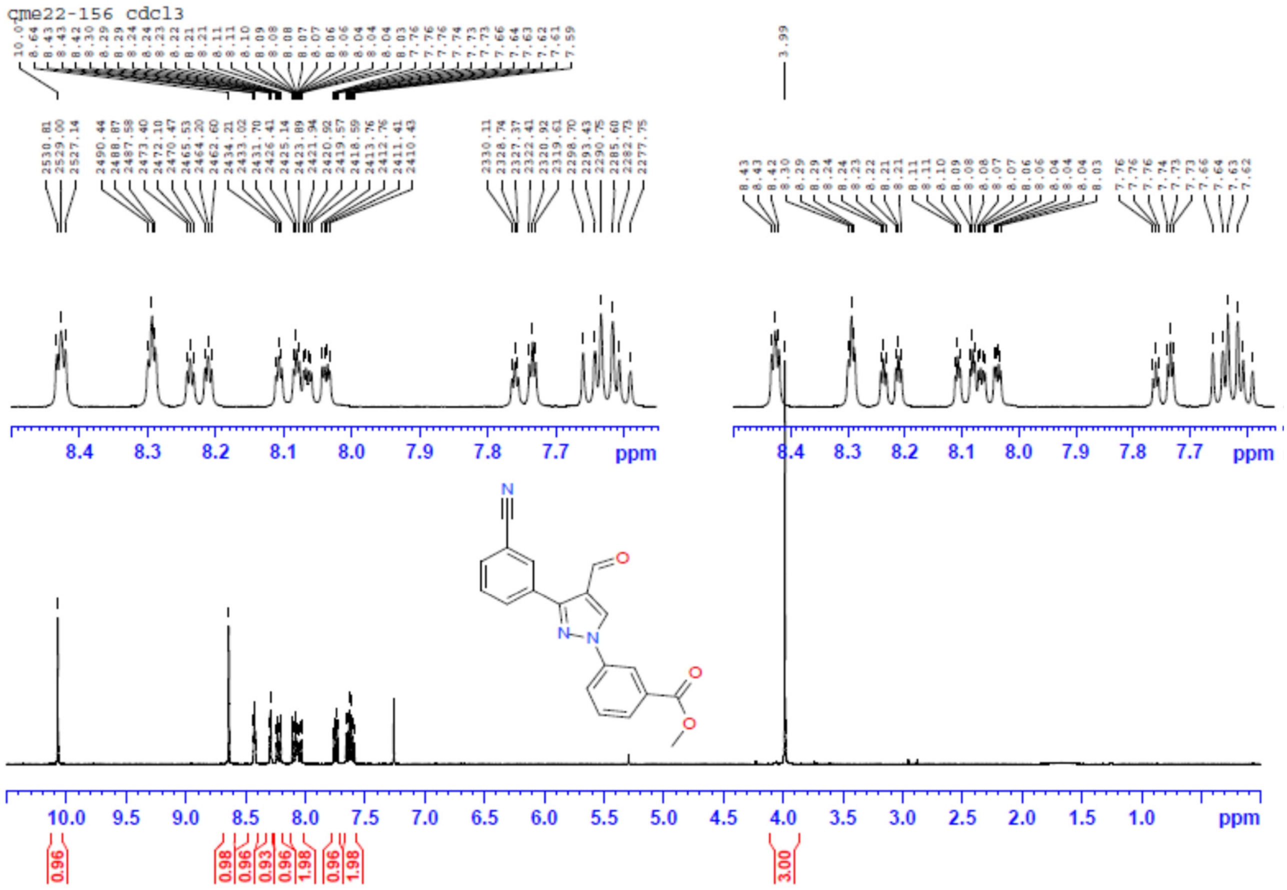

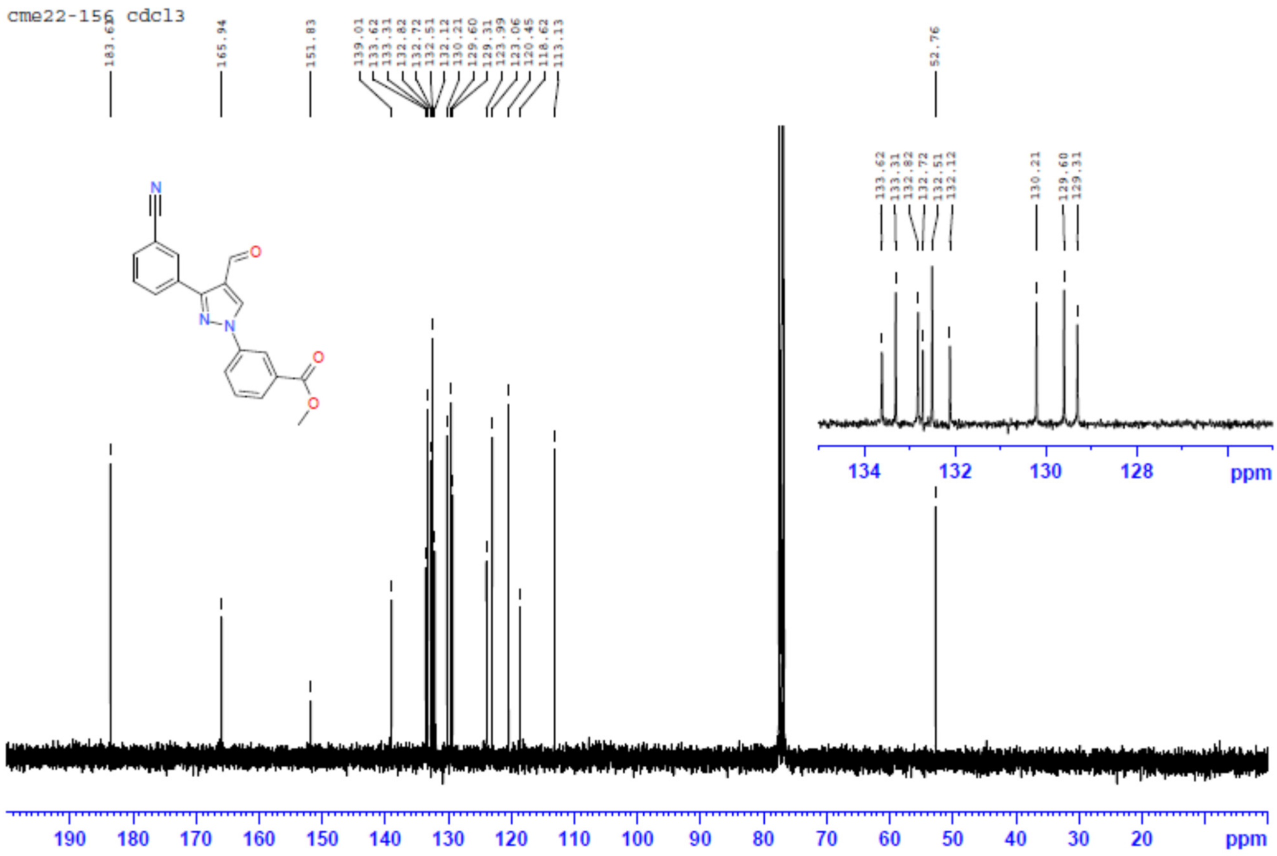

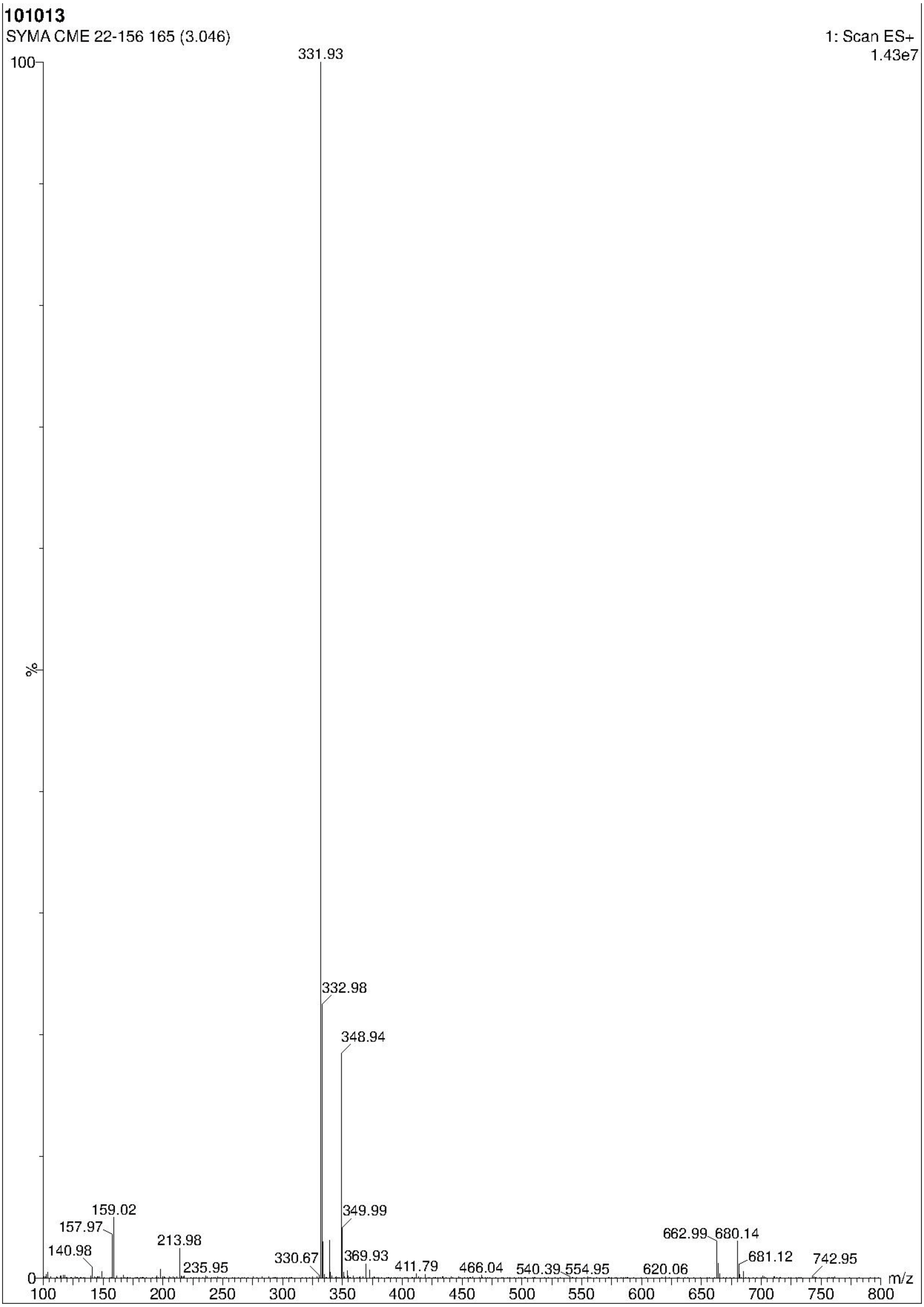

101013
SYMA CME 22-156 165 (3.046)
1: Scan ES+
1.43e7
331.93
332.98
348.94
349.99
159.02
157.97
140.98
213.98
235.95
330.67
369.93
411.79
466.04
540.39
554.95
620.06
662.99
680.14
681.12
742.95
%
m/z
100
0
100
150
200
250
300
350
400
450
500
550
600
650
700
750
800

methyl 3-[3-(4-cyanophenyl)-4-methyl-pyrazol-1-yl]benzoate (**42**)

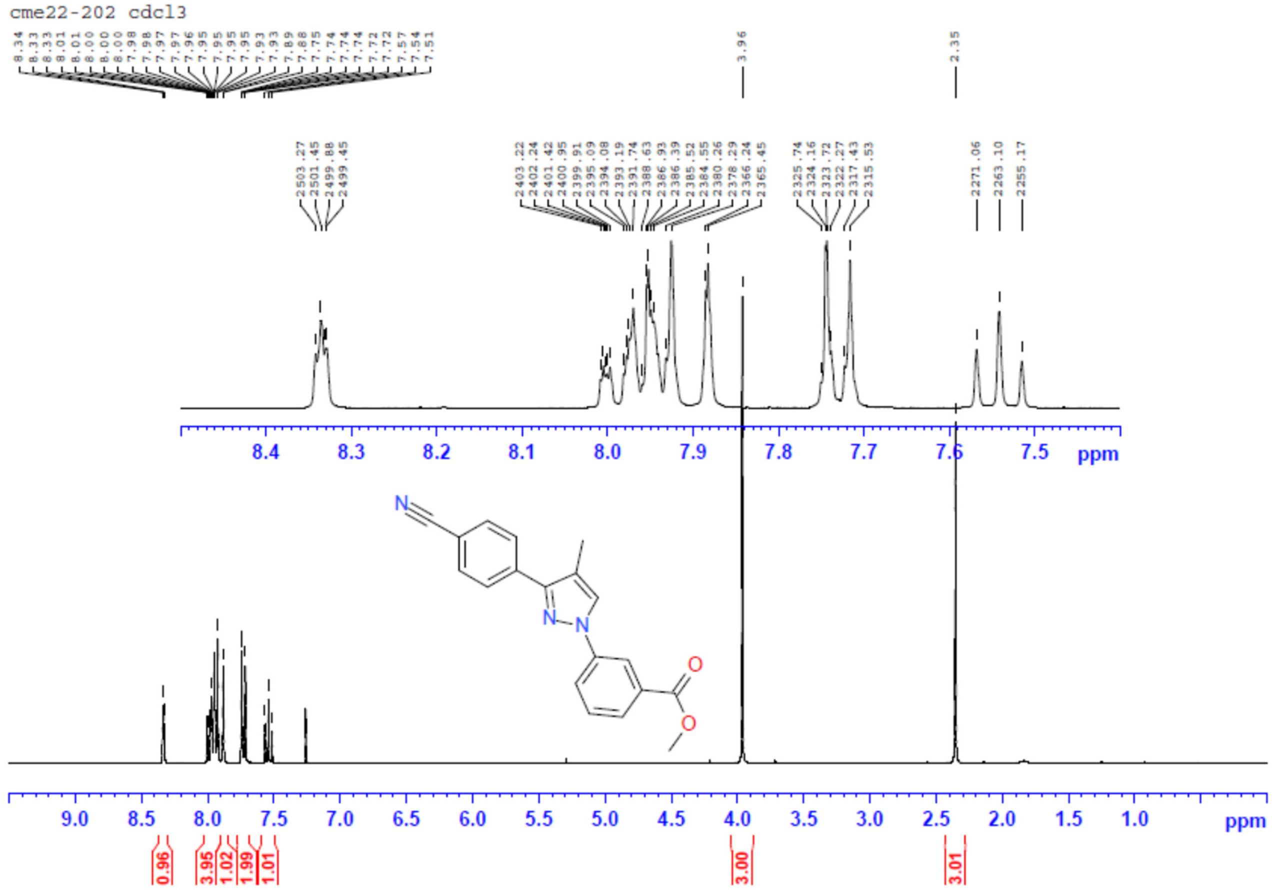

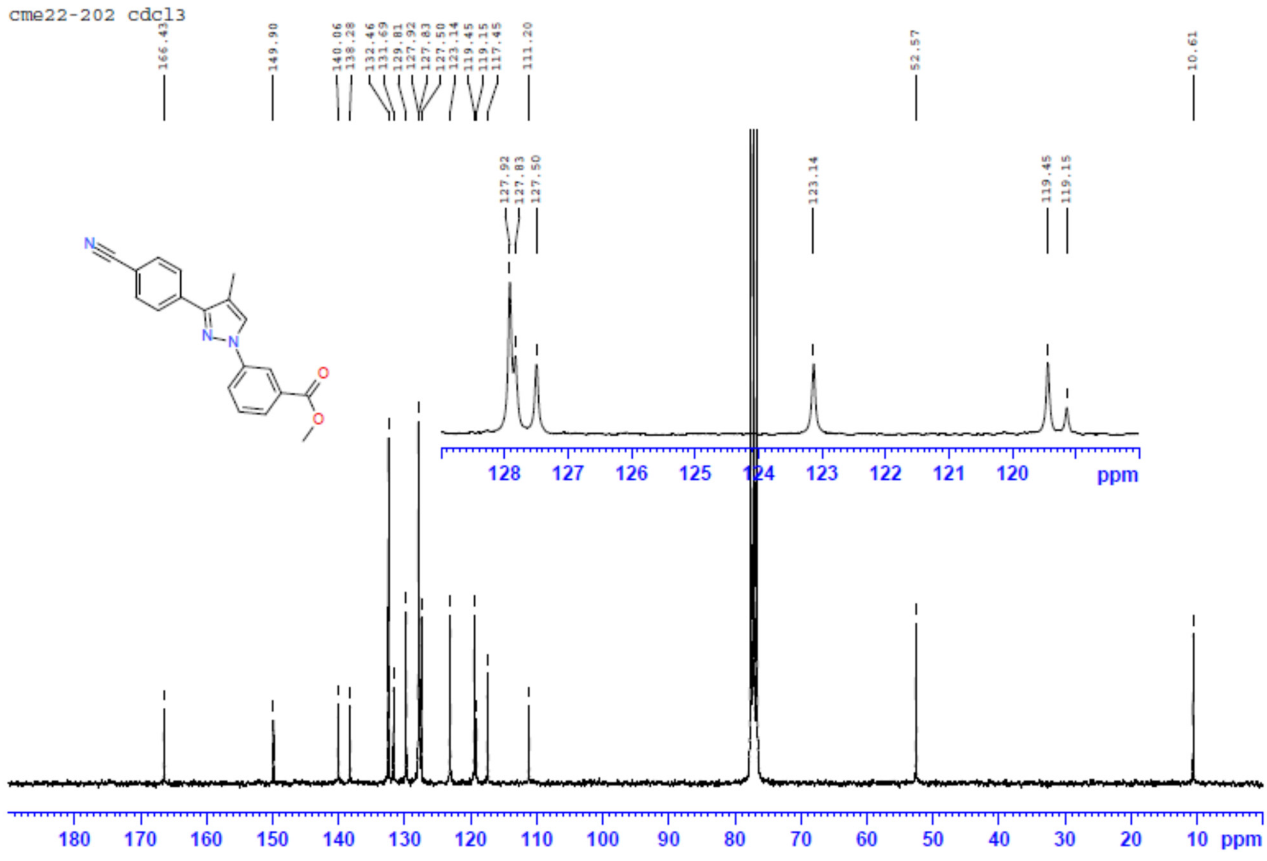

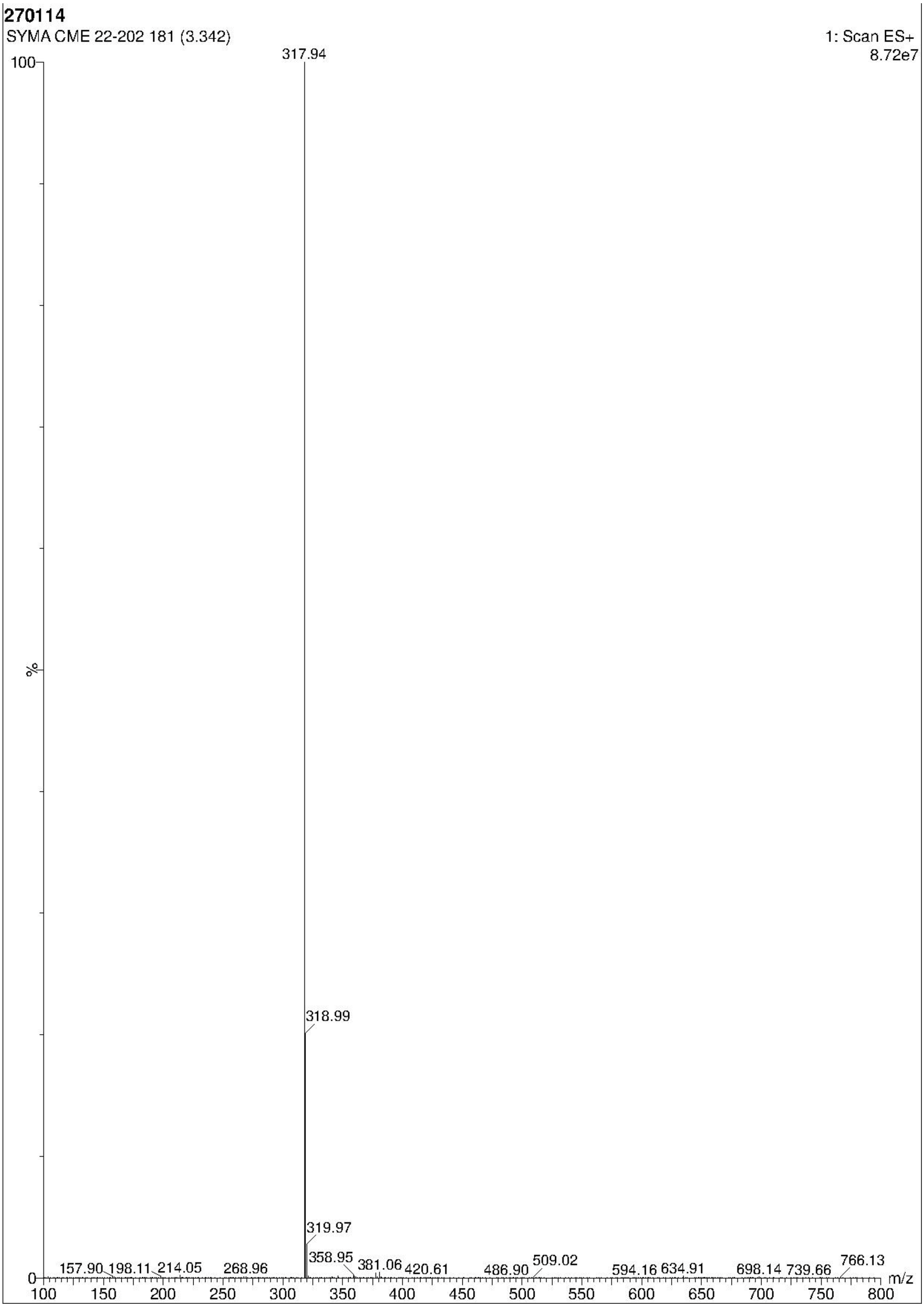
270114
SYMA CME 22-202 181 (3.342)
1: Scan ES+
8.72e7
100
%
0
317.94
318.99
319.97
358.95
381.06
420.61
486.90
509.02
594.16
634.91
698.14
739.66
766.13
157.90
198.11
214.05
268.96
m/z
100
150
200
250
300
350
400
450
500
550
600
650
700
750
800

methyl 3-[3-(3-cyanophenyl)-4-methyl-pyrazol-1-yl]benzoate (**43**)

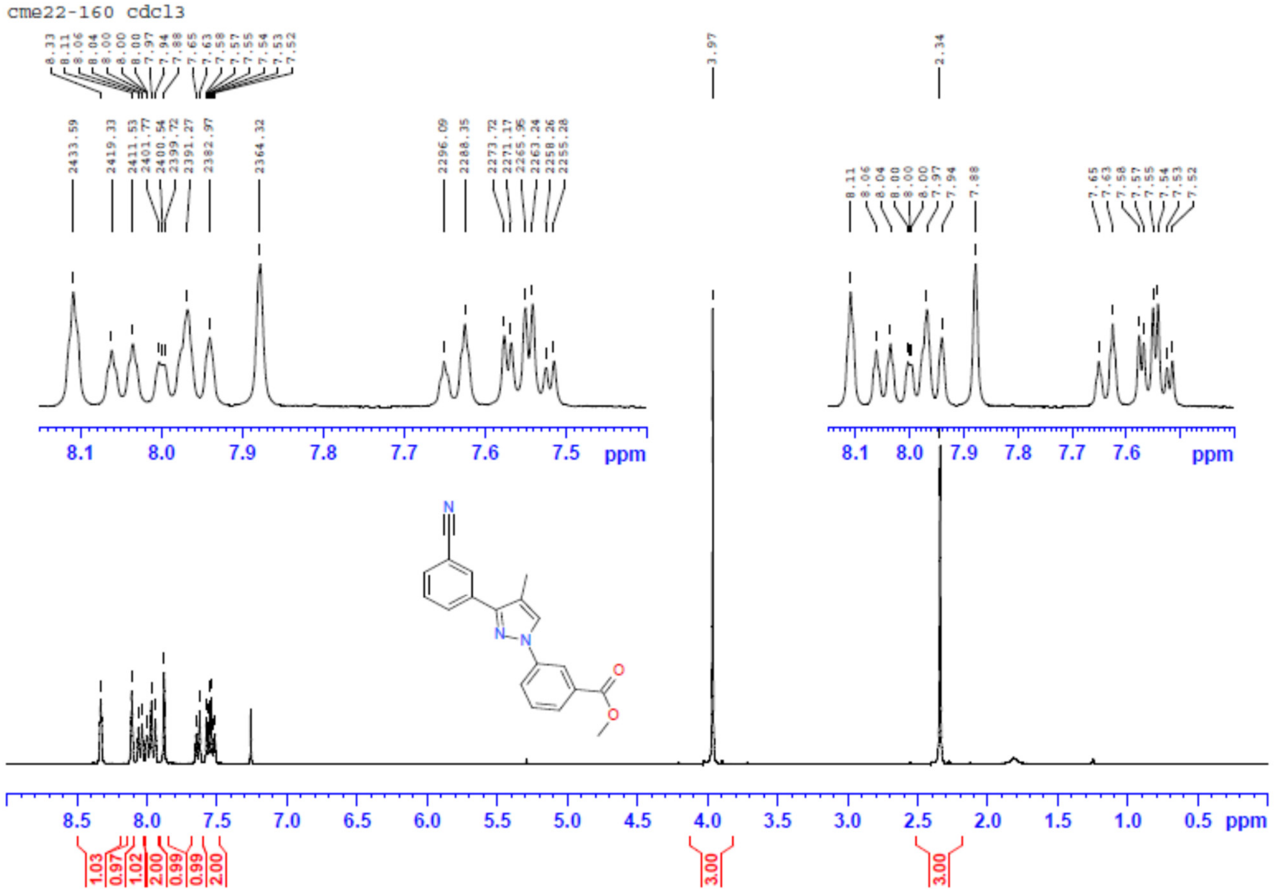

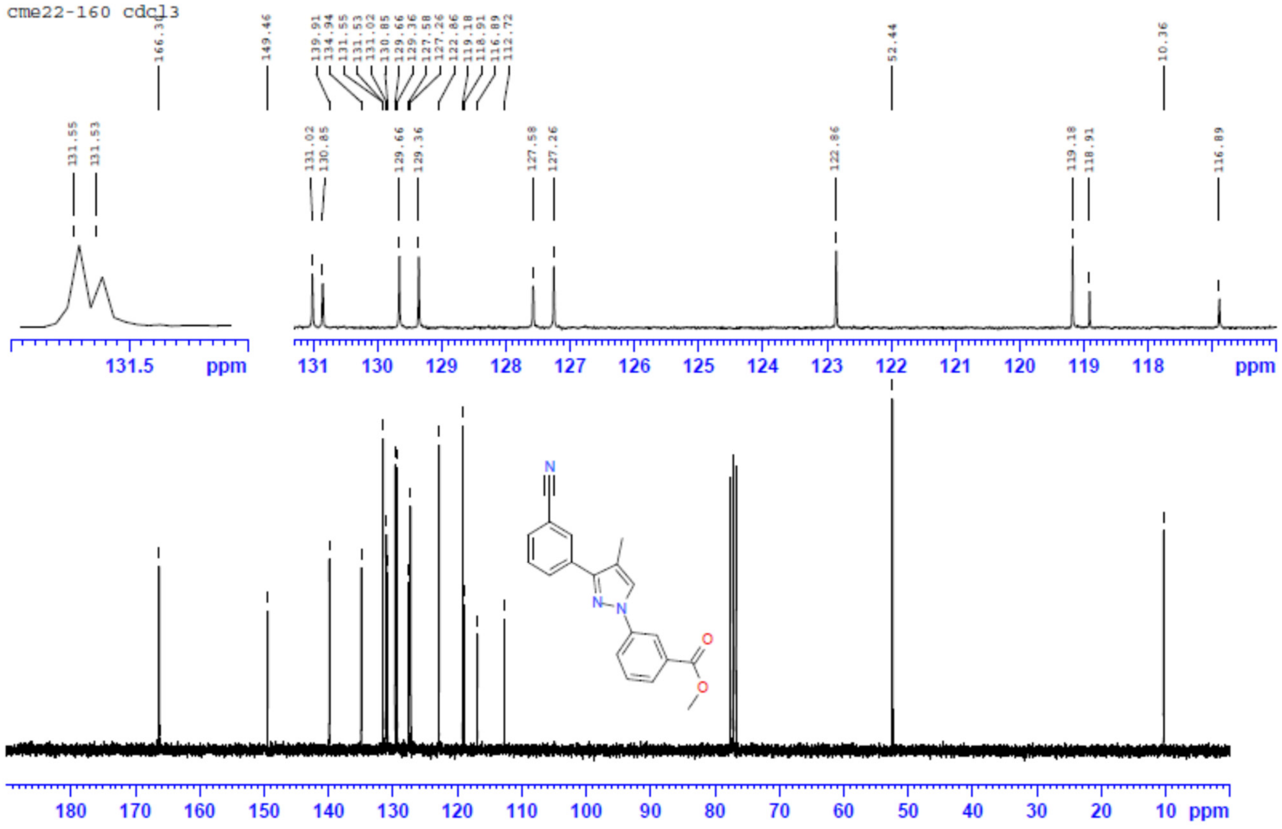

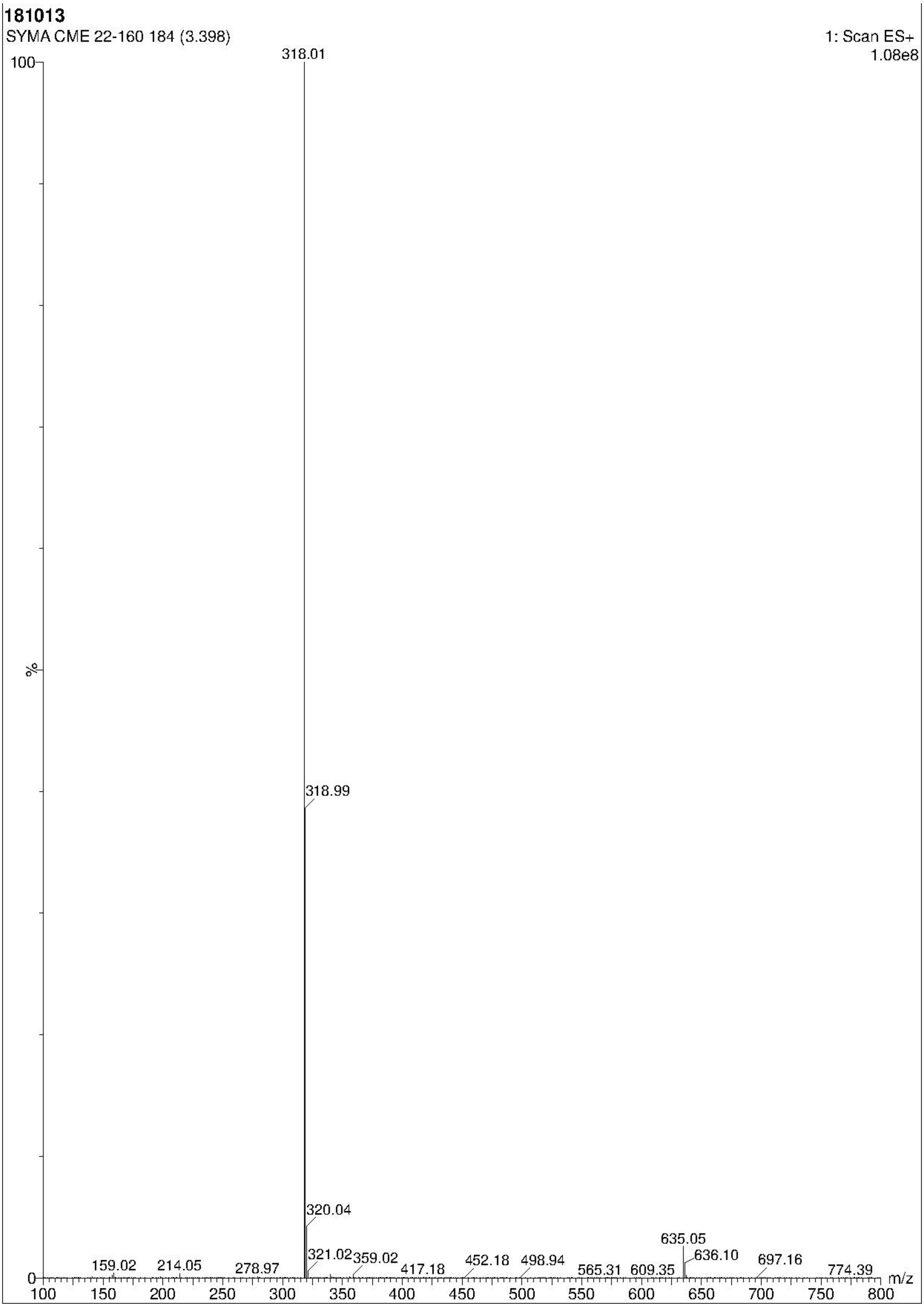
181013
SYMA CME 22-160 184 (3.398)
1: Scan ES+
1.08e8
100
%
0
318.01
318.99
320.04
321.02
359.02
159.02
214.05
278.97
417.18
452.18
498.94
565.31
609.35
635.05
636.10
697.16
774.39
m/z
100 150 200 250 300 350 400 450 500 550 600 650 700 750 800

methyl 3-[3-[4-(aminomethyl)phenyl]-4-methyl-pyrazol-1-yl]benzoate (**44**)

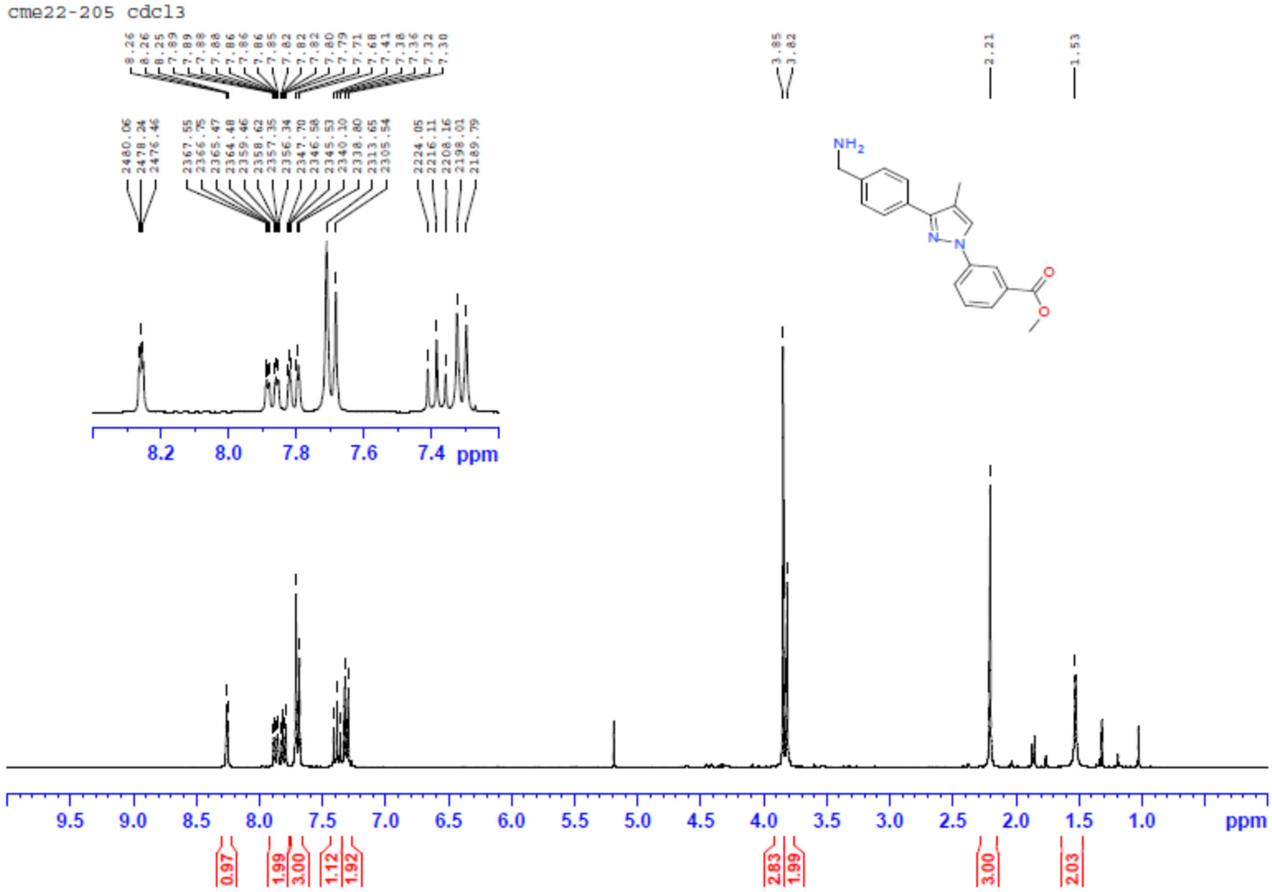

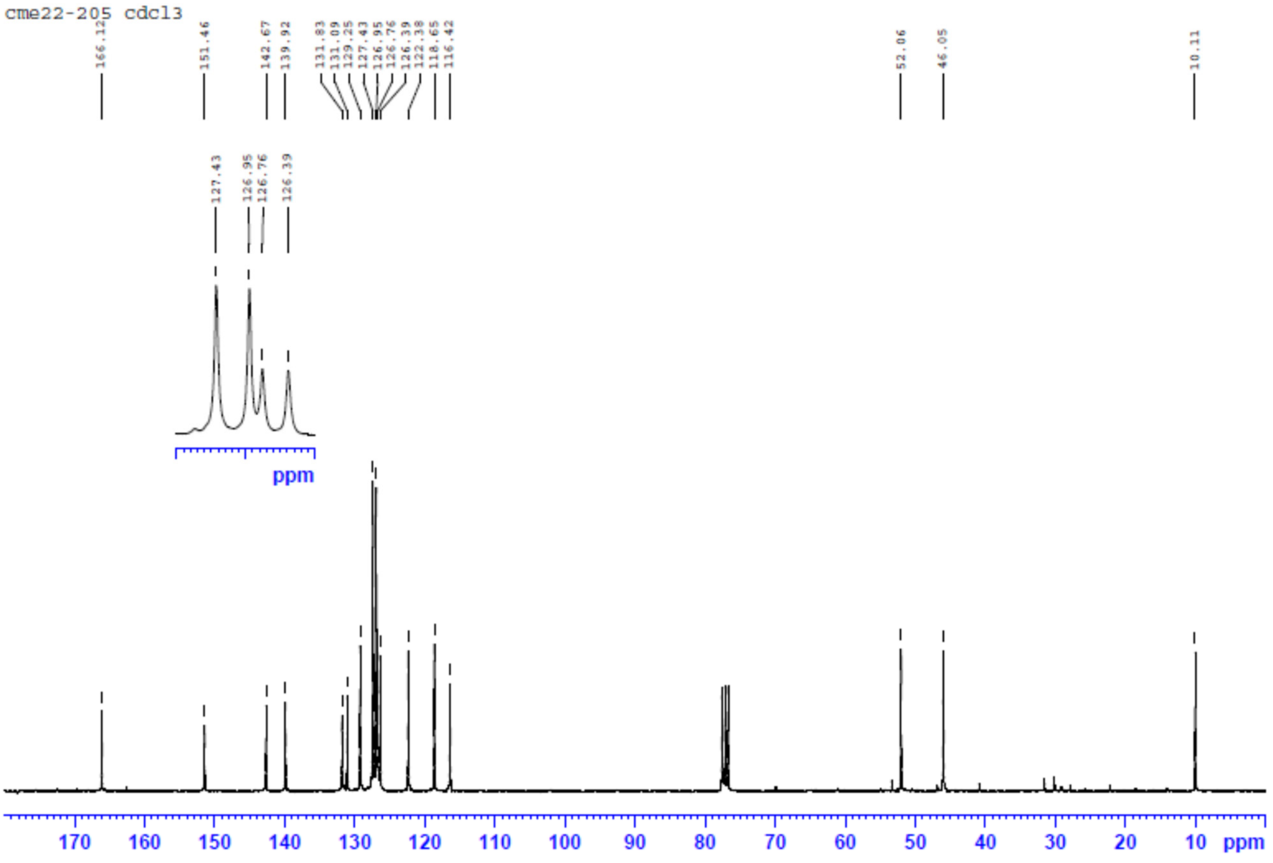

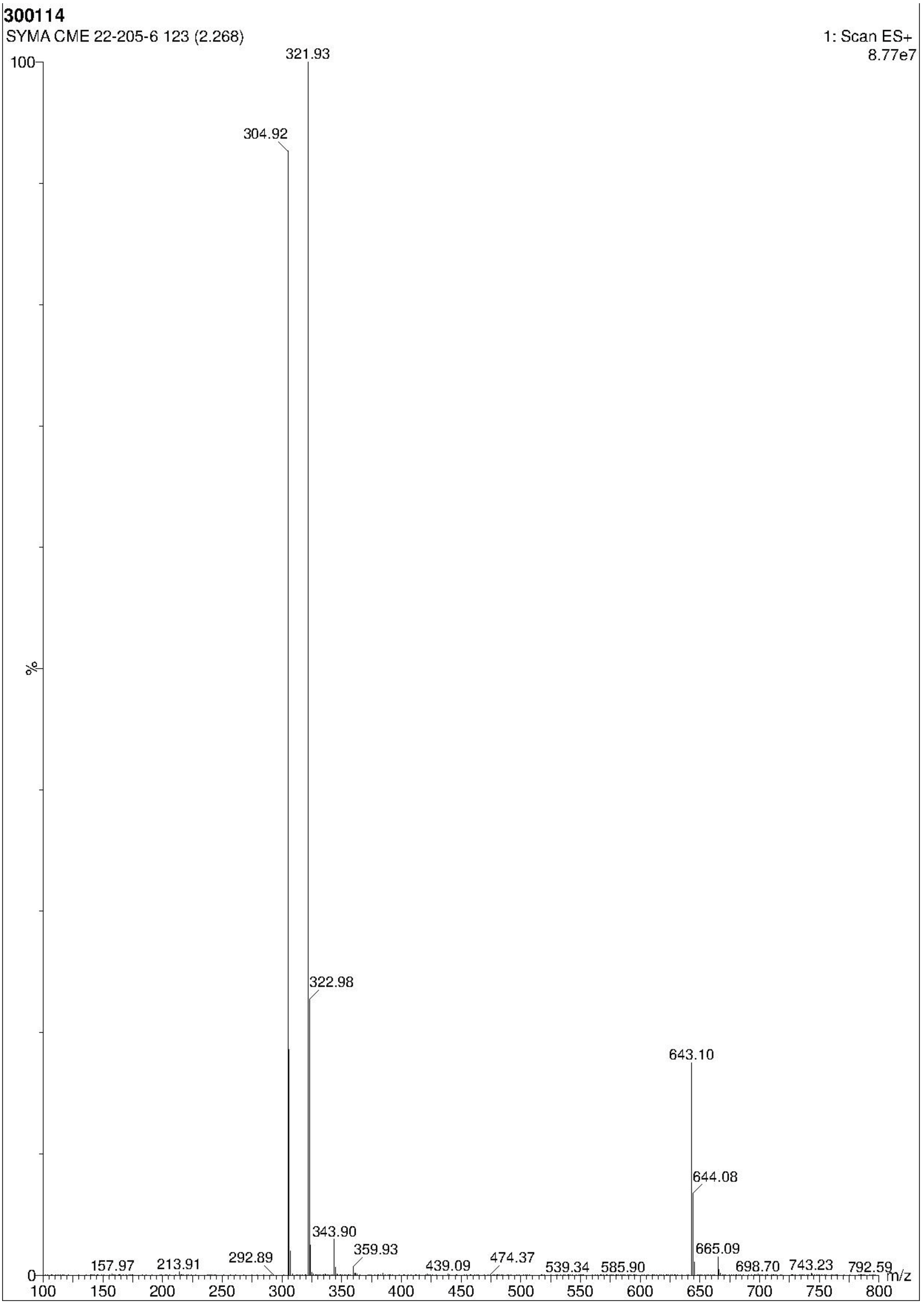
300114
SYMA CME 22-205-6 123 (2.268)
1: Scan ES+
8.77e7
321.93
304.92
322.98
643.10
644.08
343.90
359.93
665.09
157.97
213.91
292.89
439.09
474.37
539.34
585.90
698.70
743.23
792.59
%
m/z
100
0
100
150
200
250
300
350
400
450
500
550
600
650
700
750
800

methyl 3-[3-[3-(aminomethyl)phenyl]-4-methyl-pyrazol-1-yl]benzoate (**45**)

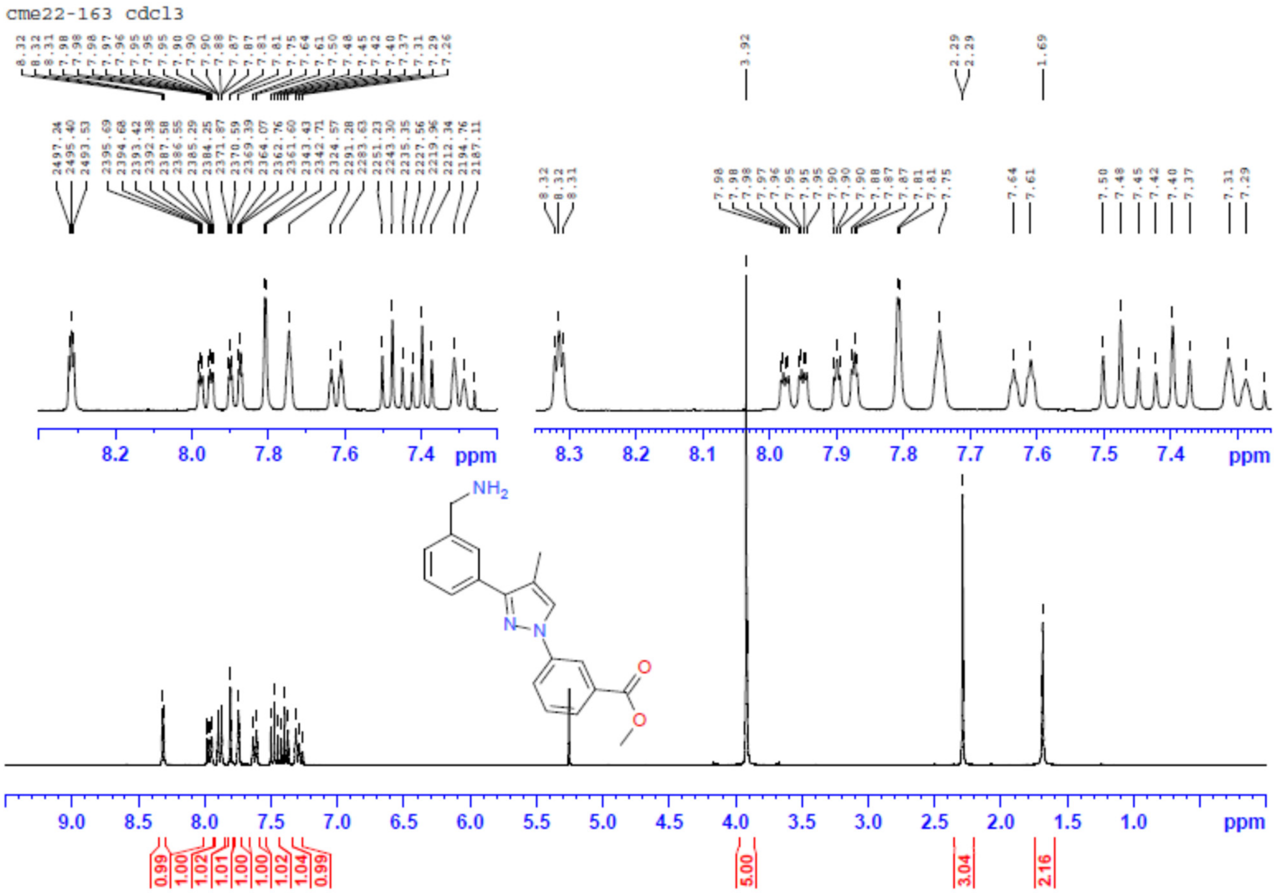

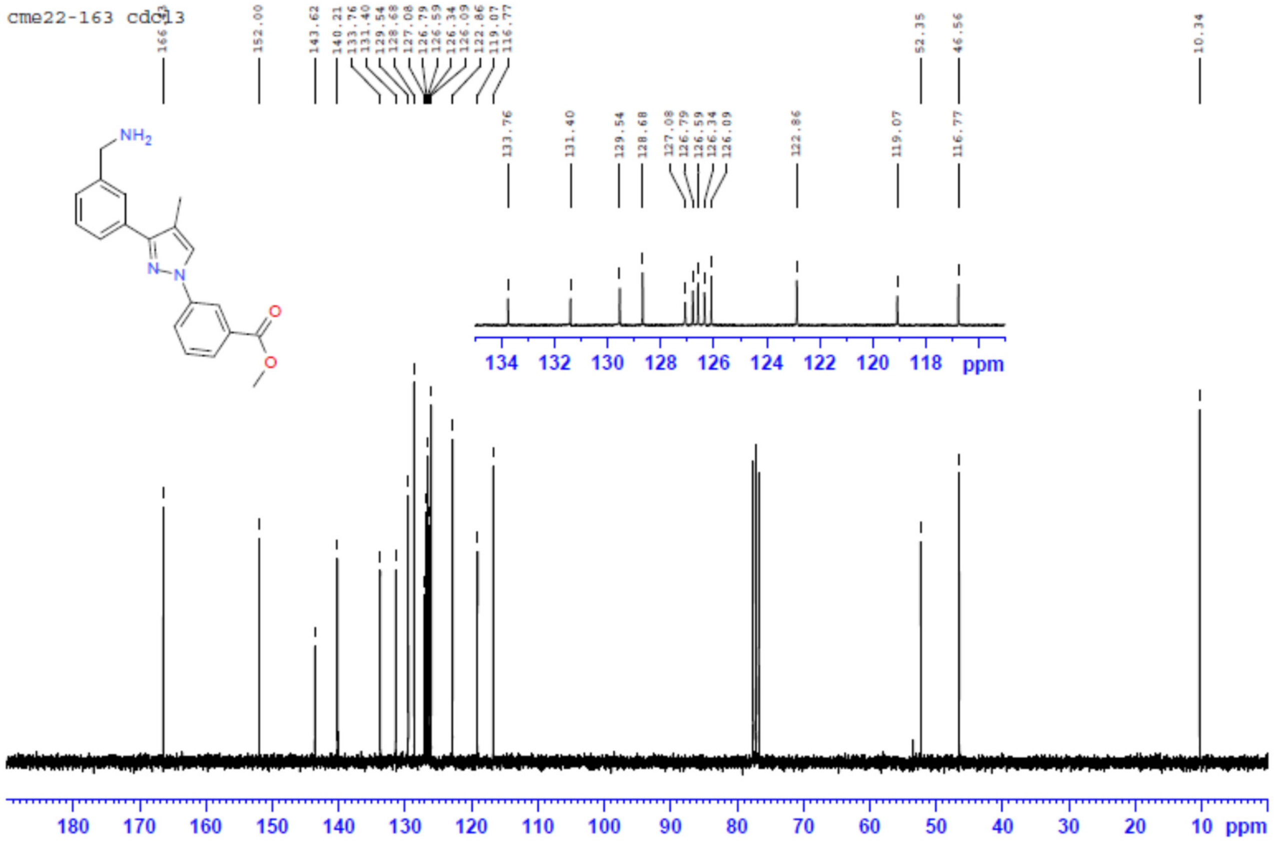

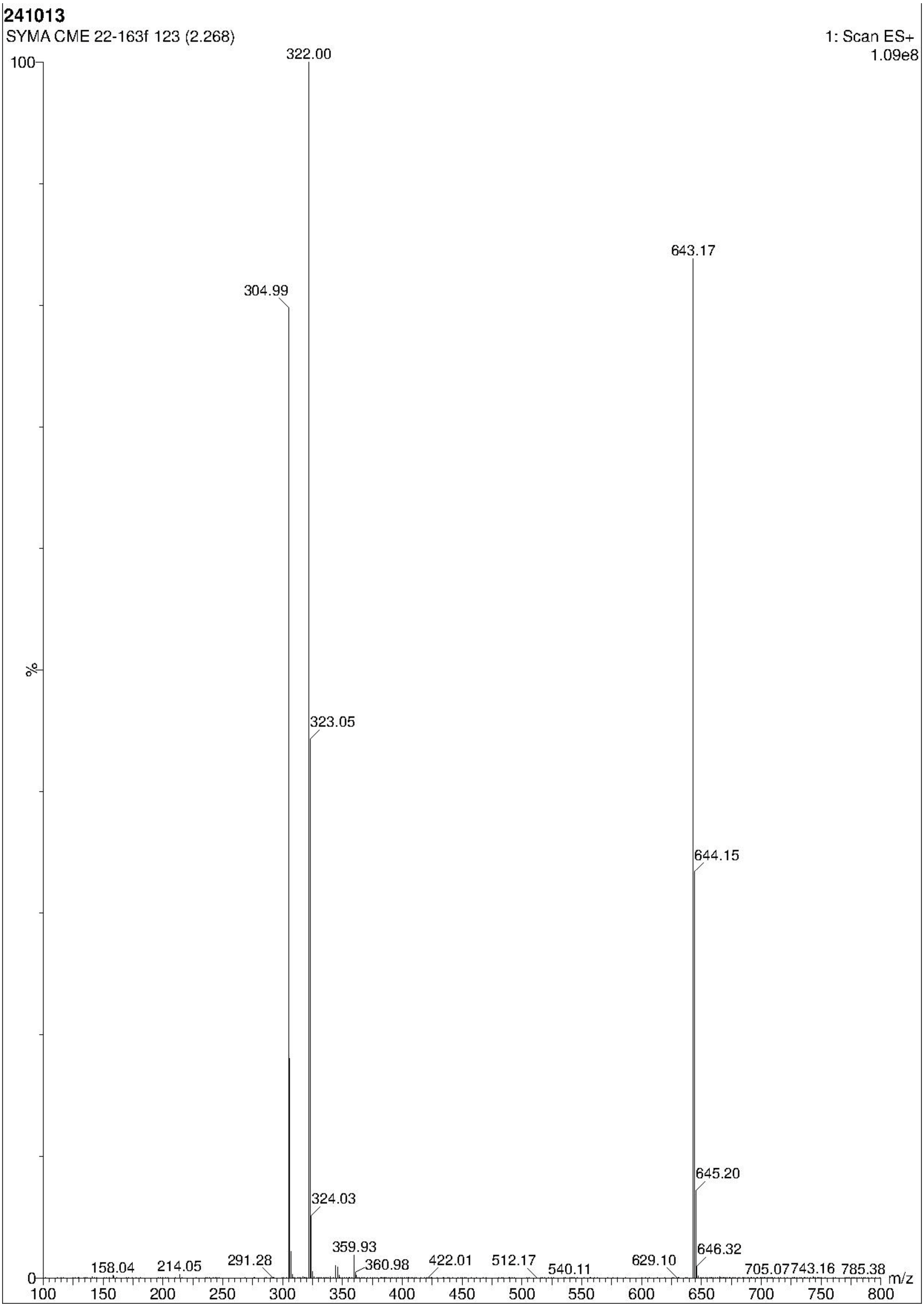
241013
SYMA CME 22-163f 123 (2.268)
1: Scan ES+
1.09e8
322.00
643.17
304.99
323.05
644.15
645.20
324.03
359.93
360.98
158.04
214.05
291.28
422.01
512.17
540.11
629.10
646.32
705.07
743.16
785.38
%
100
0
100
150
200
250
300
350
400
450
500
550
600
650
700
750
800
m/z

methyl 3-[3-[4-(dimethylaminomethyl)phenyl]-4-methyl-pyrazol-1-yl]benzoate (**46**)

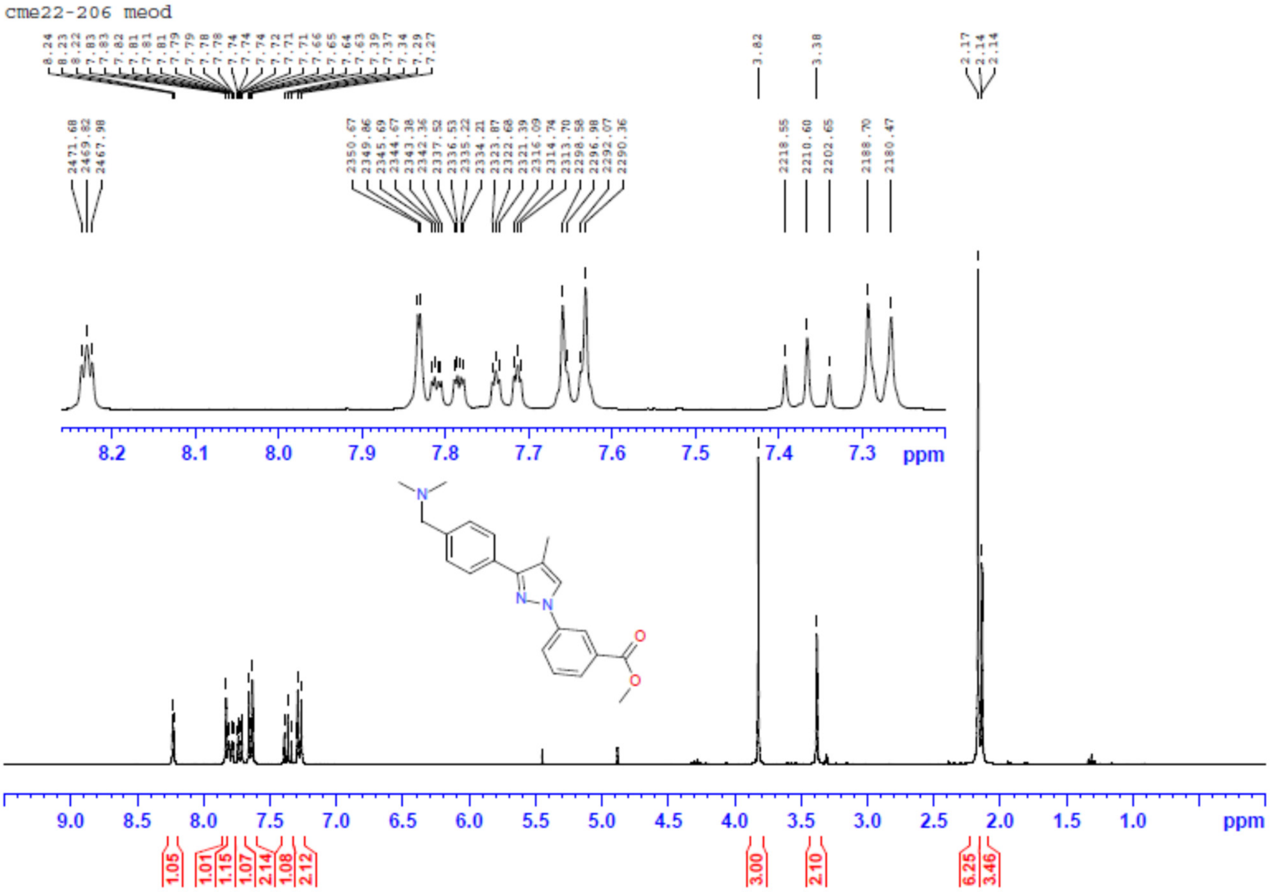

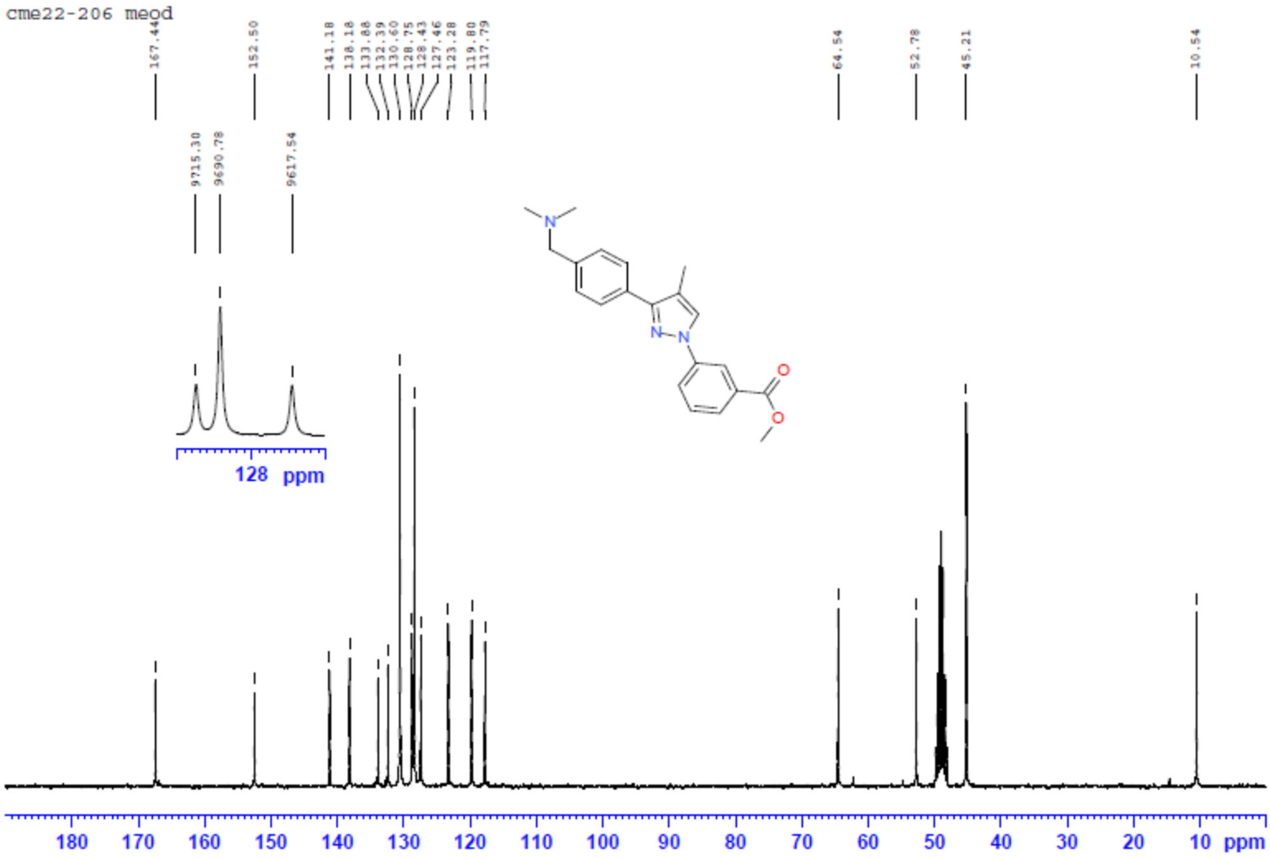

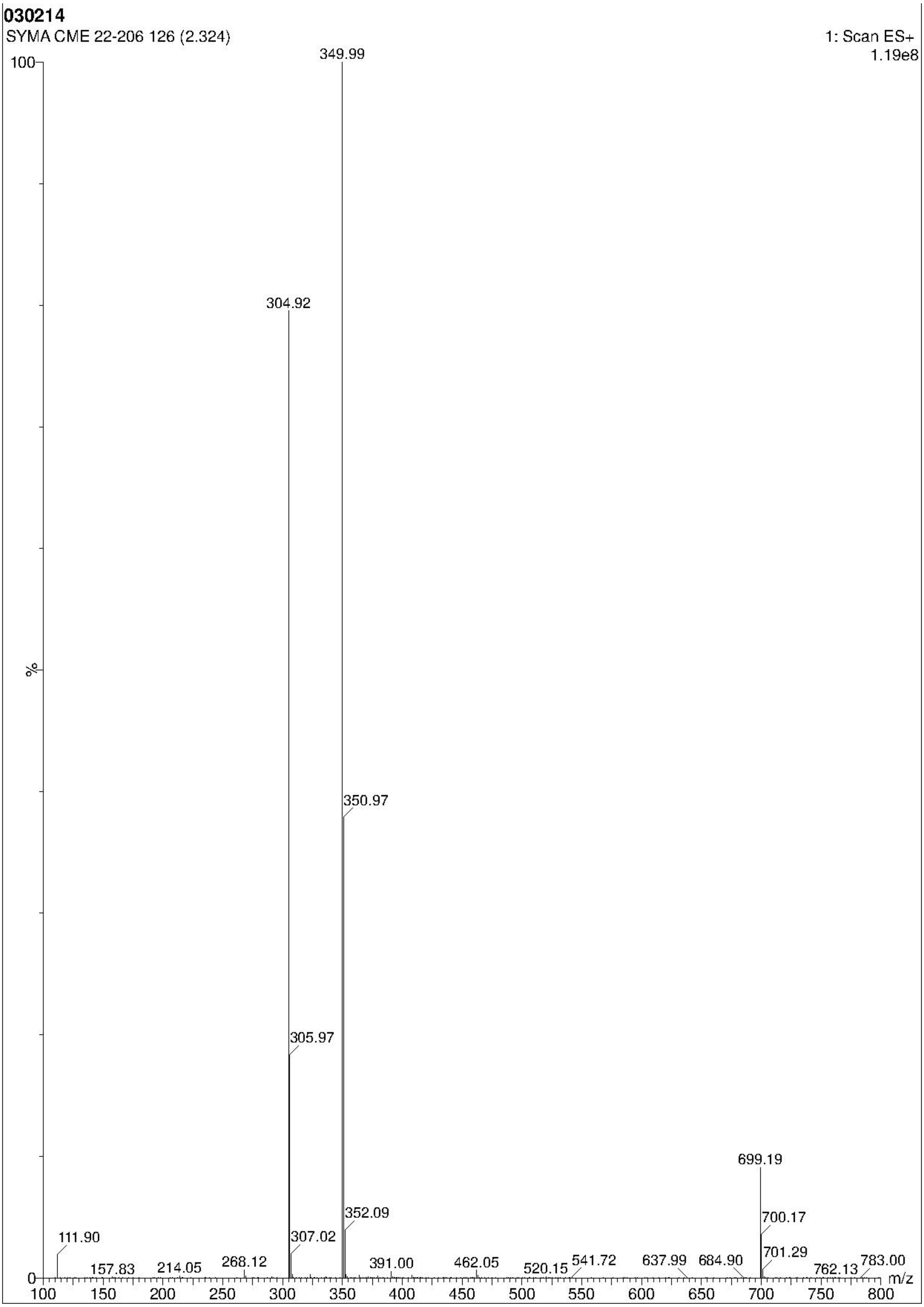
030214
SYMA CME 22-206 126 (2.324)
1: Scan ES+
1.19e8
100
%
0
349.99
304.92
350.97
305.97
699.19
352.09
700.17
111.90
307.02
701.29
157.83
214.05
268.12
391.00
462.05
520.15
541.72
637.99
684.90
762.13
783.00
m/z
100
150
200
250
300
350
400
450
500
550
600
650
700
750
800

methyl 3-[3-[3-(dimethylaminomethyl)phenyl]-4-methyl-pyrazol-1-yl]benzoate (**47**)

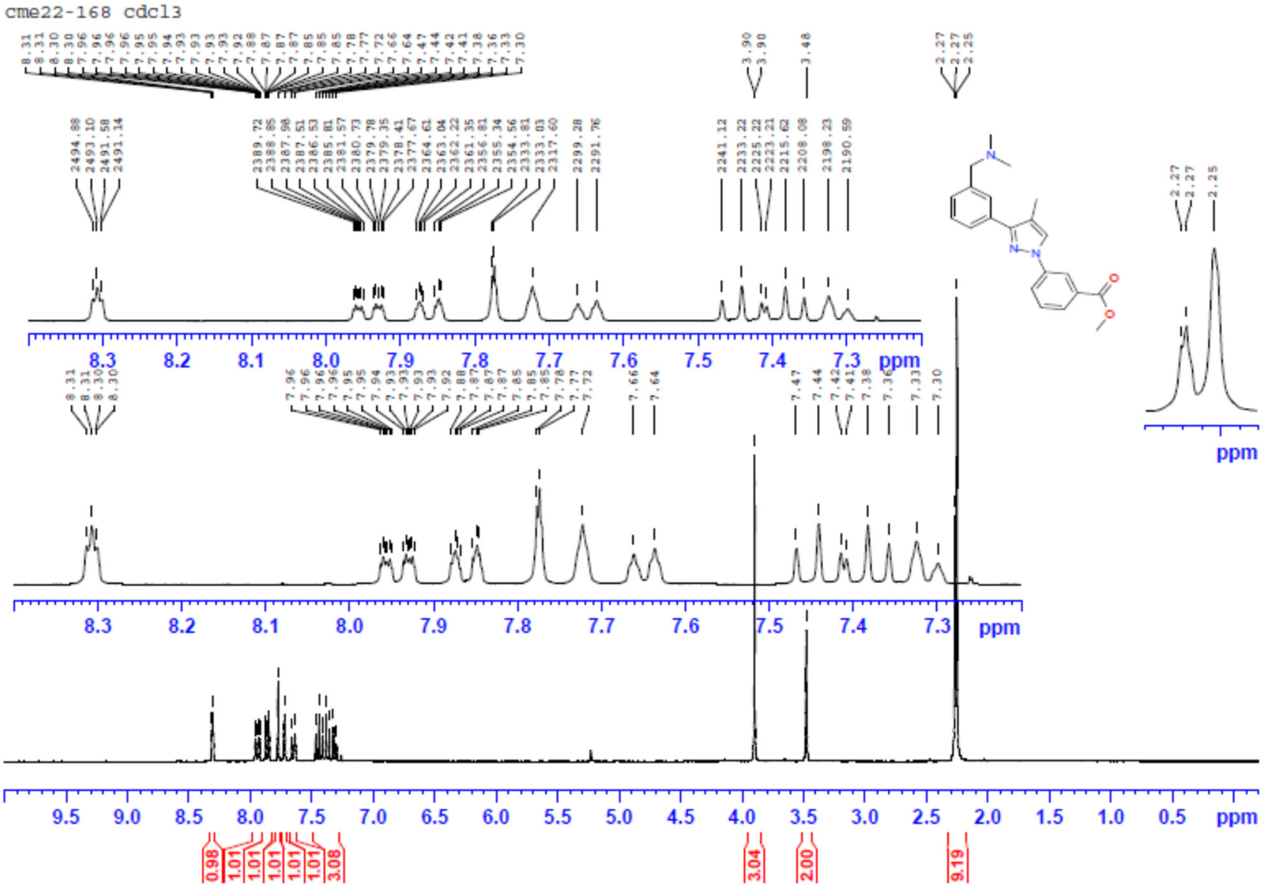

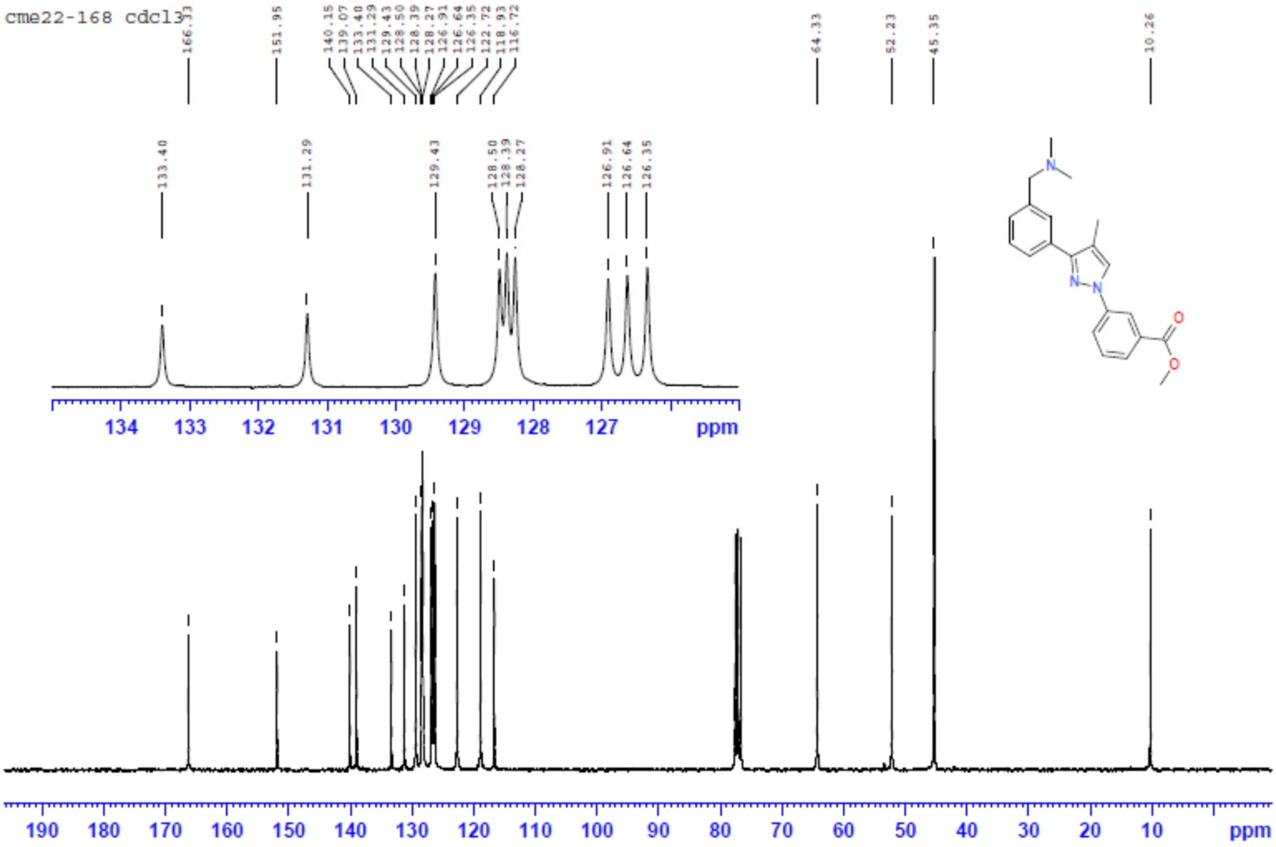

[3-[3-[4-(dimethylaminomethyl)phenyl]-4-methyl-pyrazol-1-yl]phenyl]methanol (**48**)

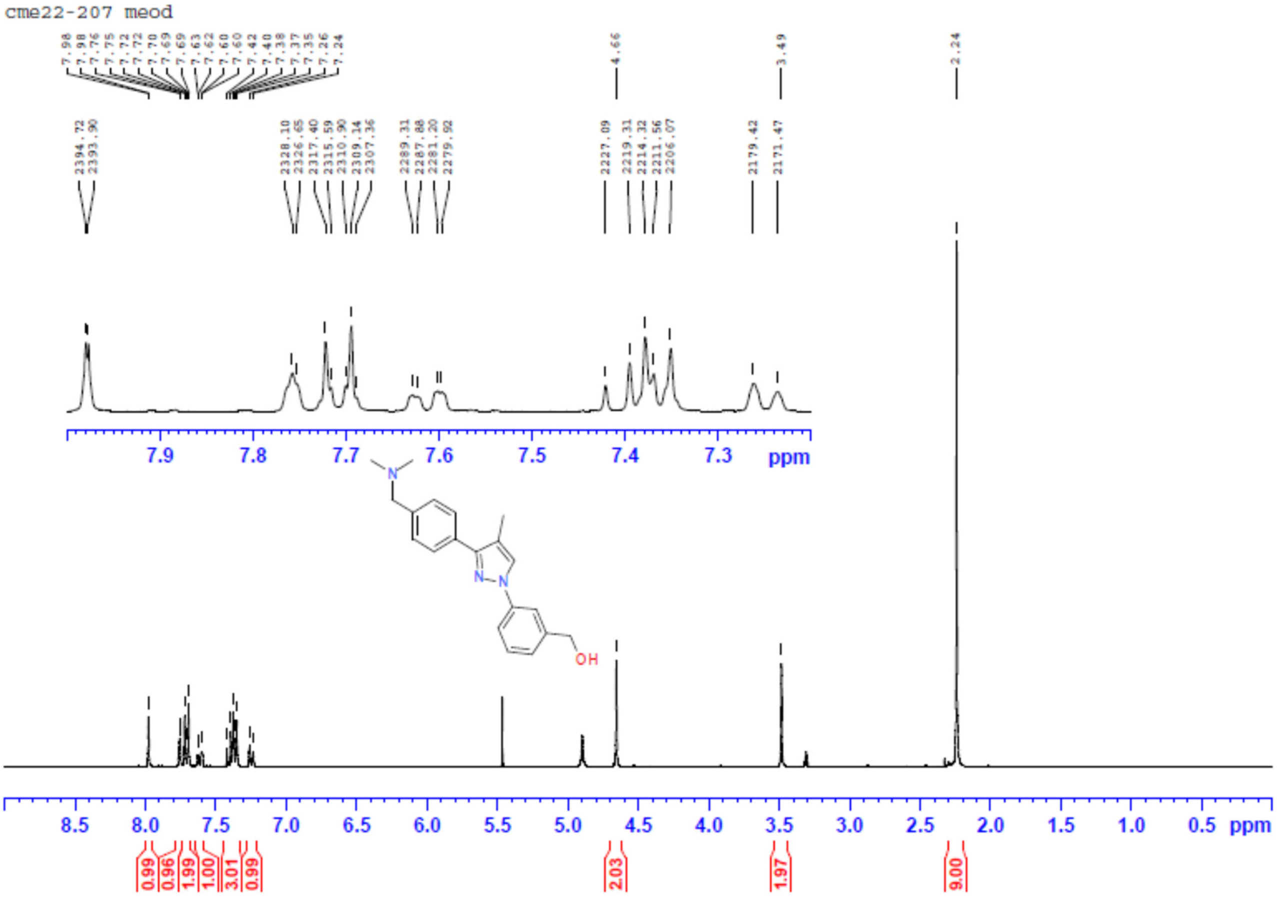

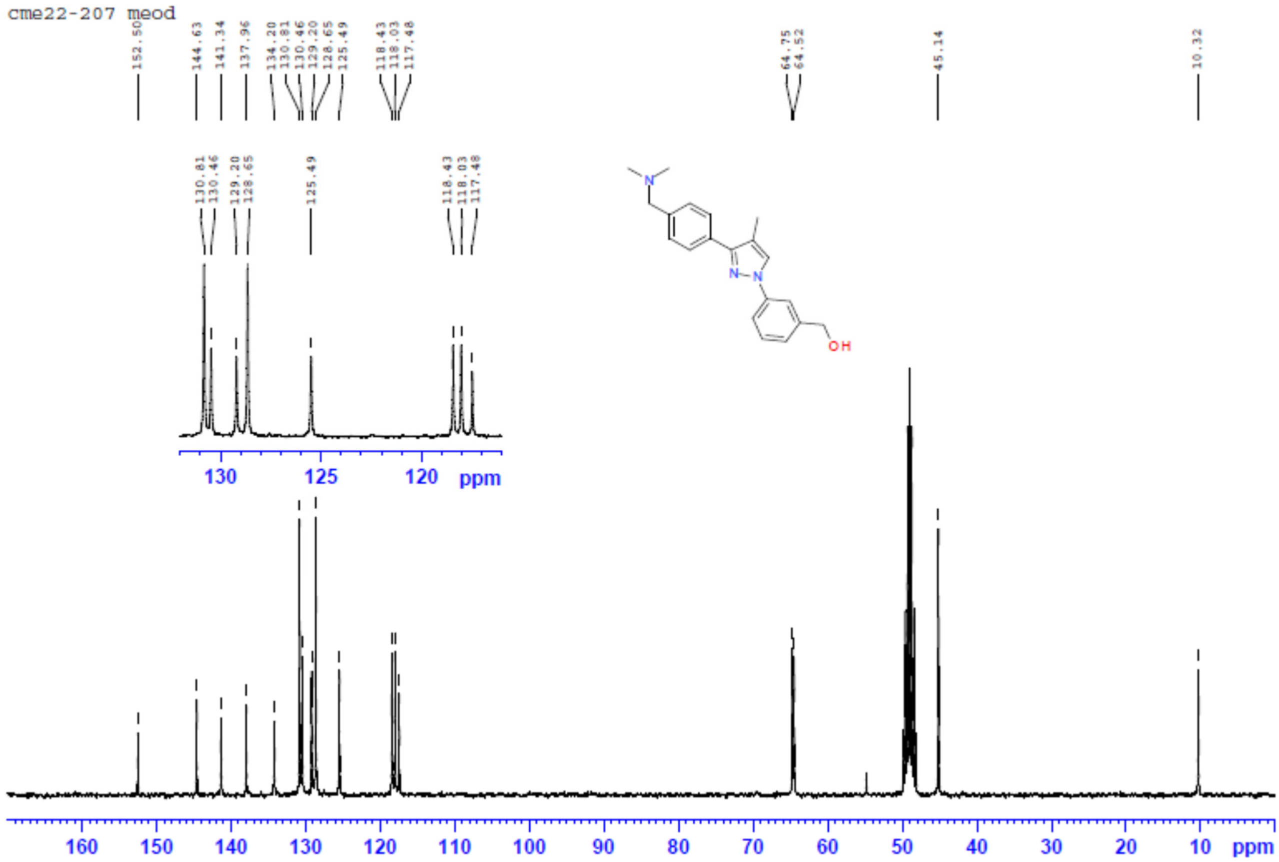

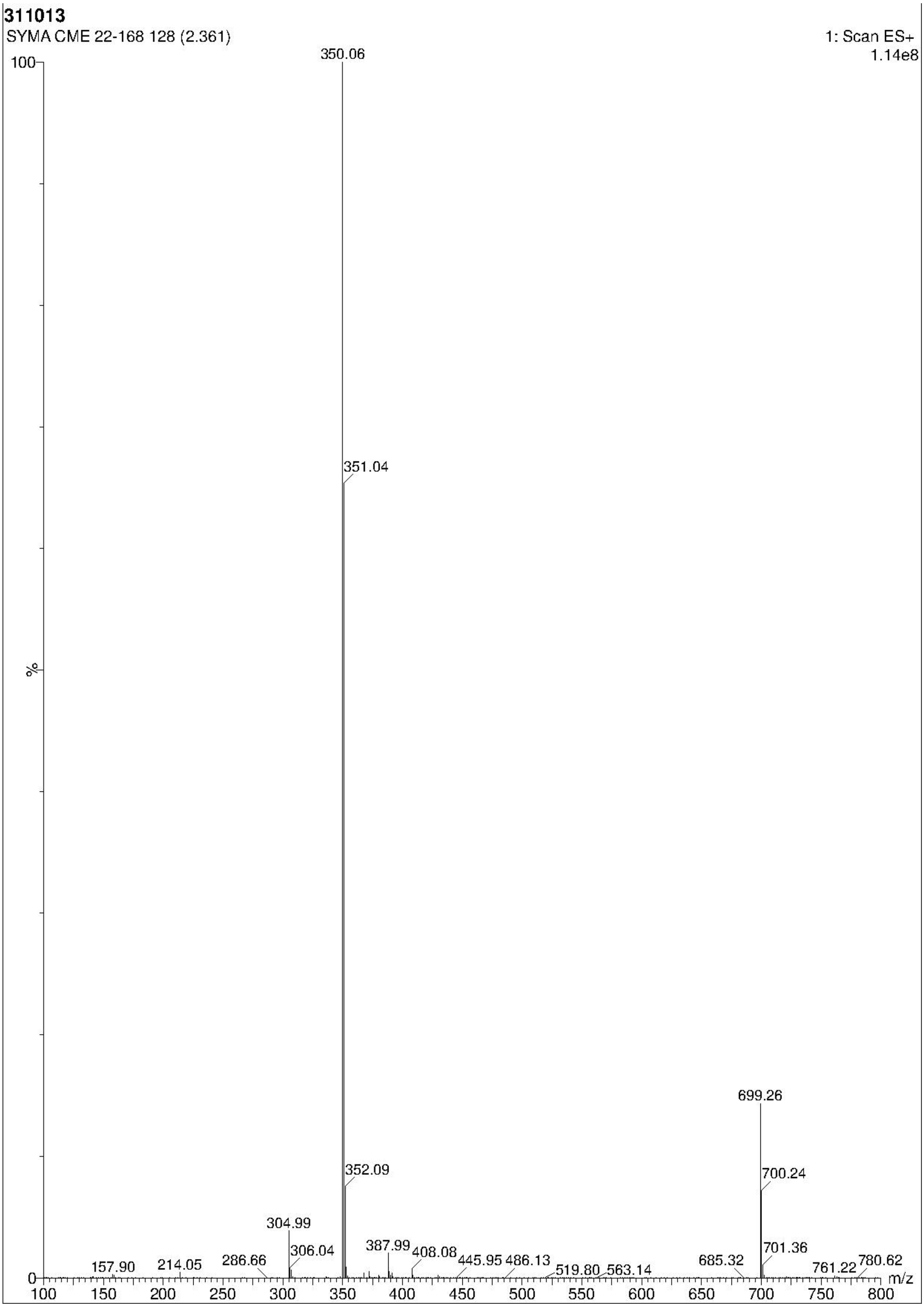

311013
SYMA CME 22-168 128 (2.361)
1: Scan ES+
1.14e8
100
%
0
350.06
351.04
352.09
304.99
306.04
387.99
408.08
445.95
486.13
519.80
563.14
157.90
214.05
286.66
685.32
699.26
700.24
701.36
761.22
780.62
m/z
100
150
200
250
300
350
400
450
500
550
600
650
700
750
800

[3-[3-[3-(dimethylaminomethyl)phenyl]-4-methyl-pyrazol-1-yl]phenyl]methanol (**49**)

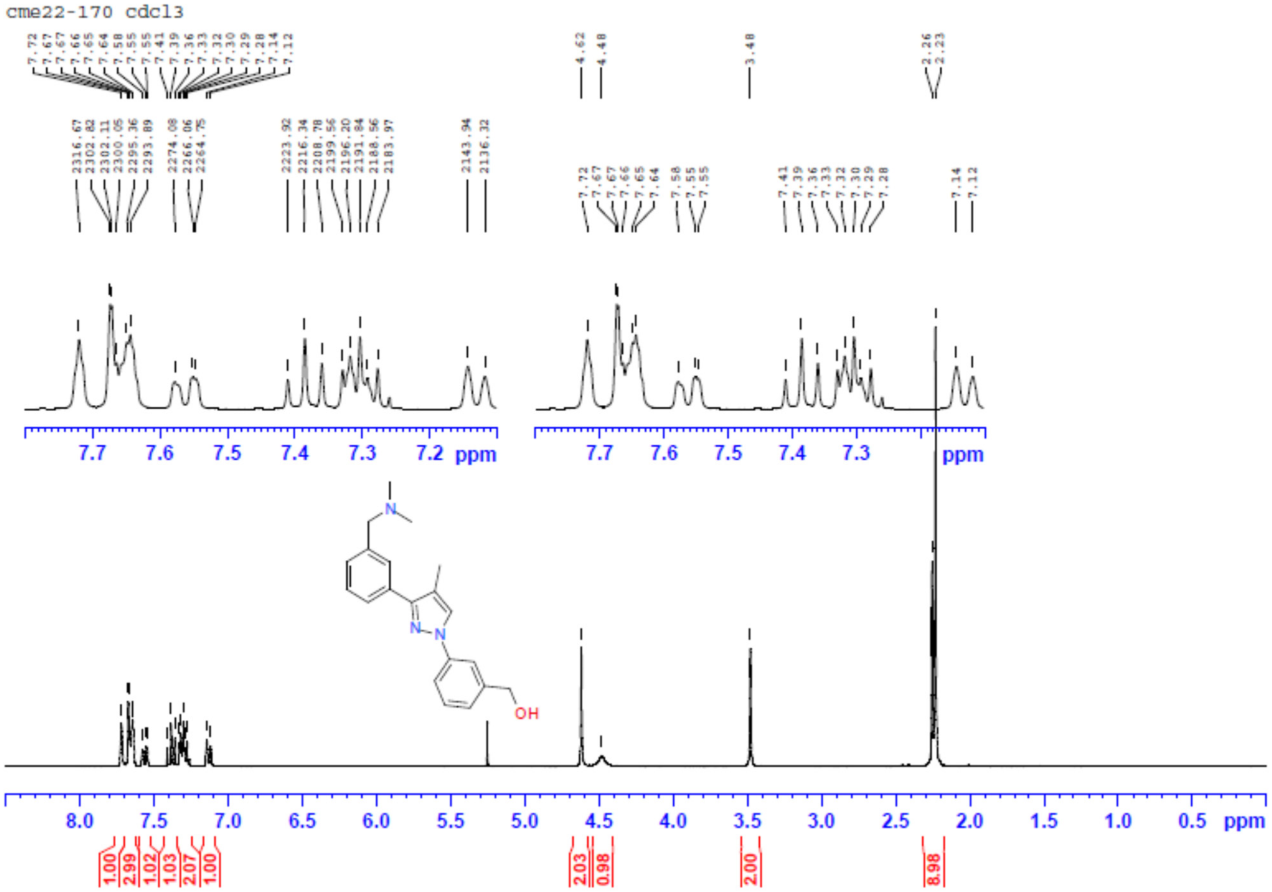

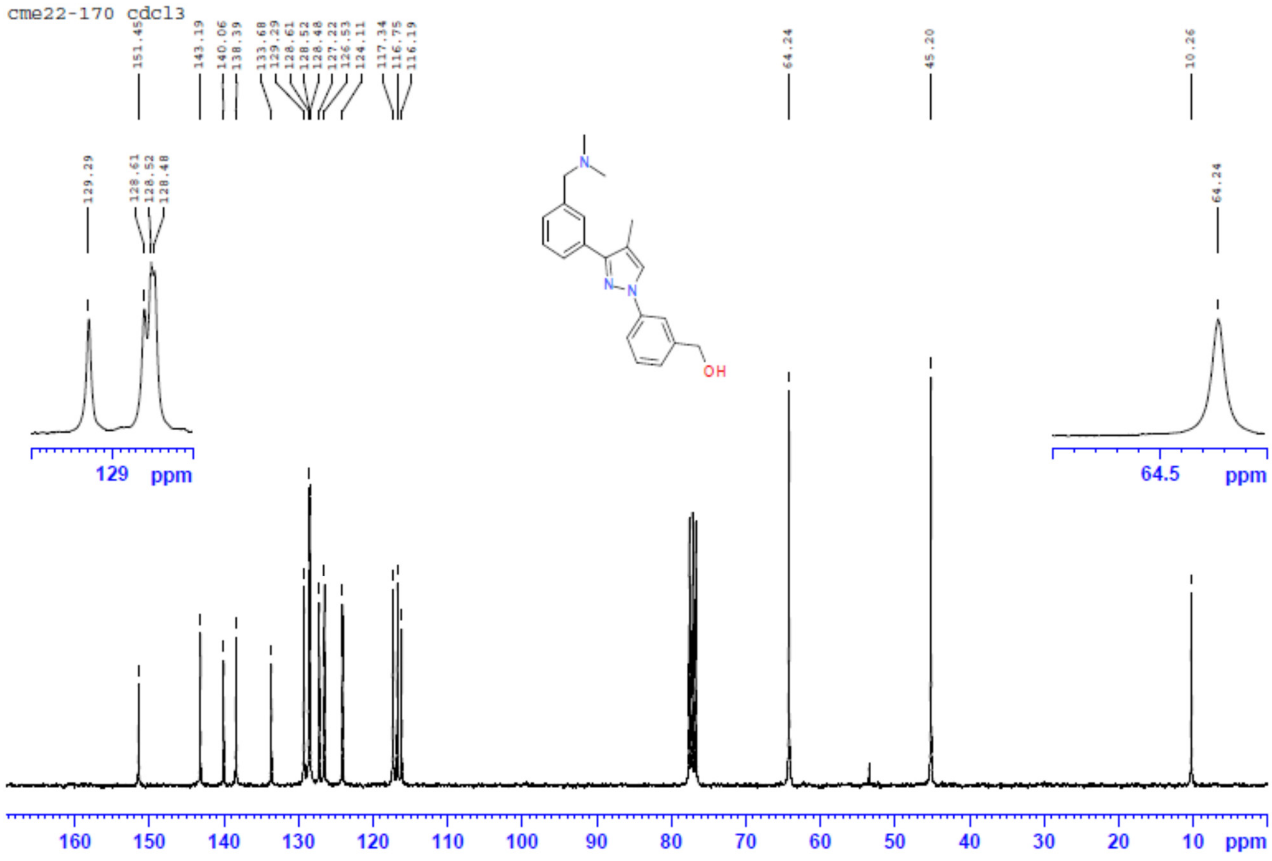

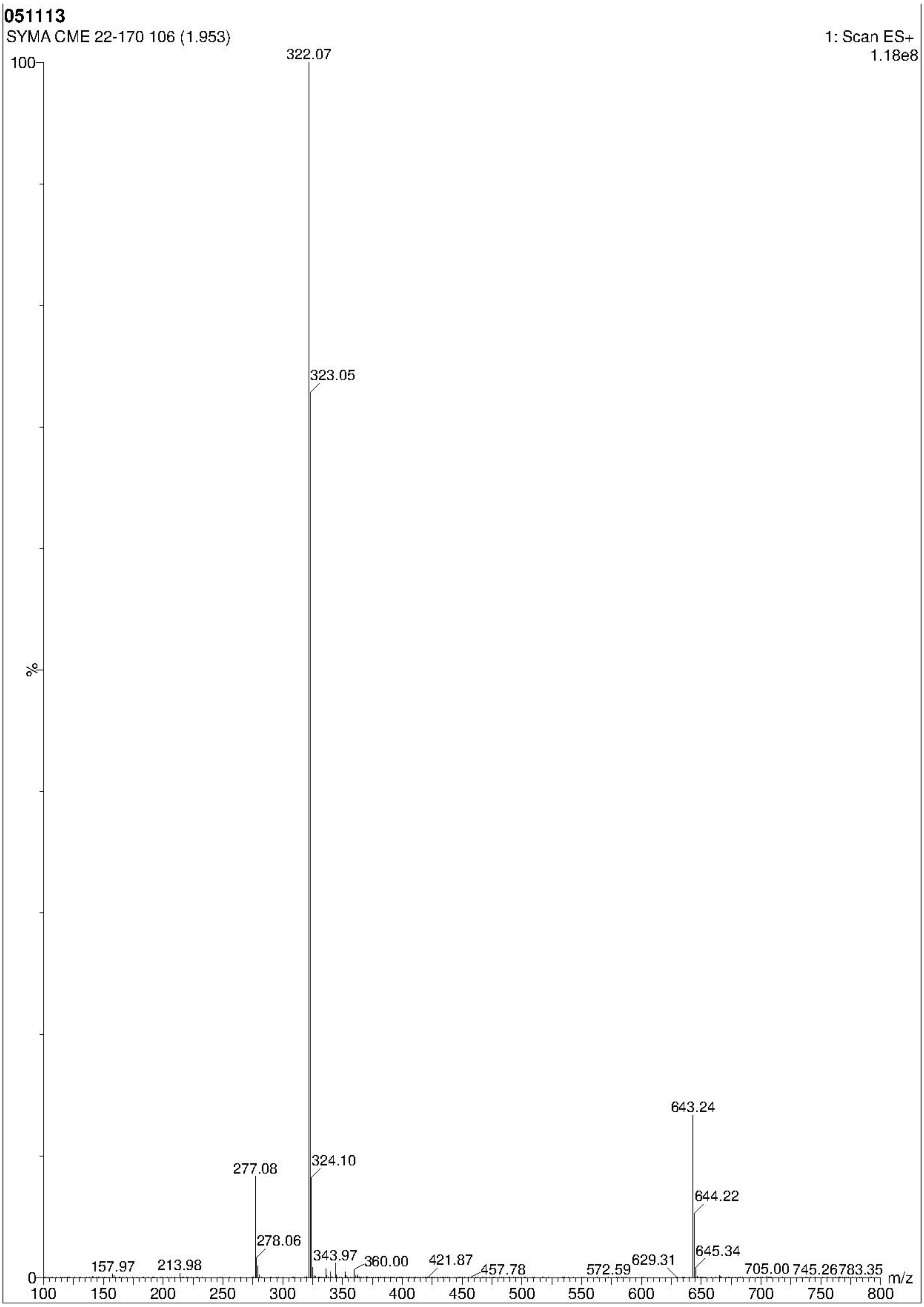

051113
SYMA CME 22-170 106 (1.953)
1: Scan ES+
1.18e8
322.07
323.05
324.10
277.08
278.06
643.24
644.22
645.34
157.97
213.98
343.97
360.00
421.87
457.78
572.59
629.31
705.00
745.26
783.35
%
m/z
100
0
100
150
200
250
300
350
400
450
500
550
600
650
700
750
800

3-(dimethylamino)propanoic acid hydrochloride (**50**)

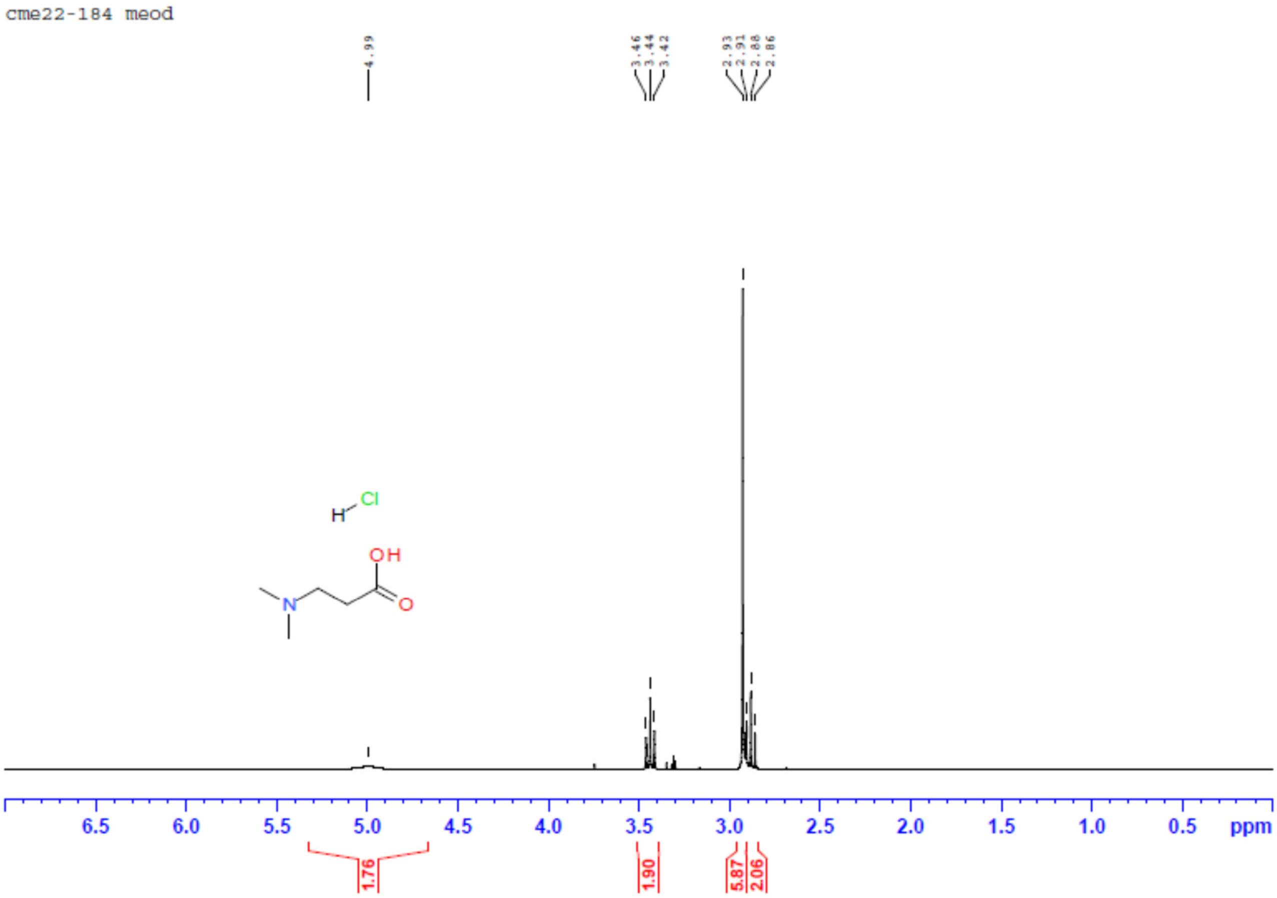

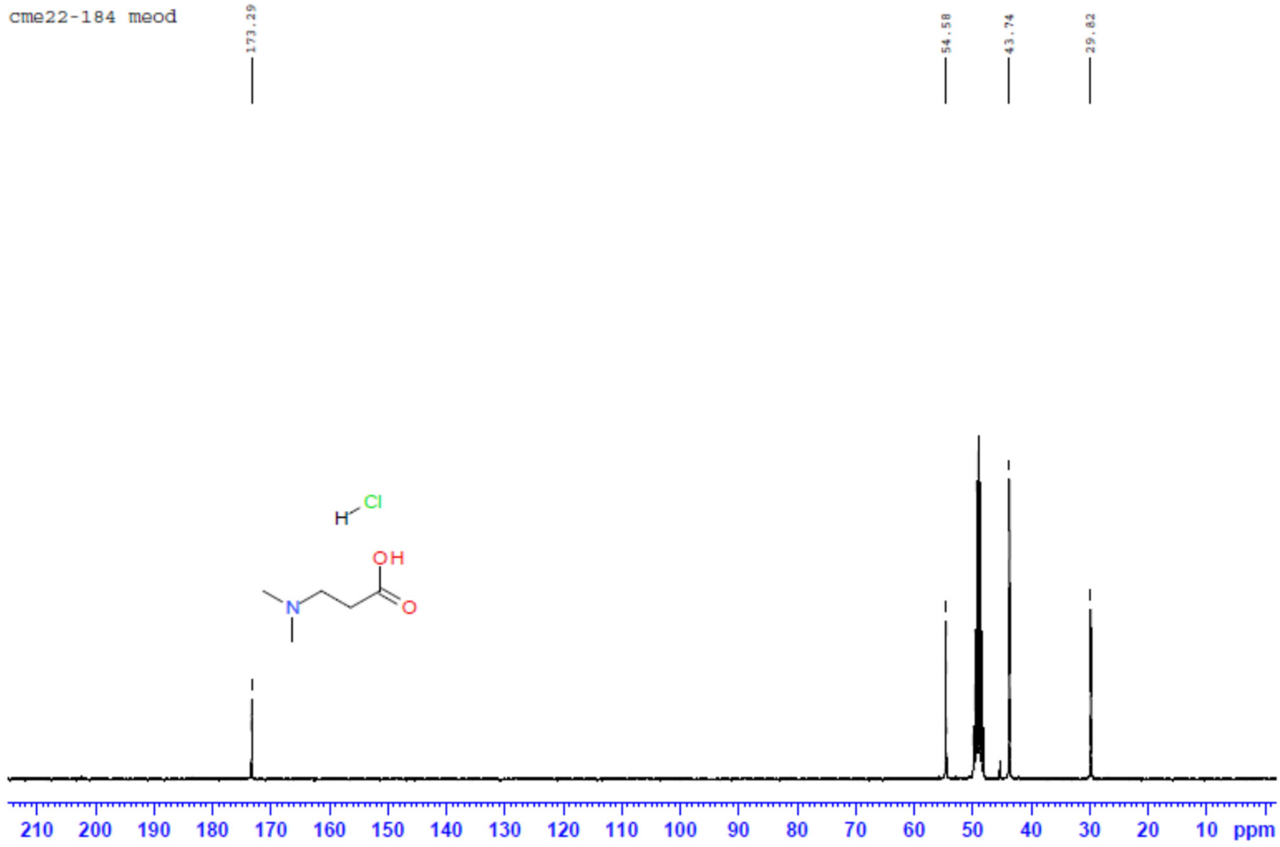

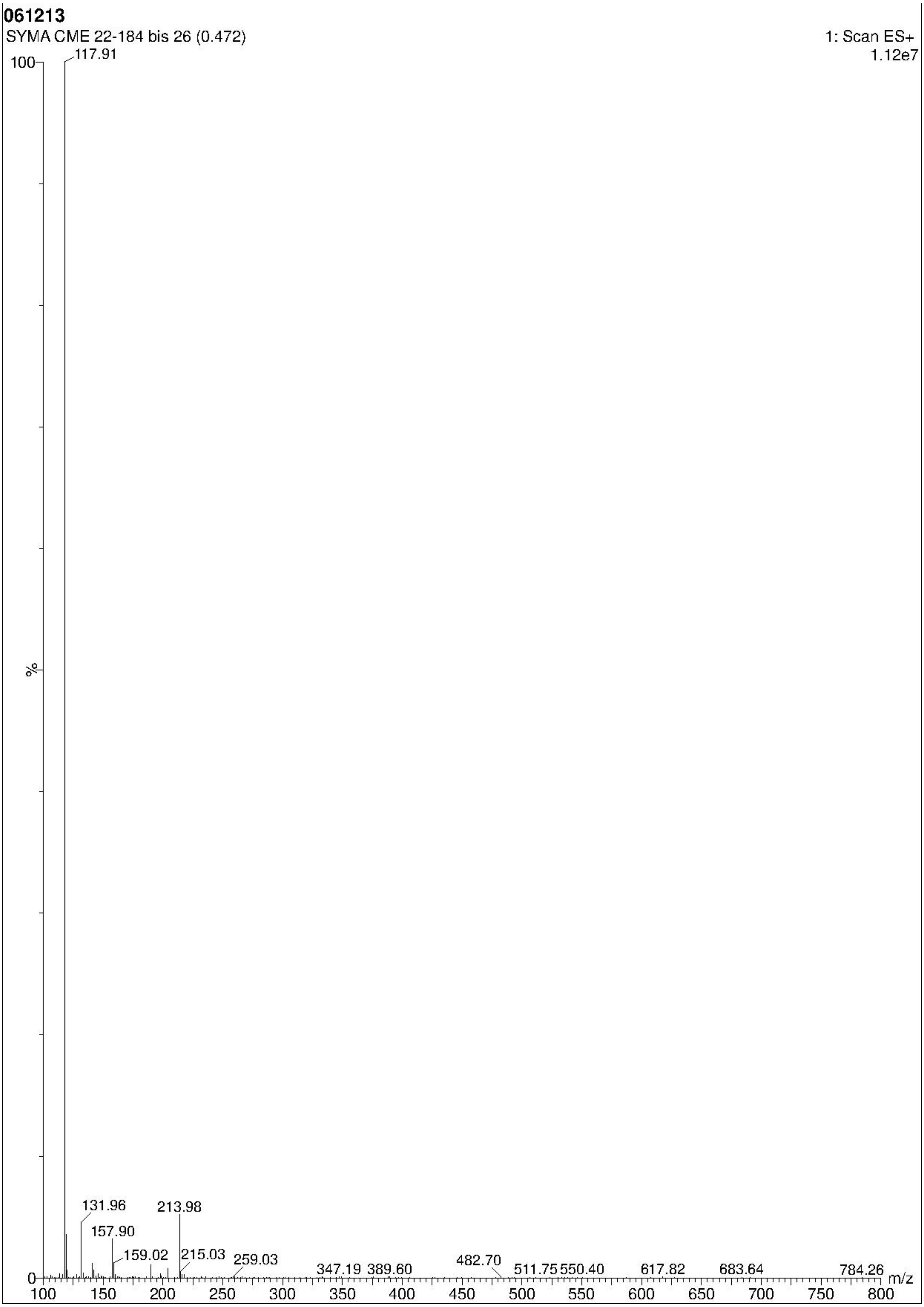

061213
SYMA CME 22-184 bis 26 (0.472)
1: Scan ES+
1.12e7
100
%
0
117.91
131.96
213.98
157.90
159.02
215.03
259.03
347.19
389.60
482.70
511.75
550.40
617.82
683.64
784.26
m/z
100
150
200
250
300
350
400
450
500
550
600
650
700
750
800

methyl 3-[3-(4-{[3-(dimethylamino)propanamido]methyl}phenyl)-4-methyl-1*H*-pyrazol-1-yl] benzoate (**51**)

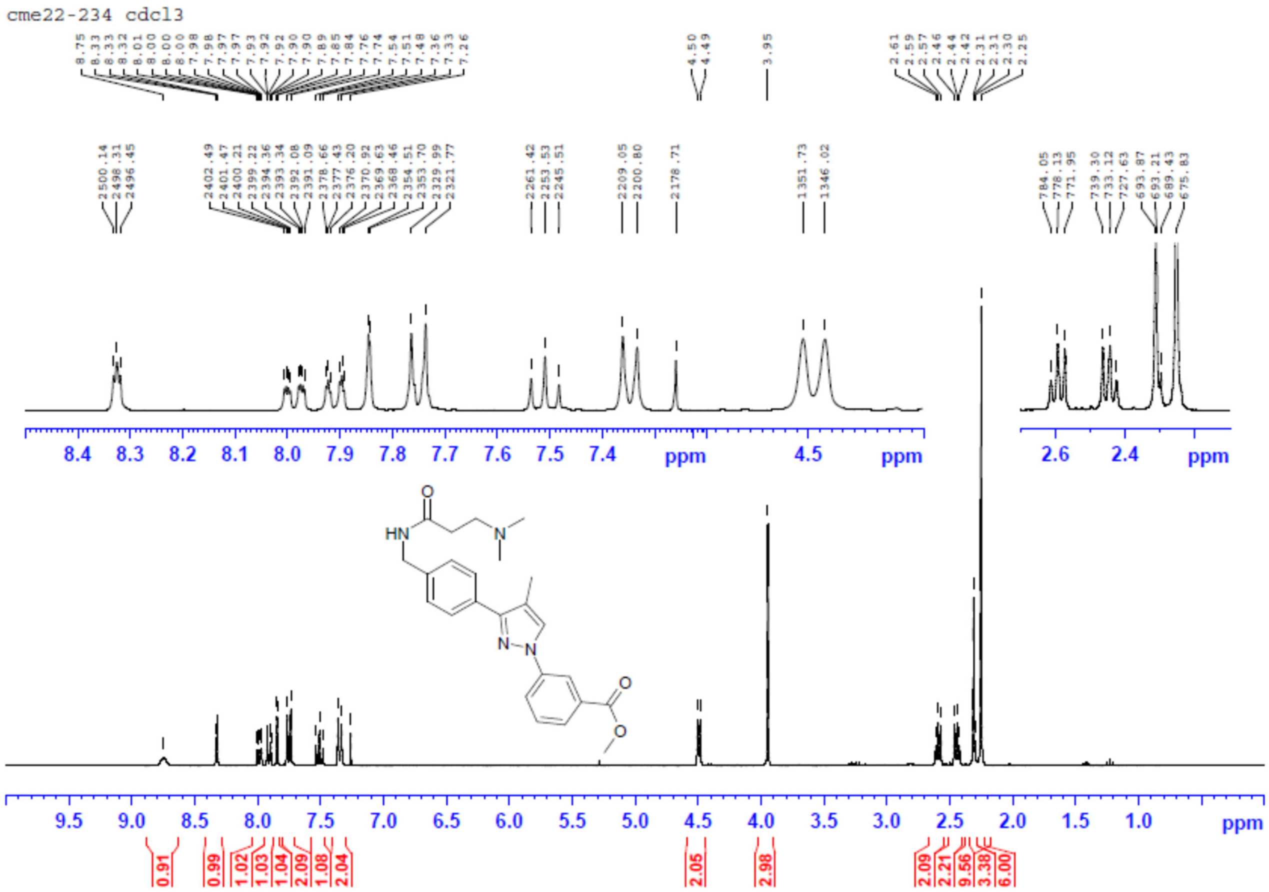

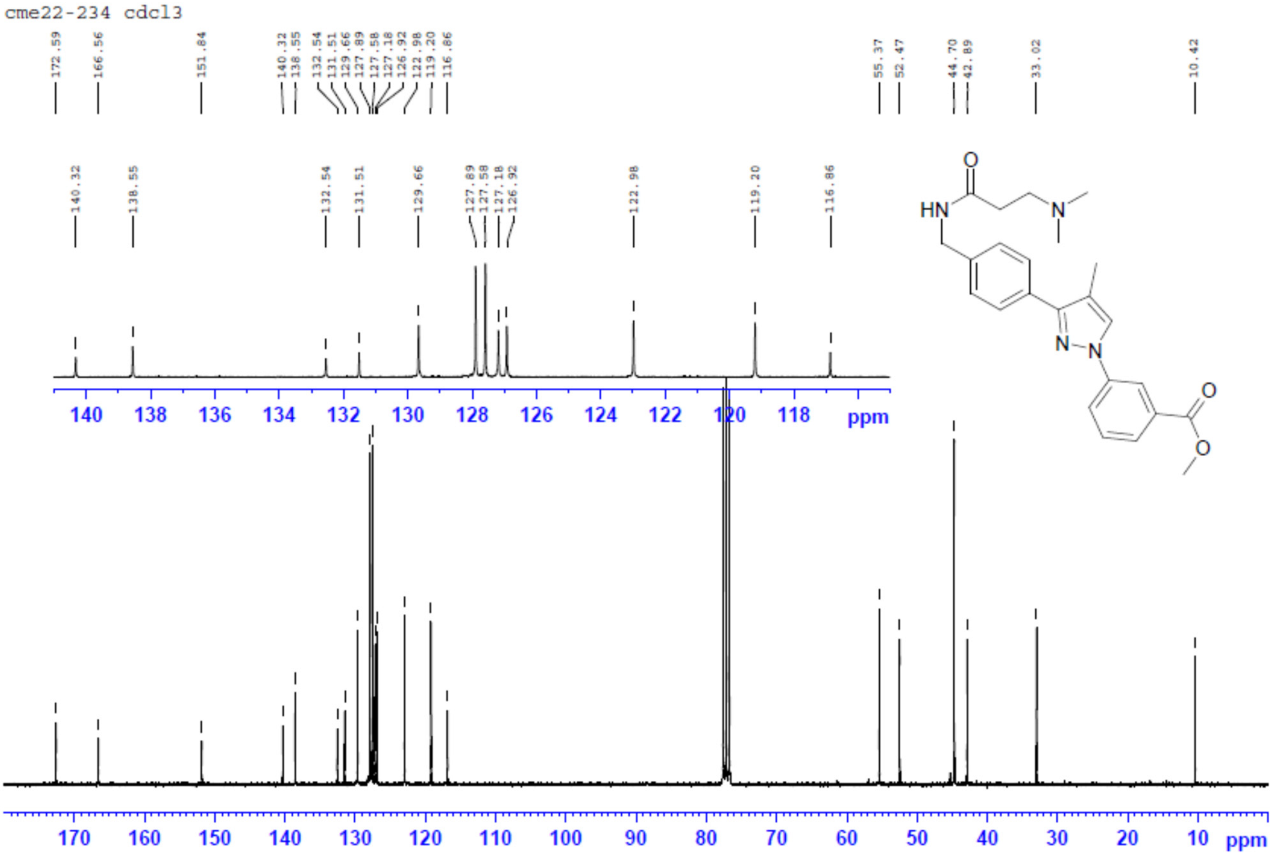

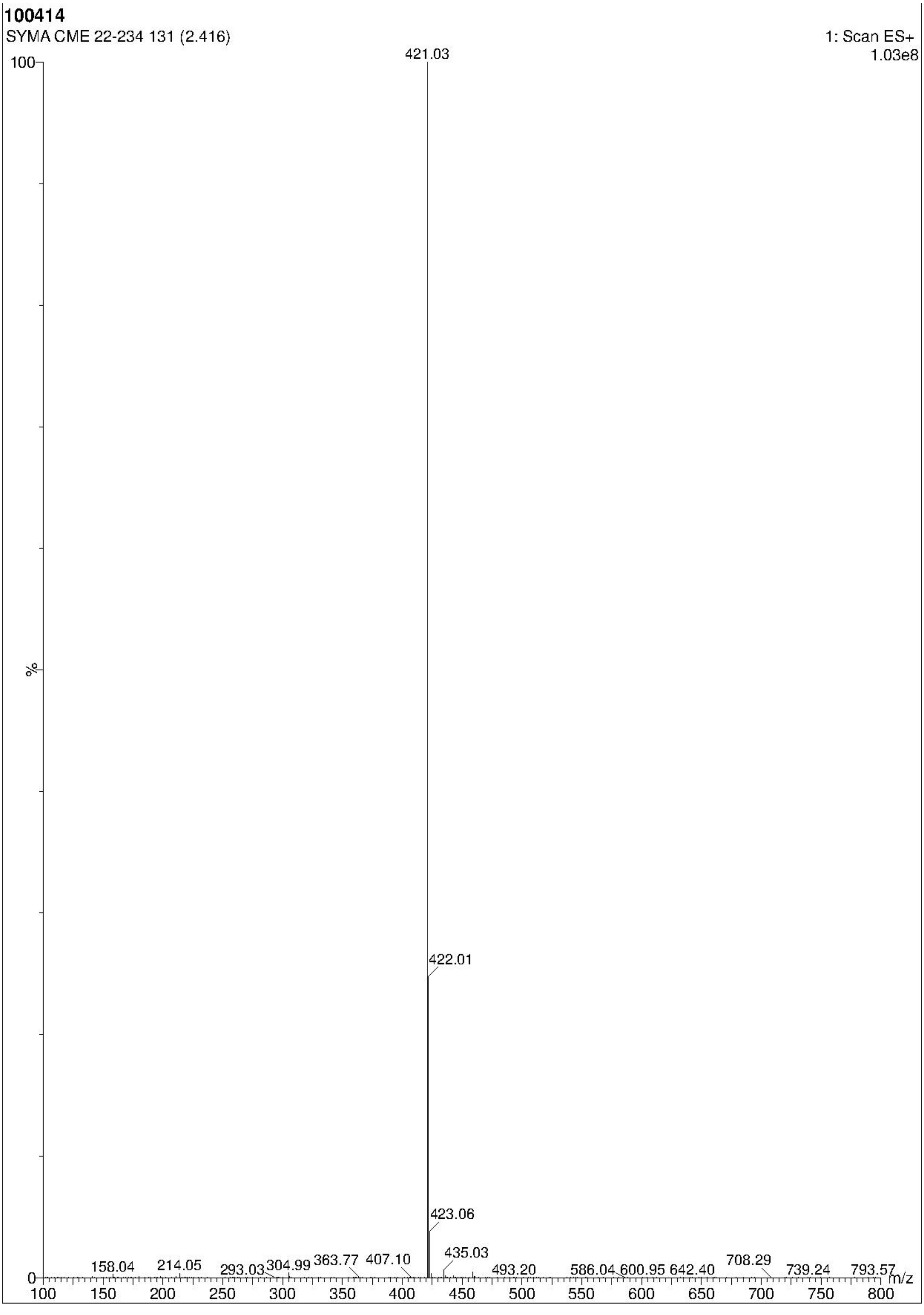

100414
SYMA CME 22-234 131 (2.416)
1: Scan ES+
1.03e8
421.03
422.01
423.06
435.03
158.04
214.05
293.03
304.99
363.77
407.10
493.20
586.04
600.95
642.40
708.29
739.24
793.57
%
m/z
100
0
100 150 200 250 300 350 400 450 500 550 600 650 700 750 800

methyl 3-[3-[3-[[3-(dimethylamino)propanoylamino]methyl]phenyl]-4-methyl-pyrazol-1-yl]benzoate (**52**)

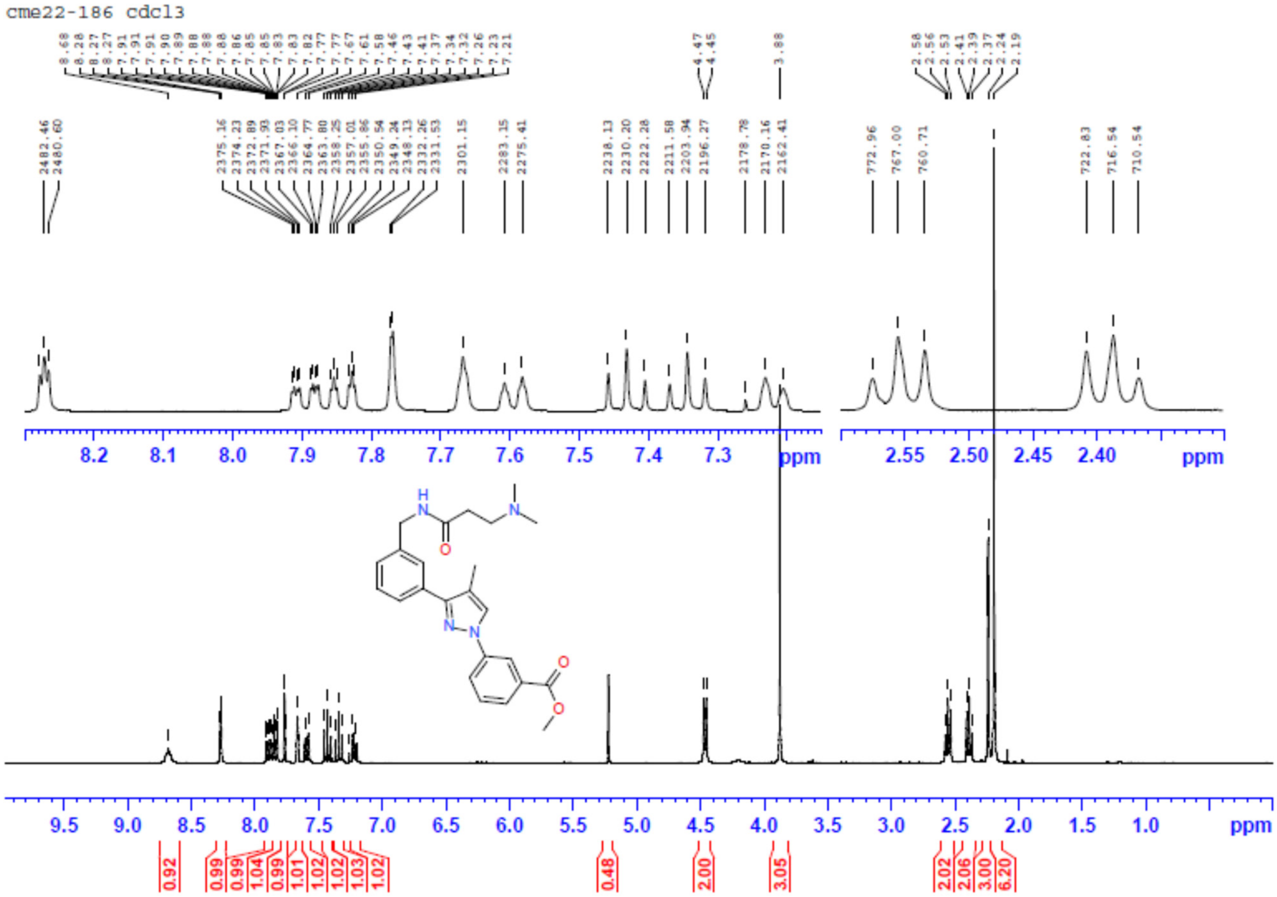

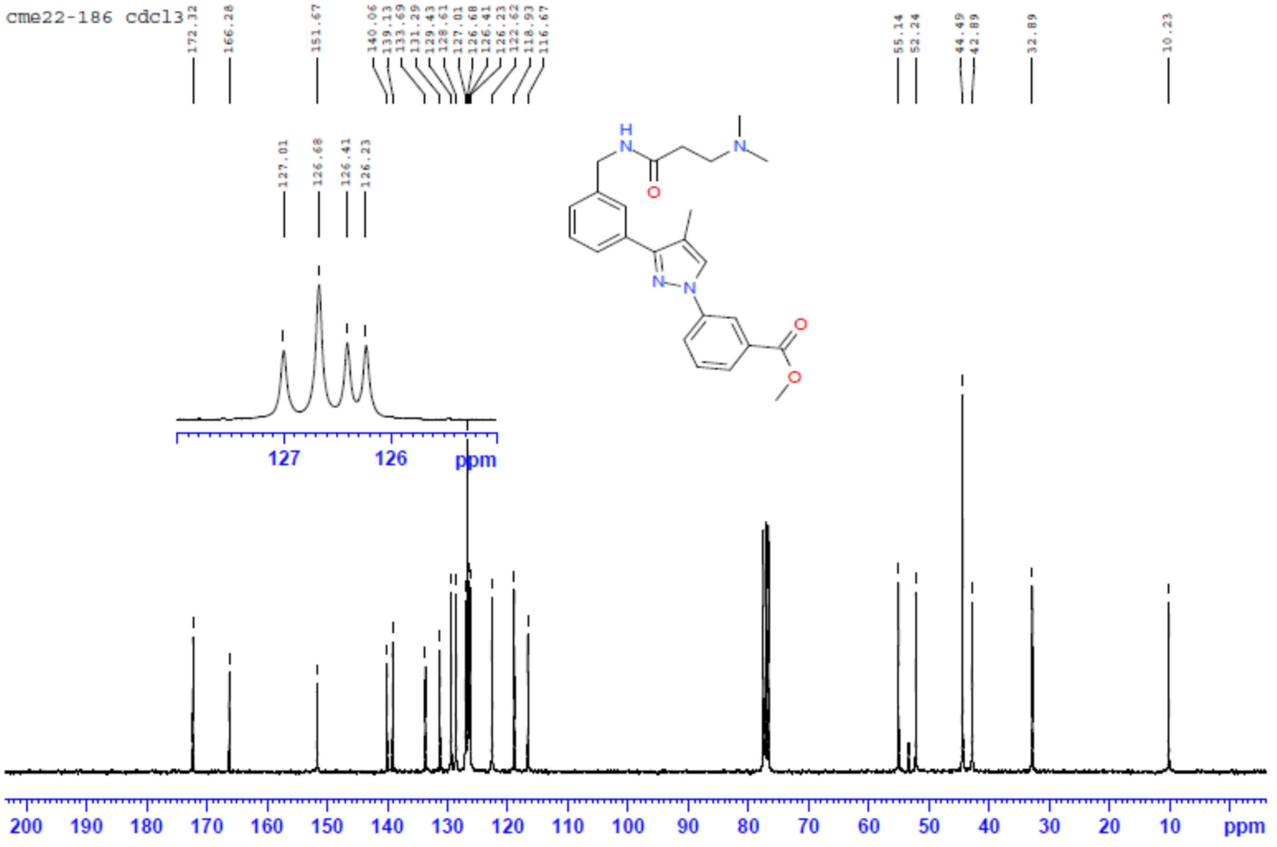

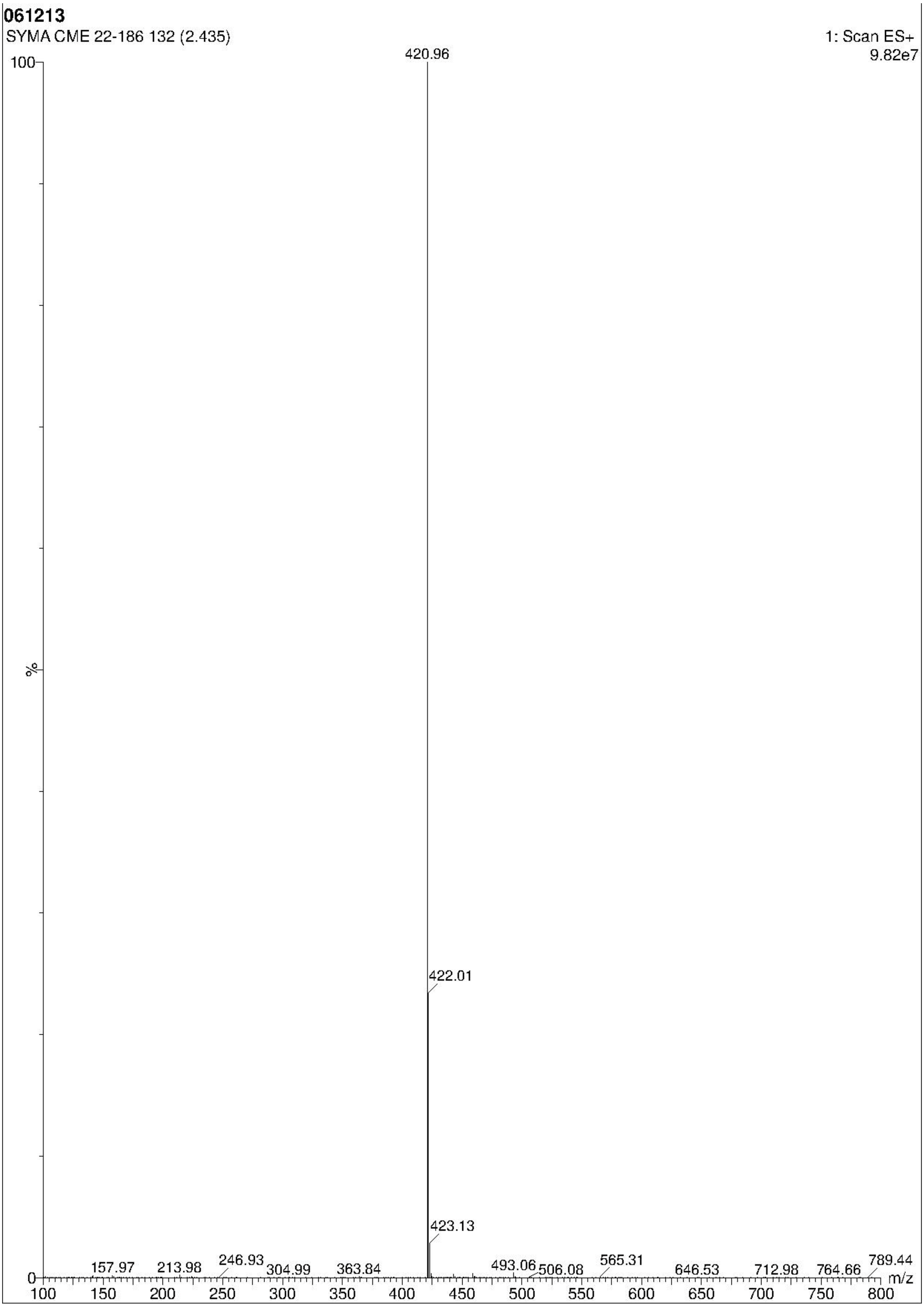
061213
SYMA CME 22-186 132 (2.435)
1: Scan ES+
9.82e7
420.96
422.01
423.13
157.97
213.98
246.93
304.99
363.84
493.06
506.08
565.31
646.53
712.98
764.66
789.44
%
m/z
100
0
100
150
200
250
300
350
400
450
500
550
600
650
700
750
800

(3-{3-[4-({[3-(dimethylamino)propyl]amino}methyl)phenyl]-4-methyl-1*H*-pyrazol-1-yl}phenyl)methanol (**53**)

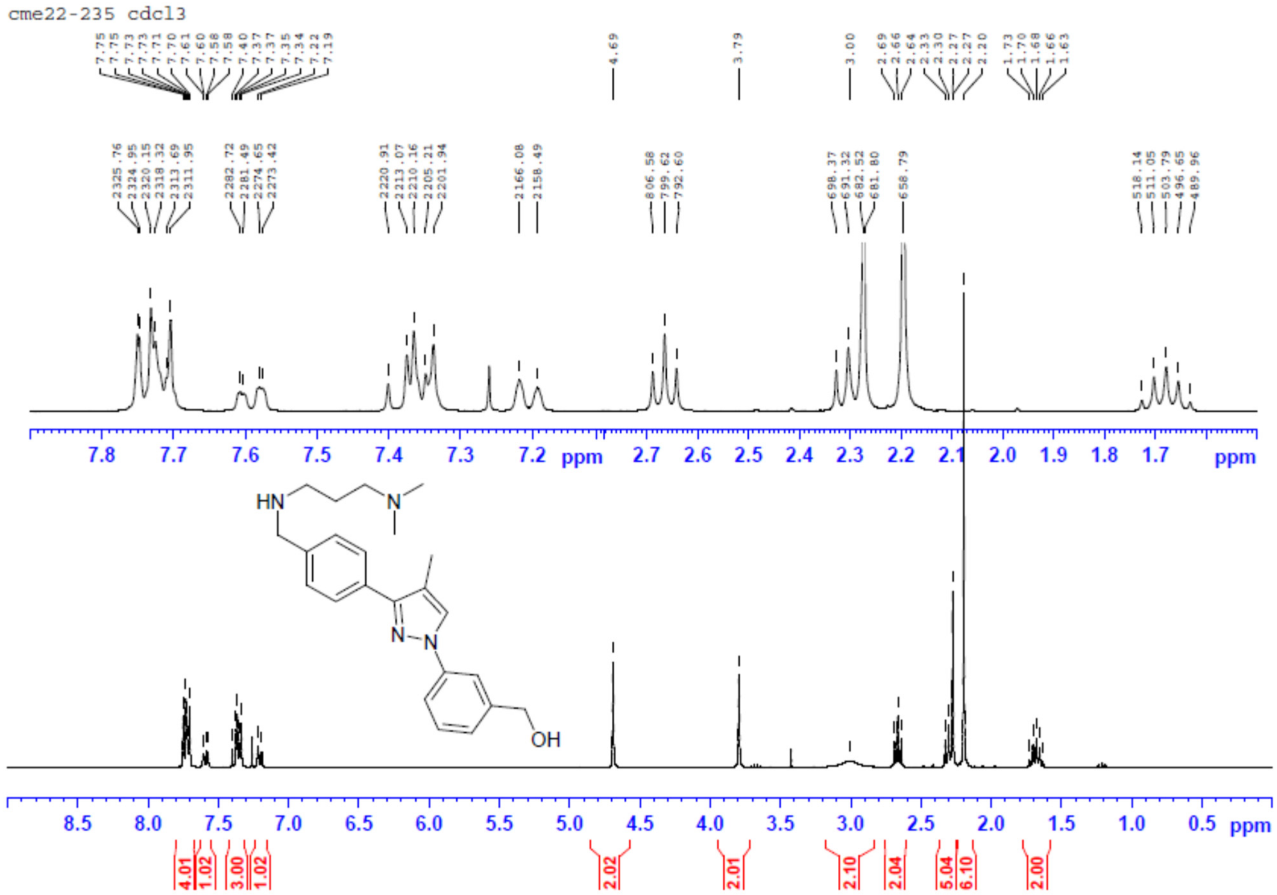

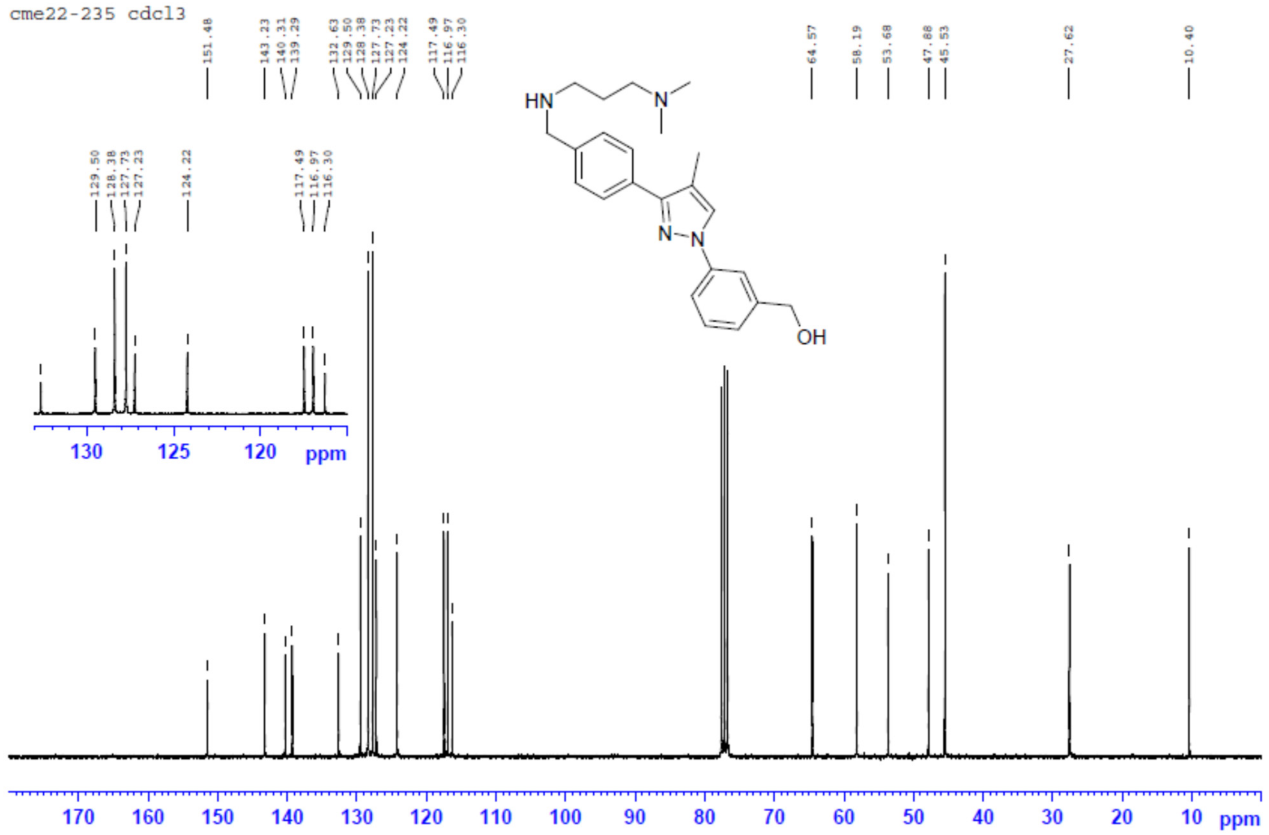

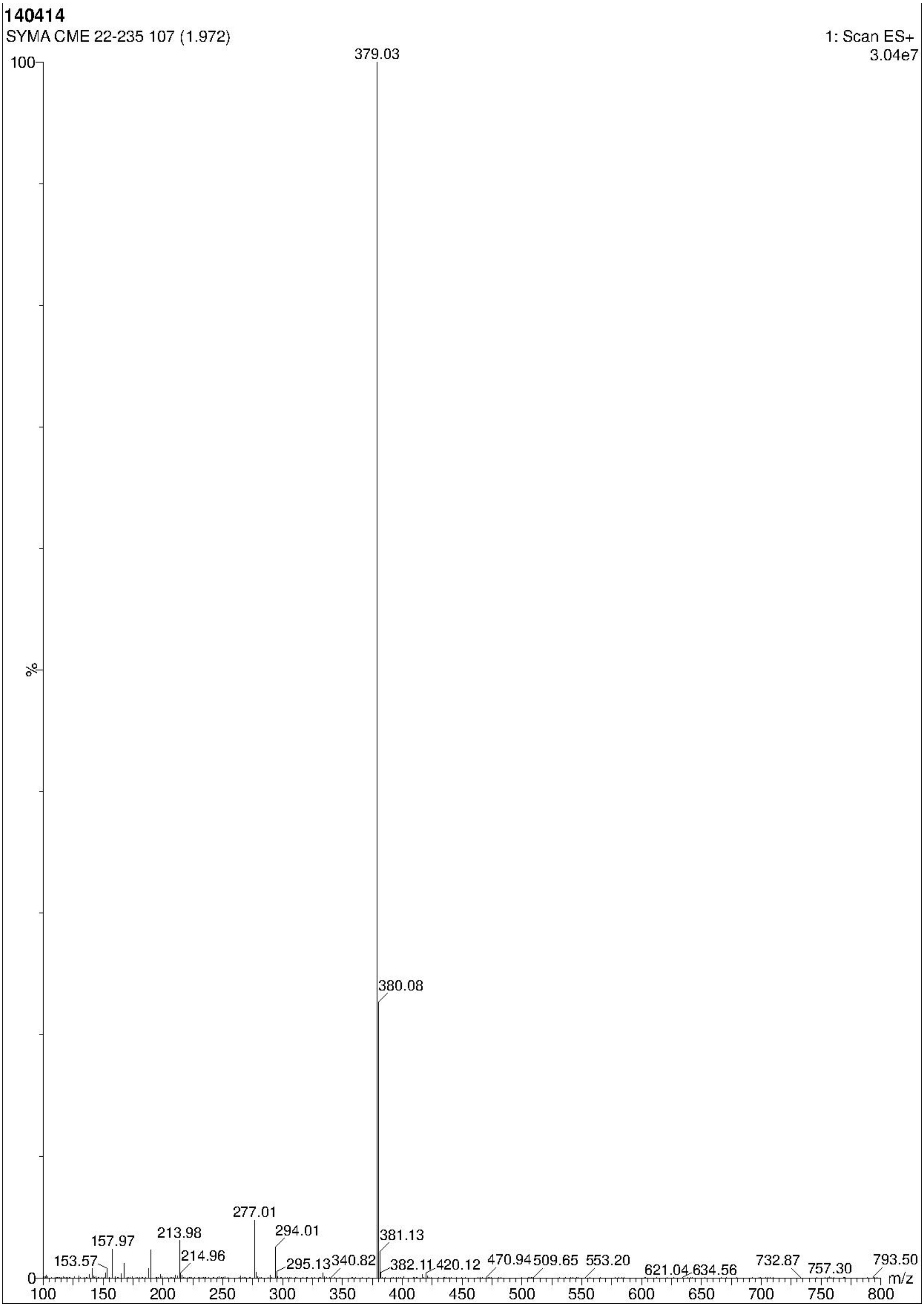
140414
SYMA CME 22-235 107 (1.972)
1: Scan ES+
3.04e7
100
%
0
379.03
380.08
381.13
382.11
277.01
294.01
295.13
340.82
213.98
214.96
157.97
153.57
420.12
470.94
509.65
553.20
621.04
634.56
732.87
757.30
793.50
m/z
100
150
200
250
300
350
400
450
500
550
600
650
700
750
800

[3-[3-[3-[[3-(dimethylamino)propylamino]methyl]phenyl]-4-methyl-pyrazol-1-yl]phenyl]methanol (**54**)

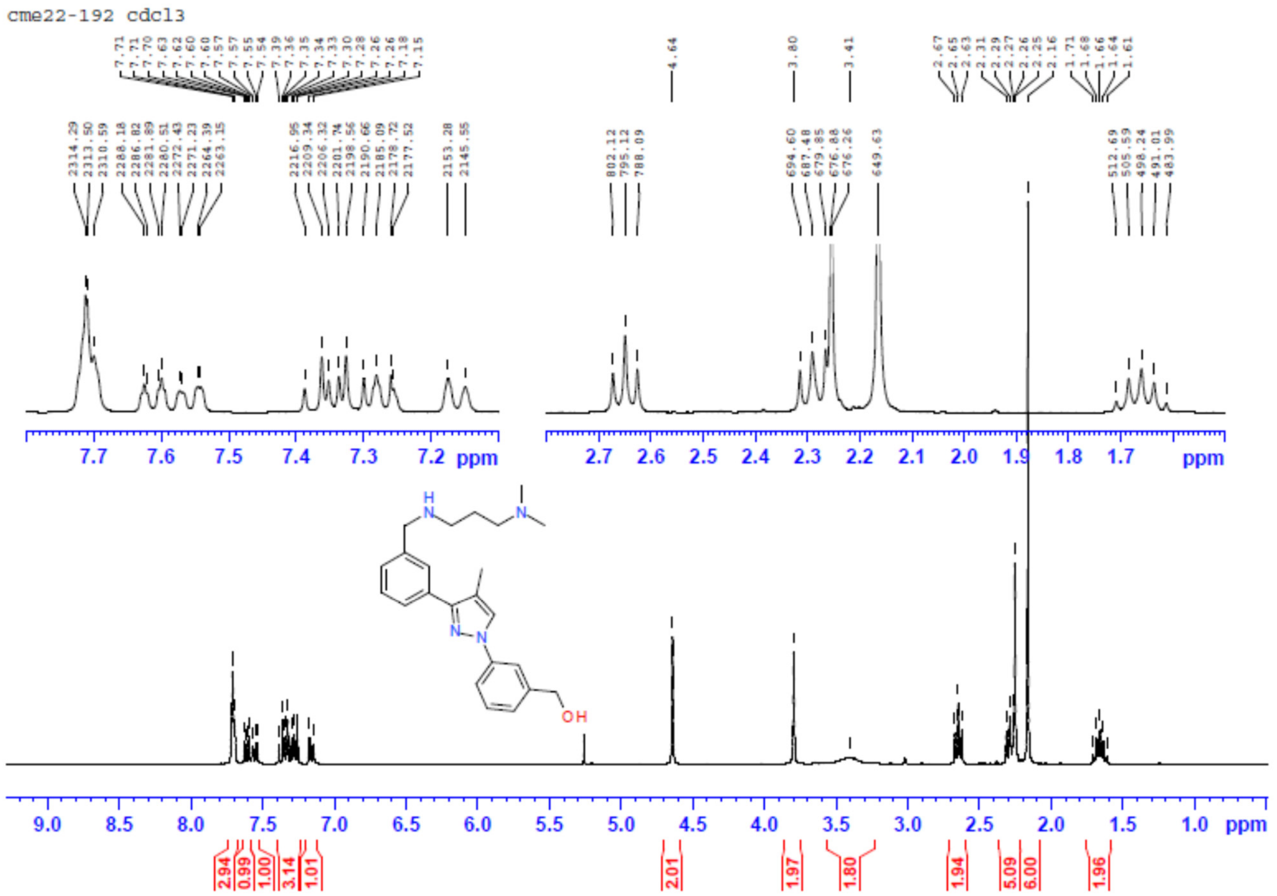

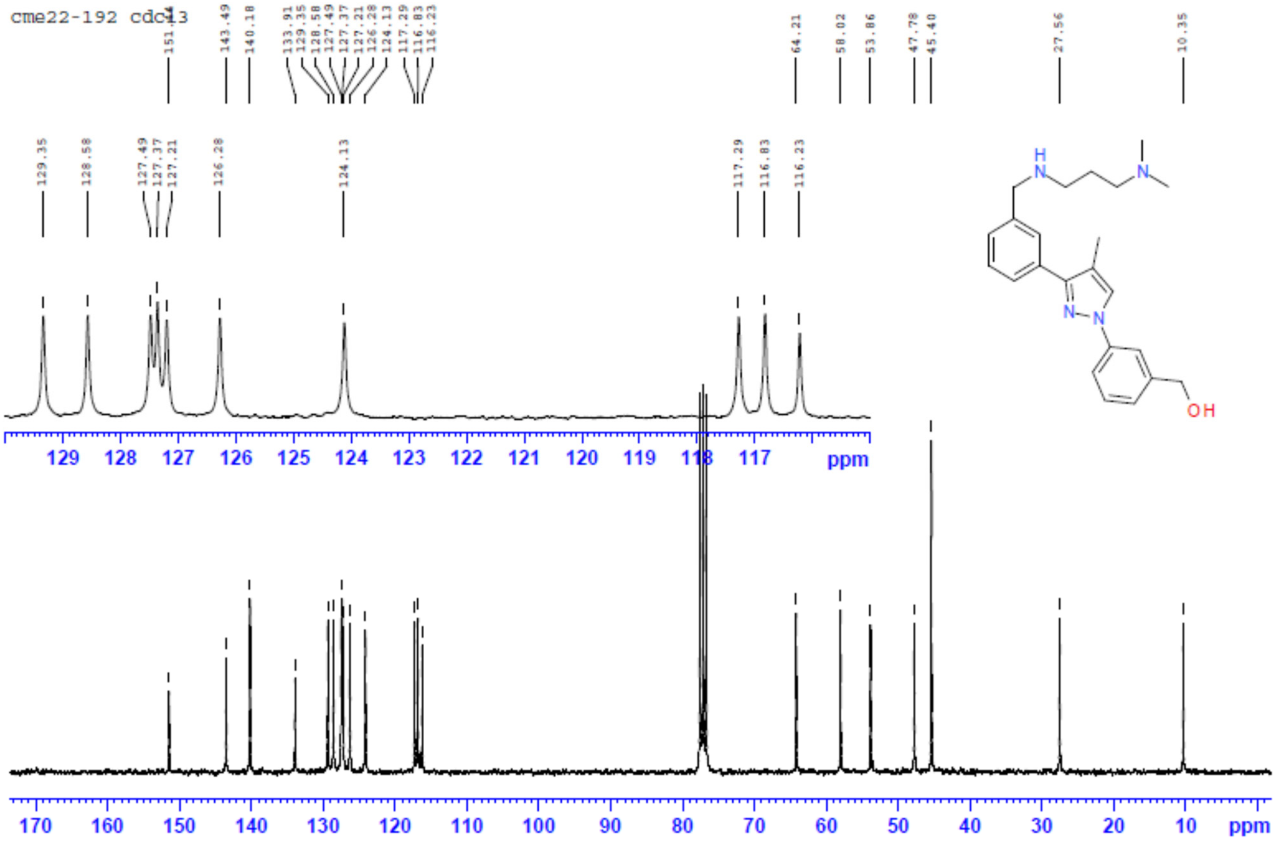

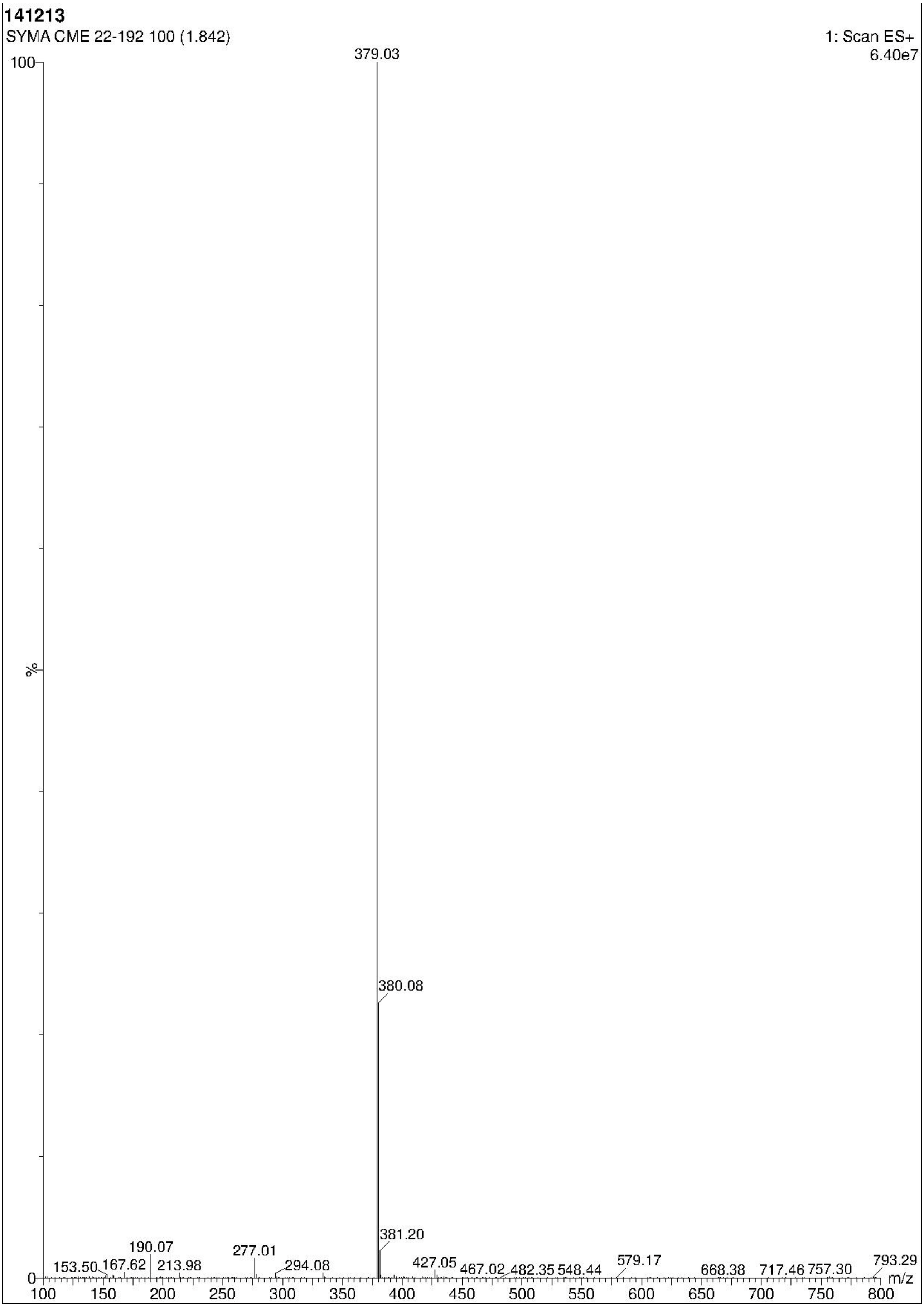
141213
SYMA CME 22-192 100 (1.842)
1: Scan ES+
6.40e7
379.03
380.08
381.20
153.50
167.62
190.07
213.98
277.01
294.08
427.05
467.02
482.35
548.44
579.17
668.38
717.46
757.30
793.29
%
m/z
100
0
100 150 200 250 300 350 400 450 500 550 600 650 700 750 800

(3-{3-[4-({[3-(dimethylamino)propyl](methyl)amino}methyl)phenyl]-4-methyl-1*H*-pyrazol-1-yl}phenyl)methanol (**55**)

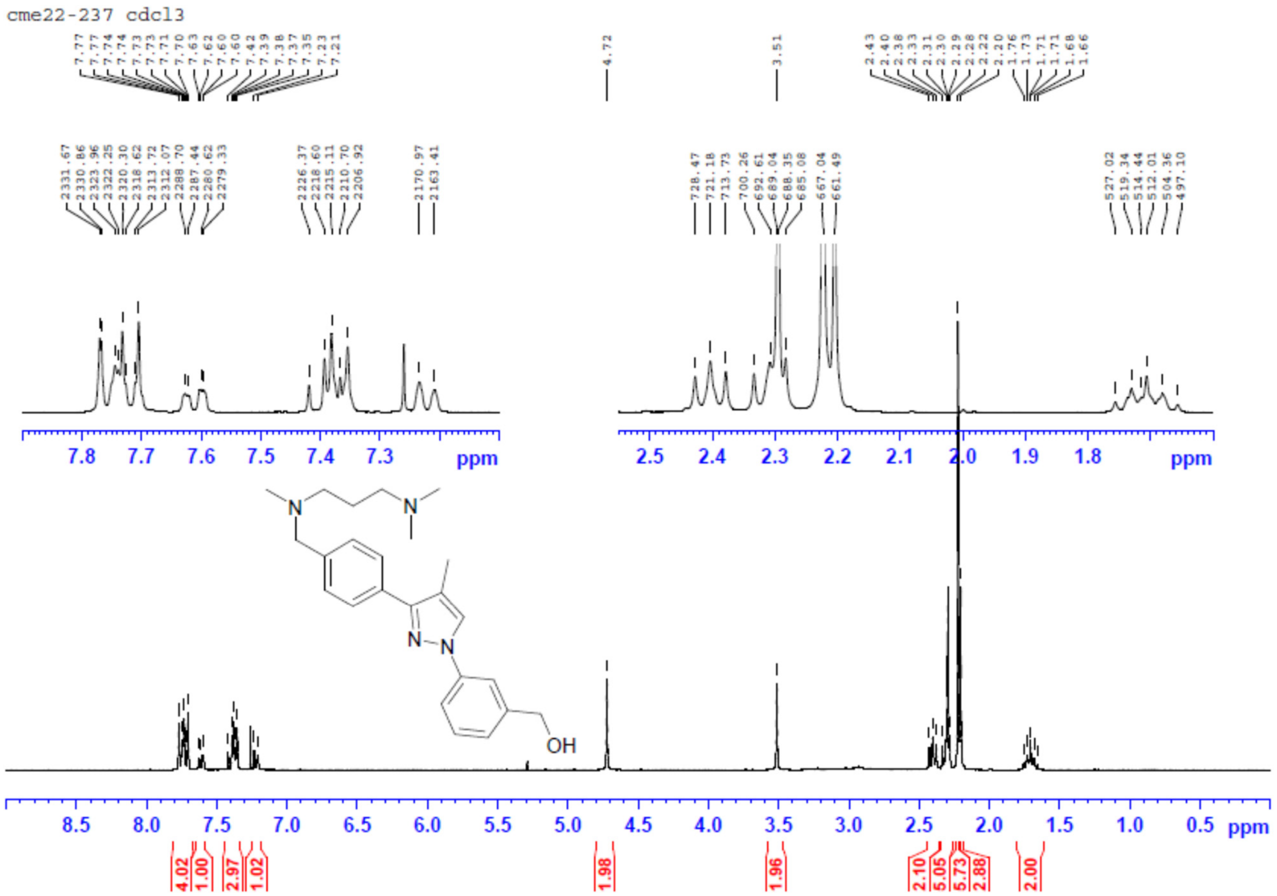

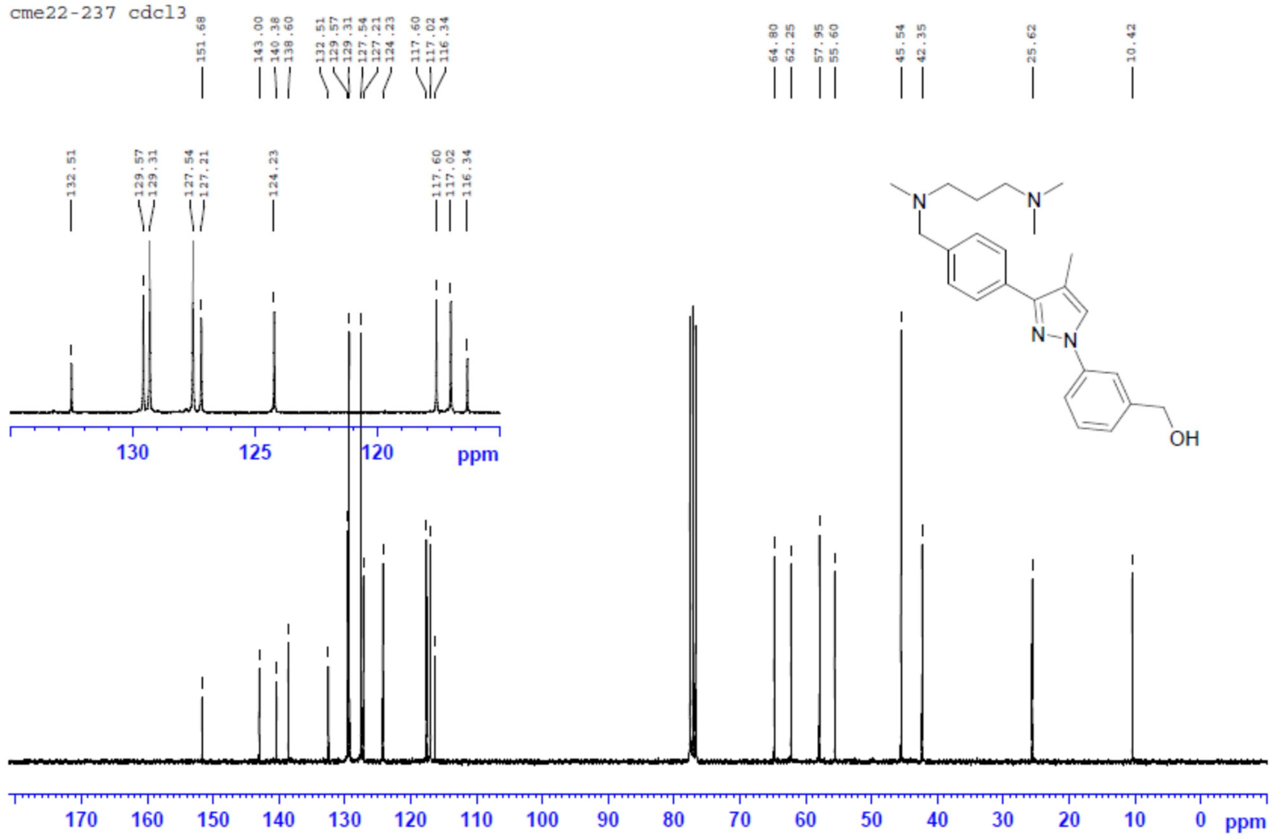

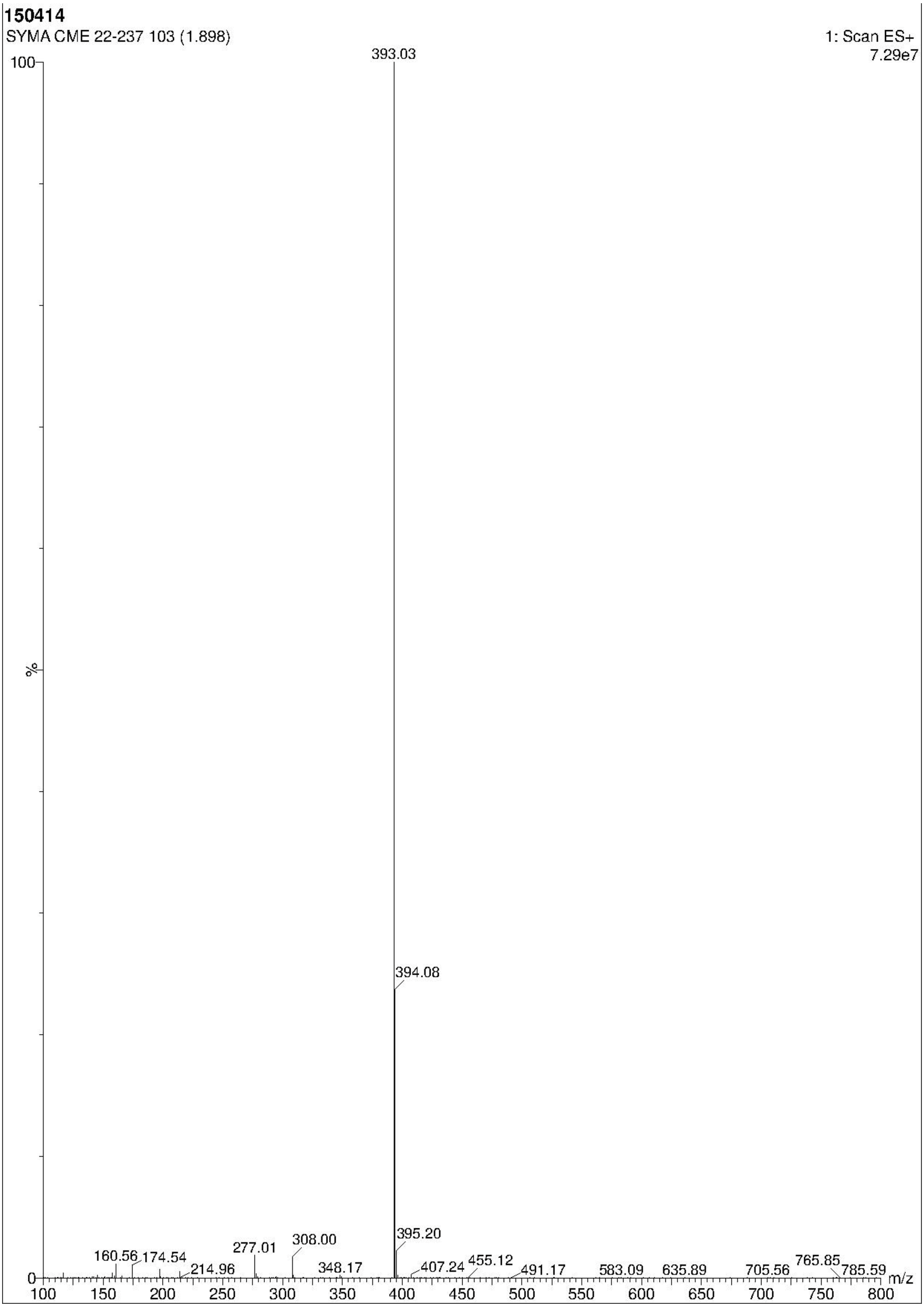

150414
SYMA CME 22-237 103 (1.898)
1: Scan ES+
7.29e7
393.03
394.08
395.20
160.56
174.54
214.96
277.01
308.00
348.17
407.24
455.12
491.17
583.09
635.89
705.56
765.85
785.59
%
m/z
100
0
100 150 200 250 300 350 400 450 500 550 600 650 700 750 800

[3-[3-[3-[[3-(dimethylamino)propyl-methyl-amino]methyl]phenyl]-4-methyl-pyrazol-1-yl]phenyl]methanol (**56**)

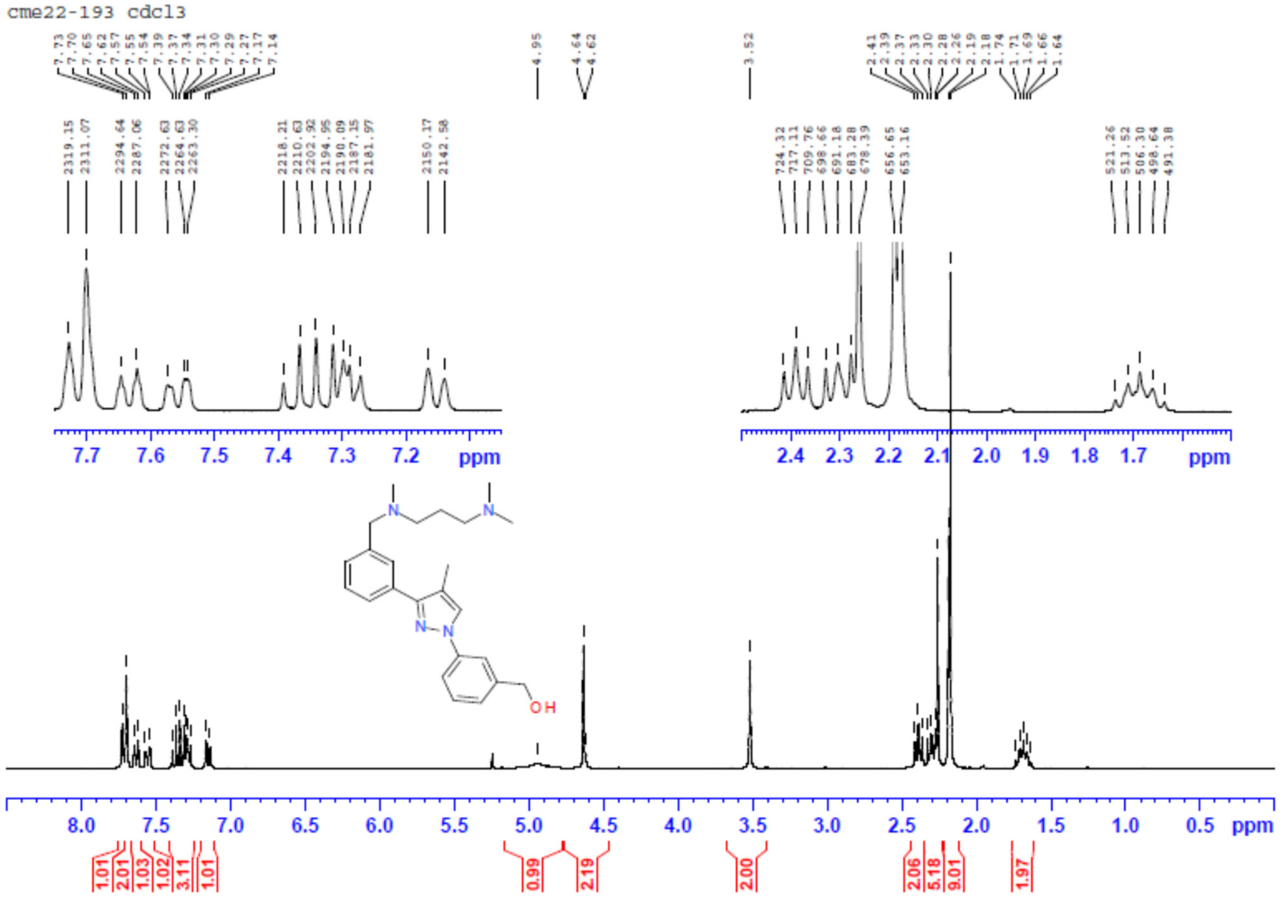

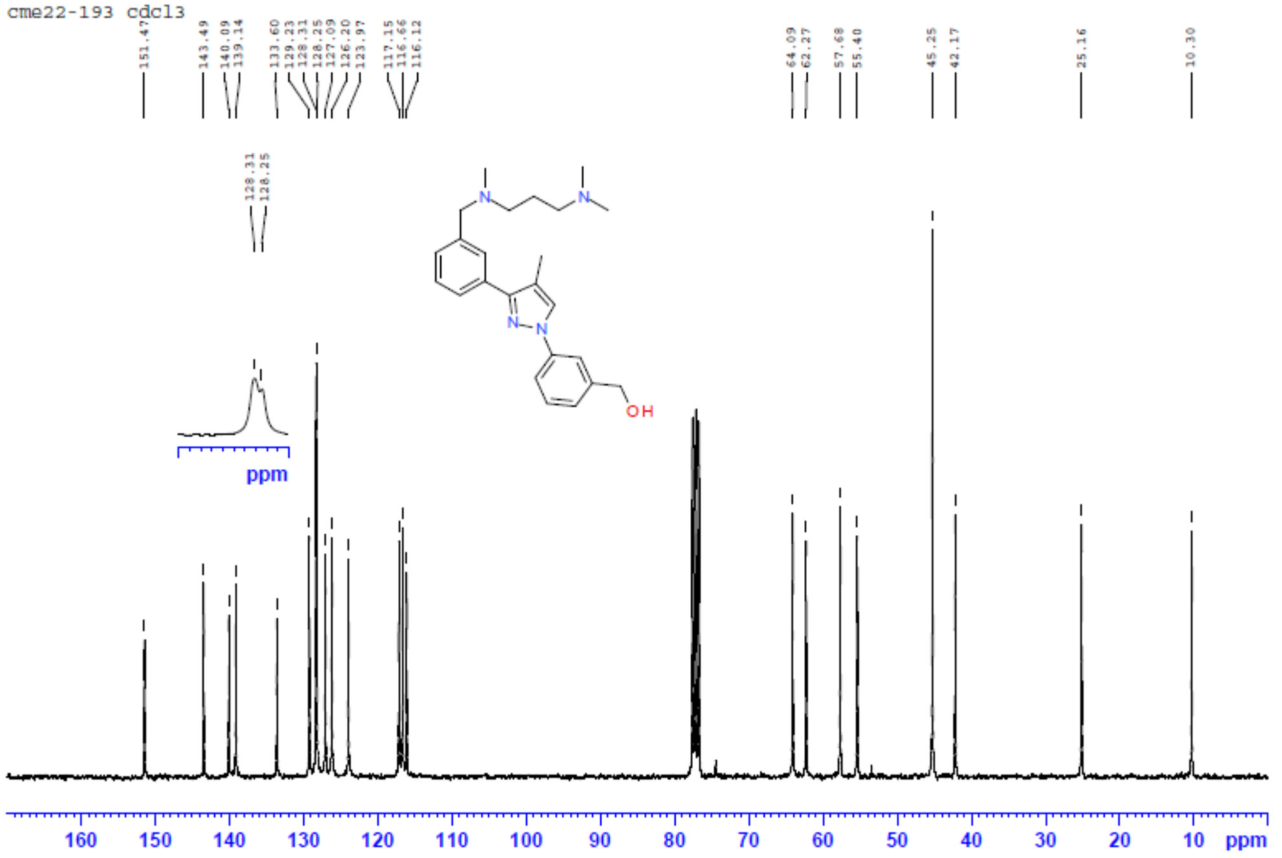

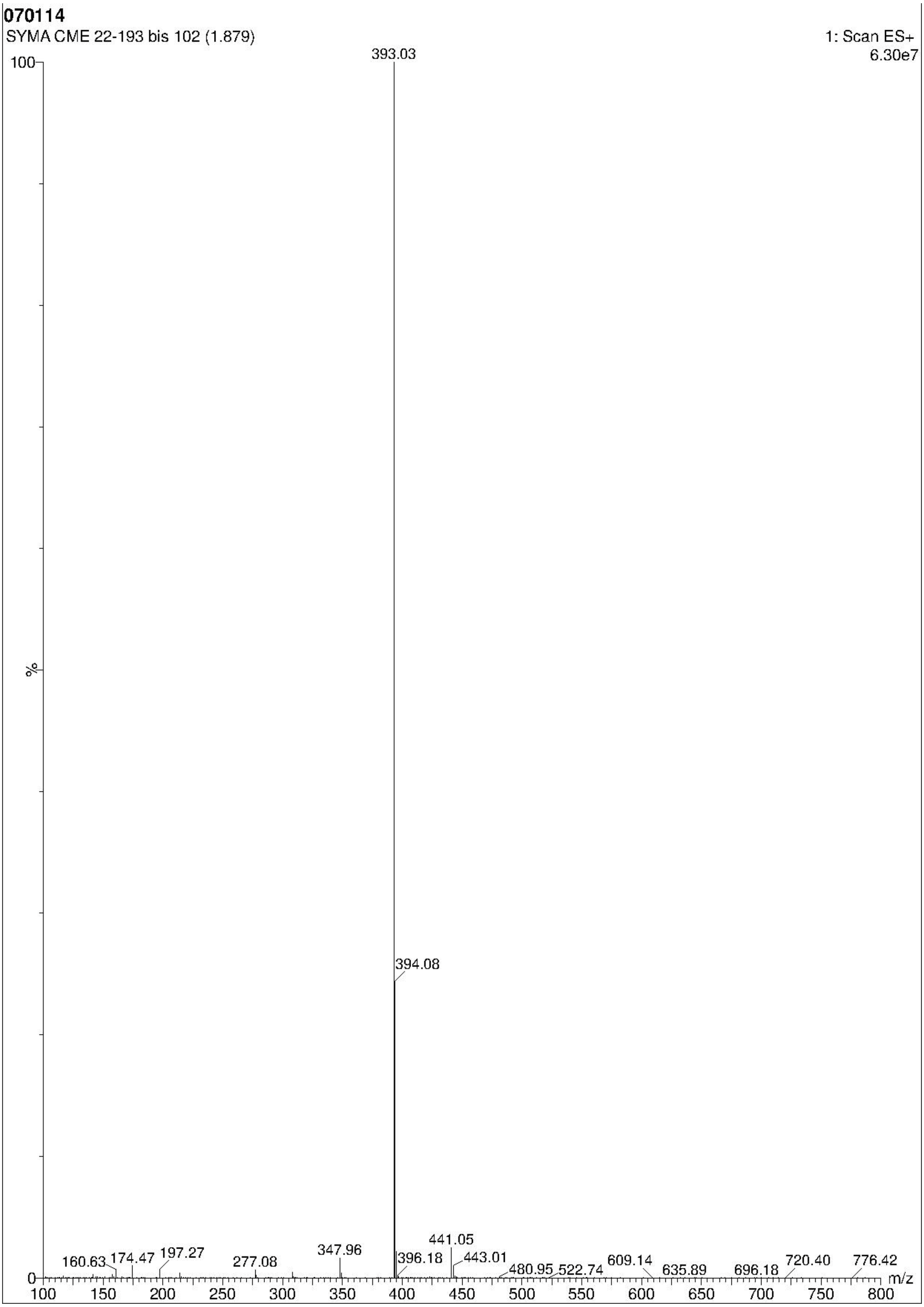

070114
SYMA CME 22-193 bis 102 (1.879)
1: Scan ES+
6.30e7
393.03
394.08
160.63
174.47
197.27
277.08
347.96
396.18
441.05
443.01
480.95
522.74
609.14
635.89
696.18
720.40
776.42
%
m/z

3-[3-[4-(dimethylaminomethyl)phenyl]-4-methyl-pyrazol-1-yl]benzaldehyde (**57**)

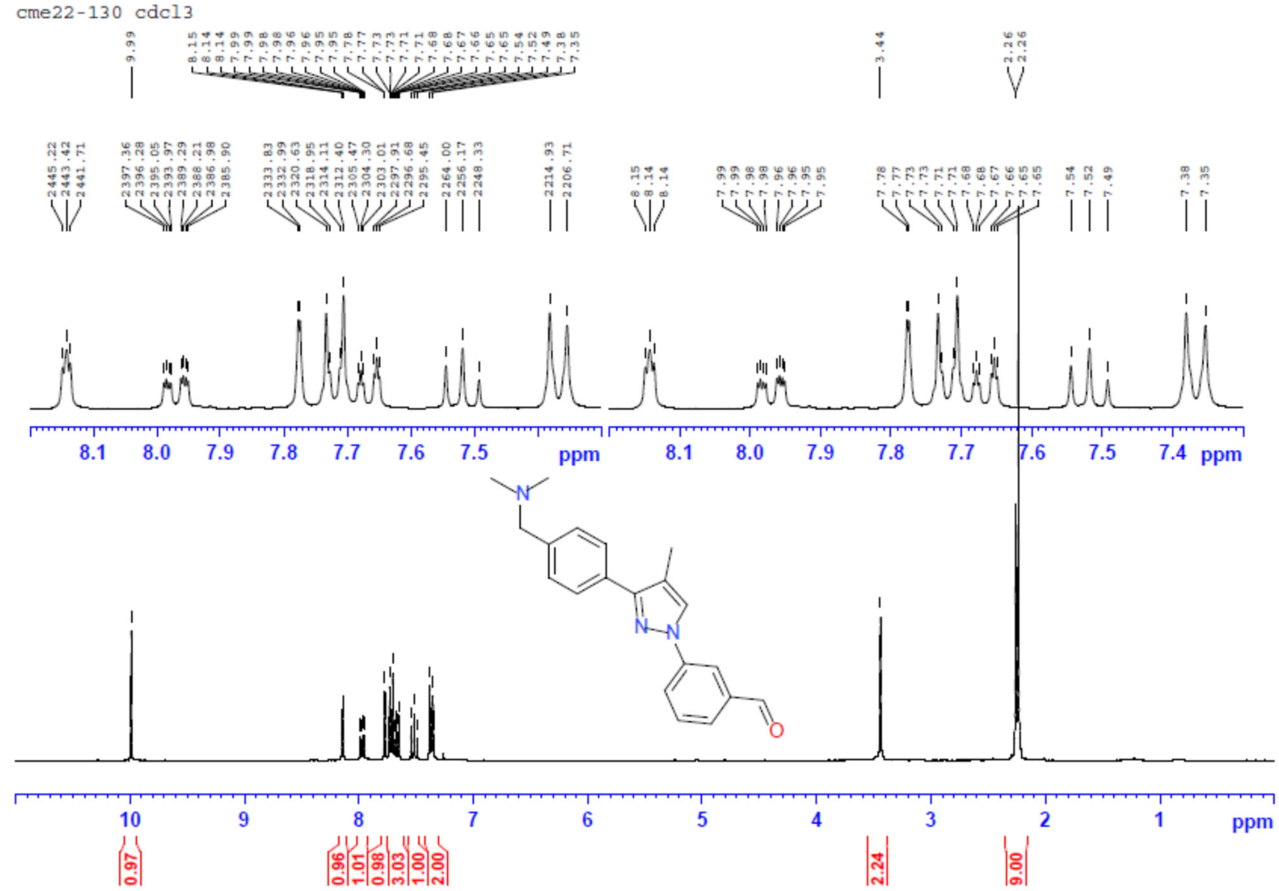

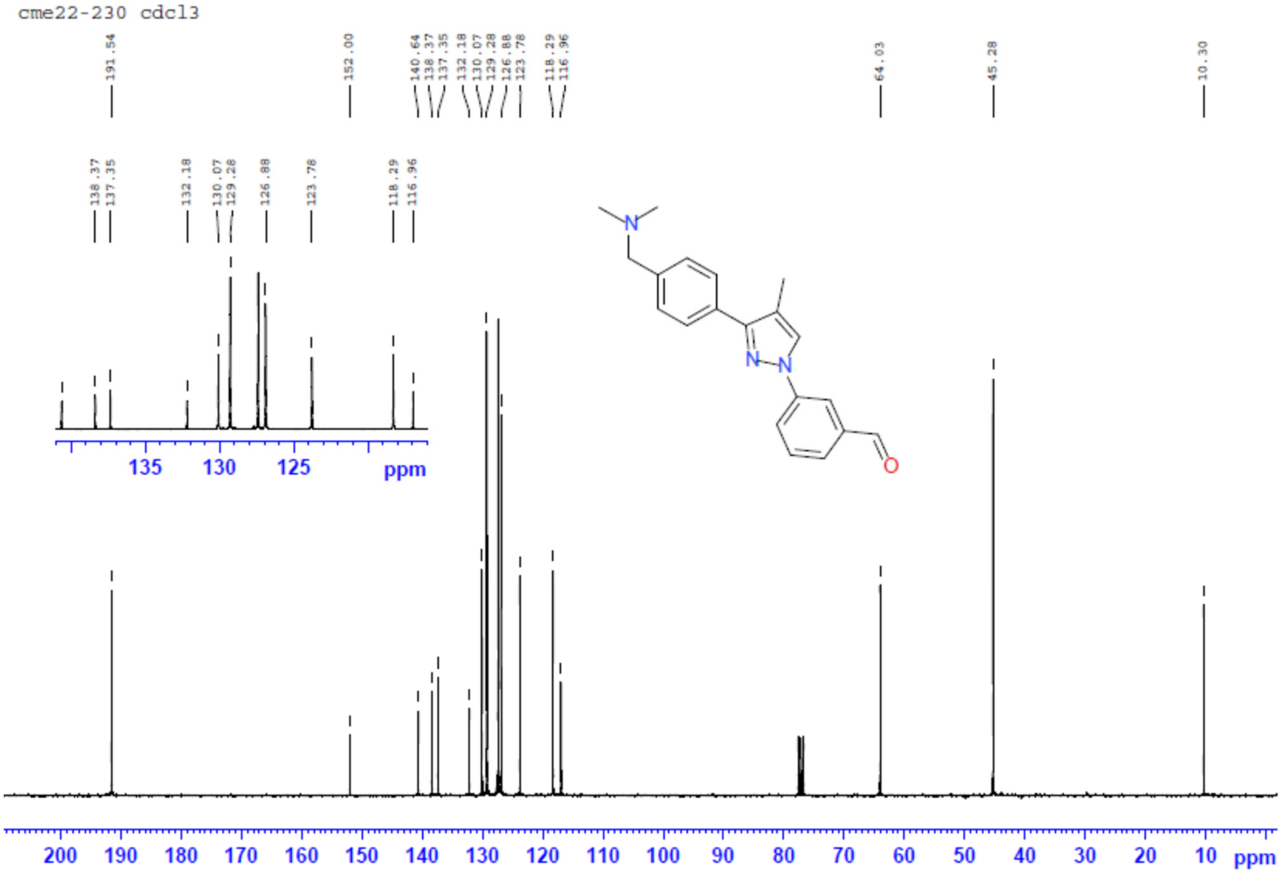

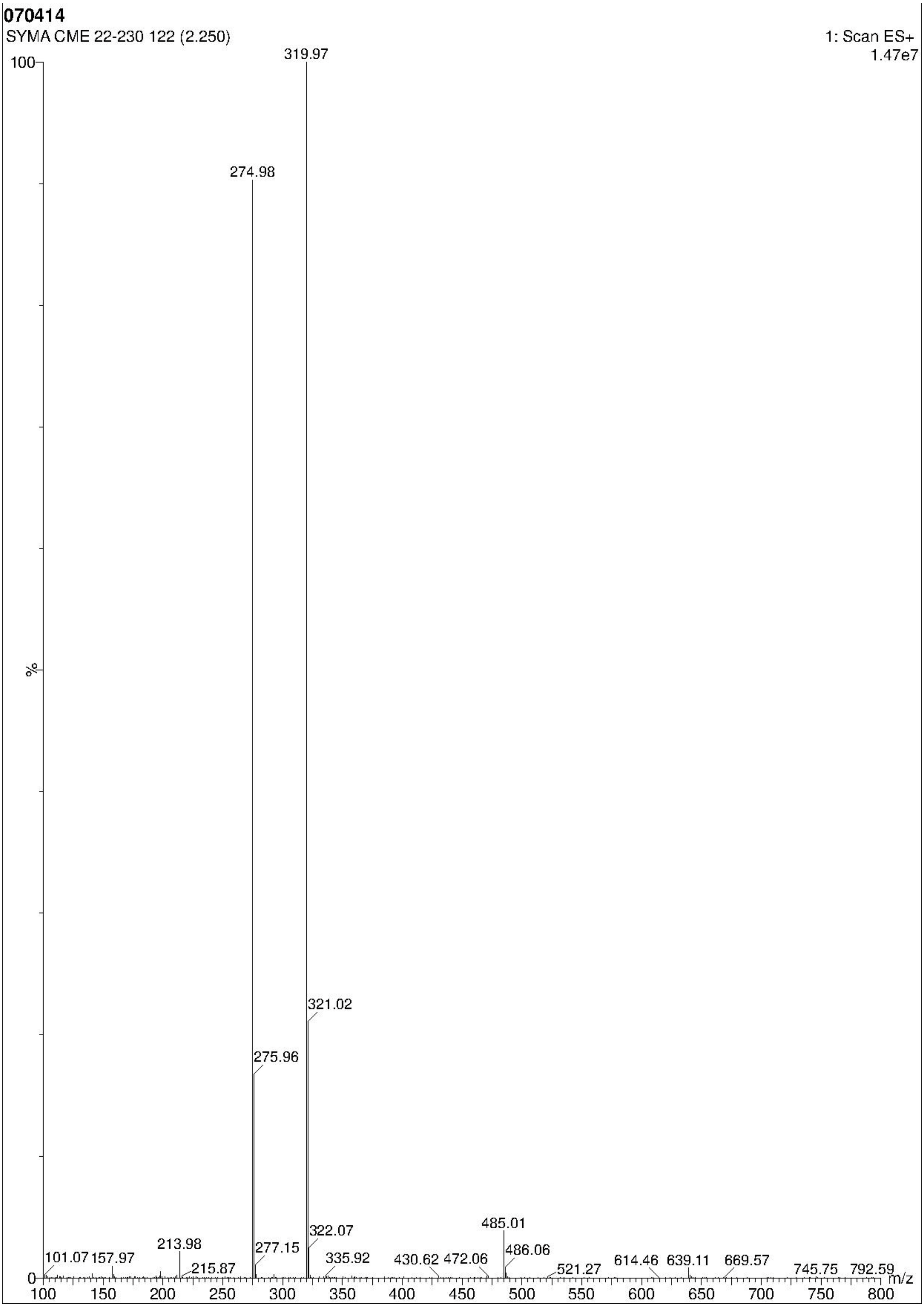
070414
SYMA CME 22-230 122 (2.250)
1: Scan ES+
1.47e7
100
%
0
319.97
274.98
321.02
275.96
322.07
277.15
485.01
486.06
213.98
215.87
101.07
157.97
335.92
430.62
472.06
521.27
614.46
639.11
669.57
745.75
792.59
100
150
200
250
300
350
400
450
500
550
600
650
700
750
800
m/z

3-[3-[3-(dimethylaminomethyl)phenyl]-4-methyl-pyrazol-1-yl]benzaldehyde (**58**)

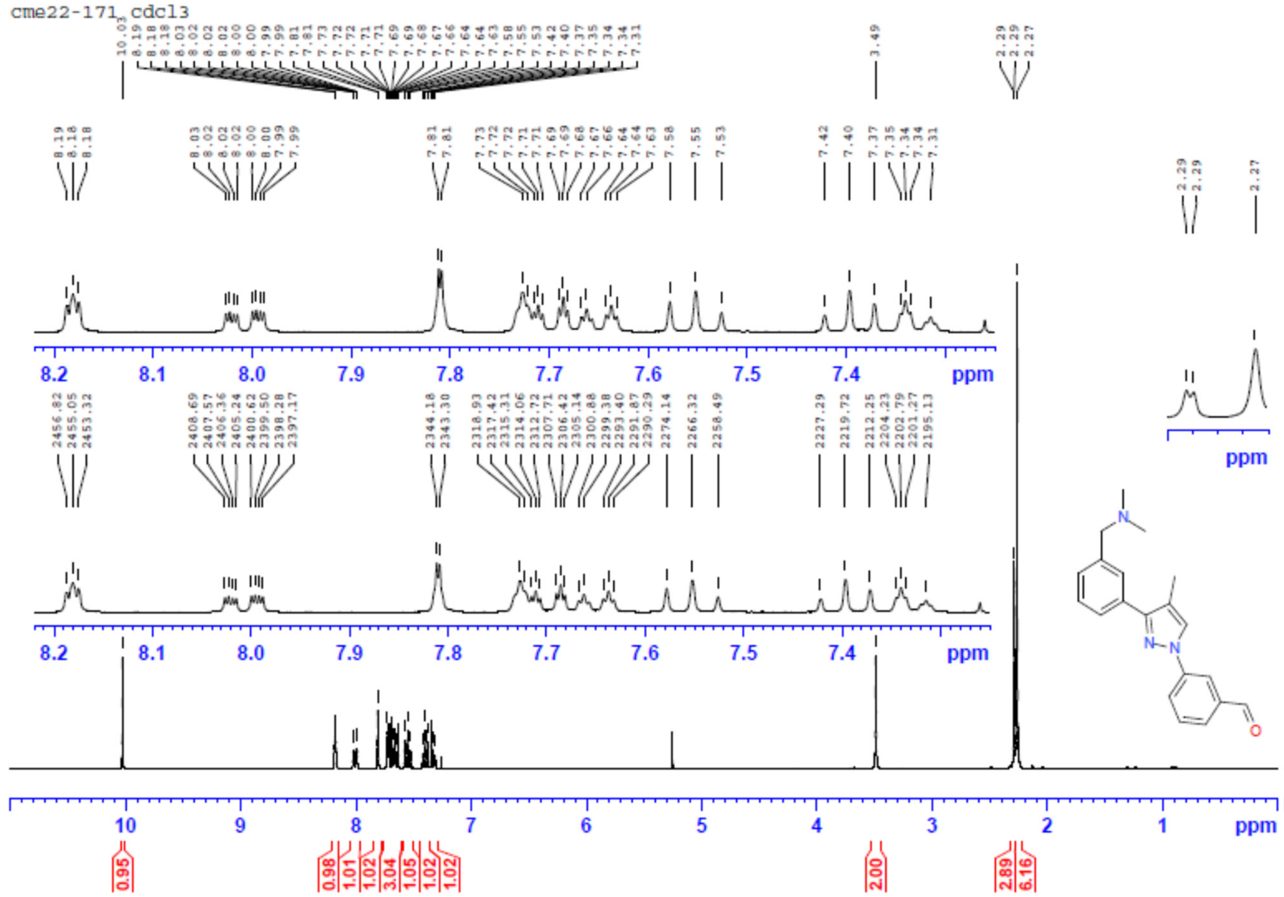

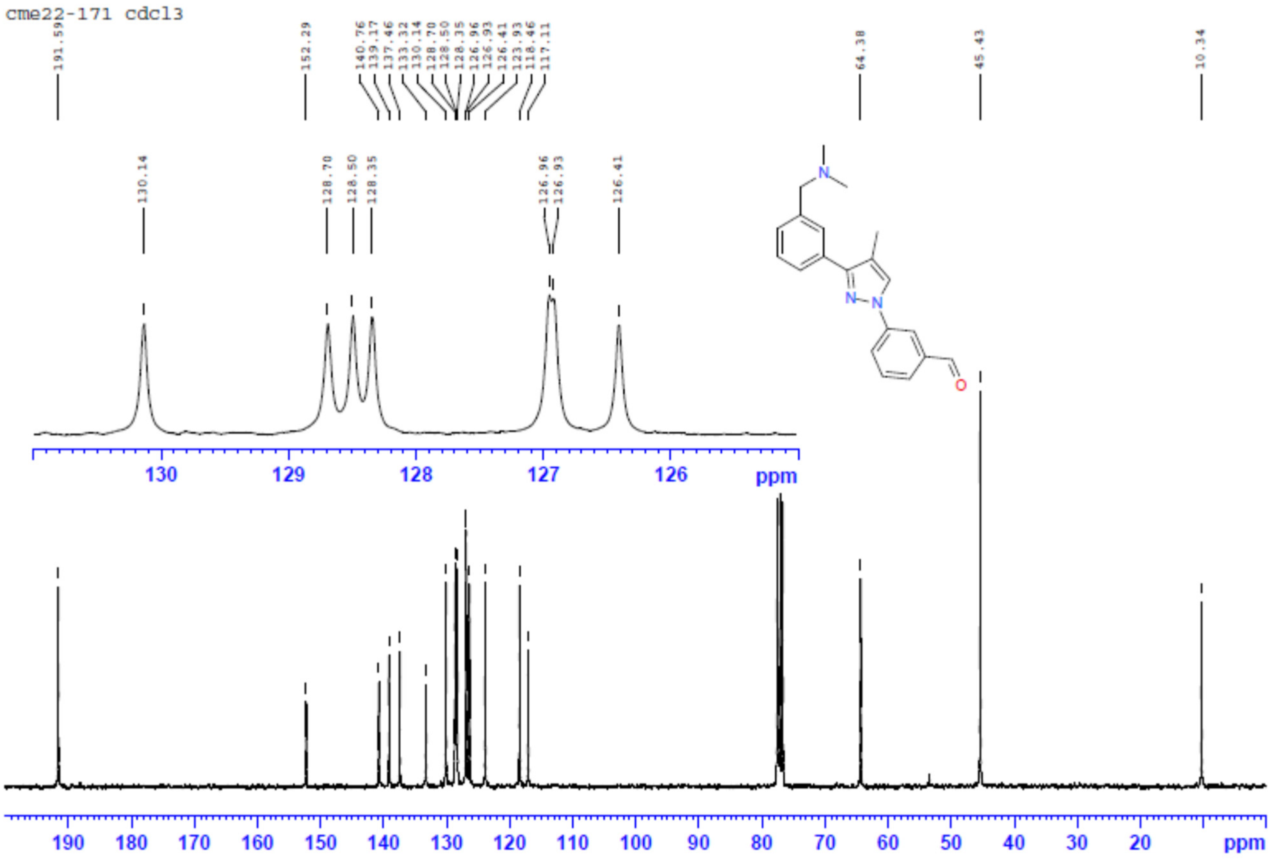

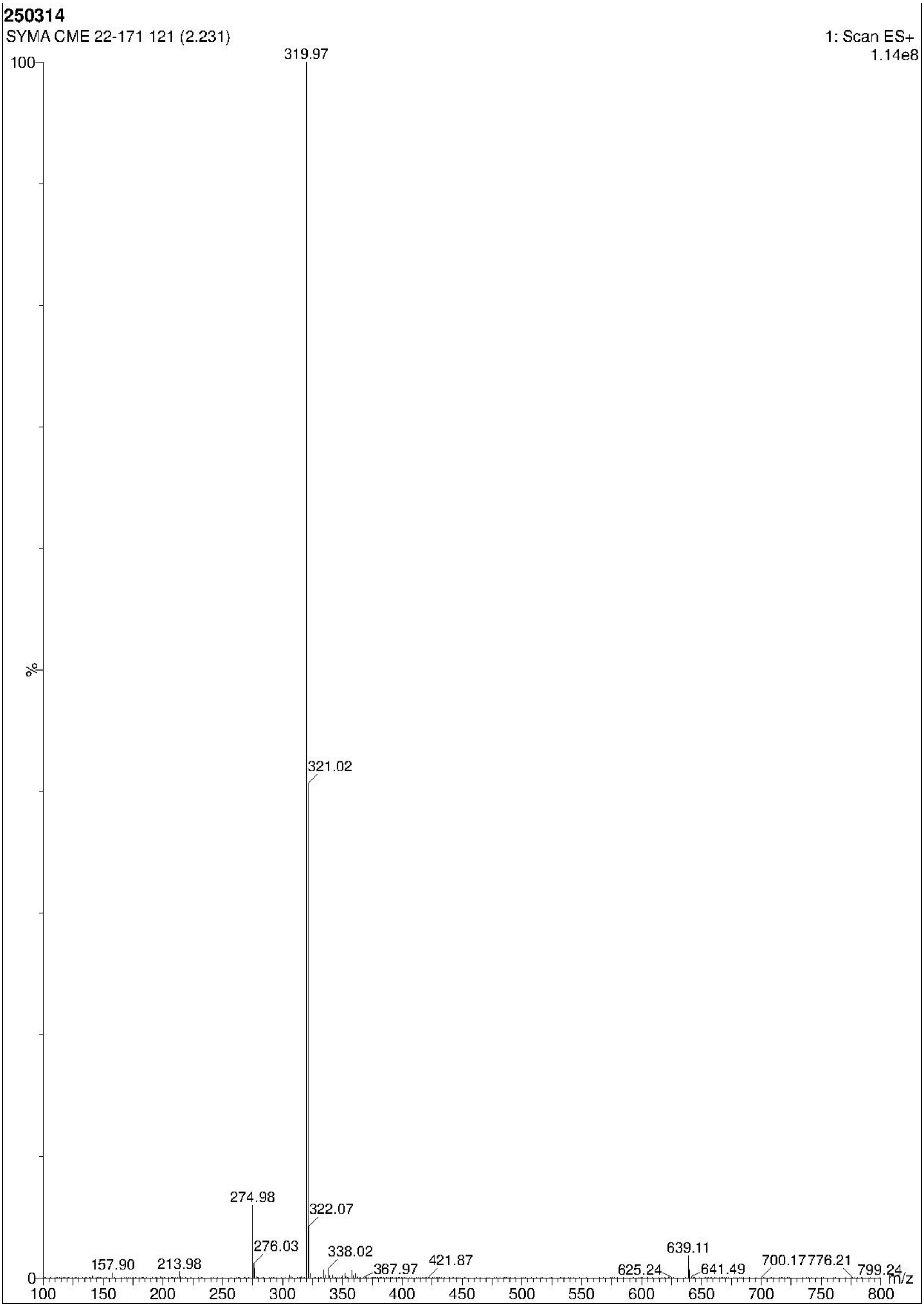
250314
SYMA CME 22-171 121 (2.231)
1: Scan ES+
1.14e8
100
%
0
319.97
321.02
274.98
322.07
276.03
338.02
157.90
213.98
367.97
421.87
625.24
639.11
641.49
700.17
776.21
799.24
m/z
100
150
200
250
300
350
400
450
500
550
600
650
700
750
800

3-{3-[4-({[3-(dimethylamino)propyl](methyl)amino}methyl)phenyl]-4-methyl-1*H*-pyrazol-1-yl}benzaldehyde (**59**)

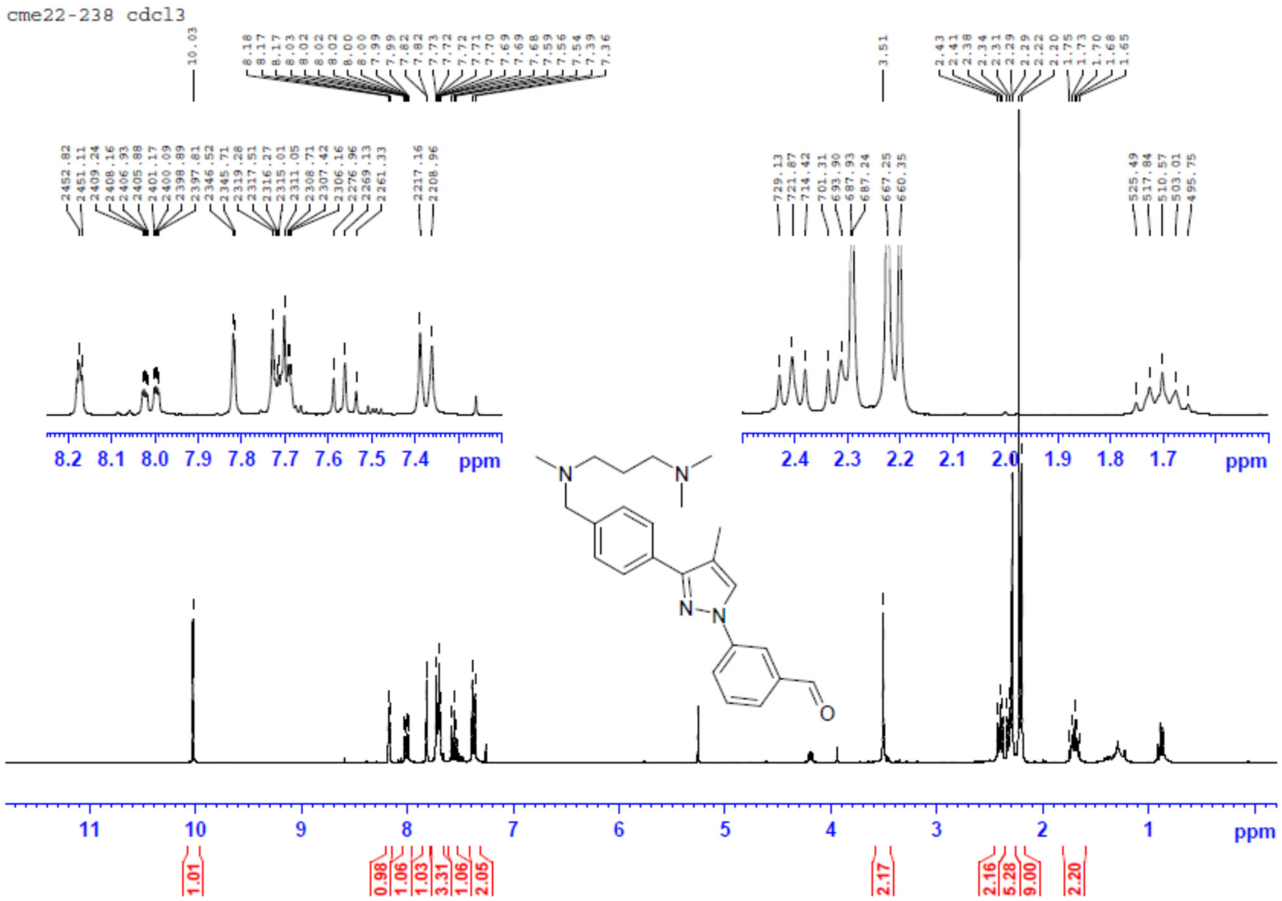

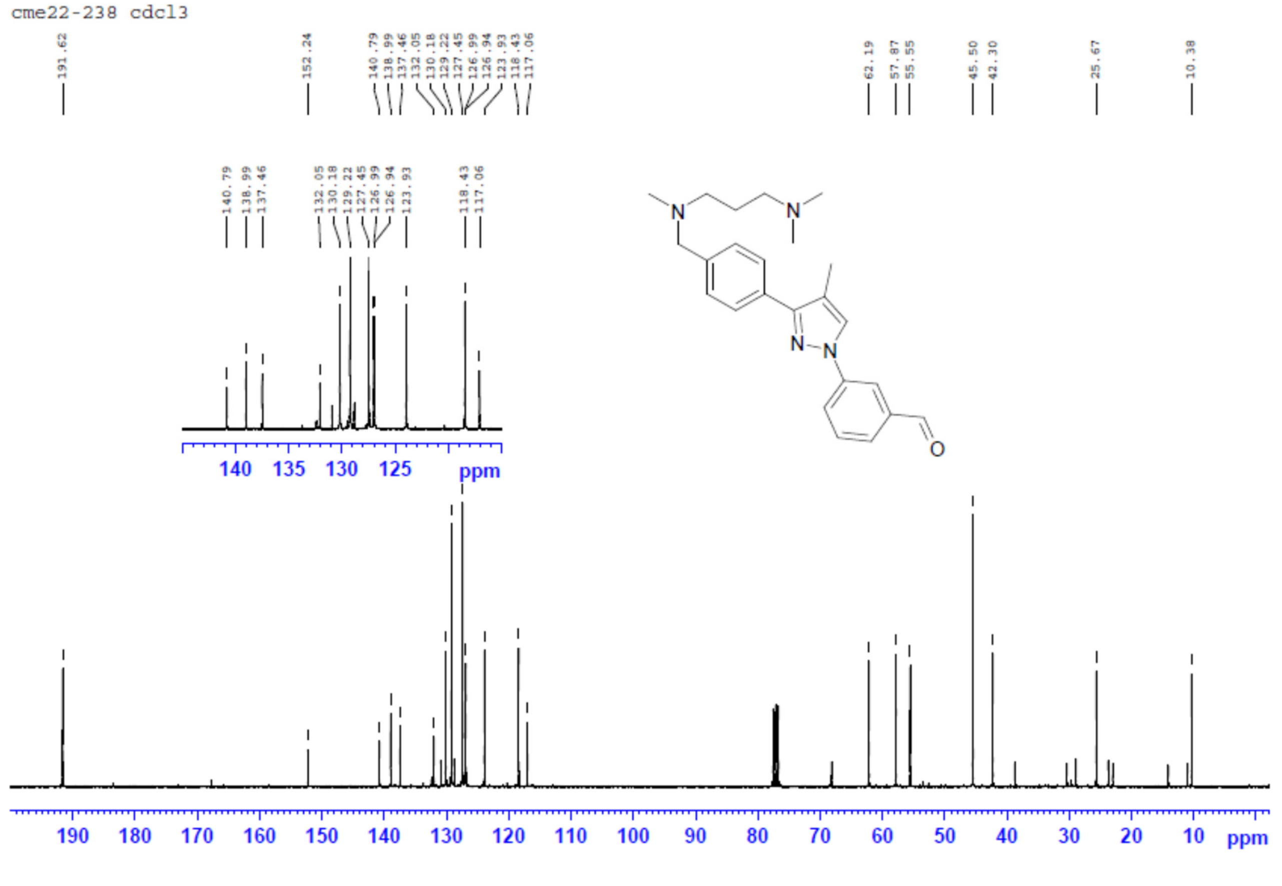

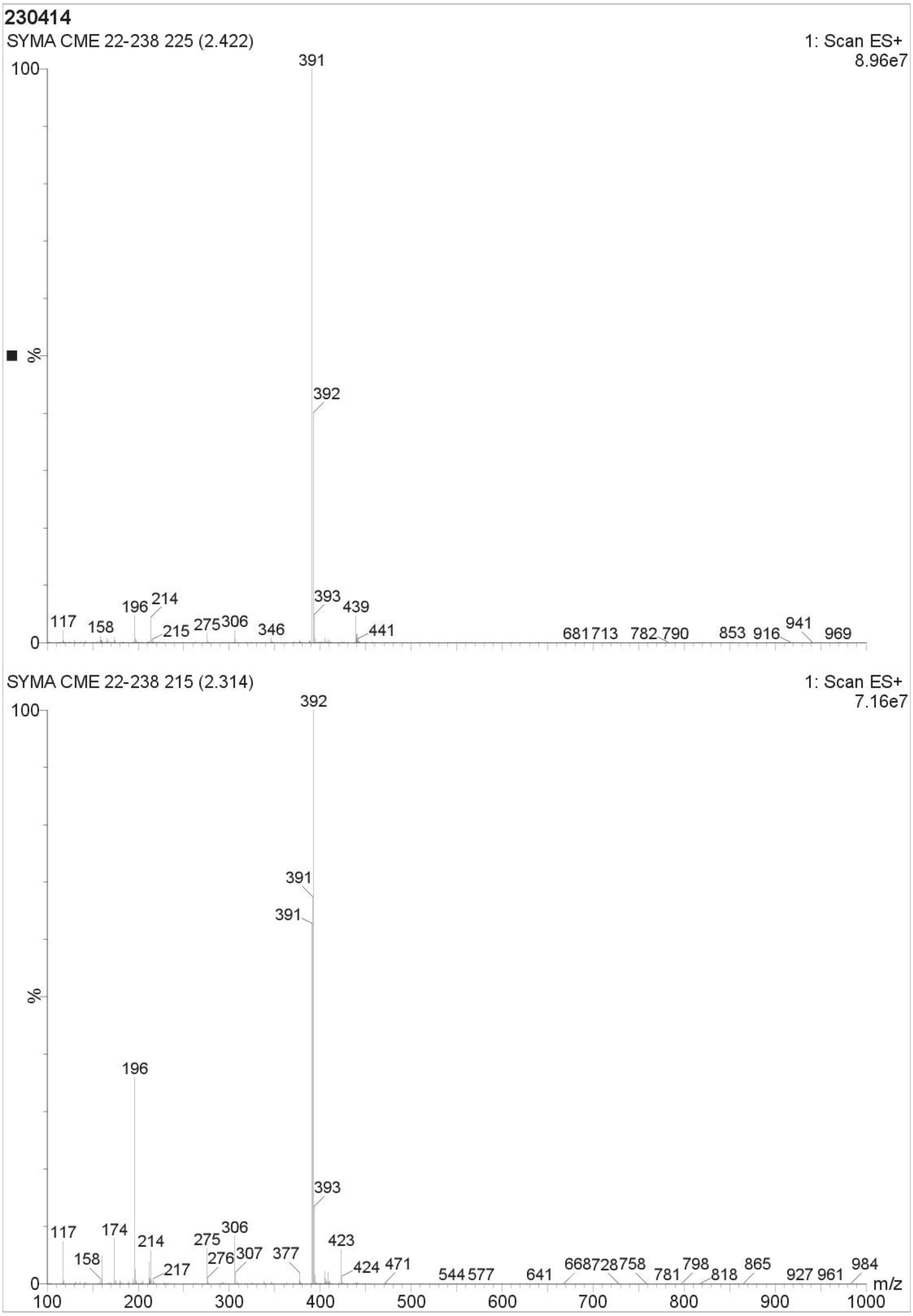
230414
SYMA CME 22-238 225 (2.422)
1: Scan ES+
8.96e7
391
392
393
439
441
117
158
196
214
215
275
306
346
681
713
782
790
853
916
941
969
%
100
0
SYMA CME 22-238 215 (2.314)
1: Scan ES+
7.16e7
392
391
391
196
393
117
174
158
214
217
275
276
306
307
377
423
424
471
544
577
641
668
728
758
781
798
818
865
927
961
984
m/z
100
200
300
400
500
600
700
800
900
1000

3-[3-[3-[[3-(dimethylamino)propyl-methyl-amino]methyl]phenyl]-4-methyl-pyrazol-1-yl]benzaldehyde (**60**)

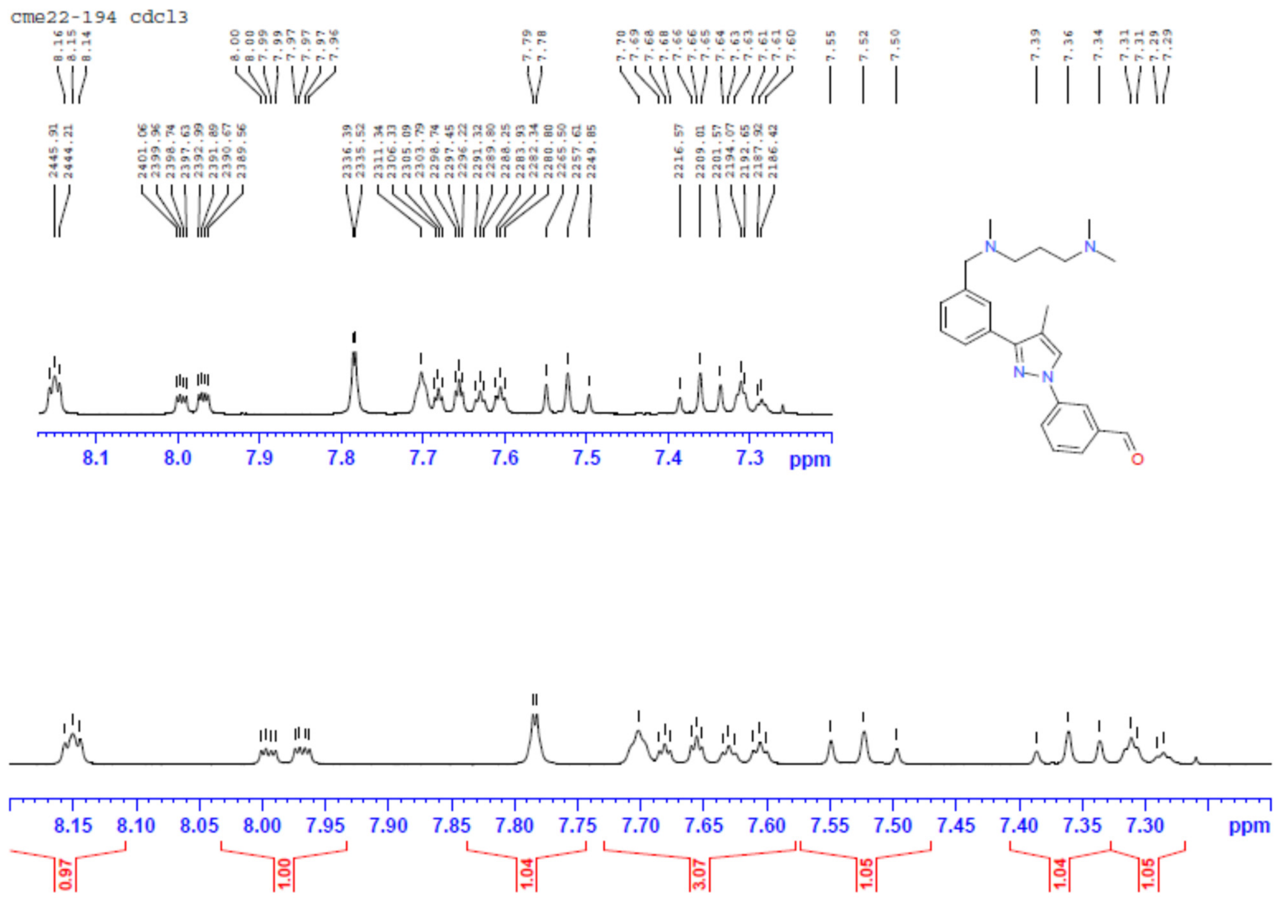

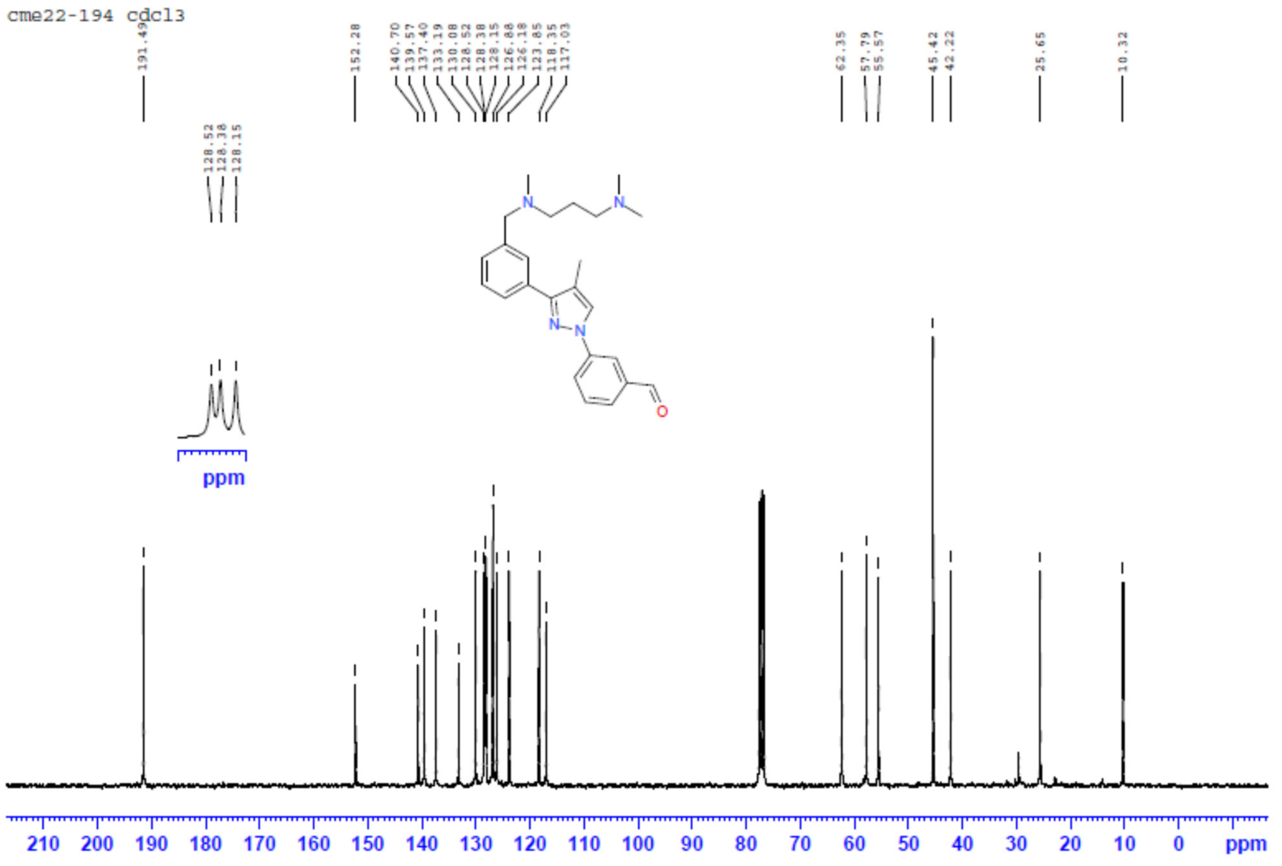

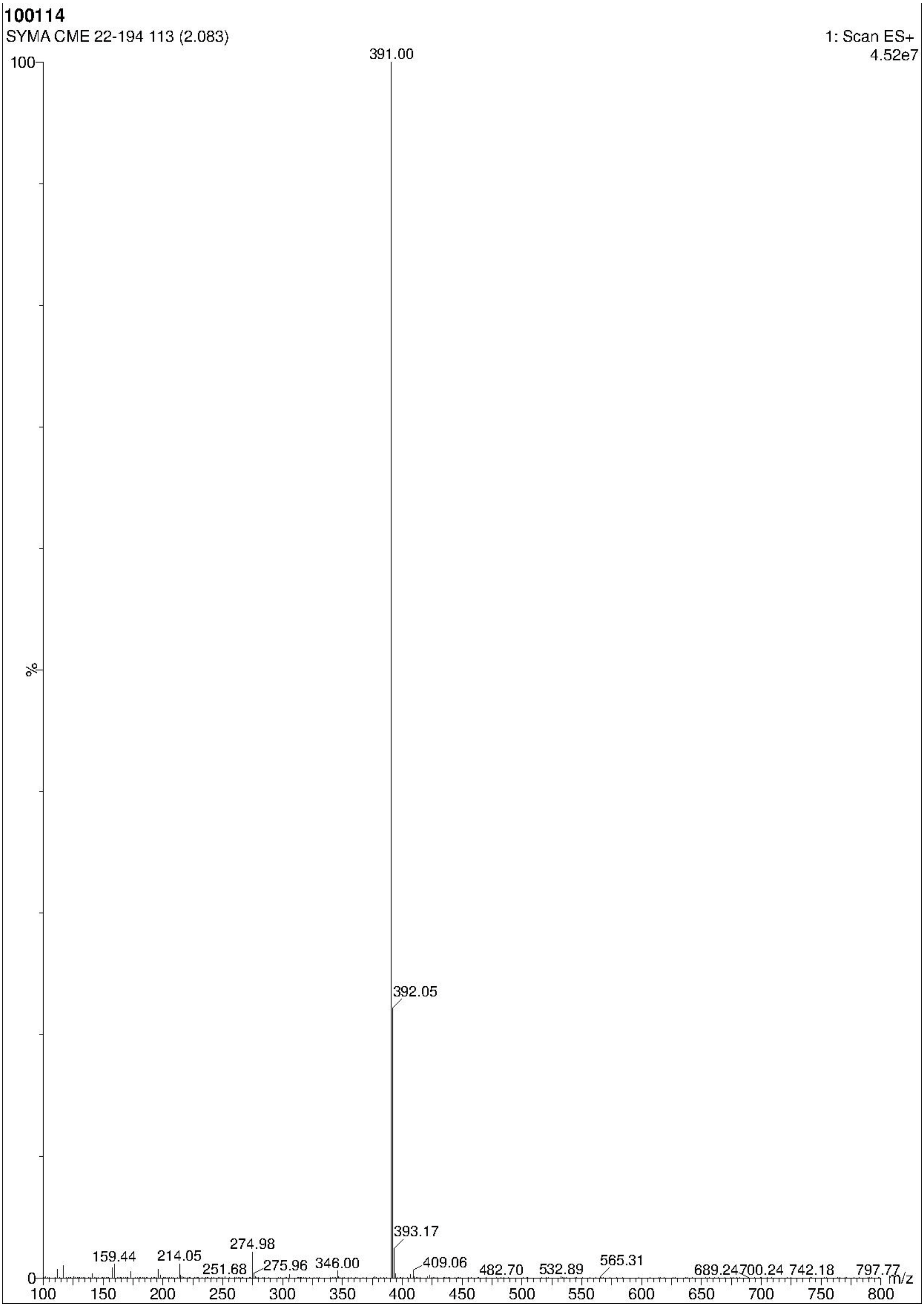
100114
SYMA CME 22-194 113 (2.083)
1: Scan ES+
4.52e7
391.00
392.05
393.17
409.06
159.44
214.05
251.68
274.98
275.96
346.00
482.70
532.89
565.31
689.24
700.24
742.18
797.77
%
m/z
100
0
100 150 200 250 300 350 400 450 500 550 600 650 700 750 800

{[4-(1-{3-[(dimethylamino)methyl]phenyl}-4-methyl-1*H*-pyrazol-3-yl)phenyl]methyl}dimethylamine (**61**)

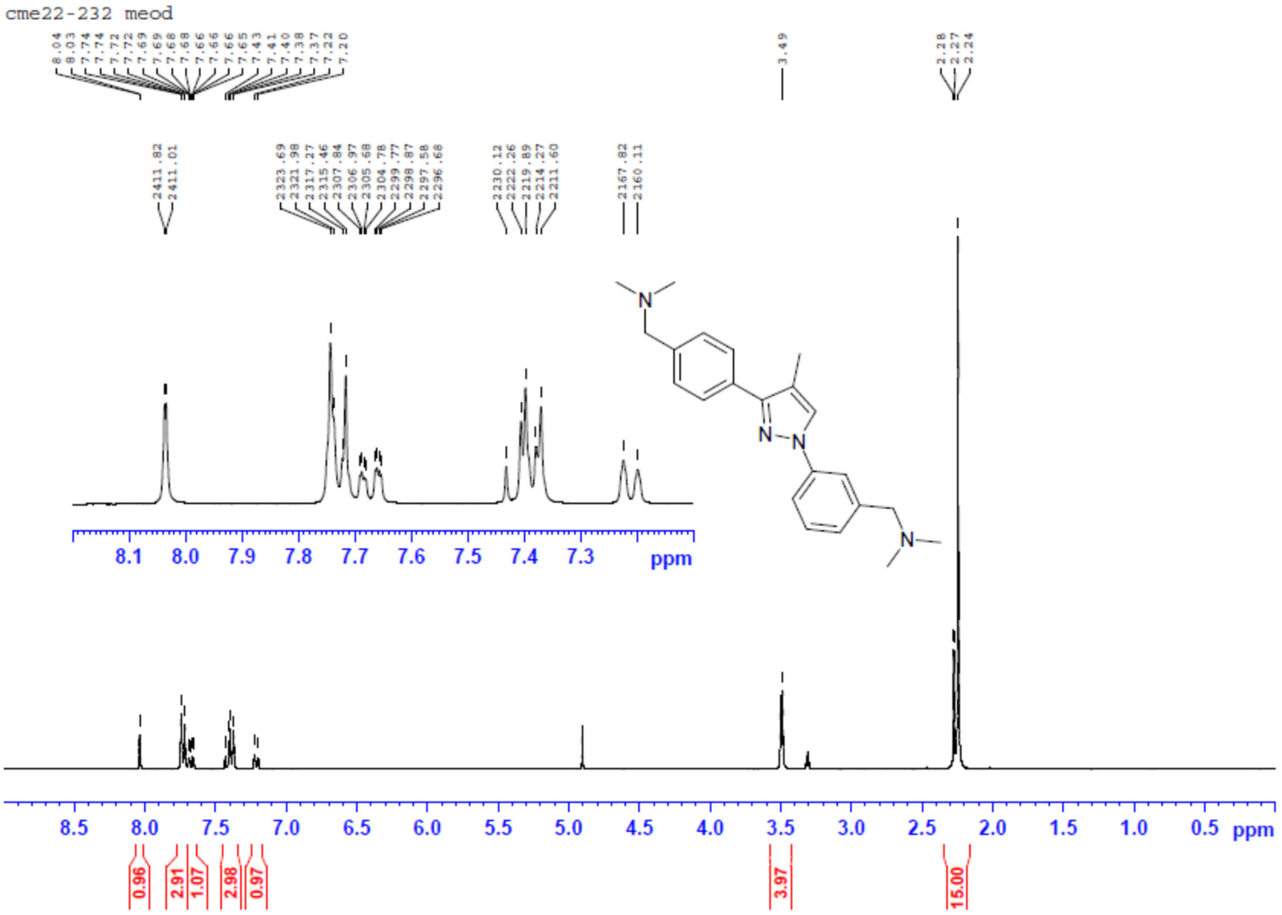

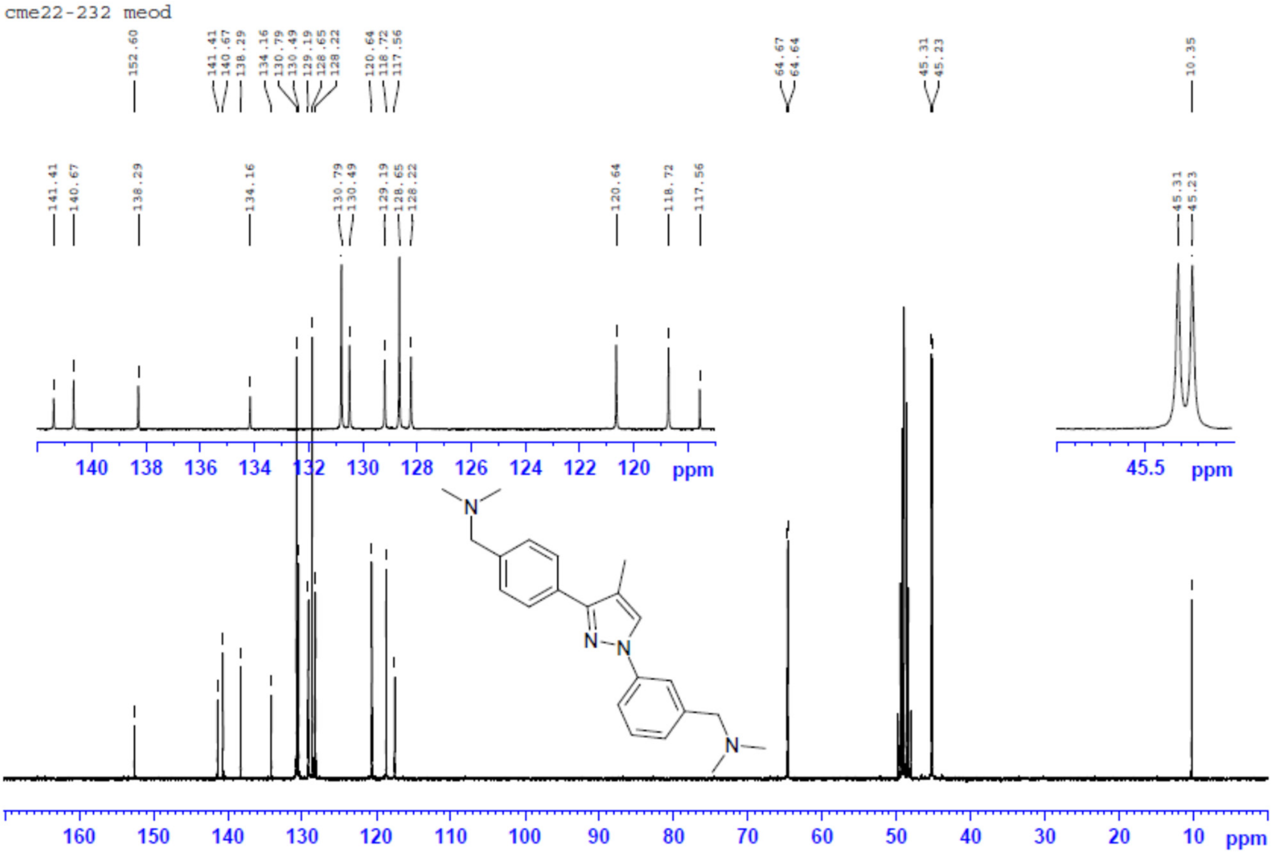

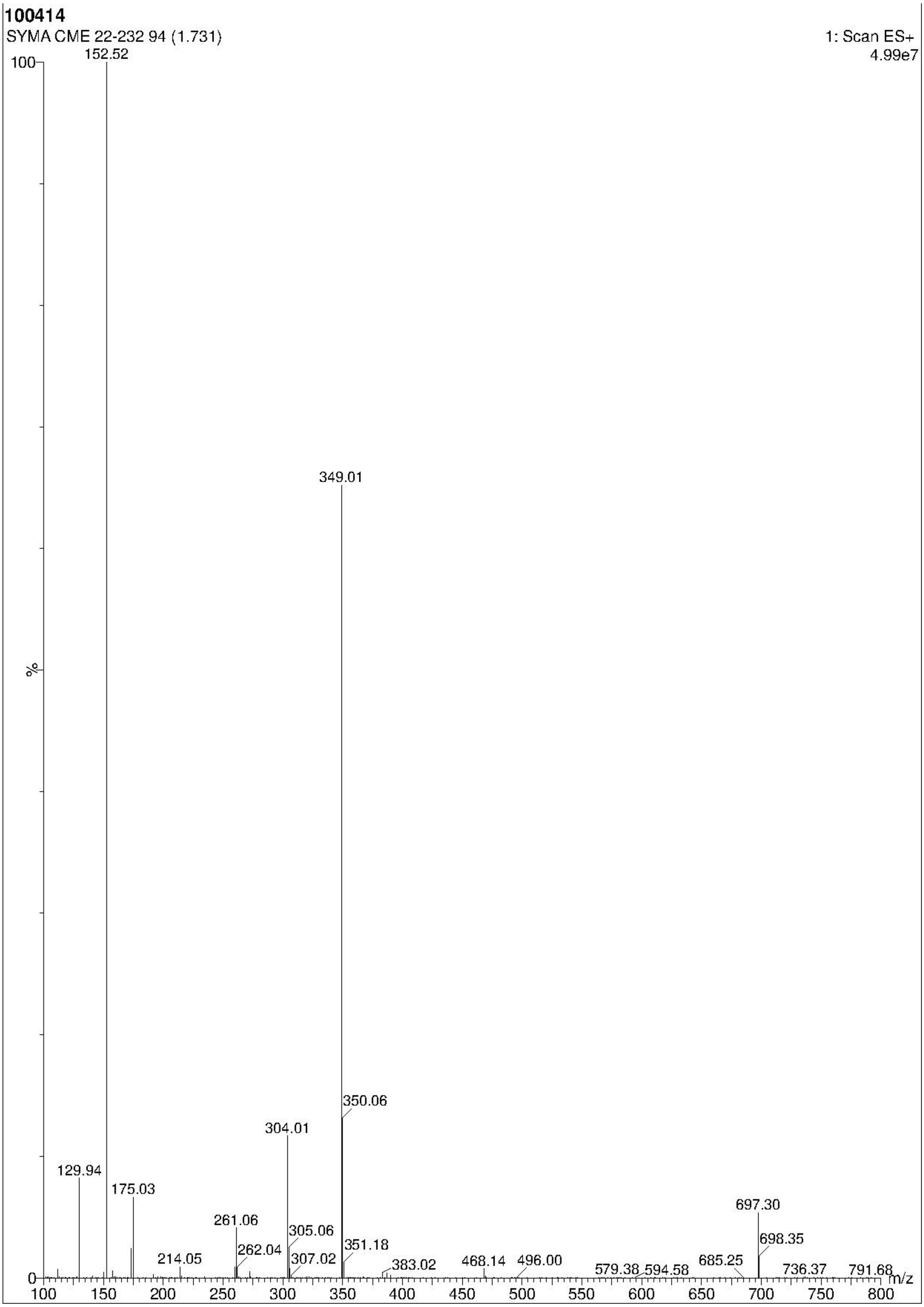
100414
SYMA CME 22-232 94 (1.731)
1: Scan ES+
4.99e7
100
%
0
152.52
349.01
350.06
304.01
129.94
175.03
261.06
305.06
262.04
214.05
307.02
351.18
383.02
468.14
496.00
579.38
594.58
685.25
697.30
698.35
736.37
791.68
m/z
100
150
200
250
300
350
400
450
500
550
600
650
700
750
800

HPLC C4-column

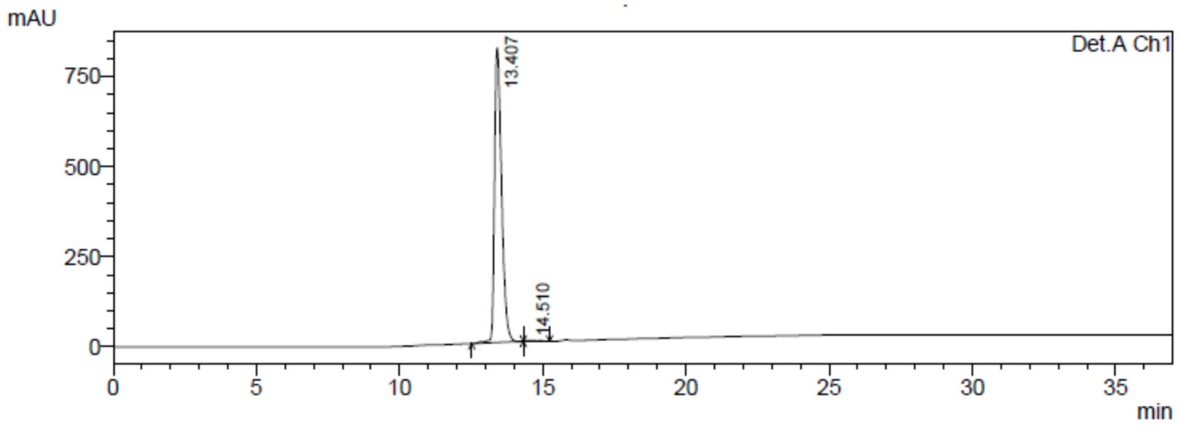

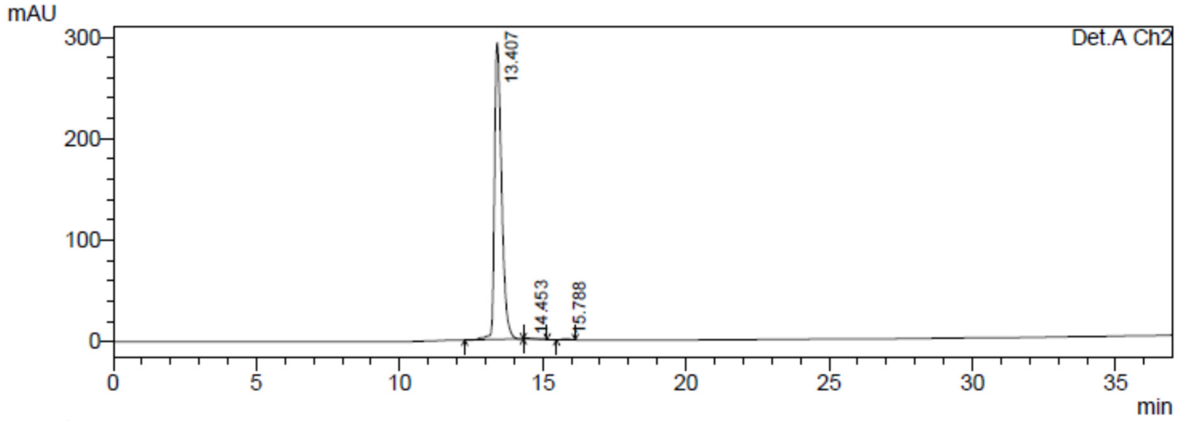

1 Det.A Ch1/215nm
2 Det.A Ch2/254nm

PeakTable

Detector A Ch1 215nm

Peak#	Ret. Time	Area	Area %
1	13.407	13947523	99.617
2	14.510	53660	0.383
Total		14001183	100.000

PeakTable

Detector A Ch2 254nm

Peak#	Ret. Time	Area	Area %
1	13.407	5019815	99.331
2	14.453	17056	0.338
3	15.788	16755	0.332
Total		5053627	100.000

HPLC C18-column

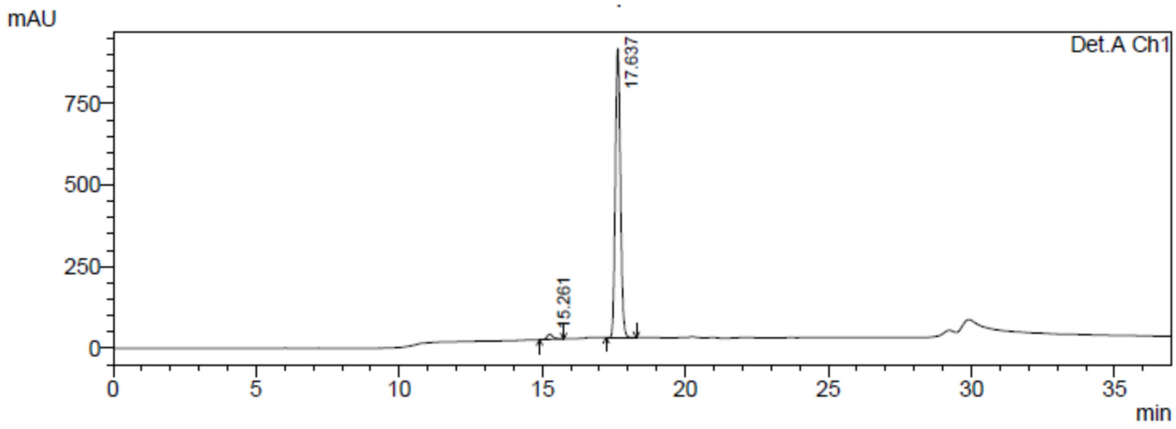

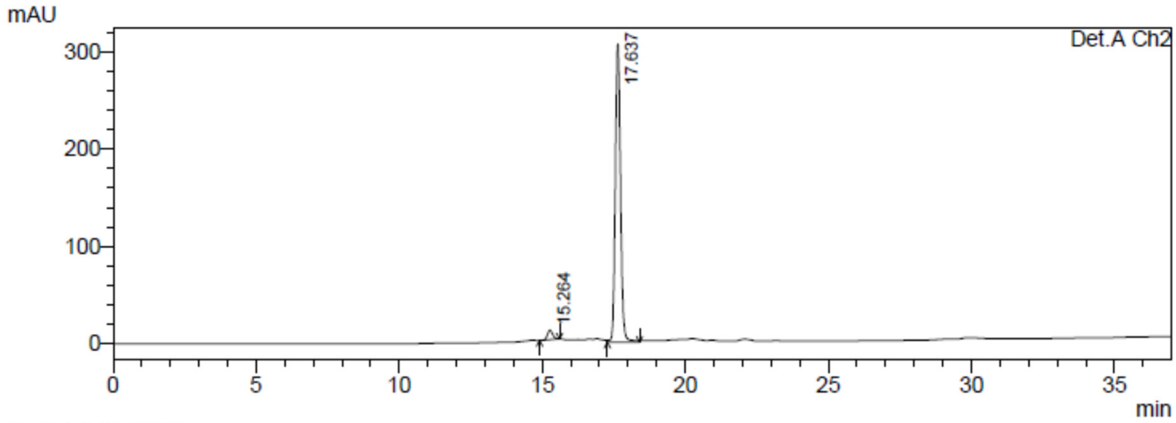

1 Det.A Ch1/215nm
2 Det.A Ch2/254nm

PeakTable

Detector A Ch1 215nm

Peak#	Ret. Time	Area	Area %
1	15.261	224790	2.002
2	17.637	11001590	97.998
Total		11226380	100.000

PeakTable

Detector A Ch2 254nm

Peak#	Ret. Time	Area	Area %
1	15.264	125039	3.122
2	17.637	3879807	96.878
Total		4004846	100.000

dimethyl({[4-(4-methyl-1-{3-[(4-methylpiperazin-1-yl)methyl]phenyl}-1*H*-pyrazol-3-yl)phenyl]methyl})amine (**62**)

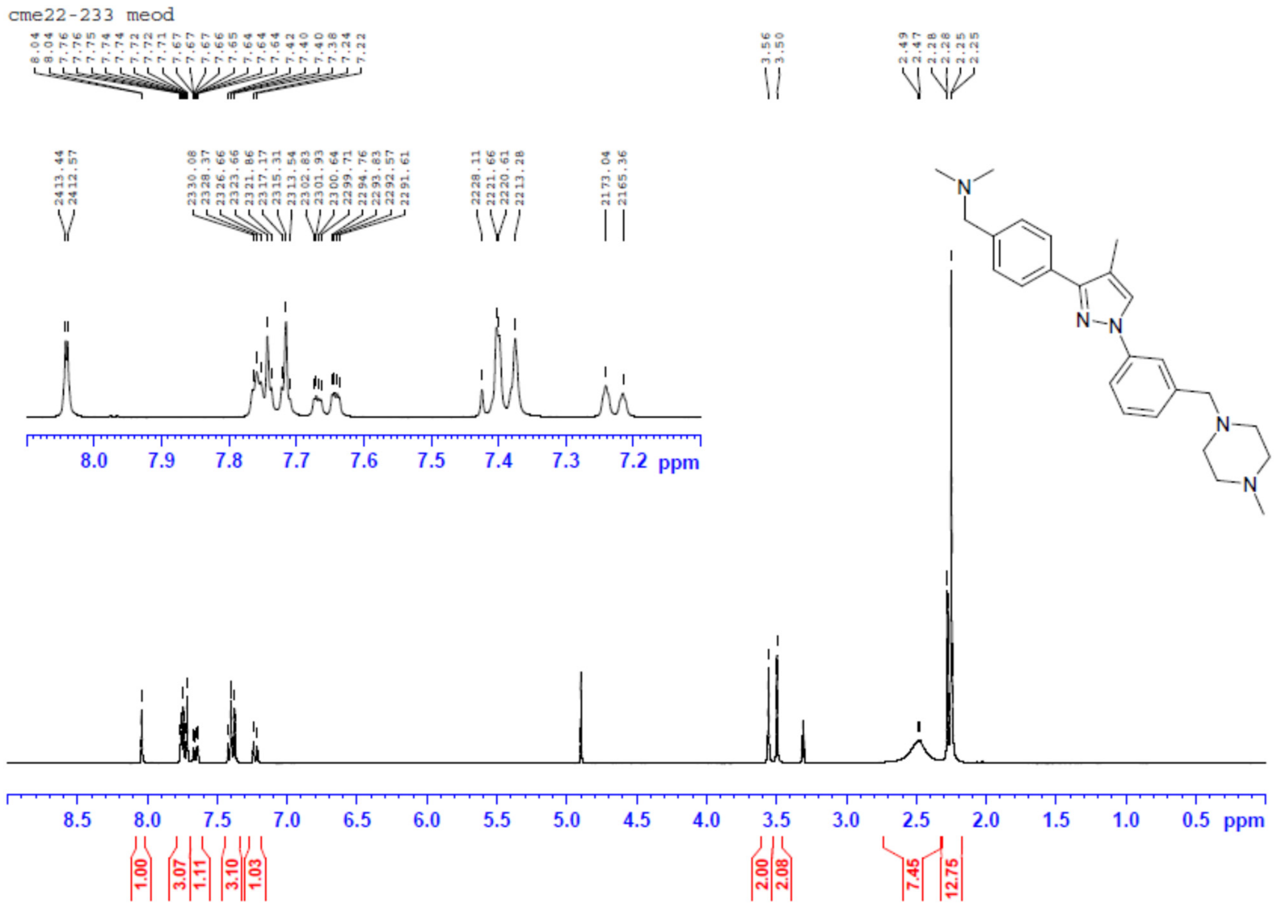

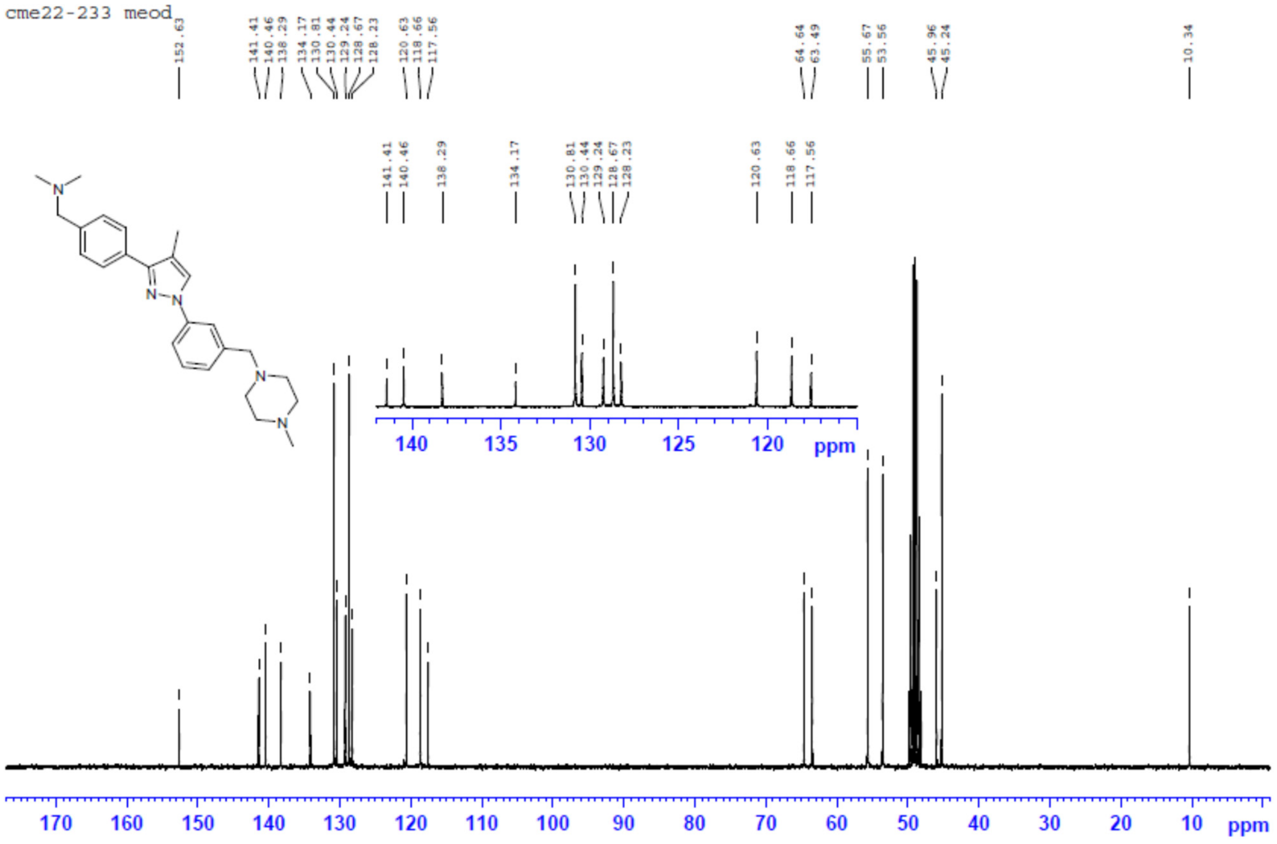

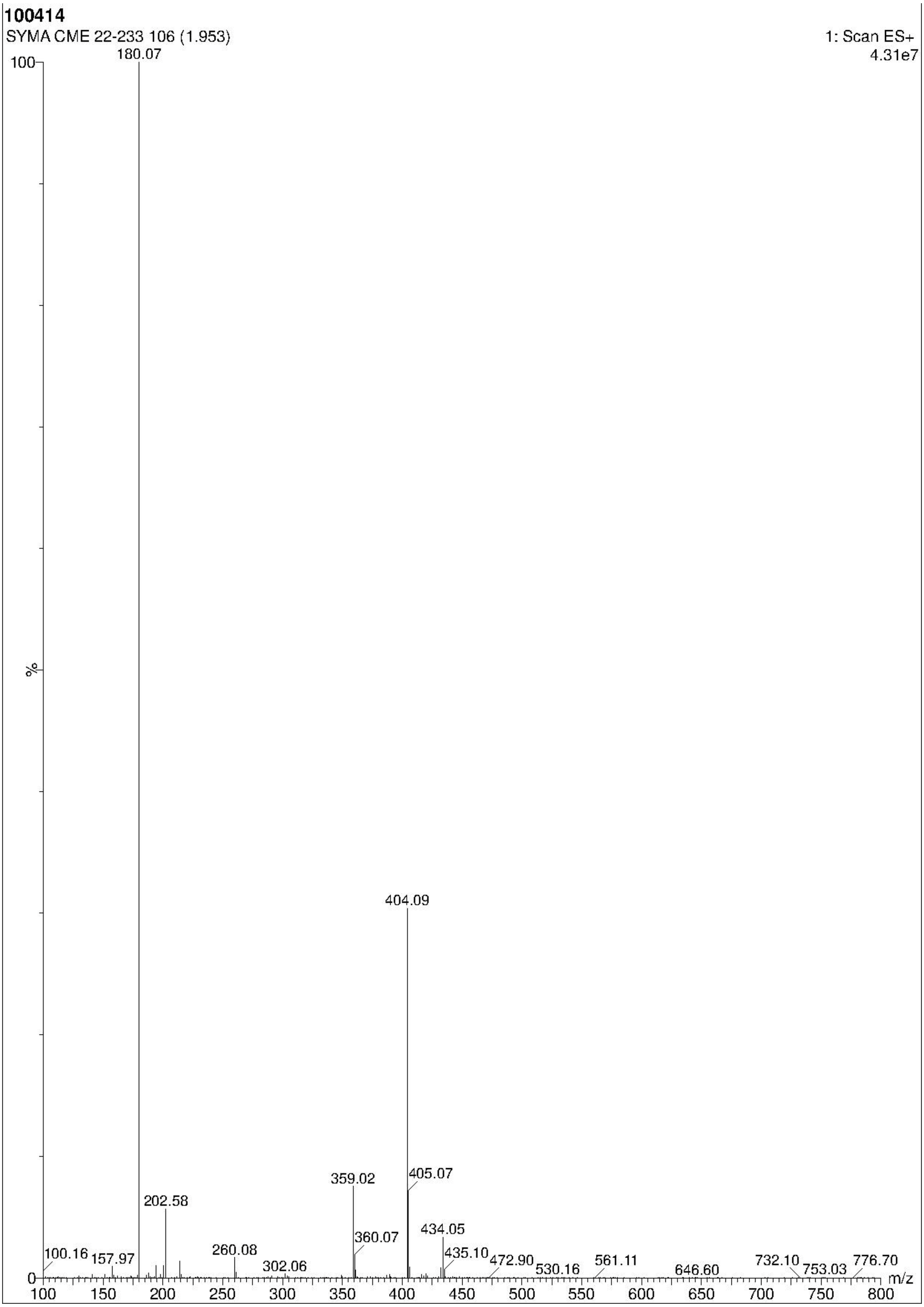
100414
SYMA CME 22-233 106 (1.953)
1: Scan ES+
4.31e7
180.07
404.09
359.02
405.07
202.58
434.05
360.07
260.08
435.10
100.16
157.97
302.06
472.90
530.16
561.11
646.60
732.10
753.03
776.70
%
m/z
100
0
100 150 200 250 300 350 400 450 500 550 600 650 700 750 800

HPLC C4-column

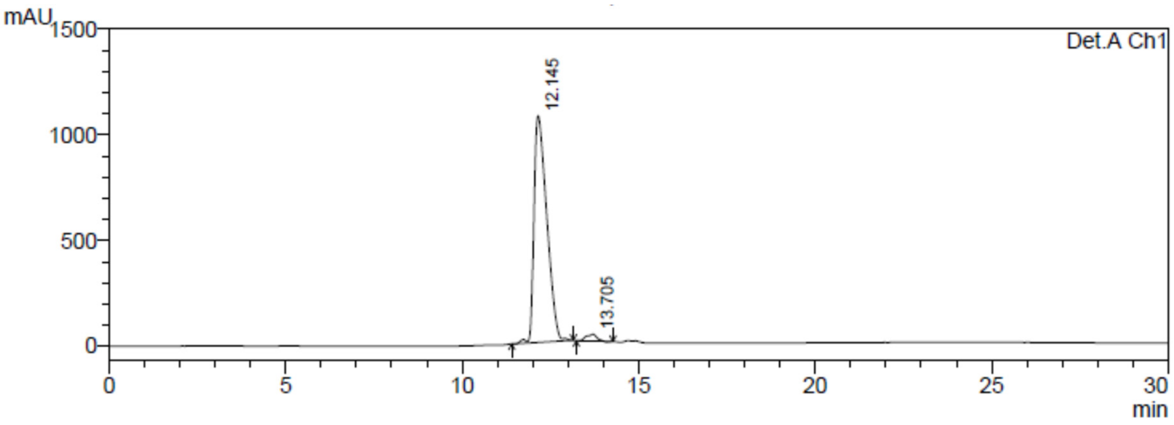

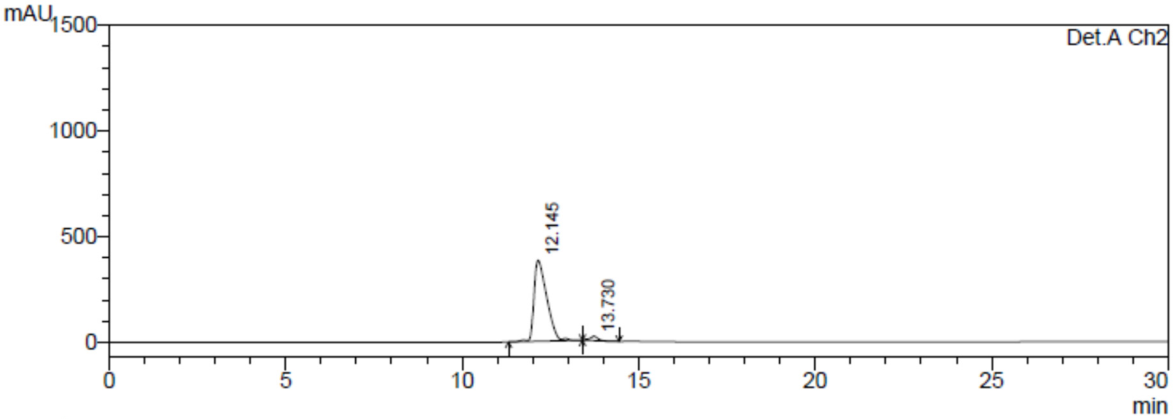

1 Det.A Ch1/215nm
2 Det.A Ch2/254nm

PeakTable

Detector A Ch1 215nm

Peak#	Ret. Time	Area	Area %
1	12.145	26870425	97.662
2	13.705	643410	2.338
Total		27513835	100.000

PeakTable

Detector A Ch2 254nm

Peak#	Ret. Time	Area	Area %
1	12.145	9594704	96.701
2	13.730	327346	3.299
Total		9922050	100.000

HPLC C18-column

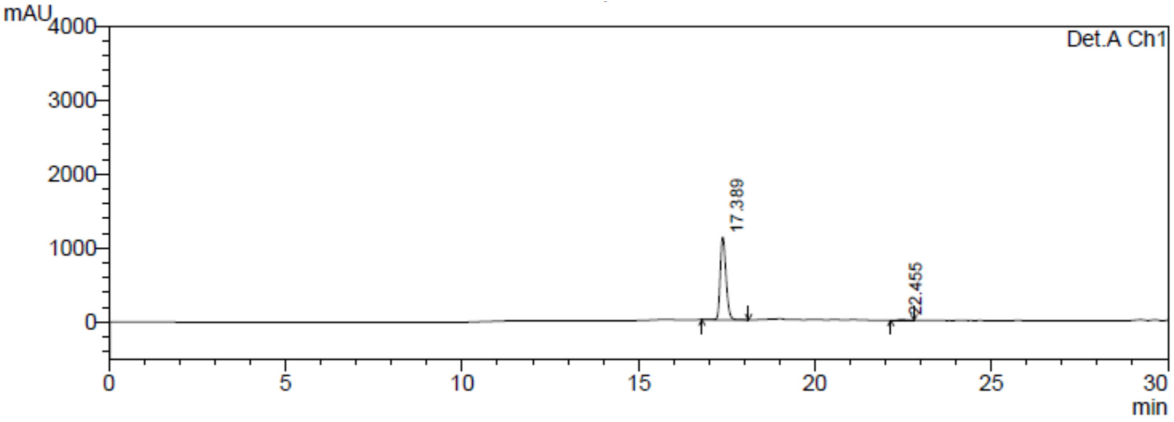

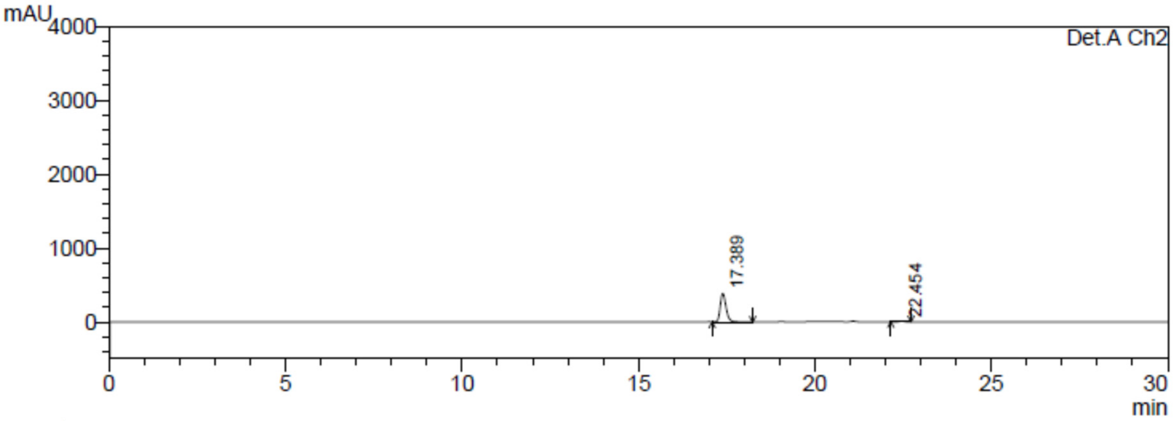

1 Det.A Ch1/215nm
2 Det.A Ch2/254nm

PeakTable

Detector A Ch1 215nm

Peak#	Ret. Time	Area	Area %
1	17.389	12880269	98.993
2	22.455	131013	1.007
Total		13011281	100.000

PeakTable

Detector A Ch2 254nm

Peak#	Ret. Time	Area	Area %
1	17.389	4513313	97.815
2	22.454	100805	2.185
Total		4614118	100.000

[(4-{1-[3-({[3-(dimethylamino)propyl](methyl)amino}methyl)phenyl]-4-methyl-1*H*-pyrazol-3-yl}phenyl)methyl]dimethylamine (**63**)

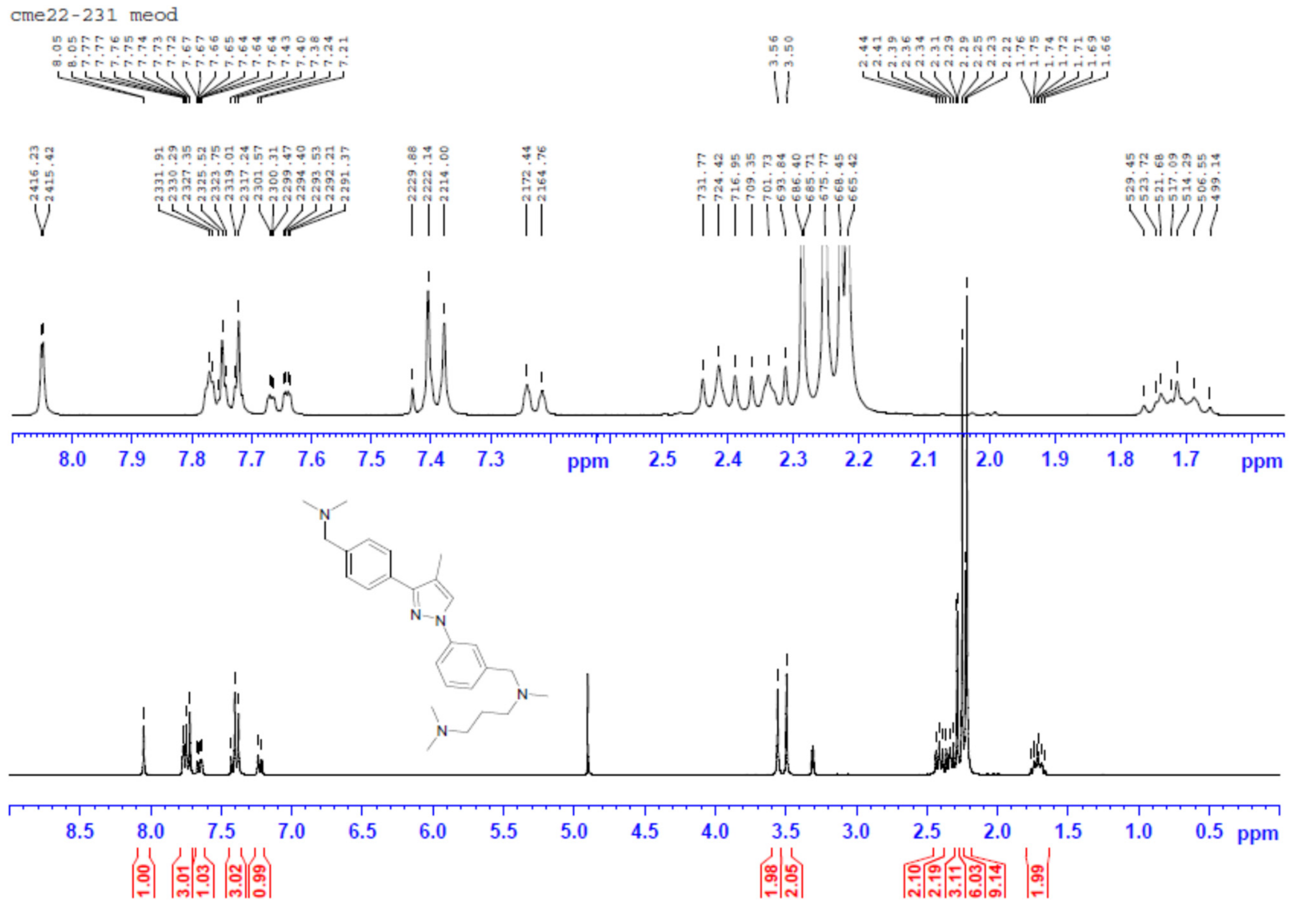

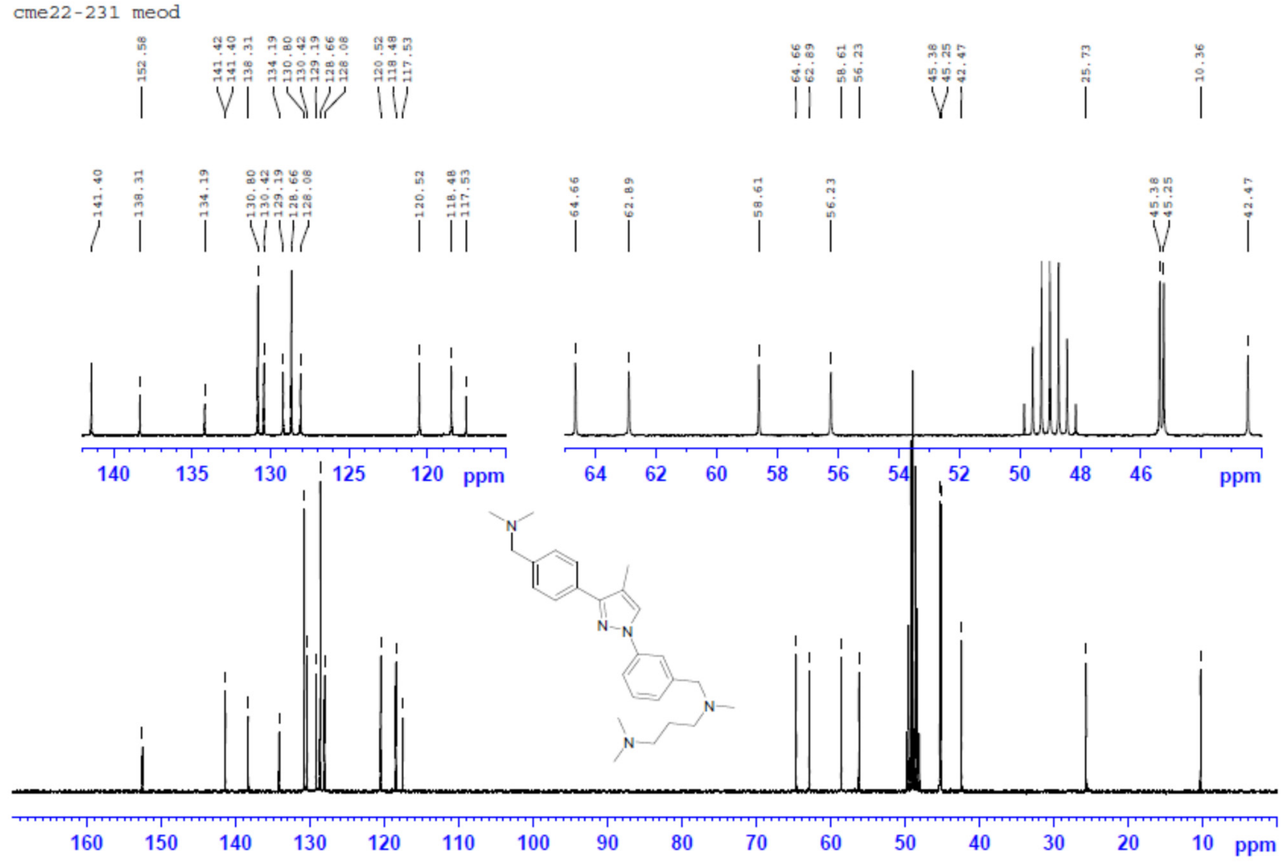

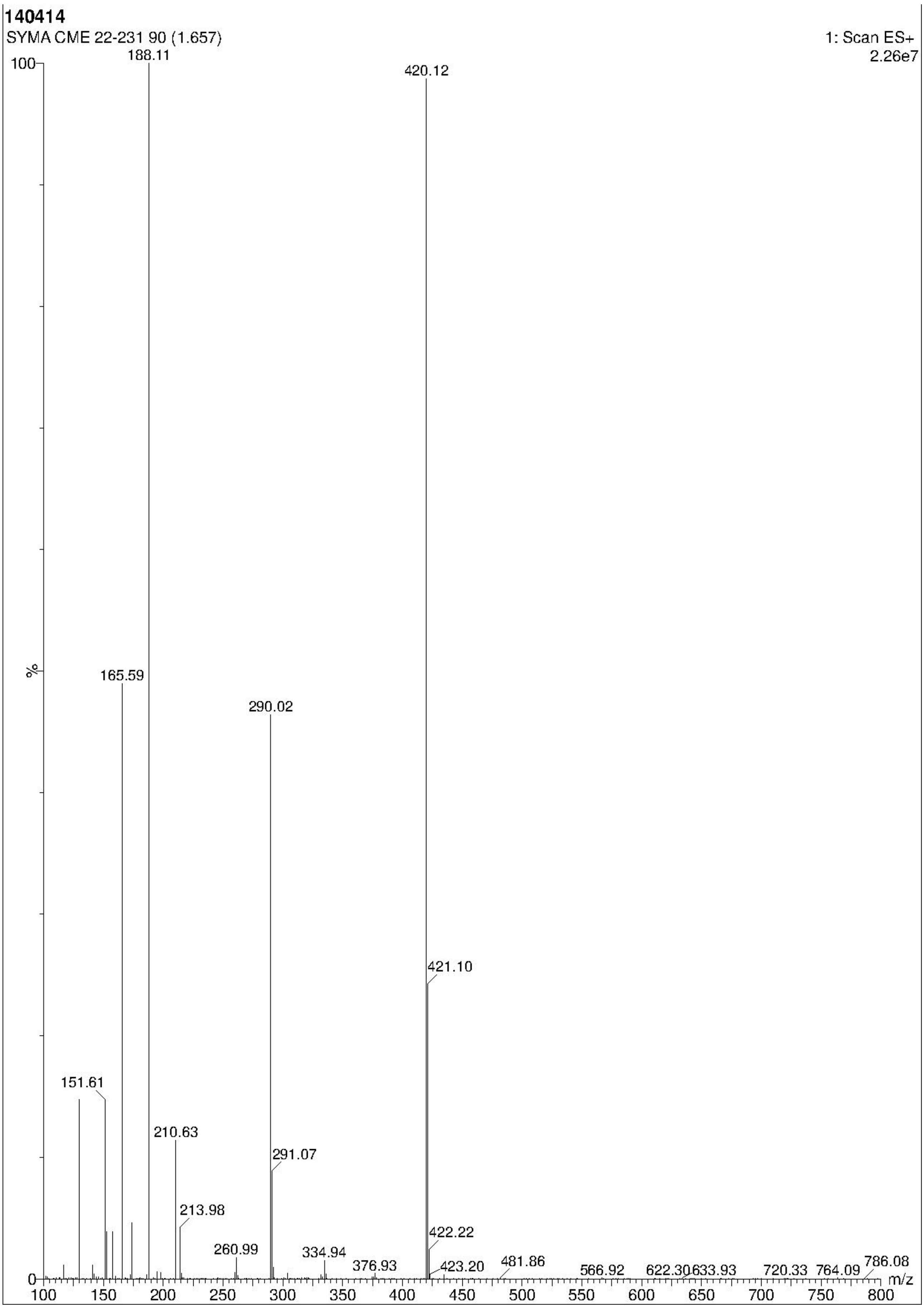

140414
SYMA CME 22-231 90 (1.657)
1: Scan ES+
2.26e7
100
%
0
188.11
420.12
165.59
290.02
421.10
151.61
210.63
291.07
213.98
422.22
260.99
334.94
376.93
423.20
481.86
566.92
622.30
633.93
720.33
764.09
786.08
m/z
100
150
200
250
300
350
400
450
500
550
600
650
700
750
800

HPLC C4-column

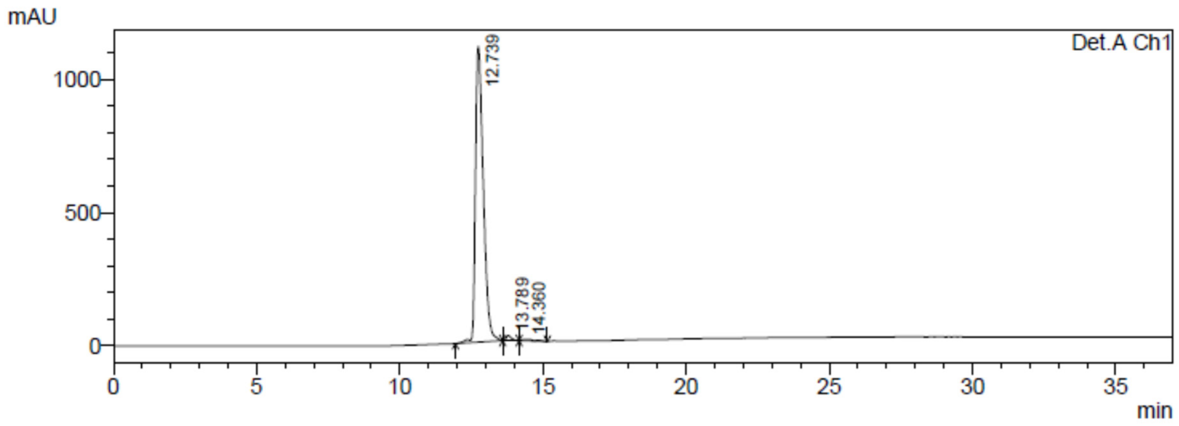

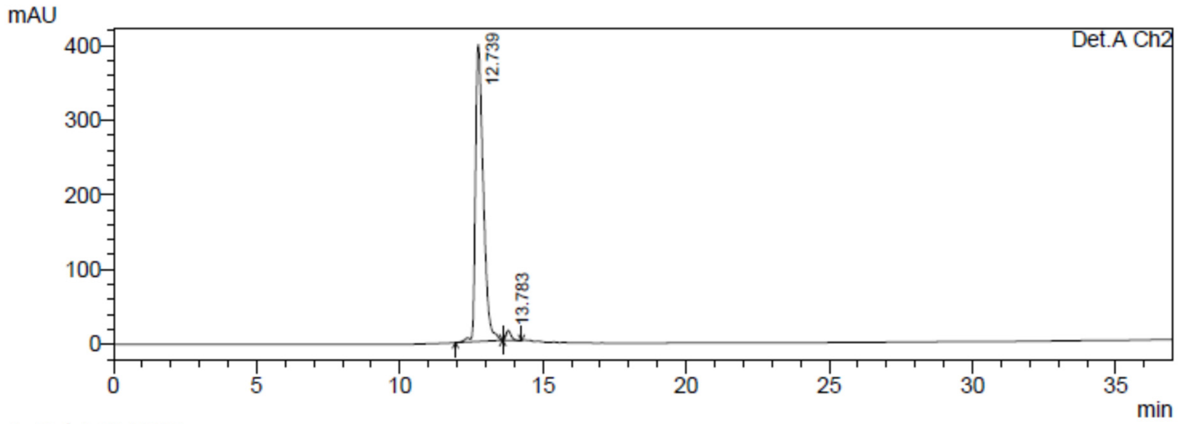

1 Det.A Ch1/215nm
2 Det.A Ch2/254nm

PeakTable
Detector A Ch1 215nm

Peak#	Ret. Time	Area	Area %
1	12.739	21223940	98.226
2	13.789	231835	1.073
3	14.360	151568	0.701
Total		21607343	100.000

PeakTable
Detector A Ch2 254nm

Peak#	Ret. Time	Area	Area %
1	12.739	7571763	98.054
2	13.783	150283	1.946
Total		7722046	100.000

HPLC C18-column

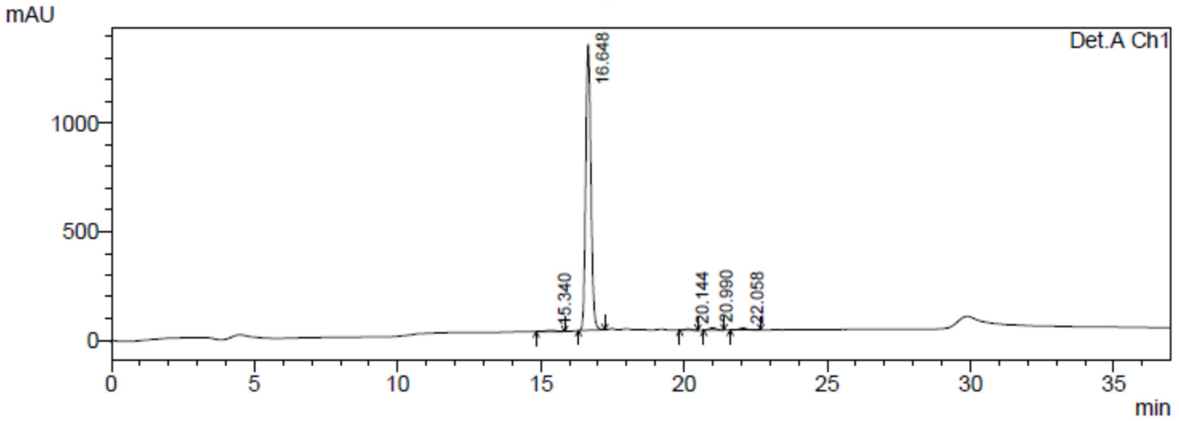

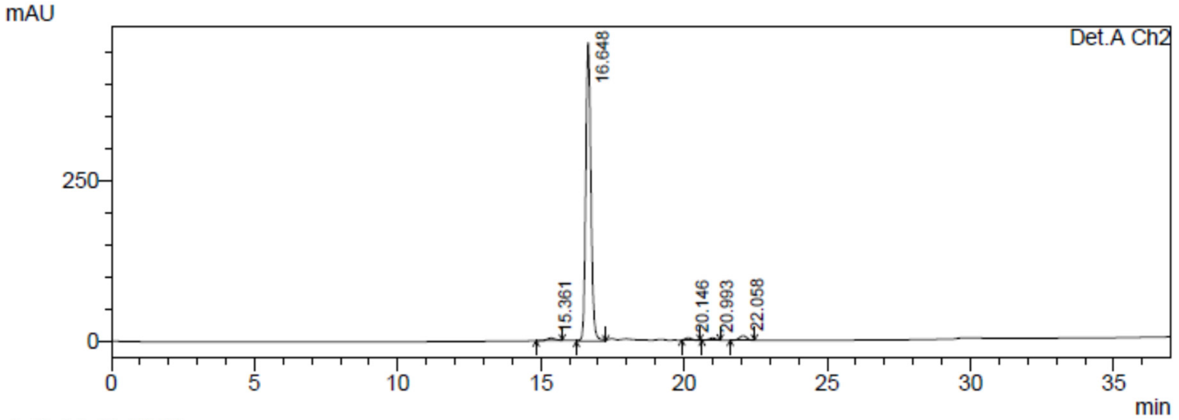

1 Det.A Ch1/215nm
2 Det.A Ch2/254nm

PeakTable

Detector A Ch1 215nm

Peak#	Ret. Time	Area	Area %
1	15.340	138220	0.806
2	16.648	16607662	96.849
3	20.144	85380	0.498
4	20.990	162404	0.947
5	22.058	154269	0.900
Total		17147936	100.000

PeakTable

Detector A Ch2 254nm

Peak#	Ret. Time	Area	Area %
1	15.361	75093	1.225
2	16.648	5835045	95.150
3	20.146	50840	0.829
4	20.993	50638	0.826
5	22.058	120876	1.971
Total		6132491	100.000

1-[3-[1-[3-(dimethylaminomethyl)phenyl]-4-methyl-pyrazol-3-yl]phenyl]-*N*,*N*-dimethyl-methanamine (**64**)

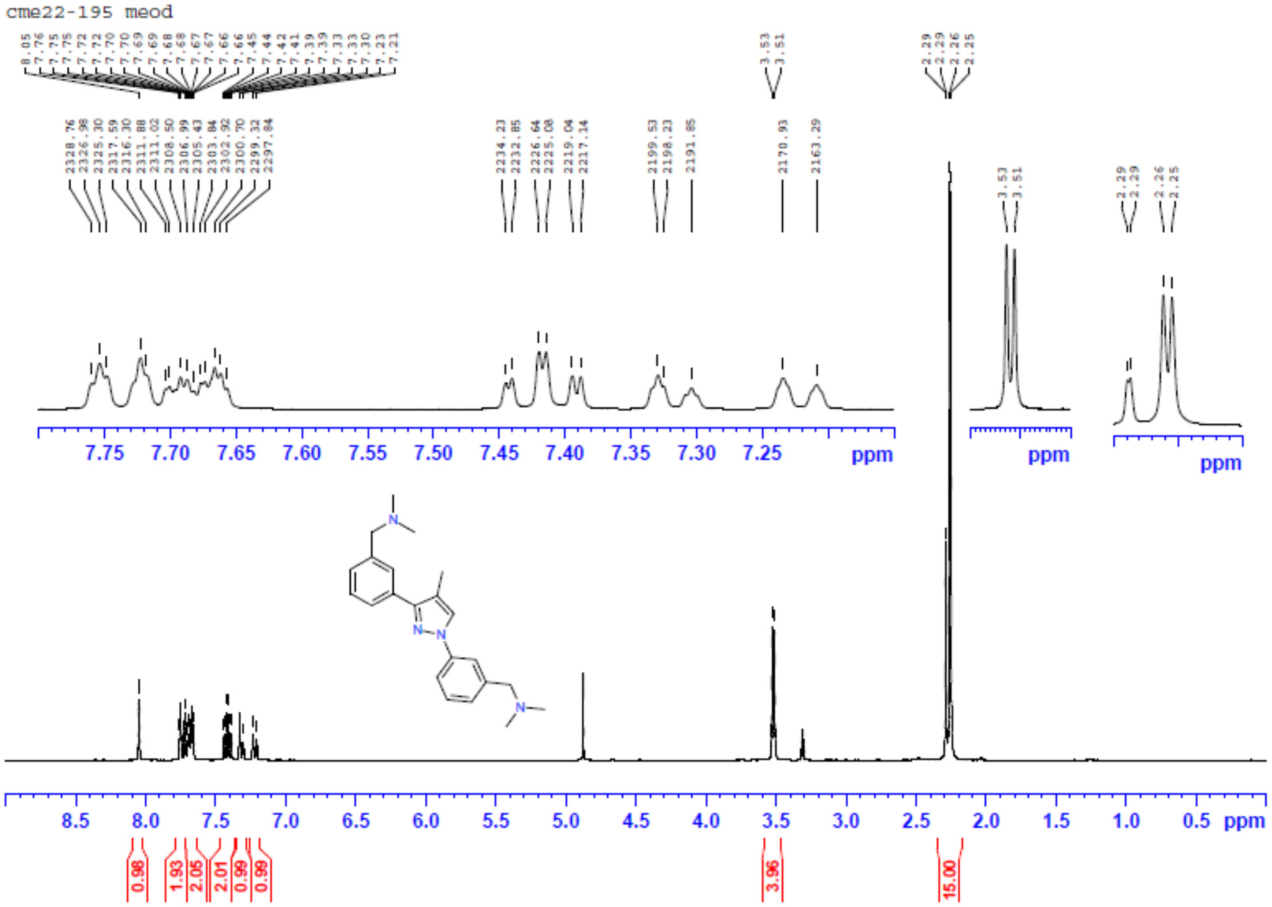

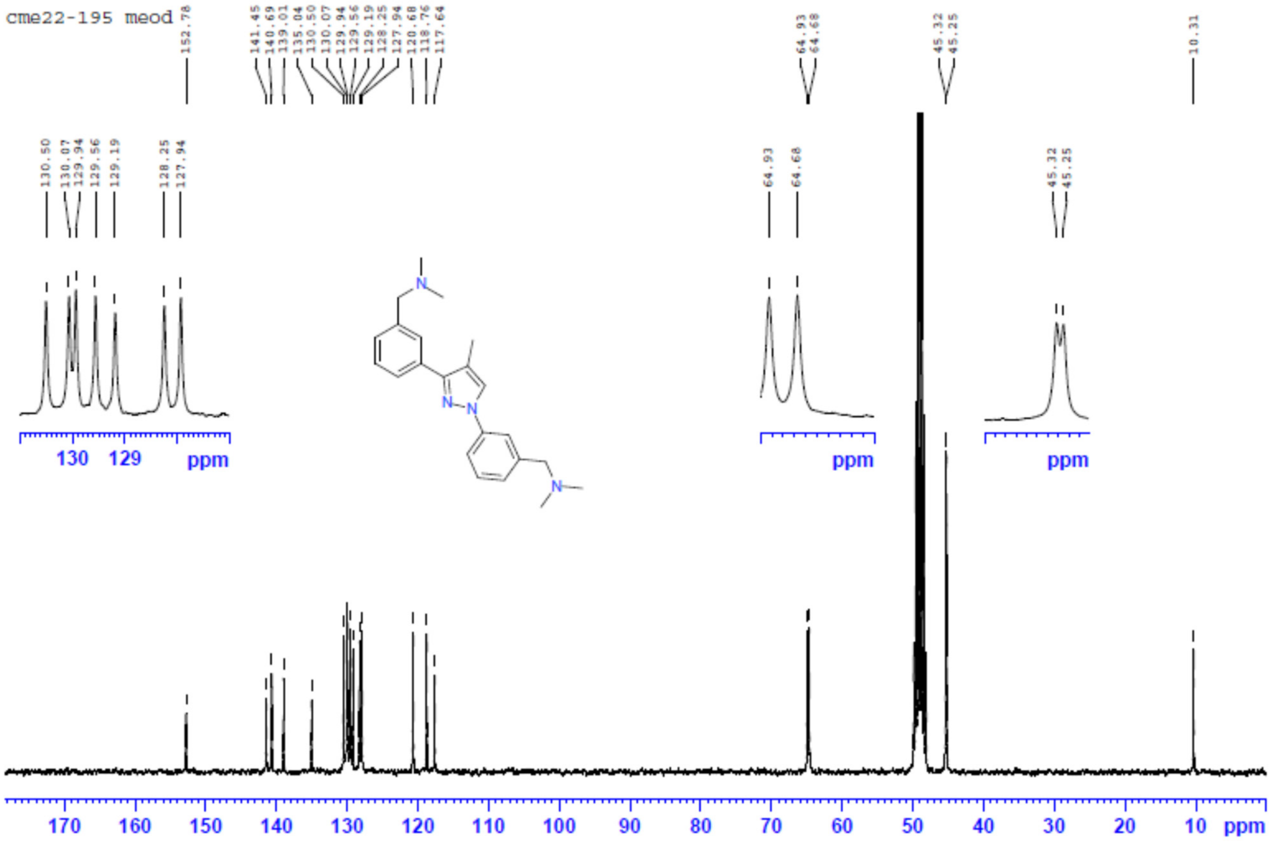

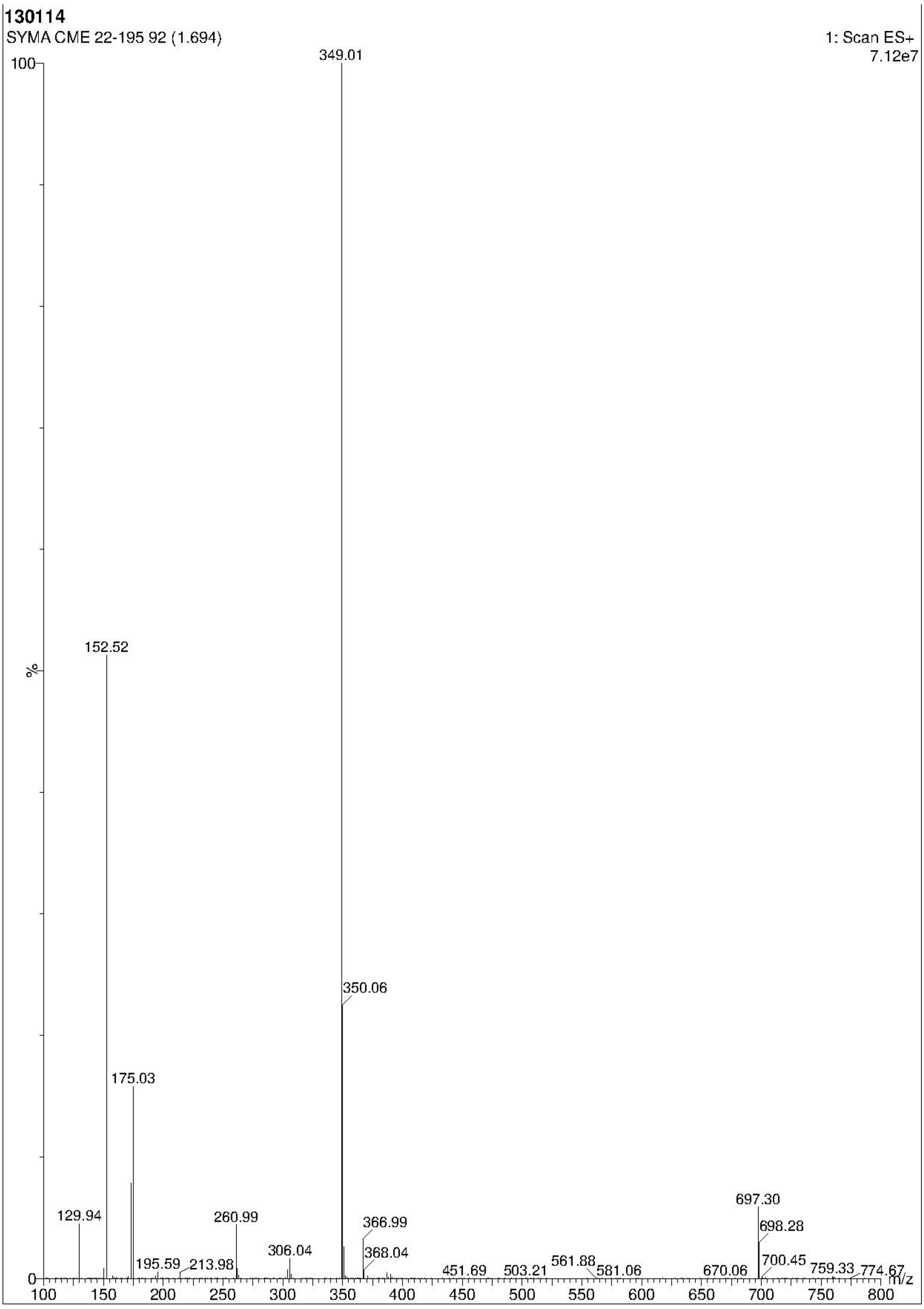
130114
SYMA CME 22-195 92 (1.694)
1: Scan ES+
7.12e7
349.01
152.52
350.06
175.03
129.94
260.99
366.99
306.04
368.04
195.59
213.98
451.69
503.21
561.88
581.06
670.06
697.30
698.28
700.45
759.33
774.67
%
100
0
100
150
200
250
300
350
400
450
500
550
600
650
700
750
800
m/z

HPLC C4-column

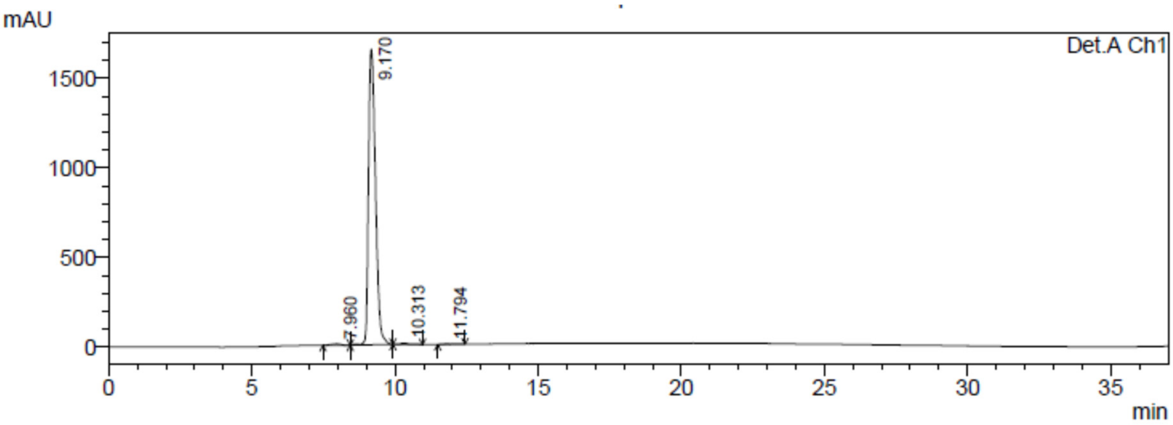

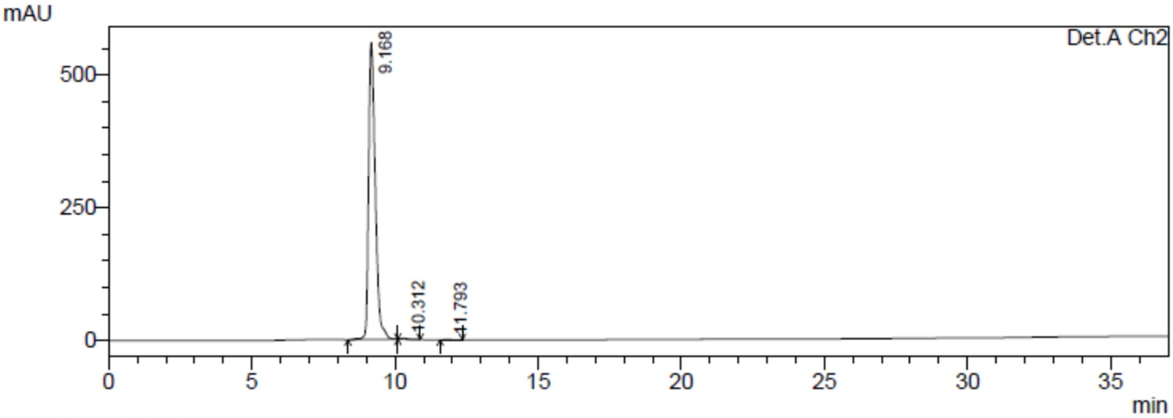

1 Det.A Ch1/215nm
2 Det.A Ch2/254nm

PeakTable

Detector A Ch1 215nm

Peak#	Ret. Time	Area	Area %
1	7.960	199050	0.701
2	9.170	27984885	98.614
3	10.313	120539	0.425
4	11.794	73771	0.260
Total		28378246	100.000

PeakTable

Detector A Ch2 254nm

Peak#	Ret. Time	Area	Area %
1	9.168	9042488	99.560
2	10.312	24727	0.272
3	11.793	15236	0.168
Total		9082451	100.000

HPLC C18-column

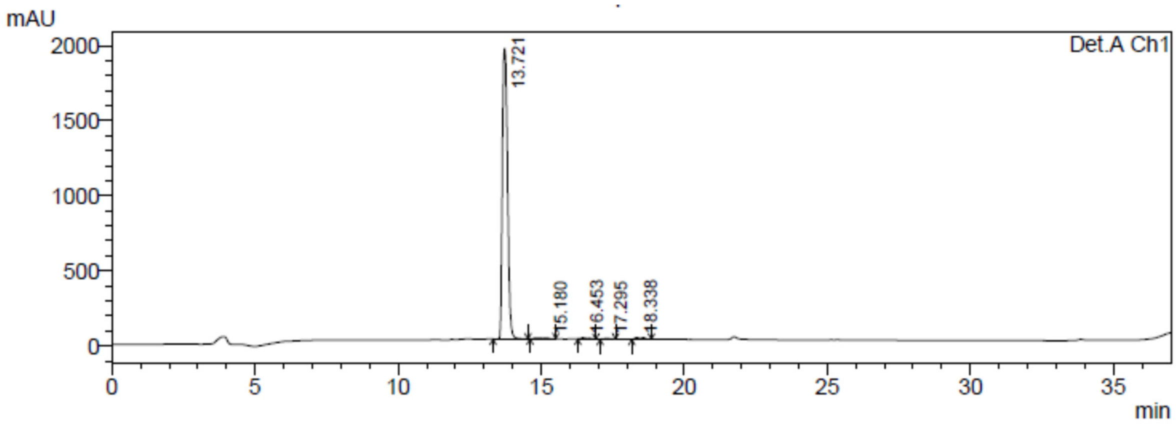

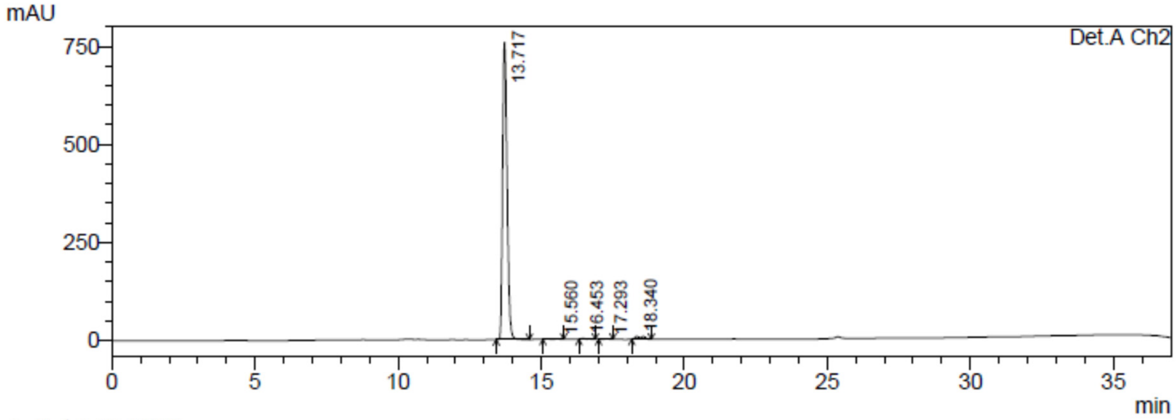

1 Det.A Ch1/215nm
2 Det.A Ch2/254nm

PeakTable

Detector A Ch1 215nm

Peak#	Ret. Time	Area	Area %
1	13.721	24326909	97.322
2	15.180	168693	0.675
3	16.453	165940	0.664
4	17.295	78500	0.314
5	18.338	256191	1.025
Total		24996232	100.000

PeakTable

Detector A Ch2 254nm

Peak#	Ret. Time	Area	Area %
1	13.717	8281640	97.005
2	15.560	43593	0.511
3	16.453	35339	0.414
4	17.293	21819	0.256
5	18.340	154932	1.815
Total		8537323	100.000

N,*N*-dimethyl-1-[3-[4-methyl-1-[3-[(4-methylpiperazin-1-yl)methyl]phenyl]pyrazol-3-yl]phenyl]methanamine (**65**)

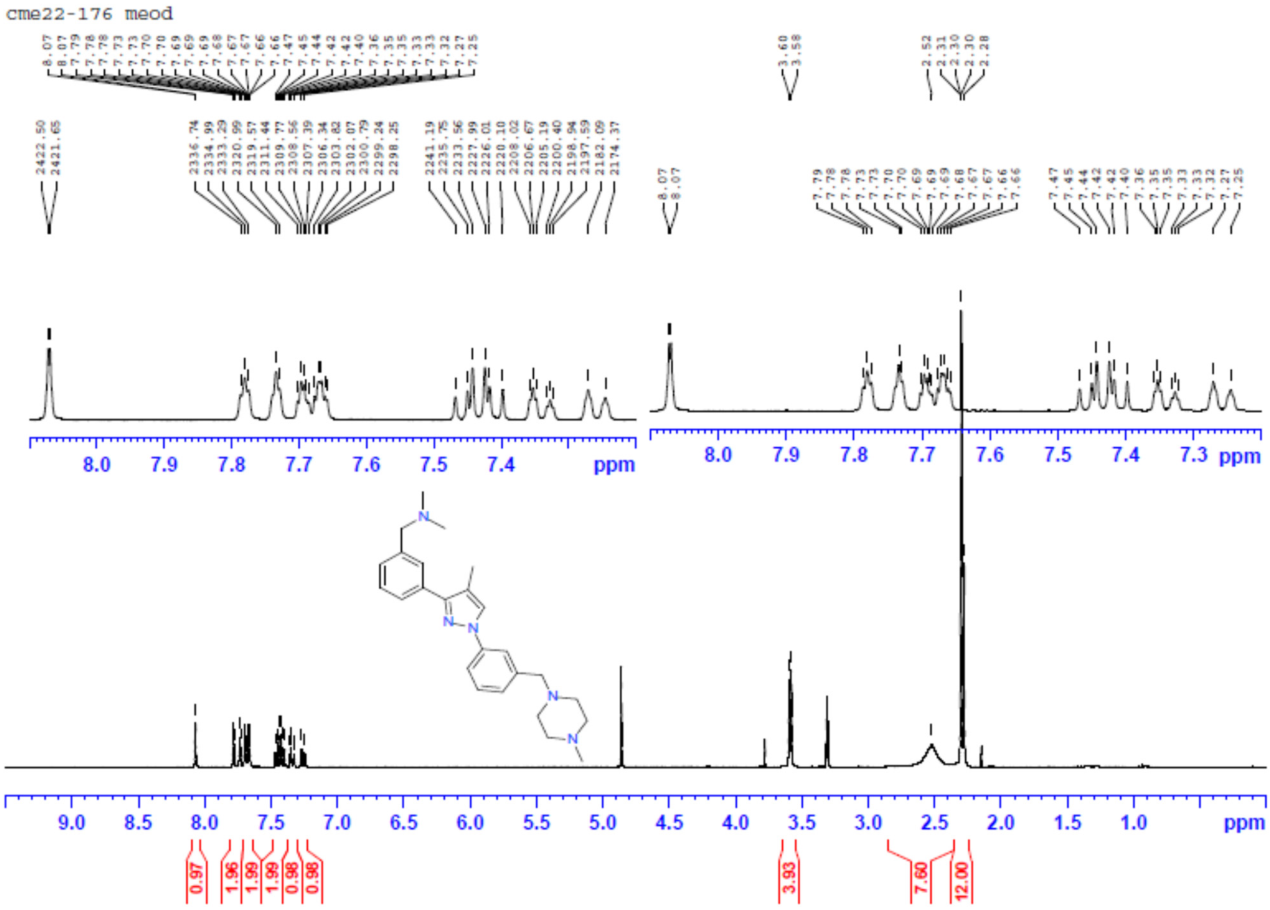

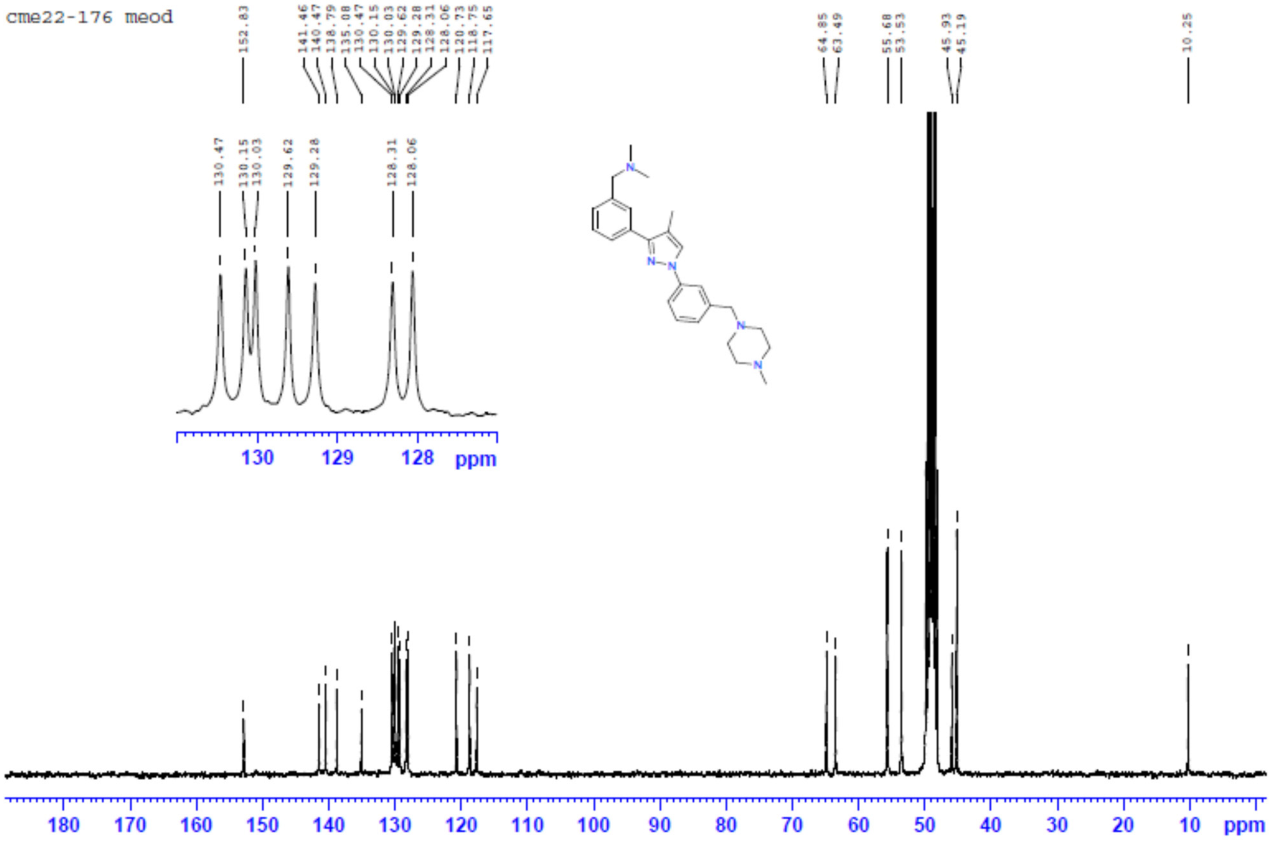

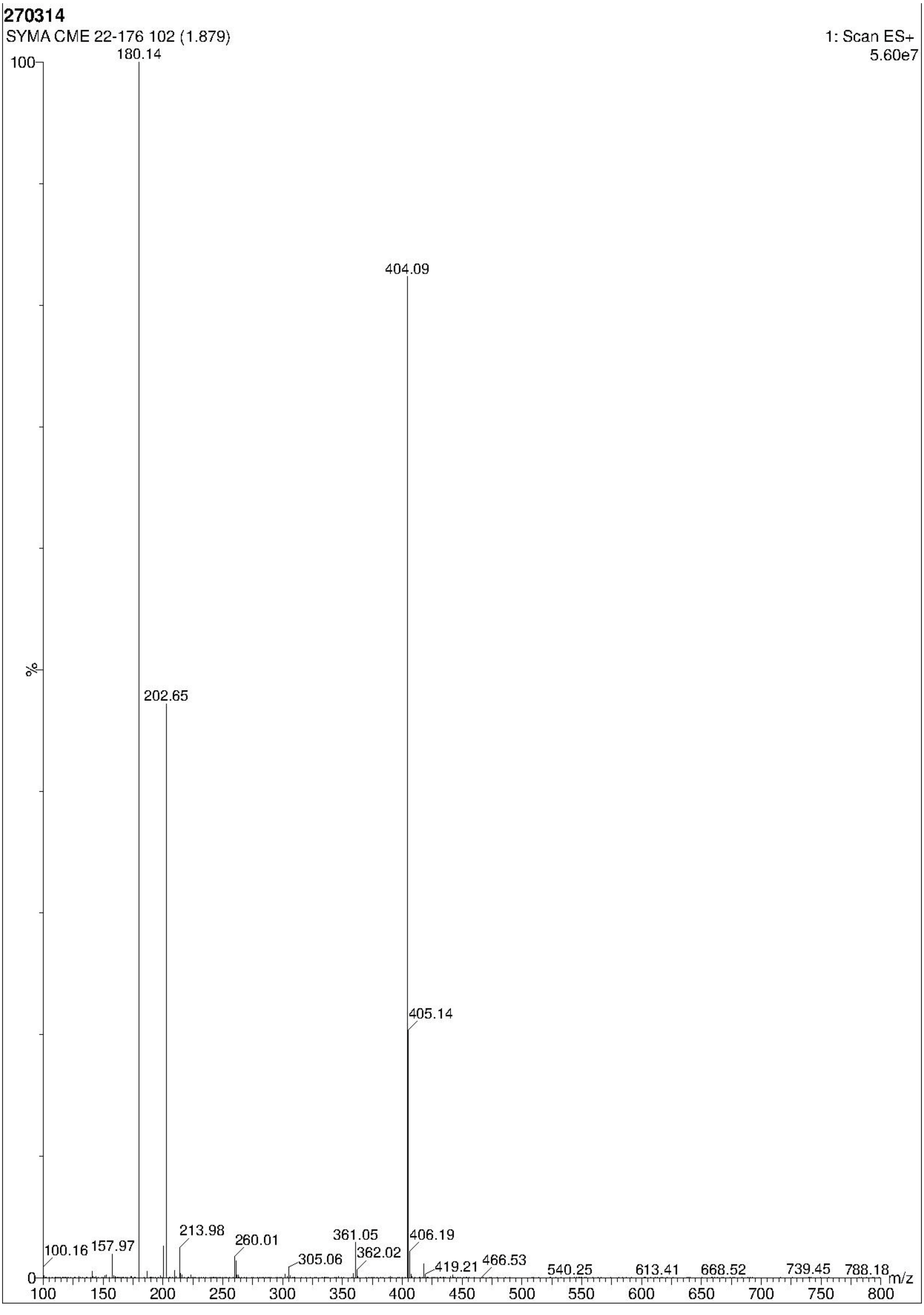

270314
SYMA CME 22-176 102 (1.879)
1: Scan ES+
5.60e7
100
%
0
180.14
404.09
202.65
405.14
100.16
157.97
213.98
260.01
305.06
361.05
362.02
406.19
419.21
466.53
540.25
613.41
668.52
739.45
788.18
m/z
100
150
200
250
300
350
400
450
500
550
600
650
700
750
800

HPLC C4-column

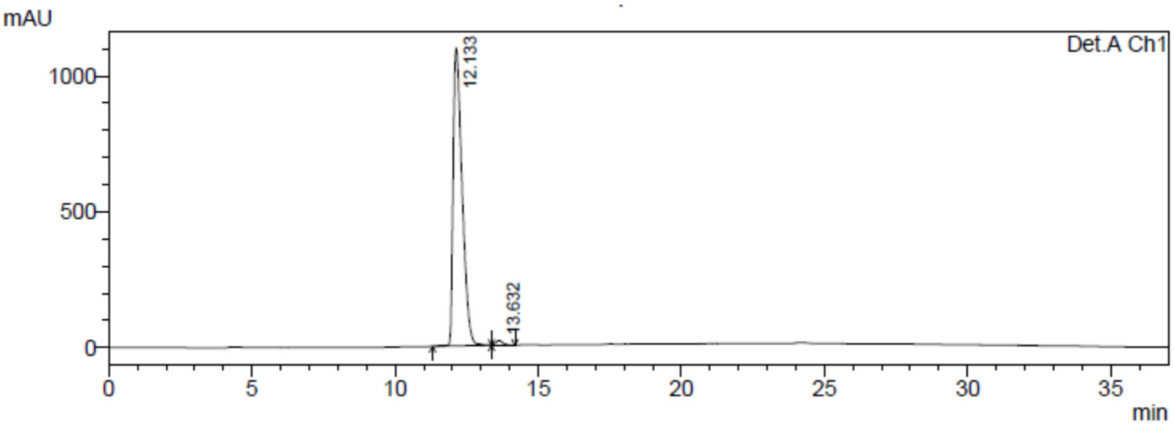

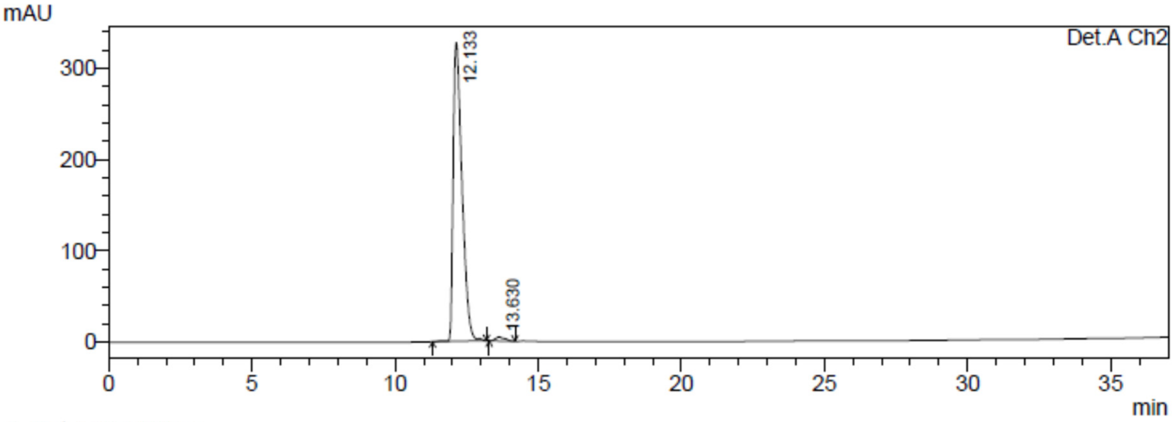

1 Det.A Ch1/215nm
2 Det.A Ch2/254nm

PeakTable

Detector A Ch1 215nm

Peak#	Ret. Time	Area	Area %
1	12.133	23797315	98.791
2	13.632	291180	1.209
Total		24088495	100.000

PeakTable

Detector A Ch2 254nm

Peak#	Ret. Time	Area	Area %
1	12.133	6992005	98.625
2	13.630	97478	1.375
Total		7089483	100.000

HPLC C18-column

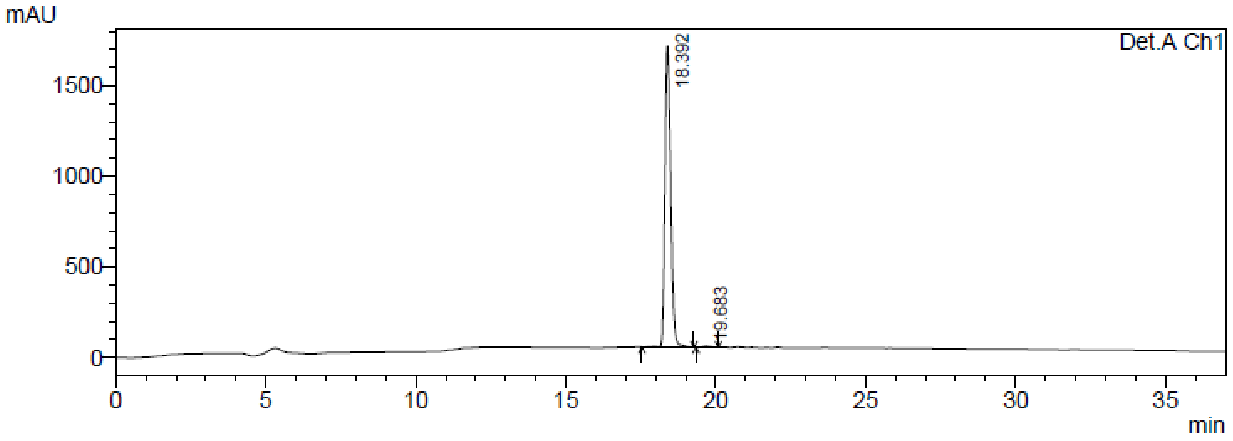

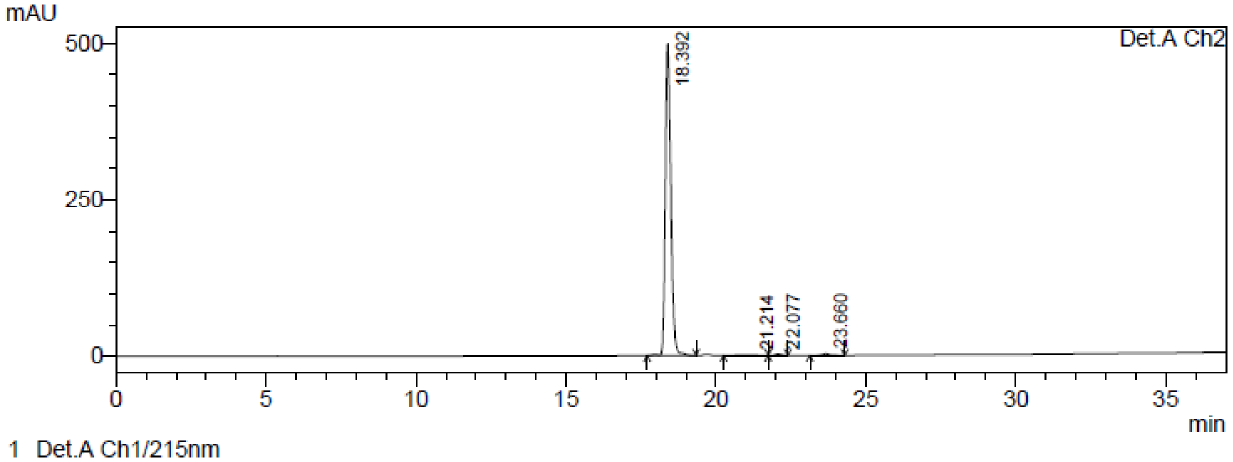

1 Det.A Ch1/215nm
2 Det.A Ch2/254nm

PeakTable

Detector A Ch1 215nm

Peak#	Ret. Time	Area	Area %
1	18.392	22295618	99.573
2	19.683	95629	0.427
Total		22391248	100.000

PeakTable

Detector A Ch2 254nm

Peak#	Ret. Time	Area	Area %
1	18.392	6424273	98.839
2	21.214	18510	0.285
3	22.077	23886	0.367
4	23.660	33098	0.509
Total		6499767	100.000

N'-[[3-[3-[3-(dimethylaminomethyl)phenyl]-4-methyl-pyrazol-1-yl]phenyl]methyl]-*N*,*N*,*N*'-trimethyl-propane-1,3-diamine (**66**)

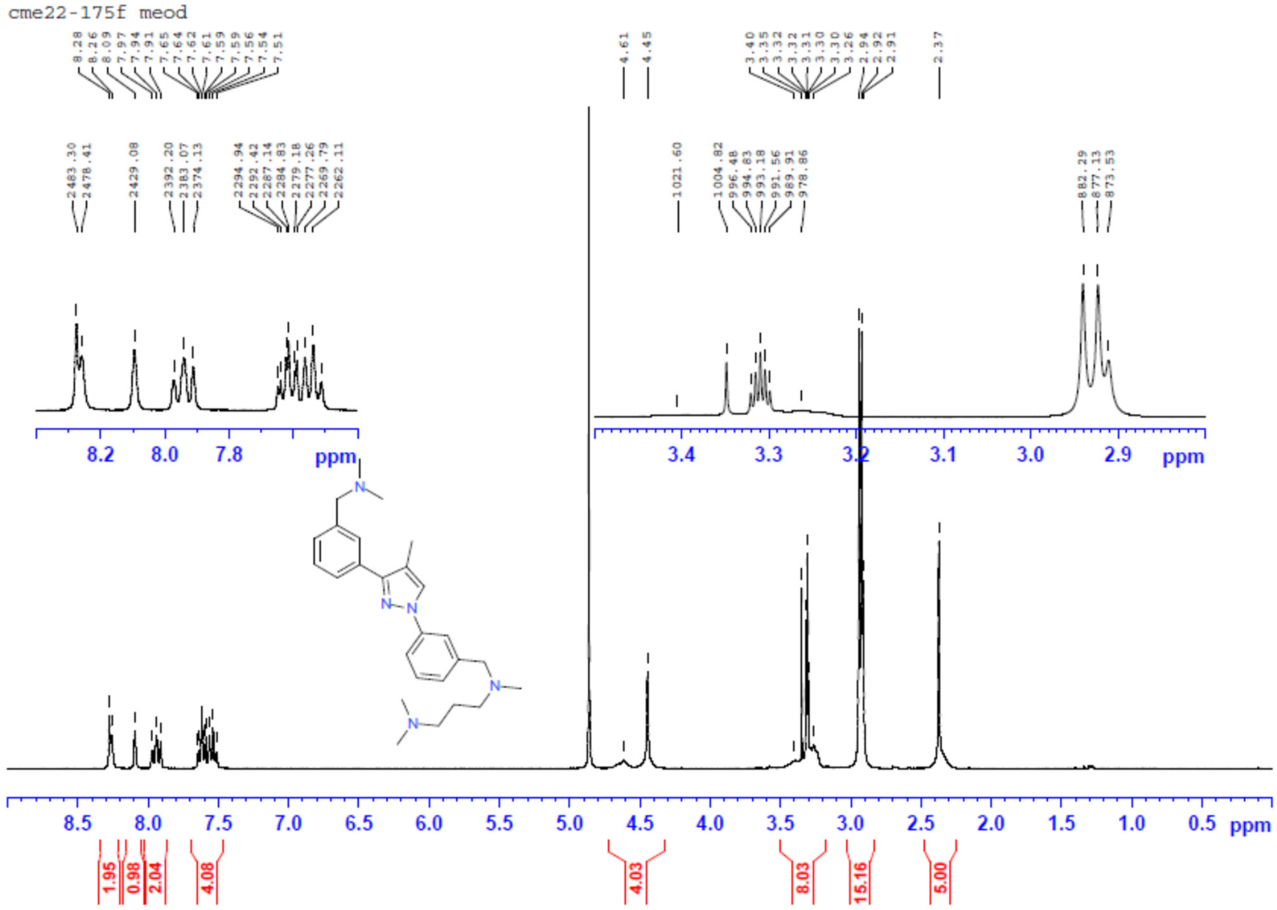

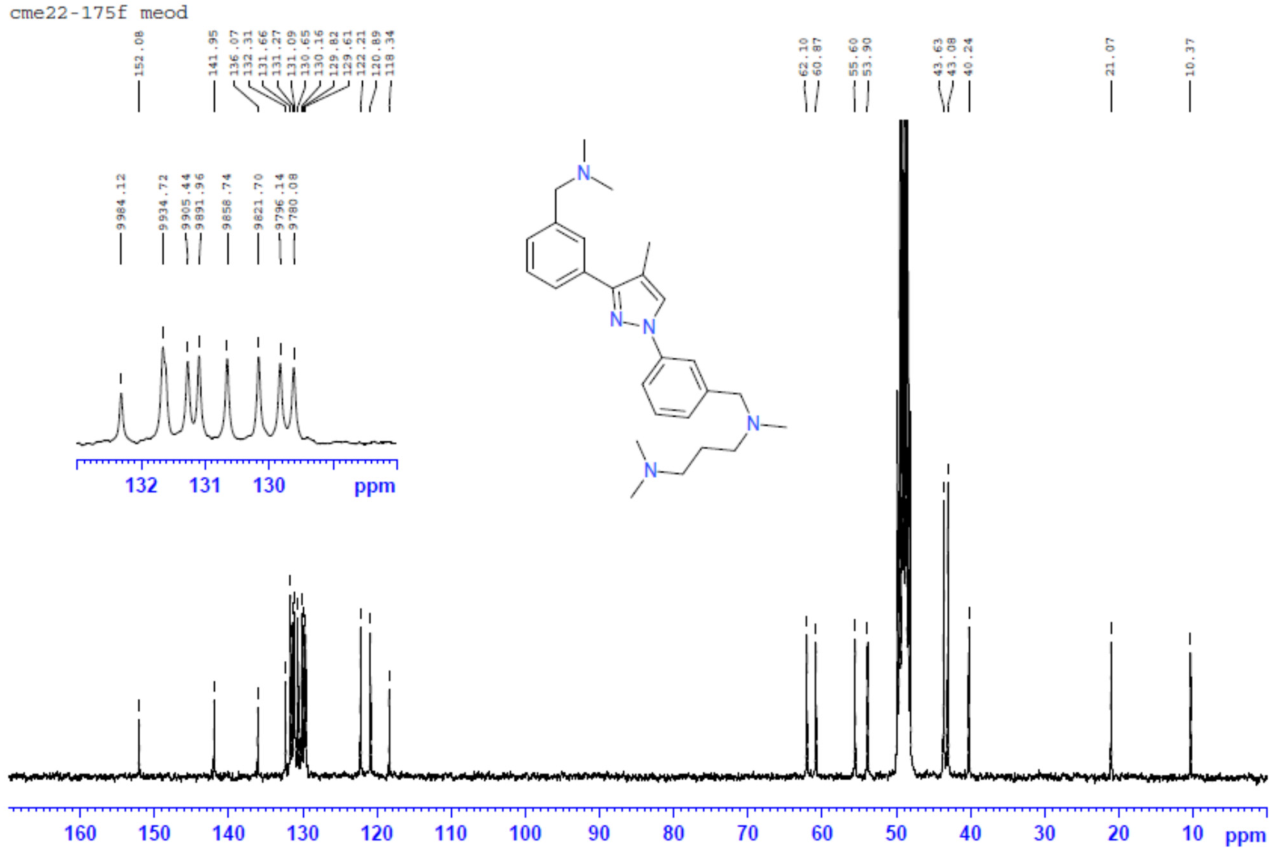

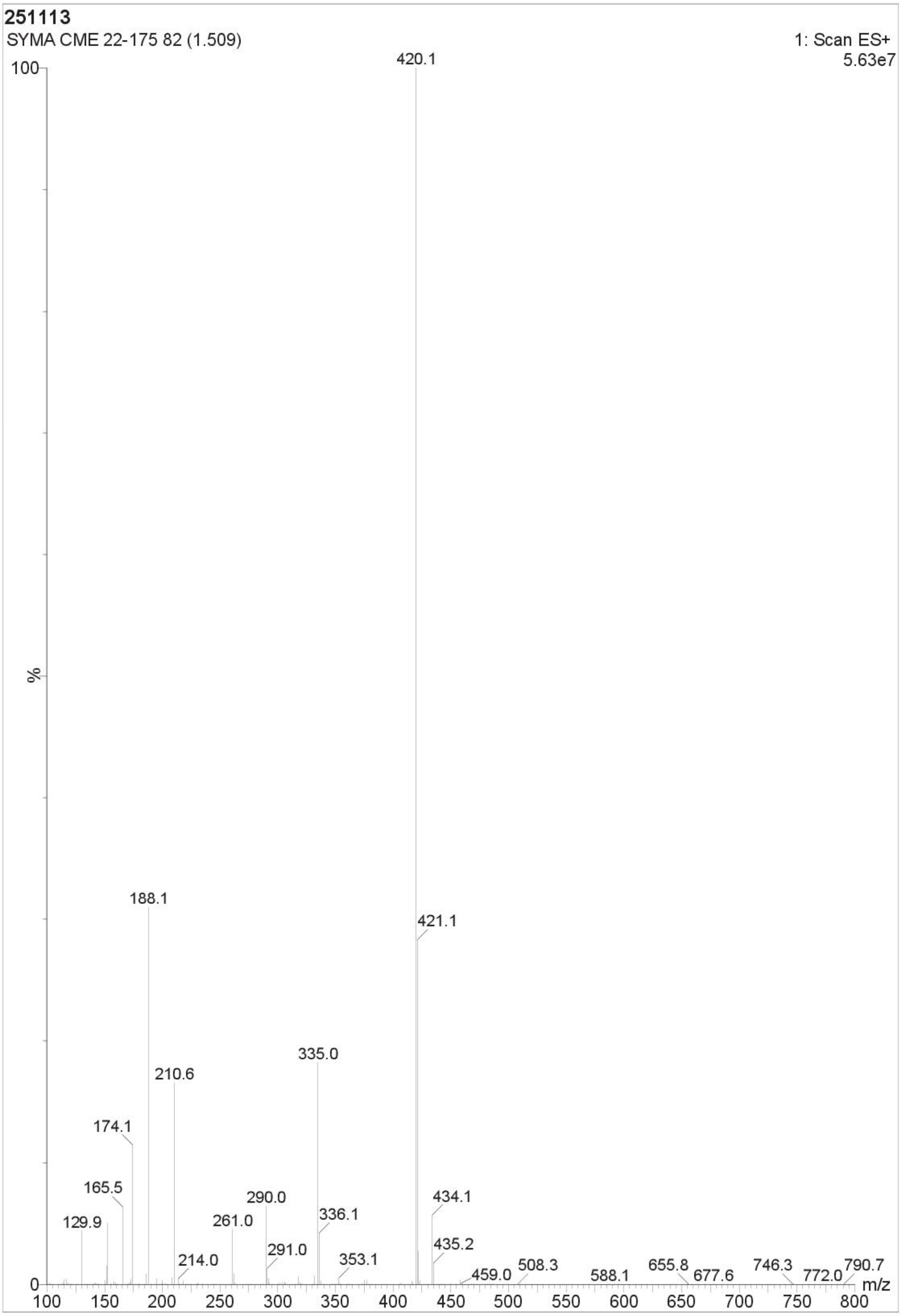
251113
SYMA CME 22-175 82 (1.509)
1: Scan ES+
5.63e7
100
%
0
420.1
188.1
421.1
335.0
210.6
174.1
165.5
290.0
434.1
129.9
261.0
336.1
291.0
435.2
214.0
353.1
459.0
508.3
588.1
655.8
677.6
746.3
772.0
790.7
m/z
100
150
200
250
300
350
400
450
500
550
600
650
700
750
800

HPLC C4-column

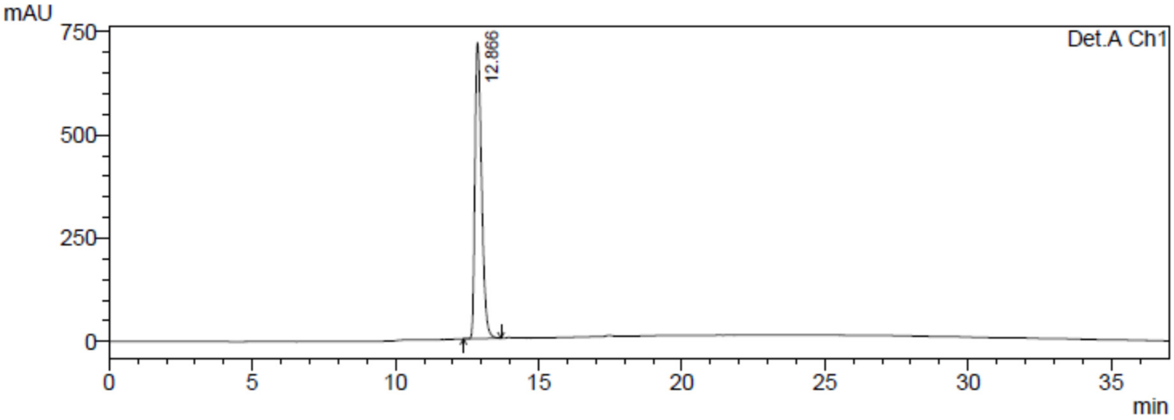

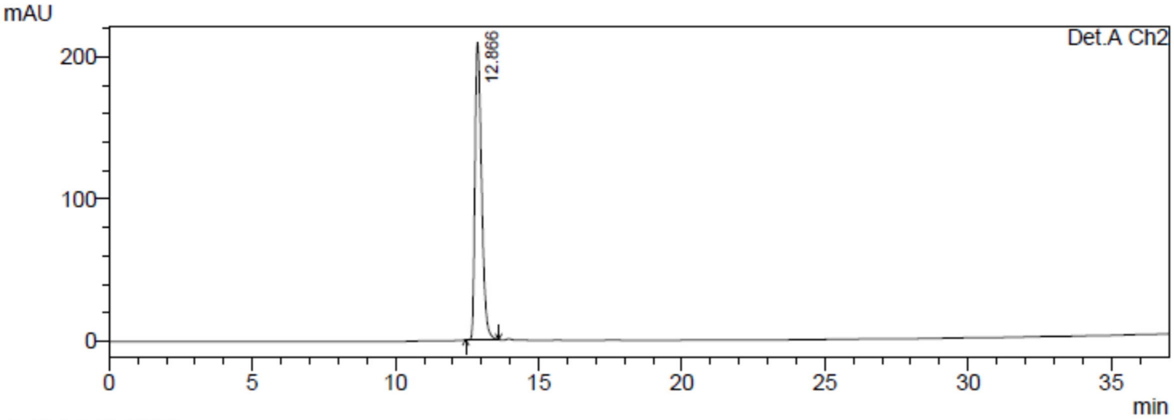

1 Det.A Ch1/215nm
2 Det.A Ch2/254nm

PeakTable

Detector A Ch1 215nm

Peak#	Ret. Time	Area	Area %
1	12.866	11566953	100.000
Total		11566953	100.000

PeakTable

Detector A Ch2 254nm

Peak#	Ret. Time	Area	Area %
1	12.866	3338628	100.000
Total		3338628	100.000

HPLC C18-column

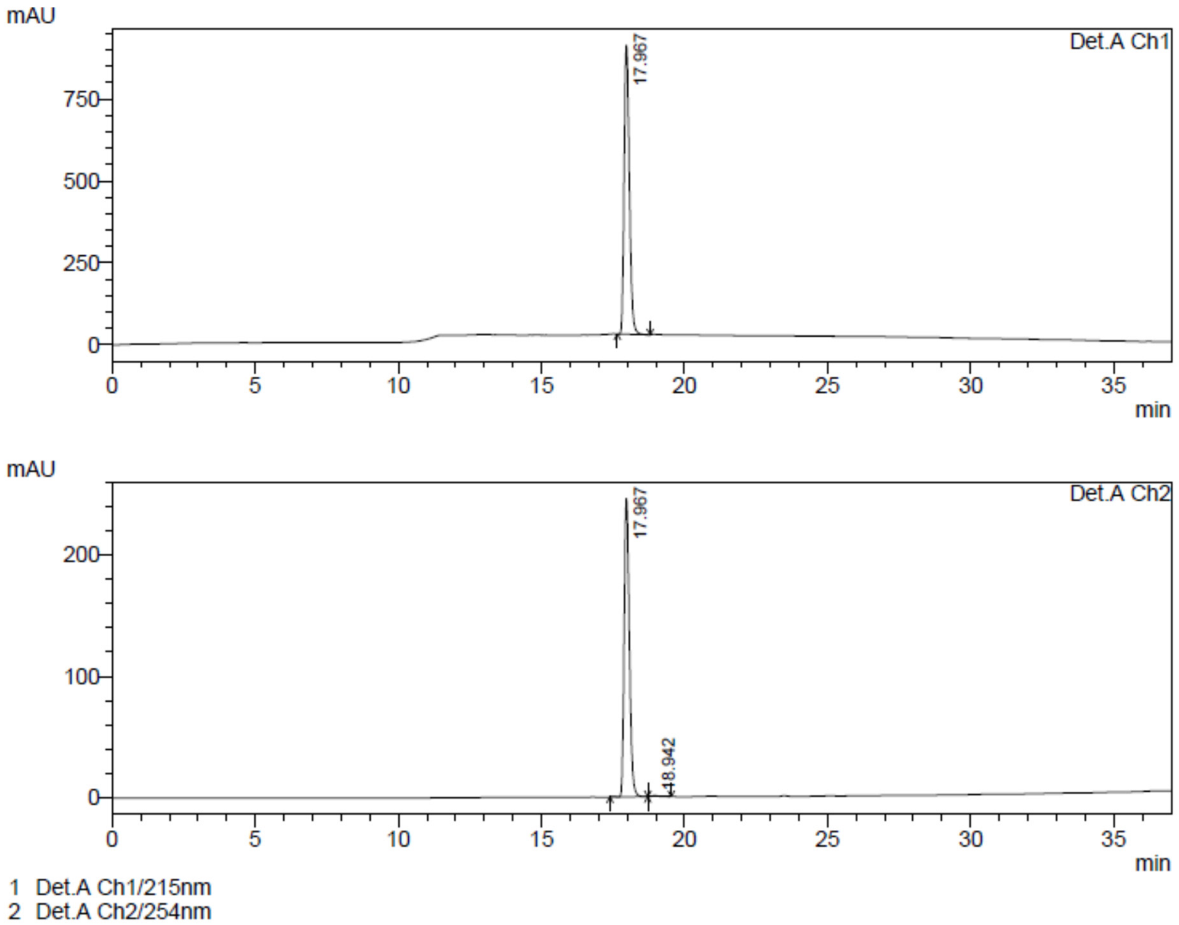

PeakTable

Detector A Ch1 215nm

Peak#	Ret. Time	Area	Area %
1	17.967	11145280	100.000
Total		11145280	100.000

PeakTable

Detector A Ch2 254nm

Peak#	Ret. Time	Area	Area %
1	17.967	3078683	99.498
2	18.942	15534	0.502
Total		3094217	100.000

{[4-(1-{3-[(dimethylamino)methyl]phenyl}-4-methyl-1*H*-pyrazol-3-yl)phenyl]methyl}[3-(dimethylamino)propyl]methylamine (**67**)

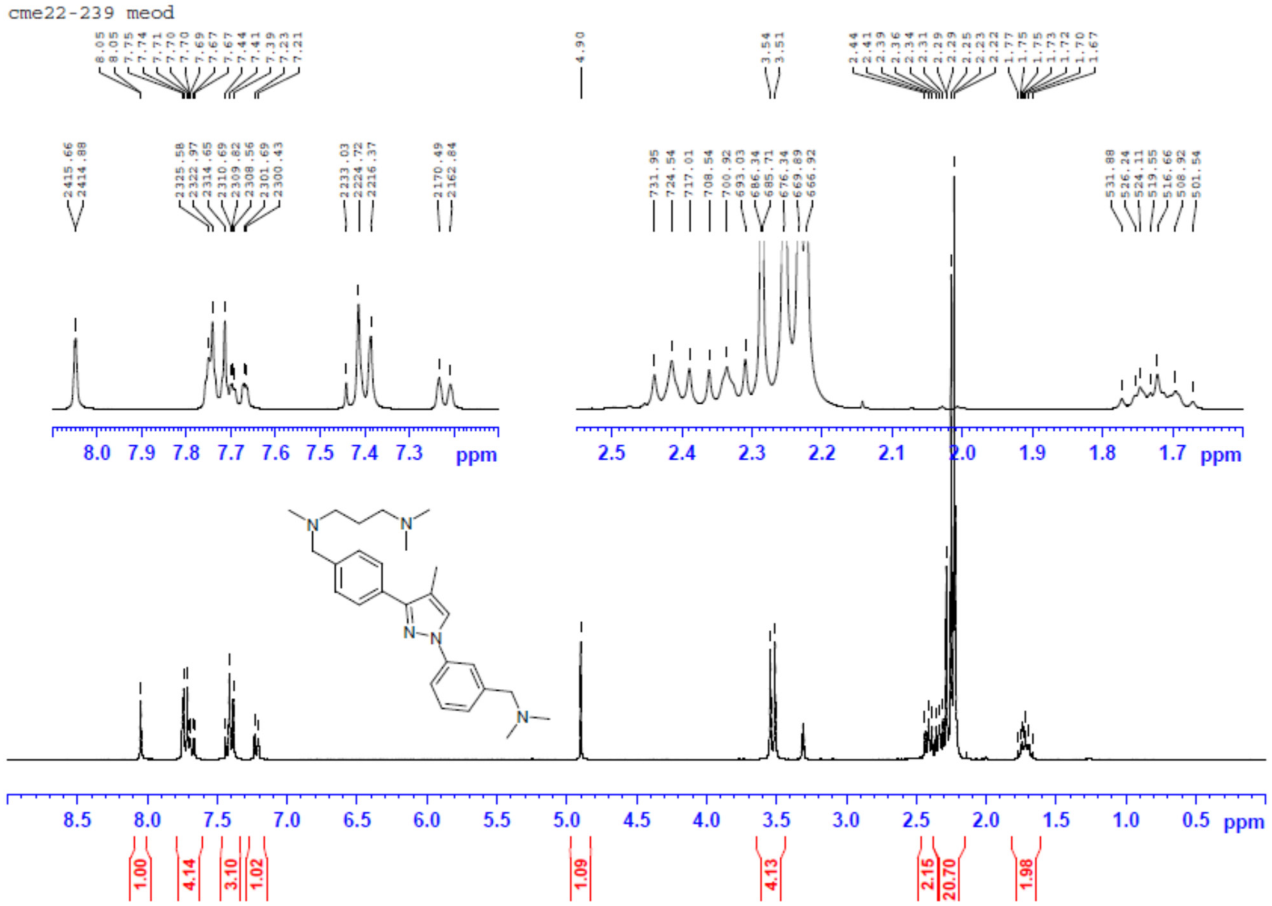

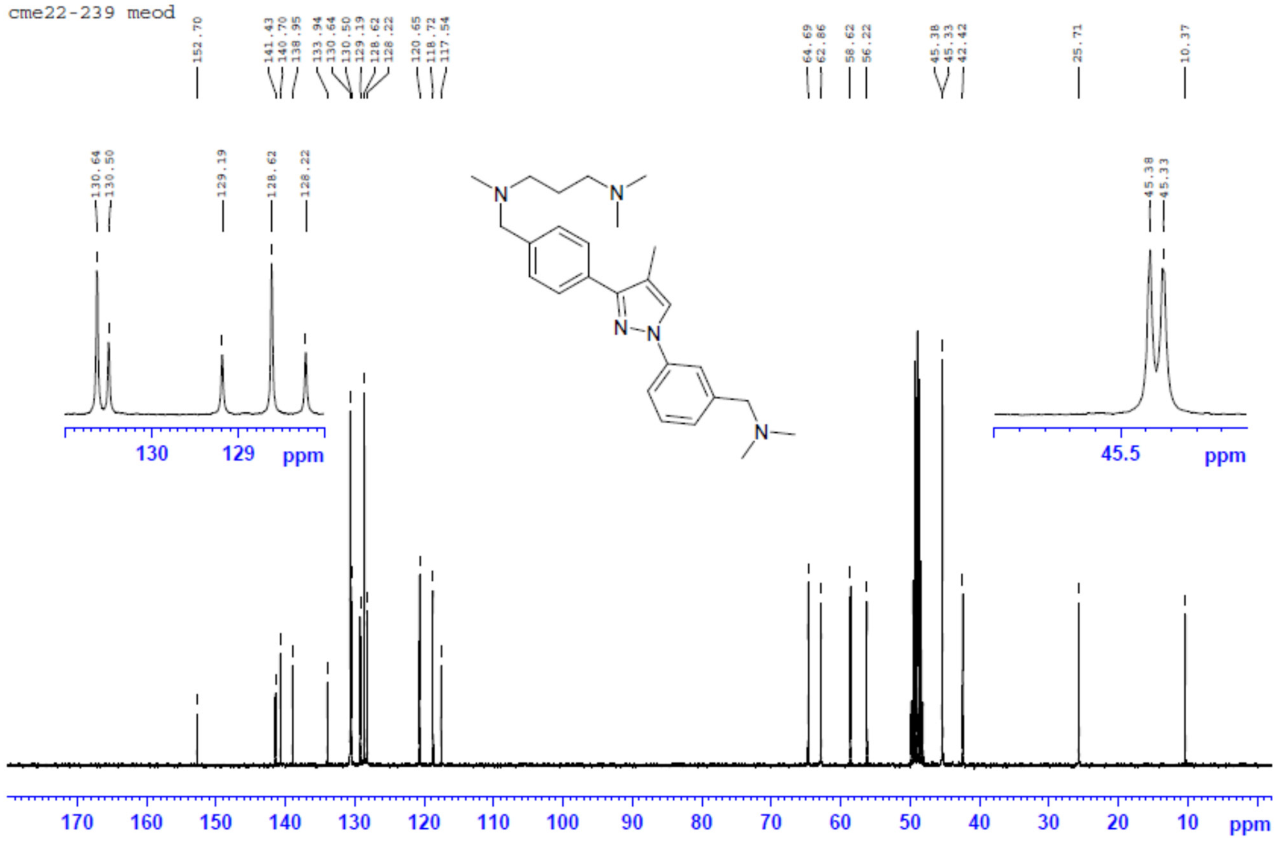

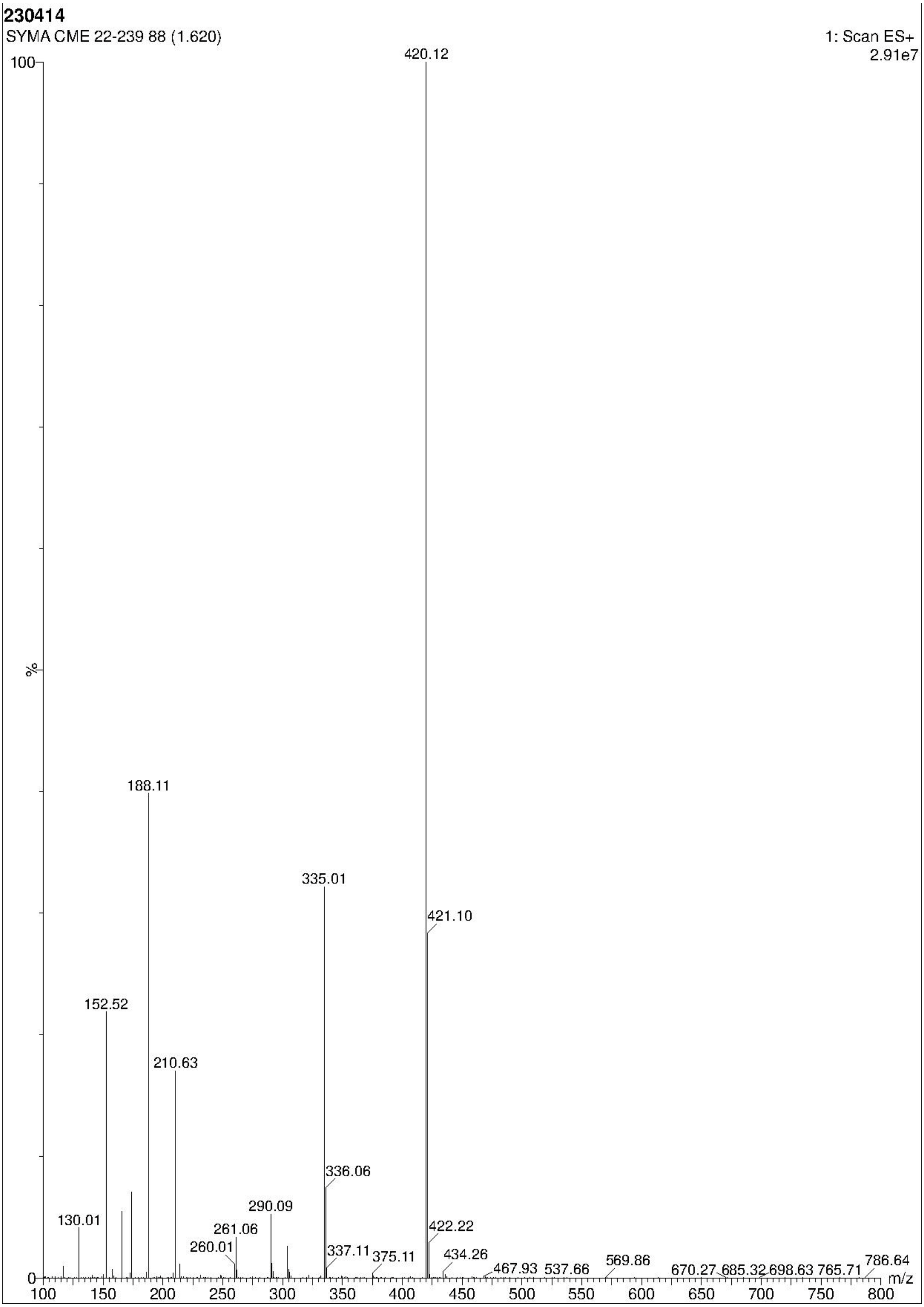
230414
SYMA CME 22-239 88 (1.620)
1: Scan ES+
2.91e7
100
%
0
420.12
188.11
335.01
421.10
152.52
210.63
336.06
290.09
130.01
261.06
422.22
260.01
337.11
375.11
434.26
467.93
537.66
569.86
670.27
685.32
698.63
765.71
786.64
m/z
100
150
200
250
300
350
400
450
500
550
600
650
700
750
800

HPLC C4-column

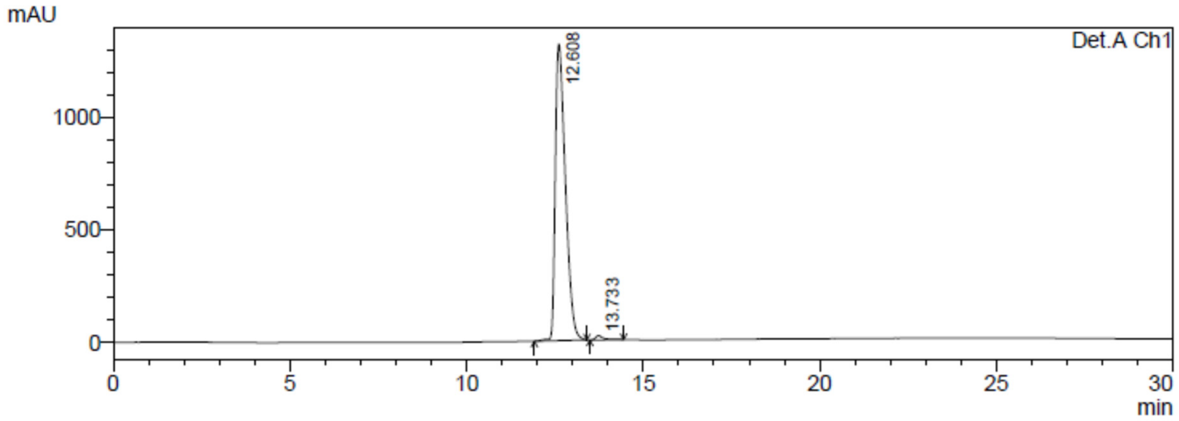

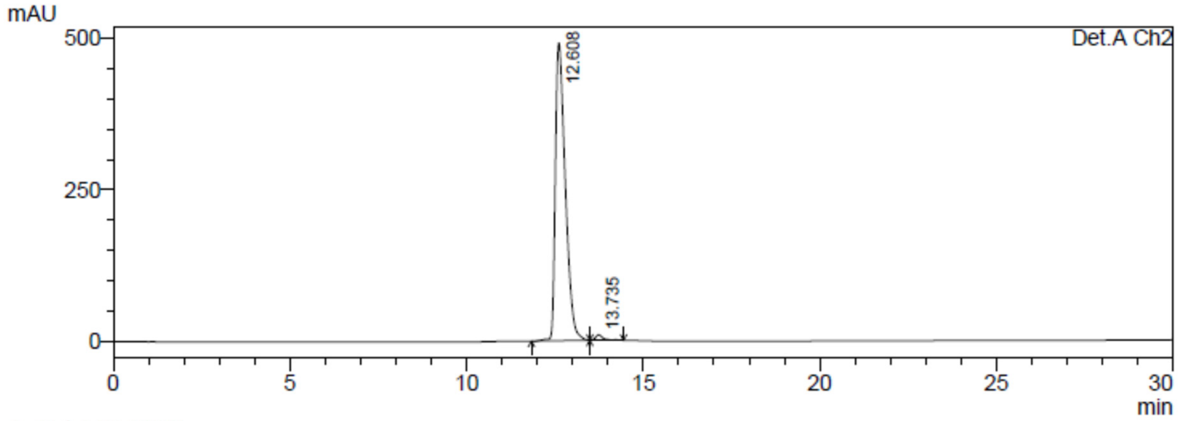

1 Det.A Ch1/215nm
2 Det.A Ch2/254nm

PeakTable

Detector A Ch1 215nm

Peak#	Ret. Time	Area	Area %
1	12.608	26241965	98.887
2	13.733	295439	1.113
Total		26537405	100.000

PeakTable

Detector A Ch2 254nm

Peak#	Ret. Time	Area	Area %
1	12.608	9627091	98.696
2	13.735	127163	1.304
Total		9754254	100.000

HPLC C18-column

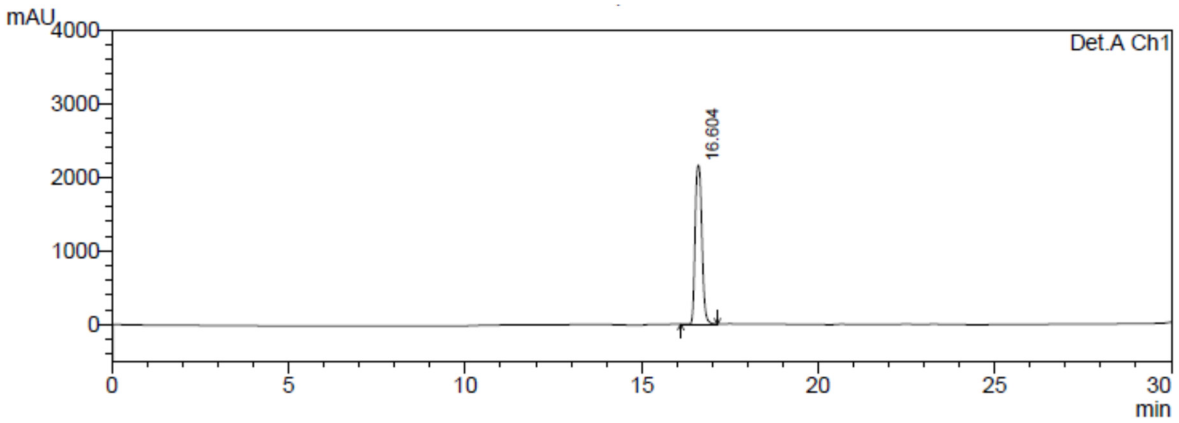

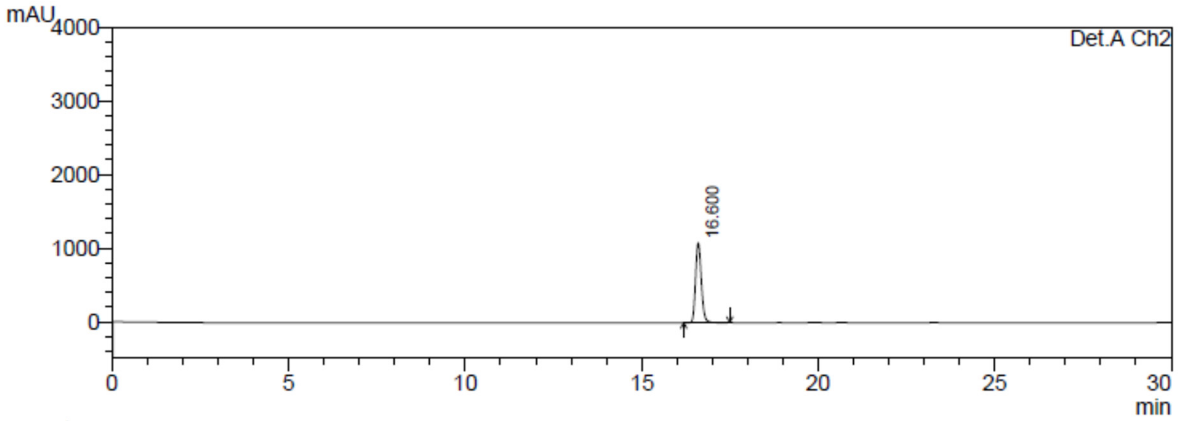

1 Det.A Ch1/215nm
2 Det.A Ch2/254nm

PeakTable

Detector A Ch1 215nm

Peak#	Ret. Time	Area	Area %
1	16.604	28843794	100.000
Total		28843794	100.000

PeakTable

Detector A Ch2 254nm

Peak#	Ret. Time	Area	Area %
1	16.600	12236148	100.000
Total		12236148	100.000

[3-(dimethylamino)propyl](methyl){[4-(4-methyl-1-{3-[(4-methylpiperazin-1-yl)methyl]phenyl}-1*H*-pyrazol-3-yl)phenyl]methyl}amine (**68**)

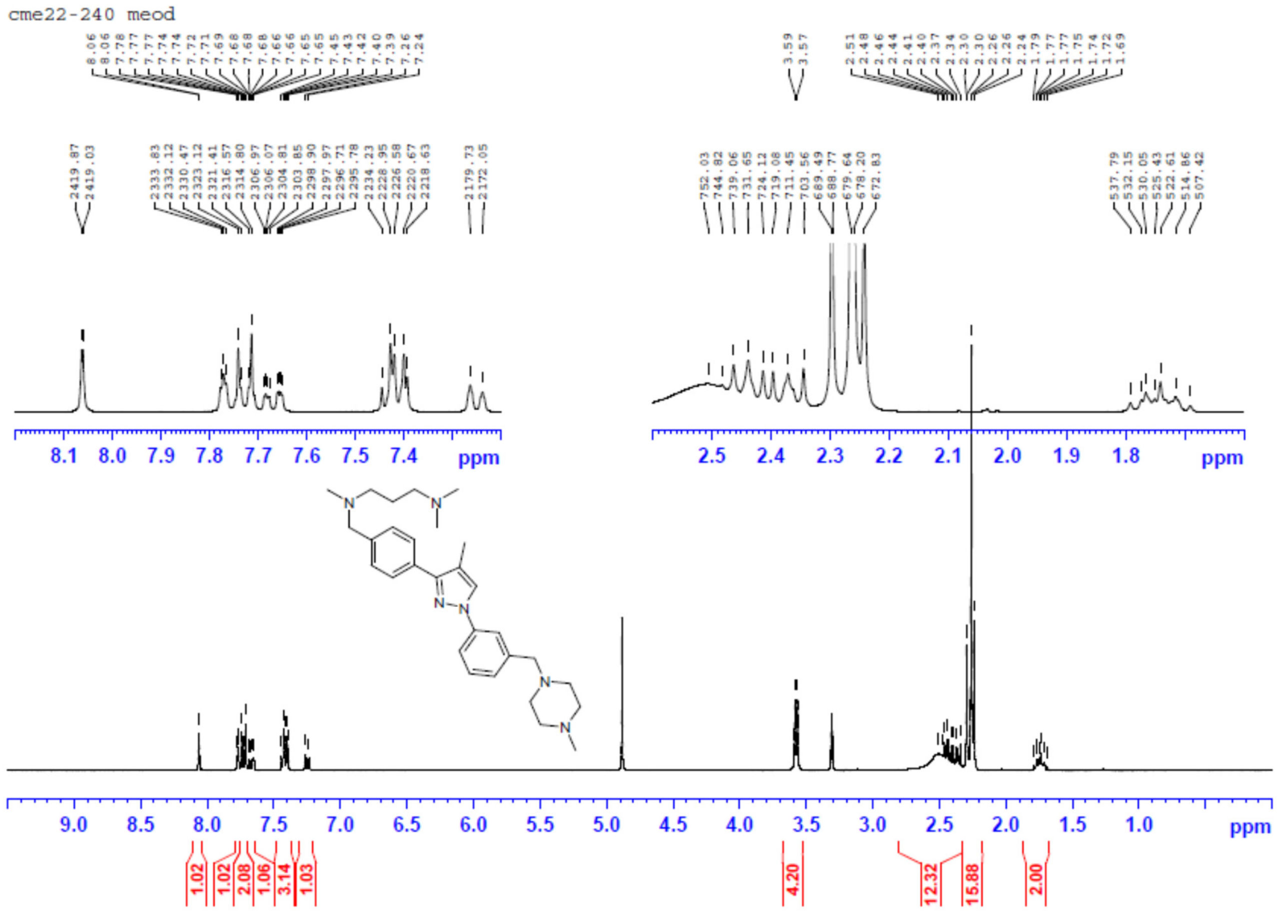

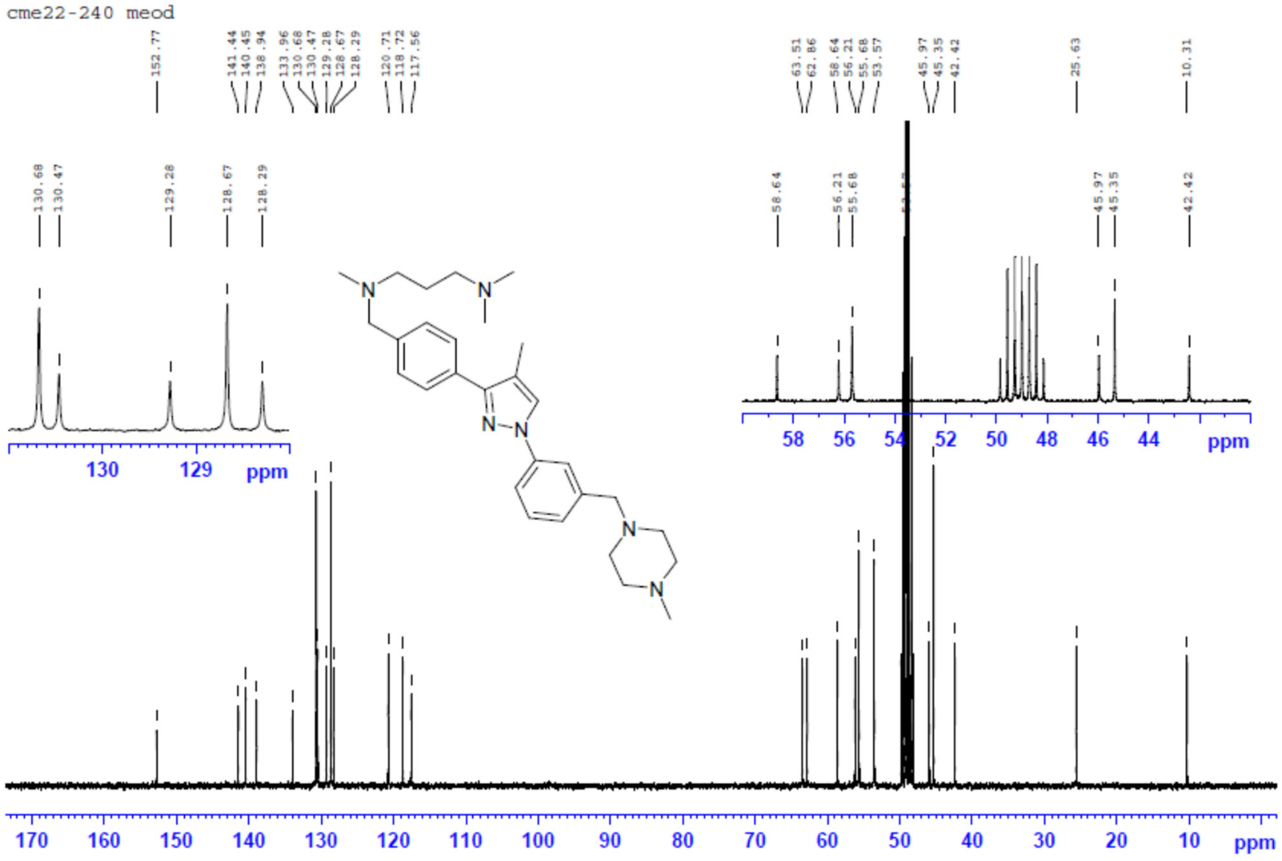

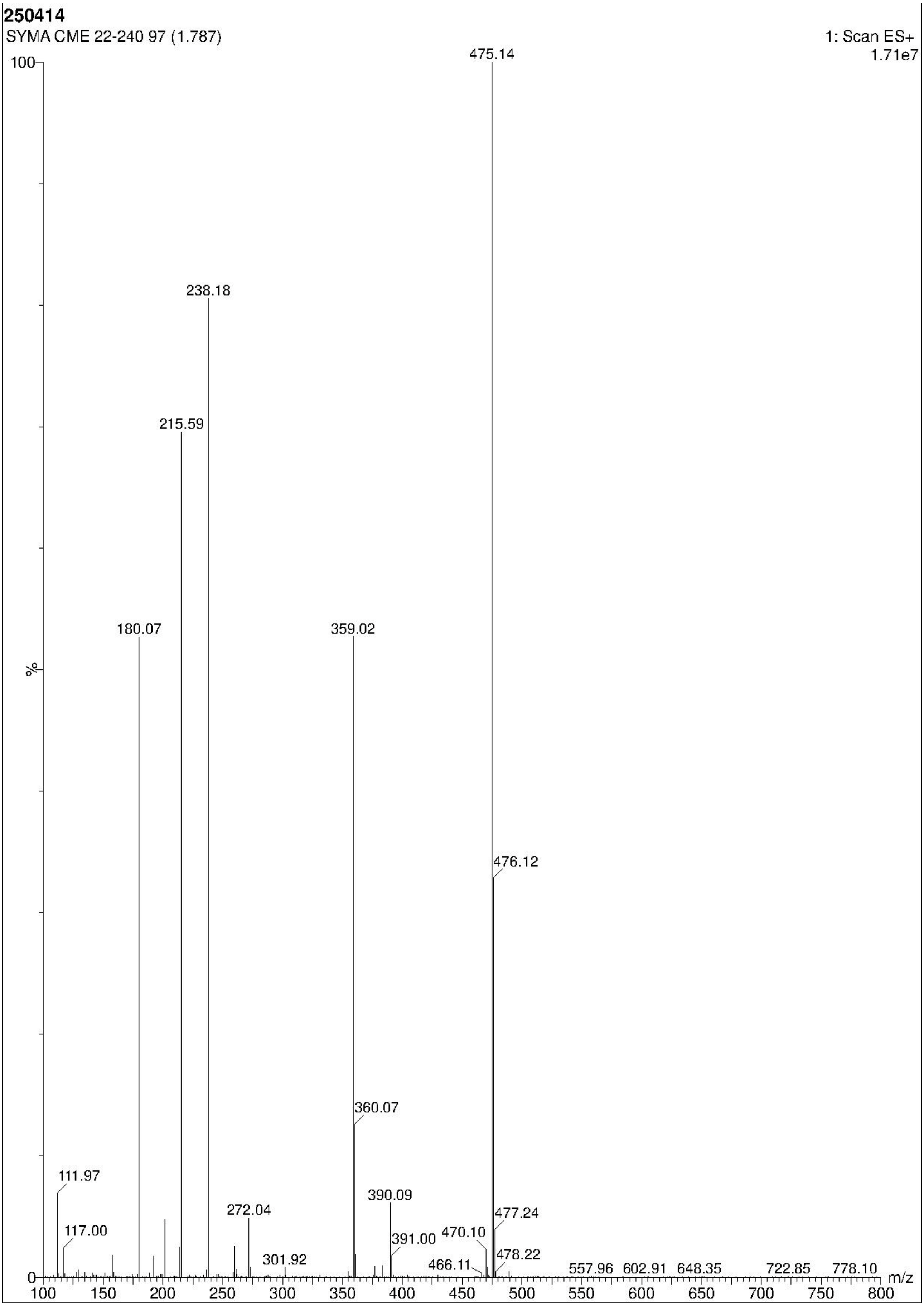
250414
SYMA CME 22-240 97 (1.787)
1: Scan ES+
1.71e7
475.14
238.18
215.59
180.07
359.02
476.12
360.07
111.97
390.09
272.04
117.00
470.10
477.24
391.00
301.92
478.22
466.11
557.96
602.91
648.35
722.85
778.10
%
m/z
100
0
100
150
200
250
300
350
400
450
500
550
600
650
700
750
800

HPLC C4-column

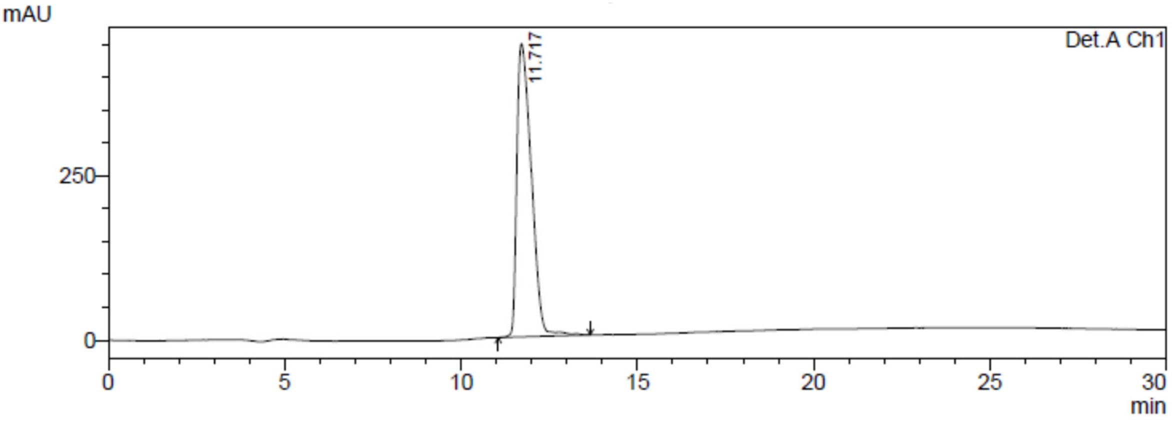

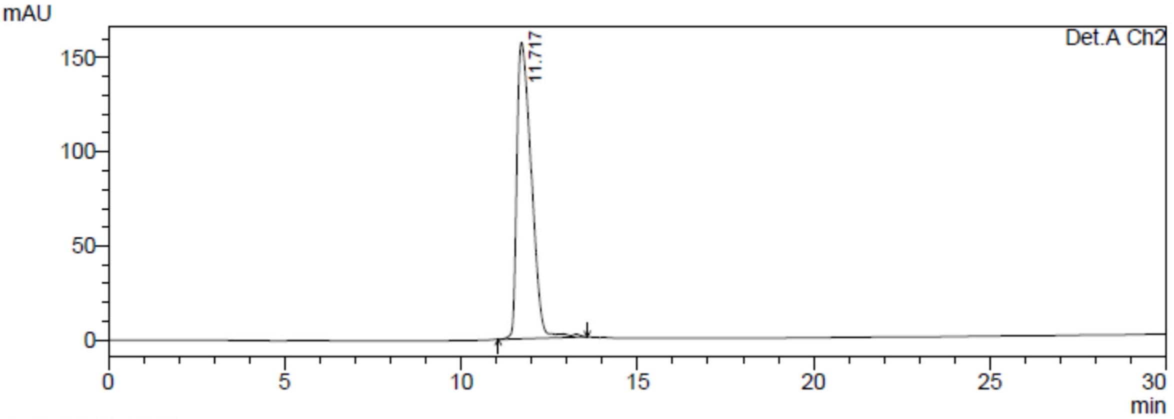

1 Det.A Ch1/215nm
2 Det.A Ch2/254nm

PeakTable
Detector A Ch1 215nm

Peak#	Ret. Time	Area	Area %
1	11.717	12648208	100.000
Total		12648208	100.000

PeakTable
Detector A Ch2 254nm

Peak#	Ret. Time	Area	Area %
1	11.717	4470636	100.000
Total		4470636	100.000

HPLC C18-column

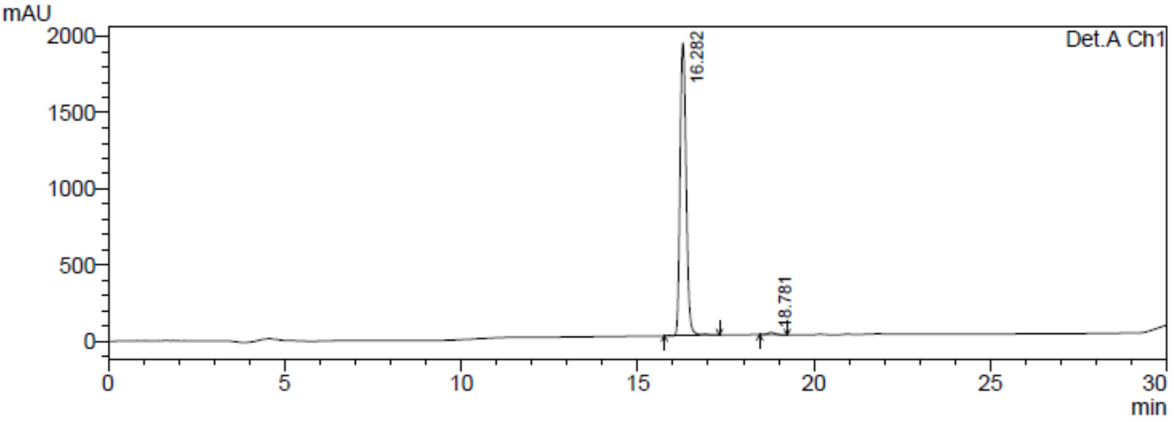

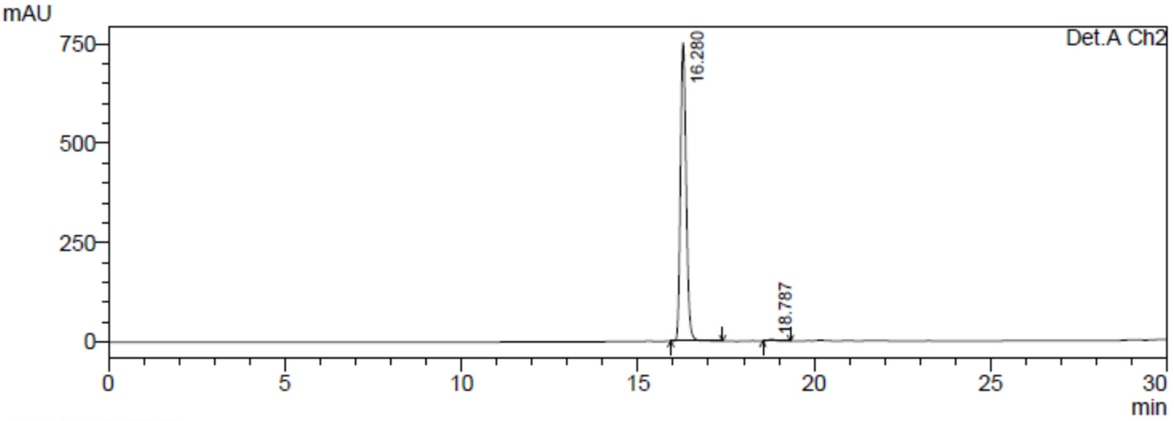

1 Det.A Ch1/215nm
2 Det.A Ch2/254nm

PeakTable

Detector A Ch1 215nm

Peak#	Ret. Time	Area	Area %
1	16.282	23108565	99.168
2	18.781	193957	0.832
Total		23302522	100.000

PeakTable

Detector A Ch2 254nm

Peak#	Ret. Time	Area	Area %
1	16.280	8439637	99.324
2	18.787	57425	0.676
Total		8497062	100.000

[3-(dimethylamino)propyl][(4-{1-[3-({[3-(dimethylamino)propyl](methyl)amino}methyl)phenyl]-4-methyl-1*H*-pyrazol-3-yl}phenyl)methyl]methylamine (**69**)

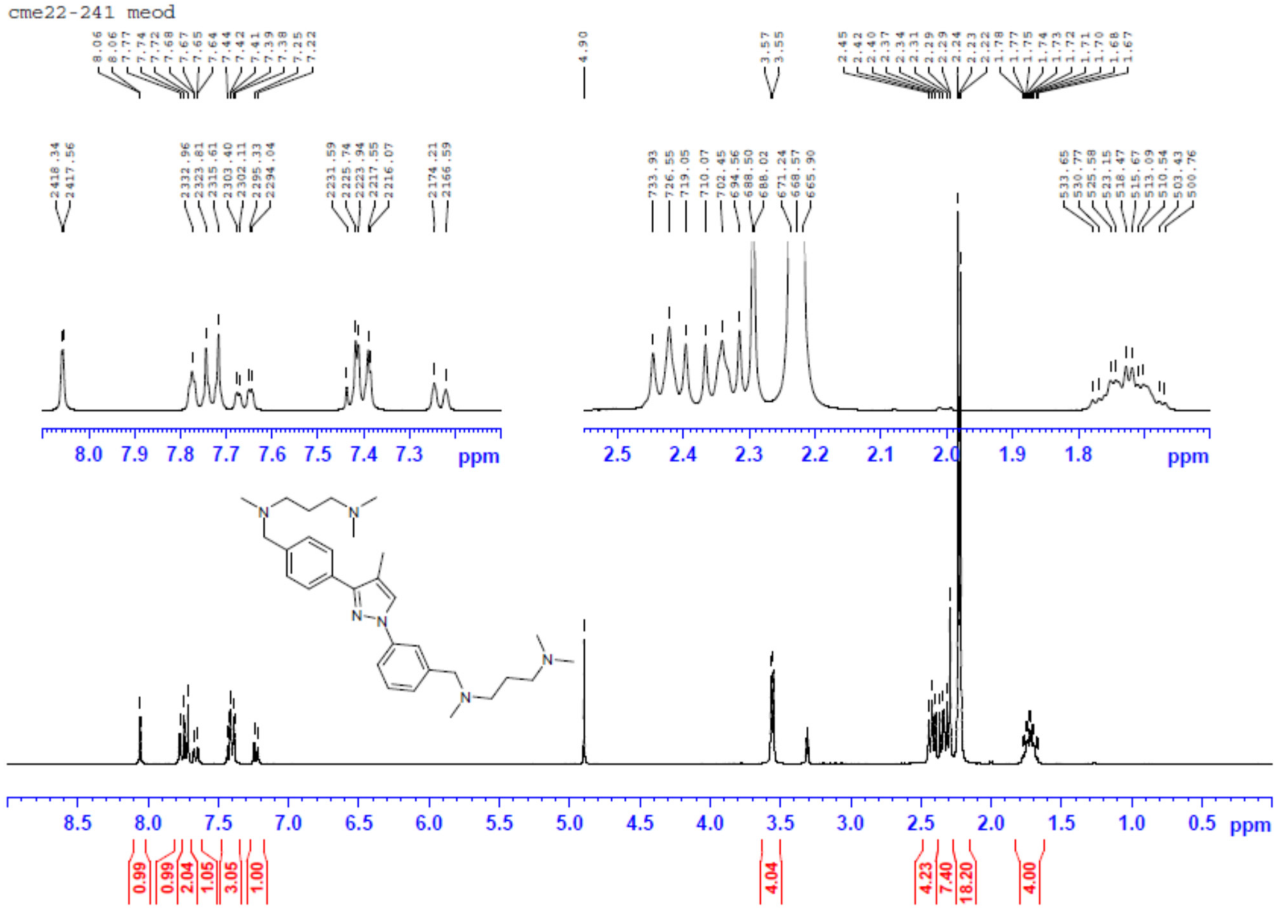

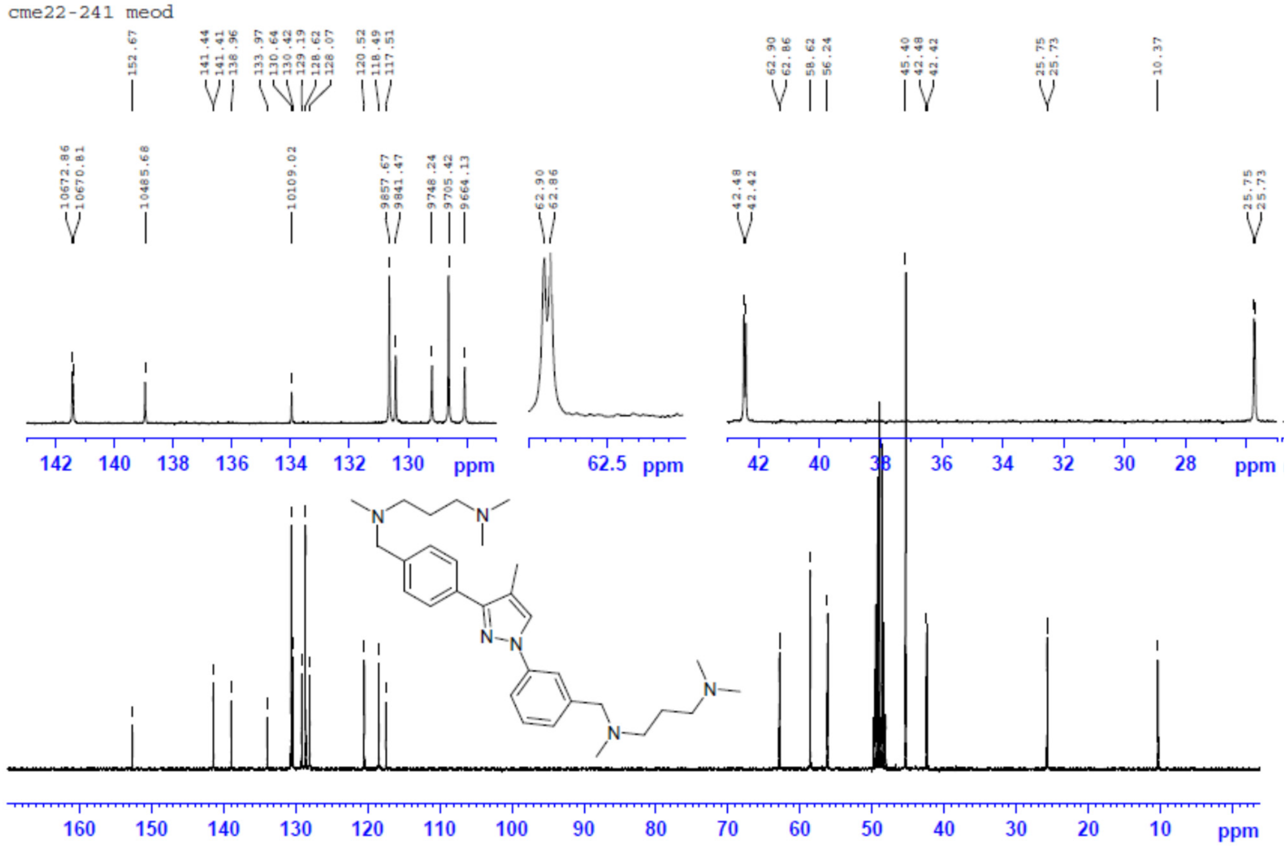

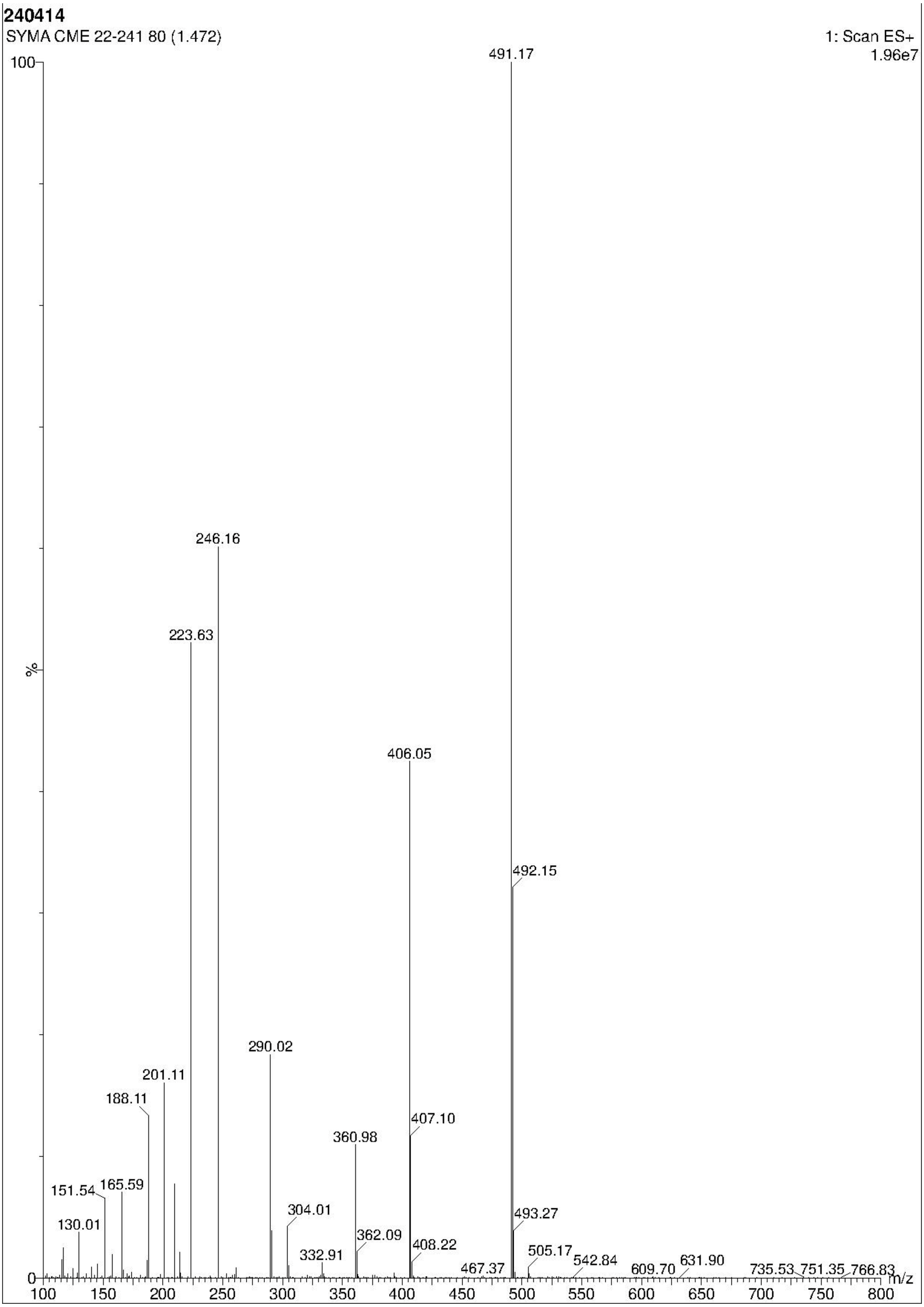

240414
SYMA CME 22-241 80 (1.472)
1: Scan ES+
1.96e7
491.17
246.16
223.63
406.05
492.15
290.02
201.11
188.11
407.10
360.98
151.54
165.59
304.01
130.01
493.27
362.09
332.91
408.22
505.17
542.84
467.37
609.70
631.90
735.53
751.35
766.83
%
m/z
100
0
100 150 200 250 300 350 400 450 500 550 600 650 700 750 800

HPLC C4-column

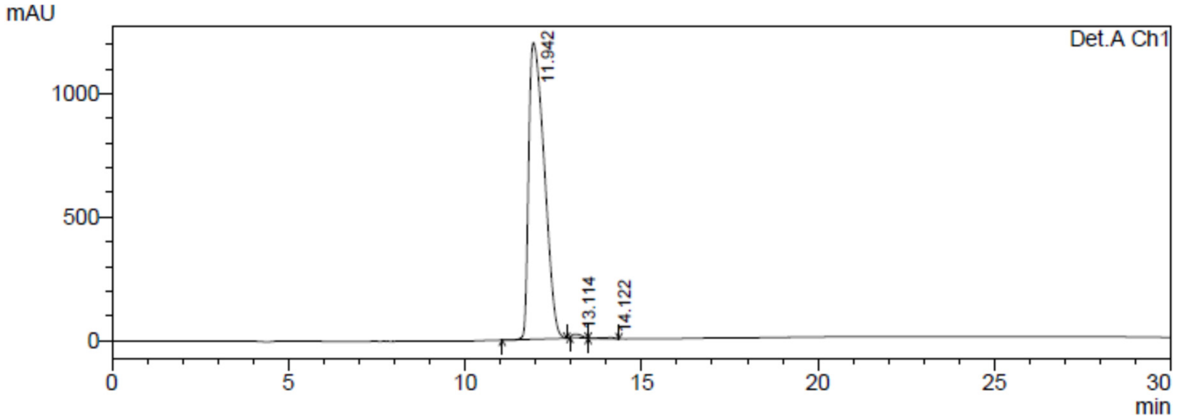

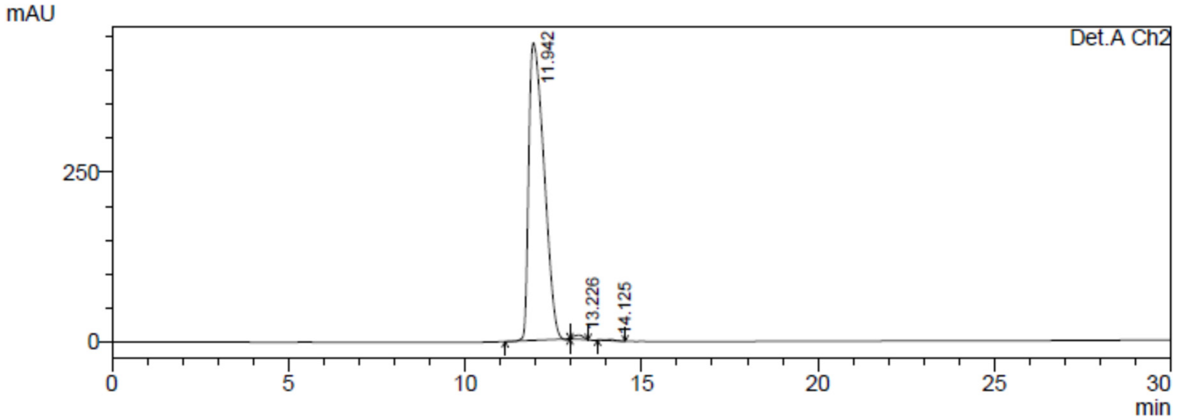

1 Det.A Ch1/215nm
2 Det.A Ch2/254nm

PeakTable

Detector A Ch1 215nm

Peak#	Ret. Time	Area	Area %
1	11.942	36313054	99.068
2	13.114	237083	0.647
3	14.122	104668	0.286
Total		36654805	100.000

PeakTable

Detector A Ch2 254nm

Peak#	Ret. Time	Area	Area %
1	11.942	13003500	98.951
2	13.226	114623	0.872
3	14.125	23294	0.177
Total		13141417	100.000

HPLC C18-column

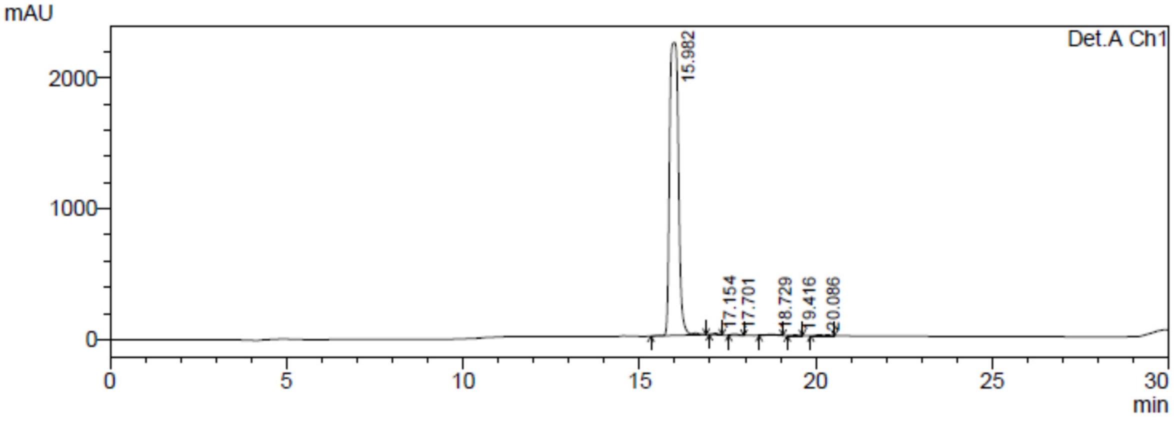

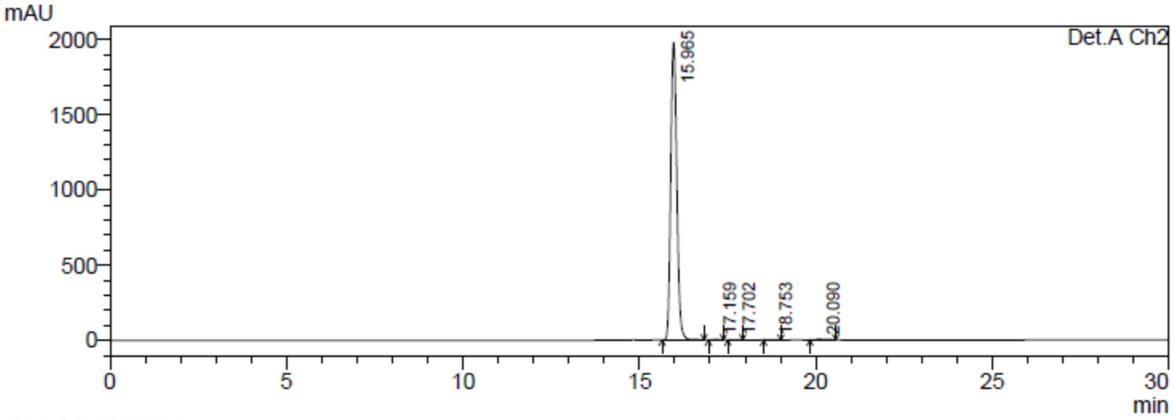

1 Det.A Ch1/215nm
2 Det.A Ch2/254nm

PeakTable

Detector A Ch1 215nm

Peak#	Ret. Time	Area	Area %
1	15.982	39140638	98.491
2	17.154	103380	0.260
3	17.701	111123	0.280
4	18.729	147486	0.371
5	19.416	68637	0.173
6	20.086	168971	0.425
Total		39740236	100.000

PeakTable

Detector A Ch2 254nm

Peak#	Ret. Time	Area	Area %
1	15.965	23840269	98.958
2	17.159	40357	0.168
3	17.702	38624	0.160
4	18.753	28303	0.117
5	20.090	143654	0.596
Total		24091207	100.000

N'-[[3-[1-[3-(dimethylaminomethyl)phenyl]-4-methyl-pyrazol-3-yl]phenyl]methyl]-*N*,*N*,*N'*-trimethyl-propane-1,3-diamine (**70**)

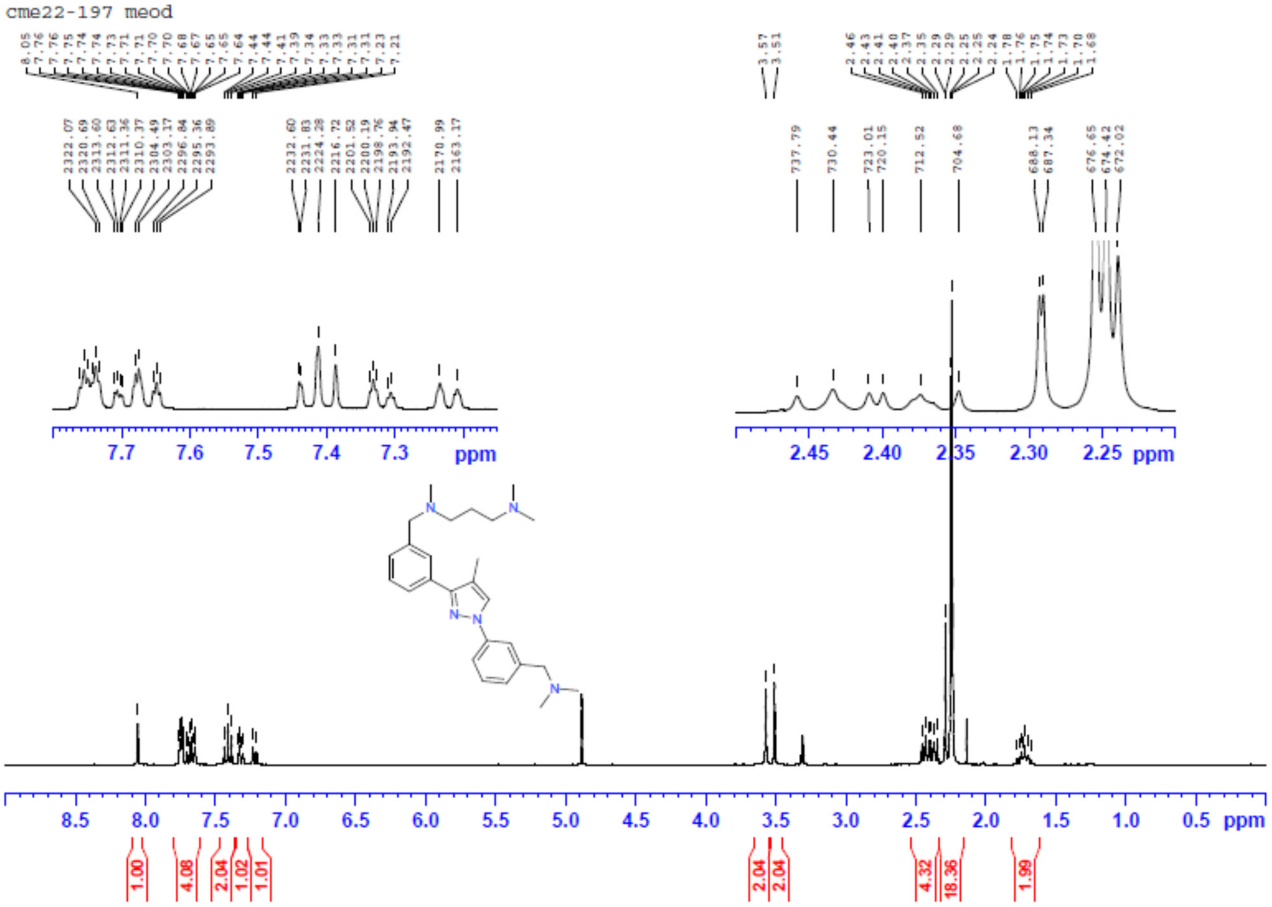

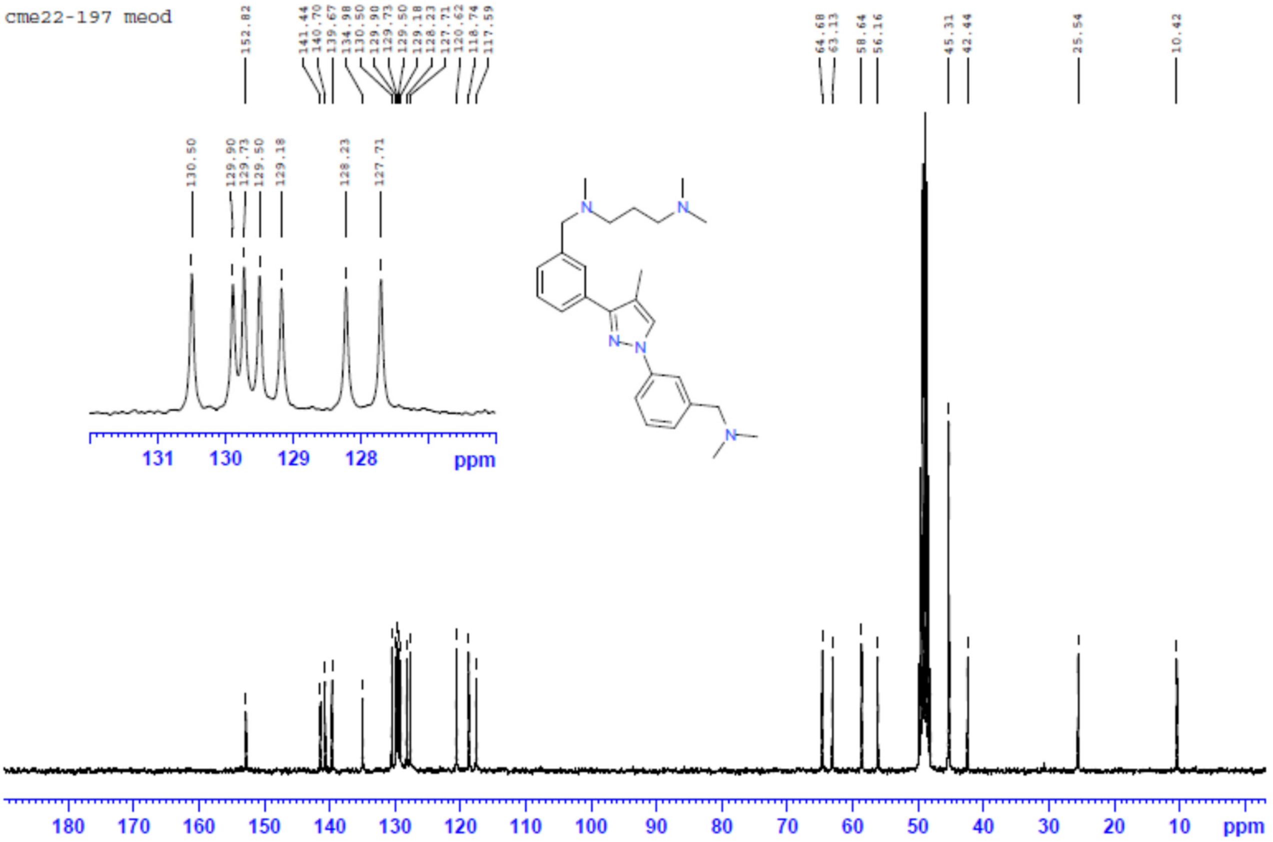

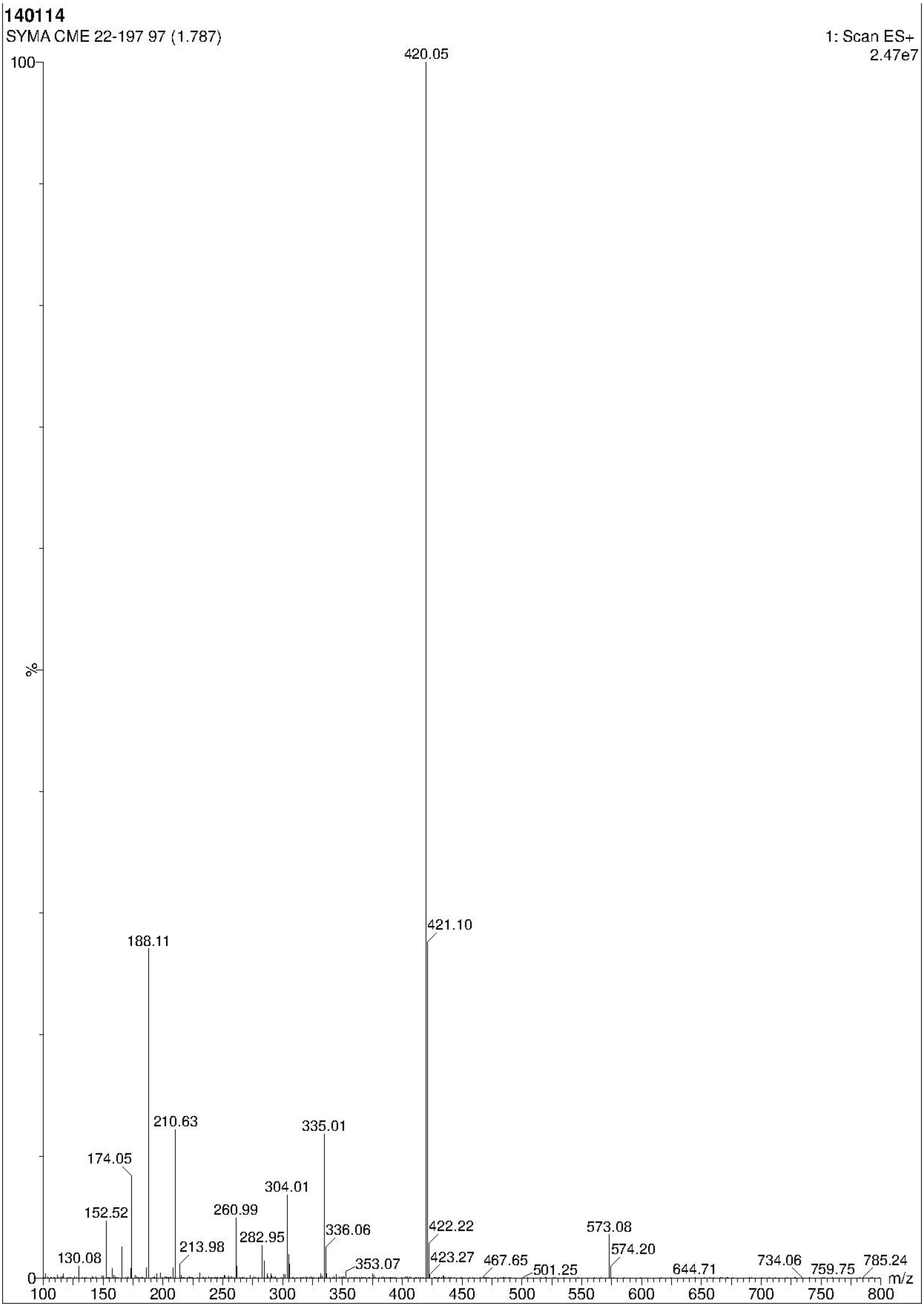

140114
SYMA CME 22-197 97 (1.787)
1: Scan ES+
2.47e7
100
%
0
420.05
421.10
188.11
210.63
335.01
174.05
304.01
152.52
260.99
336.06
422.22
573.08
282.95
574.20
213.98
423.27
130.08
467.65
734.06
785.24
353.07
501.25
644.71
759.75
100
150
200
250
300
350
400
450
500
550
600
650
700
750
800
m/z

HPLC C4-column

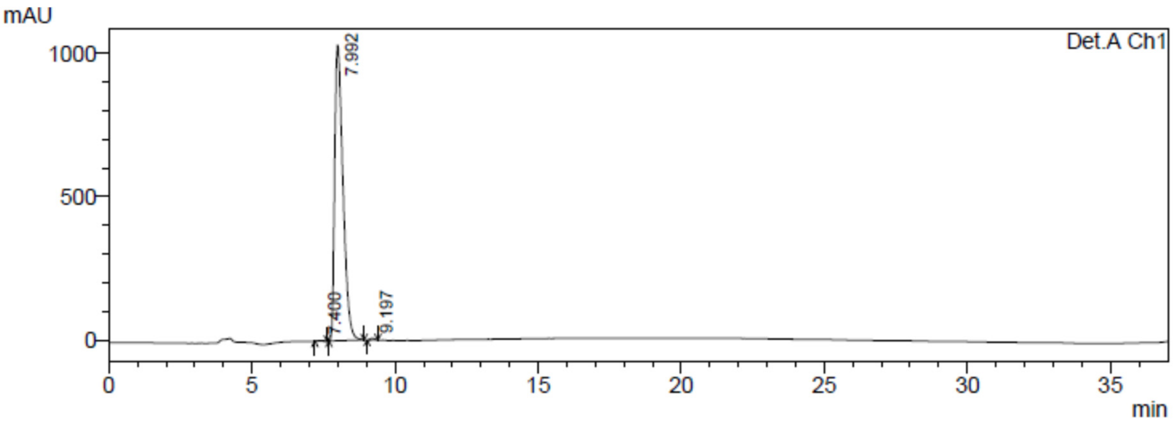

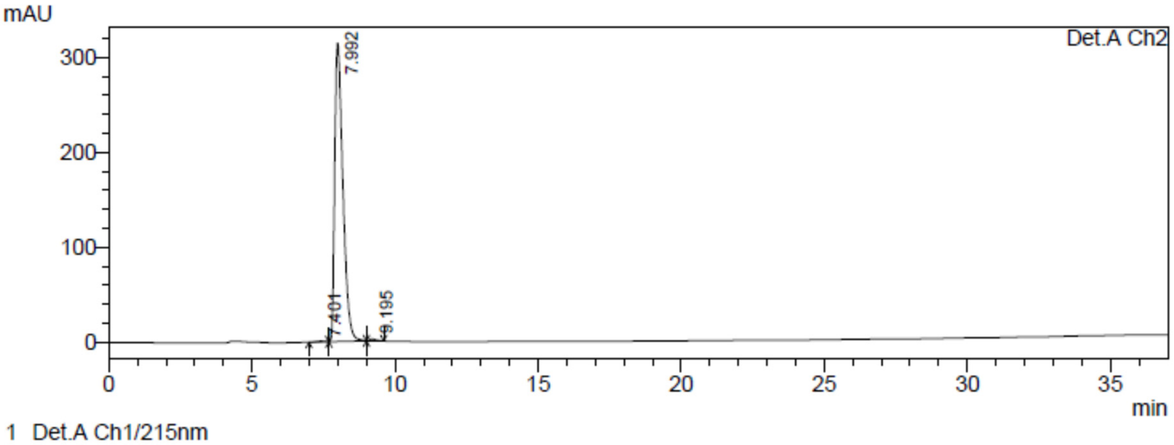

1 Det.A Ch1/215nm
2 Det.A Ch2/254nm

PeakTable

Detector A Ch1 215nm

Peak#	Ret. Time	Area	Area %
1	7.400	34569	0.165
2	7.992	20881833	99.500
3	9.197	70419	0.336
Total		20986821	100.000

PeakTable

Detector A Ch2 254nm

Peak#	Ret. Time	Area	Area %
1	7.401	17833	0.282
2	7.992	6267148	99.248
3	9.195	29642	0.469
Total		6314622	100.000

HPLC C18-column

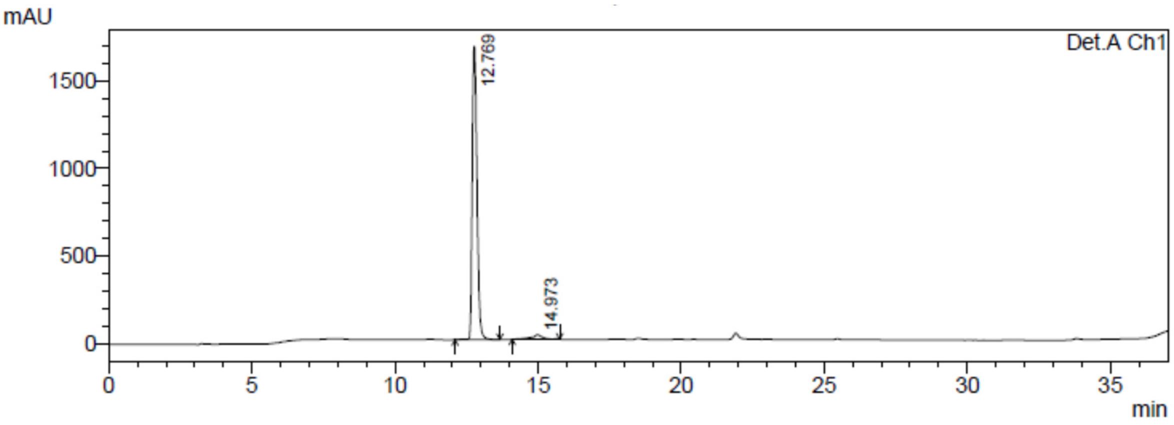

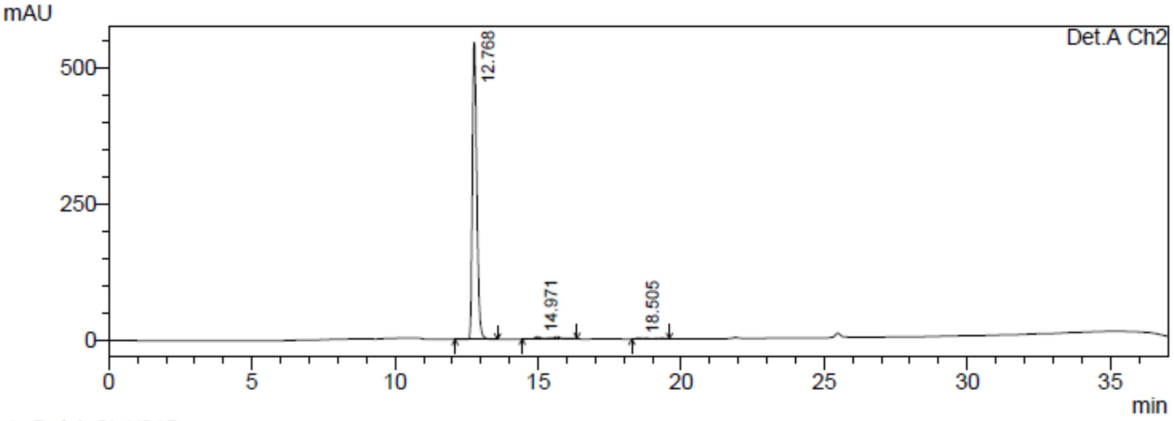

1 Det.A Ch1/215nm
2 Det.A Ch2/254nm

PeakTable
Detector A Ch1 215nm

Peak#	Ret. Time	Area	Area %
1	12.769	19454024	96.729
2	14.973	657870	3.271
Total		20111894	100.000

PeakTable
Detector A Ch2 254nm

Peak#	Ret. Time	Area	Area %
1	12.768	5900947	97.130
2	14.971	122462	2.016
3	18.505	51901	0.854
Total		6075310	100.000

N,*N*,*N'*-trimethyl-*N'*-[[3-[4-methyl-1-[3-[(4-methylpiperazin-1-yl)methyl]phenyl]pyrazol-3-yl]phenyl]methyl]propane-1,3-diamine (**71**)

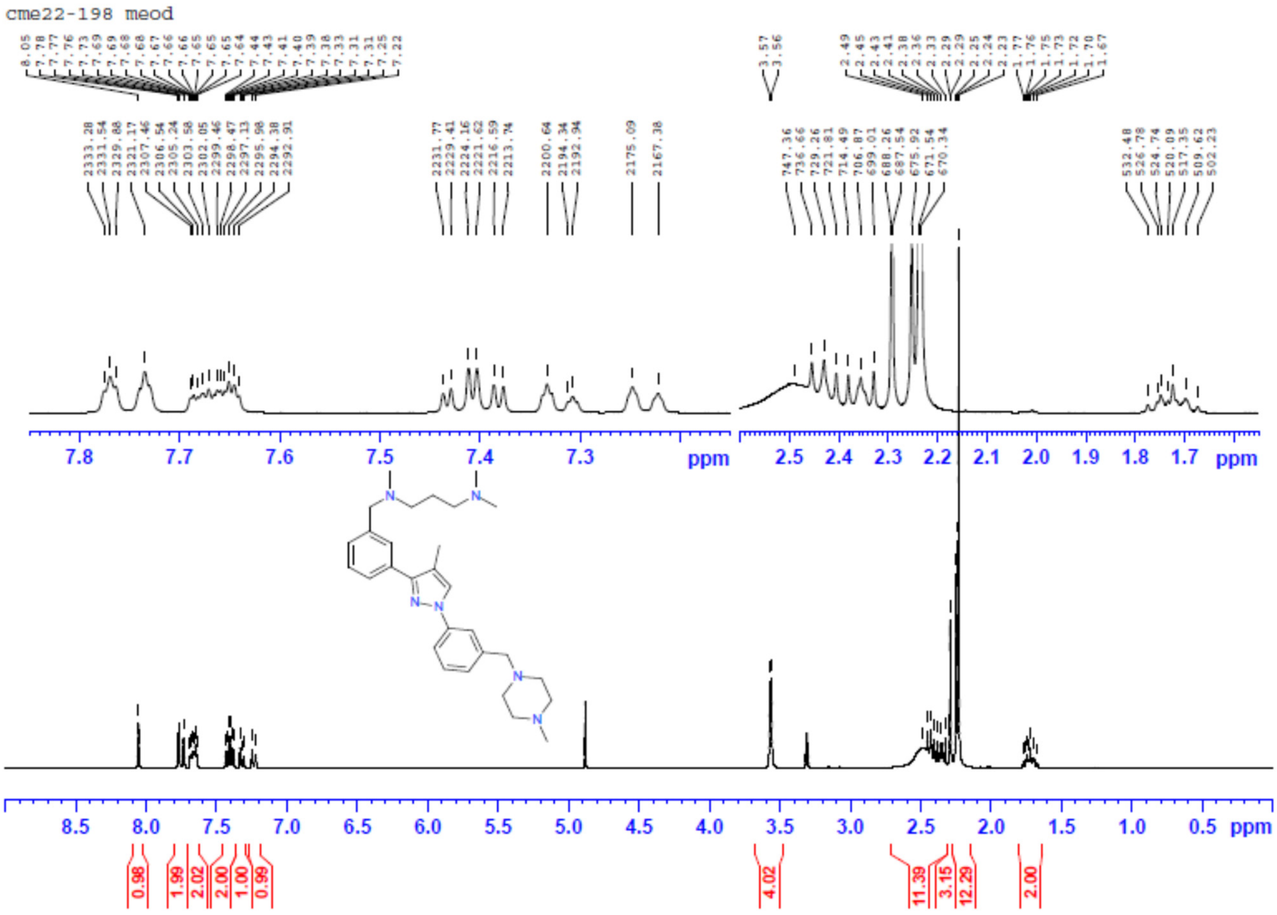

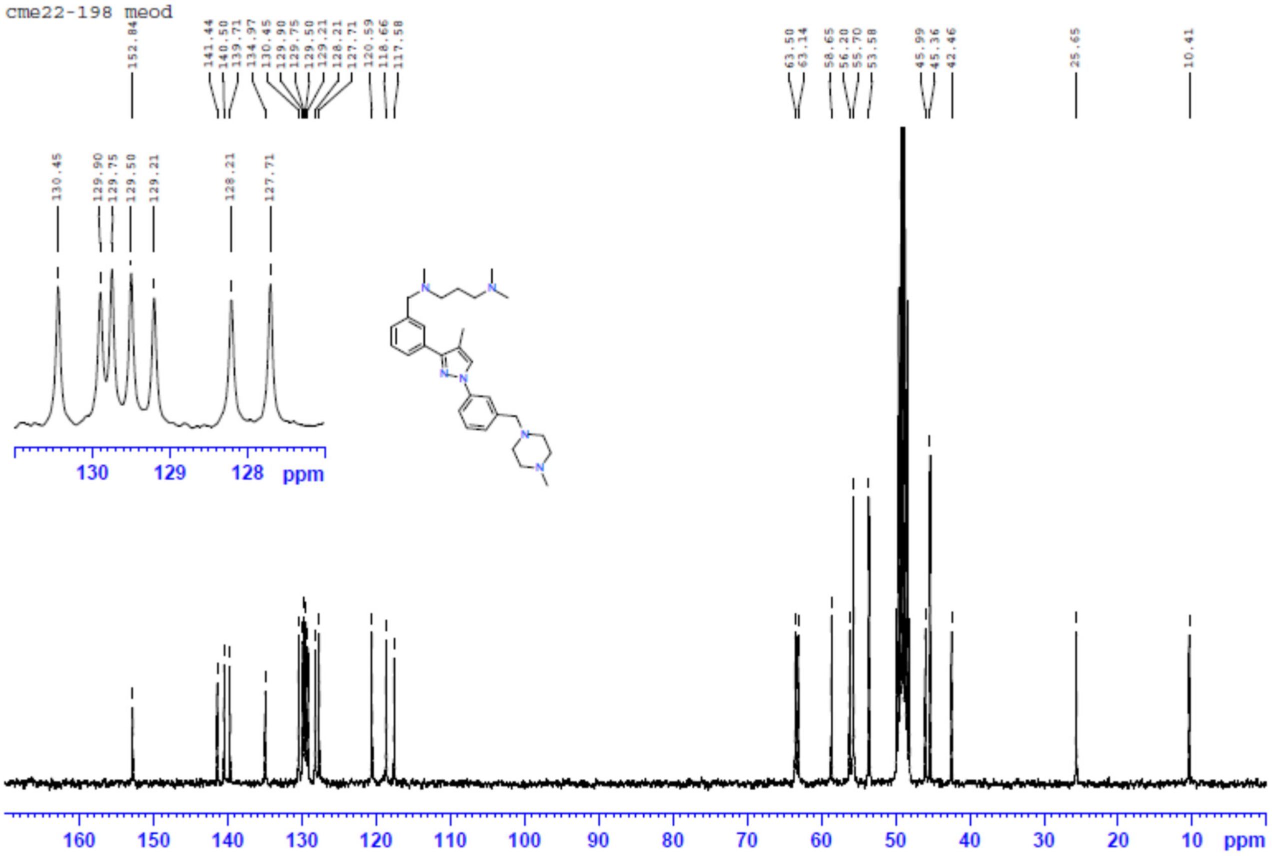

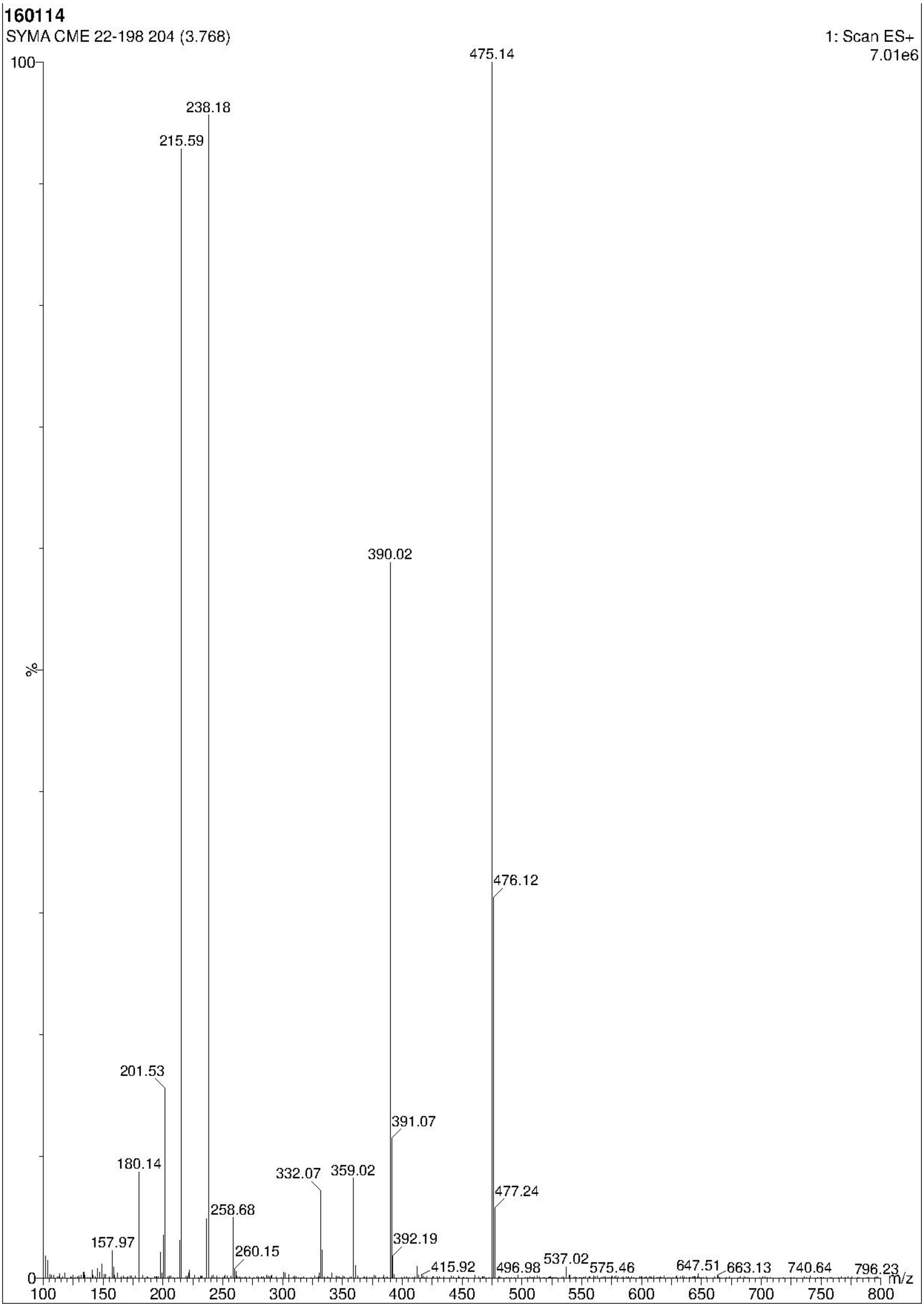
160114
SYMA CME 22-198 204 (3.768)
1: Scan ES+
7.01e6
475.14
238.18
215.59
390.02
476.12
201.53
391.07
180.14
332.07
359.02
477.24
258.68
157.97
392.19
260.15
415.92
496.98
537.02
575.46
647.51
663.13
740.64
796.23
%
m/z
100
0
100 150 200 250 300 350 400 450 500 550 600 650 700 750 800

HPLC C4-column

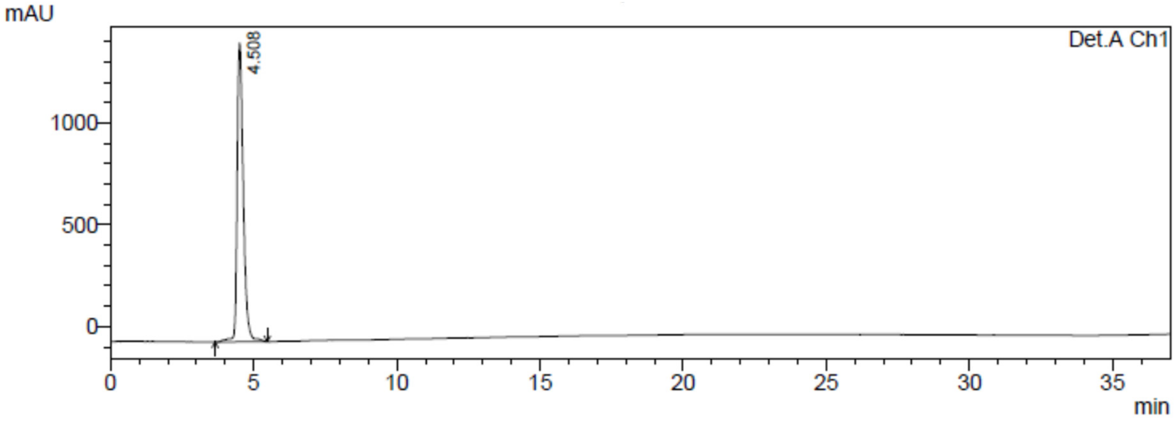

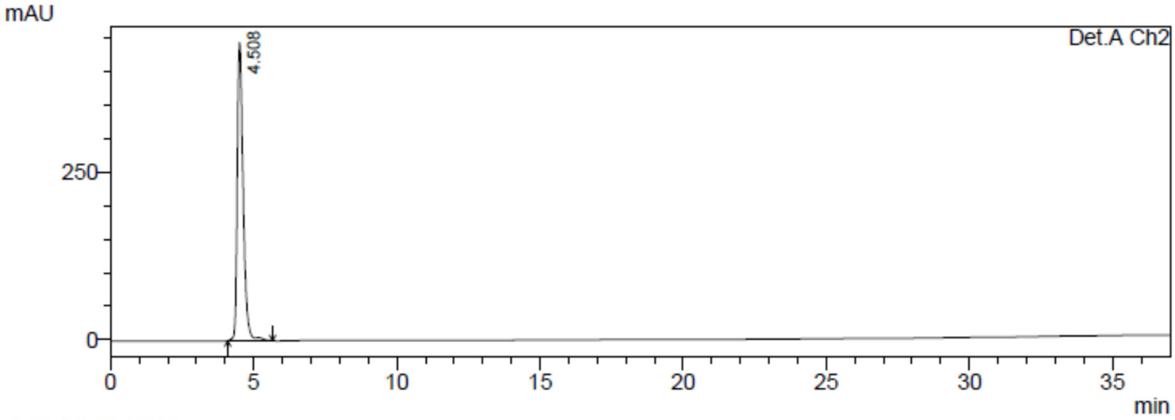

1 Det.A Ch1/215nm
2 Det.A Ch2/254nm

PeakTable
Detector A Ch1 215nm

Peak#	Ret. Time	Area	Area %
1	4.508	22018518	100.000
Total		22018518	100.000

PeakTable
Detector A Ch2 254nm

Peak#	Ret. Time	Area	Area %
1	4.508	6395459	100.000
Total		6395459	100.000

HPLC C18-column

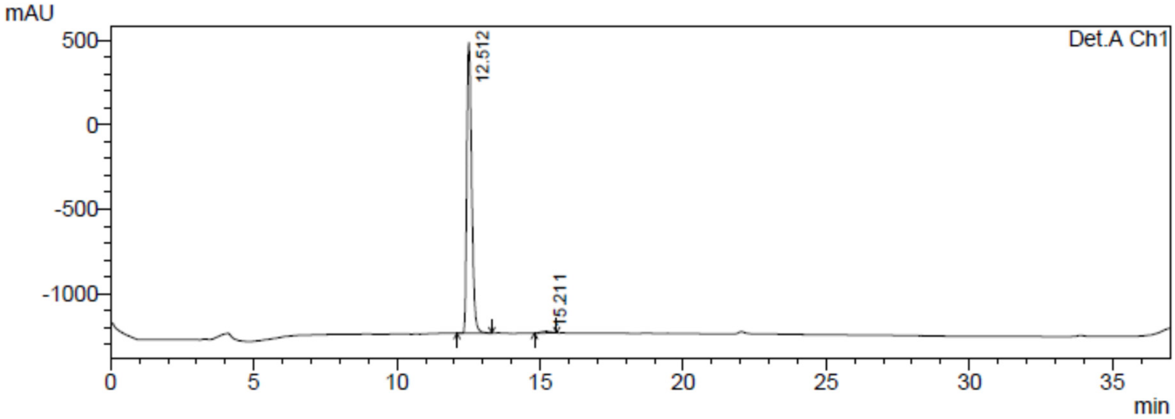

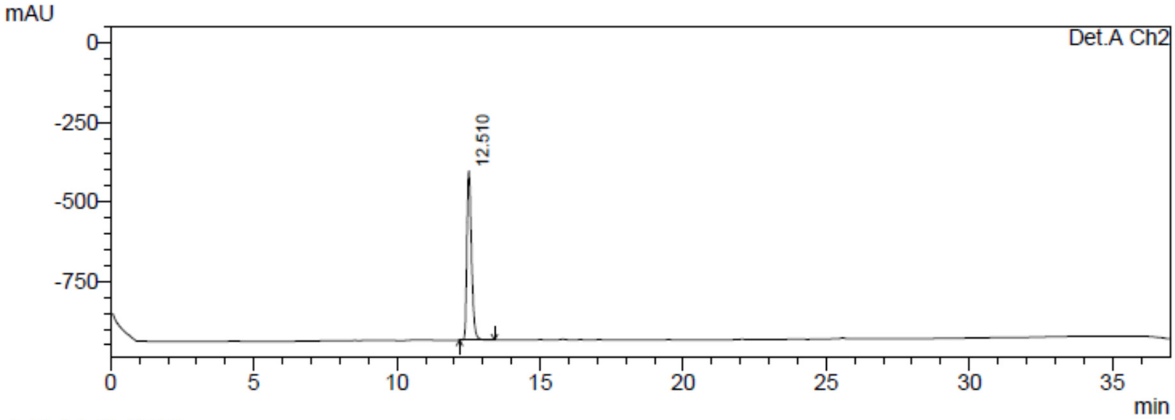

1 Det.A Ch1/215nm
2 Det.A Ch2/254nm

PeakTable

Detector A Ch1 215nm

Peak#	Ret. Time	Area	Area %
1	12.512	20647524	99.012
2	15.211	205958	0.988
Total		20853482	100.000

PeakTable

Detector A Ch2 254nm

Peak#	Ret. Time	Area	Area %
1	12.510	6025867	100.000
Total		6025867	100.000

N'-[[3-[1-[3-[[3-(dimethylamino)propyl-methyl-amino]methyl]phenyl]-4-methyl-pyrazol-3-yl]phenyl]methyl]-*N*,*N*,*N'*-trimethyl-propane-1,3-diamine (**72**)

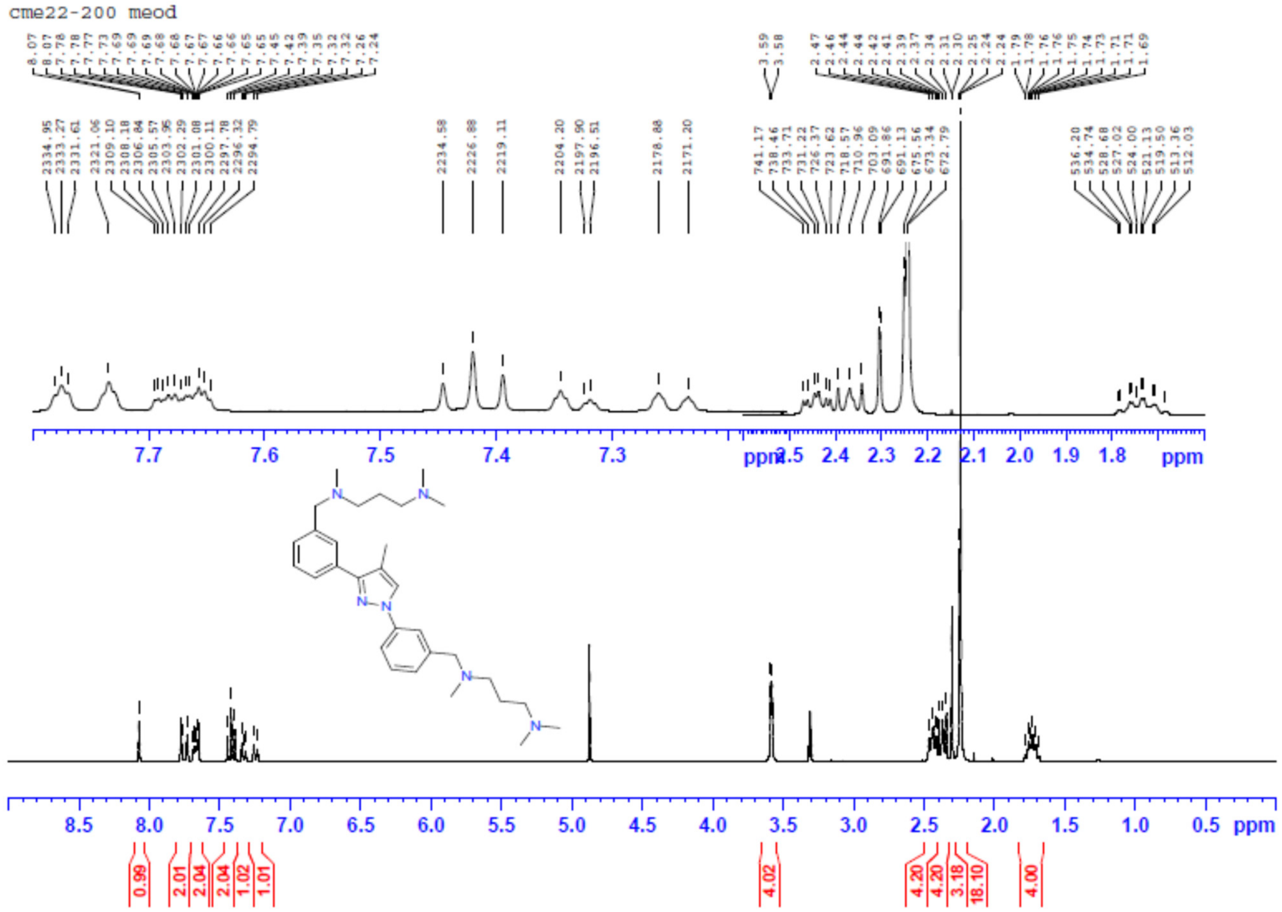

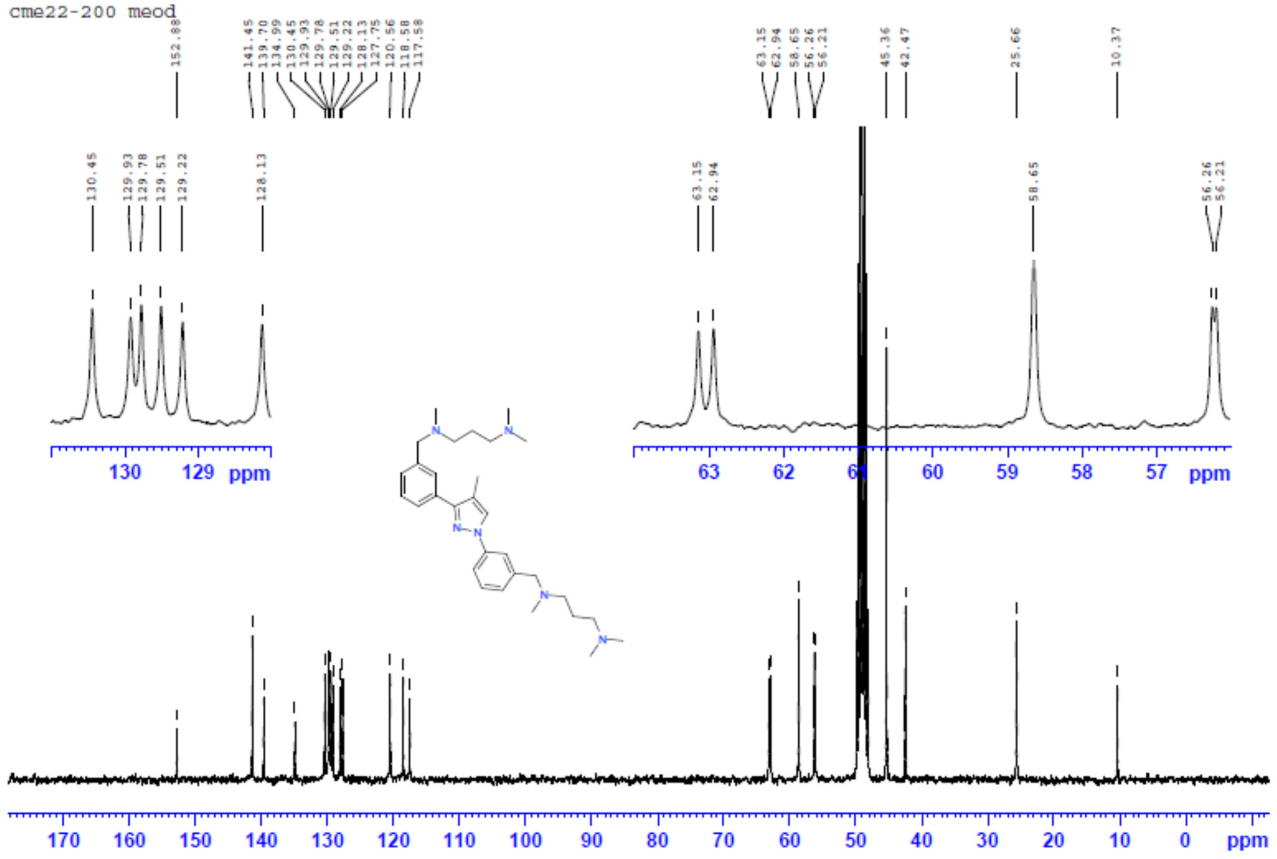

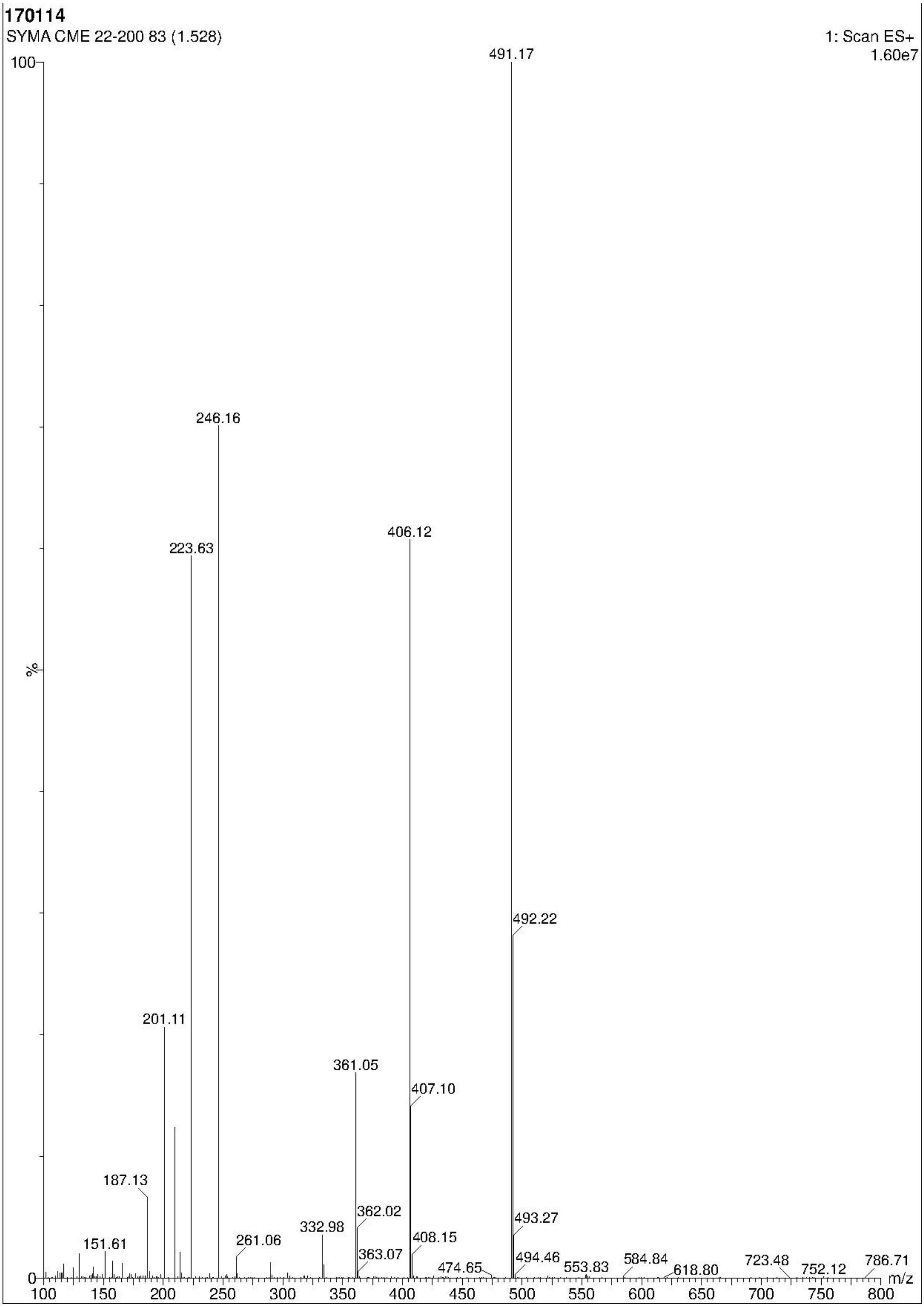
170114
SYMA CME 22-200 83 (1.528)
1: Scan ES+
1.60e7
100
%
0
491.17
246.16
223.63
406.12
492.22
201.11
361.05
407.10
187.13
362.02
493.27
332.98
408.15
151.61
261.06
363.07
494.46
474.65
553.83
584.84
618.80
723.48
752.12
786.71
m/z
100
150
200
250
300
350
400
450
500
550
600
650
700
750
800

HPLC C4-column

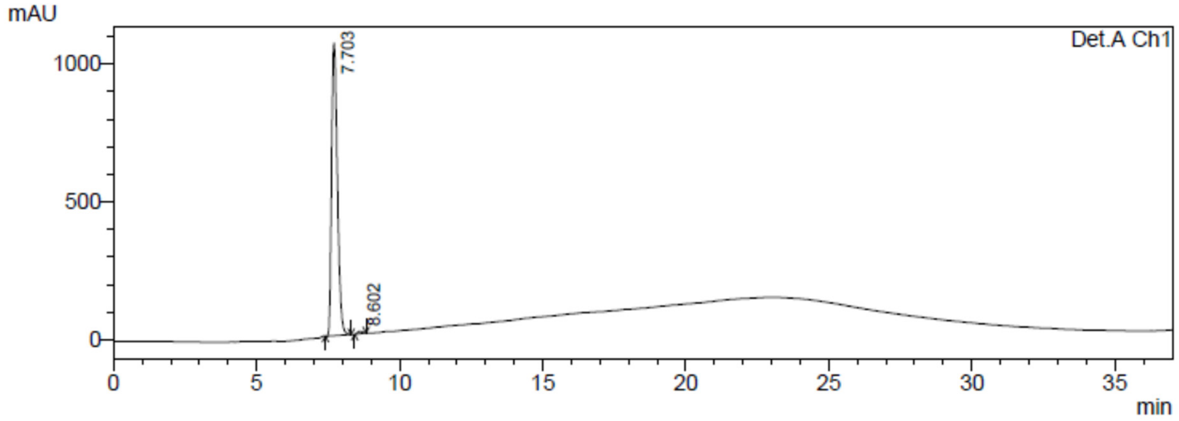

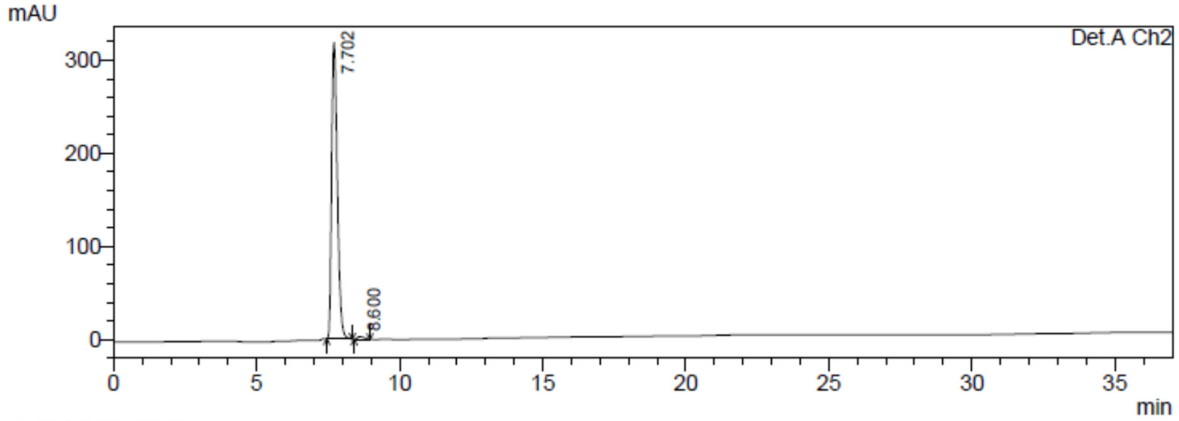

1 Det.A Ch1/215nm
2 Det.A Ch2/254nm

PeakTable

Detector A Ch1 215nm

Peak#	Ret. Time	Area	Area %
1	7.703	14933616	99.243
2	8.602	113871	0.757
Total		15047488	100.000

PeakTable

Detector A Ch2 254nm

Peak#	Ret. Time	Area	Area %
1	7.702	4423294	99.000
2	8.600	44690	1.000
Total		4467985	100.000

HPLC C18-column

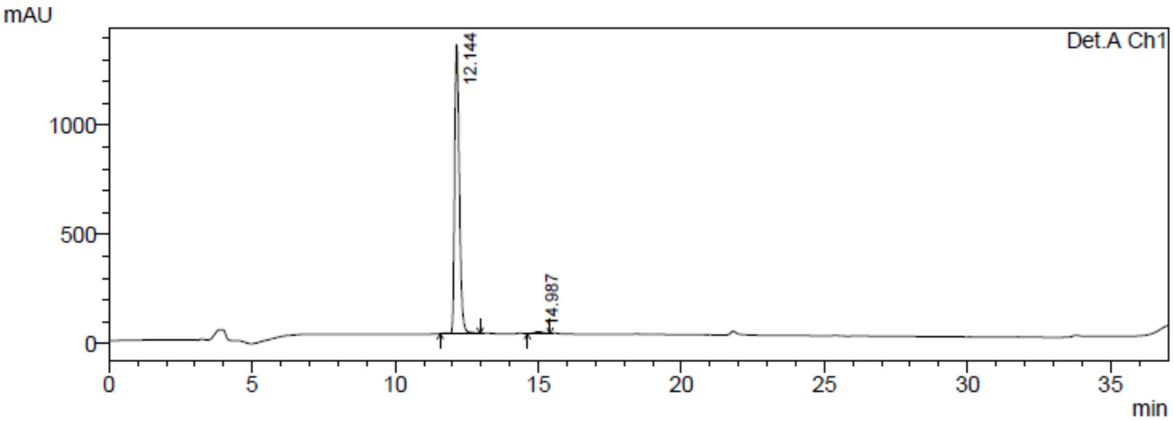

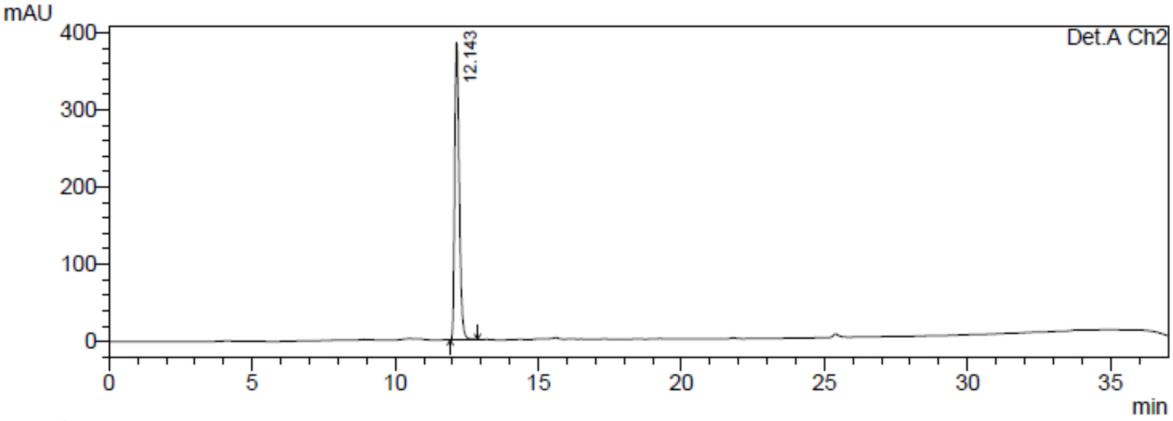

1 Det.A Ch1/215nm
2 Det.A Ch2/254nm

PeakTable

Detector A Ch1 215nm

Peak#	Ret. Time	Area	Area %
1	12.144	15058312	98.912
2	14.987	165609	1.088
Total		15223922	100.000

PeakTable

Detector A Ch2 254nm

Peak#	Ret. Time	Area	Area %
1	12.143	4248846	100.000
Total		4248846	100.000
